# Cationic, Iodine(III)‐Mediated and Directed Diastereoselective Oxidation of Inert C−H Bonds in Cyclic Hydrocarbons

**DOI:** 10.1002/anie.202421872

**Published:** 2025-03-06

**Authors:** Nicolas G.‐Simonian, Bogdan R. Brutiu, Daniel Kaiser, Nuno Maulide

**Affiliations:** ^1^ Institute of Organic Chemistry University of Vienna Währinger Straße 38 1090 Vienna Austria

**Keywords:** oxidation, C−H activation, carbocations, stereoselective synthesis, hypervalent iodine

## Abstract

Over the past three decades, the functionalization of unactivated C−H bonds has become a workhorse of synthesis. In the field of C−H bond oxygenation, most established methods hinge on single‐electron reactivity, encountering challenges pertaining to regio‐ and/or stereocontrol. Herein, we describe a conceptually distinct strategy relying on the unique features of carbocation chemistry. Our iodine(III)‐mediated method achieves the diastereoselective oxygenation of remote C−H bonds at traditionally unreactive sites and enables late‐stage functionalization at steroidal frameworks as well as an unusual chirality relay.

## Graphical abstract

An I(III)‐mediated, selective oxidation of remote C−H bonds is reported. This approach relies on the generation of a destabilized carbocation that is coerced into a predetermined position through charge relocation. In‐depth experiments give insight into the mechanistic intricacies of this highly diastereoselective transformation.

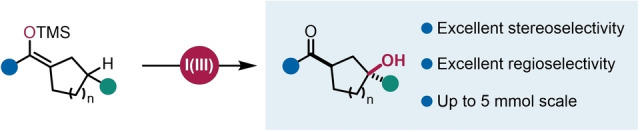

The direct functionalization of organic compounds, the predominant bond type of which are strong and unreactive C−H bonds, is arguably one of the most appealing strategies to improve the efficiency of synthetic processes through the avoidance of functional‐group interconversion.[^1^, ^2^] Among the methods to modify C−H bonds, oxygenation (i.e., the formation of a C−O bond from an effectively inert C−H bond) has blossomed over the last decades as a promising path to elicit reactivity at otherwise poorly reactive positions.[^3^, ^4^]

While the harnessing of metal bound, formal carbanions for C−H oxygenation has been reported,[^5^, ^6^, ^7^, ^8^] oxidative generation of alkyl radicals by hydrogen‐atom abstraction has been the most frequently deployed paradigm in this context (Scheme 1A). The high bond‐dissociation energy of C(sp^3^)−H bonds (up to 104.9 kcal/mol)^[9]^ however, mandates the use of strong oxidants, which often act indiscriminately at several positions, resulting in a lack of regio‐ and stereoselectivity (see inset in Scheme 1A).[^10^, ^11^]

**Scheme 1:**
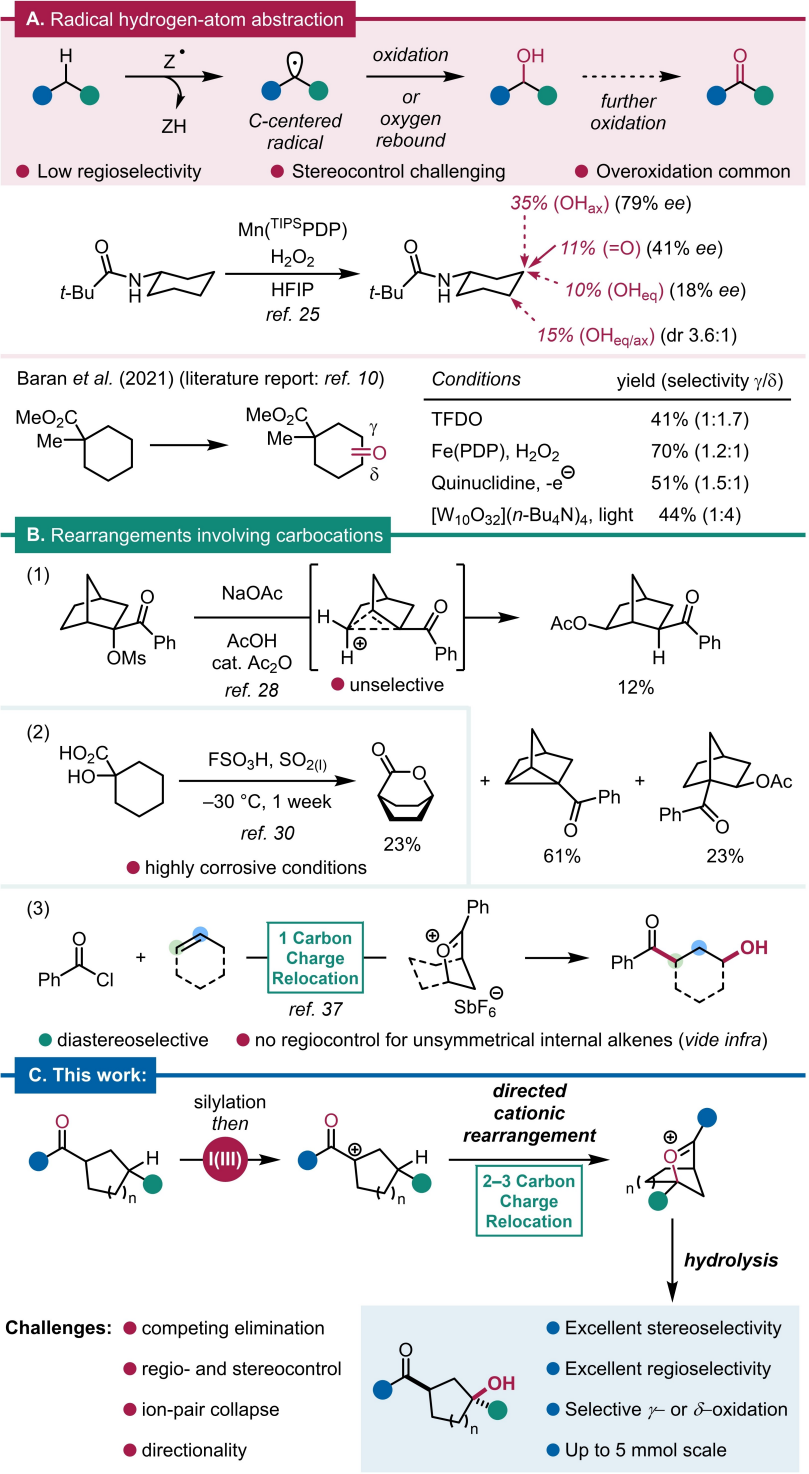
Strategies for C−H bond oxygenation. (A) Radical hydrogen‐atom abstraction strategies; (B) Rearrangements involving carbocations; (C) This work: Iodine(III)‐mediated, directed diastereoselective oxidation. PDP=2‐[[2‐[1‐(pyridin‐2‐ylmethyl)pyrrolidin‐2‐yl]pyrrolidin‐1‐yl]methyl]pyridine, TIPS=triisopropylsilyl, HFIP=hexafluoroisopropanol.

Indeed, while various systems have been reported for C−H oxygenation, most prominently methyl(trifluoromethyl)dioxirane (TFDO),[^12^, ^13^] metal‐oxo complexes (cf. White‐Chen oxidation),[^4^, ^14^, ^15^, ^16^, ^17^, ^18^, ^19^, ^20^, ^21^] heteroatom‐mediated electrochemical oxidation,[^10^, ^22^] and decatungstate photocatalysis,^[23]^ established methods fail to provide good stereocontrol on simple alicyclic substrates.

Similarly, while a certain degree of (even absolute) stereocontrol is possible with chiral manganese complexes, such protocols also illustrate the challenges of metal‐catalyzed single‐electron oxidation of C−H bonds to control regio‐ and diastereoselectivity, as well as the oxidation state of the product (Scheme 1A).[^24^, ^25^, ^26^]

We were intrigued by the apparent dearth of C−H oxidation methods invoking discrete carbocationic intermediates. The oxidation of C−H bonds has only been reported to be possible using rearrangement reactions of carbocations in isolated cases (Scheme 1B),[^27^, ^28^, ^29^, ^30^] and, whereas superelectrophilic C−H functionalization reactions have gained significant interest, they rely on highly corrosive and challenging conditions.[^31^, ^32^, ^33^, ^34^]

The limited occurrence of carbocationic intermediates in selective C−H oxidation methods is likely a result of the intrisic high reactivity of these species. Indeed, carbocations are, in most cases, short‐lived intermediates prone to undergo E1 elimination or intermolecular ion‐pair collapse (as they are typically accompanied by a non‐innocent counteranion). Furthermore, the *sp* ^*2*^‐hybridization of carbocations renders them challenging reaction intermediates for the control of relative (or absolute) stereochemical configuration.[^35^, ^36^] A method for regio‐ and stereoselective oxidation of unreactive C−H bonds in unbiased substrates, with product control, thus remains in high demand.

We recently reported a study on oxocarbenium ion formation through the addition of an acylium ion to an alkene, and the ability of carbocations to be guided by weakly coordinating functional groups in an approach we termed charge relocation (Scheme 1B, eq. 3).^[37]^ Notably, whereas this method allows exquisite diastereocontrol, acylium ion addition only proceeds regioselectively when terminal or symmetrical alkenes are employed. Hoping to address this problem and uncover more details about the mechanism of charge relocation, we were inspired to generate analogous carbocationic intermediates by other means.

Thus, building on our previous results, we have developed a new conceptual framework in which a carbonyl group assumes the dual role of (a) *generating* a destabilized, positively charged center and (b) *directing* the fate of that charge (Scheme 1C). By rendering the process of carbocation formation intramolecular, and by invoking a longer‐range charge relocation—by virtue of ionization α to the ketone, charge relocation occurs along 2–3 carbons (compared to the previous report of 1 carbon relocation^[37]^)—we aimed to address specific limitations of our previous work, while also enabling a more detailed analysis of the underlying mechanisms.

We herein describe our efforts toward the implementation of such a concept, resulting in a method for the diastereo‐ and regioselective oxygenation of remote C−H bonds with several intriguing mechanistic features, including a chirality relay (Scheme 1C). It was pointed out by a reviewer, that this process can be viewed as a “remote Rubottom oxidation”.

As shown in Scheme 1C, we designed the initial cation‐forming event to occur through iodine(III)‐mediated *Umpolung*, which is well known to lead to the (formal) generation of α‐keto carbocations.[^38^, ^39^, ^40^]

Following the concept of charge relocation,^[37]^ the resulting charge, destabilized by the proximity of the electron‐depleting carbonyl, might evolve through sequential hydride migration along the carbon skeleton, culminating in capture by the carbonyl oxygen as the ultimate driving force.[^27^, ^41^, ^42^]

To prevent the possibility of competing elimination or nucleophilic capture through ion‐pair collapse, we postulated the requirement for an entirely base‐ and nucleophile‐free reaction medium—challenging criteria for traditional iodine(III) chemistry.[^43^, ^44^] Indeed, all attempts to express the aforementioned reactivity using standard iodine(III) reagents such as bis(acetoxy)iodobenzene (PIDA), bis(trifluoroacetoxy)iodobenzene (PIFA) or iodosobenzene ([PhIO]_n_) led to the formation of side‐products resulting from elimination, desilylation and unselective oxidation (Scheme 2A).

**Scheme 2:**
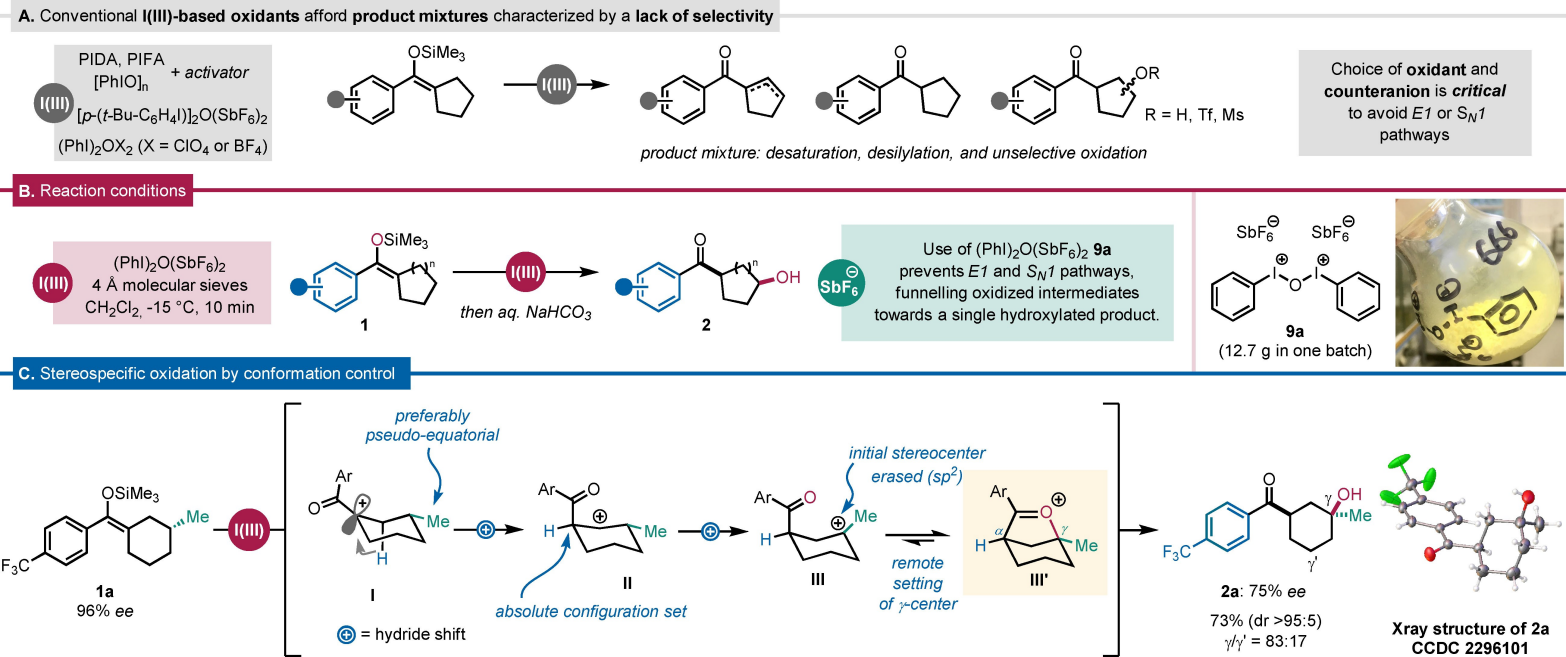
(A) The choice of oxidant and counteranion is crucial for the suppression of side reactions; (B) Reaction set‐up: silyl enol ether (1.5 equiv.), iodine(III) reagent (**9 a**, 1.0 equiv.), CH_2_Cl_2_, −15 °C, 10 min; yield determined based on iodine(III) reagent. (C) Stereospecific oxidation by conformation control; PIDA=(diacetoxyiodo)benzenephenyl, PIFA=(bis(trifluoroacetoxy)iodo)benzene, dr=diastereomeric ratio; *ee*=enantiomeric excess.

Following extensive optimization, we were able to identify a family of dicationic μ‐oxo‐bis[(aryl)iodine] reagents, (ArI)_2_OX_2_ (X=BF_4_^−^, ClO_4_^−^, SbF_6_^−^), which satisfied the above requirements.[^45^, ^46^] In particular, (PhI)_2_O(SbF_6_)_2_ (**9a**) was found suitable to generate a carbocation carrying a non‐basic and non‐nucleophilic hexafluoroantimonate counteranion. Notably, synthesis of this reagent was achieved on a scale of up to 12 g and, during the course of our work, we found that it can be stored indefinitely at −20 °C (>6 months, see Supporting Information) (Scheme 2B).

We began our investigations into the aptitude of our approach, both in terms of reaction design and choice of reagent, by probing the formation of keto alcohol **2 a**, which we hoped to also provide insight into potential stereospecificity of the reaction (Scheme 2C). The question of stereospecificity was particularly intriguing, as carbocationic intermediates are well‐known vehicles for loss of stereochemical information. To our delight, treatment of enantioenriched silyl enol ether **1 a** with **9 a** led to the formation of **2 a** with complete diastereoselectivity (dr >95 : 5) and, more astonishingly—given the transient erasure of the stereogenic center—, largely retained absolute stereochemical information (96 % *ee*→75 % *ee*).

This result suggests that, in accordance with stereoelectronic considerations, the initially formed α‐carbonyl carbocation (intermediate **I**) triggers a first hydride shift which is favored for the pseudo‐axial hydrogen, affording intermediate **II**. As a result, an unusual “long‐range chirality transfer” takes place, as the γ‐methyl group relays information by favoring one conformation over the other. Through a further hydride shift, affording tertiary carbocation **III**, the initial stereocenter is erased, after which internal capture of the cation by the carbonyl provides oxocarbenium ion **III’**, setting the configuration on the γ‐center, and yielding, after aqueous work‐up, the enantioenriched *syn‐*cyclohexanol **2 a** (CCDC 2296101).^[47]^

In order to test the scope of our reaction design, we directed our efforts toward a more complex substrate. The steroid‐derived silyl enol ether **1 b** underwent late‐stage oxidation accompanied by skeletal reorganization, yielding tertiary alcohol **2 b**, (57 % yield, single stereoisomer) (Scheme 3A).^[48]^

**Scheme 3:**
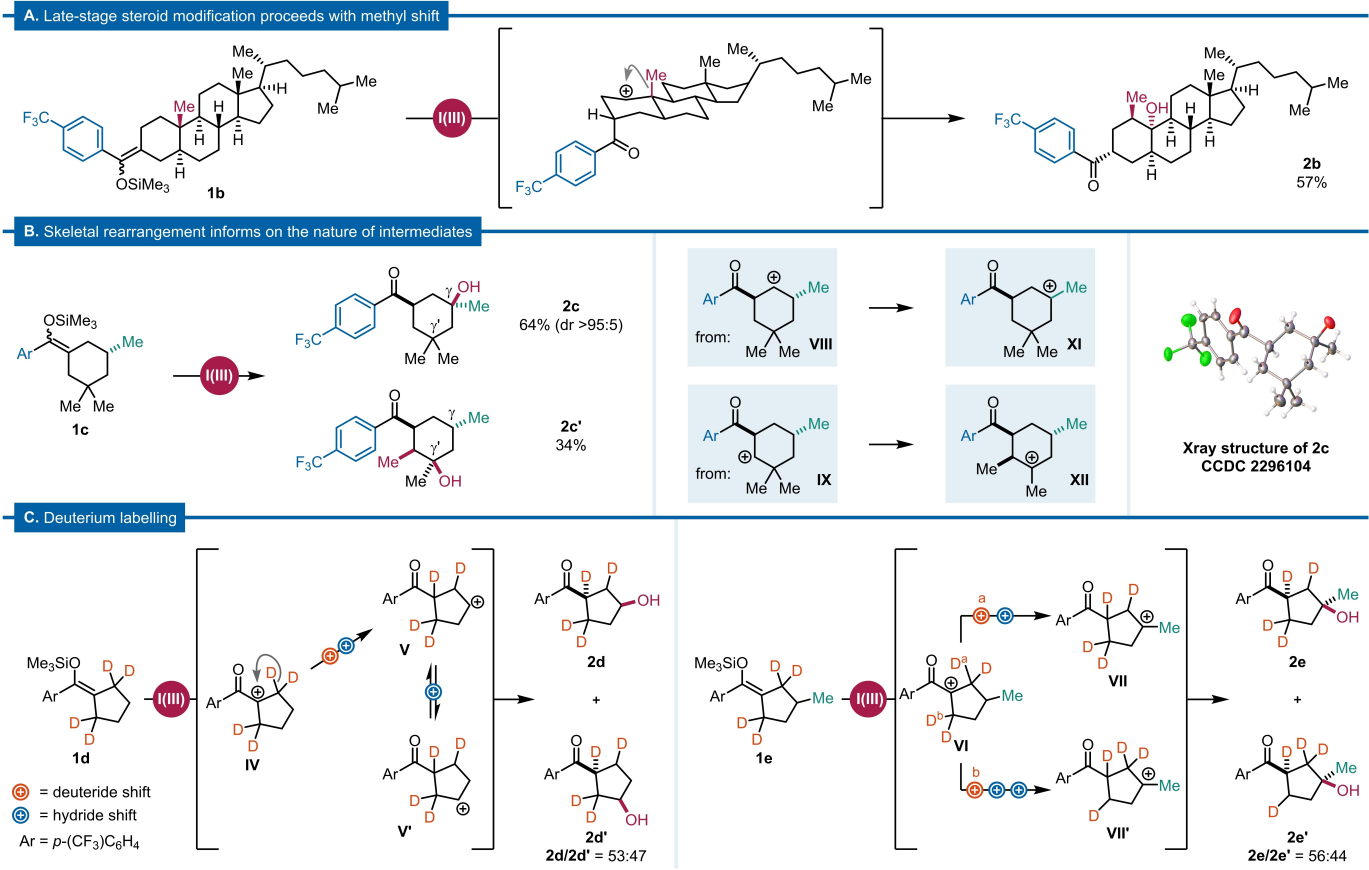
Mechanistic study. (A) Remote oxidation on a steroid silyl enol ether; (B) Skeletal rearrangement of a *gem*‐dimethyl‐containing substrate; (C) Deuterium labelling study.

Product **2 b** constitutes a rare example of highly selective alkyl‐group migration in the context of charge relocation. While the underlying Wagner–Meerwein rearrangement is a textbook process, its implementation within the framework of remote and regio‐ as well as stereoselective C−H oxidation is noteworthy.

Further substrates proved helpful to delineate a more refined mechanistic picture (Scheme 3B). Silyl enol ether **1 c**, bearing a *gem*‐dimethyl motif, yielded a mixture of tertiary alcohol **2 c** (64 %, CCDC 2296104) and a second product, **2 c’** (34 %), which we believe arises from the shift of a methyl substituent from the *gem*‐dimethyl pair, strengthening our previous results from the late‐stage oxidation of a steroid derivative.

The formation of regioisomers, biased towards γ (over γ’) by formation of a tertiary carbocation, opened the question of the directionality of this transformation. Indeed, once formed, two identical routes of rearrangement are possible, resulting in a symmetry‐breaking scenario. To obtain further insight, deuterium‐labelled silyl enol ethers **1 d** and **1 e** were subjected to remote oxidation). **1 d** led to the formation of two isotopomers, **2 d** and **2 d’**, in an almost statistical ratio (53 : 47) (Scheme 3C, left). The symmetry inherent to cyclopentane likely facilitates the establishment of an equilibrium between the two γ‐ and γ’‐carbocations **V** and **V**’ by sequential and reversible hydride shifts, forming two virtually indiscriminate cyclic oxocarbenium ions and, thus, products **2 d** and **2 d’**. Equally as interesting is the case of methyl‐substituted silyl enol ether **1 e**. Here, two isotopomers, **2 e** and **2 e’** (56 : 44) were obtained (Scheme 3C, right). This result again suggests that unidirectionality is not a pre‐requisite of directed cationic rearrangement, as the initial direction of migration is evenly distributed, although the positive charge is eventually funneled exclusively toward the tertiary carbocation.

Taken together, the results obtained using deuterium‐labelled substrates suggest that cationic migration entails a sequence of two *de facto* irreversible hydride shifts—as evidenced by the lack of deuterium incorporation on the γ‐carbon—, followed by an equilibrium involving the reciprocal exchange of relatively stable carbocations between the γ‐ and γ’‐positions through further hydride shifts.

These data are consistent with a mechanistic picture (see Supporting Information for a full mechanistic proposal and a rationale for the observed regioselectivity) in which rapidly interconverting intermediates converge to a terminal species more closely resembling an oxocarbenium ion. In fact, NMR experiments enabled direct observation of such a species as the resting state of the reaction (see Supporting Information for further details).

To probe the applicability of our transformation within a synthetic context, a range of silyl enol ethers was subjected to the reaction conditions. When treated with (PhI)_2_O(SbF_6_)_2_ (**9 a**), cyclopentyl silyl enol ether **1 f** afforded C−H oxidation product **2 f** in 74 % yield (Scheme 4) and with outstanding *syn*‐selectivity (dr >95 : 5). This transformation was scalable up to 5 mmol with neither noticeable erosion of yield (69 %) nor diastereoselectivity. Generally, electron‐withdrawing groups and halides were found to be well tolerated in different positions of the aromatic substituent (**2 g**–**w**), while electron‐donating groups were found to provide inferior results. Overall, substrates bearing (Lewis) basic functionality were found to perform poorly, giving low yields and leading to significant side‐product formation, including elimination and hydrolysis of the starting material. Particularly, trifluoromethyl‐substituted arenes were found to give the highest yields, an outcome which we ascribe to the carbonyl's diminished basicity, preventing the occurrence of undesired elimination. While it should be noted that product **2 h**, bearing *o*‐CF_3_ substitution on the arene, was not obtained diastereomerically pure (due to minor epimerization at the—particularly acidic— carbon α to the ketone ), other substrates, bearing *m*‐CF_3_, nitrile, ester or halide functionality were amenable to this C−H oxidation reaction with excellent stereocontrol (dr >95 : 5), affording the corresponding alcohols (**2 g**, **2 i**, **2 j**, **2 k** and **2 l**) in yields ranging from 41–61 %. Unsubstituted aromatic groups such as phenyl and naphthyl were also well tolerated and gave the desired products in yields of 57 % and 52 %, respectively, and with full regio‐ and stereocontrol (**2 m** and **2 n**). Notably, unsubstituted cyclohexanes were selectively oxidized in the γ‐position, and formation of the δ‐alcohol was not observed (**2 o** and **2 p**).

**Scheme 4:**
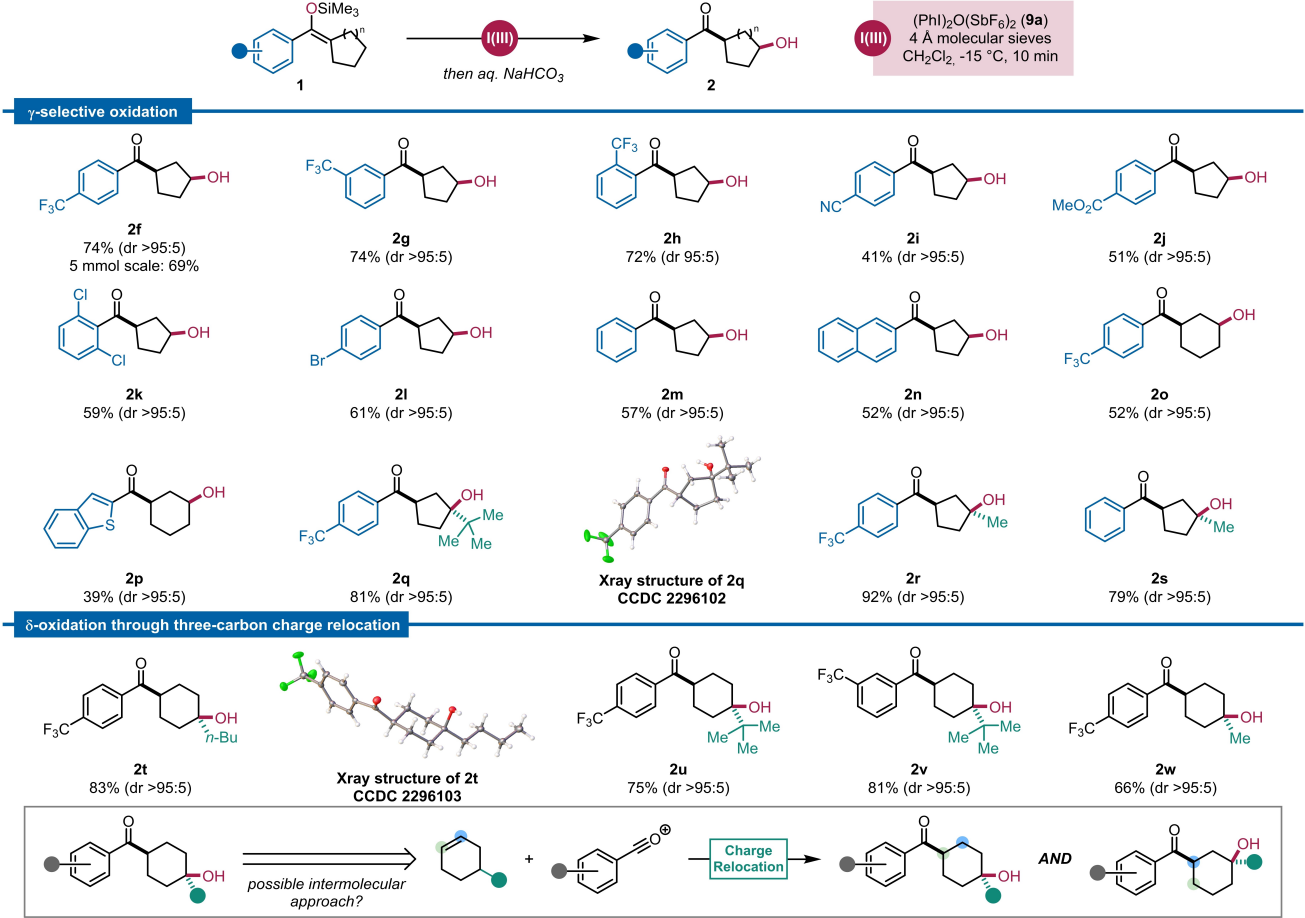
Scope of the reaction under optimized conditions: **1** (1.5 equiv.), **9 a** (1 equiv.), 4 Å molecular sieves, CH_2_Cl_2_, −15 °C, 10 min, *then* NaHCO_3_ (sat. aq.), see the Supporting Information for further experimental details.

We were further pleased to observe that alkyl‐substituted cyclopentyl rings allowed the selective formation of the corresponding tertiary alcohols in excellent yields, maintaining complete diastereoselectivity (**2 q**, **2 r** and **2 s**). Even a sterically hindered neopentylic alcohol (**2 q**) was formed in high yield (81 %) using this protocol; X‐ray analysis of this product unambiguously substantiates the stereochemical outcome of these transformations (**2 q**, CCDC 2296102).

Distinctly, δ‐substituted cyclohexanes afforded the corresponding tertiary δ‐alcohols **2 t**–**w** with full regio‐ and diastereoselectivity, again suggesting that carbocationic stabilization plays a crucial role in this process. Once more, the *syn* stereochemical relationship was confirmed by X‐ray analysis (**2 t**, CCDC 2296103). Importantly, as shown in Scheme 4 (bottom inset), owing to the non‐symmetrical nature of a putative alkene substrate, this range of 1,4‐disubstituted cyclohexanols is fundamentally inaccessible using our previously reported intermolecular hydroxyacylation protocol, as this would inevitably provide a regioisomeric mixture of products.^[37]^ It is also worthy of note that the products **2 t**–**w** represent examples of charge relocation over three bonds, involving three distinct hydride shifts—a step further in the development of selective C−H bond functionalization through charge relocation.

In summary, we have developed a new strategy for the oxygenation of inert C−H bonds by leveraging carbocation chemistry. The intramolecular design of this reaction allowed the circumvention of regioselectivity limitations observed in our previous work, notably allowing the use of unsymmetrically substituted cyclic substrates, late‐stage functionalization of a steroid and an enantiospecific transformation. It also allowed the study of the fundamental question of directionality in carbocation rearrangements, and deuterium labelling experiments, as well as the observation of methyl shifts, provided a clearer picture of the reaction mechanism.

## Supporting Information

The authors have cited additional references within the Supporting Information.[^49^, ^50^, ^51^, ^52^, ^53^, ^54^, ^55^, ^56^, ^57^, ^58^, ^59^, ^60^, ^61^, ^62^, ^63^, ^64^, ^65^, ^66^, ^67^, ^68^, ^69^, ^70^, ^71^, ^72^, ^73^, ^74^, ^75^, ^76^, ^77^, ^78^, ^79^, ^80^, ^81^]

## Conflict of Interests

The authors declare no conflict of interest.

## Supporting information

As a service to our authors and readers, this journal provides supporting information supplied by the authors. Such materials are peer reviewed and may be re‐organized for online delivery, but are not copy‐edited or typeset. Technical support issues arising from supporting information (other than missing files) should be addressed to the authors.

Supporting Information

## Data Availability

The data that support the findings of this study are available in the supplementary material of this article.
