# Supplementary material for: Cationic, Iodine(III)‐Mediated and Directed Diastereoselective Oxidation of Inert C−H Bonds in Cyclic Hydrocarbons
Source: Angew Chem Int Ed Engl. 2025 Mar 6;64(13):e202421872. doi: 10.1002/anie.202421872 (PMC11933533; doi:10.1002/anie.202421872)
Supplement: Supplementary file 1 — Supporting Information [file ANIE-64-e202421872-s001.pdf]

Angewandte Chemie
Eine Zeitschrift der Gesellschaft Deutscher Chemiker

Supporting Information

Cationic, Iodine(III)-Mediated and Directed Diastereoselective Oxidation of Inert C–H Bonds in Cyclic Hydrocarbons

*N. G.-Simonian, B. R. Brutiu, D. Kaiser, N. Maulide**

Cationic, Iodine(III)-Mediated, and Directed Diastereoselective Oxidation of Inert C–H Bonds in Cyclic Hydrocarbons

Nicolas G.-Simonian,[a],† Bogdan R. Brutiu,[a],† Daniel Kaiser[a] and Nuno Maulide[a],*

[a]Institute of Organic Chemistry, University of Vienna; Währinger Straße 38, 1090 Vienna, Austria.

†Equal contribution

*Corresponding author; nuno.maulide@univie.ac.at

Table of Contents

1 General information 3
2 Optimization of reaction conditions 4
3 Synthesis of starting materials 6
3.1 Grignard Reagents 11
3.2 Weinreb amides 6a-6d 12
3.3 *N*-Tosylhydrazones 7a-7h 16
3.4 Ketones 8a-8c, 8f-8r and 8t-8w 24
3.5 Preparation of 8s 44
3.6 Silyl enol ethers 1a-1c and 1f-1w 48
4 Synthesis of the iodonium reagents 9a-9d 69
5 Oxidation products 2a-2c’ and 2f-2w 75
6 Comparison with White-Chen oxidation 102
7 Mechanistic studies 109
7.1 Overview hydride shifts depending on ring size and substituent position 109
7.2 NMR studies 112
7.3 Deuterium-labelling studies 116
7.4 Limitations 131
8 X-ray data 132
9 NMR Spectra 144
10 References (Manuscript 1-48, Supporting Information 49-81) 337

1 General information

General procedures. All reactions were performed in round-bottom flasks or vials fitted with rubber septa and with magnetic stirring, unless otherwise stated. Reaction vessels were flushed with argon prior to use, unless otherwise stated. Liquids and solutions were transferred *via* syringe. All reactions were performed using anhydrous solvents obtained from Acros Organics, TCI or Sigma-Aldrich. Reaction progress was monitored by thin layer chromatography (TLC) performed on aluminum plates coated with silica gel F_{254} with 0.2 mm thickness. Chromatograms were visualized by fluorescence quenching with UV light at 254 nm or by staining using potassium permanganate, followed by heating. Flash column chromatography was performed using silica gel 60 (230-400 mesh, Merck and co.), or pre-packed columns and reagent grade solvents.

Materials. All commercial reagents and solvents were used without further purification.

Instrumentation. All ^{1}H NMR, ^{13}C DEPTQ-135 NMR, ^{13}C CPD NMR and ^{19}F NMR spectra were recorded using a Bruker AV-400, AV-500, AV-600 or AV-700 spectrometer at 300 K. Chemical shifts (δ) were given in parts per million (ppm), referenced to the solvent peak of $CDCl_3$, defined at δ = 7.26 ppm (^{1}H NMR) and δ = 77.16 ppm (^{13}C NMR). Spectra of iodonium reagents were recorded in $CDCl_3$/DMSO-d_6 (8:2 mixture), in which case the chemical shifts were referenced to the residual peak of DMSO-d_6, defined at δ = 2.50 ppm (^{1}H NMR) and δ = 39.52 ppm (^{13}C NMR). Coupling constants (J) are reported in Hertz (Hz). ^{1}H NMR splitting patterns are designated as singlet (s), doublet (d), triplet (t), quartet (q), quintet (quint.) or a combination thereof, as they appeared in the spectrum. If the appearance of a signal differs from the expected splitting pattern, the observed pattern is designated as apparent (app). Splitting patterns that could not be interpreted or easily visualized are designated as multiplet (m) or broad (br). Infrared (IR) spectra were obtained using Perkin-Elmer Spectrum 100 FT-IR spectrometer. Wavenumbers (ν_{max}) are reported in cm^{-1}. Mass spectra were obtained using a Bruker maXis UHR-TOF spectrometer (70 eV), using electrospray ionization (ESI) or atmospheric-pressure chemical ionization (APCI) or an Agilent 7200B GC/Q-TOF spectrometer (70 eV), using electron ionization (EI). Optical rotations were measured on a Perkin Elmer 341 polarimeter using a 100 mm path-length cell at 589 nm (c given in g / (100 mL)). Chiral HPLC was performed using an AGILENT Infinity 1260 with Chiralpak IA-3 or Lux-3 Cellulose-3 columns. Details of chromatographic conditions are indicated under each compound.

2 Optimization of reaction conditions

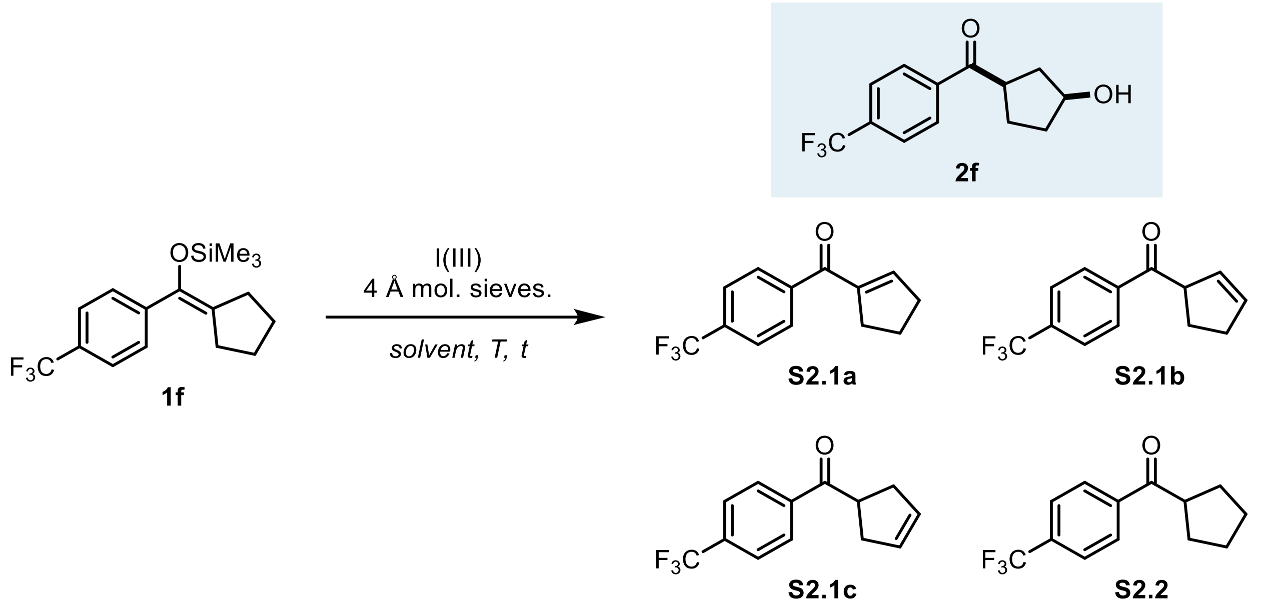

entry	I(III)	Solvent	*T* (°C)	*t* (min)	**S2.1a** (%)	**S2.1b** (%)	**S2.1c** (%)	**S2.2** (%)	**2f**(%)	d.r.
1	$(PhI)_2O(SbF_6)_2$ (1 equiv.)	PhMe	-15	60	<5	<5	<1	63	**7**	-
2	$(PhI)_2O(SbF_6)_2$ (1 equiv.)	$CHCl_3$	-15	60	<5	-	-	19	**40**	>95:5
3	$(PhI)_2O(SbF_6)_2$ (1 equiv.)	EtOAc	-15	60	26	<5	<1	<5	**<5**	-
4	$(PhI)_2O(SbF_6)_2$ (1 equiv.)	$PhCF_3$	-15	60	7	-	-	27	**27**	-
5	$(PhI)_2O(SbF_6)_2$ (1 equiv.)	*o*-DFB	-15	60	25	-	-	13	**20**	
6	$(PhI)_2O(SbF_6)_2$ (1 equiv.)	$MeNO_2$	-15	60	2	-	-	31	**25**	
7	$(PhI)_2O(SbF_6)_2$ (1 equiv.)	PhH	-15	60	2	-	-	15	**36**	
8	$(PhI)_2O(SbF_6)_2$ (1 equiv.)	1,2-DCE	-15	60	2	-	-	35	**15**	
9	$(PhI)_2O(SbF_6)_2$ (1 equiv.)	CH_2Cl_2	-15	60	<5	-	-	19	**45**	>95:5
10[a,b]	PIDA/Me_3SiOTf (1.2/1.4 equiv.)	CH_2Cl_2	-78	60	48	-	-	-	**-**	-
11[a,b]	PIFA/Me_3SiOTf (1.2/1.4 equiv.)	CH_2Cl_2	-78	60	35	-	-	10	**-**	-
12 [a,b]	PhI(*t*-BuCOO)$_2$/Me_3SiOTf (1.2/1.4 equiv.)	CH_2Cl_2	-78	60	75	-	-	-	**-**	-
13[a]	HBX/Me_3SiOTf (1.2/1.2 equiv.)	CH_2Cl_2	-15	60	16	-	-	75	**-**	-

14[a,b]	AcBX / Me_3SiOTf (1.2/1.4 equiv.)	CH_2Cl_2	-78	60	10	-	-	10	**35**	
15[a,b]	PivBX / Me_3SiOTf (1.2/1.4 equiv.)	CH_2Cl_2	-78	60	3	-	-	87	**-**	-
16[a,b]	$[PhIO]_n/Tf_2O$ (2.4/2.2 equiv.)	CH_2Cl_2	-15	60	40	-	-	25	**-**	-
17	$(PhI)_2O(BF_4)_2$ (1 equiv.)	CH_2Cl_2	-15	60	*Complex alkenes mixture*		11		**5**	
18	$(PhI)_2O(ClO_4)_2$ (1 equiv.)	CH_2Cl_2	-15	60	15	-	-	38	**-**	-
19	$(t\text{-}BuPhI)_2O(SbF_6)_2$ (1 equiv.)	CH_2Cl_2	-15	60	26	2	-	56	**-**	-
20[b]	$(PhI)_2O(SbF_6)_2$ (1 equiv.)	CH_2Cl_2	-15	60	4	-	-	23	**43**	
21[c]	$(PhI)_2O(SbF_6)_2$ (1 equiv.)	CH_2Cl_2	-15	60	6	-	-	23	**53**	>95:5
22[c]	$(PhI)_2O(SbF_6)_2$ (1 equiv.)	CH_2Cl_2	-15	10	8	-	-	21	**62**	>95:5
23[d]	$(PhI)_2O(SbF_6)_2$ (1 equiv.)	CH_2Cl_2	-15	10	9	-	-	29	**79 (74%)[e]**	>95:5

Table S1. Optimization of reaction conditions. All reactions were carried out on a 0.1 mmol scale. All indicated yields were calculated by analysis of the ^1H NMR spectrum of the crude material, using mesitylene as an internal standard. [a] Activation of the iodine species was performed analogously to procedure already reported.[49-50] [b] Reaction performed without 4 Å molecular sieves. [c] Reaction performed with 1.25 equiv. of **1f**. [d] Reaction performed with 1.5 equiv. of **1f**. [e] In parenthesis, isolated yield. *o*-DFB = *o*-difluorobenzene, 1,2-DCE = 1,2-dichloroethane, HBX = 1-hydroxy-1,2-benziodoxol-3(1*H*)-one, AcBX = 1-(acetyloxy)-1,2-benziodoxol-3(1*H*)-one, PivBX = 1-(pivaloyloxy)-1,2-benziodoxol-3(1*H*)-one.

3 Synthesis of starting materials

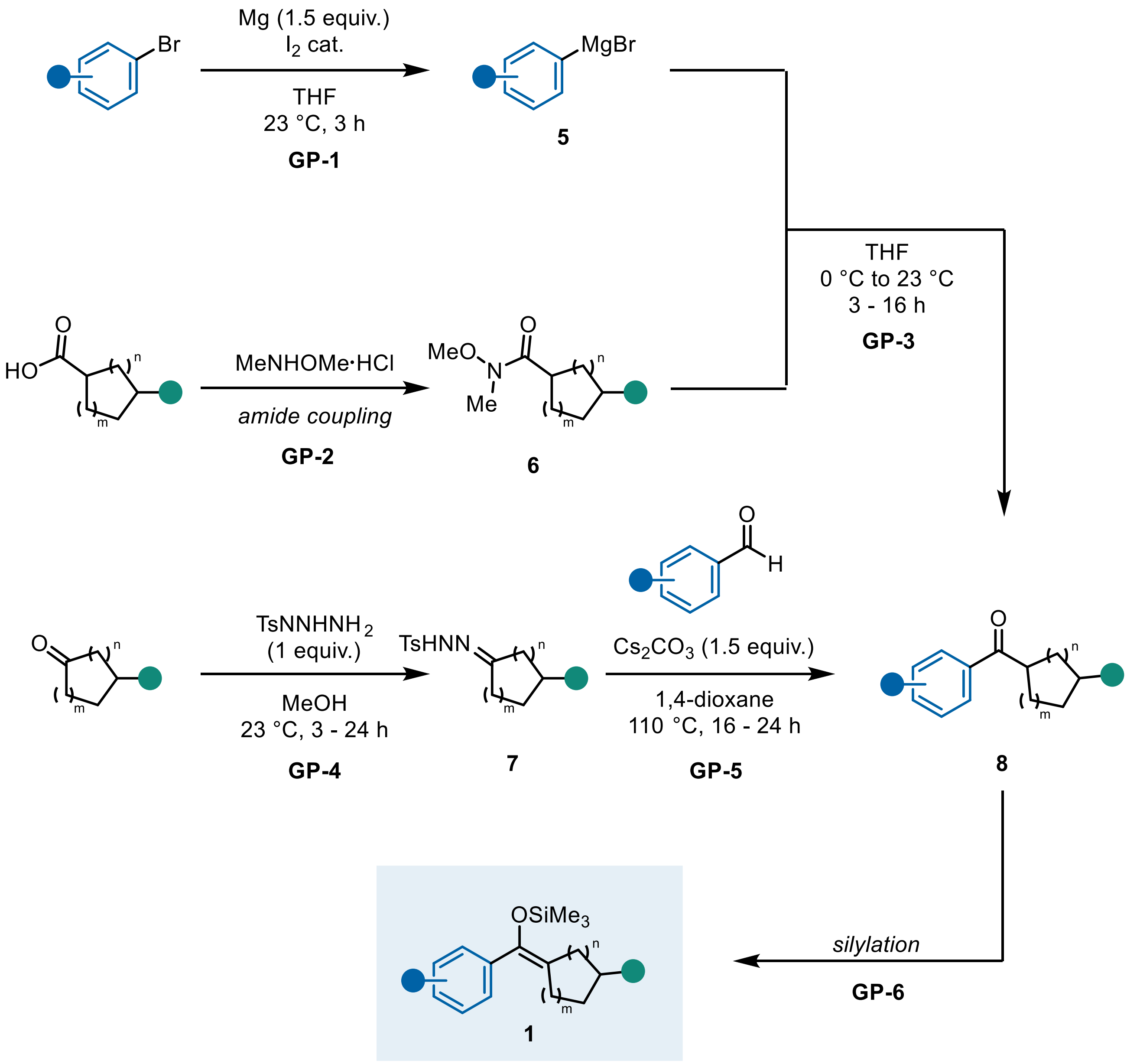

Figure S1. General strategies for the synthesis of starting materials.

General procedure GP-1

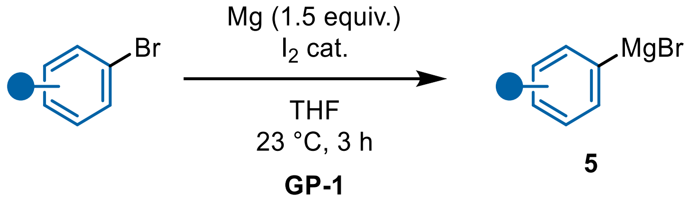

Grignard reagents were synthesized according to a standard procedure:

Magnesium turnings (1.50 equiv.) were added to a flame-dried flask under argon and covered with dry, unstabilized THF (*ca* 2.00 mL). A crystal of iodine was added and the mixture was stirred until a homogenous coloration was obtained. Without stirring, a few drops of the pure aryl bromide were added (*ca* 5 drops). Upon discoloration, indicating initiation of the reaction, the rest of the bromide (1.00 equiv. in total) was solubilized in THF (0.75 M in total according to bromide) and the resulting solution was added dropwise (over *ca* 20 min). The resulting mixture was left stirring for 3 h and the resulting solution of Grignard reagent was titrated according to the procedure described by B. E. Love.[51]

General procedure GP-2

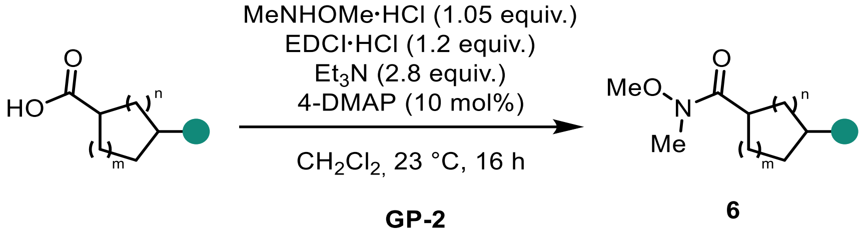

To a solution of the carboxylic acid (1.00 equiv.) in CH_2Cl_2 (0.2 M), *N,O*-dimethylhydroxylamine hydrochloride (1.05 equiv.), EDCI•HCl (1.20 equiv.), 4-DMAP (10 mol%) and Et_3N (2.80 equiv.) were successively added and the resulting mixture was left stirring at 23 °C for 16 h. An aq. solution of HCl (1 M) was subsequently added and the phases were separated. The organic phase was washed with aq. HCl (1 M), sat. aq. $NaHCO_3$ twice and brine. The resulting solution was dried over $MgSO_4$, filtered and the solvent was evaporated under reduced pressure to yield the corresponding Weinreb amide, which was used in the next step without further purification.

General procedure GP-3

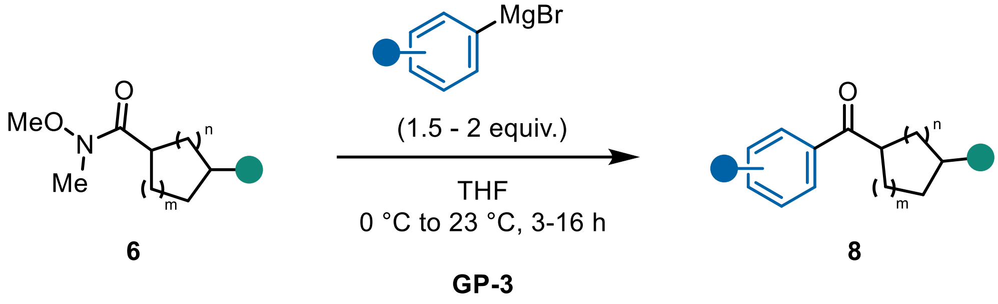

To a solution of the Weinreb amide (1.00 equiv.) in THF (0.25 M) was added a THF solution of Grignard reagent (1.50 – 2.00 equiv.) at 0 °C. The resulting solution was warmed to 23 °C and left stirring until TLC indicated full conversion of the Weinreb amide (3 – 16 h). Upon completion, a sat. aq. NH_4Cl solution was added, the phases were separated and the aqueous phase was extracted with Et_2O (3 x). The combined organic phases were washed with brine, dried over $MgSO_4$, filtered and concentrated *in vacuo*. The residue was purified by flash column chromatography on silica gel (typical eluent: heptanes/EtOAc = 100:0 to 90:10) to afford the pure ketone.

General procedure GP-4

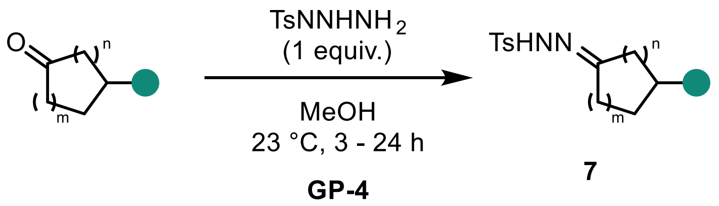

Hydrazones were prepared according to a procedure previously described in the literature.[52]

To a solution of the ketone (1.00 equiv.) in MeOH (0.5 M) was added 4- methylbenzenesulfonylhydrazide (1.00 equiv.) and the mixture was left stirring at 23 °C for 3 – 16 h. Then, the solvent was evaporated under reduced pressure to afford the hydrazone which was used in the next step without further purification.

General procedure GP-5

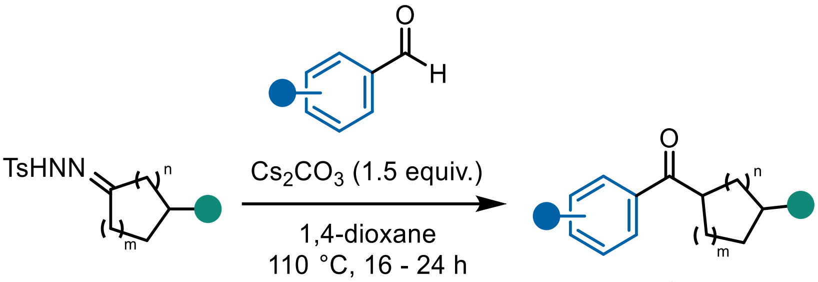

7 **GP-5** **8**

Ketones were prepared according to a procedure previously described in the literature.[52]

To a mixture of hydrazone (1.00 equiv.) and Cs_2CO_3 (1.50 equiv.) in 1,4-dioxane (0.1 M) was added the aromatic aldehyde (1.00 – 1.20 equiv.) and the mixture was refluxed for 16 – 24 h. After cooling, sat. aq. NH_4Cl was added and the phases were separated and the aqueous phase was extracted with CH_2Cl_2 (3 x). The combined organic phases were washed with brine, dried over $MgSO_4$, filtered and concentrated under reduced pressure. The residue was purified by flash column chromatography on silica gel (typical eluent: heptanes/EtOAc 100:0 to 90:10) to afford the corresponding ketone, which was used in the next step (silylation) without further purification.

In some cases, formation of a gel, which hindered stirring, was observed upon heating. In this scenario, additional 1,4-dioxane was added. This usually does not affect the efficiency of the reaction.

General procedure GP-6

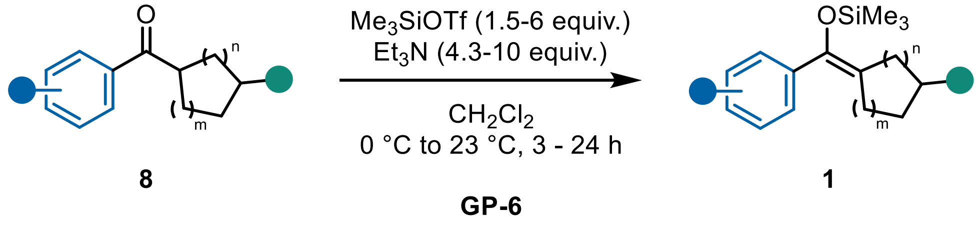

To a solution of the ketone (1.00 equiv.) and Et_3N (4.30 – 10.0 equiv.) in CH_2Cl_2 (0.5 M) at 0 °C Me_3SiOTf (1.50 – 6.00 equiv.) was added dropwise and the mixture was subsequently allowed to slowly warm to 23 °C. Upon completion of the reaction (indicated by TLC), the mixture was cooled to 0 °C and a sat. aq. solution of $NaHCO_3$ was added slowly. The phases were separated and the aqueous phase was extracted with CH_2Cl_2 (3 x). The combined organic phases were dried over $MgSO_4$, filtered and concentrated under reduced pressure. The residue was purified by flash column chromatography on silica gel (pentane) to afford the pure silyl enol ether.

3.1 Grignard Reagents

5a – [4-(Trifluoromethyl)phenyl]magnesium bromide

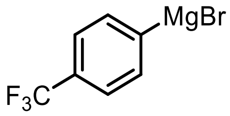

Synthesized following **GP-1**, using 4-bromobenzotrifluoride (6.36 mL, 45.0 mmol, 1.00 equiv.) and magnesium (1.64 g, 67.5 mmol, 1.50 equiv.). The resulting solution of **5a** (70.0 mL, 0.46 M in THF, 32.0 mmol, 72%) was immediately used in the next step.

5b – [3-(Trifluoromethyl)phenyl]magnesium bromide

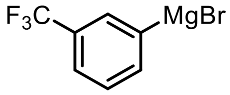

Synthesized following **GP-1**, using 3-bromobenzotrifluoride (1.67 mL, 12.0 mmol, 1.00 equiv.) and magnesium (438 mg, 18.0 mmol, 1.50 equiv.). The resulting solution of **5b** (17.0 mL, 0.49 M in THF, 8.30 mmol, 69%) was immediately used in the next step.

5c – [3,5-bis(trifluoromethyl)phenyl]magnesium bromide

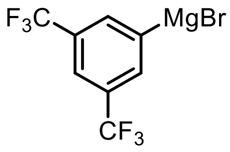

Synthesized following **GP-1**, using 1,3-bis(trifluoromethyl)-5-bromobenzene (1.20 mL, 7.00 mmol, 1.00 equiv.) and magnesium (255 mg, 10.5 mmol, 1.50 equiv.). The resulting solution of **5c** (11.0 mL, 0.60 M in THF, 6.42 mmol, 92%) was immediately used in the next step.

3.2 Weinreb amides 6a-6d

6a – *N*-Methoxy-*N*-methylcyclopentanecarboxamide

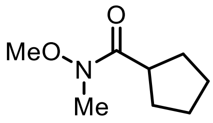

Formula: $C_8H_{15}NO_2$

MW: 157.2 g/mol

Synthesized following **GP-2**, using cyclopentanecarboxylic acid (2.17 mL, 20.0 mmol, 1.00 equiv.), *N,O*-dimethylhydroxylamine hydrochloride (2.05 g, 21.0 mmol, 1.05 equiv.), EDCI•HCl (4.60 g, 24.0 mmol, 1.20 equiv.), 4-DMAP (0.240 g, 2.00 mmol, 10 mol%) and Et_3N (7.80 mL, 56.0 mmol, 2.80 equiv.). The resulting product **6a** (2.67 g, 17.0 mmol, 85%) was used in the next step without further purification.

Spectral data were in accordance with the literature.[53]

^{1}H NMR (400 MHz, $CDCl_3$): δ 3.69 (s, 3H), 3.18 (s, 3H), 3.14 – 3.02 (m, 1H), 1.91 – 1.67 (m, 6H), 1.62 – 1.51 (m, 2H).

6b – *N*-Methoxy-*N*-methylcyclohexanecarboxamide

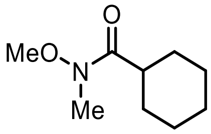

Formula: $C_9H_{17}NO_2$

MW: 171.2 g/mol

Synthesized following **GP-2**, using cyclohexanecarboxylic acid (1.27 mL, 10.0 mmol, 1.00 equiv.), *N,O*-dimethylhydroxylamine hydrochloride (1.02 g, 10.5 mmol, 1.05 equiv.), EDCI•HCl (2.30 g, 12.0 mmol, 1.20 equiv.), 4-DMAP (0.120 g, 1.00 mmol, 10 mol%) and Et_3N (3.90 mL, 28.0 mmol, 2.80 equiv.). The resulting product **6b** (1.40 g, 8.19 mmol, 82%) was used in the next step without further purification.

Spectral data were in accordance with the literature.[54]

^{1}H NMR (400 MHz, $CDCl_3$): δ 3.68 (s, 3H), 3.16 (s, 3H), 2.67 (app br t, *J* = 10.6 Hz, 1H), 1.84 – 1.63 (m, 5H), 1.54 – 1.41 (m, 2H), 1.35 – 1.19 (m, 3H).

6c – 4-(*tert*-Butyl)-*N*-methoxy-*N*-methylcyclohexane-1-carboxamide

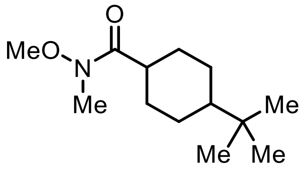

Formula: $C_{13}H_{25}NO_2$

MW: 227.3 g/mol

Synthesized following **GP-2**, using 4-*tert*-butylcyclohexanecarboxylic acid (1.84 g, 10.0 mmol, 1.00 equiv.), *N,O*-dimethylhydroxylamine hydrochloride (1.02 g, 10.5 mmol, 1.05 equiv.), EDCI•HCl (2.30 g, 12.0 mmol, 1.20 equiv.), 4-DMAP (0.120 g, 1.00 mmol, 10 mol%) and Et_3N (3.90 mL, 28.0 mmol, 2.80 equiv.). The resulting product **6c** (2.22 g, 9.78 mmol, 98%, *trans*/*cis* = 6:4) was used in the next step without further purification.

Spectral data were in accordance with the literature.[55]

^{1}H NMR (700 MHz, $CDCl_3$): δ 3.69 (s, 1.8H), 3.67 (s, 1.2H), 3.17 (s, 1.8H), 3.16 (s, 1.2H), 2.92 (br s, 0.4H), 2.69 – 2.54 (m, 0.6H), 2.02 – 1.95 (m, 0.8H), 1.87 – 1.80 (m, 2.4H), 1.59 – 1.38 (m, 3.6H), 1.08 – 0.90 (m, 2.2H), 0.84 (s, 5.4H), 0.84 (s, 3.6H).

6d – *trans*-4-Butyl-*N*-methoxy-*N*-methylcyclohexane-1-carboxamide

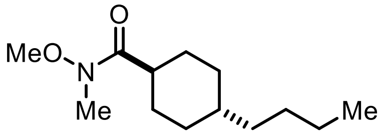

Formula: $C_{13}H_{25}NO_2$

MW: 227.3 g/mol

Synthesized following **GP-2**, using *trans*-4-butylcyclohexanecarboxylic acid (931 mg, 5.00 mmol, 1.00 equiv.), *N,O*-dimethylhydroxylamine hydrochloride (512 mg, 5.25 mmol, 1.05 equiv.), EDCI•HCl (1.15 g, 6.00 mmol, 1.20 equiv.), 4-DMAP (61.0 mg, 0.500 mmol, 10 mol%) and Et_3N (2.00mL, 14.0 mmol, 2.80 equiv.). The resulting product **6d** (1.00 g, 4.41 mmol, 88%) was used in the next step without further purification.

^{1}H NMR (700 MHz, CDCl$_3$): δ 3.67 (s, 3H), 3.16 (s, 3H), 2.61 (br s, 1H), 1.83 – 1.73 (m, 4H), 1.49 (qd, *J* = 12.5, 3.3 Hz, 2H), 1.30 – 1.20 (m, 5H), 1.19 – 1.13 (m, 2H), 0.92 (qd, *J* = 12.0, 3.3 Hz, 2H), 0.88 – 0.84 (m, 3H);

^{13}C NMR (176 MHz, CDCl$_3$): δ 177.8 (br), 61.6, 40.2, 37.2, 37.0, 32.6 (2C), 32.3 (br), 29.2, 29.1 (2C), 23.1, 14.2;

IR (neat) ν_{max}: 2923, 2855, 1661, 1449, 1413, 1384, 1349, 1327, 1175, 1003 cm^{-1};

HRMS (ESI$^+$): exact mass calculated for $[M+H]^+$ ($C_{13}H_{25}NO_2H^+$) requires *m/z* 228.1958, found *m/z* 228.1955.

3.3 *N*-Tosylhydrazones 7a-7h

7a – *N*'-Cyclopentylidene-4-methylbenzenesulfonohydrazide

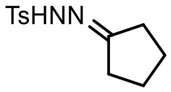

Formula: $C_{12}H_{16}N_2O_2S$

MW: 252.3 g/mol

Synthesized following **GP-4**, using cyclopentanone (0.880 mL, 10.0 mmol, 1.00 equiv.) and 4-methylbenzenesulfonylhydrazide (1.86 g, 10.0 mmol, 1.00 equiv.). The resulting product **7a** (2.17 g, 8.59 mmol, 86%) was immediately used in the next step without further purification.

Spectral data were in accordance with literature.[52]

^{1}H NMR (400 MHz, CDCl$_3$): δ 7.85 (d, *J* = 8.4 Hz, 2H), 7.31 (d, *J* = 8.4 Hz, 2H), 7.26 (br s, 1H, *N–H*), 2.43 (s, 3H), 2.35 (t, *J* = 7.5 Hz, 2H), 2.14 (t, *J* = 7.5 Hz, 2H), 1.79 (quint., *J* = 7.2 Hz, 2H), 1.69 (quint., *J* = 7.2 Hz, 2H).

7b – *N*'-Cyclohexylidene-4-methylbenzenesulfonohydrazide

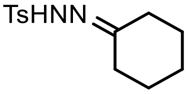

Formula: $C_{13}H_{18}N_2O_2S$

MW: 266.4 g/mol

Synthesized following **GP-4**, using cyclohexanone (2.07 mL, 20.0 mmol, 1.00 equiv.) and 4-methylbenzenesulfonylhydrazide (3.72 g, 20.0 mmol, 1.00 equiv.). The resulting product **7b** (3.61 g, 13.6 mmol, 68%) was immediately used in the next step without further purification.

^{1}H NMR (400 MHz, $CDCl_3$): δ 7.84 (d, *J* = 8.3 Hz, 2H), 7.31 (d, *J* = 8.4 Hz, 2H), 7.15 (br s, 1H, *N–H*), 2.43 (s, 3H), 2.29 – 2.14 (m, 4H), 1.75 – 1.51 (m, 6H);

^{13}C NMR (101 MHz, $CDCl_3$): δ 198.1, 144.1, 135.6, 129.6 (2C), 128.3 (2C), 35.4, 26.9, 26.8, 25.8, 25.5, 21.8;

IR (neat) ν_{max}: 3218, 3067, 2934, 2859, 1640, 1598, 1495, 1448, 1433, 1399, 1327, 1291,1256, 1225, 1185, 1163, 1119, 1093, 1016, 926, 898, 842, 814, 768 cm^{-1};

HRMS (ESI^+): exact mass calculated for $[M+H]^+$ ($C_{13}H_{18}O_2N_2SH^+$) requires *m/z* 267.1162, found *m/z* 267.1170.

7c – 4-Methyl-*N*'-(3-methylcyclopentylidene)benzenesulfonohydrazide

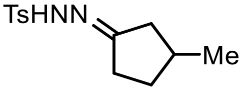

Formula: $C_{13}H_{18}N_2O_2S$

MW: 266.4 g/mol

Synthesized following **GP-4**, using 3-methylcyclopentanone (1.08 mL, 10.0 mmol, 1.00 equiv.) and 4-methylbenzenesulfonylhydrazide (1.86 g, 10.0 mmol, 1.00 equiv.). The resulting product **7c** (2.67 g, 10.0 mmol, quant., *E*/*Z* = 1:1) was immediately used in the next step without further purification.

^{1}H NMR (700 MHz, $CDCl_3$): δ 7.84 (d, *J* = 8.1 Hz, 2H), 7.30 (d, *J* = 8.1 Hz, 2H), 7.24 – 7.05 (m, 1H, *N–H*), 2.53 – 2.24 (m, 2.5H), 2.43 (s, 3H), 2.19 – 2.05 (m, 1H), 2.05 – 1.99 (m, 0.5H), 1.98 – 1.92 (m, 1H), 1.92 – 1.87 (m, 0.5H), 1.65 (ddd, *J* = 17.6, 9.0, 2.1 Hz, 0.5H), 1.35 (dq, *J* = 12.5, 9.0 Hz, 0.5H), 1.26 (tdd, *J* = 12.3, 9.0, 6.4 Hz, 0.5H), 1.03 (d, *J* = 6.6 Hz, 1.5H), 0.99 (d, *J* = 6.6 Hz, 1.5H);

^{13}C NMR (176 MHz, $CDCl_3$): δ 167.9, 144.1, 135.7, 129.7 (2C), [128.16 and 128.14] (2C), [41.6 and 36.2], [33.4 and 33.2], [33.3 and 32.7], [32.9 and 27.9], 21.7, [20.0 and 19.5];

IR (neat) ν_{max}: 3225, 1452, 1167, 1038, 937, 815, 662, 632, 553 cm^{-1};

HRMS (ESI$^+$): exact mass calculated for $[M+Na]^+$ ($C_{13}H_{18}N_2O_2SNa^+$) requires *m*/*z* 289.0981, found *m*/*z* 289.0976.

7d – 4-Methyl-*N*'-(3-methylcyclohexylidene)benzenesulfonohydrazide

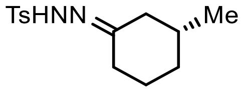

Formula: $C_{14}H_{20}N_2O_2S$

MW: 280.4 g/mol

Synthesized following **GP-4**, using 3-methylcyclohexanone (0.62 mL, 5.00 mmol, 1.00 equiv.) and 4-methylbenzenesulfonylhydrazide (0.930 g, 5.00 mmol, 1.00 equiv.). The resulting product **7d** (1.28 g, 4.67 mmol, 91%, *E*/*Z* = 1:1) was immediately used in the next step without further purification.

(–)-(*R*)-7d was synthesized using the same procedure, staring from (+)-(*R*)-3-methylcyclohexanone [Sigma-Aldrich, 98%, $[\alpha]_D^{20}$ +13.7 (c = 2.0, $CHCl_3$), lit.[56] +14.2 (c = 4.13, $CHCl_3$)].

The enantiomeric ratio for the starting material was calculated to be 98:2 => 96% *ee* for (+)-(*R*)-3-methylcyclohexanone.

Given that hydrazide **7d** is obtained as a mixture of E- and Z-configured isomers, determination of enantiomeric ratios was not readily possible. As neither of the following steps affect the methine stereocenter, and there is no known risk of erosion of *ee*, we can presume all the following products to have an enantiomeric excess of 96%.

Spectral data were in accordance with the literature.[57]

^{1}H NMR (400 MHz, $CDCl_3$): δ 7.86 – 7.81 (m, 2H), 7.32 – 7.28 (m, 2H), 7.27 (br s, 1H, *N–H*), 2.60 – 2.52 (m, 1H), 2.42 (s, 3H) 2.41 – 3.31 (m, 1H), 2.07 – 1.95 (m, 1H), 1.89 – 1.30 (m, 5H), 1.18 – 1.02 (m, 1H), 0.94 (d, *J* = 6.5 Hz, 1.5H), 0.91 (d, *J* = 6.5 Hz, 1.5H);

$[\alpha]_D^{20}$ –13.9 (c = 2.0, $CHCl_3$).

7e – *N*'-[3-(*tert*-Butyl)cyclopentylidene]-4-methylbenzenesulfonohydrazide

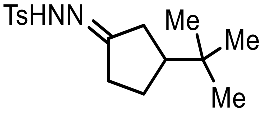

Formula: $C_{16}H_{24}N_2O_2S$

MW: 308.4 g/mol

3-*tert*-Butylcyclopentanone was prepared according to a procedure described in the literature.[58]

7e was synthesized following **GP-4**, using 3-*tert*-butylcyclopentanone (421 mg, 3.00 mmol, 1.00 equiv.) and 4-methylbenzenesulfonylhydrazide (559 mg, 3.00 mmol, 1.00 equiv.). The resulting product **7e** (922 mg, 2.99 mmol, quant.) was immediately used in the next step without further purification. The product was initially obtained as a 1:1 *E*/*Z* mixture that slowly evolved to an 85:15 *E*/*Z* mixture upon storage at 23 °C for 2 months. The *E*/*Z* ratio was determined by ^{13}C NMR.

^{1}H NMR (400 MHz, $CDCl_3$): δ 7.88 – 7.81 (m, 2H), 7.34 – 7.28 (m, 2H), 7.14 (br s, 1H, *N–H*), 2.43 (s, 3H), 2.41 – 2.27 (m, 2H), 2.15 – 1.99 (m, 2H), 1.91 – 1.80 (m, 1H), 1.79 – 1.67 (m, 1H), 1.50 – 1.35 (m, 1H), 0.85 (s, 9H);

^{13}C NMR (101 MHz, $CDCl_3$): δ [167.64 and 167.57], 144.1, 135.7, 129.7 (2C), 128.2 (2C), [50.1 and 49.7], [35.1 and 33.7], [31.7 and 31.6], [29.7 and 28.2], [27.54 and 27.48] (3C), [25.9 and 25.6], 21.8;

IR (neat) ν_{max}: 3226, 2959, 2866, 1401, 1335, 1265, 1166, 1090, 1035, 930, 815, 666 cm^{-1};

HRMS (ESI$^+$): exact mass calculated for $[M+Na]^+$ ($C_{16}H_{24}N_2O_2SNa^+$) requires *m/z* 331.1451, found *m/z* 331.1459.

7f – 4-Methyl-*N*'-(3,3,5-trimethylcyclohexylidene)benzenesulfonohydrazide

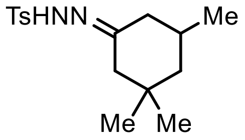

Formula: $C_{16}H_{24}N_2O_2S$

MW: 308.4 g/mol

3,3,5-Trimethylcyclohexan-1-one was prepared according to a procedure described in the literature from commercial isophorone.[59]

7f was synthesized following **GP-4**, using 3,3,5-trimethylcyclohexan-1-one (701 mg, 5.00 mmol, 1.00 equiv.) and 4-methylbenzenesulfonylhydrazide (0.890 g, 4.75 mmol, 0.950 equiv.). The resulting product **7f** (1.30 g, 4.30 mmol, 86%) was triturated with pentane and immediately used in the next step without further purification.

^{1}H NMR (600 MHz, CDCl$_3$): δ 7.81 (m, 2H), 7.29 (t, *J* = 8.0 Hz, 2H), 2.71 – 2.22 (m, 5H), 2.18 – 1.87 (m, 1H), 1.79 – 1.65 (m, 1H), 1.64 – 1.54 (m, 1H), 1.44 (t, *J* = 12.5 Hz, 1H), 1.07 – 1.03 (m, 1H), 1.03– 0.93 (m, 5H), 0.91 (d, *J* = 6.4 Hz, 1H), 0.69 (d, *J* = 8.2 Hz, 3H);

^{13}C NMR (151 MHz, CDCl$_3$): δ 144.1, 135.5, 129.4 (2C), 128.1 (2C), 48.1, [47.82 and 47.76], [43.4 and 39.7], 34.8, [32.1 and 32.0], 30.0, [25.3 and 25.1], [22.52 and 22.27], [21.77 and 21.76]; *some quaternary carbons could not be detected.*

IR (neat) ν_{max}: 2956, 2927, 2870, 1706, 1663, 1598, 1455, 1363, 1337, 1223, 1165, 1122, 1091, 1033, 1008, 915, 813, 730, 680 cm^{-1};

HRMS (ESI$^+$): exact mass calculated for [M+Na]$^+$ ($C_{16}H_{24}N_2O_2SNa^+$) requires *m/z* 331.1451, found *m/z* 331.1449.

7g – 4-Methyl-*N*'-(4-methylcyclohexylidene)benzenesulfonohydrazide

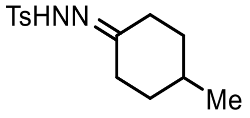

Formula: $C_{14}H_{20}N_2O_2S$

MW: 280.4 g/mol

Synthesized following **GP-4**, using 4-methylcyclohexanone (1.20 mL, 10.0 mmol, 1.00 equiv.) and 4-methylbenzenesulfonylhydrazide (1.86 g, 10.0 mmol, 1.00 equiv.). The resulting product **7g** (2.80 g, 10.0 mmol, quant.) was immediately used in the next step without further purification

Spectral data were in accordance with the literature.[57]

^{1}H NMR (400 MHz, $CDCl_3$): δ 7.83 (d, *J* = 8.3 Hz, 2H), 7.30 (d, *J* = 8.0 Hz, 2H), 2.71 – 2.56 (m, 1H), 2.48 – 2.27 (m, 5H), 2.17 – 2.04 (m, 1H), 1.87 – 1.73 (m, 3H), 1.67 – 1.53 (m, 1H), 1.19 – 0.97 (m, 2H), 0.91 (d, *J* = 6.6 Hz, 3H).

7h – *N*'-((5*S*,8*R*,9*S*,10*S*,13*R*,14*S*,17*R*,*E*)-10,13-Dimethyl-17-((*R*)-6-methylheptan-2-yl)hexa-decahydro-3H-cyclopenta[a]phenanthren-3-ylidene)-4-methylbenzenesulfonohydrazide

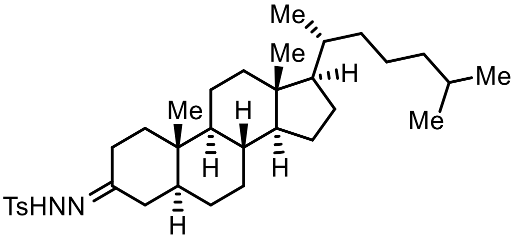

Formula: $C_{34}H_{54}N_2O_2S$

MW: 554.9 g/mol

Synthesized following **GP-4**, using 5α-cholestan-3-one (1.93 g, 5.00 mmol, 1.00 equiv.) and 4-methylbenzenesulfonylhydrazide (931 mg, 5.00 mmol, 1.00 equiv.). The resulting product **7h** (2.54 g, 4.58 mmol, 92%) was immediately used in the next step without further purification.

^{1}H NMR (400 MHz, $CDCl_3$): δ 7.83 (d, *J* = 8.3 Hz, 2H), 7.31 (d, *J* = 8.2 Hz, 2H), 7.03 (br s, 1H), 2.59 – 2.40 (m, 3H), 2.40 – 2.25 (m, 1H), 2.25 – 2.08 (m, 1H), 2.07 – 1.75 (m, 4H), 1.66 (s, 2H), 1.60 – 1.41 (m, 4H), 1.41 – 0.92 (m, 19H), 0.90 – 0.82 (m, 12H), 0.64 (s, 3H);

IR (neat) ν_{max}: 3221, 2949, 2929, 2867, 2846, 2358, 2338, 1740, 1643, 1629, 1597, 1467, 1444, 1428, 1408, 1382, 1345, 1327, 1304, 1268, 1234, 1211, 1188, 1165, 1124, 1093, 1068, 1034, 1020, 1006, 954, 921, 853, 813, 783 cm^{-1};

HRMS (ESI$^+$): exact mass calculated for $[M+H]^+$ ($C_{34}H_{54}N_2O_2SH^+$) requires *m*/*z* 555.3979, found *m*/*z* 555.3975.

3.4 Ketones 8a-8c, 8f-8r and 8t-8w

8a – (3-Methylcyclohexyl)[4-(trifluoromethyl)phenyl]methanone

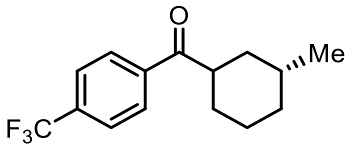

Formula: $C_{15}H_{17}F_3O$

MW: 270.3 g/mol

Synthesized following **GP-5**, using **7d** (0.840 g, 3.00 mmol, 1.00 equiv.), 4-(trifluoromethyl)benzaldehyde (0.430 mL, 3.10 mmol, 1.05 equiv.) and Cs_2CO_3 (1.47 g, 4.50 mmol, 1.50 equiv.). The crude product was purified by flash column chromatography on silica gel (heptanes/EtOAc 100:0 to 95:5) to afford **8a** (0.470 g, 1.70 mmol, 58%, 8:2 d.r.) as a colorless oil.

Following column chromatography, **8a** was obtained contaminated with unreacted aldehyde and other undefined impurities of similar polarity. Despite these contaminants, **8a** was subjected to the subsequent step, silyl enol ether formation. The corresponding silyl enol ether, being less polar, was then easily separable from the aforementioned impurities due to the change in the relative polarities.

(–)-(*R*)-8a was obtained using the same procedure, staring from **(–)-(*R*)-7d**. (See **(–)-(*R*)-7d** for details regarding *ee* calculations)

^{1}H NMR (400 MHz, $CDCl_3$): δ 8.02 (d, *J* = 8.2 Hz, 1.6H), 7.98 (d, *J* = 8.1 Hz, 0.4H), 7.74 – 7.69 (m, 2H), 3.59 – 3.47 (m, 0.2H), 3.35 – 3.19 (m, 0.8H), 1.96 – 1.68 (m, 4H), 1.66 – 1.35 (m, 3H), 1.14 (q, *J* = 12.6 Hz, 0.8H), 0.98 (d, *J* = 6.8 Hz, 0.6H), 0.93 (d, *J* = 6.6 Hz, 2.4H);

^{19}F NMR (376 MHz, $CDCl_3$): δ [-63.07 and -63.09] (3F).

$[\alpha]_D^{20}$ –9.0 (c = 1.0, $CHCl_3$).

8b – ((5*S*,8*R*,9*S*,10*S*,13*R*,14*S*,17*R*)-10,13-Dimethyl-17-((*R*)-6-methylheptan-2-yl)hexadecahydro-1*H*-cyclopenta[a]phenanthren-3-yl)(4-(trifluoromethyl)phenyl)methanone

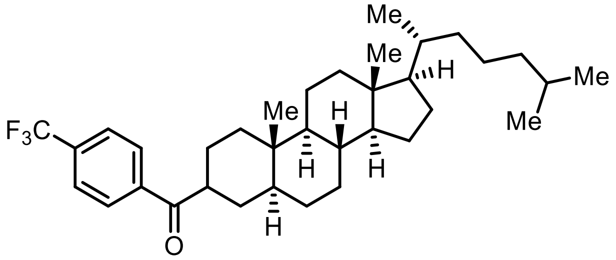

Formula: $C_{35}H_{51}F_3O$

MW: 544.8 g/mol

Synthesized following **GP-5**, using **7h** (1.11 g, 2.00 mmol, 1.00 equiv.), 4-(trifluoromethyl)benzaldehyde (348 mg, 2.00 mmol, 1.00 equiv.) and Cs_2CO_3 (977 mg, 3.00 mmol, 1.50 equiv.). The crude product was purified by flash column chromatography on silica gel (heptanes/EtOAc 100:0 to 98:2) to afford **8b** (802 mg, 1.47 mmol, 74%) as a pale-white solid.

Following column chromatography, **8b** was obtained contaminated with unreacted aldehyde and other undefined impurities of similar polarity. Despite these contaminants, **8b** was subjected to the subsequent step, silyl enol ether formation. The corresponding silyl enol ether, being less polar, was then easily separable from the aforementioned impurities due to the change in the relative polarities.

^{1}H NMR (400 MHz, CDCl$_3$): δ 8.02 (d, *J* = 8.1 Hz, 2H), 7.72 (d, *J* = 8.3 Hz, 2H), 3.29 (dt, *J* = 20.6, 7.8 Hz, 1H), 1.98 (dd, *J* = 12.5, 3.5 Hz, 1H), 1.91 – 1.78 (m, 2H), 1.78 – 1.63 (m, 3H), 1.61 – 1.55 (m, 1H), 1.53 – 1.44 (m, 3H), 1.40 – 1.20 (m, 10H), 1.19 – 0.97 (m, 10H), 0.91 (d, *J* = 6.5 Hz, 3H), 0.87 (d, *J* = 1.8 Hz, 3H), 0.86 (d, J = 1.8 Hz, 3H). 0.84 (s, 3H), 0.75 – 0.67 (m, 1H), 0.66 (s, 3H);

^{19}F NMR (376 MHz, CDCl$_3$): δ -63.07;

IR (neat) ν_{max}: 2951, 2929, 2867, 2846, 1688, 1620, 1582, 1512, 1467, 1445, 1410, 1383, 1323, 1265, 1245, 1213, 1166, 1130, 1109, 1067, 1016, 993, 975, 954, 931, 854, 819, 771 cm^{-1};

HRMS could not be recorded for this compound.

8c – [4-(trifluoromethyl)phenyl](3,3,5-trimethylcyclohexyl)methanone

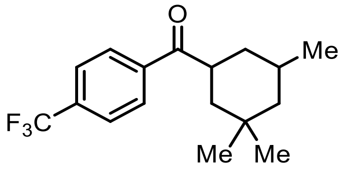

Formula: $C_{17}H_{21}F_3O$

MW: 298.3 g/mol

Synthesized following **GP-5**, using **7f** (0.620 g, 2.00 mmol, 1.00 equiv.), 4-(trifluoromethyl)benzaldehyde (0.270 mL, 2.00 mmol, 1.00 equiv.) and Cs_2CO_3 (0.980 g, 3.00 mmol, 1.50 equiv.). The crude product was purified by flash column chromatography on silica gel (heptanes/EtOAc 100:0 to 90:10) to afford **8c** (0.290 g, 0.980 mmol, 49%) as a colorless oil.

Following column chromatography, **8c** was obtained contaminated with unreacted aldehyde and other undefined impurities of similar polarity. Despite these contaminants, **8c** was subjected to the subsequent step, silyl enol ether formation. The corresponding silyl enol ether, being less polar, was then easily separable from the aforementioned impurities due to the change in the relative polarities.

^{1}H NMR (400 MHz, $CDCl_3$): δ 8.02 (d, *J* = 8.3 Hz, 2H), 7.73 (d, *J* = 8.3 Hz, 2H), 3.49 (tt, *J* = 12.3, 3.2 Hz, 1H), 1.86 – 1.67 (m, 2H), 1.60 – 1.52 (m, 1H), 1.49 – 1.40(m, 1H), 1.32 – 1.22 (m, 2H), 1.05 (s, 3H), 0.96 (s, 3H), 0.92 (d, *J* = 6.4 Hz, 3H); *One aliphatic proton could not be identified, as the 2–1 ppm region was complex.*

^{19}F NMR (376 MHz, $CDCl_3$): δ -63.1 (3F).

8f – Cyclopentyl[4-(trifluoromethyl)phenyl]methanone

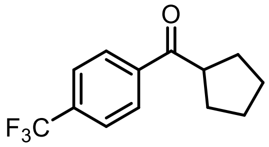

Formula: $C_{13}H_{13}F_3O$

MW: 242.2 g/mol

Synthesized following **GP-3**, using **6a** (3.14 g, 20.0 mmol, 1.00 equiv.) and **5a** (65.0 mL, 0.46 M in THF, 30.0 mmol, 1.50 equiv.). The crude product was purified by flash column chromatography on silica gel (heptanes/EtOAc 100:0 to 95:5) to afford **8f** (4.23 g, 17.5 mmol, 87%) as a colorless oil.

^{1}H NMR (400 MHz, CDCl$_3$): δ 8.07 (d, *J* = 8.3 Hz, 2H), 7.72 (d, *J* = 8.3 Hz, 2H), 3.75 – 3.66 (m, 1H), 2.00 – 1.85 (m, 4H), 1.79 – 1.61 (m, 4H);

^{13}C NMR (101 MHz, CDCl$_3$): δ 201.9, 139.8, 134.2 (q, *J* = 32.8 Hz), 128.9 (2C), 125.7 (q, *J* = 4.1 Hz, 2C), 123.8 (q, *J* = 272.1 Hz), 46.8, 29.9 (2C), 26.4 (2C);

^{19}F NMR (376 MHz, CDCl$_3$): δ -63.1 (3F);

IR (neat) ν_{max}: 2951, 2923, 2855, 1694, 1461, 1322, 1266, 1172, 1134, 1112, 1070, 858, 829, 746 cm^{-1};

HRMS (ESI$^+$): exact mass calculated for [M+H]$^+$ ($C_{13}H_{13}F_3OH^+$) requires *m/z* 243.0991, found *m/z* 243.0991.

8g – Cyclopentyl[3-(trifluoromethyl)phenyl]methanone

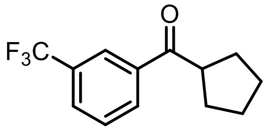

Formula: $C_{13}H_{13}F_3O$

MW: 242.2 g/mol

Synthesized following **GP-3**, using **6a** (314 mg, 2.00 mmol, 1.00 equiv.) and **5b** (8.20 mL, 0.49 M in THF, 4.00 mmol, 2.00 equiv.). The crude product was purified by flash column chromatography on silica gel (heptanes/EtOAc 100:0 to 95:5) to afford **8g** (450 mg, 1.86 mmol, 93%) as a colorless oil.

^{1}H NMR (400 MHz, CDCl$_3$): δ 8.22 (s, 1H), 8.15 (d, *J* = 7.8 Hz, 1H), 7.80 (dd, *J* = 7.8, 0.5 Hz, 1H), 7.61 (t, *J* = 7.8 Hz, 1H), 3.76 – 3.66 (m, 1H), 1.99 – 1.86 (m, 4H), 1.81 – 1.62 (m, 4H);

^{13}C NMR (101 MHz, CDCl$_3$): δ 201.5, 137.6, 131.7, 131.3 (q, *J* = 32.8 Hz), 129.33, 129.27 (q, *J* = 3.7 Hz), 125.4 (q, *J* = 3.8 Hz), 123.9 (q, *J* = 271.8 Hz), 46.6, 30.0 (2C), 26.4 (2C);

^{19}F NMR (376 MHz, CDCl$_3$): δ -62.8 (3F);

IR (neat) ν_{max}: 2957, 2934, 2872, 2208, 2111, 1691, 1658, 1645, 1616, 1575, 1466, 794 cm^{-1};

HRMS (ESI$^+$): exact mass calculated for $[M+H]^+$ ($C_{13}H_{13}F_3OH^+$) requires *m/z* 243.0991, found *m/z* 243.0992.

8h – Cyclopentyl[2-(trifluoromethyl)phenyl]methanone

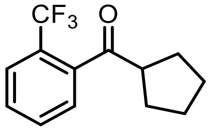

Formula: $C_{13}H_{13}F_3O$

MW: 242.2 g/mol

Synthesized following **GP-5**, using **7a** (1.00 g, 4.00 mmol, 1.00 equiv.), 2-trifluoromethylbenzaldehyde (0.540 mL, 4.00 mmol, 1.00 equiv.) and Cs_2CO_3 (1.90 g, 5.90 mmol, 1.50 equiv.). The crude product was purified by flash column chromatography on silica gel (heptanes/EtOAc 100:0 to 90:10) to afford **8h** (0.630 g, 2.60 mmol, 66%) as a colorless oil.

^{1}H NMR (400 MHz, $CDCl_3$): δ 7.71 (d, *J* = 7.7 Hz, 1H), 7.62 – 7.50 (m, 2H), 7.43 (d, *J* = 7.5 Hz, 1H), 3.42 (quint., *J* = 8.0 Hz, 1H), 1.96 – 1.82 (m, 4H), 1.79 – 1.67 (m, 2H), 1.67 – 1.56 (m, 2H);

^{13}C NMR (101 MHz, $CDCl_3$): δ 207.6, 140.9 (q, *J* = 2.0 Hz), 131.8, 129.9, 127.28, 127.26 (q, *J* = 32.1 Hz), 126.9 (q, *J* = 5.3 Hz), 123.8 (q, *J* = 273.0 Hz), 51.5, 29.7 (2C), 26.1 (2C);

^{19}F NMR (376 MHz, $CDCl_3$): δ -58.0 (3F);

IR (neat) ν_{max}: 2958, 2872, 1699, 1580, 1448, 1357, 1311, 1272, 1216, 1164, 1126, 1061, 1034, 998, 766 cm^{-1};

HRMS (ESI^+): exact mass calculated for $[M+Na]^+$ ($C_{13}H_{13}F_3ONa^+$) requires *m/z* 265.0811, found *m/z* 265.0807.

8i – 4-(Cyclopentanecarbonyl)benzonitrile

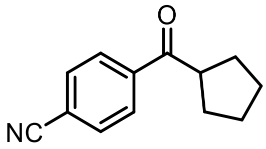

Formula: $C_{13}H_{13}NO$

MW: 199.2 g/mol

Synthesized following **GP-5**, using **7a** (1.00 g, 4.00 mmol, 1.00 equiv.), 4-cyanobenzaldehyde (0.460 mL, 4.20 mmol, 1.05 equiv.) and Cs_2CO_3 (2.00 g, 6.00 mmol, 1.50 equiv.). The crude product was purified by flash column chromatography on silica gel (heptanes/EtOAc 100:0 to 90:10) to afford **8i** (0.470 g, 2.40 mmol, 59%) as a colorless oil.

Spectral data were in accordance with the literature.[60]

^{1}H NMR (400 MHz, $CDCl_3$): δ 8.07 – 8.01 (m, 2H), 7.79 – 7.73 (m, 2H), 3.74-3.62 (m, 1H), 2.01 – 1.84 (m, 4H), 1.79 – 1.61 (m, 4H).

8j – Methyl 4-(cyclopentanecarbonyl)benzoate

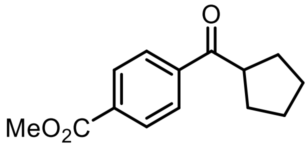

Formula: $C_{14}H_{16}O_3$

MW: 232.3 g/mol

Synthesized following **GP-5**, using **7a** (505 mg, 2.00 mmol, 1.00 equiv.), methyl 4-formylbenzoate (394 mg, 2.40 mmol, 1.20 equiv.) and Cs_2CO_3 (0.980 g, 3.00 mmol, 1.50 equiv.). The crude product was purified by flash column chromatography on silica gel (heptanes/EtOAc 100:0 to 90:10) to afford **8j** (276 mg, 1.19 mmol, 59%) as a colorless oil.

Spectral data were in accordance with literature.[61]

^{1}H NMR (400 MHz, $CDCl_3$): δ 8.13 – 8.09 (m, 2H), 8.03 – 7.99 (m, 2H), 3.95 (s, 3H), 3.77 – 3.66 (m, 1H), 2.00 – 1.86 (m, 4H), 1.80 – 1.61 (m, 4H).

8k – Cyclopentyl(2,6-dichlorophenyl)methanone

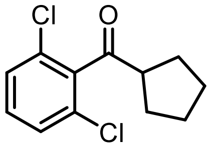

Formula: $C_{12}H_{12}Cl_2O$

MW: 243.1 g/mol

Synthesized following **GP-5**, using **7a** (505 mg, 2.00 mmol, 1.00 equiv.), 2,6-dichlorobenzaldehyde (350 mg, 2.00 mmol, 1.00 equiv.) and Cs_2CO_3 (977 mg, 3.00 mmol, 1.50 equiv.). The crude product was purified by flash column chromatography on silica gel (heptanes/EtOAc 100:0 to 95:5) to afford **8k** (349 mg, 1.28 mmol, 64%) as a colorless oil.

Following column chromatography, **8k** was obtained contaminated with unreacted aldehyde and other undefined impurities of similar polarity. Despite these contaminants, **8k** was subjected to the subsequent step, silyl enol ether formation. The corresponding silyl enol ether, being less polar, was then easily separable from the aforementioned impurities due to the change in the relative polarities.

^{1}H NMR (400 MHz, $CDCl_3$): δ 7.32 (d, *J* = 1.8 Hz, 1H), 7.30 (d, *J* = 0.4 Hz, 1H), 7.26 (s, 1H), 3.38 (p, *J* = 8.0 Hz, 1H), 2.07 – 1.96 (m, 2H), 1.95 – 1.85 (m, 2H), 1.82 – 1.71 (m, 2H), 1.69 – 1.58 (m, 2H).

8l – (4-Bromophenyl)(cyclopentyl)methanone

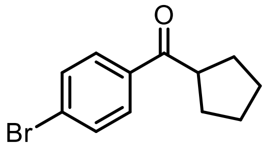

Formula: $C_{12}H_{13}BrO$

MW: 253.1 g/mol

Synthesized following **GP-5**, using **7a** (757 mg, 3.00 mmol, 1.00 equiv.), 4-bromobenzaldehyde (555 mg, 3.00 mmol, 1.00 equiv.) and Cs_2CO_3 (1.50 g, 4.50 mmol, 1.50 equiv.). The crude product was purified by flash column chromatography on silica gel (heptanes/EtOAc 100:0 to 90:10) to afford **8l** (556 mg, 2.20 mmol, 73%) as a colorless oil.

Following column chromatography, **8l** was obtained contaminated with unreacted aldehyde and other undefined impurities of similar polarity. Despite these contaminants, **8l** was subjected to the subsequent step, silyl enol ether formation. The corresponding silyl enol ether, being less polar, was then easily separable from the aforementioned impurities due to the change in the relative polarities.

Spectral data were in accordance with literature.[61]

^{1}H NMR (400 MHz, $CDCl_3$): δ 7.85 – 7.80 (m, 2H), 7.61 – 7.57 (m, 2H), 3.65 (quint., *J* = 7.8 Hz, 1H), 1.96 – 1.85 (m, 4H), 1.77 – 1.60 (m, 4H).

8m – Cyclopentyl(phenyl)methanone

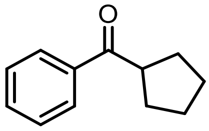

Formula: $C_{12}H_{14}O$

MW: 174.2 g/mol

Synthesized following **GP-3**, using **6a** (729 mg, 4.64 mmol, 1.00 equiv.) and phenylmagnesium bromide (2.00 mL, 2.8 M in Et_2O, 5.60 mmol, 1.20 equiv.). The crude product was purified by flash column chromatography on silica gel (heptanes/EtOAc 100:0 to 95:5) to afford **8m** (655 mg, 3.76 mmol, 81%) as a colorless oil.

Spectral data were in accordance with literature.[62]

^{1}H NMR (600 MHz, $CDCl_3$): δ 7.99 – 7.95 (m, 2H), 7.57 – 7.52 (m, 1H), 7.48 – 7.43 (m, 2H), 3.72 (quint., *J* = 7.8 Hz, 1H), 1.96 – 1.87 (m, 4H), 1.77 – 1.70 (m, 2H), 1.69 – 1.61 (m, 2H).

8n – cyclopentyl(naphthalen-2-yl)methanone

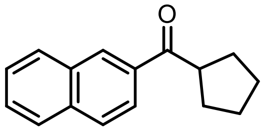

Formula: $C_{16}H_{16}O$

MW: 224.3 g/mol

Synthesized following **GP-5**, using **7a** (505 mg, 2.00 mmol, 1.00 equiv.), 2-naphthaldehyde (312 mg, 2.00 mmol, 1.00 equiv.) and Cs_2CO_3 (0.980 g, 3.00 mmol, 1.50 equiv.). The crude product was purified by flash column chromatography on silica gel (heptanes/EtOAc 100:0 to 90:10) to afford **8n** (251 mg, 1.12 mmol, 56%) as a colorless oil.

Following column chromatography, **8n** was obtained contaminated with unreacted aldehyde and other undefined impurities of similar polarity. Despite these contaminants, **8n** was subjected to the subsequent step, silyl enol ether formation. The corresponding silyl enol ether, being less polar, was then easily separable from the aforementioned impurities due to the change in the relative polarities.

Spectral data were in accordance with the literature.[63]

^{1}H NMR (400 MHz, $CDCl_3$): δ 8.49 (s, 1H), 8.05 (dd, *J* = 8.5, 1.8 Hz, 1H), 7.97 (m, 1H), 7.92 – 7.86 (m, 2H), 7.61 – 7.52 (m, 2H), 3.88 (quint., *J* = 7.8 Hz, 1H), 2.06 – 1.92 (m, 4H), 1.84 – 1.64 (m, 4H).

8o – Cyclohexyl[4-(trifluoromethyl)phenyl]methanone

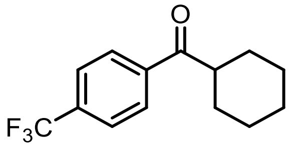

Formula: $C_{14}H_{15}F_3O$

MW: 256.3 g/mol

Synthesized following **GP-3**, using **6b** (856 mg, 5.00 mmol, 1.00 equiv.) and **5a** (16.0 mL, 0.46 M in THF, 7.50 mmol, 1.50 equiv.). The crude product was purified by flash column chromatography on silica gel (heptanes/EtOAc 100:0 to 98:2) to afford **8o** (936 mg, 3.70 mmol, 73%) as a colorless oil.

^{1}H NMR (400 MHz, CDCl$_3$): δ 8.03 (d, *J* = 8.1 Hz, 2H), 7.72 (d, *J* = 8.2 Hz, 2H), 3.25 (tt, *J* = 11.4, 3.2 Hz, 1H), 2.00 – 1.83 (m, 4H), 1.75 (ddd, *J* = 12.6, 4.6, 2.4 Hz, 1H), 1.56 – 1.16 (m, 5H);

^{13}C NMR (151 MHz, CDCl$_3$): δ 203.0, 139.3, 134.2 (q, *J* = 32.7 Hz), 128.7 (2C), 125.8 (q, *J* = 3.7 Hz, 2C), 123.8 (d, *J* = 272.6 Hz), 46.1, 29.4 (2C), 26.0, 25.9 (2C);

^{19}F NMR (376 MHz, CDCl$_3$): δ -63.08 (s);

IR (neat) ν_{max}: 2933, 2857, 1687, 1510, 1451, 1409, 1322, 1249, 1206, 1167, 1128, 1111, 1066, 1016, 975, 894, 849, 818, 789 cm^{-1};

HRMS (ESI$^+$): exact mass calculated for $[M+H]^+$ ($C_{14}H_{15}F_3OH^+$) requires *m/z* 257.1148, found *m/z* 257.1149.

8p – Benzo[*b*]thiophen-2-yl(cyclohexyl)methanone

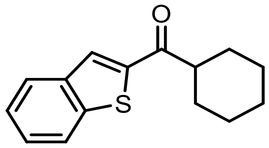

Formula: $C_{15}H_{16}OS$

MW: 244.4 g/mol

Synthesized following **GP-5**, using **7b** (533 mg, 2.00 mmol, 1.00 equiv.), benzo[*b*]thiophene-2-carboxaldehyde (331 mg, 2.00 mmol, 1.00 equiv.) and Cs_2CO_3 (977 mg, 3.00 mmol, 1.50 equiv.). The crude product was purified by flash column chromatography on silica gel (heptanes/EtOAc 100:0 to 90:10) to afford **8p** (322 mg, 1.32 mmol, 66%) as a colorless oil.

Spectral data were in accordance with the literature.[64]

^{1}H NMR (400 MHz, CDCl$_3$): δ 7.97 (s, 1H), 7.88 (t, *J* = 8.2 Hz, 2H), 7.43 (dtd, *J* = 14.8, 7.1, 1.2 Hz, 2H), 3.24 (tt, *J* = 11.6, 3.4 Hz, 1H), 1.97 (d, *J* = 13.5 Hz, 2H), 1.93 – 1.85 (m, 2H), 1.76 (ddd, *J* = 12.7, 4.9, 2.5 Hz, 1H), 1.67 – 1.55 (m, 2H), 1.48 – 1.36 (m, 2H), 1.36 – 1.24 (m, 1H).

8q – [3-(*tert*-Butyl)cyclopentyl][4-(trifluoromethyl)phenyl]methanone

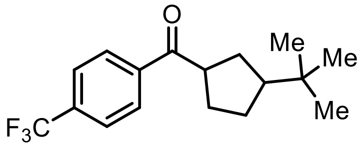

Formula: $C_{17}H_{21}F_3O$

MW: 298.3 g/mol

Synthesized following **GP-5**, using **7e** (0.620 g, 2.00 mmol, 1.00 equiv.), 4-trifluoromethylbenzaldehyde (0.290 mL, 2.10 mmol, 1.05 equiv.) and Cs_2CO_3 (0.980 g, 3.00 mmol, 1.50 equiv.). The crude product was purified by flash column chromatography on silica gel (heptanes/EtOAc 100:0 to 90:10) to afford **8q** (338 mg, 1.13 mmol, 57%, *cis*/*trans* = 1:1) as a colorless oil.

Following column chromatography, **8q** was obtained contaminated with unreacted aldehyde and other undefined impurities of similar polarity. Despite these contaminants, **8q** was subjected to the subsequent step, silyl enol ether formation. The corresponding silyl enol ether, being less polar, was then easily separable from the aforementioned impurities due to the change in the relative polarities.

^{1}H NMR (400 MHz, $CDCl_3$): δ 8.05 (d, *J* = 8.2 Hz, 2H), 7.72 (d, *J* = 8.2 Hz, 2H), 3.78 – 3.65 (m, 1H), 2.05 – 1.57 (m, 6H), 1.51 – 1.35 (m, 1H), 0.89 (s, 4.5H), 0.88 (s, 4.5H);

^{19}F NMR (376 MHz, $CDCl_3$): δ -63.1 (3F).

8r – (3-Methylcyclopentyl)[4-(trifluoromethyl)phenyl]methanone

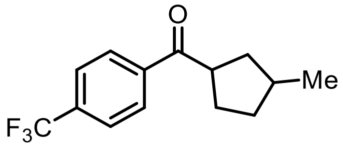

Formula: $C_{14}H_{15}F_3O$

MW: 256.3 g/mol

Synthesized following **GP-5**, using **7c** (1.10 g, 4.00 mmol, 1.00 equiv.), 4-trifluoromethylbenzaldehyde (0.550 mL, 4.00 mmol, 1.00 equiv.) and Cs_2CO_3 (2.00 g, 6.00 mmol, 1.50 equiv.). The crude product was purified by flash column chromatography on silica gel (heptanes/EtOAc 100:0 to 90:10) to afford **8r** (656 mg, 2.56 mmol, 64%, *cis*/*trans* = 1:1) as a colorless oil.

Following column chromatography, **8r** was obtained contaminated with unreacted aldehyde and other undefined impurities of similar polarity. Despite these contaminants, **8r** was subjected to the subsequent step, silyl enol ether formation. The corresponding silyl enol ether, being less polar, was then easily separable from the aforementioned impurities due to the change in the relative polarities.

^{1}H NMR (400 MHz, $CDCl_3$): δ 8.05 (d, *J* = 8.1 Hz, 2H), 7.71 (d, *J* = 8.1 Hz, 2H), 3.85 – 3.68 (m, 1H), 2.15 – 1.99 (m, 3H), 1.98 – 1.80 (m, 2H), 1.55 – 1.42 (m, 1H), 1.34 – 1.20 (m, 1H), 1.05 (d, *J* = 6.3 Hz, 1.5H), 1.03 (d, *J* = 6.4 Hz, 1.5H);

^{19}F NMR (376 MHz, $CDCl_3$): δ -63.1 (3F).

8t – *trans*-(4-Butylcyclohexyl)[4-(trifluoromethyl)phenyl]methanone

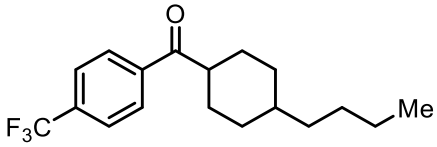

Formula: $C_{18}H_{23}F_{3}O$

MW: 312.4 g/mol

Synthesized following **GP-3**, using **6d** (386 mg, 1.70 mmol, 1.00 equiv.) and **5a** (7.60 mL, 0.45 M in THF, 3.40 mmol, 2.00 equiv.). The crude product was purified by flash column chromatography on silica gel (heptanes/EtOAc 100:0 to 90:10) to afford **8t** (424 g, 1.36 mmol, 80%) as a colorless oil.

^{1}H NMR (400 MHz, CDCl$_3$): δ 8.03 (d, *J* = 8.0 Hz, 2H), 7.72 (d, *J* = 8.0 Hz, 2H), 3.19 (tt, *J* = 11.9, 3.0 Hz, 1H), 1.95 – 1.85 (m, 4H), 1.51 (qd, *J* = 12.5, 3.8 Hz, 2H), 1.35 – 1.19 (m, 7H), 1.04 (qd, *J* = 14.0, 4.1 Hz, 2H), 0.90 (t, *J* = 7.2 Hz, 3H);

^{13}C NMR (101 MHz, CDCl$_3$): δ 203.1, 139.3, 134.2 (q, *J* = 33.5 Hz), 128.7 (2C), 125.8 (q, *J* = 3.7 Hz, 2C), 123.8 (q, *J* = 273.0 Hz), 46.4, 37.2, 37.1, 32.7 (2C), 29.4 (2C), 29.2, 23.1, 14.3;

^{19}F NMR (376 MHz, CDCl$_3$): δ -63.1 (3F);

IR (neat) ν_{max}: 2951, 2916, 2851, 1682, 1326, 1167, 1120, 1067, 1015, 997, 843 cm^{-1};

HRMS (ESI$^+$): exact mass calculated for $[M+H]^+$ ($C_{18}H_{23}F_{3}OH^+$) requires *m/z* 313.1774, found *m/z* 313.1774.

8u – [4-(*tert*-Butyl)cyclohexyl][4-(trifluoromethyl)phenyl]methanone

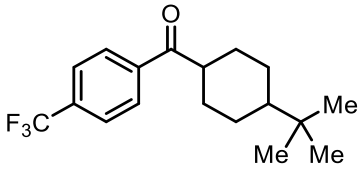

Formula: $C_{18}H_{23}F_3O$

MW: 312.4 g/mol

Synthesized following **GP-3**, using **6c** (1.16 g, 5.10 mmol, 1.00 equiv.) and **5a** (15 mL, 0.51 M in THF, 7.60 mmol, 1.50 equiv.). The crude product was purified by flash column chromatography on silica gel (heptanes/EtOAc 100:0 to 90:10) to afford **8u** (1.04 g, 3.35 mmol, 66%, d.r. 6:4) as a colorless oil.

^{1}H NMR (400 MHz, CDCl$_3$): δ 8.03 (d, *J* = 8.2 Hz, 1.2H), 7.95 (d, *J* = 8.1 Hz, 0.8H), 7.74 – 7.68 (m, 2H), 3.51 (m, 0.4H), 3.17 (tt, *J* = 11.9, 3.1 Hz, 0.6H), 2.21 – 2.12 (m, 0.8H), 2.01 – 1.88 (m, 2.2H), 1.72 – 1.58 (m, 1.8H), 1.54 - 142 (m, 1.2H), 1.31 – 0.96 (m, 3H), 0.88 (s, 5.4H), 0.82 (s, 3.6H);

^{13}C NMR (101 MHz, CDCl$_3$): δ [203.5 and 203.1], [139.9 and 139.3], [134.2 (q, *J* = 32.6 Hz) and 133.8 (q, *J* = 32.2 Hz)], [128.7 and 128.8] (2C), [125.8 (q, *J* = 3.6 Hz), 125.7 (q, *J* = 4.2 Hz)] (2C), [123.82 (q, *J* = 272.4 Hz) and 123.79 (q, *J* = 272.8 Hz)], [48.2 and 47.6], [46.3 and 41.1], [32.7 and 32.6], [29.8 and 28.2] (2C), [27.64 and 27.60] (3C), [26.9 and 23.5] (2C);

^{19}F NMR (376 MHz, CDCl$_3$): δ [-63.0 and -63.1] (3F);

IR (neat) ν_{max}: 2943, 2854, 1676, 1408, 1365, 1323, 1164, 1126, 1067, 1014, 977, 861 cm^{-1};

HRMS (ESI$^+$): exact mass calculated for $[M+H]^+$ ($C_{18}H_{23}F_3OH^+$) requires *m/z* 313.1774, found *m/z* 313.1772.

8v – [4-(*tert*-Butyl)cyclohexyl][3-(trifluoromethyl)phenyl]methanone

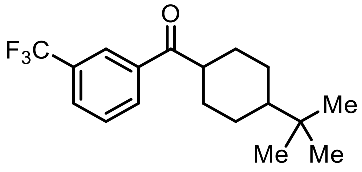

Formula: $C_{18}H_{23}F_3O$

MW: 312.4 g/mol

Synthesized following **GP-3**, using **6c** (455 mg, 2.00 mmol, 1 equiv.) and **5b** (8.20 mL, 0.49 M in THF, 4.00 mmol, 2.00 equiv.). The crude product was purified by flash column chromatography on silica gel (heptanes/EtOAc 100:0 to 95:5) to afford **8v** (456 mg, 1.46 mmol, 73%, 6:4 d.r.) as a colorless oil.

^{1}H NMR (700 MHz, CDCl$_3$): δ 8.18 (s, 0.6H), 8.13 – 8.10 (m, 1H), 8.04 (d, *J* = 7.8 Hz, 0.4H), 7.81 – 7.76 (m, 1H), 7.62 – 7.56 (m, 1H), 3.55 – 3.51 (m, 0.4 H), 3.18 (tt, *J* = 12.0, 3.3 Hz, 0.6H), 2.18 – 2.13 (m, 0.8H), 1.99 – 1.89 (m, 2H), 1.71 – 1.64 (m, 0.8H), 1.63 – 1.56 (m, 1.2H), 1.51 (qd, *J* = 12.2, 3.0 Hz, 1.2H), 1.26 (qd, *J* = 12.5, 3.3 Hz, 0.8H), 1.17 (qd, *J* = 12.7, 3.0 Hz, 1.2H), 1.08 (tt, *J* = 11.9, 2.9 Hz, 0.6H), 1.01 (tt, *J* = 12.1, 3.4 Hz, 0.4H), 0.88 (s, 5.4H), 0.83 (s, 3.6H);

^{13}C NMR (176 MHz, CDCl$_3$): δ [202.9 and 202.7], [137.5 and 137.1], [131.54 and 131.50], [131.4 (q, *J* = 32.3 Hz) and 131.3 (q, *J* = 32.7 Hz)], [129.4 and 129.28], [129.32 (q, *J* = 4.0 Hz) and 128.9 (q, *J* = 3.8 Hz)], 125.2 (q, *J* = 3.8 Hz), [123.91 (q, *J* = 271.0 Hz) and 123.89 (q, *J* = 272.1 Hz)], [48.2 and 47.6], [46.1 and 40.8], [32.7 and 32.8], [29.9 and 28.2] (2C), [27.64 and 27.60] (3C), [26.9 and 23.5] (2C);

^{19}F NMR (659 MHz, CDCl$_3$): δ -62.7 (3F);

IR (neat) ν_{max}: 2941, 2863, 1687, 1610, 1365, 1327, 1248, 1198, 1166, 1126, 1097, 1072, 694 cm^{-1};

HRMS (ESI$^+$): exact mass calculated for $[M+H]^+$ ($C_{18}H_{23}F_3OH^+$) requires *m/z* 313.1774, found *m/z* 313.1774.

8w – (4-Methylcyclohexyl)[4-(trifluoromethyl)phenyl]methanone

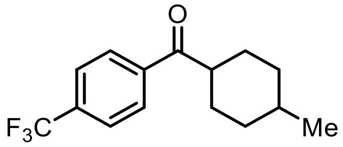

Formula: $C_{15}H_{17}F_3O$

MW: 270.3 g/mol

Synthesized following **GP-5**, using **7g** (1.12 g, 4.00 mmol, 1.00 equiv.), 4-(trifluoromethyl)benzaldehyde (0.58 mL, 4.20 mmol, 1.05 equiv.) and Cs_2CO_3 (2.00 g, 6.00 mmol, 1.50 equiv.). The crude product was purified by flash column chromatography on silica gel (heptanes/EtOAc 100:0 to 90:10) to afford **8w** (762 mg, 2.82 mmol, 70%, 8:2 d.r.) as a colorless oil.

Following column chromatography, **8w** was obtained contaminated with unreacted aldehyde and other undefined impurities of similar polarity. Despite these contaminants, **8w** was subjected to the subsequent step, silyl enol ether formation. The corresponding silyl enol ether, being less polar, was then easily separable from the aforementioned impurities due to the change in the relative polarities.

^{1}H NMR (400 MHz, CDCl$_3$): δ 8.02 (d, J = 8.2 Hz, 1.6H), 7.98 (d, J = 8.3 Hz, 0.4H), 7.74 – 7.69 (m, 2H), 3.35 (tt, J = 7.0, 4.5 Hz, 0.2H), 3.18 (tt, J = 11.9, 3.3 Hz, 0.8H), 1.96 – 1.34 (m, 7.4H), 1.07 (qd, J = 11.8, 3.1 Hz, 1.6H), 0.94 (d, J = 6.8 Hz, 0.6H), 0.93 (d, J = 6.5 Hz, 2.4H);

^{19}F NMR (376 MHz, CDCl$_3$): δ [-63.08 and -63.22] (3F);

IR (neat) ν_{max}: 2949, 2926, 2852, 1686, 1673, 1453, 1408, 1322, 1253, 1203, 1164, 1123, 1110, 1065, 1013, 947 cm^{-1};

HRMS (ESI$^+$): exact mass calculated for $[M+H]^+$ ($C_{15}H_{17}F_3OH^+$) requires m/z 271.1304, found m/z 271.1304.

3.5 Preparation of 8s

Ketone **8s** was prepared following an alternative route.

NB: This route was initially explored as a general procedure, but the hydrazone route proved to be more performant.

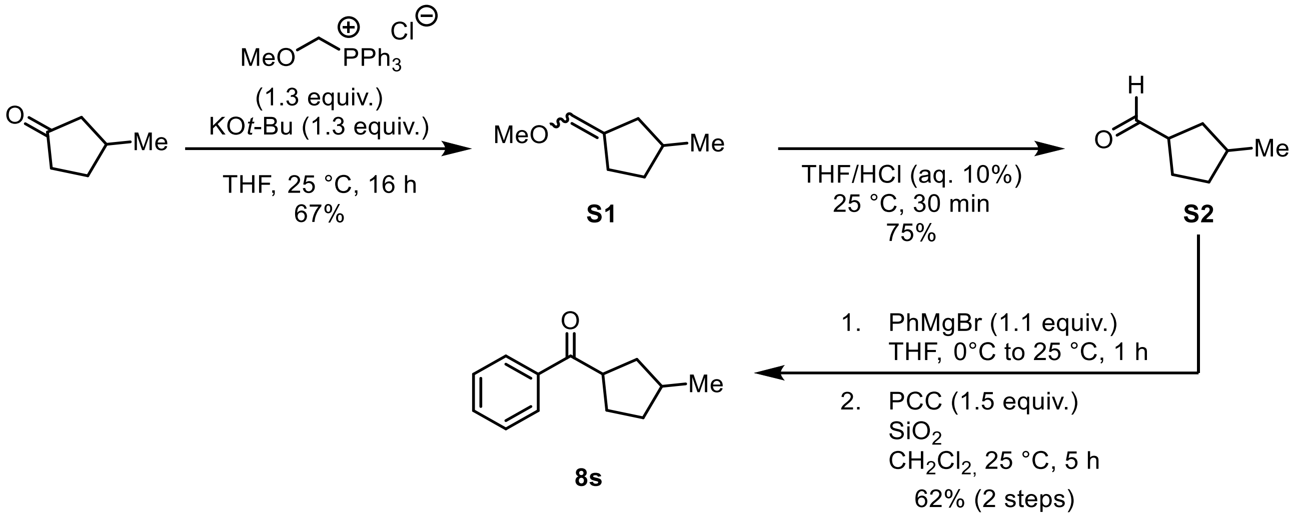

Figure S2. Preparation of **8s**.

S1 – 1-(Methoxymethylene)-3-methylcyclopentane

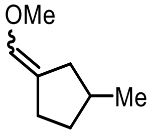

MW = 126.2 g/mol

Formula: $C_8H_{14}O$

Adapted from a known procedure.[65]

To a 0 °C suspension of (methoxymethyl)triphenylphosphonium chloride (4.50 g, 13.0 mmol, 1.30 equiv.) in THF (40 mL) was added *t*-BuOK (1.50 g, 13.0 mmol, 1.30 equiv.) and the resulting red mixture was left stirring at 0 °C for 1 h. A solution of 3-methylcyclopentanone (1.10 mL, 10.0 mmol, 1.00 equiv.) in THF (20 mL) was then added dropwise and the mixture was left stirring at rt for 16 h. Et_2O was subsequently added, followed by sat. aq. NH_4Cl, and the phases were separated. The aqueous phase was extracted with Et_2O (3 x 10 mL). The combined organic phases were washed with sat. aq. NaCl, dried over $MgSO_4$, filtered, and concentrated *in vacuo*. Et_2O was added and the precipitated triphenylphosphine oxide was removed by filtration. The filtrate was concentrated *in vacuo* (careful: volatile product, 40 °C, 457 mbar). The crude product was subjected to bulb-to-bulb distillation (100 °C to 140 °C, 40 mbar) to afford **S1** (851 mg, 6.74 mmol, 67%, (*E*)/(*Z*) = 1:1) as a colorless oil.

^{1}H NMR (600 MHz, $CDCl_3$): δ = 5.87 – 5.84 (m, 1H), 3.545 (s, 1.5H), 3.542 (s, 1.5H), 2.47 (dd, *J* = 16.6, 8.0 Hz, 0.5H), 2.39 – 2.13 (m, 2.5H), 2.03 – 1.88 (m, 1H), 1.84 – 1.73 (m, 2H), 1.25 – 1.11 (m, 1H), 1.00 (d, *J* = 6.7 Hz, 1.5H), 0.97 (d, *J* = 6.7 Hz, 1.5H);

^{13}C NMR (151 MHz, $CDCl_3$): δ = [138.8 and 138.7], 120.9, 59.4, [37.7 and 35.6], [35.00 and 34.4], [34.97 and 34.6], [28.6 and 26.5], [20.2 and 19.7];

IR (neat) ν_{max}: 2950, 2930, 2868, 2831, 1744, 1727, 1692, 1456, 1439, 1376, 1356, 1222, 1176, 1114, 734 cm^{-1};

MS (QTOF, EI$^+$, 70 eV): *m*/*z* 126 (100) [$M^{+\bullet}$], 111 (96) [M^+-CH_3], 97 (18), 84 (48), 81 (63), 79 (84), 69 (32).

S2 – 3-Methylcyclopentane-1-carbaldehyde

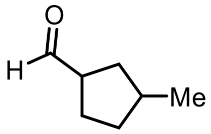

MW = 112.2 g/mol

Formula: $C_7H_{12}O$

At rt, aq. 10% HCl (60 mL) was added to a solution of **S1** (0.76 g, 6.00 mmol, 1.00 equiv.) in THF (60 mL), and the resulting mixture was stirred vigorously for 30 min. Et_2O was then added and the phases were separated. The aqueous phase was extracted with Et_2O (3 x 30 mL). The combined organic phases were washed with water and sat. aq. NaCl, then dried over $MgSO_4$, filtered and concentrated *in vacuo* (careful: volatile product). The crude product was purified by flash column chromatography on silica gel (pentane/Et_2O, 100:0 to 95:5) to afford **S2** (0.500 mg, 4.50 mmol, 75%, *cis*/*trans* = 1:1) as a colorless oil.

^{1}H NMR (600 MHz, $CDCl_3$): δ = 9.607 (s, 0.5H), 9.603 (s, 0.5H), 2.86 – 2.79 (m, 0.5H), 2.79 – 2.73 (m, 0.5H), 2.08 – 1.75 (m, 5H), 1.40 – 1.31 (m, 1H), 1.23 – 1.09 (m, 1H), 1.02 (d, *J* = 6.3 Hz, 1.5H), 0.99 (d, *J* = 6.6 Hz, 1.5H);

^{13}C NMR (151 MHz, $CDCl_3$): δ = 204.2, [52.0 and 51.2], [35.3 and 34.38], [35.3 and 34.6], [34.7 and 34.36], [26.1 and 25.9], [20.2 and 20.0];

IR (neat) ν_{max}: 2958, 2925, 2868, 2855, 1783, 1462, 1252 cm^{-1}.

HRMS could not be recorded for this compound.

8s – (3-Methylcyclopentyl)(phenyl)methanone

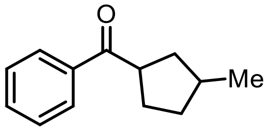

Formula: $C_{13}H_{16}O$

MW: 188.3 g/mol

A solution of phenylmagnesium bromide in Et_2O (2.8 M, 1.40 mL, 3.90 mmol, 1.10 equiv.) was added to a cooled (0 °C) solution of **S2** (0.400 g, 3.60 mmol, 1.00 equiv.) in THF (20 mL), and the resulting mixture was warmed to rt and stirred for 1 h. Following cooling to 0 °C, sat. aq. NH_4Cl was added and the mixture was again warmed to rt. The phases were separated and the aqueous phase was extracted with Et_2O (3 x 30 mL). The combined organic phases were washed with sat. aq. NaCl, dried over $MgSO_4$, filtered and concentrated *in vacuo* to afford the crude alcohol, which was used in the next step without further purification.

At rt, a solution of the previously obtained crude alcohol in CH_2Cl_2 (4 mL) was added to a suspension of pyridinium chlorochromate (1.20 g, 5.30 mmol, 1.50 equiv.) and silica (2.40 g) in CH_2Cl_2 (10 mL), and the resulting mixture was left stirring for 5 h. Subsequently, Et_2O (*ca* 50 mL) was added and the mixture was vigorously stirred for 1 h. The mixture was filtered through a short silica plug, eluting with Et_2O, and the filtrate was concentrated *in vacuo*. The crude product was purified by flash column chromatography on silica gel (heptanes/EtOAc, 100:0 to 90:10) to afford **8s** (419 mg, 2.23 mmol, 62%) as a colorless oil.

Spectral data were in accordance with the literature.[66]

^{1}H NMR (400 MHz, $CDCl_3$): δ 7.89 (d, *J* = 7.9 Hz, 2H), 7.47 (t, *J* = 7.0 Hz, 1H), 7.38 (t, *J* = 7.2 Hz, 2H), 3.80 – 3.64 (m, 1H), 2.10 – 1.98 (m, 3H), 1.97 – 1.89 (m, 1H), 1.28 – 1.12 (m, 2H), 0.97 (d, *J* = 6.4 Hz, 1.5H), 0.95 (d, *J* = 6.5 Hz, 1.5H).

3.6 Silyl enol ethers 1a-1c and 1f-1w

1a – Trimethyl{(3-methylcyclohexylidene)[4-(trifluoromethyl)phenyl]methoxy}silane

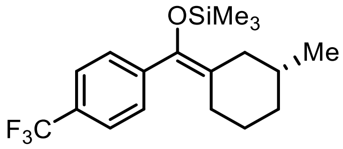

Formula: $C_{18}H_{25}F_3OSi$

MW: 342.5 g/mol

Synthesized following **GP-6**, using **8a** (0.410 g, 1.50 mmol, 1.00 equiv.), Me_3SiOTf (1.80 mL, 9.75 mmol, 6.50 equiv.) and Et_3N (2.10 mL, 15.0 mmol, 10.0 equiv.). The crude product was purified by flash column chromatography on silica gel (heptanes/EtOAc 100:0 to 95:5) to afford **1a** (0.370 g, 1.10 mmol, 71%, *E*/*Z* = 1:1) as a colorless oil.

(–)-(*R*)-1a was obtained by the same procedure starting from **(–)-(*R*)-8a**. See **(–)-(*R*)-7d** for details regarding *ee* calculations)

^{1}H NMR (400 MHz, $CDCl_3$): δ 7.57 (d, *J* = 8.3 Hz, 1H), 7.56 (d, *J* = 8.3 Hz, 1H), 7.40 (d, *J* = 8.3 Hz, 2H), 2.91 – 2.79 (m, 1H), 2.40 – 2.30 (m, 1H), 1.87 – 1.68 (m, 3H), 1.58 – 1.20 (m, 3H), 1.11 – 1.04 (m, 1H), 0.98 (d, *J* = 6.1 Hz, 1.5H), 0.85 (d, *J* = 6.4 Hz, 1.5H), -0.02 (s, 4.5H), -0.03 (s, 4.5H);

^{13}C NMR (151 MHz, $CDCl_3$): δ [142.8 and 142.7], [140.2 and 140.1], [129.6 and 129.5] (2C), 129.2 (q, *J* = 32.5 Hz), [124.87 and 124.85] (q, *J* = 3.7 Hz, 2C), 124.3 (q, *J* = 271.8 Hz), [122.6 and 122.5], [38.1 and 36.3], [35.3 and 35.2], [34.1 and 33.7], [29.4 and 26.4], 27.4, [22.7 and 22.4], [0.51 and 0.49] (3C);

^{19}F NMR (376 MHz, $CDCl_3$): δ [-62.44 and -62.45] (3F);

IR (neat) ν_{max}: 2956, 2924, 2842, 1322, 1252, 1218, 1164, 1124, 1105, 1064, 1017, 900, 865, 839, 750 cm^{-1};

MS (QTOF, EI^+, 70 eV): *m/z* 342 [$M^{+\bullet}$] (96), 327 [M^+-Me] (28), 273 [M^+-CF_3] (70), 191 (58), 73 [Me_3Si^+] (100). *HRMS could not be recorded for this compound.*

$[\alpha]_D^{20}$ –32.9 (c = 1.0, $CHCl_3$).

1b – (((5*S*,8*R*,9*S*,10*S*,13*R*,14*S*,17*R*)-10,13-dimethyl-17-((*R*)-6-methylheptan-2-yl)hexa decahydro-3*H*-cyclopenta[a]phenanthren-3-ylidene)(4-trifluoromethyl)phenyl)methoxy) trimethylsilane

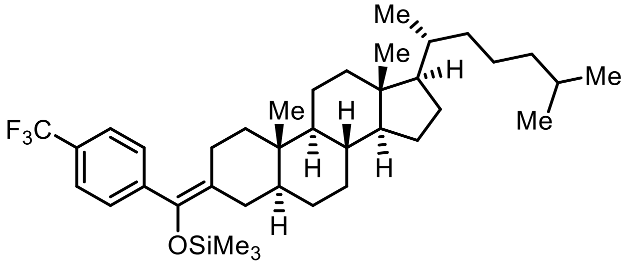

Formula: $C_{38}H_{59}F_3OSi$

MW: 616.0 g/mol

Synthesized following **GP-6**, using **8b** (163 mg, 0.300 mmol, 1.00 equiv.), Et_3N (0.420 mL, 3.00 mmol, 10.0 equiv.) and Me_3SiOTf (0.360 mL, 1.95 mmol, 6.50 equiv.). The crude product was purified by flash column chromatography on silica gel (heptanes/EtOAc 100:0 to 96:4) to afford **1b** (131 mg, 0.210 mmol, 71%, *E*/*Z* = 1:1) as a colorless solid.

^{1}H NMR (400 MHz, $CDCl_3$): δ 7.56 (d, *J* = 8.0 Hz, 2H), 7.41 (d, *J* = 8.0 Hz, 2H), 2.83 (d, *J* = 14.5 Hz, 0.5H), 2.57 (d, *J* = 12.7 Hz, 0.5H), 2.25 (s, 0.5H), 2.05 (d, *J* = 18.8 Hz, 0.5H), 2.02 – 1.58 (m, 7H), 1.51 – 0.94 (m, 22H), 0.92 – 0.89 (m, 3H), 0.88 – 0.86 (m, 6H), 0.85 (d, *J* = 1.6 Hz, 3H), 0.63 (d, *J* = 17.1 Hz, 3H), -0.03 (s, 9H);

^{13}C NMR (101 MHz, $CDCl_3$): δ 147.4, 142.8, 130.6, 129.6, 129.5, 124.8, 124.8, 122.8, 122.6,

56.6, 56.4, 54.5, 47.8, 47.2, 42.8, 40.2, 39.7, 38.9, 36.3, 36.0, 35.6, 32.3, 30.4, 29.0, 28.4, 28.2,

24.4, 24.0, 23.5, 23.0, 22.7, 21.3, 18.8, 12.2, 11.9, 0.5 (3C);

^{19}F NMR (376 MHz, $CDCl_3$): δ -62.4;

IR (neat) ν_{max}: 2955, 2928, 2868, 2848, 1325, 1267, 1251, 1164, 1128, 1105, 1089, 1067, 883, 866, 844, 746 cm^{-1};

HRMS (ESI$^+$): exact mass calculated for $[M+H]^+$ ($C_{38}H_{59}F_3OSiH^+$) requires *m/z* 617.4360, found *m/z* 617.4359.

1c – Trimethyl{[4-(trifluoromethyl)phenyl](3,3,5-trimethylcyclohexylidene)methoxy}silane

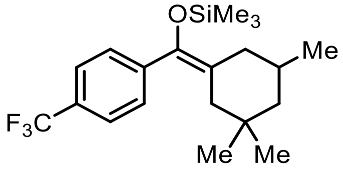

Formula: $C_{20}H_{29}F_3OSi$

MW: 370.5 g/mol

To a 0 °C solution of LiHMDS (1 M in THF, 0.840 mL, 0.840 mmol, 1.50 equiv.) was added a solution of **8c** (167 mg, 0.560 mmol, 1.00 equiv.) in THF (4.00 mL) and the mixture was left stirring for 30 min at the same temperature. Then, TMSCl (0.110 mL, 0.840 mmol, 1.50 equiv.) was added and the mixture was warmed to rt and left stirring overnight. The mixture was cooled to 0 °C and a sat. aq. solution of $NaHCO_3$ was added followed by Et_2O. The phases were separated and the aqueous phase was extracted with Et_2O (3 x 5 mL). The combined organic phases were dried over $MgSO_4$, filtered and concentrated *in vacuo*. The crude product was purified by flash column chromatography on silica gel (heptanes) to afford **1c** (71.0 mg, 0.190 mmol, 34%) as a colorless oil.

E/Z isomerism cannot be observed by NMR.

^{1}H NMR (600 MHz, $CDCl_3$): δ 7.57 (d, *J* = 8.1 Hz, 2H), 7.41 (d, *J* = 8.1 Hz, 2H), 2.64 (dt, *J* = 13.1, 2.0 Hz, 1H), 2.36 – 2.30 (m, 1H), 1.63 – 1.49 (m, 1H), 1.53 (d, *J* = 13.1 Hz, 1H), 1.45 – 1.33 (m, 1H), 1.36 (t, *J* = 12.7 Hz, 1H), 1.00 (s, 3H), 0.94 (t, *J* = 12.7 Hz, 1H), 0.90 (s, 3H), 0.80 (d, *J* = 6.3 Hz, 3H), -0.03 (s, 9H);

^{13}C NMR (151 MHz, $CDCl_3$): δ 142.9, 141.6, 129.7 (2C), 129.3 (q, *J* = 32.0 Hz), 124.9 (q, *J* = 3.9 Hz, 2C), 124.4 (q, *J* = 272.4 Hz), 120.6, 49.0, 40.5, 38.1, 33.2, 32.8, 29.8, 25.7, 22.7, 0.74 (3C);

^{19}F NMR (565 MHz, $CDCl_3$): δ -62.4 (3F);

IR (neat) ν_{max}: 2954, 2901, 2869, 2833, 1457, 1406, 1324, 1266, 1253, 1216, 1168, 1128, 1100, 1067, 1018, 895, 876, 845 cm^{-1};

MS (QTOF, EI$^+$, 70 eV): *m/z* 370 [$M^{+\bullet}$] (100), 355 [M^+-Me] (22), 287 (72), 247 (20), 195 (57), 191 (30), 73 [$SiMe_3^+$] (88). *HRMS could not be recorded for this compound.*

1f – {Cyclopentylidene[4-(trifluoromethyl)phenyl]methoxy}trimethylsilane

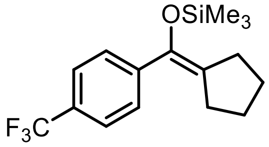

Formula: $C_{16}H_{21}F_3OSi$

MW: 314.4 g/mol

To a cooled (0 °C) solution of the **8f** (3.88 g, 16.0 mmol, 1.00 equiv.) and Et_3N (13.4 mL, 96.0 mL, 6.00 equiv.) in CH_2Cl_2 (25.0 mL), Me_3SiOTf (5.90 mL, 32.0 mmol, 2.00 equiv.) was added dropwise, and the resulting mixture was warmed to rt. After 16 h, the mixture was cooled to 0 °C and a sat. aq. solution of $NaHCO_3$ was added slowly. The phases were separated and the aqueous phase was extracted with CH_2Cl_2 (3 x 20 mL). The combined organic phases were dried over $MgSO_4$, filtered and concentrated under reduced pressure. The residue was purified by flash column chromatography on silica gel (heptanes/EtOAc 100:0 to 98:2) to afford **1f** (4.61 g, 14.7 mmol, 92%) as a colorless oil that slowly solidify upon standing at –20 °C.

^{1}H NMR (400 MHz, $CDCl_3$): δ 7.58 – 7.48 (m, 4H), 2.48 – 2.36 (m, 4H), 1.72 – 1.62 (m, 4H), 0.06 (s, 9H);

^{13}C NMR (101 MHz, $CDCl_3$): δ 143.2, 139.9, 128.7, 128.6 (q, J = 32.5 Hz), 127.7 (2C), 124.8 (q, J = 4.0 Hz, 2C), 124.4 (q, J = 271 Hz), 31.4, 30.8, 27.8, 25.9, 0.75 (3C);

^{19}F NMR (376 MHz, $CDCl_3$): δ -62.4 (3 F);

IR (neat) ν_{max}: 2957, 2870, 1616, 1408, 1322, 1252, 1164, 1123, 1097, 1066, 1015, 870, 840 cm^{-1};

HRMS could not be recorded for this compound. HRMS is given for the corresponding ketone:

HRMS (ESI$^+$): exact mass calculated for $[M+H]^+$ ($C_{13}H_{13}F_3O^+$) requires *m/z* 243.0991, found *m/z* 243.0991.

1g – {Cyclopentylidene[3-(trifluoromethyl)phenyl]methoxy}trimethylsilane

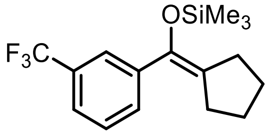

Formula: $C_{16}H_{21}F_3OSi$

MW: 314.4 g/mol

Synthesized following **GP-6**, using **8g** (0.240 g, 1.00 mmol, 1.00 equiv.), Me_3SiOTf (1.20 mL, 6.50 mmol, 6.50 equiv.) and Et_3N (1.40 mL, 10.0 mmol, 10.0 equiv.). The crude product was purified by flash column chromatography on silica gel (heptanes/EtOAc 100:0 to 95:5) to afford **1g** (0.320 g, 1.00 mmol, quant.) as a colorless oil.

^{1}H NMR (400MHz, $CDCl_3$): δ 7.69 (s, 1H), 7.58 (d, *J* = 7.5 Hz, 1H), 7.46 (d, *J* = 7.7 Hz, 1H), 7.42 (q, *J* = 7.6 Hz, 1H), 2.47 – 2.36 (m, 4H), 1.73 – 1.63 (m, 4H), 0.06 (s, 9H);

^{13}C NMR (101 MHz, $CDCl_3$): δ 140.4, 139.7, 130.6, 130.3 (q, *J* = 31.9 Hz), 128.2, 128.0, 124.38 (q, *J* = 272.5 Hz), 124.31 (q, *J* = 4.3 Hz), 123.4 (q, *J* = 3.6 Hz), 31.3, 30.7, 27.8, 25.9, 0.69 (3C);

^{19}F NMR (376 MHz, $CDCl_3$): δ -62.7 (3F);

IR (neat) ν_{max}: 2957, 2895, 2870, 1436, 1335, 1251, 1164, 1124, 1108, 1071, 868, 839, 804, 753, 701, 624 cm^{-1};

MS (QTOF, EI$^+$, 70 eV): *m/z* 314 [M$^{+\bullet}$] (89), 245 (100), 221 (70), 205 (57), 145 [Ar$^+$] (40) 126 (60), 73 [Me_3Si^+] (81). *HRMS could not be recorded for this compound.*

1h – {Cyclopentylidene[2-(trifluoromethyl)phenyl]methoxy}trimethylsilane

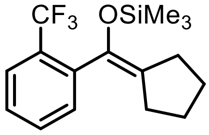

Formula: $C_{16}H_{21}F_3OSi$

MW: 314.4 g/mol

Synthesized following **GP-6**, using **8h** (0.550 g, 2.30 mmol, 1.00 equiv.), Me_3SiOTf (0.630 mL, 3.40 mmol, 1.50 equiv.) and Et_3N (1.40 mL, 9.80 mmol, 4.30 equiv.). The crude product was purified by flash column chromatography on silica gel (heptanes/EtOAc 100:0 to 95:5) to afford **1h** (696 mg, 2.21 mmol, 97%) as a colorless oil.

^{1}H NMR (600 MHz, $CDCl_3$): δ 7.66 (d, *J* = 7.9 Hz, 1H), 7.47 (t, *J* = 7.4 Hz, 1H), 7.38 (t, *J* = 7.5 Hz, 1H), 7.34 (d, *J* = 7.5 Hz, 1H), 2.55 – 2.43 (m, 1H), 2.34 – 2.25 (m, 1H), 2.13 – 2.04 (m, 1H), 1.96 – 1.86 (m, 1H), 1.73 – 1.59 (m, 3H), 1.58 – 1.50 (m, 1H), -0.04 (s, 9H);

^{13}C NMR (151 MHz, $CDCl_3$): δ 136.7, 138.4 (q, *J* = 2.2 Hz), 131.9, 131.4, 128.9 (q, *J* = 30.9 Hz), 127.8, 126.8 (q, *J* = 5.1 Hz), 126.1, 124.2 (q, *J* = 272.9 Hz), 30.4, 29.3, 27.3, 26.4, 0.52 (3C);

^{19}F NMR (565 MHz, $CDCl_3$): δ -60.6 (3F);

IR (neat) ν_{max}: 2956, 2895, 2869, 1684, 1449, 1313, 1282, 1252, 1167, 1132, 1108, 1055, 1035, 955, 914, 885, 841, 767 cm^{-1};

HRMS (ESI$^+$): exact mass calculated for $[M+Na]^+$ ($C_{16}H_{21}F_3OSiNa^+$) requires *m/z* 337.1206, found *m/z* 337.1211.

1i – 4-{Cyclopentylidene[(trimethylsilyl)oxy]methyl}benzonitrile

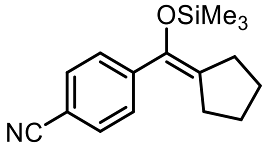

Formula: $C_{16}H_{21}NOSi$

MW: 271.4 g/mol

Synthesized following **GP-6**, using **8i** (0.200 g, 1.00 mmol, 1.00 equiv.), Me_3SiOTf (0.270 mL, 1.50 mmol, 1.50 equiv.) and Et_3N (0.600 mL, 4.30 mmol, 4.30 equiv.). The crude product was purified by flash column chromatography on silica gel (heptanes/EtOAc 100:0 to 90:10) to afford **1i** (0.270 g, 1.00 mmol, quant.) as a colorless oil.

^{1}H NMR (700 MHz, $CDCl_3$): δ 7.60 – 7.57 (m, 2H), 7.51 – 7.49 (m, 2H), 2.46 – 2.42 (m, 2H), 2.41 – 2.36 (m, 2H), 1.71 – 1.65 (m, 4H), 0.06 (s, 9H);

^{13}C NMR (176 MHz, $CDCl_3$): δ 144.2, 139.6, 131.7 (2C), 130.3, 127.9 (2C), 119.2, 110.1, 31.5, 31.1, 27.8, 25.8, 0.73 (3C);

IR (neat) ν_{max}: 2955, 2869, 2226, 1604, 1501, 1435, 1317, 1251, 1166, 1097, 956, 868, 839 cm^{-1};

HRMS (ESI$^+$): exact mass calculated for $[M+Na]^+$ ($C_{16}H_{21}NOSiNa^+$) requires *m/z* 294.1285, found *m/z* 294.1291.

1j – Methyl 4-{cyclopentylidene[(trimethylsilyl)oxy]methyl}benzoate

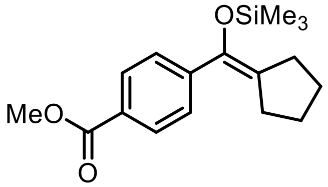

Formula: $C_{17}H_{24}O_3Si$

MW: 304.5 g/mol

Synthesized following **GP-6**, using **8j** (149 mg, 0.640 mmol, 1.00 equiv.), Me_3SiOTf (0.180 mL, 0.960 mmol, 1.50 equiv.) and Et_3N (0.380 mL, 2.80 mmol, 4.30 equiv.). The crude product was purified by flash column chromatography on silica gel (heptanes/EtOAc 100:0 to 95:5) to afford **1j** (176 mg, 0.580 mmol, 90%) as a colorless oil that slowly solidify upon standing at –20 °C.

^{1}H NMR (600 MHz, $CDCl_3$): δ 7.97 (d, J = 8.2 Hz, 2H), 7.47 (d, J = 8.2 Hz, 2H), 3.91 (s, 3H), 2.44 (t, J = 6.5 Hz, 2H), 2.41 (t, J = 6.5 Hz, 2H), 1.71 – 1.63 (m, 4H), 0.05 (s, 9H);

^{13}C NMR (151 MHz, $CDCl_3$): δ 167.1, 144.3, 140.3, 129.2 (2C), 128.9, 128.3, 127.4 (2C), 52.2, 31.4, 30.9, 27.8, 25.9, 0.74 (3C);

IR (neat) ν_{max}: 2953, 2867, 1722, 1606, 1435, 1406, 1313, 1269, 1250, 1165, 1094, 871, 838 cm^{-1};

HRMS (ESI$^+$): exact mass calculated for $[M+Na]^+$ ($C_{17}H_{24}O_3SiNa^+$) requires m/z 327.1387, found m/z 327.1381.

1k – (cyclopentylidene(2,6-dichlorophenyl)methoxy)trimethylsilane

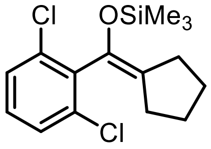

Formula: $C_{15}H_{20}Cl_2OSi$

MW: 315.3 g/mol

Synthesized following **GP-6**, using **8k** (146 mg, 0.600 mmol, 1.00 equiv.), Et_3N (0.840 mL, 6.00 mmol, 10.0 equiv.) and Me_3SiOTf (0.720 mL, 3.90 mmol, 6.50 equiv.). The crude product was purified by flash column chromatography on silica gel (heptanes/EtOAc 100:0 to 96:4) to afford **1k** (166 mg, 0.530 mmol, 88%) as a colorless oil.

^{1}H NMR (400 MHz, $CDCl_3$): δ 7.30 (d, *J* = 8.0 Hz, 2H), 7.14 (dd, *J* = 8.5, 7.6 Hz, 1H), 2.43 (dd, *J* = 10.1, 4.3 Hz, 2H), 1.94 (dd, *J* = 7.6, 6.3 Hz, 2H), 1.73 – 1.66 (m, 2H), 1.66 – 1.57 (m, 2H), 0.03 (s, 9H);

^{13}C NMR (101 MHz, $CDCl_3$): δ 137.2, 135.6 (2C), 135.2, 129.4, 128.2 (2C), 127.8, 29.4, 29.0, 27.2, 26.5, 0.71 (3C);

IR (neat) ν_{max}: 2956, 2891, 2867, 2839, 1687, 1647, 1580, 1556, 1426, 1312, 1298, 1267, 1249, 1191, 1169, 1123, 1087, 1064, 1014, 955, 914, 896, 871, 839, 773 cm^{-1};

HRMS (ESI$^+$): exact mass calculated for $[M+H]^+$ ($C_{15}H_{20}Cl_2OSiH^+$) requires *m/z* 315.0733, found *m/z* 315.0737.

1l – [(4-Bromophenyl)(cyclopentylidene)methoxy]trimethylsilane

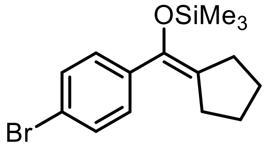

Formula: $C_{15}H_{21}BrOSi$

MW: 325.3 g/mol

Synthesized following **GP-6**, using **8l** (0.510 g, 2.00 mmol, 1.00 equiv.), Me_3SiOTf (0.560 mL, 3.00 mmol, 1.50 equiv.) and Et_3N (1.20 mL, 8.60 mmol, 4.30 equiv.). The crude product was purified by flash column chromatography on silica gel (heptanes/EtOAc 100:0 to 95:5) to afford **1l** (0.490 g, 1.50 mmol, 75%) as a colorless oil.

^{1}H NMR (400 MHz, $CDCl_3$): δ 7.44 – 7.40 (m, 2H), 7.29 – 7.25 (m, 2H), 2.44 – 2.31 (m, 4H), 1.71 – 1.60 (m, 4H), 0.05 (s, 9H);

^{13}C NMR (101 MHz, $CDCl_3$): δ 140.0, 138.7, 130.9 (2C), 129.2 (2C), 127.1, 120.6, 31.3, 30.6, 27.8, 25.9, 0.75 (3C);

IR (neat) ν_{max}: 2954, 2891, 2867, 1587, 1483, 1392, 1311, 1281, 1250, 1164, 1093, 1070, 1008, 870, 837, 751 cm^{-1};

MS (QTOF, EI$^+$, 70 eV): *m/z* 326 [(^{81}Br)$M^{+\bullet}$] (42), 324 [(^{79}Br)$M^{+\bullet}$] (41), 245 [M^+-Br] (89), 185 (24), 183 (24), 155 (20), 128 (16), 73 [Me_3Si^+] (100). *HRMS could not be recorded for this compound.*

1m – [Cyclopentylidene(phenyl)methoxy]trimethylsilane

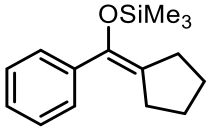

Formula: $C_{15}H_{22}OSi$

MW: 246.4 g/mol

Synthesized following **GP-6**, using **8m** (0.440 g, 2.50 mmol, 1.00 equiv.), Me_3SiOTf (0.700 mL, 3.80 mmol, 1.50 equiv.) and Et_3N (1.50 mL, 11.0 mmol, 4.30 equiv.). The crude product was purified by flash column chromatography on silica gel (heptanes/EtOAc 100:0 to 90:10) to afford **1m** (0.590 g, 2.40 mmol, 95%) as a colorless oil.

Spectral data were in accordance with literature.[67]

^{1}H NMR (400 MHz, CDCl$_3$): δ 7.40 (d, *J* = 7.9 Hz, 2H), 7.29 (t, *J* = 7.6 Hz, 2H), 7.20 (tt, *J* = 7.4, 2.1 Hz, 1H), 2.43 (tt, *J* = 7.5, 1.6 Hz, 2H), 2.38 (tt, *J* = 7.5, 1.6 Hz, 2H), 1.69 – 1.59 (m, 4H), -0.05 (s, 9H).

1n – [Cyclopentylidene(naphthalen-2-yl)methoxy]trimethylsilane

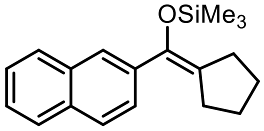

Formula: $C_{19}H_{24}OSi$

MW: 296.5 g/mol

Synthesized following **GP-6**, using **8n** (0.200 g, 0.890 mmol, 1.00 equiv.), Me_3SiOTf (0.250 mL, 1.30 mmol, 1.50 equiv.) and Et_3N (0.540 mL, 3.80 mmol, 4.30 equiv.). The crude product was purified by flash column chromatography on silica gel (heptanes/EtOAc 100:0 to 95:5) to afford **1n** (0.200 g, 0.670 mmol, 75%) as a colorless oil.

^{1}H NMR (400 MHz, $CDCl_3$): δ 7.84 – 7.74 (m, 4H), 7.58 (dd, *J* = 8.6, 1.7 Hz, 1H), 7.48 – 7.41 (m, 2H), 2.51 – 2.44 (m, 4H), 1.75 – 1.62 (m, 4H), 0.06 (s, 9H);

^{13}C NMR (151 MHz, $CDCl_3$): δ 141.0, 137.2, 133.1, 132.5, 128.2, 127.7, 127.2, 126.9, 126.5, 126.1, 126.0, 125.8, 31.4, 30.6, 27.8, 26.0, 0.78 (3C);

IR (neat) ν_{max}: 3056, 2952, 2891, 2865, 2837, 1233, 1090, 964, 870, 838, 819, 745 cm^{-1};

MS (QTOF, EI$^+$, 70 eV): *m/z* 296 [$M^{+\bullet}$] (68), 295 (100), 155 (29), 73 [Me_3Si^+] (32). *HRMS could not be recorded for this compound.*

1o – {Cyclohexylidene[4-(trifluoromethyl)phenyl]methoxy}trimethylsilane

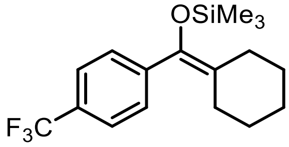

Formula: $C_{17}H_{23}F_3OSi$

MW: 328.5 g/mol

Synthesized following **GP-6**, using **8o** (0.510 g, 2.00 mmol, 1.00 equiv.), Me_3SiOTf (0.840 mL, 4.50 mmol, 2.25 equiv.) and Et_3N (1.80 mL, 13.0 mmol, 6.50 equiv.). The crude product was purified by flash column chromatography on silica gel (heptanes/EtOAc 100:0 to 95:5) to afford **1o** (0.560 g, 1.70 mmol, 85%) as a colorless oil.

^{1}H NMR (400 MHz, CDCl$_3$): δ 7.56 (d, *J* = 8.1 Hz, 2H), 7.41 (d, *J* = 8.0 Hz, 2H), 2.34 (t, *J* = 5.8 Hz, 2H), 2.20 – 2.07 (m, 2H), 1.68 – 1.55 (m, 4H), 1.49 (d, *J* = 5.7 Hz, 2H), -0.03 (s, 9H);

^{13}C NMR (101 MHz, CDCl$_3$): δ 142.7, 140.0, 129.6 (2C), 127.8, 126.11 (q, *J* = 3.7 Hz), 124.9 (q, *J* = 3.8 Hz, 2C), 122.9, 29.9, 28.2, 28.0, 27.5, 26.9, 0.5 (3C);

^{19}F NMR (376 MHz, CDCl$_3$): δ -62.4 (3F);

IR (neat) ν_{max}: 2930, 2856, 1728, 1688, 1581, 1511, 1451, 1409, 1323, 1252, 1207, 1168, 1129, 1066, 1018, 975, 946, 913, 879, 842, 765 cm^{-1};

HRMS (ESI$^+$): exact mass calculated for $[M+H]^+$ ($C_{17}H_{24}F_3OSi^+$) requires *m/z* 329.1543, found *m/z* 329.1543.

1p – [Benzo[*b*]thiophen-2-yl(cyclohexylidene)methoxy]trimethylsilane

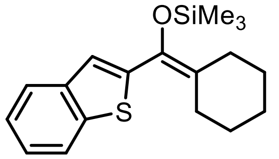

Formula: $C_{18}H_{24}OSSi$

MW: 316.5 g/mol

Synthesized following **GP-6**, using **8p** (122 mg, 0.500 mmol, 1.00 equiv.), Et_3N (0.700 mL, 5.00 mmol, 10.0 equiv.) and Me_3SiOTf (0.600 mL, 3.30 mmol, 6.50 equiv.). The crude product was purified by flash column chromatography on silica gel (heptanes/EtOAc 100:0 to 98:2) to afford **1p** (152 mg, 0.540 mmol, 96%) as a colorless oil.

^{1}H NMR (400 MHz, CDCl$_3$): δ 7.83 – 7.79 (m, 1H), 7.74 (dd, *J* = 7.0, 1.5 Hz, 1H), 7.38 – 7.27 (m, 2H), 7.16 (s, 1H), 2.47 – 2.33 (m, 4H), 1.59 (m, 6H), 0.11 (s, 9H);

^{13}C NMR (101 MHz, CDCl$_3$): δ 142.0, 139.9, 139.5, 135.1, 125.5, 124.3, 124.2, 123.6, 123.0, 122.3, 30.3, 28.4, 28.2, 27.5, 26.8, 0.5 (3C);

IR (neat) ν_{max}: 3058, 2925, 2852, 1659, 1528, 1457, 1447, 1436, 1348, 1335, 1304, 1249, 1195, 1177, 1155, 1120, 1073, 1049, 1024, 1015, 976, 929, 906, 865, 841, 786 cm^{-1};

HRMS (ESI$^+$): exact mass calculated for $[M+H]^+$ ($C_{18}H_{24}OSSiH^+$) requires *m*/*z* 317.1390, found *m*/*z* 317.1388.

1q – {[3-(*tert*-Butyl)cyclopentylidene][4-(trifluoromethyl)phenyl]methoxy}trimethylsilane

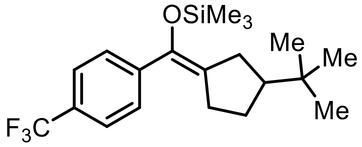

Formula: $C_{20}H_{29}F_3OSi$

MW: 370.5 g/mol

Synthesized following **GP-6**, using **8q** (0.300 g, 1.00 mmol, 1.00 equiv.), Me_3SiOTf (1.20 mL, 6.50 mmol, 6.50 equiv.) and Et_3N (1.40 mL, 10.0 mmol, 10.0 equiv.). The crude product was purified by flash column chromatography on silica gel (heptanes/EtOAc 100:0 to 98:2) to afford **1q** (0.290 g, 0.790 mmol, 79%, *E*/*Z* = 1:1) as an off-white solid.

^{1}H NMR (600 MHz, CDCl$_3$): δ 7.58 – 7.53 (m, 2H), 7.52 – 7.47 (m, 2H), 2.57 – 2.32 (m, 3H), 2.16 (m, 1H), 1.81 – 1.67 (m, 2H), 1.34 (m, 1H), 0.91 (s, 4.5H), 0.87 (s, 4.5H), 0.07 (s, 4.5H), 0.06 (s, 4.5H);

^{13}C NMR (151 MHz, CDCl$_3$): δ [143.2 and 143.1], 139.9, 128.6* (q, *J* = 32.2 Hz), [128.4 and 128.3], [127.7 and 127.6] (2C), [124.81 and 124.77] (q, *J* = 4.3 Hz, 2C), 124.4 (q, *J* = 272.5 Hz), [52.7 and 50.8], [32.6 and 30.6], [32.3 and 31.3], [31.80 and 31.79], [28.6 and 26.6], [27.89 and 27.86] (3C), [0.77 and 0.76] (3C); **appears as qd because of the two stereoisomers.*

^{19}F NMR (565 MHz, CDCl$_3$): δ [-62.38 and -62.39] (3F);

IR (neat) ν_{max}: 2960, 2869, 1407, 1364, 1320, 1252, 1162, 1123, 1098, 1065, 1015, 870, 840 cm^{-1};

MS (QTOF, EI$^+$, 70 eV): *m/z* 370 [$M^{+\bullet}$] (76), 313 [M^+-CMe$_3$] (68), 301 (71), 273 (32), 221 (55), 173 (40), 153 (23), 73 [Me_3Si^+] (100). *HRMS could not be recorded for this compound.*

1r – Trimethyl{(3-methylcyclopentylidene)[4-(trifluoromethyl)phenyl]methoxy}silane

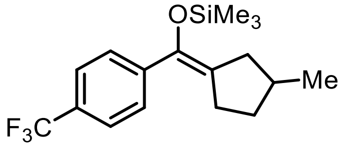

Formula: $C_{17}H_{23}F_3OSi$

MW: 328.5 g/mol

Synthesized following **GP-6**, using **8r** (0.510 g, 2.00 mmol, 1.00 equiv.), Me_3SiOTf (0.550 mL, 3.00 mmol, 1.50 equiv.) and Et_3N (1.20 mL, 8.60 mmol, 4.30 equiv.). The crude product was purified by flash column chromatography on silica gel (heptanes/EtOAc 100:0 to 90:10) to afford **1r** (0.590 g, 1.80 mmol, 90%, *E/Z* = 1:1) as a colorless oil.

^{1}H NMR (400 MHz, CDCl$_3$): δ 7.58 – 7.47 (m, 4H), 2.70 – 2.59 (m, 0.5H), 2.58 – 2.34 (m, 2.5H), 2.11 – 1.93 (m, 2H), 1.92 – 1.78 (m, 1H), 1.36 – 1.18 (m, 1H), 1.05 (d, *J* = 6.4 Hz, 1.5H), 0.99 (d, *J* = 6.3 Hz, 1.5H), 0.06 (s, 9H);

^{13}C NMR (151 MHz, CDCl$_3$): δ [143.18 and 143.09], [139.97 and 139.87], 128.6* (q, *J* = 21.3 Hz), [128.55 and 128.50], [127.69 and 127.66] (2C), 124.8 (q, *J* = 4.1 Hz, 2C), 124.4 (q, *J* = 271.5 Hz), [40.0 and 39.3], [36.0 and 35.8], [34.1 and 34.0], [30.9 and 30.4], [20.1 and 19.8], 0.75 (3C); **appears as a qd because of the E/Z mixture.*

^{19}F NMR (376 MHz, CDCl$_3$): δ -62.4 (3F);

IR (neat) ν_{max}: 2954, 1615, 1407, 1323, 1252, 1163, 1124, 1099, 1066, 1016 ,871, 842, 749 cm^{-1};

MS (QTOF, EI$^+$, 70 eV): *m/z* 328 [$M^{+\bullet}$] (80), 313 [M^+-Me] (63), 259 [M^+-CF_3] (100), 217 (39), 173 (28), 73 [Me_3Si^+] (82). *HRMS could not be recorded for this compound.*

1s – Trimethyl[(3-methylcyclopentylidene)(phenyl)methoxy]silane

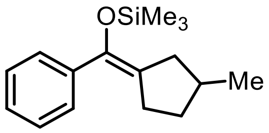

Formula: $C_{16}H_{24}OSi$

MW: 260.5 g/mol

Synthesized following **GP-6**, using **8s** (0.420 g, 2.20 mmol, 1.00 equiv.), Me_3SiOTf (0.610 mL, 3.30 mmol, 1.50 equiv.) and Et_3N (1.30 mL, 9.60 mmol, 4.30 equiv.). The crude product was purified by flash column chromatography on silica gel (heptanes/EtOAc 100:0 to 95:5) to afford **1s** (0.380 g, 1.50 mmol, 49%, *E*/*Z* = 1:1) as a colorless oil.

^{1}H NMR (400 MHz, $CDCl_3$): δ 7.38 (dd, *J* = 7.4, 1.9 Hz, 2H), 7.29 (td, *J* = 7.3, 0.8 Hz, 2H), 7.19 (td, *J* = 7.3, 1.6 Hz, 1H), 2.69 – 2.59 (m, 0.5H), 2.57 – 2.32 (m, 2.5H), 2.09 – 1.89 (m, 2H), 1.89 – 1.75 (m, 1H), 1.28 – 1.12 (m, 1H), 1.03 (d, *J* = 6.5 Hz, 1.5H), 0.97 (d, *J* = 6.2 Hz, 1.5H), 0.04 (s, 9H);

^{13}C NMR (151 MHz, $CDCl_3$): δ [141.0 and 140.9], [139.6 and 139.5], 127.73 (2C), [127.69 and 127.67] (2 C), [126.85 and 126.83], [125.97 and 125.92], [39.9 and 39.1], [35.9 and 35.8], [34.15 and 34.11], [30.8 and 30.0], [20.2 and 19.9], 0.73 (3C);

IR (neat) ν_{max}: 2954, 2867, 2838, 1660, 1600, 1492, 1443, 1315, 1270, 1250, 1165, 1099, 956, 871, 839, 754, 698 cm^{-1};

HRMS (ESI$^+$): exact mass calculated for $[M+H]^+$ ($C_{16}H_{25}SiO^+$) requires *m*/*z* 261.1669, found *m*/*z* 261.1656.

1t – {(4-Butylcyclohexylidene)[4-(trifluoromethyl)phenyl]methoxy}trimethylsilane

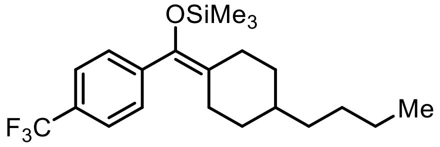

Formula: $C_{21}H_{31}F_3OSi$

MW: 384.6 g/mol

Synthesized following **GP-6**, using **8t** (0.310 g, 1.00 mmol, 1.00 equiv.), Me_3SiOTf (1.20 mL, 6.50 mmol, 6.50 equiv.) and Et_3N (1.40 mL, 10.0 mmol, 10.0 equiv.). The crude product was purified by flash column chromatography on silica gel (heptanes/EtOAc 100:0 to 95:5) to afford **1t** (0.320 g, 0.820 mmol, 82%) as a colorless oil.

^{1}H NMR (400 MHz, $CDCl_3$): δ 7.56 (d, *J* = 8.1 Hz, 2H), 7.41 (d, *J* = 8.1 Hz, 2H), 2.96 – 2.86 (m, 1H), 2.45 – 2.36 (m, 1H), 1.92 – 1.70 (m, 4H), 1.46 – 1.35 (m, 1H), 1.33 – 1.17 (m, 6H), 1.08 – 0.90 (m, 2H), 0.89 (t, *J* = 7.1 Hz, 3H), -0.03 (s, 9H);

^{13}C NMR (101 MHz, $CDCl_3$): δ 142.8, 139.9, 129.5 (2C), 129.3 (q, *J* = 32.1 Hz), 124.8 (q, *J* = 4.0 Hz, 2C), 124.3 (q, *J* = 271.8 Hz), 122.9, 37.8, 36.6, 34.5, 33.8, 29.4, 29.3, 27.4, 23.1, 14.3, 0.50 (3C);

^{19}F NMR (376 MHz, $CDCl_3$): δ -62.4 (3F);

IR (neat) ν_{max}: 2958, 2918, 2850, 1406, 1322, 1252, 1164, 1125, 1091, 1064, 1017, 865, 840 cm^{-1};

MS (QTOF, EI^+, 70 eV): *m/z* 384 [$M^{+\bullet}$] (93), 315 (73), 273 (37), 247 (60), 237 (55), 191 (65), 173 (40), 135 (42), 73 [Me_3Si^+] (100). *HRMS could not be recorded for this compound.*

1u – {[4-(*tert*-Butyl)cyclohexylidene][4-(trifluoromethyl)phenyl]methoxy}trimethylsilane

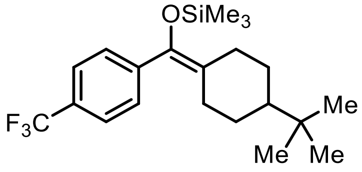

Formula: $C_{21}H_{31}F_3OSi$

MW: 384.6 g/mol

Synthesized following **GP-6**, using **8u** (0.780 g, 2.50 mmol, 1.00 equiv.), Me_3SiOTf (3.00 mL, 16.3 mmol, 6.50 equiv.) and Et_3N (3.50 mL, 25.0 mmol, 10.0 equiv.). The crude product was purified by flash column chromatography on silica gel (heptanes/EtOAc 100:0 to 95:5) to afford **1u** (0.620 g, 1.60 mmol, 64%) as a colorless solid.

^{1}H NMR (600 MHz, $CDCl_3$): δ 7.56 (d, *J* = 8.0 Hz, 2H), 7.42 (d, *J* = 8.0 Hz, 2H), 3.01 (dq, *J* = 13.9, 3.0 Hz, 1H), 2.48 (dq, *J* = 13.8, 2.6 Hz, 1H), 1.93 – 1.89 (m, 1H), 1.86 (td, *J* = 13.1, 3.8 Hz, 1H), 1.80 – 1.71 (m, 2H), 1.17 (tt, *J* = 11.7, 2.7 Hz, 1H), 1.09 (qd, *J* = 12.5, 3.7 Hz, 1H), 0.97 (qd, *J* = 12.5, 3.6 Hz, 1H), 0.85 (s, 9H), -0.02 (s, 9H);

^{13}C NMR (151 MHz, $CDCl_3$): δ 142.7, 139.7, 129.5 (2C), 129.2 (q, *J* = 32.4 Hz), 124.8 (q, *J* = 4.1 Hz, 2C), 123.9 (q, *J* = 271.9 Hz), 122.9, 48.4, 32.6, 29.9, 28.9, 28.1, 28.0, 27.7 (3C), 0.51 (3C);

^{19}F NMR (565 MHz, $CDCl_3$): δ -62.4 (3F);

IR (neat) ν_{max}: 2961, 2938, 2917, 2857, 1316, 1249, 1166, 1129, 1115, 1103, 1075, 1060, 863, 844 cm^{-1};

MS (QTOF, EI^+, 70 eV): *m/z* 384 [$M^{+\bullet}$] (97), 327 [M^+-CMe_3] (30), 315 [M^+-CF_3] (48), 237 (37), 191 (56), 173 (34), 73 [Me_3Si^+] (100). *HRMS could not be recorded for this compound.*

1v – {[4-(*tert*-Butyl)cyclohexylidene][3-(trifluoromethyl)phenyl]methoxy}trimethylsilane

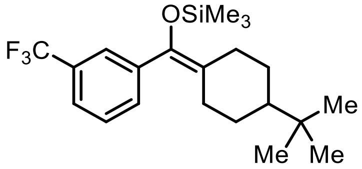

Formula: $C_{21}H_{31}F_3OSi$

MW: 384.6 g/mol

Synthesized following **GP-6**, using **8v** (0.310 g, 1.00 mmol, 1.00 equiv.), Me_3SiOTf (1.20 mL, 6.50 mmol, 6.50 equiv.) and Et_3N (1.40 mL, 10.0 mmol, 10.0 equiv.). The crude product was purified by flash column chromatography on silica gel (heptanes/EtOAc 100:0 to 95:5) to afford **1v** (0.260 g, 0.670 mmol, 67%) as a colorless oil.

^{1}H NMR (400 MHz, $CDCl_3$): δ 7.58 (s, 1H), 7.51 – 7.45 (m, 2H), 7.45 – 7.39 (m, 1H), 3.01 (dq, *J* = 13.6, 2.8 Hz, 1H), 2.47 (dq, *J* = 13.6, 2.5 Hz, 1H), 1.94 – 1.82 (m, 2H), 1.81 – 1.69 (m, 2H), 1.21 – 0.91 (m, 3H), 0.85 (s, 9H), -0.03 (s, 9H);

^{13}C NMR (101 MHz, $CDCl_3$): δ 139.8, 139.6, 132.5, 130.4 (q, *J* = 32.1 Hz), 128.3, 126.1 (q, *J* = 3.6 Hz), 124.3 (q, *J* = 272.8 Hz), 123.9 (q, *J* = 3.7 Hz), 122.5, 48.4, 32.6, 29.9, 28.9, 28.1, 28.0, 27.7 (3C), 0.46 (3C);

^{19}F NMR (376 MHz, $CDCl_3$): δ -62.7 (3F);

IR (neat) ν_{max}: 2957, 2868, 2840, 1365, 1334, 1264, 1252, 1164, 1125, 1070, 864, 840 cm^{-1};

MS (QTOF, EI$^+$, 70 eV): *m/z* 384 [$M^{+\bullet}$] (100), 327 [M^+-C(Me_3)] (26), 279 (22), 235 (23), 191 (30), 181 (20), 155 (29), 73 [Me_3Si^+] (75), 57 [Me_3C^+] (15). *HRMS could not be recorded for this compound.*

1w – Trimethyl{(4-methylcyclohexylidene)[4-(trifluoromethyl)phenyl]methoxy}silane

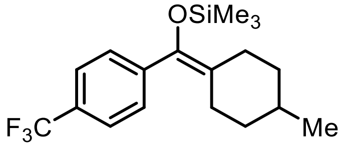

Formula: $C_{18}H_{25}F_3OSi$

MW: 342.5 g/mol

Synthesized following **GP-6**, using **8w** (0.410 g, 1.50 mmol, 1.00 equiv.), Me_3SiOTf (1.80 mL, 9.75 mmol, 6.50 equiv.) and Et_3N (2.10 mL, 15.0 mmol, 10.0 equiv.). The crude product was purified by flash column chromatography on silica gel (heptanes/EtOAc 100:0 to 95:5) to afford **1w** (0.330 g, 0.960 mmol, 64%) as a colorless solid.

^{1}H NMR (400 MHz, $CDCl_3$): δ 7.56 (d, J = 8.1 Hz, 2H), 7.41 (d, J = 8.1 Hz, 2H), 2.95 – 2.85 (m, 1H), 2.43 – 2.35 (m, 1H), 1.88 (td, J = 13.3, 4.2 Hz, 1H), 1.85 – 1.76 (m, 2H), 1.75 – 1.64 (m, 1H), 1.61 – 1.47 (m, 1H), 1.10 – 0.92 (m, 2H), 0.91 (d, J = 6.6 Hz, 3H), -0.03 (s, 9H);

^{13}C NMR (101 MHz, $CDCl_3$): δ 142.8, 140.1, 129.5 (2C), 129.3 (q, J = 32.1 Hz), 124.8 (q, J = 4.0 Hz, 2C), 124.3 (q, J = 271.8 Hz), 122.5, 36.4, 35.7, 32.8, 29.3, 27.4, 22.2, 0.50 (3C);

^{19}F NMR (376 MHz, $CDCl_3$): δ -62.4 (3F);

IR (neat) ν_{max}: 2954, 2913, 2846, 1455, 1405, 1321, 1252, 1222, 1163, 1124, 1084, 1064, 909, 863, 839, 749 cm^{-1};

MS (QTOF, EI$^+$, 70 eV): m/z 342 [$M^{+\bullet}$] (100), 273 [M^+-CF_3] (89), 247 (23), 191 (44), 183 (35), 73 [Me_3Si^+] (93). *HRMS could not be recorded for this compound.*

4 Synthesis of the iodonium reagents 9a-9d

General procedure GP-7

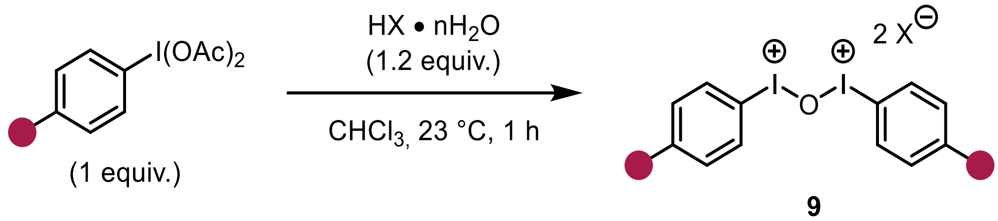

Adapted from a literature procedure.[68]

To a cooled (0 °C) solution of thecorresponding diacetoxyiodoarene (1.00 equiv.) in chloroform ($CHCl_3$, 2 M) the acid (1.20 equiv.) was slowly added, and the resulting mixture was allowed to warm to 23 °C. After 1 h, water (1.00 mL for 1.00 mmol of starting diacetoxyiodoarene) was added, and the mixture was cooled to 0 °C. After 3 h at 0 °C, a precipitate had formed and the mixture was filtered. The precipitate was washed with $CHCl_3$, H_2O and pentane. The obtained solid was crushed to a powder and the washing steps were repeated in the same order twice. The resulting solid was dried under high vacuum (10^{-2}–10^{-3} mbar) for at least 5 h to afford the dicationic iodonium reagent as a bright-yellow powder.

Troubleshooting:

- This reaction should not be performed on electron-rich (*e.g.* *para*-OMe) diacetoxyiodoarenes, as they can decompose violently upon addition of strong acid.
- The acids were used as solutions in water or as water adducts: $HSbF_6{\cdot}6H_2O$, HBF_4 (48 w% in H_2O), $HClO_4$ (70 w% in H_2O).
- $HSbF_6{\cdot}6H_2O$ was weighed.
- Crushing the precipitate to a fine powder is important, as it allows more efficient washing.
- Iodonium reagents can be analyzed by NMR using 2:8 mixture of DMSO-d_6/$CDCl_3$. Spectra have to be taken rapidly, as the reagents slowly decompose in this solvent mixture.
- Once dried, the iodonium reagents can be kept for months in a freezer (–20°C) with no sign of degradation.

9a – $(PhI)_2O(SbF_6)_2$ – Oxybis(phenyliodonium) bis[hexafluorostilbate(V)]

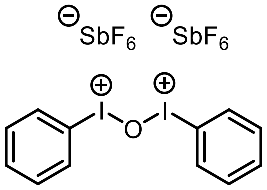

Formula: $C_{12}H_{10}F_{12}I_2OSb_2$

MW: 895.5 g/mol

Following general procedure **GP-7** on a 50.0 mmol scale, diacetoxyiodobenzene (16.4 g, 50.0 mmol, 1.00 equiv.) and $HSbF_6 \cdot 6H_2O$ (22.0 g, 61.0 mmol, 1.20 equiv.) afforded **9a** (12.7 g, 14.2 mmol, 57%) as a bright-yellow powder.

Spectral data were in accordance with the literature.[68] *Carbon spectrum could not be recorded, as product decomposes readily in the presence of DMSO.*

^{1}H NMR (400 MHz, 2:8 DMSO-d_6/$CDCl_3$): δ 7.74 – 7.53 (m, 4H), 7.47 – 7.35 (m, 2H), 7.32 – 7.14 (m, 4H);

^{19}F NMR (376 MHz, 2:8 DMSO-d_6/$CDCl_3$): δ -107.5 – -137.5 (m, 12F);

IR (neat) ν_{max}: 1470, 1443, 992, 746, 658 cm^{-1};

HRMS (ESI$^+$): exact mass calculated for $[M - 2SbF_6^- + H_2O]^{2+}$ ($C_{12}H_{12}I_2O_2^{2+}$) requires *m/z* 220.9458, found *m/z* 220.9454.

HRMS (ESI$^-$): exact mass calculated for SbF_6^- requires *m/z* 234.8947 and 236.8951, found *m/z* 234.8942 and 236.8946.

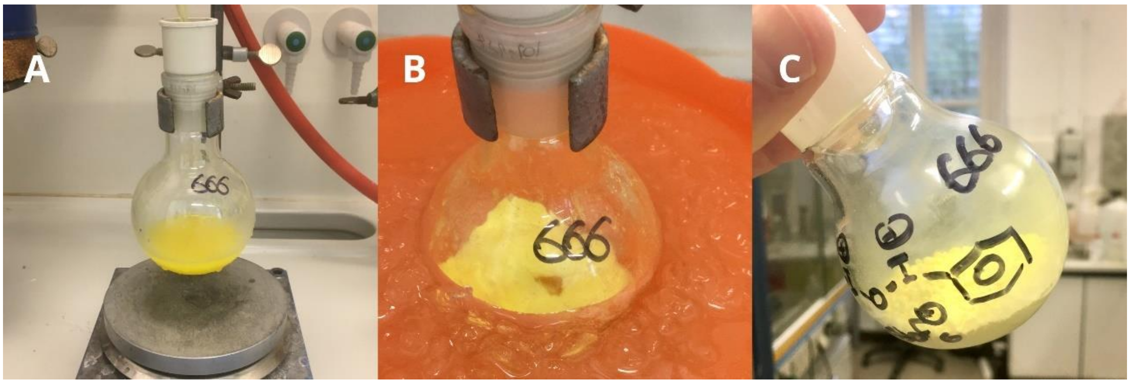

Figure S3. Synthesis of iodonium reagent (9a) on 50 mmol scale. A. Reaction mixture after addition of $HSbF_6 \bullet 6H_2O$. **B.** Reaction mixture during precipitation, 1 h after addition of water and cooling to 0 °C. **C.** Final product (in a 100 mL round-bottom flask) after washing and drying (12.7 g).

9b – $(PhI)_2O(BF_4)_2$ – Oxybis(phenyliodonium) bis[tetrafluoroborate(V)]

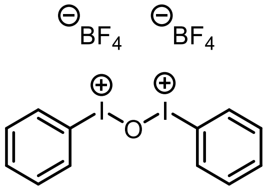

Formula: $C_{12}H_{10}F_8I_2OB_2$

MW: 597.63 g/mol

Following general procedure **GP-7** on a 10.0 mmol scale, diacetoxyiodobenzene (3.3 g, 10.0 mmol, 1.00 equiv.) and HBF_4 (2.09 mL, 16.0 mmol, 1.60 equiv. 48 w% in H_2O) afforded **9b** (845 mg, 1.41 mmol, 28%) as a bright-yellow powder.

Spectral data were in accordance with the literature.[68] *Carbon spectrum could not be recorded as product decomposes readily in the presence of DMSO.*

^{1}H NMR (400 MHz, 2:8 DMSO-d_6/$CDCl_3$): δ 7.76 – 7.55 (m, 4H), 7.44 – 7.33 (m, 2H), 7.32 – 7.12 (m, 4H);

^{19}F NMR (376 MHz, 2:8 DMSO-d_6/$CDCl_3$): δ -146.03 (m, 4F).

HRMS could not be recorded for this compound.

9c – $(PhI)_2O(ClO_4)_2$ – Oxybis(phenyliodonium) bis(perchlorate)

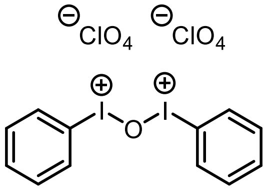

Formula: $C_{12}H_{10}Cl_2I_2O_9$

MW: 622.9 g/mol

Following general procedure **GP-7** on a 10.0 mmol scale, diacetoxyiodobenzene (3.30 g, 10.0 mmol, 1.00 equiv.) and perchloric acid (1.60 mL, 12.0 mmol, 1.20 equiv, 70 w% in H_2O) afforded **9c** (1.71 g, 2.75 mmol, 55%) as a yellow solid.

Carbon spectrum could not be recorded as product decomposes readily in the presence of DMSO.

^{1}H NMR (400 MHz, 2:8 DMSO-d_6/$CDCl_3$): δ 7.73 – 4.56 (m, 4H), 7.45 – 7.35 (m, 2 H), 7.30 – 7.16 (m, 4H);

IR (neat) ν_{max}: 3054, 1470, 1442, 1095, 1057, 991, 925, 729, 677, 620, 570, 412 cm^{-1};

HRMS (ESI$^+$): exact mass calculated for $[M - 2ClO_4^- + H_2O]^{2+}$ ($C_{12}H_{12}I_2O_2^{2+}$) requires m/z 220.9458, found m/z 220.9460;

HRMS (ESI$^-$): exact mass calculated for ClO_4^- requires m/z 98.9491, found m/z 98.9487; exact mass calculated for $[M+ClO_4]^-$ ($C_{12}H_{10}Cl_3I_2O_{13}^-$) requires m/z 720.7281, found m/z 720.7296.

9d – (*para*-*t*-BuC$_6$H$_4$I)$_2$O(SbF$_6$)$_2$

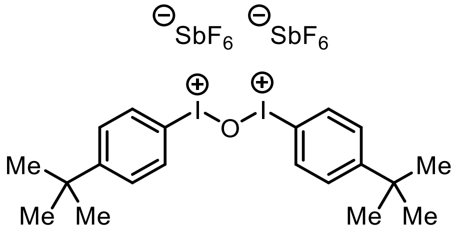

Formula: $C_{20}H_{26}F_{12}I_2OSb_2$

MW: 1007.7 g/mol

Following general procedure **GP-7** on a 6.00 mmol scale, [4-(*tert*-butyl)phenyl]-λ^3-iodanediyl diacetate [69-70] (2.30 g, 6.00 mmol, 1.00 equiv.) and $HSbF_6 \cdot 6H_2O$ (2.50 g, 7.20 mmol, 1.20 equiv.) afforded **9d** (1.88 g, 1.87 mmol, 62%) as a bright-yellow powder.

Carbon spectrum could not be recorded as product decomposes readily in the presence of DMSO.

^{1}H NMR (400 MHz, 2:8 DMSO-*d$_6$*/CDCl$_3$): δ 7.75 – 7.52 (m, 4 H), 7.39 – 7.24 (m, 4 H), 1.26 (s, 18 H);

^{19}F NMR (565 MHz, 2:8 DMSO-*d$_6$*/CDCl$_3$): δ = -110.1 – -132.4 (m, 12 F);

IR (neat) ν_{max}: 2965, 1637, 1576, 1482, 1395, 1366, 1292, 1108, 999, 833, 820, 656, 542 cm^{-1};

HRMS (ESI$^+$): exact mass calculated for $[M - 2SbF_6^- + H_2O]^{2+}$ ($C_{20}H_{28}I_2O_2^{2+}$) requires *m/z* 277.0084, found *m/z* 277.0070;

HRMS (ESI$^-$): exact mass calculated for SbF_6^- requires *m/z* 234.8947 and 236.8951, found *m/z* 234.8942 and 236.8945.

5 Oxidation products 2a-2c' and 2f-2w

General procedure GP-8

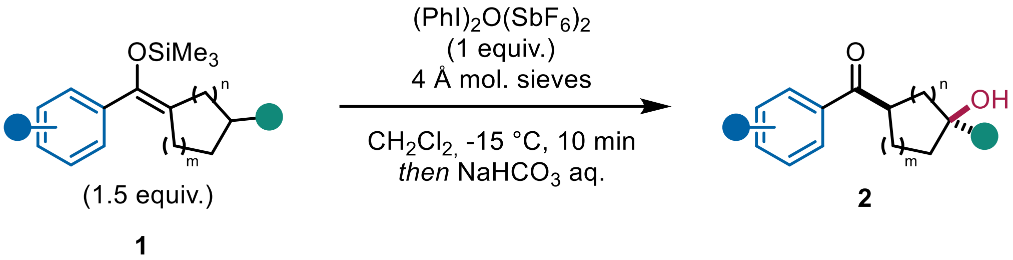

A Schlenk flask was charged with 4 Å molecular sieves (4-5 beads for 0.1 mmol of silyl enol ether), evacuated under high vacuum (*ca* 10^{-2} bar), flame-dried and back-filled with argon. After cooling to rt, the flask was further cooled to -15 °C (using a NaCl/ice mixture), and $(PhI)_2O(SbF_6)_2$ (1 equiv.) was added, followed by CH_2Cl_2 (0.1 M). The resulting mixture was vigorously stirred for 5 min, after which a solution of silyl enol ether in CH_2Cl_2 (0.15 M, 1.5 equiv.) was added dropwise, and the mixture was left stirring at -15 °C for 10 min. A sat. aq. solution of $NaHCO_3$ was then added (*ca* 2 mL for 0.1 mmol of iodonium reagent), and the mixture was warmed to 23 °C and vigorously stirred for 10 min. The biphasic mixture was transferred to a separatory funnel with a Pasteur pipette, in order to remove the molecular sieves, and the flask was rinsed with a small amount of CH_2Cl_2. The phases were separated and the aqueous phase was extracted with CH_2Cl_2 (3 x 5 mL). The combined organic phases were washed with sat. aq. NaCl, dried over $MgSO_4$, filtered and concentrated under reduced pressure. The residue was purified by flash column chromatography on silica gel (typical eluent: heptanes/EtOAc, 90:10 to 60:40) to afford the pure product of remote oxidation.

Troubleshooting:

- We experienced that vigorous stirring is crucial for the performance of the reaction, especially since the iodonium reagent is poorly soluble in CH_2Cl_2. An experiment done without stirring only led to the formation of the ketone resulting from silyl enol ether hydrolysis.
- The cooling bath used was a NaCl/ice mixture. -15 °C is the average temperature measured but this can vary slightly, with no consequences on the reaction outcome.
- In some cases, a clear yellow to orange solution is obtained before treatment with aqueous $NaHCO_3$. In some other cases, a yellow suspension is obtained. This does not prefigure the outcome of the reaction.
- On larger scale, it is possible to filter the reaction mixture before extraction to avoid crushed molecular sieves hindering phase separation.
- The silyl enol ether solution is added dropwise (typically over 30 s), mainly to avoid the resulting exotherm from elevating the reaction temperature significantly above -15 °C.
- PMA (phosphomolybdic acid) stain is recommended for product visualization.
- The iodonium reagent can be kept for months in a freezer at –20 °C under argon (>6 months). Degradation of the reagent can easily be assessed visually, as it loses its typical bright-yellow color.

2a – *cis*-3-Hydroxy-3-methylcyclohexyl][4-(trifluoromethyl)phenyl]methanone

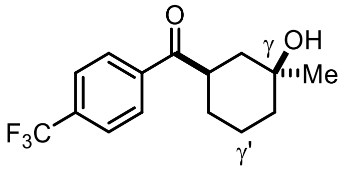

γ/γ' = 83:17

Formula: $C_{15}H_{17}F_3O_2$

MW: 286.9 g/mol

Synthesized following **GP-8**, using **1a** (51.4 mg, 0.150 mmol, 1.50 equiv.) and $(PhI)_2O(SbF_6)_2$ (89.6 mg, 0.100 mmol, 1.00 equiv.). Purification by flash column chromatography on silica gel (heptanes/EtOAc, 95:5 to 50:50) afforded the title compound (21.0 mg, 73.4 µmol, 73%, γ/γ' = 83:17) as a colorless oil.

The same procedure using **(–)-(*R*)-1a** yielded **(+)-2a** in 75% *ee*.

A single crystal, suitable for X-ray analysis, was obtained by slow evaporation, at rt, of a diethyl ether solution of the compound, which had been slowly covered by heptanes so as to ensure that the mixture remains biphasic. The x-ray crystallographic details can be found in section 8.

Spectral data are reported for the major regioisomer.

^{1}H NMR (700 MHz, $CDCl_3$): δ 8.01 (d, *J* = 8.2 Hz, 2H), 7.73 (d, *J* = 8.2 Hz, 2H), 3.49 – 3.45 (m, 1H), 2.32 (br s, 1H, *O–H*), 1.87 – 1.79 (m, 3H), 1.77 (dd, *J* = 13.1, 9.5 Hz, 1H), 1.72 – 1.47 (m, 4H), 1.33 (s, 3H);

^{13}C NMR (176 MHz, $CDCl_3$): δ 202.6, 139.1, 134.5 (q, *J* = 32.7 Hz), 128.8 (2C), 125.9 (q, *J* = 3.9 Hz, 2C), 123.7 (q, *J* = 272.4 Hz), 70.1, 43.4, 41.6, 39.9, 28.3, 27.4, 21.9;

^{19}F NMR (659 MHz, $CDCl_3$): δ -63.1 (3F);

IR (neat) ν_{max}: 3407 (br), 2934, 2863, 1684, 1408, 1322, 1165, 1111, 1065, 995, 851 cm^{-1};

HRMS (ESI$^+$): exact mass calculated for $[M+Na]^+$ ($C_{15}H_{17}F_3O_2Na^+$) requires *m/z* 309.1073, found *m/z* 309.1068;

$[\alpha]_D^{20}$ +7.82 (c = 0.46, $CHCl_3$, 75% *ee*, γ/γ' = 83:17). Enantiomeric excess 75% for γ-alcohol was determined by chiral HPLC analysis: Chiralpak IC 250x4.6 mm, Heptanes+0.1%*i*-PrOH/*i*-PrOH (9:1), 1 mL/min, 25 °C, detection at 230 nm, retention time (min): 12.0 (major), 14.5 (minor).

(±)-2a – *cis*-3-Hydroxy-3-methylcyclohexyl][4-(trifluoromethyl)phenyl]methanone

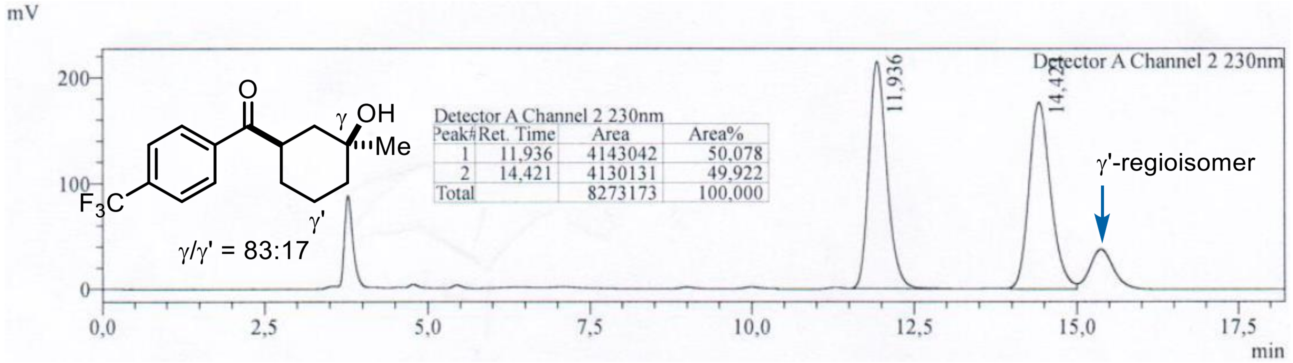

(+)-2a – [(1*R*,3*S*)-3-Hydroxy-3-methylcyclohexyl][4-(trifluoromethyl)phenyl]methanone

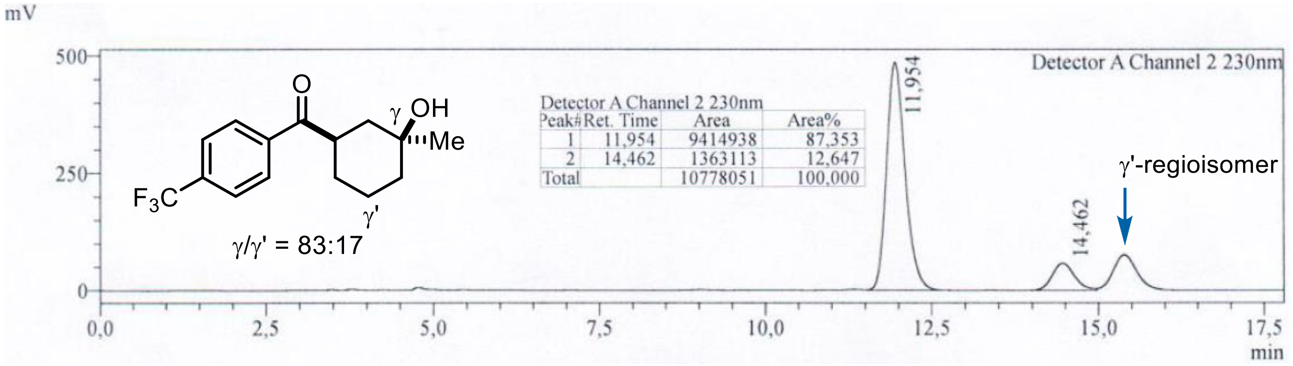

2b – ((1*R*,3*R*,8*S*,9*S*,10*S*,13*R*,14*S*,17*R*)-10-hydroxy-1,13-dimethyl-17-((*R*)-6-methylheptan-2-yl)hexadecahydro-1*H*-cyclopenta[a]phenanthren-3-yl)(4-(trifluoromethyl)phenyl)methanone

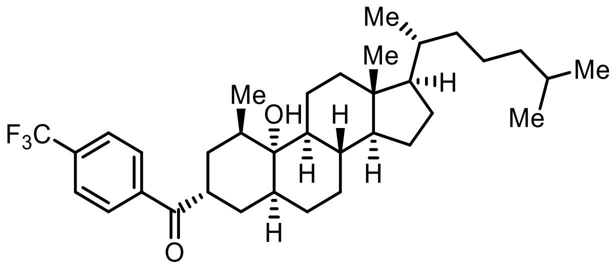

Formula: $C_{35}H_{51}F_3O_2$

MW: 560.8 g/mol

Synthesized following **GP-8**, using **1b** (61.7 mg, 0.100 mmol, 1.50 equiv.) and $(PhI)_2O(SbF_6)_2$ (59.7 mg, 66.7 µmol, 1.00 equiv.). Purification by flash column chromatography on silica gel (heptanes/EtOAc, 95:5 to 50:50) afforded the title compound (24.3 mg, 39.2 µmol, 65%) as a colorless solid.

^{1}H NMR (700 MHz, $CDCl_3$): δ 8.03 (d, *J* = 8.1 Hz, 2H), 7.74 (d, *J* = 8.2 Hz, 2H), 3.62 (tt, *J* = 12.6, 2.6 Hz, 1H), 2.29 (td, *J* = 13.3, 5.7 Hz, 1H), 2.23 (td, *J* = 13.1, 5.0 Hz, 1H), 2.08 (tt, *J* = 6.6, 4.6 Hz, 2H), 1.93 – 1.76 (m, 4H), 1.69 – 1.46 (m, 8H), 1.39 (d, *J* = 7.6 Hz, 3H), 1.37 – 0.95 (m, 15H), 0.92 (d, *J* = 6.5 Hz, 3H), 0.87 (d, *J* = 3.3 Hz, 3H), 0.86 (d, *J* = 3.2 Hz, 3H), 0.74 (s, 3H);

^{13}C NMR (176 MHz, $CDCl_3$): δ 202.8, 139.3, 134.2 (q, *J* = 32.6 Hz), 128.7 (2C), 125.9 (2C, q, *J* = 3.6 Hz), 123.8 (q, *J* = 273 Hz), 74.3, 57.7, 56.6, 56.2, 45.3, 43.1, 40.1, 39.7, 38.1, 36.6, 36.3, 36.1, 36.0, 34.1, 31.7, 31.4, 29.8, 28.4, 28.2, 24.3, 24.0, 23.0, 22.7, 20.1, 19.3, 18.8, 12.9;

IR (neat) ν_{max}: 3536, 3501, 2950, 2929, 2868, 2846, 1690, 1582, 1467, 1443, 1409, 1382, 1324, 1279, 1244, 1208, 1169, 1132, 1110, 1068, 1016, 984, 959, 913, 888, 852, 832, 773 cm^{-1};

HRMS (ESI$^+$): exact mass calculated for $[M+Na]^+$ ($C_{35}H_{51}F_3O_2Na$) requires *m/z* 583.3733, found *m/z* 583.3723.

2c – *cis*-(3-Hydroxy-3,5,5-trimethylcyclohexyl)[4-(trifluoromethyl)phenyl]methanone

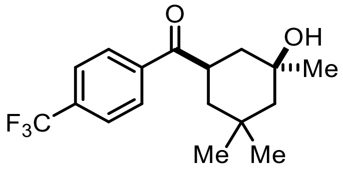

Formula: $C_{17}H_{21}F_3O_2$

MW: 314.3 g/mol

Synthesized following **GP-8**, using **1c** (37.1 mg, 0.100 mmol, 1.00 equiv.) and $(PhI)_2O(SbF_6)_2$ (89.6 mg, 0.100 mmol, 1.00 equiv.). Purification by flash column chromatography on silica gel (heptanes/EtOAc, 95:5 to 55:45) afforded the title compound **2c** (20.1 mg, 64.0 µmol, 64%) as a crystalline solid as well as the Me-shifted product **2c'** (10.7 mg, 34.0 µmol, 34%) as a colorless oil.

A single crystal, suitable for X-ray analysis, was obtained by slow evaporation, at rt, of a diethyl ether solution of the compound, which had been slowly covered by heptanes so as to ensure that the mixture remains biphasic. The x-ray crystallographic details can be found in section 8.

^{1}H NMR (600 MHz, $CDCl_3$): δ 8.01 (d, *J* = 8.2 Hz, 2H), 7.74 (d, *J* = 8.2 Hz, 2H), 3.52 (tt, *J* = 11.4, 3.4 Hz, 1H), 1.88 (ddt, *J* = 13.1, 3.2, 1.7 Hz, 1H), 1.83 (br s, 1H), 1.67 (t, *J* = 12.0 Hz, 1H), 1.64 – 1.60 (m, 2H), 1.45 (d, *J* = 13.6 Hz, 1H), 1.43 (s, 3H), 1.40 (dd, *J* = 13.7, 11.6 Hz, 1H), 1.09 (s, 3H), 0.99 (s, 3H);

^{13}C NMR (151 MHz, $CDCl_3$): δ 202.1, 139.0, 134.4 (q, *J* = 32.4 Hz), 128.8 (2C), 126.0 (q, *J* = 4.0 Hz, 2C), 123.4 (q, *J* = 273.2 Hz), 71.0, 52.8, 42.2, 41.8, 41.2, 33.7, 32.2, 29.2, 27.6;

^{19}F NMR (565 MHz, $CDCl_3$): δ -63.1 (3F);

IR (neat) ν_{max}: 3406 (br), 2953, 2927, 1685, 1409, 1322, 1286, 1165, 1125, 1110, 1066, 1014, 903, 853, 833, 741 cm^{-1};

HRMS (ESI$^+$): exact mass calculated for $[M+Na]^+$ ($C_{17}H_{21}F_3O_2Na^+$) requires *m/z* 337.1386, found *m/z* 337.1381.

2c' – *cis*-(1,3-*cis*-3-hydroxy-2,3,5-trimethylcyclohexyl)[4-(trifluoromethyl)phenyl]methanone

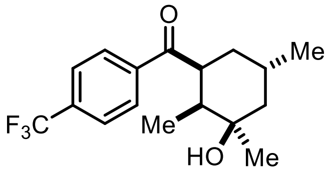

Formula: $C_{17}H_{21}F_3O_2$

MW: 314.3 g/mol

^{1}H NMR (600 MHz, $CDCl_3$): δ 8.08 (d, *J* = 8.2 Hz, 2H), 7.74 (d, *J* = 8.2 Hz, 2H), 5.67 (br s, 1H, *O–H*), 4.00 – 3.92 (m, 1H), 1.87 – 1.77 (m, 3H), 1.77 – 1.68 (m, 1H), 1.48 (ddd, *J* = 13.6, 12.4, 7.1 Hz, 1H), 1.23 (s, 3H), 1.10 (t, *J* = 13.0 Hz, 1H), 1.04 (d, *J* = 7.2 Hz, 3H), 0.84 (d, *J* = 6.6 Hz, 3H);

^{13}C NMR (151 MHz, $CDCl_3$): δ 205.1, 139.4, 135.0 (q, *J* = 33. z), 129.2 (2C), 126.0 (q, *J* = 3.4 Hz, 2C), 123.7 (q, *J* = 272.8 Hz), 69.7, 50.2, 48.6, 40.8, 37.4, 28.6, 23.2, 21.9, 13.1;

^{19}F NMR (565 MHz, $CDCl_3$): δ -63.2 (3F);

IR (neat) ν_{max}: 3426, 2954, 2926, 2873, 1672, 1580, 1510, 1456, 1410, 1376, 1323, 1275, 1212, 1167, 1128, 1066, 1045, 1010, 990, 959, 932, 911, 894, 854, 801, 777 cm^{-1}

HRMS (ESI$^+$): exact mass calculated for $[M+Na]^+$ ($C_{17}H_{21}F_3O_2Na^+$) requires m/z 337.1386, found m/z 337.1378.

2f – *cis*-(Hydroxycyclopentyl)[4-(trifluoromethyl)phenyl]methanone

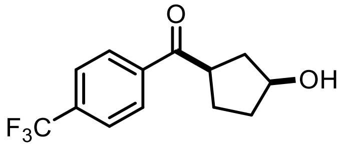

Formula: $C_{13}H_{13}F_3O_2$

MW: 258.2 g/mol

Synthesized following **GP-8**, using **1f** (47.2 mg, 0.150 mmol, 1.50 equiv.) and $(PhI)_2O(SbF_6)_2$ (89.6 mg, 0.100 mmol, 1.00 equiv.). Purification by flash column chromatography on silica gel (heptanes/EtOAc, 90:10 to 55:45) afforded the title compound (19.1 mg, 74.0 µmol, 74%) as a yellowish oil.

5 mmol scale: Synthesized following **GP-8**, using **1f** (2.36 g, 7.50 mmol, 1.50 equiv.) and $(PhI)_2O(SbF_6)_2$ (4.48 g, 5.00 mmol, 1.00 equiv.). The solution of **1f** in CH_2Cl_2 (0.15 M) was added over 5 min, using an addition funnel. Purification by flash column chromatography on silica gel (heptanes/EtOAc, 90:10 to 50:50) afforded the title compound (889 mg, 3.44 mmol, 69%) as a yellowish oil.

^{1}H NMR (600 MHz, $CDCl_3$): δ 8.08 (d, *J* = 8.2 Hz, 2H), 7.75 (d, *J* = 8.2 Hz, 2H), 4.40 (app br s, 1H), 3.93 – 3.86 (m, 1H), 2.65 (br d, *J* = 6.0 Hz, 1H, *O-H*), 2.18 – 2.07 (m, 3H), 2.06 – 1.98 (m, 1H), 1.90 – 1.81 (m, 2H);

^{13}C NMR (151 MHz, $CDCl_3$): δ = 203.2, 138.9, 134.6 (q, *J* = 33.0 Hz), 129.0 (2C), 125.7 (q, *J* = 4.2 Hz, 2C), 123.5 (q, *J* = 272.5 Hz), 73.7, 44.8, 38.0, 36.1, 28.6;

^{19}F NMR ($CDCl_3$, 565 MHz): δ = -63.1 (3 F);

IR (neat) ν_{max}: 3403, 2955, 1686, 1410, 1324, 1252, 1222, 1167, 1129, 1067, 1015, 957, 854 cm^{-1};

HRMS (ESI$^+$): exact mass calculated for $C_{13}H_{13}F_3O_2Na^+$ $[M+Na]^+$ requires *m/z* 281.0760, found *m/z* 281.0766.

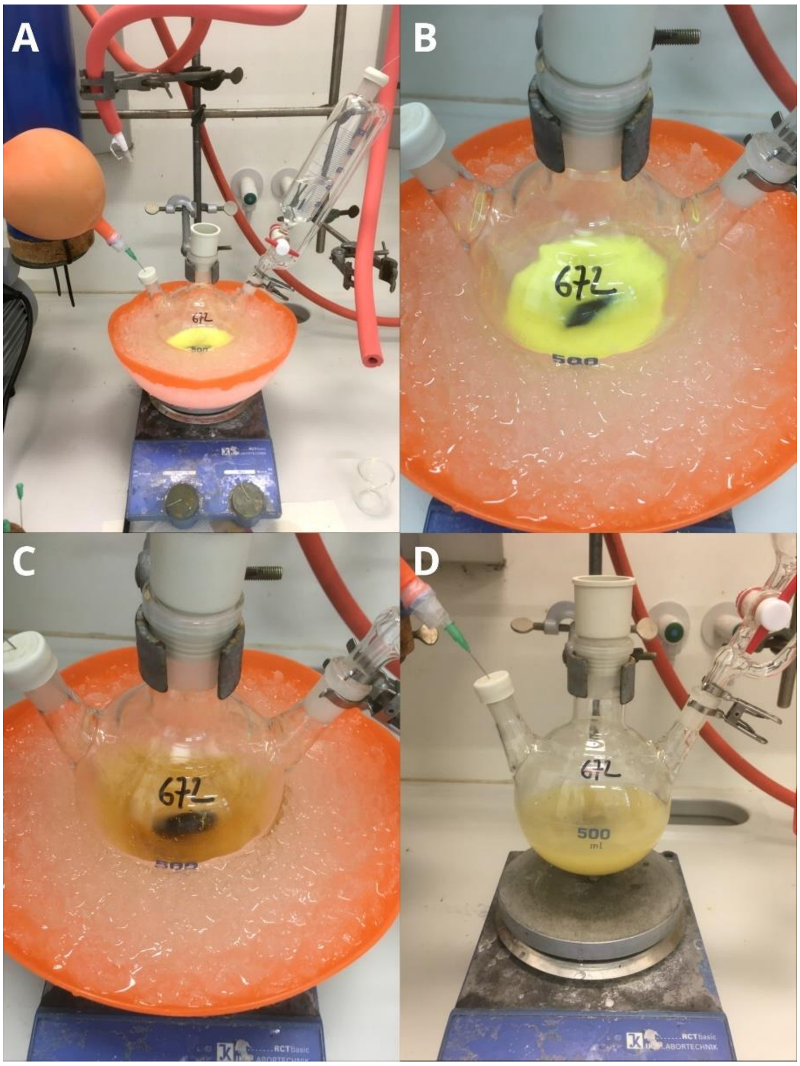

Figure S4. Synthesis of 2f on a 5 mmol scale. A. Reaction set-up before the addition of **1f**. **B.** Reaction mixture before addition of **1f** (magnified view). **C.** Reaction mixture after addition of **1f**. **D.** Reaction mixture after addition of $NaHCO_3$ (sat. aq.).

2g – *cis*-(3-Hydroxycyclopentyl)[3-(trifluoromethyl)phenyl]methanone

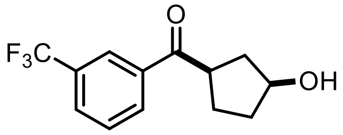

Formula: $C_{13}H_{13}F_3O_2$

MW: 258.2 g/mol

Synthesized following **GP-8**, using **1g** (47.2 mg, 0.150 mmol, 1.50 equiv.) and $(PhI)_2O(SbF_6)_2$ (89.6 mg, 0.100 mmol, 1.00 equiv.). Purification by flash column chromatography on silica gel (heptanes/EtOAc, 90:10 to 55:45) afforded the title compound (19.2 mg, 74.3 µmol, 74%) as a yellowish oil.

^{1}H NMR (700 MHz, $CDCl_3$): δ 8.22 (s, 1H), 8.16 (d, *J* = 7.7 Hz, 1H), 7.84 (d, *J* = 7.8 Hz, 1H), 7.63 (t, *J* = 7.8 Hz, 1H), 4.40 (m, 1H), 3.90 (tdd, *J* = 9.2, 6.4, 4.5 Hz, 1H), 2.72 (br s, 1H), 2.17 – 2.07 (m, 3H), 2.05 – 1.99 (m, 1H), 1.90 – 1.81 (m, 2H);

^{13}C NMR (176 MHz, $CDCl_3$): δ 203.1, 136.8, 131.9, 131.5 (q, *J* = 33.1 Hz), 129.8 (q, *J* = 2.7 Hz), 129.5, 125.7 (q, *J* = 3.9 Hz), 123.8 (q, *J* = 273.5 Hz), 73.8, 44.8, 38.1, 36.3, 28.8;

^{19}F NMR ($CDCl_3$, 659 MHz): δ -62.8 (3F);

IR (neat) ν_{max}: 3403 (br), 2944, 1686, 1610, 1327, 1205, 1165, 1122, 1071, 694 cm^{-1};

HRMS (ESI$^+$): exact mass calculated for $[M+Na]^+$ ($C_{13}H_{13}F_3O_2Na^+$) requires *m/z* 281.0760, found *m/z* 281.0754.

2h – *cis*-(3-Hydroxycyclopentyl)[2-(trifluoromethyl)phenyl]methanone

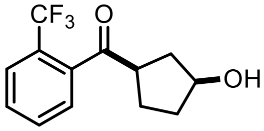

Formula: $C_{13}H_{13}F_3O_2$

MW: 258.2 g/mol

Synthesized following **GP-8**, using **1h** (47.2 mg, 0.150 mmol, 1.50 equiv.) and $(PhI)_2O(SbF_6)_2$ (89.6 mg, 0.100 mmol, 1.00 equiv.). Purification by flash column chromatography on silica gel (heptanes/EtOAc, 90:10 to 55:45) afforded the title compound (18.5 mg, 71.6 µmol, 72%) as a yellowish oil.

^{1}H NMR (400 MHz, $CDCl_3$): δ 7.72 (d, *J* = 7.6 Hz, 1H), 7.60 (q, *J* = 6.5 Hz, 1H), 7.57 (q, *J* = 7.3 Hz, 1H), 7.46, (d, *J* = 7.3 Hz, 1H), 4.35 (app br s, 1H), 3.62 – 3.53 (m, 1H), 2.51 (br s, 1H, *O-H*), 2.14 – 1.93 (m, 4H), 1.85 – 1.78 (m, 2H);

^{13}C NMR (101 MHz, $CDCl_3$): δ 209.1, 140.0 (q, *J* = 2.4 Hz), 132.0, 130.4, 127.6, 127.3 (q, *J* = 32.4 Hz), 127.0 (q, *J* = 4.8 Hz), 123.7 (q, *J* = 275.5), 73.7, 49.6, 38.6, 36.1, 28.2;

^{19}F NMR (376 MHz, $CDCl_3$) δ -57.9 (3F);

IR (neat) ν_{max}: 3388, 2961, 1699, 1447, 1313, 1275, 1221, 1167, 1131, 1065, 1035, 769 cm^{-1};

HRMS (ESI$^+$): exact mass calculated for $[M+Na]^+$ ($C_{13}H_{13}F_3O_2Na^+$) requires *m/z* 281.0760, found *m/z* 281.0760.

2i – *cis*-4-(3-Hydroxycyclopentane-1-carbonyl)benzonitrile

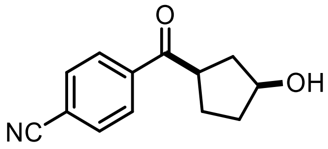

Formula: $C_{13}H_{13}NO_2$

MW: 215.2 g/mol

Synthesized following **GP-8**, using **1i** (40.7 mg, 0.150 mmol, 1.50 equiv.) and $(PhI)_2O(SbF_6)_2$ (89.6 mg, 0.100 mmol, 1.00 equiv.). Purification by flash column chromatography on silica gel (heptanes/EtOAc, 90:10 to 60:40) afforded the title compound (8.9 mg, 41.0 µmol, 41%) as a colorless oil.

^{1}H NMR (600 MHz, $CDCl_3$): δ 8.06 (d, J = 8.6 Hz, 2H), 7.78 (d, J = 8.6 Hz, 2H), 4.43 – 4.37 (m, 1H), 3.88 – 3.82 (m, 1H), 2.53 (br s, 1H, *O–H*), 2.16 – 2.06 (m, 3H), 2.05 – 1.98 (m, 1H), 1.91 – 1.80 (m, 2H);

^{13}C NMR (151 MHz, $CDCl_3$): δ 202.8, 139.4, 132.7 (2C), 129.2 (2C), 118.0, 116.6, 73.7, 45.0, 38.1, 36.2, 28.5;

IR (neat) ν_{max}: 3404, 2938, 2231, 1683, 1406, 1291, 1216, 1080, 997, 851, 757, 544 cm^{-1};

HRMS (ESI$^+$): exact mass calculated for $[M+Na]^+$ ($C_{13}H_{13}NO_2Na^+$) requires m/z 238.0838, found m/z 238.0848.

2j – *cis*-Methyl 4-(3-hydroxycyclopentane-1-carbonyl)benzoate

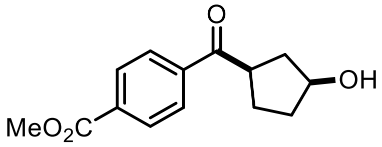

Formula: $C_{14}H_{16}O_4$

MW: 248.3 g/mol

Synthesized following **GP-8**, using **1j** (45.7 mg, 0.150 mmol, 1.50 equiv.) and $(PhI)_2O(SbF_6)_2$ (89.6 mg, 0.100 mmol, 1.00 equiv.). Purification by flash column chromatography on silica gel (heptanes/EtOAc, 90:10 to 50:50) afforded the title compound (12.7 mg, 51.2 µmol, 51%) as a colorless viscous oil.

^{1}H NMR (400 MHz, $CDCl_3$): δ 8.13 (d, *J* = 8.5 Hz, 2H), 8.03 (d, *J* = 8.5 Hz, 2H), 4.39 (app br s, 1H), 3.95 (s, 3H), 4.00 – 3.87 (m, 1H), 2.74 (br s, 1 H, *O-H*), 2.18 – 2.07 (m, 3H), 2.06 – 1.94 (m, 1H), 1.89 – 1.81 (m, 2H);

^{13}C NMR (101 MHz, $CDCl_3$): δ 204.1, 166.3, 139.5, 134.2, 130.0 (2C), 128.7 (2C), 73.8, 52.6, 45.0, 38.1, 36.3, 28.8;

IR (neat) ν_{max}: 3429, 2954, 1723, 1684, 1436, 1407, 1280, 1221, 1108, 1015, 721 cm^{-1};

HRMS (ESI$^+$): exact mass calculated for $[M+Na]^+$ ($C_{14}H_{16}O_4Na^+$) requires *m/z* 271.0941, found *m/z* 271.0948.

2k – (2,6-dichlorophenyl)(3-hydroxycyclopentyl)methanone

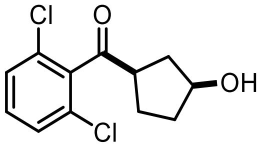

Formula: $C_{12}H_{12}Cl_2O_2$

MW: 259.1 g/mol

Synthesized following **GP-8**, using **1k** (47.3 mg, 0.150 mmol, 1.50 equiv.) and $(PhI)_2O(SbF_6)_2$ (89.6 mg, 0.100 mmol, 1.00 equiv.). Purification by flash column chromatography on silica gel (heptanes/EtOAc, 95:5 to 50:50) afforded the title compound (15.4 mg, 59.4 µmol, 59%) as a colorless solid.

^{1}H NMR (600 MHz, $CDCl_3$): δ 7.30 (dd, *J* = 7.9, 1.3 Hz, 2H), 7.23 (dd, *J* = 5.8, 3.5 Hz, 1H), 4.34 (app s, 1H), 3.50 (tdd, *J* = 8.9, 7.2, 5.7 Hz, 1H), 2.37 (app br s, 1H), 2.26 – 2.04 (m, 3H), 1.95 (ddt, *J* = 13.0, 8.8, 6.3 Hz, 1H), 1.82 (ddd, *J* = 8.6, 6.4, 4.7 Hz, 2H);

^{13}C NMR (151 MHz, $CDCl_3$): δ 206.7, 140.0, 130.9 (2C), 130.8, 128.5 (2C), 73.7, 50.8, 38.5, 36.2, 27.9;

IR (neat) ν_{max}: 3559, 3393, 3072, 2967, 2941, 2871, 1741, 1704, 1692, 1661, 1578, 1559, 1466, 1427, 1352, 1328, 1298, 1265, 1241, 1213, 1191, 1152, 1105, 1085, 1067, 1007, 984, 956, 919, 880, 852, 777 cm^{-1};

HRMS (ESI$^+$): exact mass calculated for $[M+Na]^+$ ($C_{12}H_{12}Cl_2O_2Na$) requires *m/z* 281.0107, found *m/z* 281.0110.

2l – *cis*-(4-bromophenyl)(3-hydroxycyclopentyl)methanone

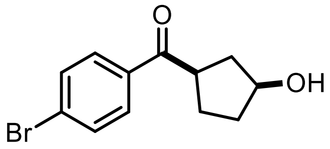

Formula: $C_{12}H_{13}BrO_2$

MW: 269.1 g/mol

Synthesized following **GP-8**, using **1l** (97.6 mg, 0.300 mmol, 1.50 equiv.) and $(PhI)_2O(SbF_6)_2$ (179 mg, 0.200 mmol, 1.00 equiv.). Purification by flash column chromatography on silica gel (heptanes/EtOAc, 90:10 to 55:45) afforded the title compound (33.1 mg, 123 µmol, 61%) as a yellowish oil.

^{1}H NMR (700 MHz, $CDCl_3$): δ 7.86 – 7.83 (m, 2H), 7.63 – 7.61 (m, 2H), 4.38 (app br s, 1H), 3.86 (dq, *J* = 9.5, 6.5 Hz, 1H), 2.84 (br s, 1H, *O–H*), 2.15 – 2.05 (m, 3H), 2.01 – 1.94 (m, 1H), 1.86 – 1.81 (m, 2H);

^{13}C NMR (176 MHz, $CDCl_3$): δ 203.6, 134.9, 132.2 (2C), 130.4 (2C), 128.7, 73.8, 44.6, 38.1, 36.4, 28.9;

IR (neat) ν_{max}: 3406 (br), 2939, 2870, 1676, 1583, 1484, 1396, 1278, 1216, 1069, 1006, 837, 739 cm^{-1};

HRMS (ESI$^+$): exact mass calculated for $[M+Na]^+$ ($C_{12}H_{13}BrO_2Na^+$) requires *m/z* 290.9991 and 292.9972, found *m/z* 290.9984 and 292.9964.

2m – *cis*-(3-Hydroxycyclopentyl)(phenyl)methanone

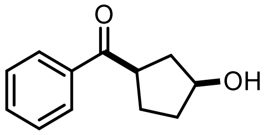

Formula: $C_{12}H_{14}O_2$

MW: 190.2 g/mol

Synthesized following **GP-8**, using **1m** (37.0 mg, 0.150 mmol, 1.50 equiv.) and $(PhI)_2O(SbF_6)_2$ (89.6 mg, 0.100 mmol, 1.00 equiv.). Purification by flash column chromatography on silica gel (heptanes/EtOAc, 90:10 to 50:50) afforded the title compound (10.8 mg, 56.8 µmol, 57%) as a yellowish oil.

^{1}H NMR (400 MHz, $CDCl_3$): δ 8.02 – 7.96 (m, 2H), 7.58 (dd, *J* = 10.5, 4.2 Hz, 1H), 7.48 (t, *J* = 7.6 Hz, 2H), 4.42 – 4.33 (m, 1H), 3.94 (ddd, *J* = 13.3, 9.4, 6.2 Hz, 1H), 3.05 (br s, 1H, *O–H*), 2.18 – 2.04 (m, 3H), 2.04 – 1.93 (m, 1H), 1.88 – 1.80 (m, 2H);

^{13}C NMR (101 MHz, $CDCl_3$): δ 205.0, 136.2, 133.5, 128.9 (2C), 128.8 (2C), 73.9, 44.5, 38.2, 36.4, 29.1;

IR (neat) ν_{max}: 3394, 2955, 2870, 1677, 1596, 1579, 1448, 1361, 1281, 1225, 1180, 1160, 1079, 1052, 1002, 993, 958, 932, 882, 842, 790, 766, 701 cm^{-1};

HRMS (ESI$^+$): exact mass calculated for $[M+Na]^+$ ($C_{12}H_{14}O_2Na^+$) requires *m/z* 213.0886, found *m/z* 213.0879.

2n – *cis*-(3-Hydroxycyclopentyl)(naphthalen-2-yl)methanone

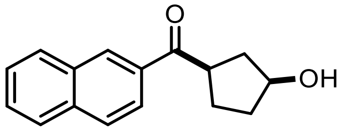

Formula: $C_{16}H_{16}O_2$

MW: 240.3 g/mol

Synthesized following **GP-8**, using **1n** (44.5 mg, 0.150 mmol, 1.50 equiv.) and $(PhI)_2O(SbF_6)_2$ (89.6 mg, 0.100 mmol, 1.00 equiv.). Purification by flash column chromatography on silica gel (heptanes/EtOAc, 95:5 to 50:50) afforded the title compound (12.5 mg, 52.0 µmol, 52%) as a yellowish oil.

^{1}H NMR (400 MHz, $CDCl_3$): δ 8.52 (s, 1H), 8.06 (dd, *J* = 8.6, 1.7 Hz, 1H), 7.98 (d, *J* = 8.0 Hz, 1H), 7.94 – 7.86 (m, 2H), 7.62 (td, *J* = 6.8, 1.2 Hz, 1H), 7.56 (td, *J* = 8.2, 1.0 Hz, 1H), 4.42 (app s, 1H), 4.20 – 4.08 (m, 1H), 3.13 (br s, 1H, *O–H*), 2.28 – 2.11 (m, 3H), 2.11 – 2.01 (m, 1H), 1.92 – 1.85 (m, 2H);

^{13}C NMR (101 MHz, $CDCl_3$): δ 205.0, 135.9, 133.5, 132.7, 130.7, 129.8, 128.8, 128.7, 128.0, 127.0, 124.5, 74.0, 44.6, 38.4, 36.6, 29.4;

IR (neat) ν_{max}: 3405 (br), 3057, 2868, 1671, 1625, 1595, 1464, 1435, 1277, 1253, 1217, 1185, 1124, 1080, 909, 864, 823, 757 cm^{-1};

HRMS (ESI$^+$): exact mass calculated for $[M+Na]^+$ ($C_{16}H_{16}O_2Na^+$) requires *m/z* 263.1043, found *m/z* 263.1045.

2o – *cis*-3-Hydroxy-[4-(trifluoromethyl)phenyl]methanone

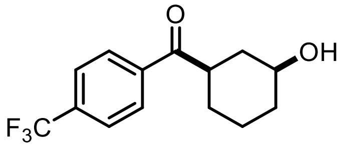

Formula: $C_{14}H_{15}F_3O_2$

MW: 272.3 g/mol

Synthesized following **GP-8**, using **1o** (49.3 mg, 0.150 mmol, 1.50 equiv.) and $(PhI)_2O(SbF_6)_2$ (89.6 mg, 0.100 mmol, 1.00 equiv.). Purification by flash column chromatography on silica gel (heptanes/EtOAc, 90:10 to 60:40) afforded the title compound (14.3 mg, 52.5 µmol, 52%) as a colorless oil.

^{1}H NMR (400 MHz, $CDCl_3$): δ 8.02 (d, *J* = 8.1 Hz, 2H), 7.73 (d, *J* = 8.2 Hz, 2H), 3.78 (tt, *J* = 10.6, 4.2 Hz, 1H), 3.35 (tt, *J* = 11.3, 3.4 Hz, 1H), 2.19 – 2.11 (m, 1H), 2.10 – 2.00 (m, 1H), 1.98 – 1.83 (m, 1H), 1.83 – 1.61 (m, 1H), 1.61 – 1.38 (m, 1H), 1.38 – 1.18 (m, 1H);

^{13}C NMR (176 MHz, $CDCl_3$): δ 201.5, 139.0, 134.5 (q, *J* = 32.7 Hz), 128.8 (2C), 125.9 (q, *J* = 3.7 Hz, 2C), 123.72 (q, *J* = 272.7 Hz), 70.1, 44.5, 37.7, 35.3, 28.5, 23.5;

^{19}F NMR (376 MHz, $CDCl_3$): δ -63.1 (3F);

jIR (neat) ν_{max}: 3352, 2932, 2858, 1686, 1451, 1409, 1324, 1262, 1207, 1168, 1129, 1112, 1067, 1014, 976, 956, 946, 882, 849, 813, 767 cm^{-1};

HRMS (ESI$^+$): exact mass calculated for $[M+Na]^+$ ($C_{14}H_{15}F_3O_2Na^+$) requires *m/z* 295.0921, found *m/z* 295.0916.

2p – cis-Benzo[*b*]thiophen-2-yl(3-hydroxycyclohexyl)methanone

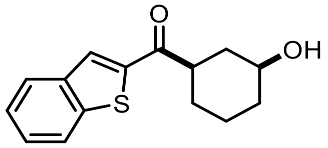

Formula: $C_{15}H_{16}O_2S$

MW: 260.4 g/mol

Synthesized following **GP-8**, using **1p** (47.5 mg, 0.150 mmol, 1.50 equiv.) and $(PhI)_2O(SbF_6)_2$ (89.6 mg, 0.100 mmol, 1.00 equiv.). Purification by flash column chromatography on silica gel (heptanes/EtOAc, 95:5 to 50:50) afforded the title compound (39.2 mg, 39.2 µmol, 39%) as a colorless solid.

^{1}H NMR (400 MHz, $CDCl_3$): δ 7.97 (s, 1H), 7.88 (t, *J* = 7.5 Hz, 2H), 7.52 – 7.44 (m, 1H), 7.44 – 7.38 (m, 1H), 3.83 – 3.71 (m, 1H), 3.34 (ddd, *J* = 11.3, 7.3, 3.3 Hz, 1H), 2.22 (dd, *J* = 12.6, 3.8 Hz, 1H), 2.05 (d, *J* = 7.6 Hz, 2H), 2.00 – 1.89 (m, 2H), 1.73 (s, 1H), 1.70 – 1.42 (m, 5H), 1.41 – 1.20 (m, 3H);

^{13}C NMR (101 MHz, $CDCl_3$): δ 196.8, 143.0, 139.3, 129.0, 127.6, 126.1, 125.2, 123.2, 122.8, 70.1, 45.8, 38.0, 35.3, 29.0, 23.6;

IR (neat) ν_{max}: 3277, 2928, 2853, 2359, 2207, 2196, 2175, 2162, 2121, 2066, 2001, 1713, 1665, 1594, 1516, 1447, 1376, 1291, 1220, 1096, 1048, 1009, 918, 871, 815, 727 cm^{-1};

HRMS (ESI$^+$): exact mass calculated for $[M+H]^+$ ($C_{15}H_{16}O_2SH$) requires *m*/*z* 261.0944, found *m*/*z* 261.0944.

2q – *cis*-(3-(*tert*-Butyl)-3-hydroxycyclopentyl)[4-(trifluoromethyl)phenyl]methanone

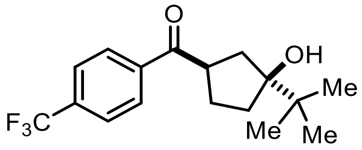

Formula: $C_{17}H_{21}F_3O_2$

MW: 314.3 g/mol

Synthesized following **GP-8**, using **1q** (55.6 mg, 0.150 mmol, 1.50 equiv.) and $(PhI)_2O(SbF_6)_2$ (89.6 mg, 0.100 mmol, 1.00 equiv.). Purification by flash column chromatography on silica gel (heptanes/EtOAc, 95:5 to 55:45) afforded the title compound (25.4 mg, 80.8 µmol, 81%) as a crystalline solid.

A single crystal, suitable for X-ray analysis, was obtained by slow evaporation, at rt, of a diethyl ether solution of the compound, which had been slowly covered by heptanes so as to ensure that the mixture remains biphasic. The x-ray crystallographic details can be found in section 8.

^{1}H NMR (400 MHz, CDCl$_3$): δ 8.06 (d, *J* = 8.1 Hz, 2H), 7.73 (d, *J* = 8.1 Hz, 2H), 3.93 – 3.83 (m, 1H), 2.64 (app s, 1H), 2.21 – 2.09 (m, 2H), 2.09 – 1.95 (m, 2H), 1.93 – 1.83 (m, 1H), 1.76 – 1.68 (m, 1H), 1.01 (s, 9H);

^{13}C NMR (101 MHz, CDCl$_3$): δ 203.5, 139.1, 134.6 (q, *J* = 32.7 Hz), 129.1 (2C), 125.9 (q, *J* = 3.9 Hz, 2C), 122.4 (q, *J* = 273.1 Hz), 87.1, 45.5, 37.1, 36.5, 35.3, 29.7, 26.1 (3C);

^{19}F NMR (376 MHz, CDCl$_3$): δ -63.1 (3F);

IR (neat) ν_{max}: 3513, 2976, 1949, 2868, 1671, 1323, 1312, 1217, 1168, 1137, 1111, 1065, 852 cm^{-1};

HRMS (ESI$^+$): exact mass calculated for $[M+Na]^+$ ($C_{17}H_{21}F_3O_2Na^+$) requires *m*/*z* 337.1386, found *m*/*z* 337.1377.

2r – *cis*-(3-Hydroxy-3-methylcyclopentyl)[4-(trifluoromethyl)phenyl]methanone

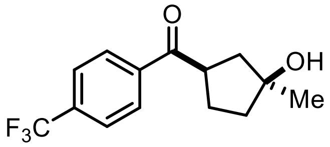

Formula: $C_{14}H_{15}F_3O_2$

MW: 272.3 g/mol

Synthesized following **GP-8**, using **1r** (49.3 mg, 0.150 mmol, 1.50 equiv.) and $(PhI)_2O(SbF_6)_2$ (89.6 mg, 0.100 mmol, 1.00 equiv.). Purification by flash column chromatography on silica gel (heptanes/EtOAc, 95:5 to 50:50) afforded the title compound (24.9 mg, 91.5 µmol, 92%) as a colorless oil.

^{1}H NMR (400 MHz, $CDCl_3$): δ 8.07 (d, *J* = 8.2 Hz, 2H), 7.74 (d, *J* = 8.2 Hz, 2H), 3.95 (tdd, *J* = 9.8, 6.8, 3.3 Hz, 1H), 2.23 – 2.11 (m, 2H), 2.11 – 2.00 (s, 1H), 1.95 (dd, *J* =m 14.0, 9.9 Hz, 1H), 1.88 (s, 1H), 1.78 – 1.68 (m, 1H), 1.41 (s, 3H);

^{13}C NMR (101 MHz, $CDCl_3$): δ 203.6, 139.0, 134.7 (q, *J* = 32.8 Hz), 129.1 (2C), 125.9 (q, *J* = 3.6 Hz, 2C), 123.7 (q, *J* = 272.3), 79.6, 45.4, 43.4, 41.9, 29.5, 26.9;

^{19}F NMR (376 MHz, $CDCl_3$): δ -63.2 (3F);

IR (neat) ν_{max}: 3451, 2965, 1686, 1581, 1511, 1410, 1323, 1222, 1166, 1125, 1065, 1014, 853 cm^{-1};

HRMS (ESI$^+$): exact mass calculated for $[M+Na]^+$ ($C_{14}H_{15}F_3O_2Na^+$) requires *m/z* 295.0916, found *m/z* 295.0924.

2s – *cis*-(3-Hydroxy-3-methylcyclopentyl)(phenyl)methanone

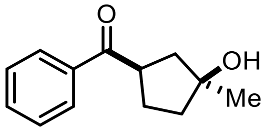

Formula: $C_{13}H_{16}O_2$

MW: 204.3 g/mol

Synthesized following **GP-8**, using **1s** (39.1 mg, 0.150 mmol, 1.50 equiv.) and $(PhI)_2O(SbF_6)_2$ (89.6 mg, 0.100 mmol, 1.00 equiv.). Purification by flash column chromatography on silica gel (heptanes/EtOAc, 95:5 to 50:50) afforded the title compound (16.2 mg, 79.3 µmol, 79%) as a colorless oil.

^{1}H NMR (600 MHz, $CDCl_3$): δ 7.97 (d, *J* = 7.4 Hz, 2H), 7.58 (t, *J* = 7.4 Hz, 1H), 7.47 (t, *J* = 7.6 Hz, 2H), 4.03 – 3.97 (m, 1H), 3.41 (app br s, 1H), 2.22 – 2.15 (m, 1H), 2.11 (dt, *J* = 13.8, 2.6 Hz, 1H), 2.08 – 2.00 (m, 1H), 1.92 (dd, *J* = 14.0, 9.8 Hz, 1H), 1.90 – 1.85 (m, 1H), 1.71 (dt, *J* = 12.9, 9.1 Hz, 1H), 1.41 (s, 3H);

^{13}C NMR (151 MHz, $CDCl_3$): δ 205.3, 136.2, 133.5, 128.86 (2C), 128.83 (2C), 79.6, 44.9, 43.6, 42.0, 29.9, 26.7;

IR (neat) ν_{max}: 3437 (br), 2962, 2931, 2871, 1679, 1596, 1580, 1447, 1363, 1224, 1075 cm^{-1};

HRMS (ESI$^+$): exact mass calculated for $[M+Na]^+$ ($C_{13}H_{16}O_2Na^+$) requires *m/z* 227.1043, found *m/z* 227.1045.

2t – *cis*-(4-Butyl-4-hydroxycyclohexyl)[4-(trifluoromethyl)phenyl]methanone

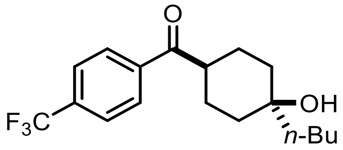

Formula: $C_{18}H_{23}F_3O_2$

MW: 328.4 g/mol

Synthesized following **GP-8**, using **1t** (57.7 mg, 0.150 mmol, 1.50 equiv.) and $(PhI)_2O(SbF_6)_2$ (89.6 mg, 0.100 mmol, 1.00 equiv.). Purification by flash column chromatography on silica gel (heptanes/EtOAc, 95:5 to 55:45) afforded the title compound (27.3 mg, 83.1 µmol, 83%) as a crystalline solid.

A single crystal, suitable for X-ray analysis, was obtained by slow evaporation, at rt, of a diethyl ether solution of the compound, which had been slowly covered by heptanes so as to ensure that the mixture remains biphasic. The x-ray crystallographic details can be found in section 8.

^{1}H NMR (700 MHz, CDCl$_3$): δ 8.03 (d, *J* = 8.2 Hz, 2H), 7.72 (d, *J* = 8.2 Hz, 2H), 3.18 (tt, *J* = 12.0, 3.3 Hz, 1H), 1.90 (qd, *J* = 12.5, 3.4 Hz, 2H), 1.79 – 1.73 (m, 4H), 1.53 – 1.45 (m, 4H), 1.39 – 1.30 (m, 4H), 1.19 (br s, 1H), 0.92 (t, *J* = 7.0 Hz, 3H);

^{13}C NMR (176 MHz, CDCl$_3$): δ 202.5, 139.2, 134.3 (q, *J* = 32.7 Hz), 128.7 (2C), 125.8 (q, *J* = 3.9 Hz, 2C), 123.8 (q, *J* = 272.1 Hz), 70.4, 45.8, 44.0, 36.3 (2C), 25.4, 24.6 (2C), 23.4, 14.2;

^{19}F NMR (659 MHz, CDCl$_3$): δ -63.1 (3F);

IR (neat) ν_{max}: 3516 (br), 2959, 2928, 2861, 1670, 1410, 1316, 1166, 1134, 1111, 1065, 1015, 964, 860, 735 cm^{-1};

HRMS (ESI$^+$): exact mass calculated for $[M+Na]^+$ ($C_{18}H_{23}F_3O_2Na^+$) requires *m/z* 351.1542, found *m/z* 351.1534.

2u – *cis*-[4-(*tert*-Butyl)-4-hydroxycyclohexyl][4-(trifluoromethyl)phenyl]methanone

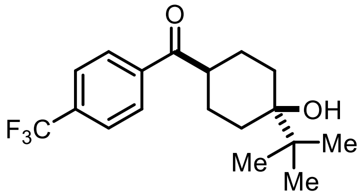

Formula: $C_{18}H_{23}F_3O_2$

MW: 328.4 g/mol

Synthesized following **GP-8**, using **1u** (57.7 mg, 0.150 mmol, 1.50 equiv.) and $(PhI)_2O(SbF_6)_2$ (89.6 mg, 0.100 mmol, 1.00 equiv.). Purification by flash column chromatography on silica gel (heptanes/EtOAc, 95:5 to 55:45) afforded the title compound (24.5 mg, 74.6 µmol, 75%) as a colorless solid.

^{1}H NMR (600 MHz, $CDCl_3$): δ 8.04 (d, *J* = 8.2 Hz, 2H), 7.72 (d, *J* = 8.2 Hz, 2H), 3.14 (tt, *J* = 12.2, 3.3 Hz, 1H), 1.89 (qd, *J* = 12.4, 3.8 Hz, 2H), 1.83 – 1.75 (m, 4H), 1.62 (td, *J* =k 13.4, 4.0 Hz, 2H), 1.13 (br s, 1H), 0.97 (s, 9H);

^{13}C NMR (151 MHz, $CDCl_3$): δ 202.6, 139.2, 134.3 (q, *J* = 32.4 Hz), 128.7 (2C), 125.8 (q, *J* = 3.3 Hz, 2C), 123.8 (q, *J* = 272.7 Hz), 74.4, 45.7, 37.8, 30.5 (2C), 25.2 (3C), 24.8 (2C);

^{19}F NMR (565 MHz, $CDCl_3$): δ -63.1 (3F);

IR (neat) ν_{max}: 3526, 2959, 2918, 2859, 1670, 1409, 1318, 1162, 1128, 1064, 954, 857 cm^{-1};

HRMS (ESI$^+$): exact mass calculated for $[M+Na]^+$ ($C_{18}H_{23}F_3O_2Na^+$) requires *m/z* 351.1542, found *m/z* 351.1538.

2v – *cis*-4-(*tert*-Butyl)-4-hydroxycyclohexyl)[3-(trifluoromethyl)phenyl]methanone

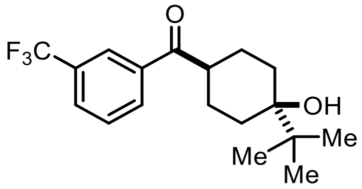

Formula: $C_{18}H_{23}F_3O_2$

MW: 328.4 g/mol

Synthesized following **GP-8**, using **1v** (57.7 mg, 0.150 mmol, 1.50 equiv.) and $(PhI)_2O(SbF_6)_2$ (89.6 mg, 0.100 mmol, 1.00 equiv.). Purification by flash column chromatography on silica gel (heptanes/EtOAc, 95:5 to 55:45) afforded the title compound (26.6 mg, 81.0 µmol, 81%) as a colorless solid.

^{1}H NMR (700 MHz, CDCl$_3$): δ 8.18 (s, 1H), 8.12 (d, *J* = 7.7 Hz, 1H), 7.80 (d, *J* = 7.8 Hz, 1H), 7.60 (t, *J* = 7.7 Hz, 1H), 3.15 (tt, *J* = 12.0, 3.1 Hz, 1H), 1.94 – 1.86 (m, 2H), 1.83 – 1.75 (m, 4H), 1.63 (td, *J* = 13.9, 4.2 Hz, 2H), 1.16 (br s, 1H, *O–H*), 0.98 (s, 9H);

^{13}C NMR (176 MHz, CDCl$_3$): δ 202.2, 137.0, 131.6, 131.4 (q, *J* = 33.6 Hz), 129.44, 129.36 (q, *J* = 3.7 Hz), 125.2 (q, *J* = 3.9 Hz), 123.9 (q, *J* = 273.1 Hz), 74.3, 45.5, 37.8, 30.5 (2C), 25.2 (3C), 24.8 (2C);

^{19}F NMR (659 MHz, CDCl$_3$): δ -62.7 (3F);

IR (neat) ν_{max}: 3544 (br), 2956, 2872, 1682, 1610, 1478, 1438, 1329, 1252, 1187, 1166, 1123, 1070, 996, 927, 694 cm^{-1};

HRMS (ESI$^+$): exact mass calculated for $[M+Na]^+$ ($C_{18}H_{23}F_3O_2Na^+$) requires *m/z* 351.1542, found *m/z* 351.1535.

2w – *cis*-(4-Hydroxy-4-methylcyclohexyl)[4-(trifluoromethyl)phenyl]methanone

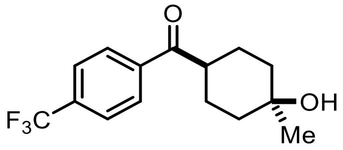

Formula: $C_{15}H_{17}F_3O_2$

MW: 286.3 g/mol

Synthesized following **GP-8**, using **1w** (51.4 mg, 0.150 mmol, 1.50 equiv.) and $(PhI)_2O(SbF_6)_2$ (89.6 mg, 0.100 mmol, 1.00 equiv.). Purification by flash column chromatography on silica gel (heptanes/EtOAc, 95:5 to 55:45) afforded the title compound (18.9 mg, 66.0 µmol, 66%) as a colorless solid.

^{1}H NMR (700 MHz, $CDCl_3$): δ 8.02 (d, *J* = 8.1 Hz, 2H), 7.72 (d, *J* = 8.1 Hz, 2H), 3.18 (tt, *J* = 11.8, 3.3 Hz, 1H), 1.90 (qd, *J* = 12.3, 2.8 Hz, 2H), 1.81 – 1.73 (m, 4H), 1.54 (td, *J* = 13.3, 3.8 Hz, 2H), 1.28 (s, 3H); *OH proton was not observed.*

^{13}C NMR (176 MHz, $CDCl_3$): δ 202.5, 139.2, 134.3 (q, *J* = 32.4 Hz), 128.7 (2C), 125.9 (q, *J* = 3.7 Hz, 2C), 123.8 (q, *J* = 273.1 Hz), 68.7, 45.3, 38.1 (2C), 31.4, 24.7 (2C);

^{19}F NMR (659 MHz, $CDCl_3$): δ -63.1 (3F);

IR (neat) ν_{max}: 3420 (br), 2928, 2869, 1682, 1510, 1446, 1410, 1319, 1207, 1166, 1125, 1065, 1014, 949, 856 cm^{-1};

HRMS (ESI$^+$): exact mass calculated for $[M+Na]^+$ ($C_{15}H_{17}F_3O_2Na^+$) requires *m/z* 309.1073, found *m/z* 309.1068.

NOESY experiment to demonstrate the relative stereochemistry

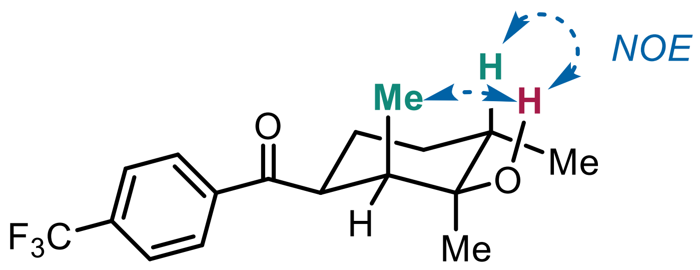

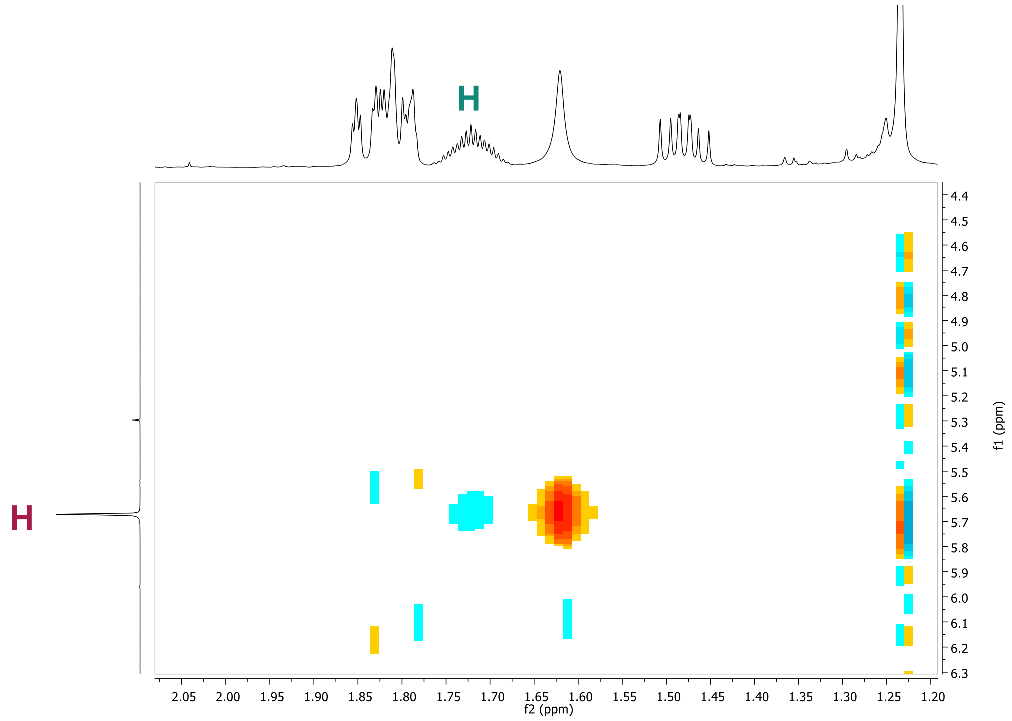

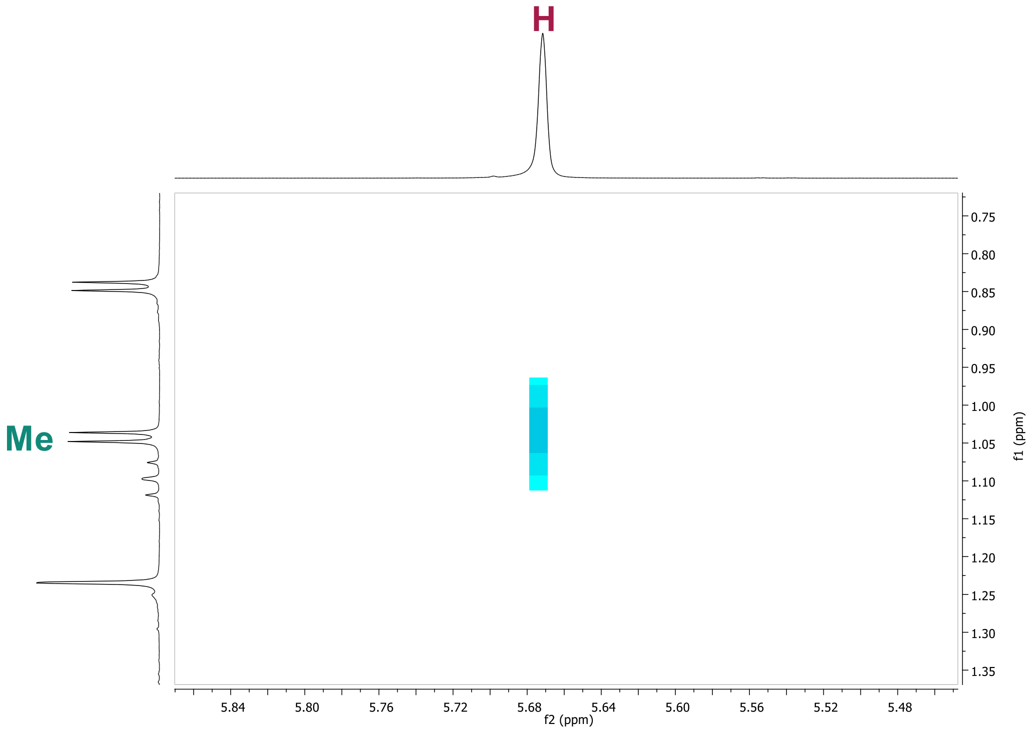

6 Comparison with White-Chen oxidation

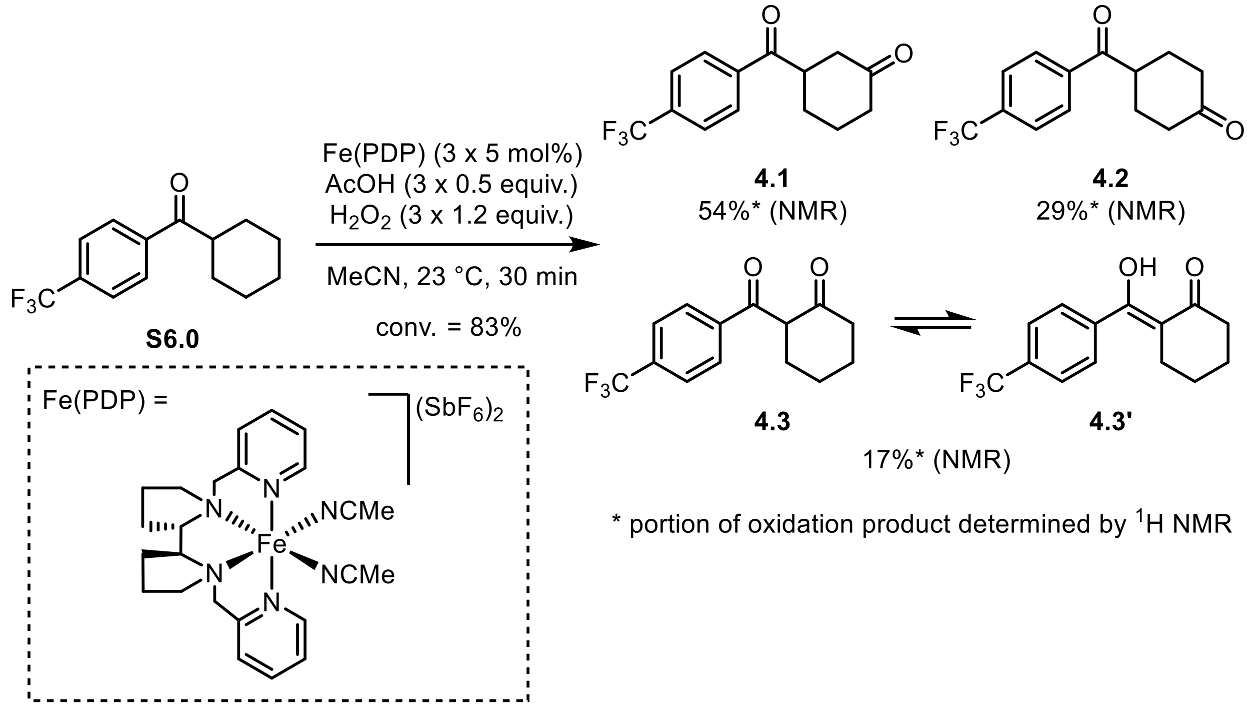

Figure S5. Comparison with White-Chen oxidation protocol.

Following the iterative procedure described by M. S. Chen and M. C. White.[71]

A stock solution of H_2O_2 in MeCN was prepared by adding H_2O_2 (30 w% in H_2O, 36.0 µL, 0.360 mmol, 3.60 equiv.) to MeCN (3.00 mL).

To a solution of **S6.0** (25.6 mg, 0.100 mmol, 1.00 equiv.), Fe(PDP) (4.70 mg, 5.00 µmol, 5 mol%) and AcOH (3.00 µL, 50.0 µmol, 0.500 equiv.) in MeCN (0.300 mL) at rt, the stock solution of H_2O_2 in MeCN (1.00 mL) was added dropwise, and the resulting mixture was left stirring for 10 min at rt. After this time, a solution of Fe(PDP) (4.70 mg, 5.00 µmol, 5 mol%) and AcOH (3.00 µL, 50.0 µmol, 0.500 equiv.) in MeCN (0.200 mL) was added, followed by dropwise addition of the stock solution of H_2O_2 in MeCN (1.00 mL), and the mixture was again left stirring for 10 min at rt. The last operation was performed one last time, giving a total of 3 rounds. Following this, the mixture was concentrated under reduced pressure and Et_2O was added, resulting in formation of a precipitate. The resulting mixture was filtered through Celite® and the pad was rinsed with Et_2O. The resulting solution was concentrated under reduced pressure. The residue was analyzed (NMR, according to $^{13}C\{^1H\}$ NMR: **4.1/4.2/4.3/4.3’** = 53:31:7:9) and subjected to flash column chromatography on silica gel (heptanes/EtOAc, 100:0 to 50:50). A fraction mainly composed of **4.3/4.3’** (*ca* 0.3 mg, *ca* 1%) was

obtained first, followed by a second fraction composed of **4.1** and **4.2** (10.0 mg, 0.037 mmol, 37%, **4.1**/**4.2** = 66:33).

Attribution of signals was made by comparison with previously reported similar structures.[72-74]

Reaction crude ^{1}H NMR (400 MHz, $CDCl_3$)

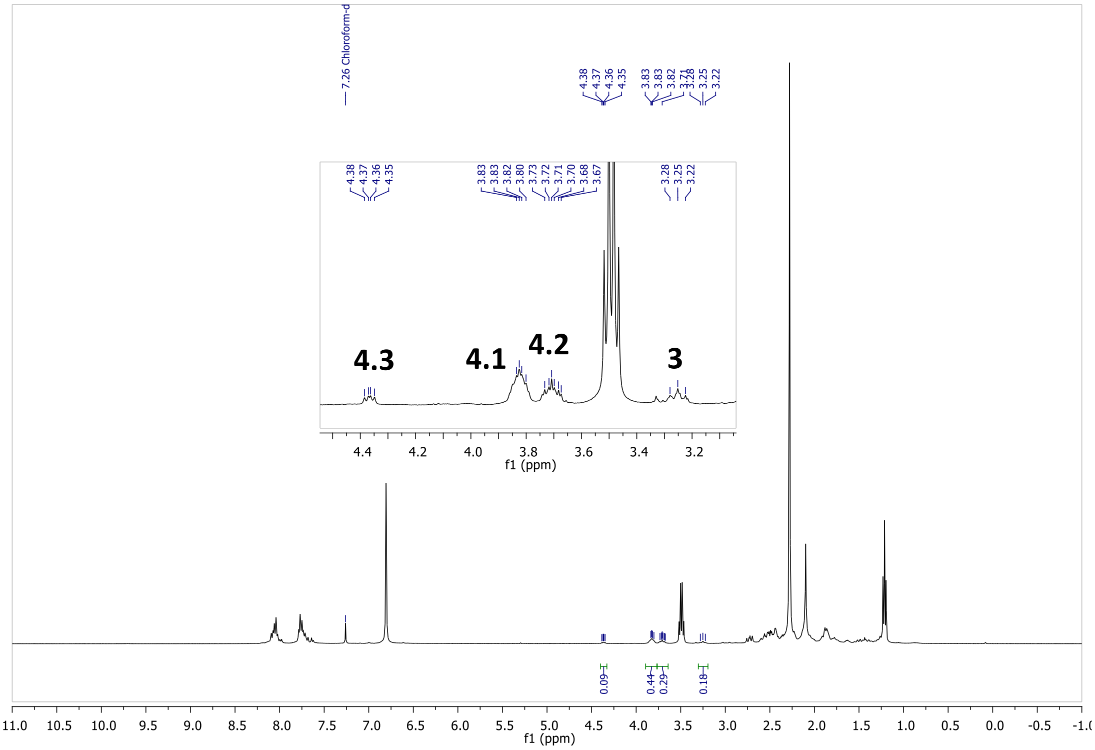

Reaction crude $^{13}C\{^{1}H\}$ NMR (151 MHz, $CDCl_3$)

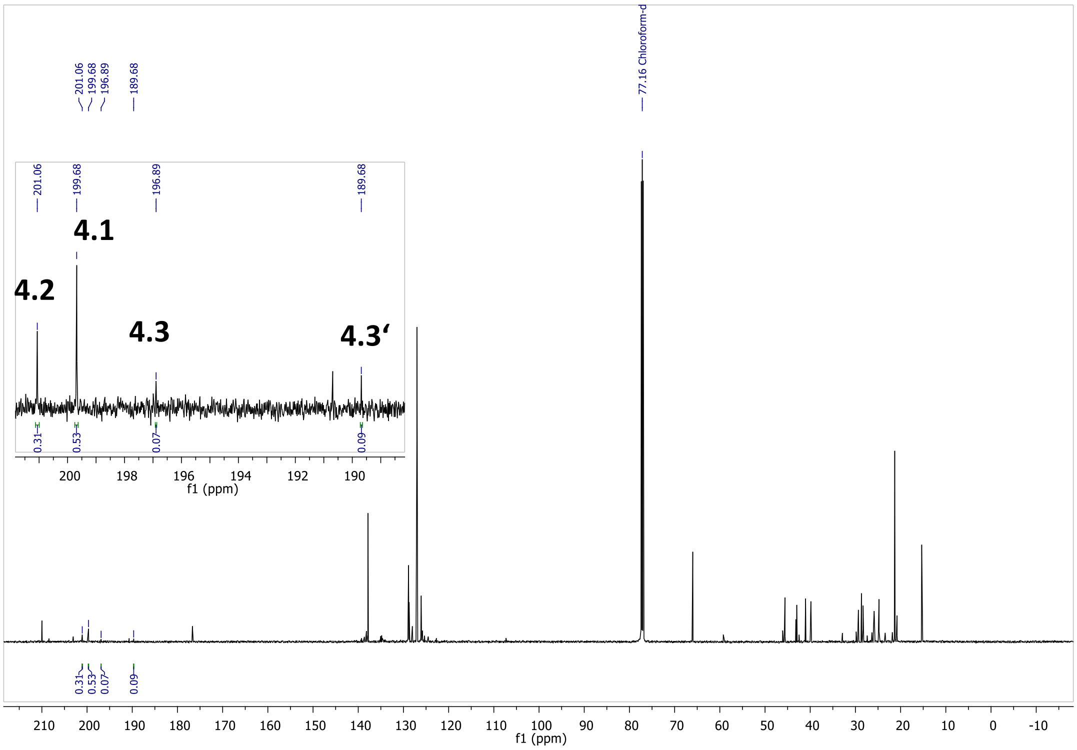

Reaction crude ^{19}F NMR (565 MHz, $CDCl_3$)

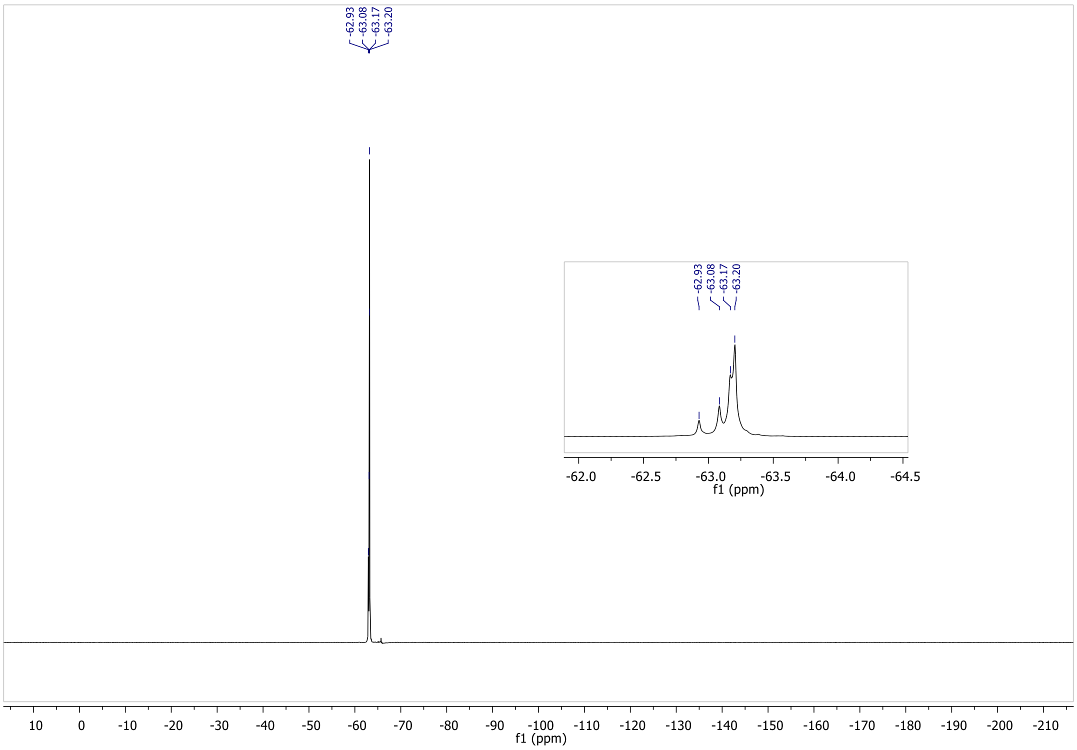

4.3/4.3' ^{1}H NMR (700 MHz, $CDCl_3$)

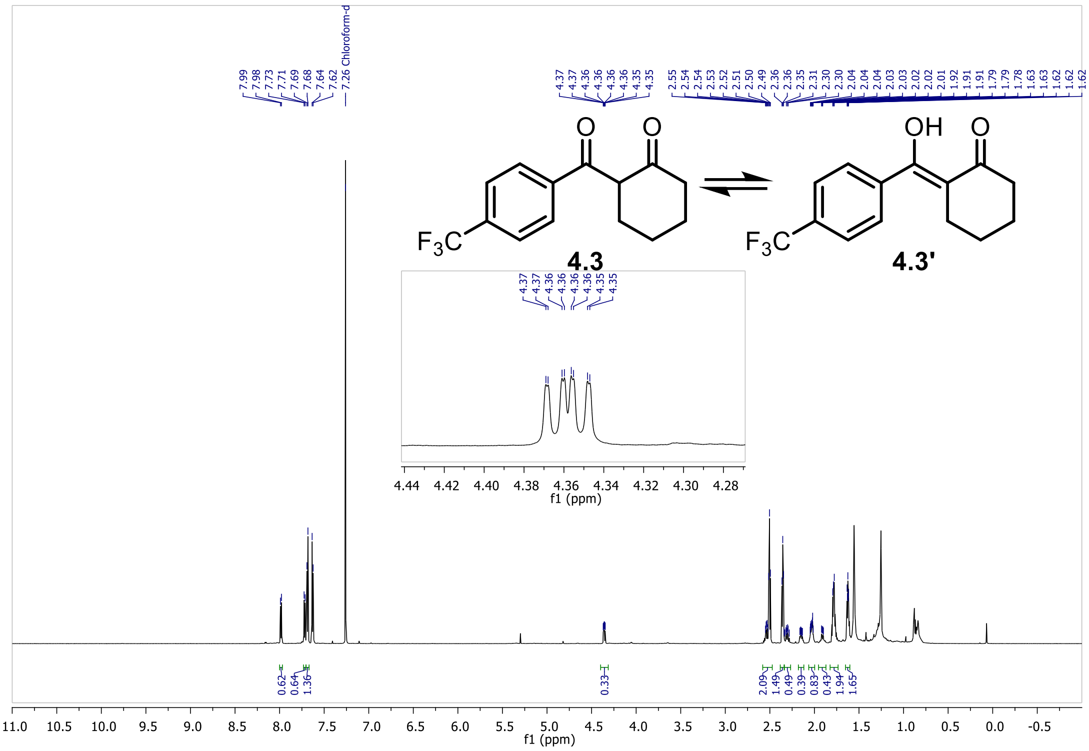

4.3/4.3' ^{13}C{^{1}H} NMR (176 MHz, $CDCl_3$)

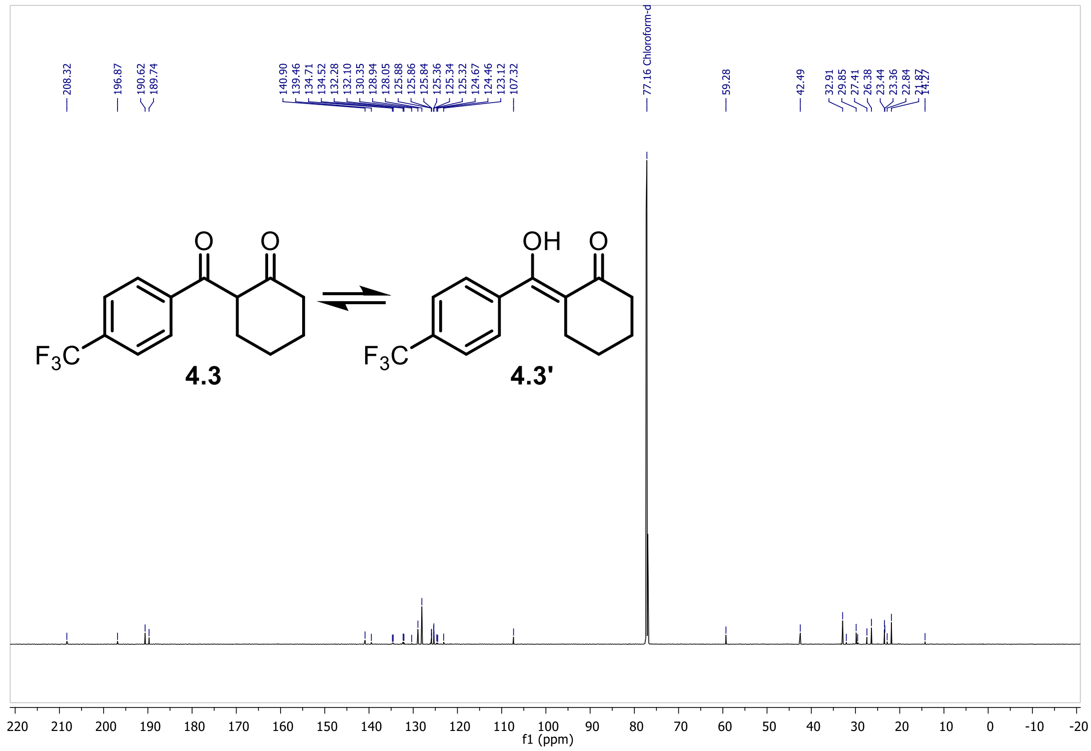

4.3/4.3' ^{19}F NMR (659 MHz, $CDCl_3$)

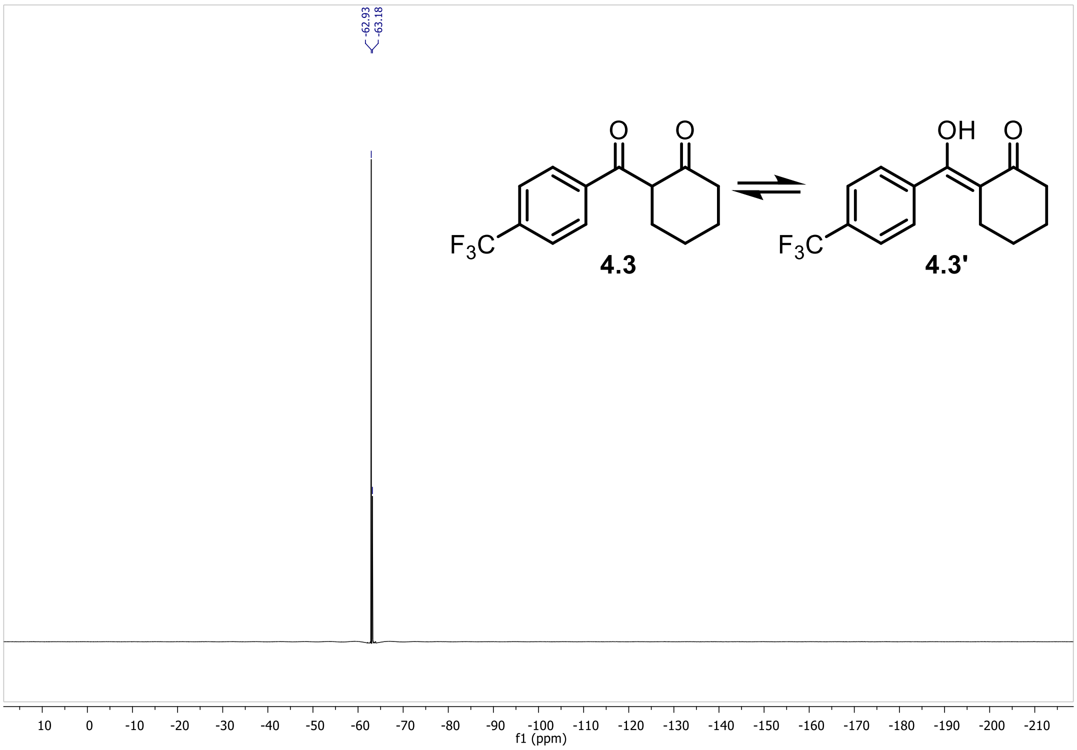

4.1/4.2 ^{1}H NMR (700 MHz, $CDCl_3$)

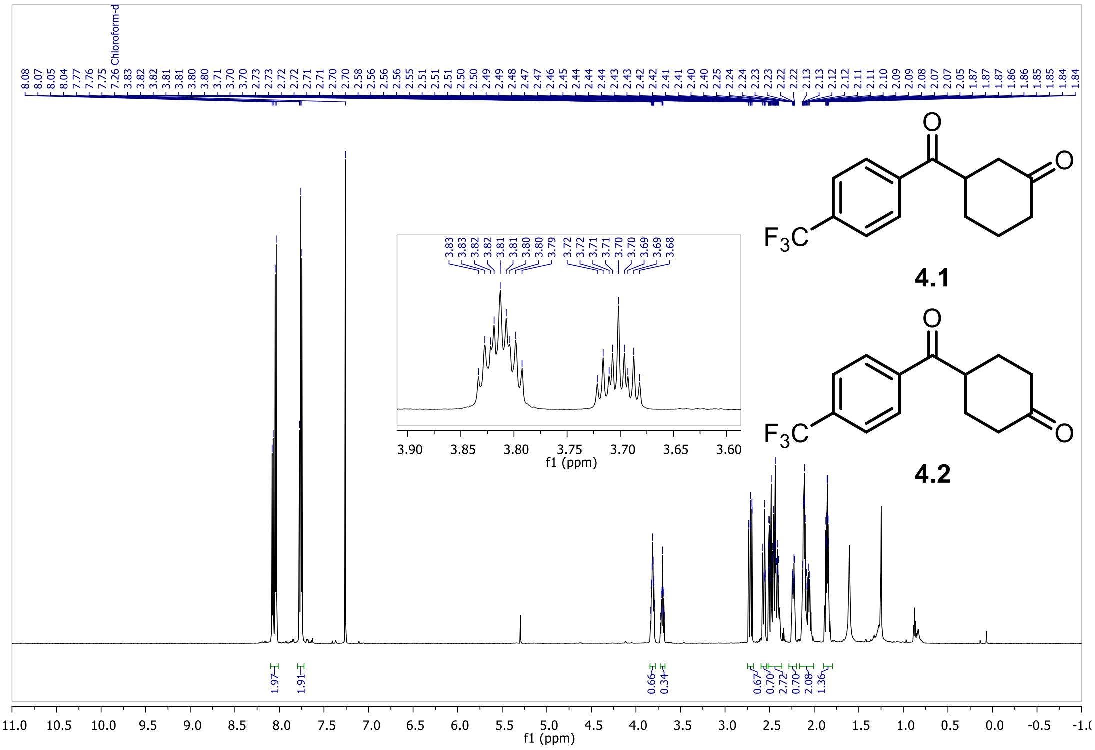

4.1/4.2 $^{13}C\{^{1}H\}$ NMR (176 MHz, $CDCl_3$)

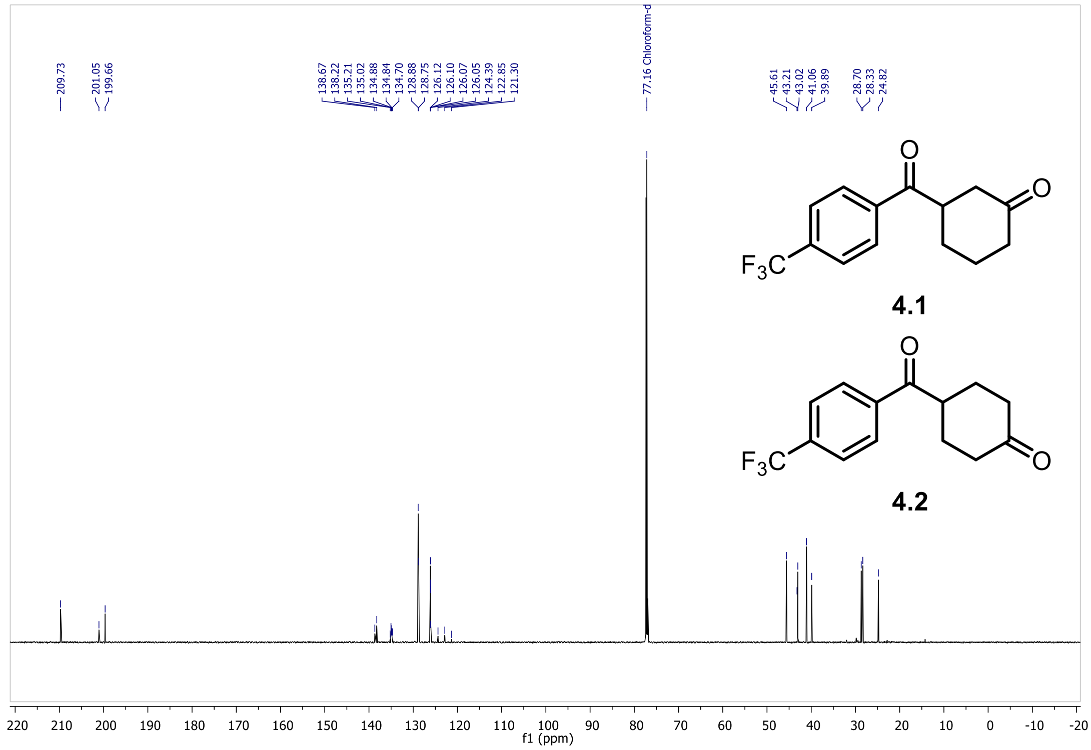

4.1/4.2 ^{19}F NMR (659 MHz, $CDCl_3$)

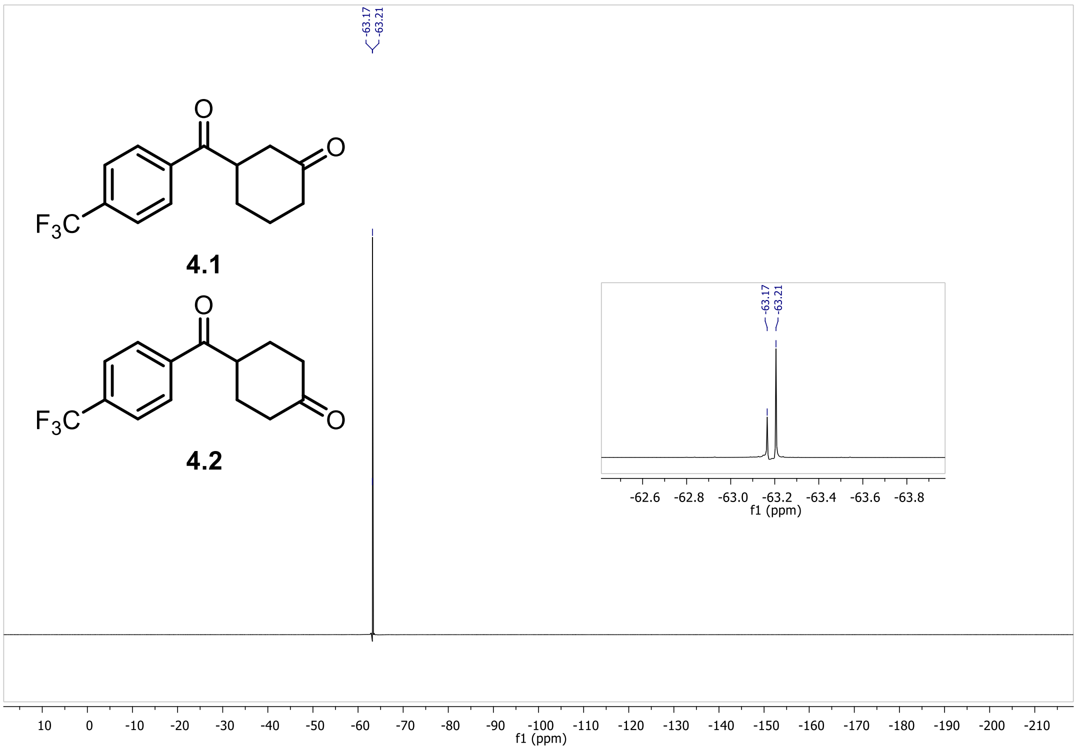

7 Mechanistic studies

7.1 Overview hydride shifts depending on ring size and substituent position

During our studies, we observed highly γ-selective oxidations for substrates derived from cyclopentane scaffolds (**I**). We attributed this regioselectivity, as well as the regioselectivity for the most substituted position, to formation of the stable 5-membered oxocarbenium ion **II'**.

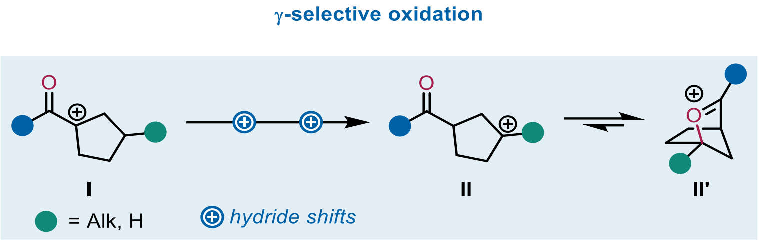

Figure S6. γ-selective oxidations I.

A similar pattern was observed for unsubstituted and γ-alkyl-substituted cyclohexane-derived substrates (**III**), where γ-oxidation was observed exclusively. While a further hydride shift would lead to a 6-membered oxocarbenium ion **V'**, such an event is disfavored by the required boat conformation of the intermediate (in addition to, for **IV**, the added penalty of transforming a 3° carbocation into a 2° cation). Therefore, only γ-oxidation is observed.

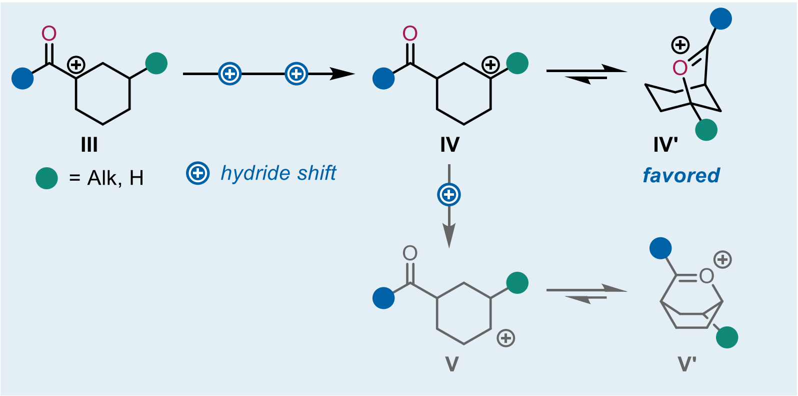

Figure S7. γ-selective oxidations II.

In the case of δ-alkyl-substituted cyclohexane substrates, the tertiary character of the oxocarbenium ion **VII’** seems to overcome the energetic penalty of adopting boat conformation, and oxidation is regioselective for the δ-position.

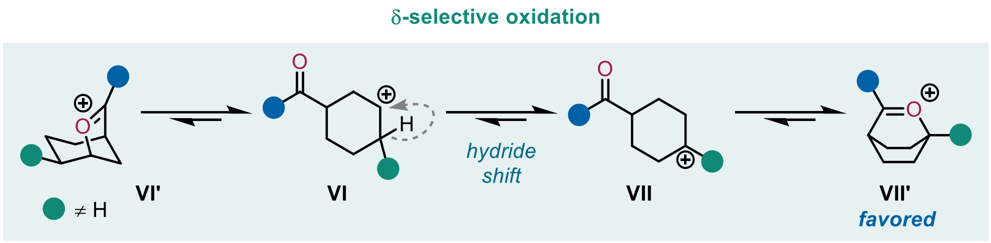

Figure S8. δ-selective oxidations.

Proposed Mechanism

We propose a mechanism starting with the formation of an α-keto carbocation **d**, generated from silyl enol ether **a** by reaction with $(PhI)_2O(SbF_6)_2$. This high-energy intermediate subsequently evolves towards a more stable carbocation through two successive hydride shifts. While all carbocations (**d**–**g**) are likely in equilibrium with the related oxocarbenium ion, only those providing 5- and 6-membered bridges (**f**→**f'** and **g**→**g'**) provide a suitable thermodynamic well. The results of the deuterium-labelling studies (*vide infra*) additionally suggest that, depending on the substitution pattern, these two relatively stable intermediates are in equilibrium and the system spontaneously evolves towards the most stable oxocarbenium ion, thus explaining the high degree of regioselectivity observed in the reaction. In fact, NMR experiments enabled direct observation of such a species as resting state of the reaction (see section 7.2 below).

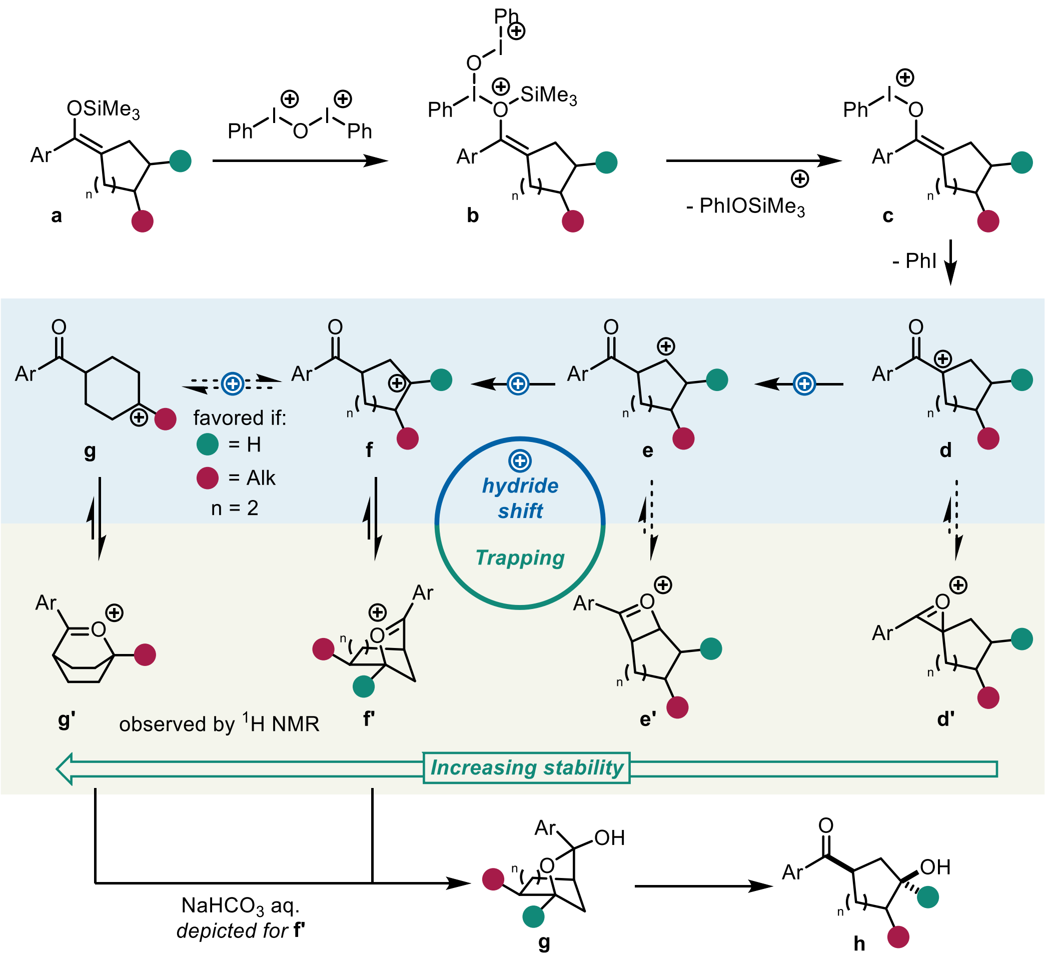

Figure S9. Proposed mechanism.

7.2 NMR studies

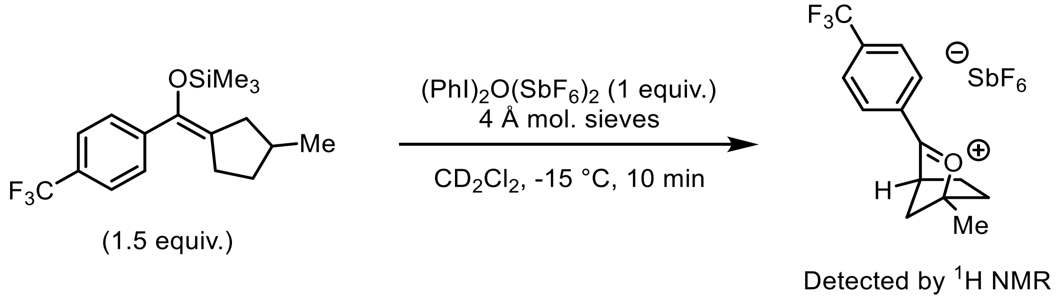

A Schlenk flask was charged with 4 Å molecular sieves (4-5 beads for 0.10 mmol of silyl enol ether), evacuated under high vacuum (*ca.* 10^{-2} bar), flame-dried and back-filled with argon. After cooling to -15 °C, $(PhI)_2O(SbF_6)_2$ (89.6 mg, 0.10 mmol, 1.00 equiv.) was added, followed by CD_2Cl_2 (1.00 mL, 0.1 M) and the resulting mixture was vigorously stirred for 5 min. A solution of **1r** in CD_2Cl_2 (0.15 M, 1.0 mL, 1.50 equiv.) was added dropwise and the mixture was left stirring at -15 °C for 10 min. After this time, 0.6 mL of the reaction mixture were transferred to an NMR tube which had been previously flushed with argon. The mixture was analyzed by ^{1}H NMR and ^{19}F NMR, to observe formation of the oxocarbenium ion. ^{13}C NMR spectra could not be recorded, as the oxocarbenium ion degrades upon standing for extended time at rt.

According to this experiment, the oxocarbenium ion is the primary product of the reaction and is stable under the reaction conditions. It can be observed with two characteristic signals (4.82 and 2.20 ppm) in ^{1}H NMR.

Reaction mixture ^{1}H NMR (400 MHz, CD_2Cl_2)

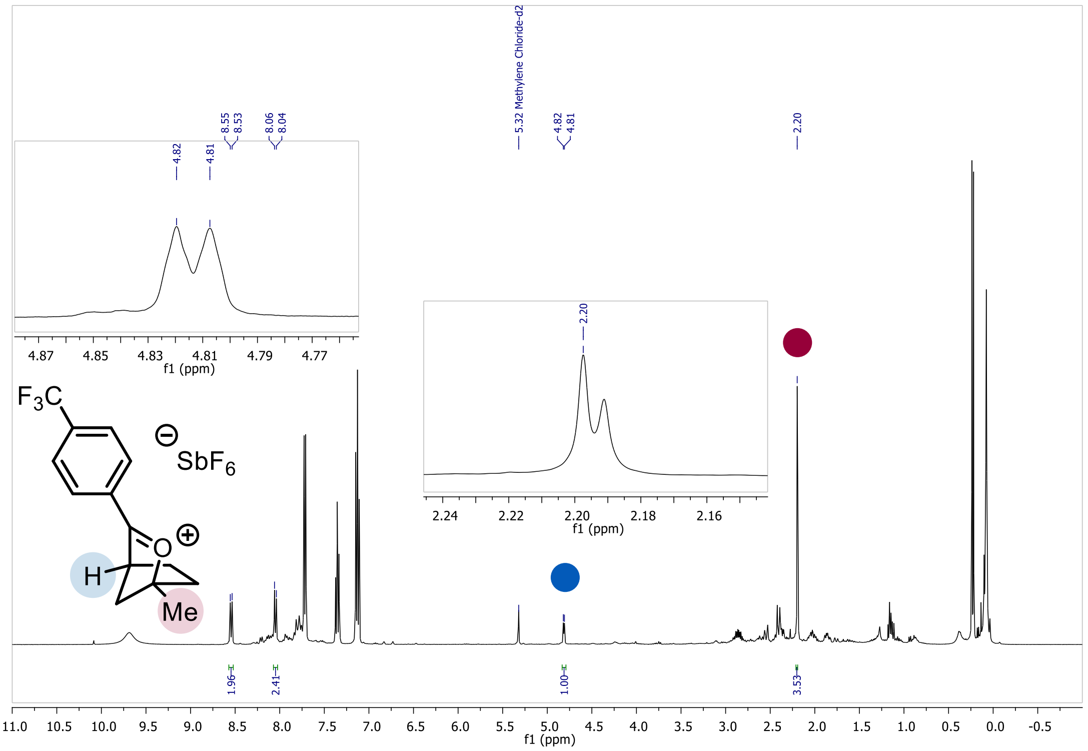

Reaction mixture ^{19}F NMR (376 MHz, CD_2Cl_2)

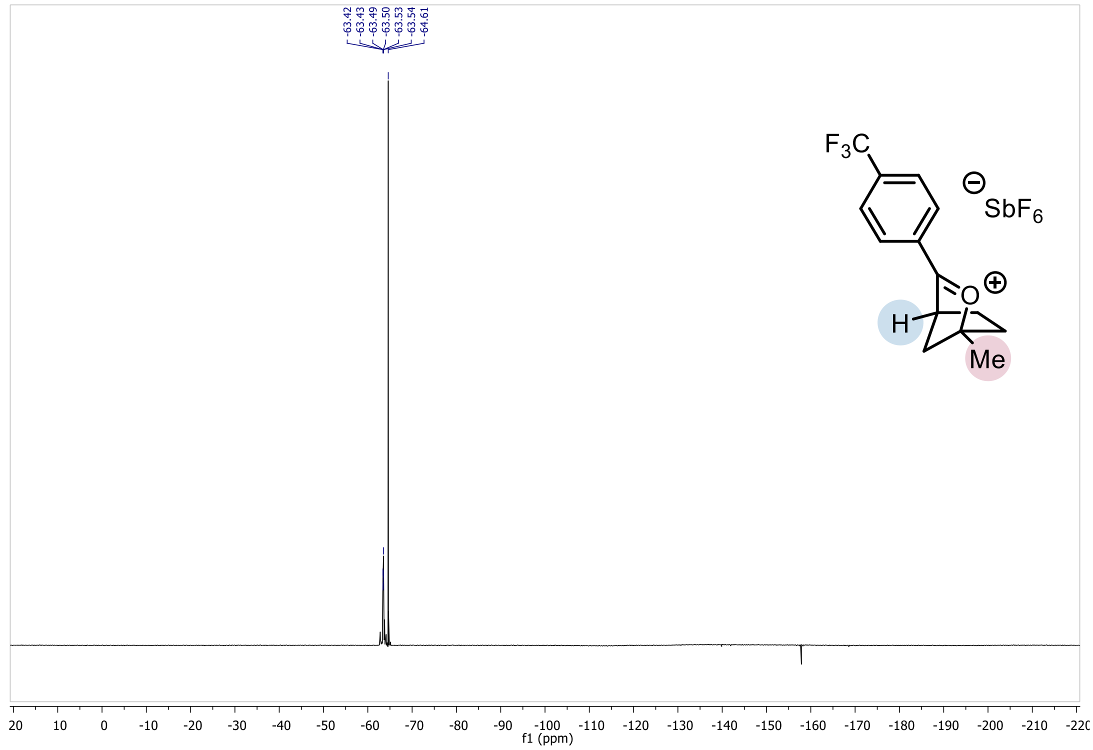

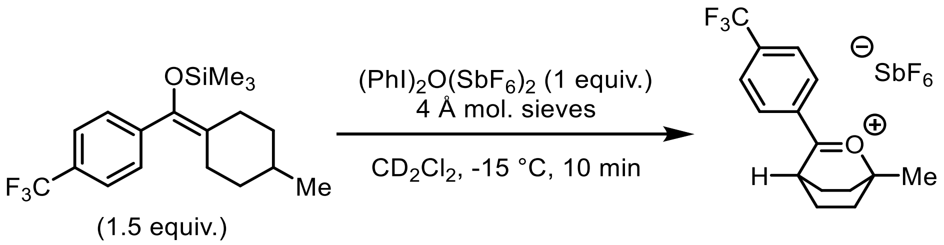

A Schlenk flask was charged with 4 Å molecular sieves (4-5 beads for 0.10 mmol of silyl enol ether), evacuated under high vacuum (*ca.* 10^{-2} bar), flame-dried and back-filled with argon. After cooling to -15 °C, $(PhI)_2O(SbF_6)_2$ (89.6 mg, 0.10 mmol, 1.0 equiv.) was added, followed by CD_2Cl_2 (1.00 mL, 0.1 M) and the resulting mixture was vigorously stirred for 5 min. A solution of **1w** in CD_2Cl_2 (0.15 M, 1.00 mL, 1.50 equiv.) was added dropwise and the mixture was left stirring at -15 °C for 10 min. After this time, 0.60 mL of the reacting mixture were transferred to an NMR tube previously flushed with argon. The mixture was analyzed by ^{1}H NMR and ^{19}F NMR, to observe formation of the oxocarbenium ion. ^{13}C NMR spectra could not be recorded as the oxocarbenium ion degrades upon standing for extended time at rt.

According to this experiment, the oxocarbenium ion is the primary product of the reaction and is stable under the reaction conditions. It can be observed with two characteristic signals (4.46 and 2.01 ppm) in ^{1}H NMR.

Reaction mixture ^{1}H NMR (400 MHz, CD_2Cl_2)

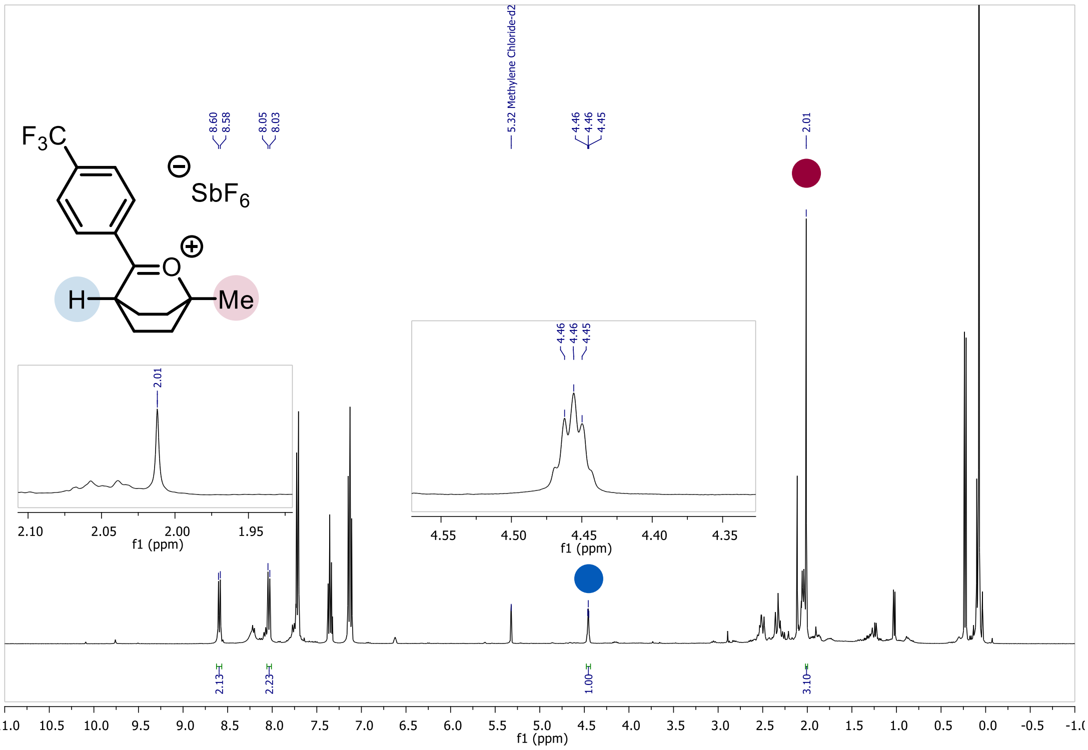

Reaction mixture ^{19}F NMR (376 MHz, CD_2Cl_2)

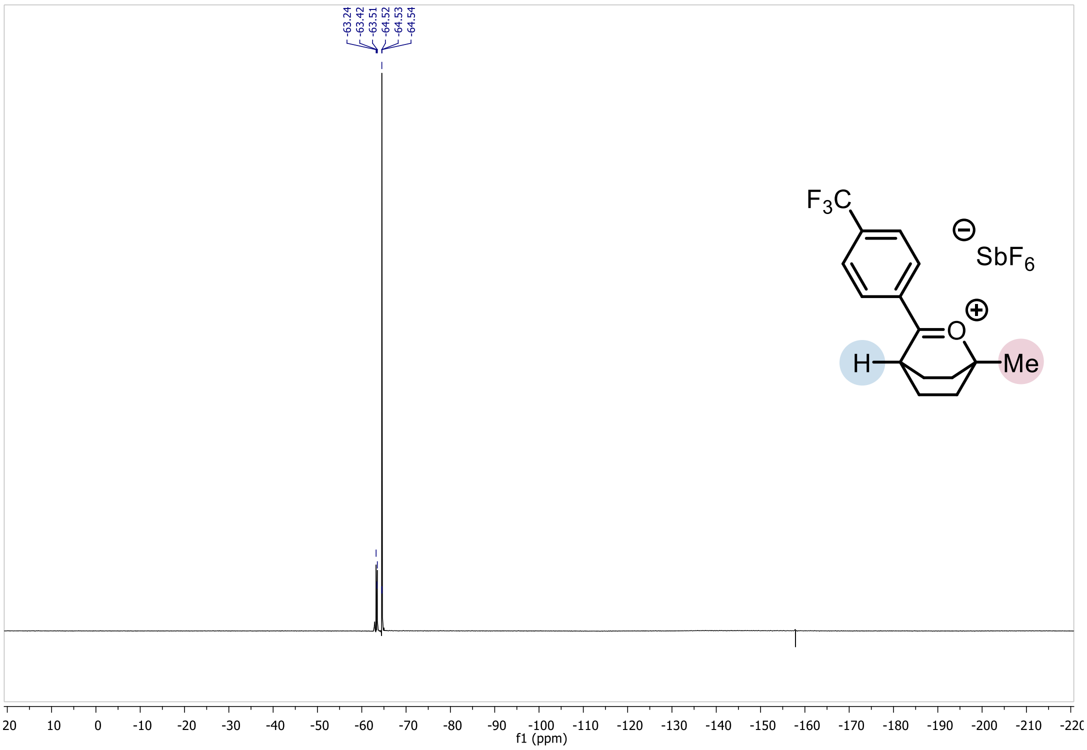

7.3 Deuterium-labelling studies

The deuterated substrates **1u** and **1v**, as well as the resulting remote oxidation products **2u** and **2v**, were prepared as described below.

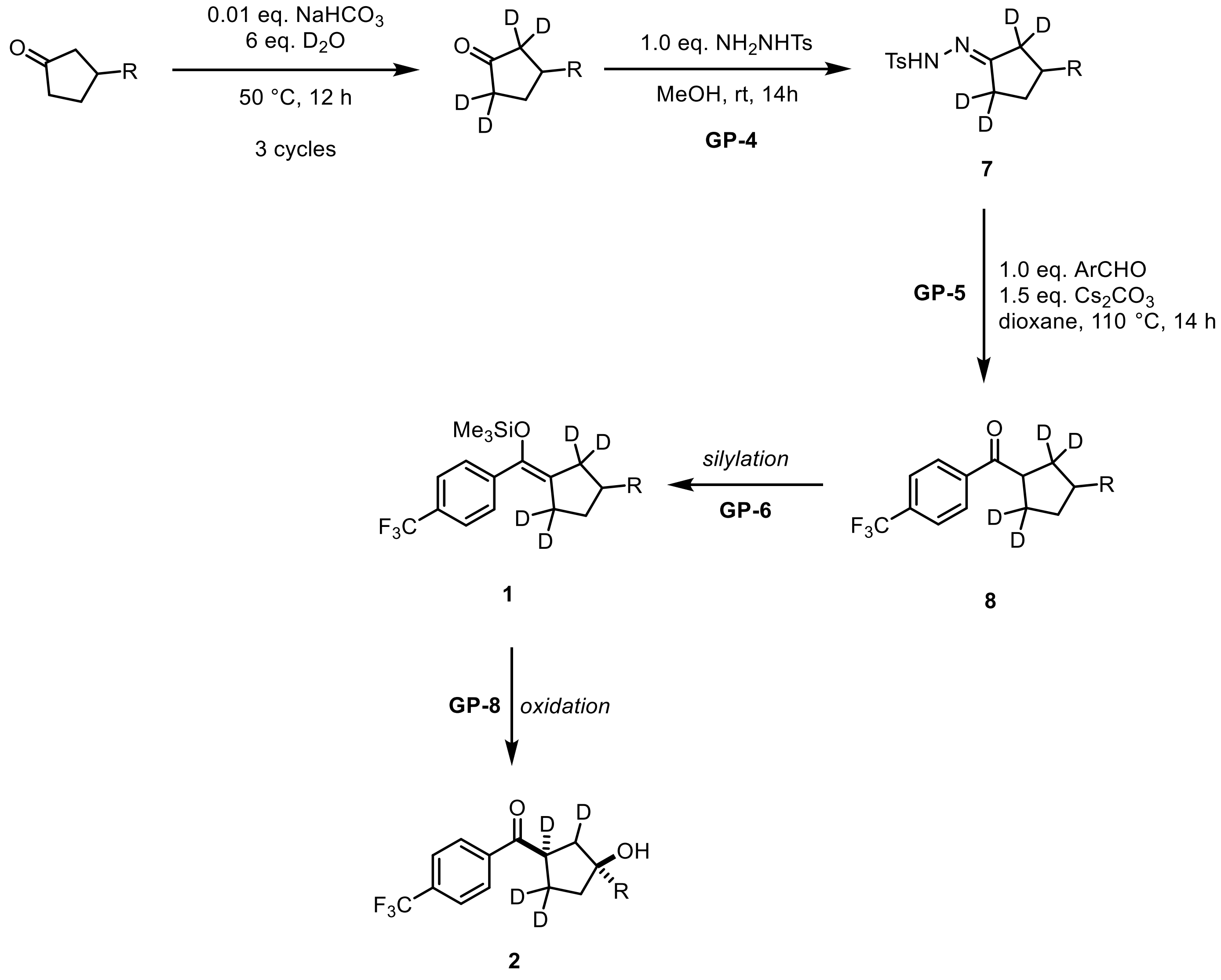

Figure S10. Synthetic route of deuterium-labelled substrates.

S3 – Cyclopentan-1-one-2,2,5,5-d_4

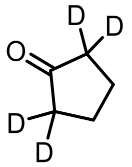

96% average incorporation

Formula: $C_5H_4D_4O$

MW: 88.1 g/mol

Synthesized following a modified literature procedure.[75] To an oven-dried vial charged with a magnetic stir bar, cyclopentanone (841 mg, 10.0 mmol, 1.00 equiv.), D_2O (1.09 mL, 60.0 mmol, 6.0 equiv.) and Na_2CO_3 (17.0 mg, 0.160 mmol, 0.160 equiv.) were added. The reaction mixture was stirred at 50 °C for 12 h. The organic layer was extracted with anhydrous diethyl ether (3 x 1 mL), and the organic layer was transferred to an oven-dried vial, dried with $MgSO_4$ and filtered over cotton to a second oven-dried vial. The ether solution was carefully evaporated at 800 mbar. An ^{1}H NMR sample was taken to check the total deuterium incorporation. After this, the same amount of Na_2CO_3 and D_2O as above was added to the ether solution and another 12afforded **S3** (700 mg, 5.24 mmol, 52%, 66% purity in ether) as a solution in ether. The highly volatile compound was used for the next step as a solution in diethyl ether and without further purification.

^{1}H NMR (400 MHz, $CDCl_3$): δ 1.94 (s, 4H).

S4 – 3-Methylcyclopentan-1-one-2,2,5,5-d_4

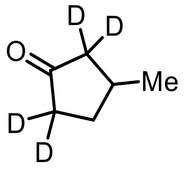

97% average incorporation

Formula: $C_6H_6D_4O$

MW: 102.1 g/mol

Synthesized following a modified literature procedure.[75] To an oven-dried vial charged with a magnetic stir bar, 3-methylcyclopentanone (981 mg, 10.0 mmol, 1.00 equiv.), D_2O (1.09 mL, 60.0 mmol, 6 equiv.) and Na_2CO_3 (17 mg, 0.16 mmol, 0.16 equiv) were added. The reaction mixture was stirred at 50 °C for 12 h. The organic layer was extracted with anhydrous diethyl ether (3 x 1 mL), and the organic layer was transferred to a dry vial, dried with $MgSO_4$ and filtered over cotton to a second oven-dried vial. The ether solution was carefully evaporated at 800 mbar. An ^{1}H NMR sample was taken to check the total deuterium incorporation. Afterwards, the same amount of Na_2CO_3 and D_2O as above was added to the ether solution and another 12afforded **S4** (537 mg, 5.25 mmol, 53%). The highly volatile compound was used for the next step as a solution in diethyl ether and without further purification.

^{1}H NMR (400 MHz, $CDCl_3$): δ 2.31 – 2.20 (m, 1H), 2.12 (dd, *J* = 12.5, 6.1 Hz, 1H), 1.54 – 1.41 (m, 1H), 1.12 (d, *J* = 6.6 Hz, 3H);

^{13}C NMR (101 MHz, $CDCl_3$): δ 208.8, 47.0 – 44.9 (m), 38.16 (dt, *J* = 39.6, 19.7 Hz), 31.7, 31.3, 20.4;

IR (neat) ν_{max}: 2919, 2180, 2164, 2052, 2005, 1691, 1265, 912, 736 cm^{-1};

MS (QTOF, EI$^+$, 70 eV): exact mass calculated for $[M]^+$ ($C_6H_6D_4O^+$) requires m/z 102.0977, found m/z 102.0974.

7i – *N'*-(cyclopentylidene-2,2,5,5-d_4)-4-methylbenzenesulfonohydrazide

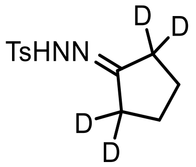

91% average incorporation

Formula: $C_{12}H_{13}D_4N_2O_2S$

MW: 256.4 g/mol

Synthesized following **GP-4**, using **S3** (700 mg, 5.24 mmol, 1.00 equiv., 66% purity in ether) and 4-methylbenzenesulfonylhydrazide (977 mg, 5.24 mmol, 1.00 equiv.). The resulting product **7i** (1.33 g, 5.19 mmol, 99%) was immediately used in the next step without further purification.

^{1}H NMR (400 MHz, CDCl$_3$): 7.85 (d, J = 8.3 Hz, 2H), 7.31 (d, J = 8.1 Hz, 2H), 7.02 (s, 1H), 2.43 (s, 3H), 1.79 (t, J = 6.5 Hz, 2H), 1.70 (q, J = 6.4 Hz, 2H);

7j – 4-Methyl-*N*'-(3-methylcyclopentylidene-2,2,5,5-d_4)benzenesulfonohydrazide

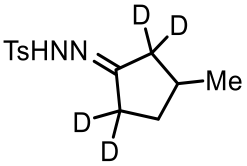

91% average incorporation

Formula: $C_{13}H_{15}D_4N_2O_2S$

MW: 270.4 g/mol

Synthesized following **GP-4**, using **S4** (537 mg, 5.25 mmol, 1.00 equiv.) and 4-methylbenzenesulfonylhydrazide (979 mg, 5.25 mmol, 1.00 equiv.). The resulting product **7j** (1.38 g, 5.09 mmol, 97%) was immediately used in the next step without further purification.

^{1}H NMR (400 MHz, $CDCl_3$): δ 7.84 (d, *J* = 8.3 Hz, 2H), 7.31 (d, *J* = 8.3 Hz, 2H), 6.94 (d, *J* = 12.1 Hz, 1H), 2.43 (s, 3H), 2.09 (ddd, *J* = 41.8, 14.5, 6.9 Hz, 1H), 1.92 (ddd, *J* = 28.5, 12.3, 6.0 Hz, 1H), 1.30 (dt, *J* = 21.2, 10.2 Hz, 1H), 1.02 (dd, *J* = 15.3, 6.5 Hz, 3H);

^{13}C NMR (176 MHz, $CDCl_3$): δ 158.2, 144.2, 135.7, 129.7, 128.2 (d, *J* = 2.5 Hz), 33.5 – 33.0 (m), 33.0 – 32.4 (m), 21.8, 20.4 (d, *J* = 9.0 Hz), 20.0 (d, *J* = 9.1 Hz), 19.5 (d, *J* = 8.1 Hz).

IR (neat) ν_{max}: 3215, 2955, 2926, 2869, 1656, 1598, 1494, 1455, 1402, 1336, 1308, 1291, 1231, 1185, 1164, 1092, 1070, 1046, 1019, 1004, 984, 909, 842, 814, 736 cm^{-1};

HRMS (ESI^{+}): exact mass calculated for $[M+Na]^+$ ($C_{13}H_{15}D_4N_2O_2SNa^+$) requires *m*/*z* 293.1232, found *m*/*z* 293.1224.

8d – (Cyclopentyl-2,2,5,5-d_4)(4-(trifluoromethyl)phenyl)methanone

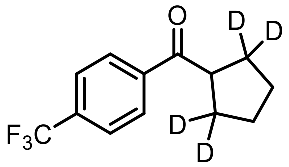

91% average incorporation

Formula: $C_{13}H_9D_4F_3O$

MW: 246.3 g/mol

Synthesized following **GP-5**, using **7i** (513 mg, 2.00 mmol, 1.00 equiv.), 4-(trifluoromethyl)benzaldehyde (348 mg, 2.00 mmol, 1.00 equiv.) and Cs_2CO_3 (977 mg, 3.00 mmol, 1.50 equiv.). The crude product was purified by flash column chromatography on silica gel (heptanes/EtOAc, 100:0 to 90:10) to afford **8d** (331 mg, 1.34 mmol, 67%) as a colorless oil.

Following column chromatography, **8d** was obtained contaminated with unreacted aldehyde and other undefined impurities of similar polarity. Despite these contaminants, **8d** was subjected to the subsequent step, silyl enol ether formation. The corresponding silyl enol ether, being less polar, was then easily separable from the aforementioned impurities due to the change in the relative polarities.

^{1}H NMR (400 MHz, CDCl$_3$): δ 8.07 (d, *J* = 8.1 Hz, 2H), 7.73 (d, *J* = 8.2 Hz, 2H), 3.68 (s, 1H), 1.78 – 1.63 (m, 4H);

^{13}C NMR (101 MHz, CDCl$_3$): δ 201.9, 139.8, 134.2 (q, *J* = 32.6 Hz), 128.9 (2C), 125.7 (q, *J* = 3.7 Hz, 2C), 123.8 (q, *J* = 272.6 Hz), 46.52 (s), 29.9 – 29.0 (m), 26.2;

^{19}F NMR (376 MHz, CDCl$_3$): δ -63.07.

IR (neat) ν_{max}: 2956, 2874, 2229, 2115, .1726, 1686, 1620, 1582, 1511, 1466, 1450, 1409, 1321, 1212, 1165, 1126, 1108, 1065, 1014, 980, 964, 909, 866, 850, 833, 781 cm^{-1};

MS (QTOF, EI+, 70 eV): exact mass calculated for $[M]^+$ ($C_{14}H_{11}D_4F_3O^+$) requires m/z 246.1164, found m/z 246.1158. *HRMS could not be recorded for this compound.*

8e – (3-Methylcyclopentyl-2,2,5,5-d_4)[4-(trifluoromethyl)phenyl]methanone

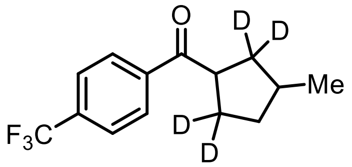

96% average incorporation

Formula: $C_{14}H_{11}D_4F_3O$

MW: 260.3 g/mol

Synthesized following **GP-5**, using **7j** (1081 mg, 4.00 mmol, 1.00 equiv.), 4-(trifluoromethyl)benzaldehyde (696 mg, 4.00 mmol, 1.00 equiv.) and Cs_2CO_3 (1.96 g, 6.00 mmol, 1.50 equiv.). The crude product was purified by flash column chromatography on silica gel (heptanes/EtOAc, 100:0 to 90:10) to afford **8e** (750 mg, 2.88 mmol, 72%) as a colorless oil.

Following column chromatography, **8e** was obtained contaminated with unreacted aldehyde and other undefined impurities of similar polarity. Despite these contaminants, **8e** was subjected to the subsequent step, silyl enol ether formation. The corresponding silyl enol ether, being less polar, was then easily separable from the aforementioned impurities due to the change in the relative polarities.

^{1}H NMR (400 MHz, CDCl$_3$): δ 8.05 (d, *J* = 8.4 Hz, 2H), 7.72 (d, *J* = 8.2 Hz, 2H), 3.75 (d, *J* = 25.5 Hz, 1H), 2.07 (dd, *J* = 14.3, 6.9 Hz, 1H), 1.87 (ddd, *J* = 19.3, 12.3, 6.6 Hz, 1H), 1.34 – 1.19 (m, 1H), 1.04 (t, *J* = 6.5 Hz, 3H);

^{13}C NMR (101 MHz, CDCl$_3$): δ [201.80 and 201.75], [139.83 and 139.68], 134.17 (q, *J* = 33.2 Hz), [128.95 and 128.90 (2C)], 125.7 (q, *J* = 3.5 Hz, 2C), 123.81 (dd, *J* = 272.7 Hz), [46.82 and 45.91], 38.3 – 36.9 (m), [35.70 and 35.08], [34.36 and 34.33], 29.2 – 28.0 (m), [20.16 and 19.85];

^{19}F NMR (377 MHz, CDCl$_3$) δ -63.07 (3F).

IR (neat) ν_{max}: 2954, 2870, 2219, 2119, 1686, 1620, 1581, 1510, 1456, 1409, 1378, 1320, 1213, 1166, 1126, 1110, 1065, 1034, 1015, 950, 934, 851, 769 cm^{-1};

MS (QTOF, EI+, 70 eV): exact mass calculated for $[M]^+$ ($C_{14}H_{11}D_4F_3O^+$) requires m/z 260.1321, found m/z 260.1316. *HRMS could not be recorded for this compound.*

1d – {(Cyclopentylidene-2,2,5,5-d_4)[4-(trifluoromethyl)phenyl]methoxy}trimethylsilane

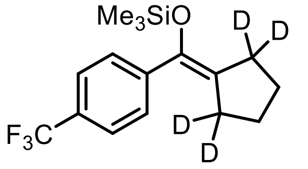

Formula: $C_{16}H_{17}D_4F_3OSi$

MW: 318.4 g/mol

Synthesized following **GP-6**, using **8d** (74 mg, 0.300 mmol, 1.00 equiv.), Me_3SiOTf (0.360 mL, 2.00 mmol, 1.50 equiv.) and Et_3N (0.420 mL, 3.00 mmol, 6.50 equiv.). The crude product was purified by flash column chromatography on silica gel (heptanes/EtOAc 100:0 to 95:5) to afford **1d** (82.0 mg, 0.260 mmol, 85%) as a colorless oil.

^{1}H NMR (700 MHz, CDCl$_3$): 7.56 (d, J = 8.3 Hz, 2H), 7.51 (d, J = 8.3 Hz, 2H), 1.73 – 1.62 (m, 4H), 0.06 (s, 9H);

^{13}C NMR (176 MHz, CDCl$_3$): δ 170.9 143.2, 140.0, 128.7 (q, J = 32.4 Hz), 127.7 (2C), 124.8 (q, J = 3.8 Hz, 2C), 124.4 (q, J = 271.9 Hz), 31.3 – 30.9 (m), 30.7 – 30.2 (m), 27.6, 25.7, 0.7 (3C);

^{19}F NMR (377 MHz, CDCl$_3$) δ -62.40 (3F);

IR (neat) ν_{max}: 2957, 2934, 2872, 2208, 2111, 1691, 1658, 1645, 1616, 1575, 1466, 1450, 1407, 1323, 1274, 1251, 1222, 1205, 1165, 1151, 1124, 1106, 1086, 1066, 1035, 1016, 979, 958, 912, 882, 866, 839, 794 cm^{-1};

MS (QTOF, EI$^+$, 70 eV): exact mass calculated for $[M]^+$ ($C_{16}H_{17}D_4F_3OSi^+$) requires m/z 318.1559, found m/z 318.1547.

1e – Trimethyl{(3-methylcyclopentylidene-2,2,5,5-d_4)[4-(trifluoromethyl)phenyl]-methoxy}silane

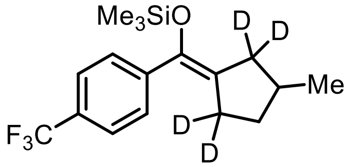

95% average incorporation

Formula: $C_{17}H_{19}D_4F_3OSi$

MW: 332.5 g/mol

Synthesized following **GP-6**, using **8e** (520 mg, 2.00 mmol, 1.00 equiv.), Me_3SiOTf (2.40 mL, 13.0 mmol, 1.50 equiv.) and Et_3N (2.80 mL, 20.0 mmol, 6.50 equiv.). The crude product was purified by flash column chromatography on silica gel (heptanes/EtOAc 100:0 to 95:5) to afford **1e** (543 mg, 1.63 mmol, 82%, *E*/*Z* = 1:1) as a colorless oil.

^{1}H NMR (400 MHz, $CDCl_3$): δ 7.56 (d, *J* = 8.5 Hz, 2H), 7.51 (dd, *J* = 8.5, 2.5 Hz, 2H), 2.10 – 1.92 (m, 1H), 1.90 – 1.81 (m, 1H), 1.25 (dd, *J* = 20.9, 9.1 Hz, 1H), 1.02 (dd, *J* = 23.0, 6.6 Hz, 3H), 0.06 (s, 9H);

^{13}C NMR (101 MHz, $CDCl_3$): δ 167.0, [143.21 and 143.12], [140.07 and 139.98], 128.49 (q, *J* = 18.4 Hz), [127.68 and 127.66 (2C)], 124.8 (q, *J* = 3.8 Hz, 2C), 124.20 (q, *J* = 271.8 Hz), 39.68 – 37.94 (m), [35.79 and 35.54], [33.86 and 33.74], 30.61 – 29.20 (m), [20.05 and 19.79], 0.7 (3C);

^{19}F NMR (377 MHz, $CDCl_3$) δ -62.40 (3F);

IR (neat) ν_{max}: 2954, 2870, 2203, 2113, 1688, 1653, 1616, 1574, 1515, 1456, 1407, 1376, 1323, 1301, 1280, 1265, 1252, 1215, 1161, 1124, 1106, 1067, 1044, 1016, 999, 876, 840, 750 cm^{-1};

MS (QTOF, EI+, 70 eV): exact mass calculated for $[M]^+$ ($C_{17}H_{19}D_4F_3OSi^+$) requires m/z 332.1716, found m/z 332.1724.

2d – *cis*-(3-Hydroxycyclopentyl-1,2,5,5-d_4)[4-(trifluoromethyl)phenyl]methanone and cis-(3-hydroxycyclopentyl-1,2,2,5-d_4)[4-(trifluoromethyl)phenyl]methanone

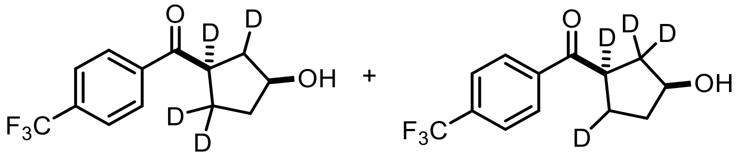

ratio = 53:47

Formula: $C_{13}H_9D_4F_3O_2$

MW: 262.3 g/mol

Synthesized following **GP-8**, using **1d** (31.8 mg, 0.100 mmol, 1.50 equiv.) and $(PhI)_2O(SbF_6)_2$ (59.7 mg, 66.7 µmol, 1 equiv.). Purification by flash column chromatography on silica gel (heptanes/EtOAc, 95:5 to 50:50) afforded the title compound (11.04 mg, 41.9 µmol, 41%) as a colorless solid.

^{1}H NMR (400 MHz, CDCl$_3$): δ 8.08 (d, *J* = 8.1 Hz, 2H), 7.75 (d, *J* = 8.2 Hz, 2H), 4.39 (t, *J* = 3.6 Hz, 1H), 2.69 (br s, 1H), 2.17 – 2.05 (m, 1H), 1.86 (dd, *J* = 11.4, 5.6 Hz, 2H);

^{13}C NMR (176 MHz, CDCl$_3$): δ [203.53 and 203.52], 139.0, 134.7 (q, *J* = 32.7 Hz), 129.1 (2C), 125.9 (q, *J* = 3.7 Hz, 2C), 123.7 (q, *J* = 272.7 Hz, 2C), [73.81 and 73.76], 44.8, 37.7 (ap t), [36.22, 36.13], 28.3 (ap t);

^{19}F NMR (376 MHz, CDCl$_3$) *δ* -63.15 (3F);

IR (neat) ν_{max}: 3518, 3363, 2940, 1689, 1409, 1328, 1311, 1271, 1241, 1169, 1129, 1111, 1067, 1016, 935, 910, 854, 769 cm^{-1};

HRMS (ESI$^+$): exact mass calculated for $[M+Na]^+$ ($C_{13}H_9D_4F_3O_2Na$) requires *m/z* 285.1011, found *m/z* 285.1016.

^{1}H NMR (400 MHz, CDCl$_3$):

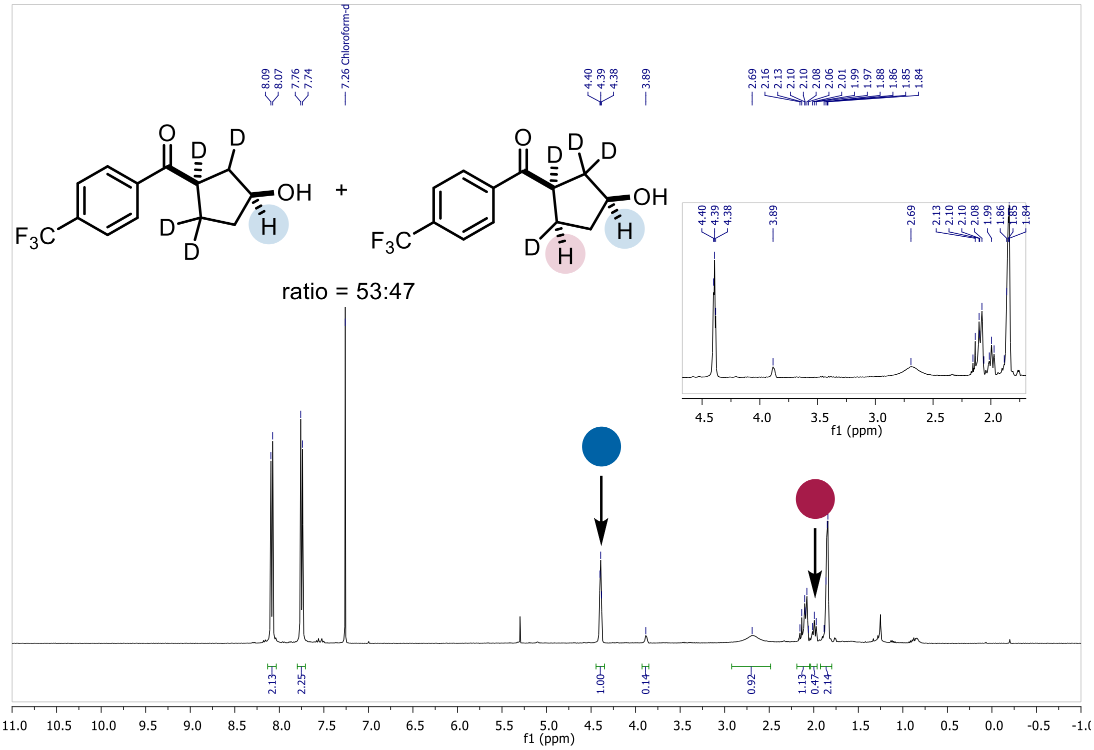

^{13}C (DEPT135) NMR (176 MHz, CDCl$_3$):

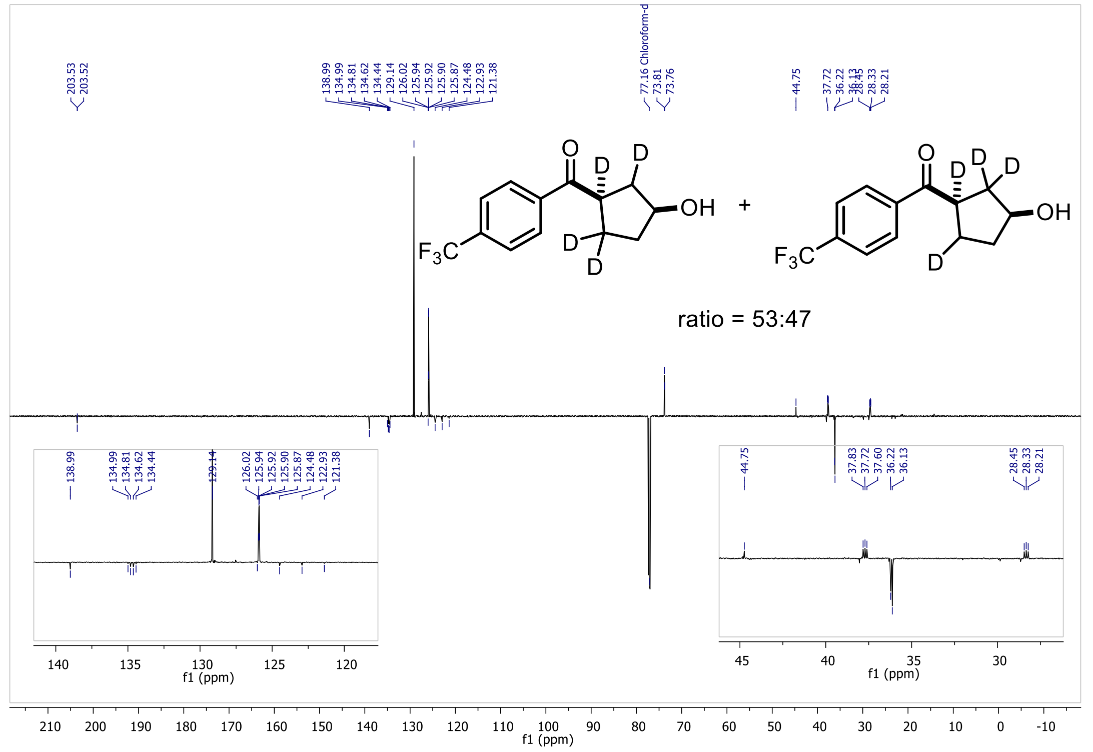

^{19}F NMR (377 MHz, $CDCl_3$):

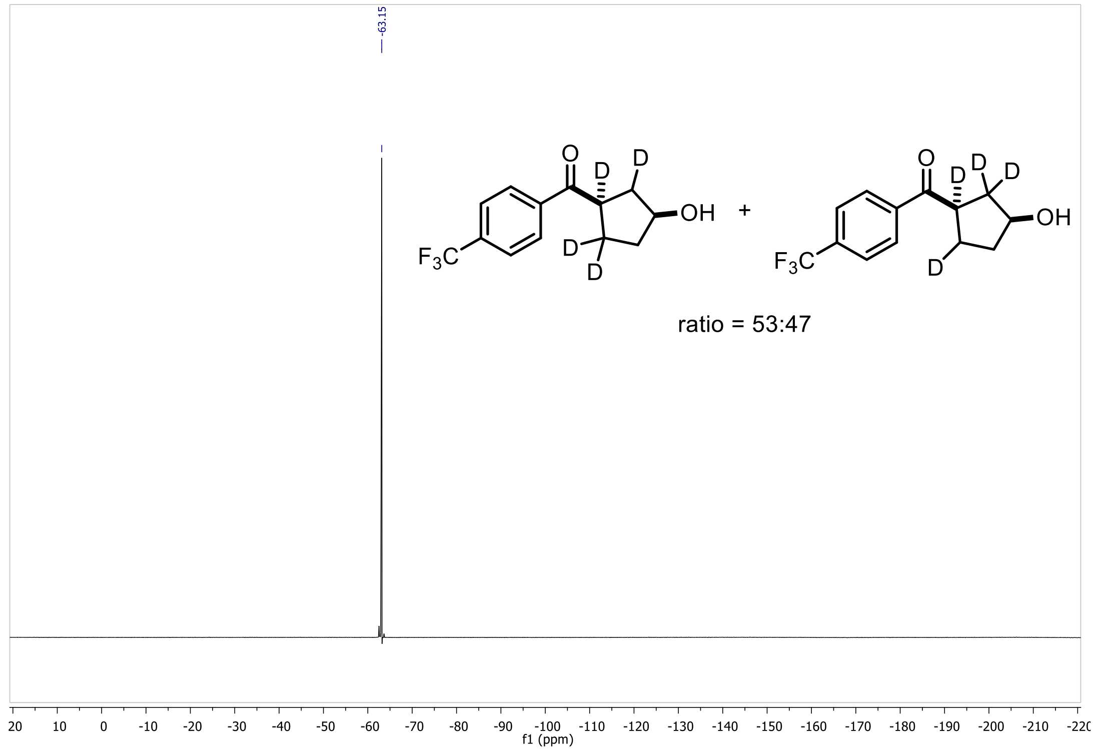

2e – *cis*-(3-Hydroxy-3-methylcyclopentyl-1,2,5,5-d_4)[4-(trifluoromethyl)phenyl]methan-one and (3-hydroxy-3-methylcyclopentyl-1,2,2,5-d_4)[4-(trifluoromethyl)phenyl]methanone

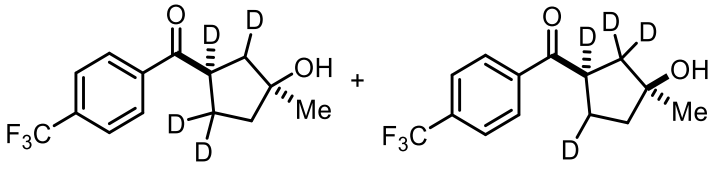

Formula: $C_{14}H_{11}D_4F_3O_2$

MW: 276.3 g/mol

Synthesized following **GP-8**, using **1e** (49.9 mg, 0.150 mmol, 1.50 equiv.) and $(PhI)_2O(SbF_6)_2$ (89.6 mg, 0.100 mmol, 1.00 equiv.). Purification by flash column chromatography on silica gel (heptanes/EtOAc, 95:5 to 50:50) afforded the title compound (25.7 mg, 93.0 µmol, 93%) as a colorless solid.

^{1}H NMR (600 MHz, CDCl$_3$): δ 8.07 (d, *J* = 8.1 Hz, 2H), 7.74 (d, *J* = 8.2 Hz, 2H), 2.95 (s, 1H), 2.11 (s, 0.53H), 2.04 (t, *J* = 8.9 Hz, 0.42H), 1.88 (dd, *J* = 12.9, 7.5 Hz, 1H), 1.72 (t, *J* = 11.3 Hz, 1H), 1.41 (s, 3H).

^{13}C NMR (151 MHz, CDCl$_3$): δ [203.77 and 203.74], 139.02, 134.72 (q, *J* = 32.7 Hz), 129.13, 125.89 (q, *J* = 3.4 Hz), 123.71 (q, *J* = 272.6 Hz), [79.62 and 79.57], 44.91 (q, *J* = 30.3 Hz), 43.23 – 42.83 (m), [41.80 and 41.70], 29.36 – 28.81 (m), [26.81 and 26.79];

^{19}F NMR (376 MHz, CDCl$_3$) *δ* -63.17 (3F);

IR (neat) ν_{max}: 3423, 3067, 2966, 2934, 2873, 2181, 1684, 1581, 1510, 1456, 1409, 1374, 1324, 1313, 1275, 1259, 1167, 1124, 1111, 1065, 1015, 978, 923, 900, 847, 772 cm^{-1};

HRMS (ESI$^+$): exact mass calculated for $[M+Na]^+$ ($C_{14}H_{11}D_4F_3O_2Na^+$) requires *m/z* 299.1167, found *m/z* 299.1165.

^{1}H NMR (600 MHz, CDCl$_3$):

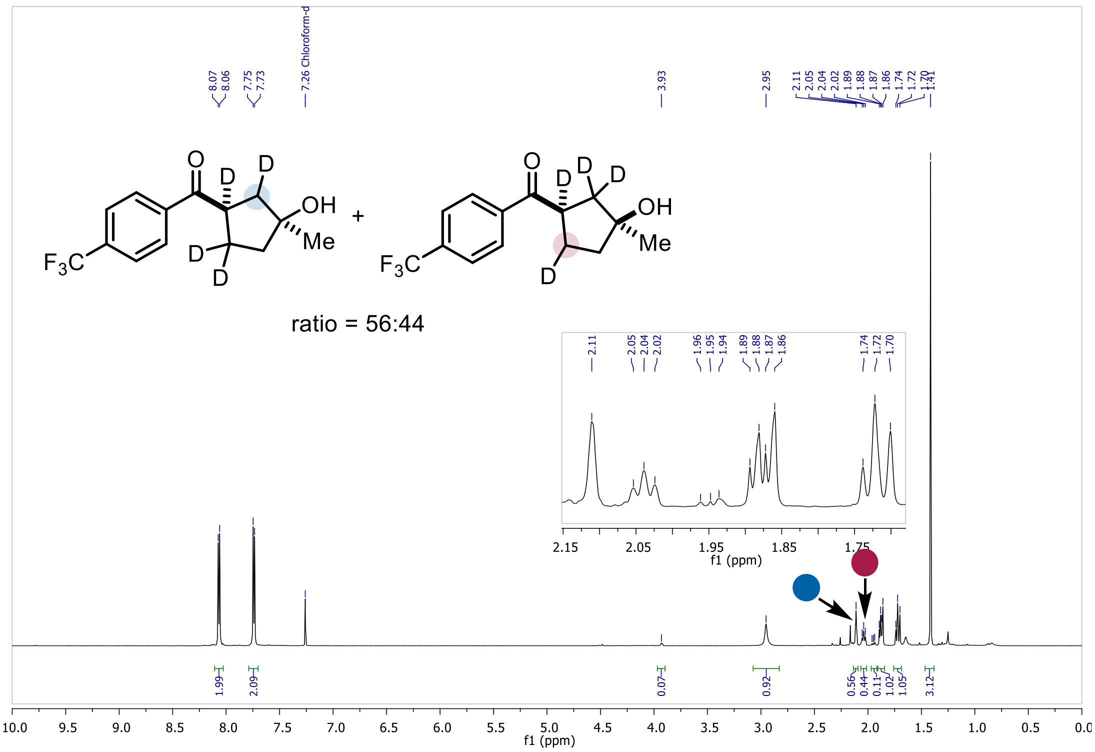

^{13}C{^{1}H} NMR (151 MHz, CDCl$_3$):

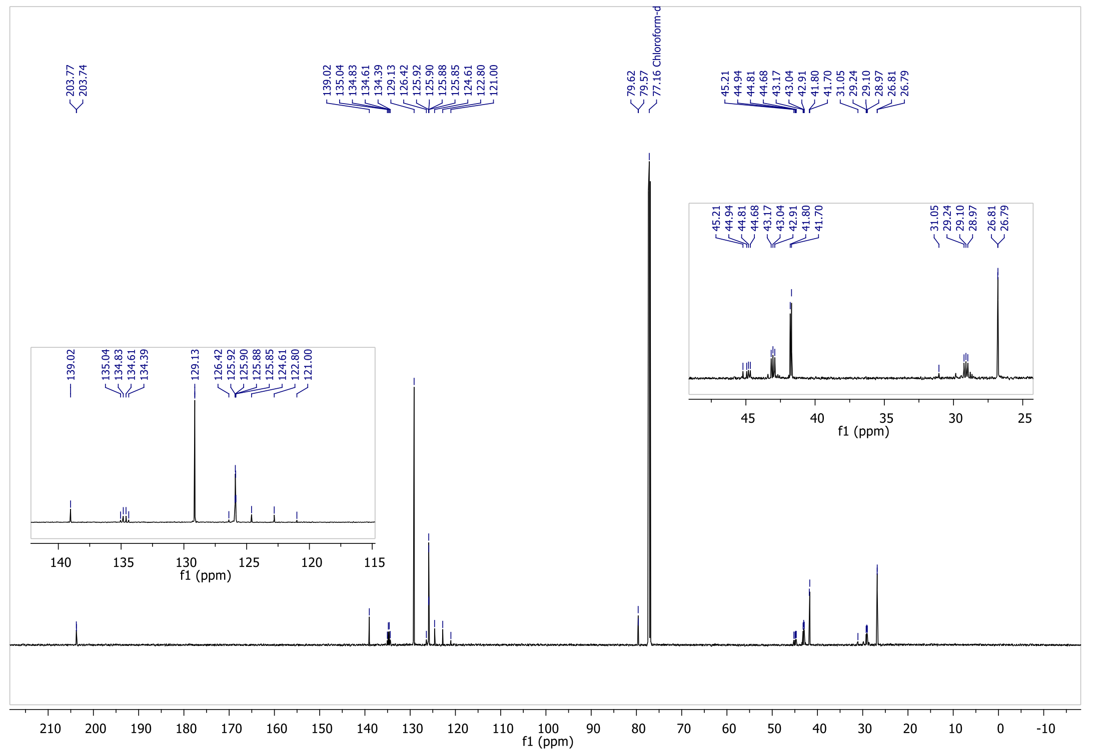

^{19}F NMR (377 MHz, $CDCl_3$):

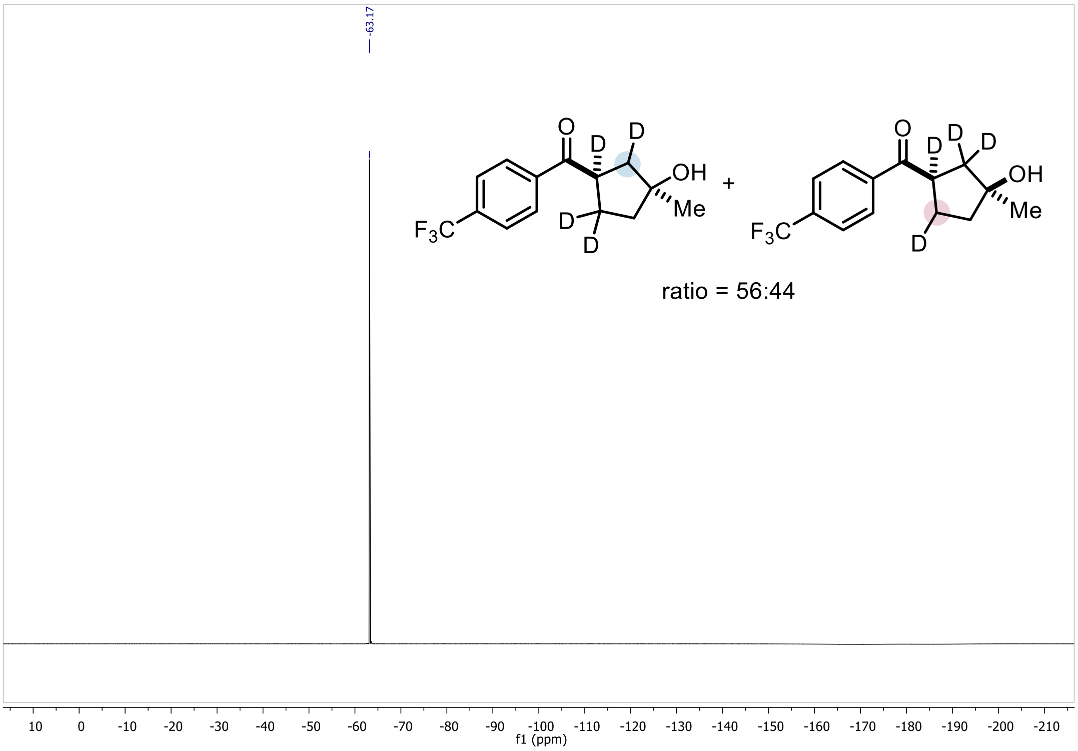

7.4 Limitations

Silyl ketene acetal: The use of a silyl ketene acetal resulted in poor yield, likely due to the low stability of the starting material, favoring hydrolysis and the increased basicity of the carbonyl.

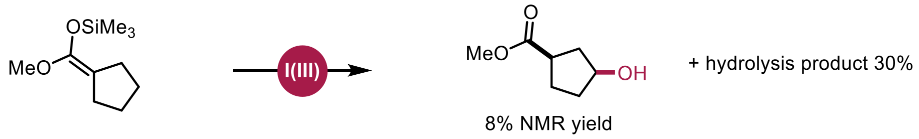

Silyl enol ether from aldehyde: The oxidation of silyl enol ether derived from aldehyde mostly lead to hydrolysis

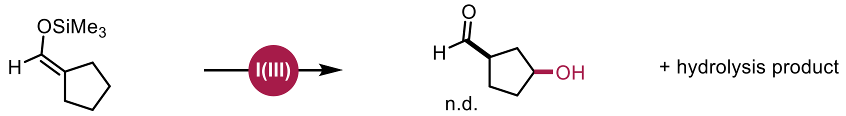

Silyl enol ethers from aliphatic ketones: The use of silyl enol ethers derived aliphatic ketones resulted in poor yields and impure products.

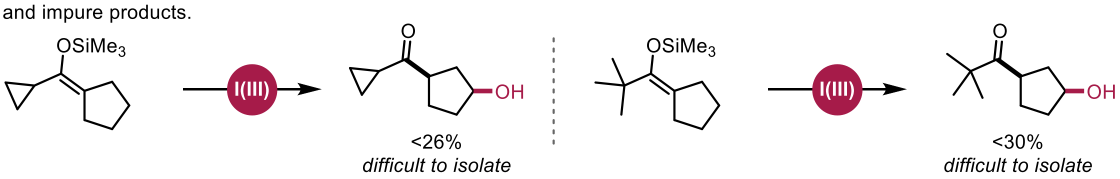

7-membered ring: Oxidation of a 7-membered ring resulted in poor yields and unspecific oxidation

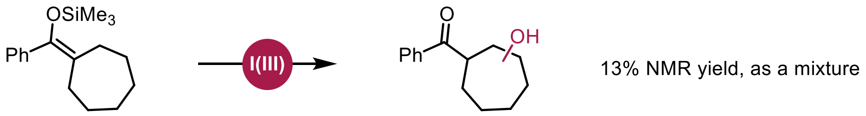

Electron-rich silyl enol ethers: Substrates with aryl group substituted by electron donating group give the targeted product in poor yield and the reaction crude is complex.

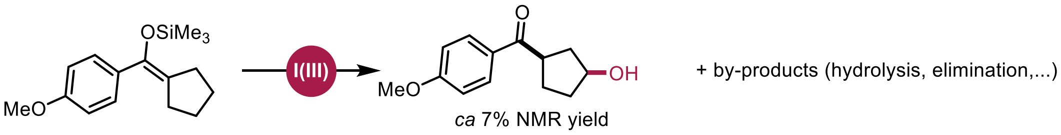

Non-cyclic substrates: Reaction with non-cyclic substrates resulted in poor yield and regioselectivity.

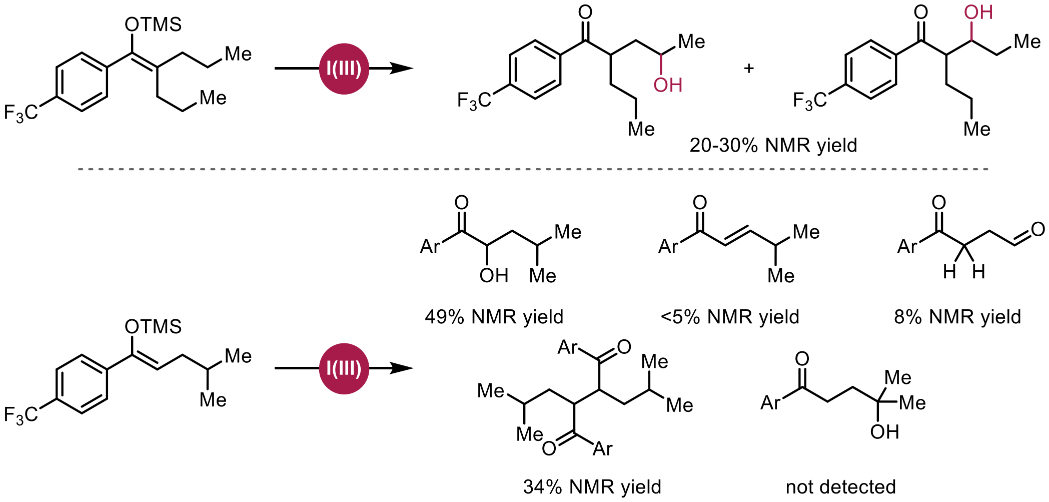

Ring expansion: Use of a linear substrate bearing a cyclic substituent yielded, among unidentifyable side products, a product resulting from ring expansion.

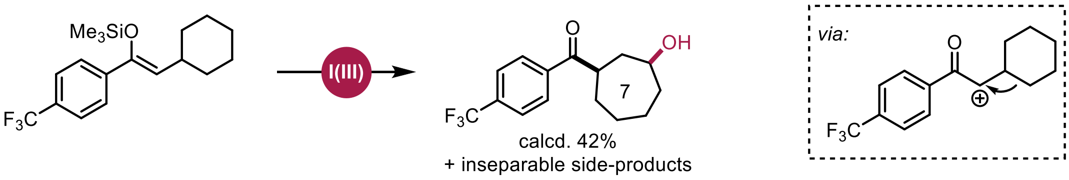

8 X-ray data

The X-ray intensity data were measured on STOE STADIVARI diffractometer equipped with multilayer monochromator, Mo K/α Primux 100 micro, micro focus sealed tube and Oxford cooling system. The structures were solved by *Intrinsic Phasing, Charge Flipping or Direct Methods*. Non-hydrogen atoms were refined with *anisotropic displacement parameters*. Hydrogen atoms were inserted at calculated positions and refined with riding model. The following software was used: X-Area Recipe,[76] *X-Area Pilatus3_SV*,[77] *OLEX2*,[78] for structure solution, refinement, molecular diagrams and graphical user-interface, *Shelxl*[79] for refinement and graphical user-interface *SHELXT-2015*[80] for structure solution, *SHELXL-2015*[80] for refinement*, Platon*[81] for symmetry check. Experimental data and CCDC-Codes Experimental data (Available online: http://www.ccdc.cam.ac.uk/conts/retrieving.html) can be found in **Table S2**. Crystal data, data collection parameters, and structure refinement details are reported in **Table S3** to **Table S5**. Structures, packing, interactions and data are visualized in **Figure S** to **Figure S**.

**Sample**	**Responsible for data evaluation**	**Machine**	**Source**	**Temp.**	**Detector Distance**	**Time/ Frame**	**#Frames**	**Frame width**	**CCDC**
				[K]	[mm]	[s]		[°]	
**2a**	A. Prado-Roller	Stoe	Cu	100	40	2	7732	0.5	2296101
**2c**	A. Prado-Roller	Stoe	Cu	100	40	6	18582	0.4	2296104
**2q**	A. Prado-Roller	Stoe	Mo	100	40	6	5468	0.5	2296102
**2t**	A. Prado-Roller	Stoe	Mo	100	40	1	2360	0.4	2296103

Table S2. Overview of samples analyzes by X-ray crystallography.

2a – *cis*-3-Hydroxy-3-methylcyclohexyl][4-(trifluoromethyl)phenyl]methanone

Crystal Data for $C_{15}H_{17.865}F_3O_{2.4325}$ (M =294.08 g/mol): tetragonal, space group P4$_2$ (no. 77), a = 14.2506(6) Å, c = 7.0976(4) Å, V = 1441.38(15) Å^3, Z = 4, T = 100 K, μ(Cu Kα) = 0.997 mm^{-1}, Dcalc = 1.355 g/cm^3, 14963 reflections measured (12.422° ≤ 2Θ ≤ 141.894°), 2553 unique (R_{int} = 0.0307, R_{sigma} = 0.0290) which were used in all calculations. The final R_1 was 0.0390 (I > 2σ(I)) and wR_2 was 0.0959 (all data).

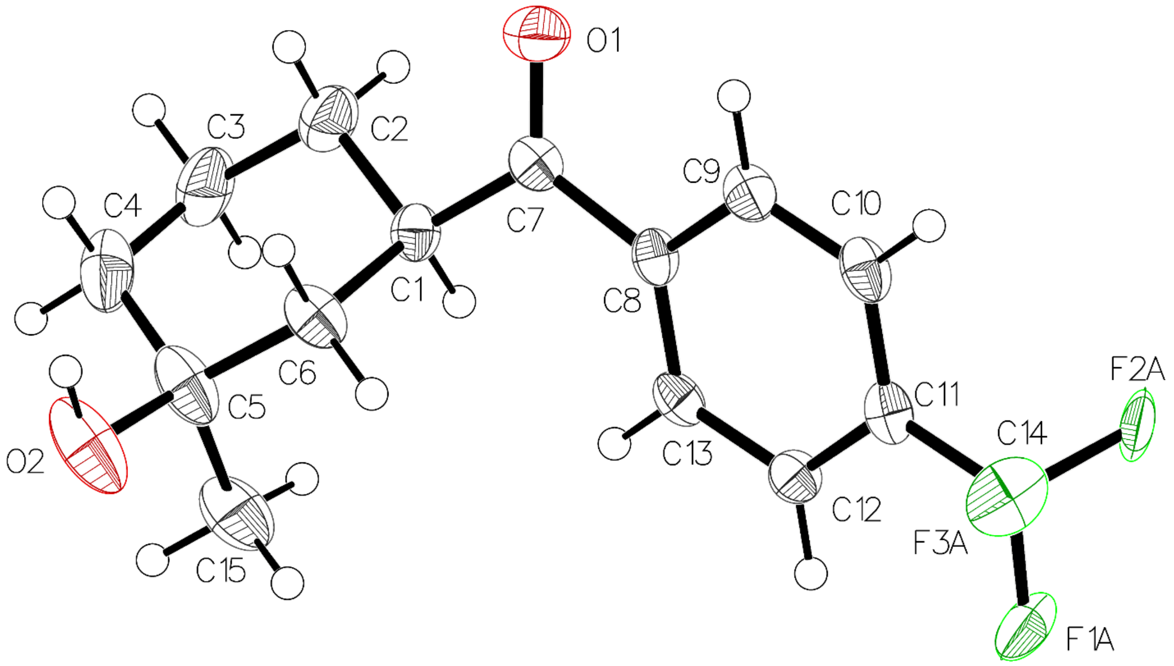

Figure S14. Crystal structure drawn with 50% displacement ellipsoid. The bond precision for C–C single bonds is 0.0041 Å. The chiral orientation of C1 and C5 is proofed by Parson's Flack and the Hooft Parameter (0.03(17),-0.12(5)). The CF_3 group is rotating almost freely and therefore disordered, visualized in **Figure S15**. A SUMP command refined the three split groups for the F atoms.

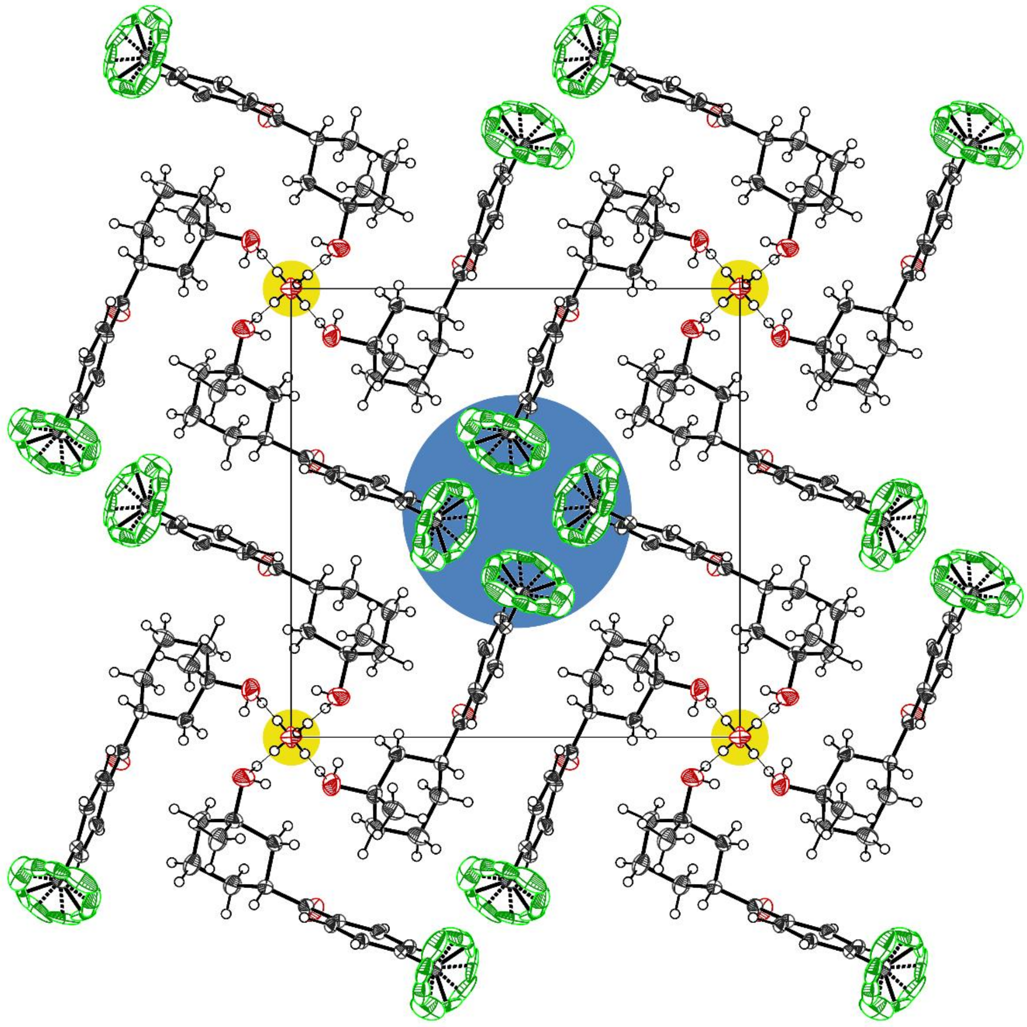

Figure S15. The packing exposes the orientation along the c axis. Co-crystallized water (yellow shaded) as well as the CF_3 (blue shaded) groups form tubes along the c axis. The co-crystallized water is not fully occupied (~86.5%) which is in good accordance to the fact that HPLC grade solvents were used during crystallization. The cases without water seem to orientate a weak hydrogen bond from O2 to the neighboring symmetry equivalent of O2.

Identification code	**2a**
Empirical formula	$C_{15}H_{17.86}F_3O_{2.43}$
Formula weight	294.08
Temperature/K	100
Crystal system	tetragonal
Space group	$P4_2$
a/Å	14.2506(6)
b/Å	14.2506(6)
c/Å	7.0976(4)
α/°	90
β/°	90
γ/°	90
Volume/Å^3	1441.38(15)
Z	4
ρ_{calc}g/cm^3	1.355
μ/mm^{-1}	0.997
F(000)	617.0
Crystal size/mm^3	0.32 × 0.14 × 0.03
Radiation	Cu Kα (λ = 1.54186)
2Θ range for data collection/°	12.422 to 141.894
Index ranges	-17 ≤ h ≤ 13, -11 ≤ k ≤ 17, -7 ≤ l ≤ 8
Reflections collected	14963
Independent reflections	2553 [R_{int} = 0.0307, R_{sigma} = 0.0290]
Data/restraints/parameters	2553/149/253
Goodness-of-fit on F^2	0.995
Final R indexes [I>=2σ (I)]	R_1 = 0.0390, wR_2 = 0.0904
Final R indexes [all data]	R_1 = 0.0524, wR_2 = 0.0959
Largest diff. peak/hole / e Å^{-3}	0.35/-0.25
Flack parameter	0.03(17)

Table S3. Sample and crystal data, data collection and structure refinement. Detailed information listed in the CIF code of CCDC: 2296101.

2c – *cis*-(3-Hydroxy-3,5,5-trimethylcyclohexyl)[4-(trifluoromethyl)phenyl]methanone

Crystal Data for $C_{17}H_{21}F_3O_2$ (M =314.34 g/mol): monoclinic, space group C2 (no. 5), a = 82.518(2) Å, b = 6.43340(10) Å, c = 37.6739(11) Å, β = 108.891(2)°, V = 18922.7(8) Å^3, Z = 48, T = 100 K, μ(Cu Kα) = 0.924 mm^{-1}, Dcalc = 1.324 g/cm^3, 154585 reflections measured (6.996° ≤ 2Θ ≤ 144.03°), 33676 unique (R_{int} = 0.0350, R_{sigma} = 0.0354) which were used in all calculations. The final R_1 was 0.0701 (I > 2σ(I)) and wR_2 was 0.2125 (all data).

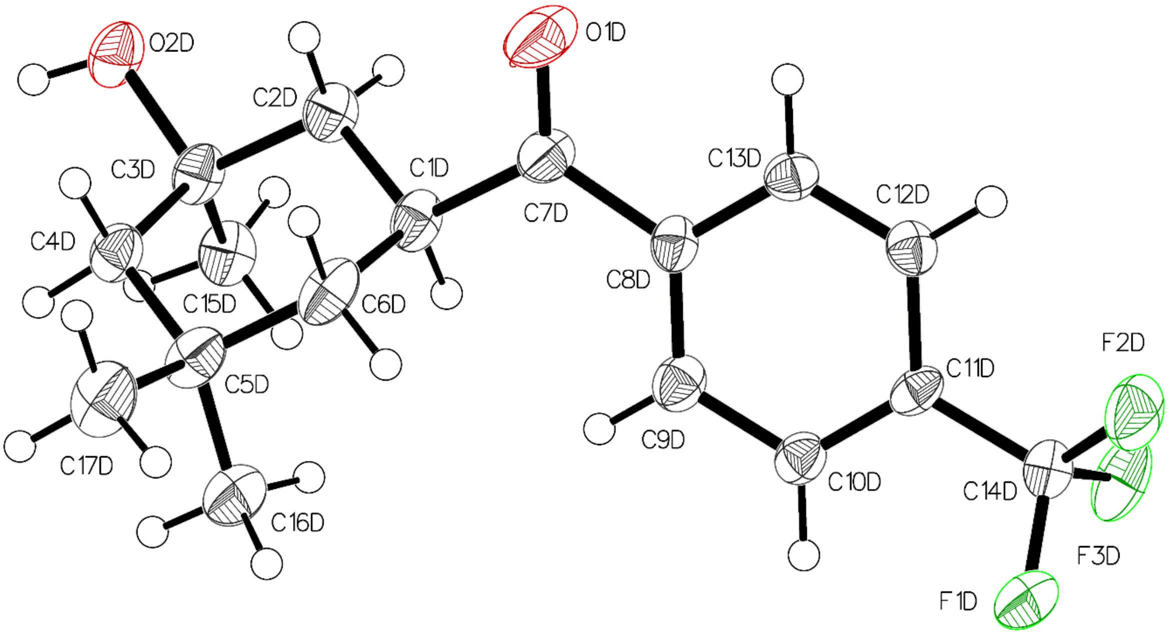

Figure S16. Crystal structure of one molecular unit in the asymmetric unit drawn with 50% displacement ellipsoid. The bond precision for C–C single bonds is 0.01215Å. Chiral positions are at C1 and C3.

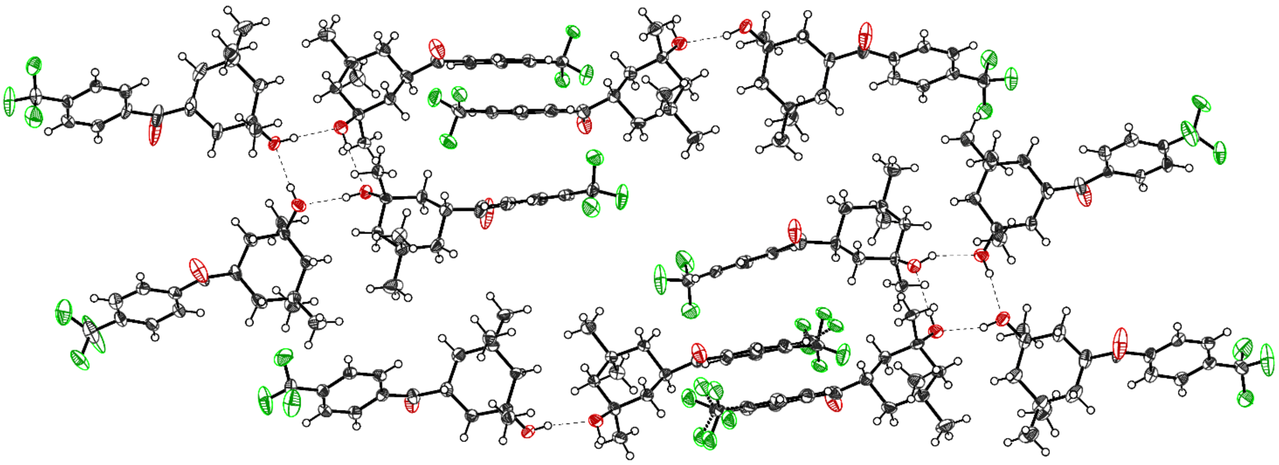

Figure S17. The very uncommon high number of 12 molecular units build the asymmetric unit. This force, for organic small molecules, a big unit cell in the monoclinic, chiral space group C2 of a=82.518, b=6.4334, c=37.6739, Beta=108.891. Surprised by the big cell two B-Alerts were not to avoid. The Low C–C bond precision of 0.01215 Ang. and 13 missing FCF reflections, which were cut at 17 degrees in two Theta because of issues in I/sigma (see **Figure S**). Even if the space group C2 is chiral, both chiral molecular species are part of the asymmetric unit (chiral parameters therefore are not listed). Both chiral setups are 6 times in the asymmetric unit and underpin the racemic form of the molecule. All single molecular units connect via hydrogen bonds to three neighboring systems. This is also valid to the groups, which connect to only one group in the asymmetric unit. These molecules connect via symmetry operations and form the very similar rhombic geometry as in the others.

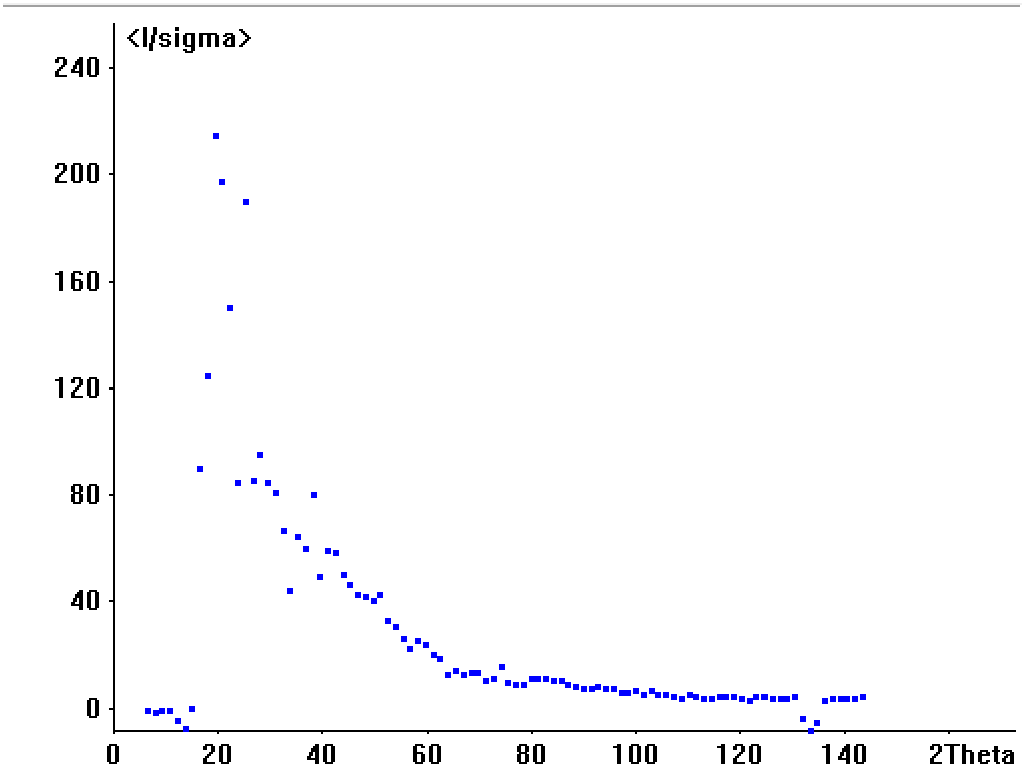

Figure S18. Two Theta values below 17 degrees are not used for data refinement.

Identification code	**2c**
Empirical formula	$C_{17}H_{21}F_3O_2$
Formula weight	314.34
Temperature/K	100
Crystal system	monoclinic
Space group	C2
a/Å	82.518(2)
b/Å	6.43340(10)
c/Å	37.6739(11)
α/°	90
β/°	108.891(2)
γ/°	90
Volume/Å^3	18922.7(8)
Z	48
ρ_{calc}g/cm^3	1.324
μ/mm^{-1}	0.924
F(000)	7968.0
Crystal size/mm^3	0.32 × 0.167 × 0.08
Radiation	Cu Kα (λ = 1.54186)
2Θ range for data collection/°	6.996 to 144.03
Index ranges	-100 ≤ h ≤ 67, -7 ≤ k ≤ 7, -46 ≤ l ≤ 37
Reflections collected	154585
Independent reflections	33676 [R_{int} = 0.0350, R_{sigma} = 0.0354]
Data/restraints/parameters	33676/34/2445
Goodness-of-fit on F^2	1.021
Final R indexes [I>=2σ (I)]	R_1 = 0.0701, wR_2 = 0.1763
Final R indexes [all data]	R_1 = 0.1094, wR_2 = 0.2125
Largest diff. peak/hole / e Å^{-3}	0.61/-0.55
Flack parameter	0.1(2)

Table S4. Sample and crystal data, data collection and structure refinement. Detailed information listed in the CIF code of CCDC: 2296104

2q – *cis*-(3-(*tert*-Butyl)-3-hydroxycyclopentyl)[4-(trifluoromethyl)phenyl]methanone

Crystal Data for $C_{17}H_{21}O_2F_3$ (M =314.34 g/mol): monoclinic, space group P2$_1$/c (no. 14), a = 13.750(3) Å, b = 10.610(2) Å, c = 11.460(2) Å, β = 113.10(3)°, V = 1537.8(6) Å^3, Z = 4, T = 100 K, μ(Mo Kα) = 0.111 mm^{-1}, Dcalc = 1.358 g/cm^3, 59408 reflections measured (7.06° ≤ 2Θ ≤ 60.068°), 4476 unique (R_{int} = 0.0712, R_{sigma} = 0.0907) which were used in all calculations. The final R_1 was 0.0401 (I > 2σ(I)) and wR_2 was 0.0895 (all data).

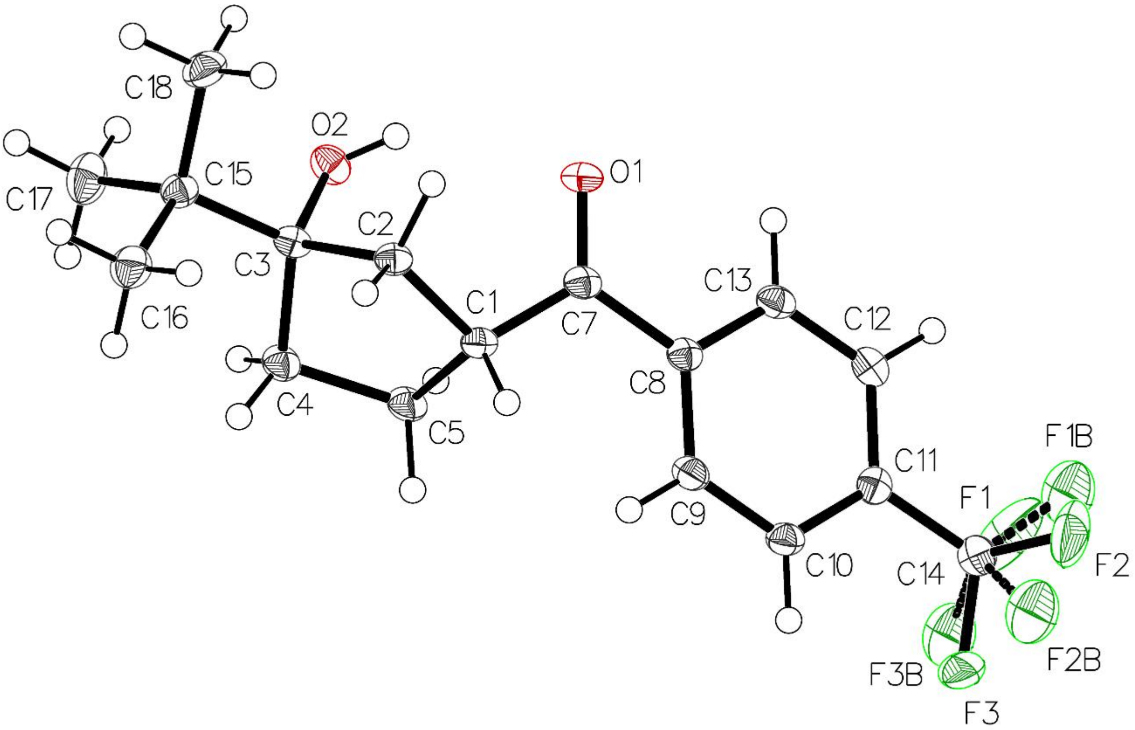

Figure S11. Crystal structure drawn with 50% displacement ellipsoid. The bond precision for C–C single bonds is 0.0018Å. Chiral positions are at C1 and C3. The centrosymmetric space group underpins the existence of the racemic form of the molecule. The CF_3 group is rotating and split into two disordered groups. The packing is unobtrusive and therefore not illustrated.

Identification code	**2q**
Empirical formula	$C_{17}H_{21}O_2F_3$
Formula weight	314.34
Temperature/K	100
Crystal system	monoclinic
Space group	$P2_1/c$
a/Å	13.750(3)
b/Å	10.610(2)
c/Å	11.460(2)
α/°	90
β/°	113.10(3)
γ/°	90
Volume/Å^3	1537.8(6)
Z	4
ρ_{calc}g/cm^3	1.358
μ/mm^{-1}	0.111
F(000)	664.0
Crystal size/mm^3	0.48 × 0.217 × 0.03
Radiation	Mo Kα (λ = 0.71073)
2Θ range for data collection/°	7.06 to 60.068
Index ranges	-19 ≤ h ≤ 19, -14 ≤ k ≤ 14, -14 ≤ l ≤ 16
Reflections collected	59408
Independent reflections	4476 [R_{int} = 0.0712, R_{sigma} = 0.0907]
Data/restraints/parameters	4476/10/218
Goodness-of-fit on F^2	0.846
Final R indexes [I>=2σ (I)]	R_1 = 0.0401, wR_2 = 0.0845
Final R indexes [all data]	R_1 = 0.0825, wR_2 = 0.0895
Largest diff. peak/hole / e Å^{-3}	0.25/-0.41

Table S5. Sample and crystal data, data collection and structure refinement. More detailed information can be found in the CIF code of CCDC: 2296102

2t – *cis*-(4-Butyl-4-hydroxycyclohexyl)[4-(trifluoromethyl)phenyl]methanone

Crystal Data for $C_{18}H_{23}F_3O_2$ (M =328.36 g/mol): monoclinic, space group $P2_1/c$ (no. 14), a = 12.1772(12) Å, b = 11.0656(9) Å, c = 12.4639(13) Å, β = 100.042(8)°, V = 1653.8(3) $Å^3$, Z = 4, T = 100 K, μ(Mo Kα) = 0.107 mm^{-1}, Dcalc = 1.319 g/cm^3, 20252 reflections measured (6.912° ≤ 2Θ ≤ 60.052°), 4694 unique (R_{int} = 0.0510, R_{sigma} = 0.1735) which were used in all calculations. The final R_1 was 0.0375 (I > 2σ(I)) and wR_2 was 0.0644 (all data).

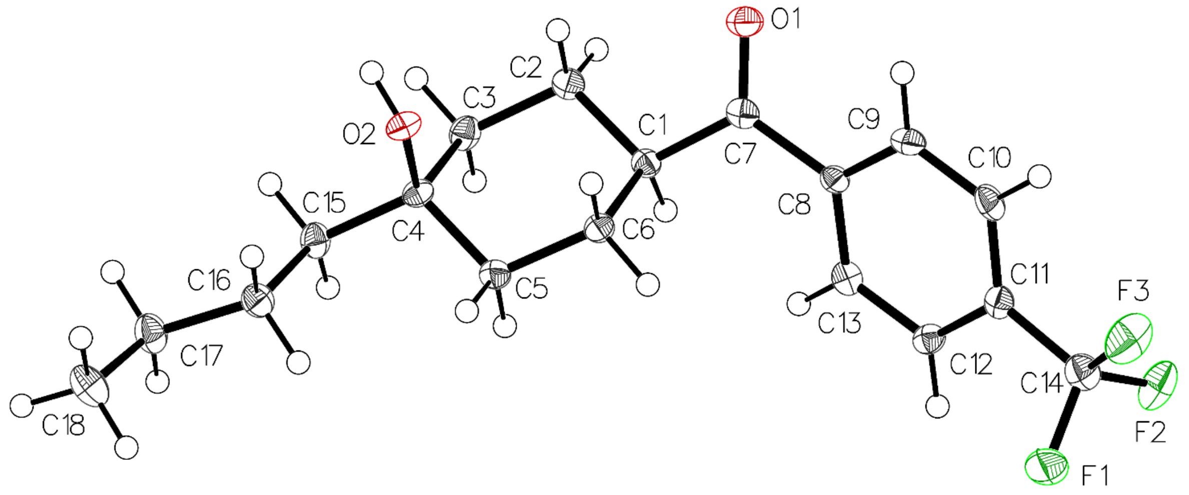

Figure S12. Crystal structure drawn with 50% displacement ellipsoid. The bond precision for C–C single bonds is 0.0017Å. Chiral positions are at C1 and C4. The centrosymmetric space group underpins the existence of the racemic form of the molecule.

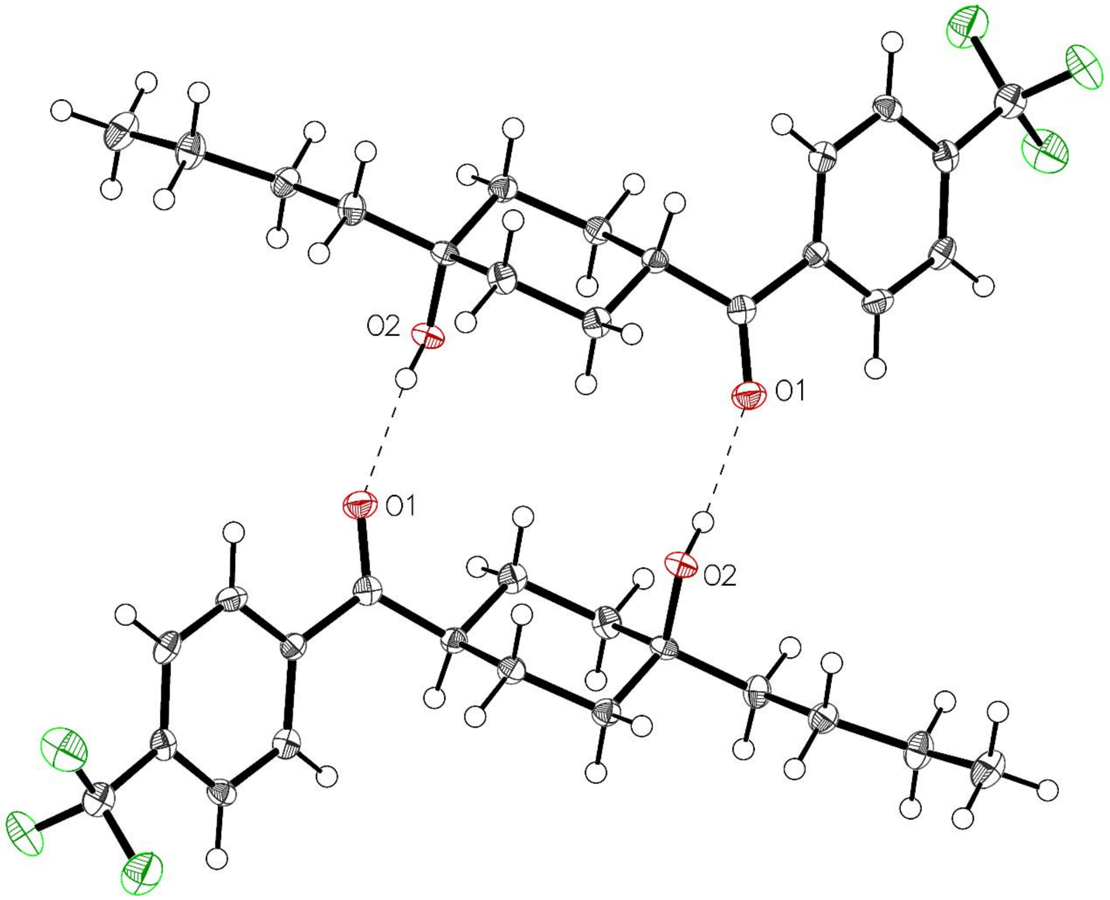

Figure S13. Two, for symmetry reasons, identical and moderate hydrogen bonds force a pairwise molecular setup in the packing. The packing shows no extraordinary effect and is characterized by van der Waals forces.

Identification code	**2t**
Empirical formula	$C_{18}H_{23}F_3O_2$
Formula weight	328.36
Temperature/K	100
Crystal system	monoclinic
Space group	$P2_1/c$
a/Å	12.1772(12)
b/Å	11.0656(9)
c/Å	12.4639(13)
α/°	90
β/°	100.042(8)
γ/°	90
Volume/Å^3	1653.8(3)
Z	4
ρ_{calc}g/cm^3	1.319
μ/mm^{-1}	0.107
F(000)	696.0
Crystal size/mm^3	0.3 × 0.263 × 0.22
Radiation	Mo Kα (λ = 0.71073)
2Θ range for data collection/°	6.912 to 60.052
Index ranges	-17 ≤ h ≤ 15, -15 ≤ k ≤ 15, -17 ≤ l ≤ 17
Reflections collected	20252
Independent reflections	4694 [R_{int} = 0.0510, R_{sigma} = 0.1735]
Data/restraints/parameters	4694/0/211
Goodness-of-fit on F^2	0.738
Final R indexes [I>=2σ (I)]	R_1 = 0.0375, wR_2 = 0.0615
Final R indexes [all data]	R_1 = 0.0823, wR_2 = 0.0643
Largest diff. peak/hole / e Å^{-3}	0.27/-0.32

Table S6. Sample and crystal data, data collection and structure refinement. Detailed information listed in the CIF code of CCDC: 2296103.

9 NMR Spectra

6a – *N*-Methoxy-*N*-methylcyclopentanecarboxamide

^{1}H NMR (400 MHz, $CDCl_3$)

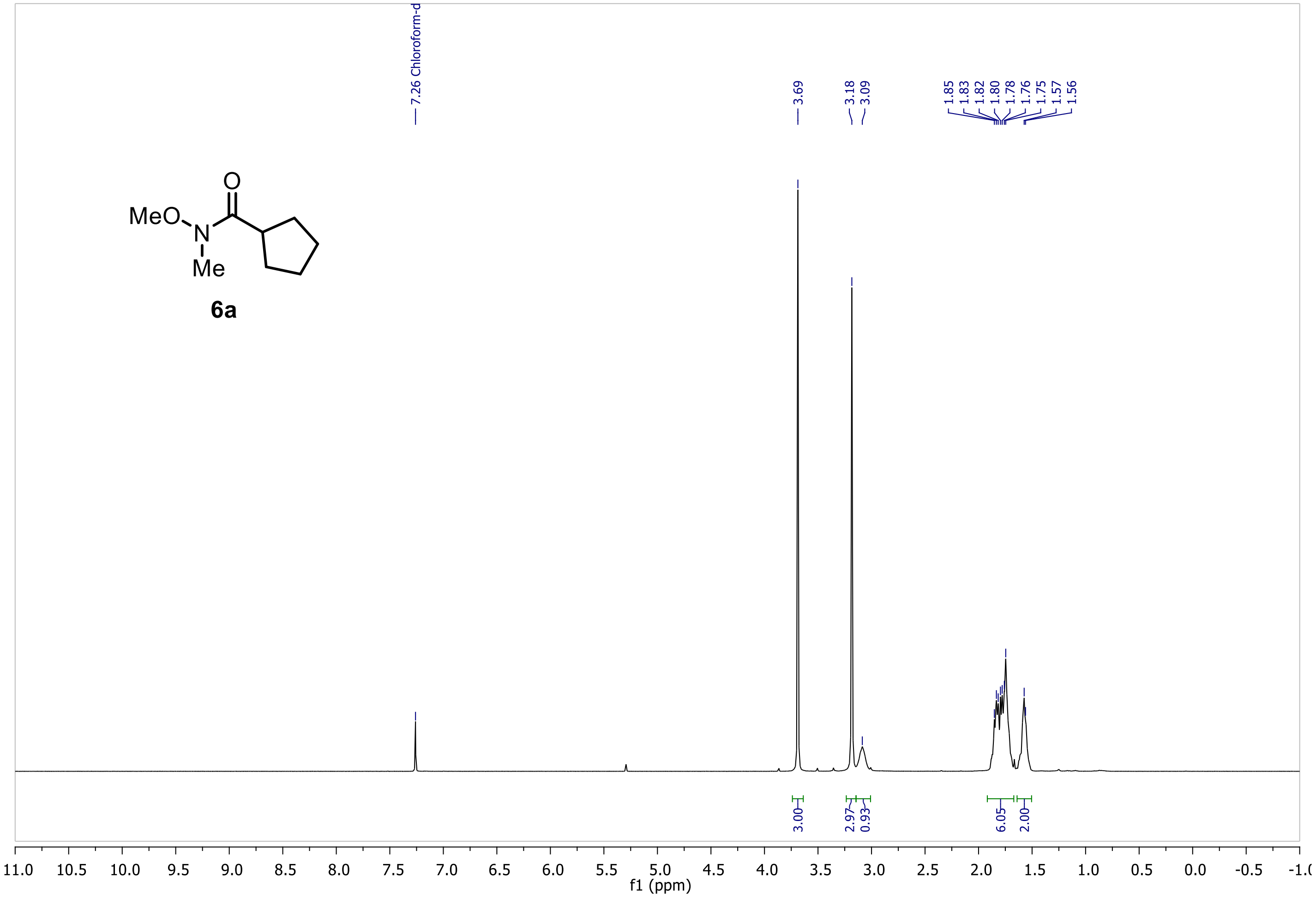

6b – *N*-Methoxy-*N*-methylcyclohexanecarboxamide

^{1}H NMR (400 MHz, $CDCl_3$)

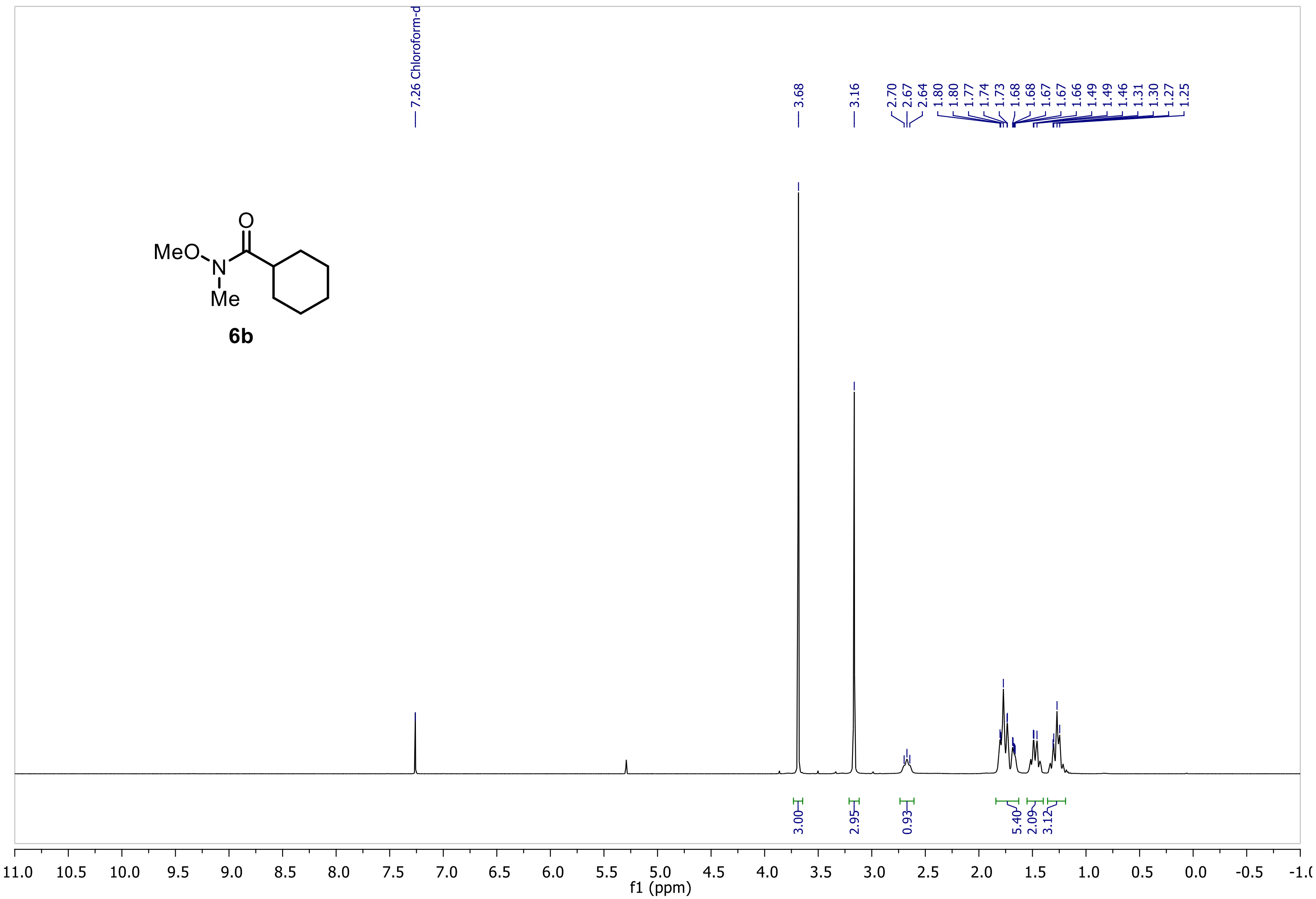

6c – 4-(*tert*-Butyl)-*N*-methoxy-*N*-methylcyclohexane-1-carboxamide

^{1}H NMR (700 MHz, $CDCl_3$)

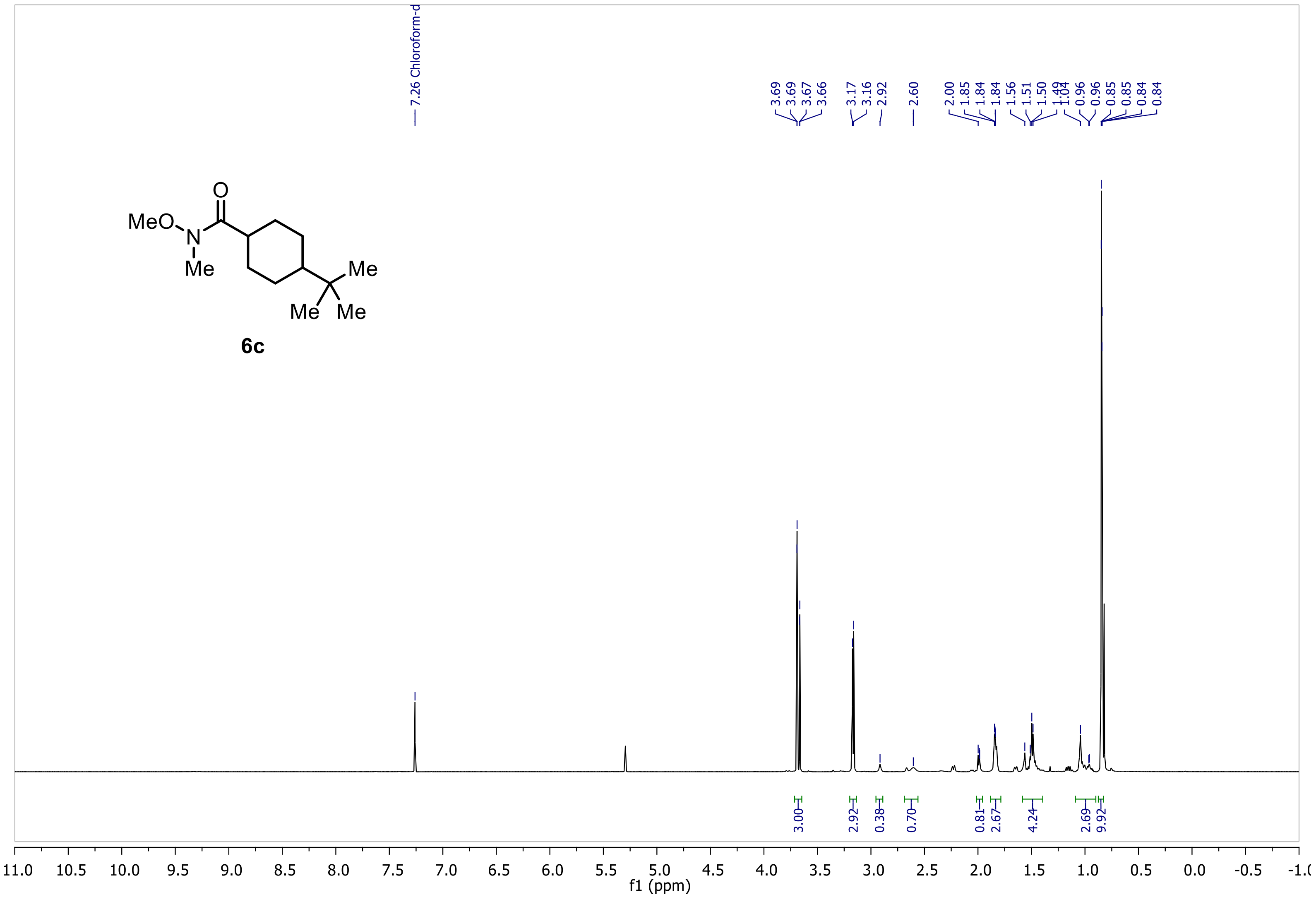

6d – *trans*-4-Butyl-*N*-methoxy-*N*-methylcyclohexane-1-carboxamide

^{1}H NMR (700 MHz, $CDCl_3$)

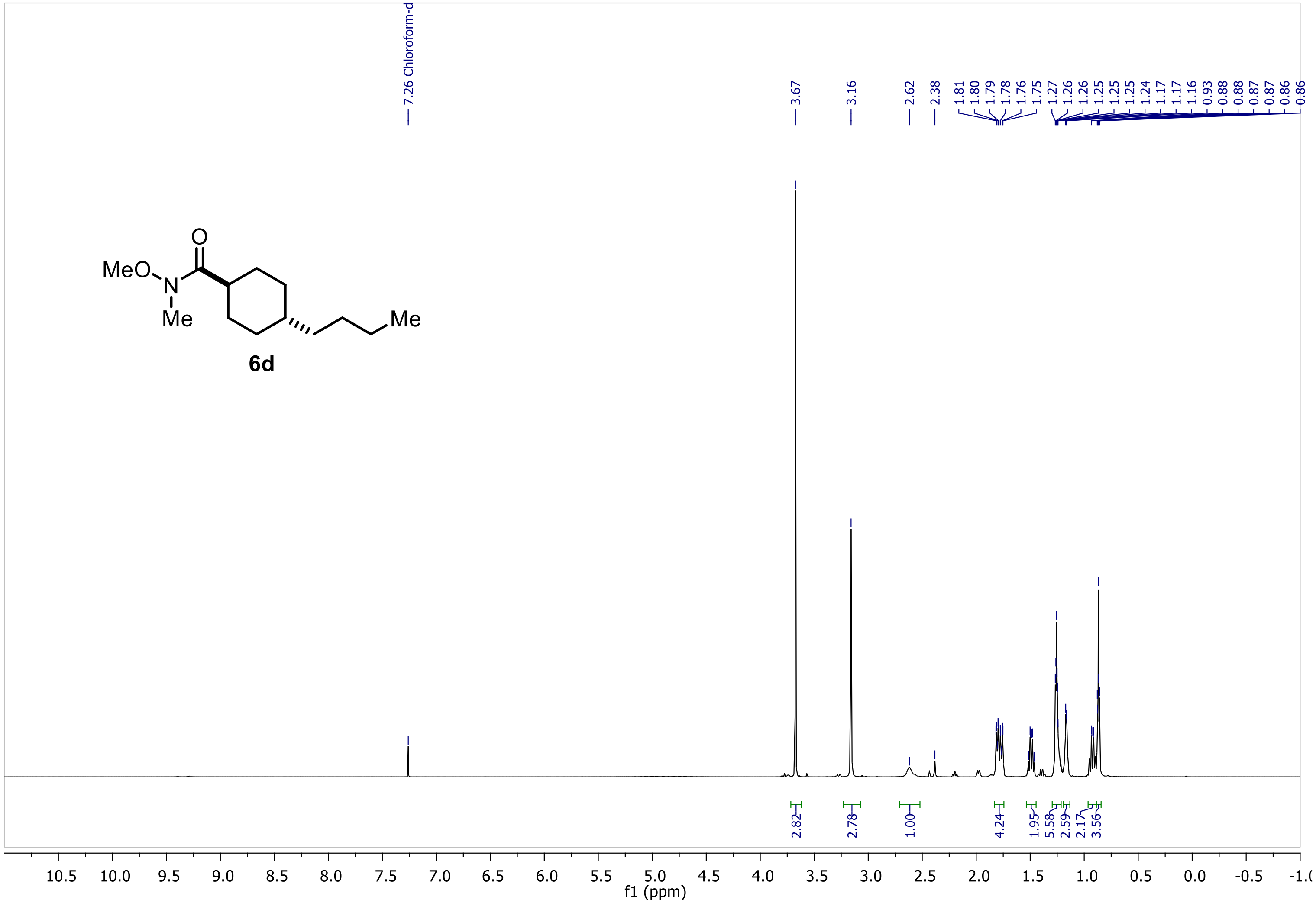

$^{13}C\{^1H\}$ NMR (176 MHz, $CDCl_3$)

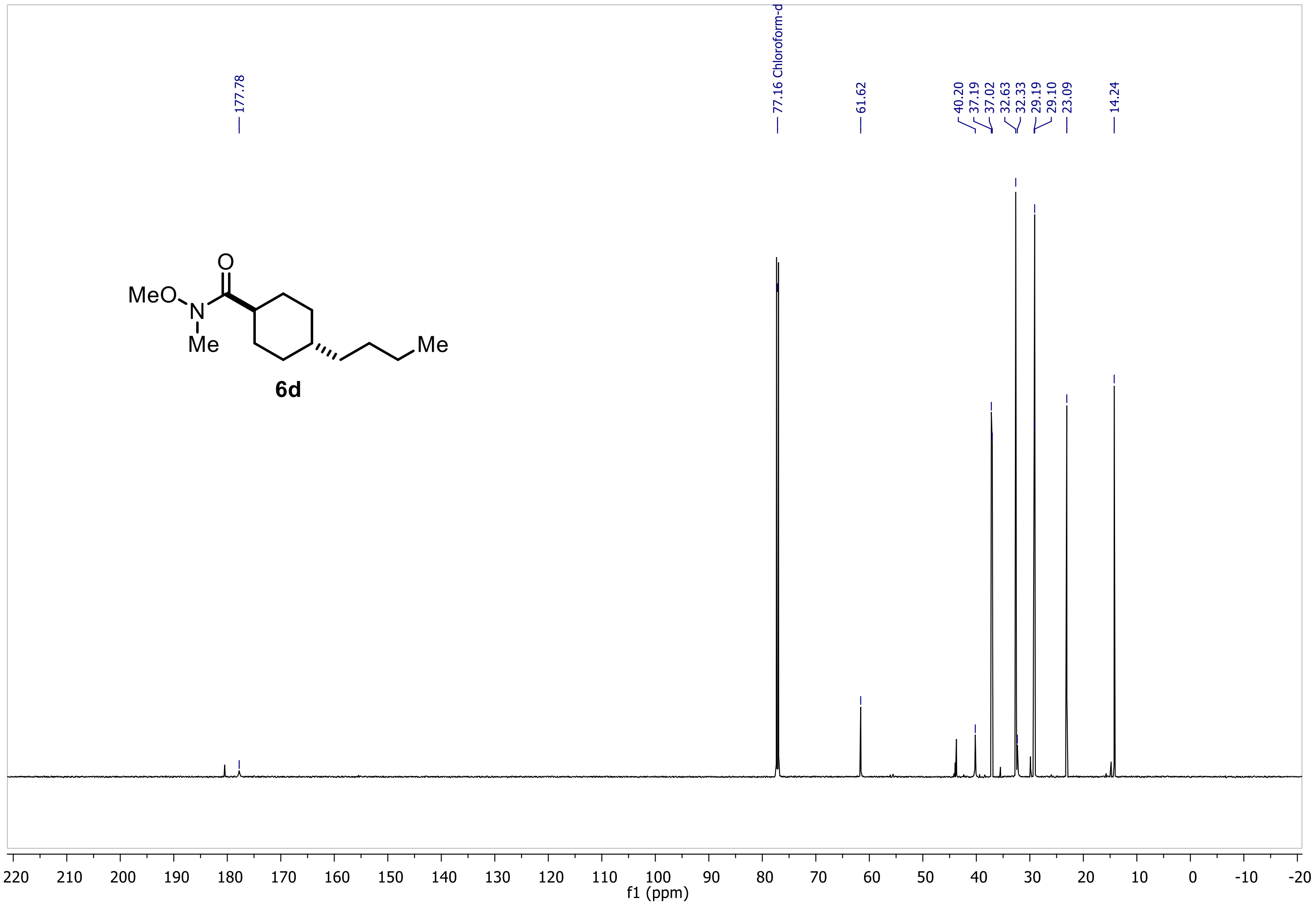

7a – *N*'-Cyclopentylidene-4-methylbenzenesulfonohydrazide

^{1}H NMR (400 MHz, $CDCl_3$)

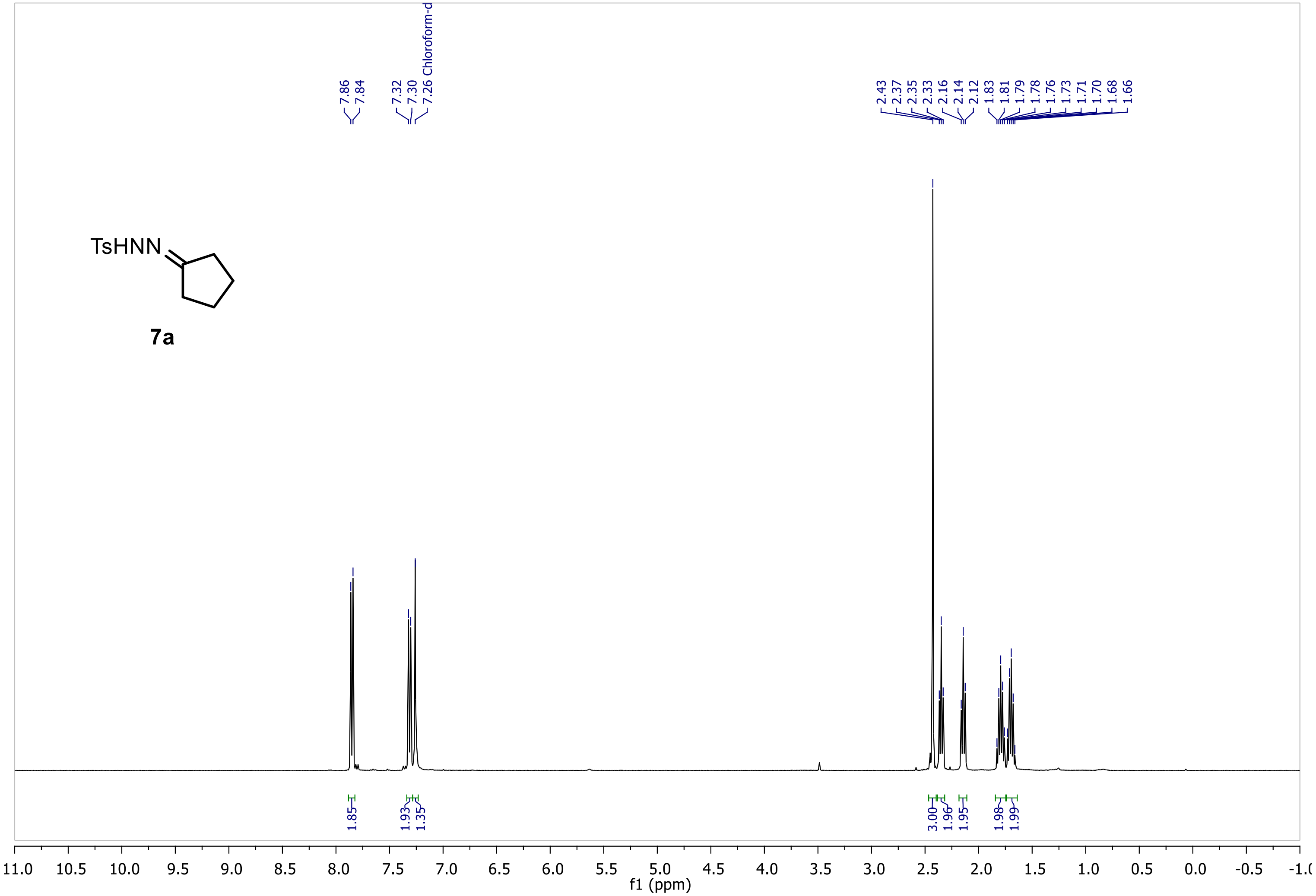

7b – *N*'-Cyclohexylidene-4-methylbenzenesulfonohydrazide

^{1}H NMR (400 MHz, $CDCl_3$)

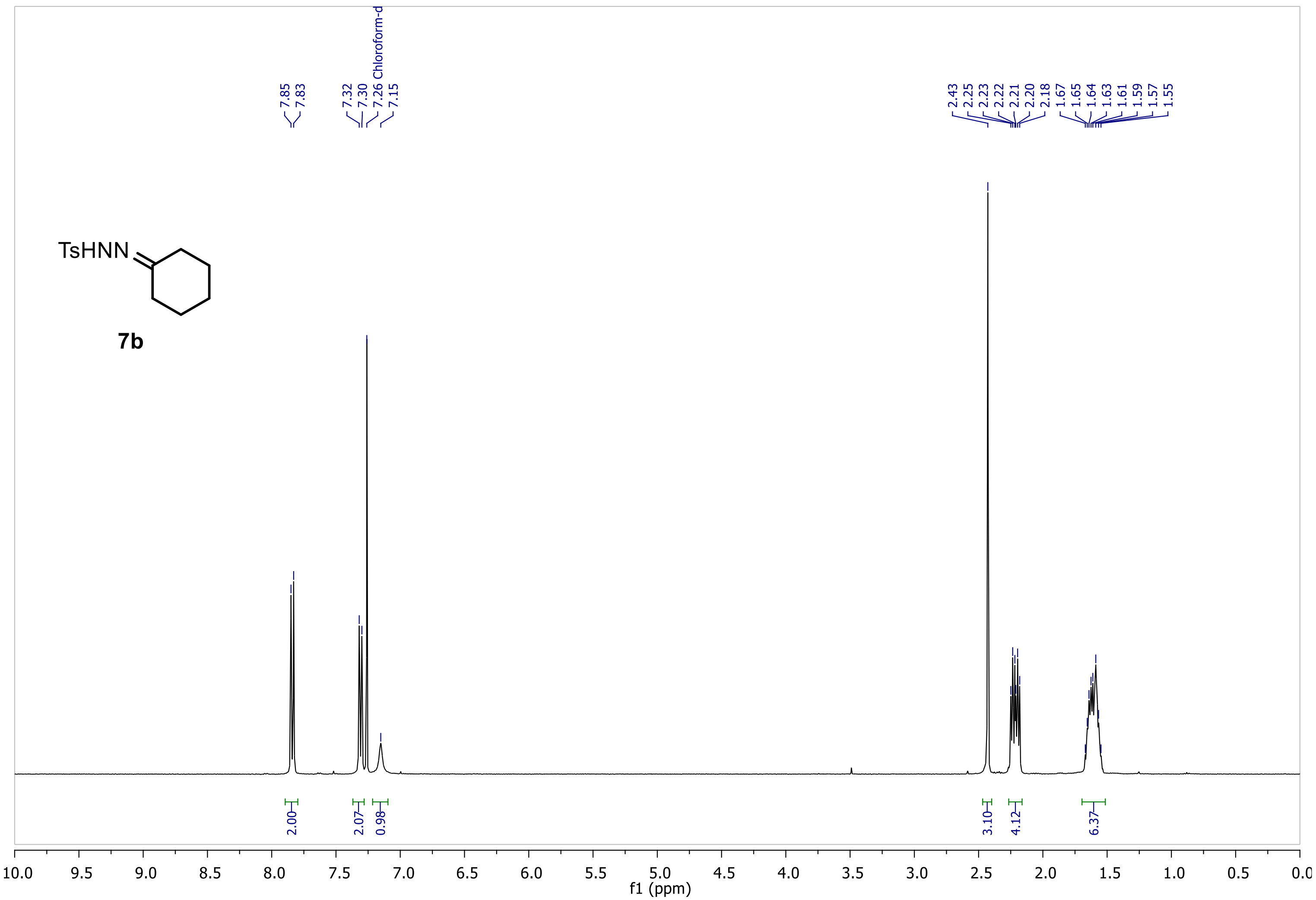

^{13}C (DEPT 135) NMR (101 MHz, $CDCl_3$)

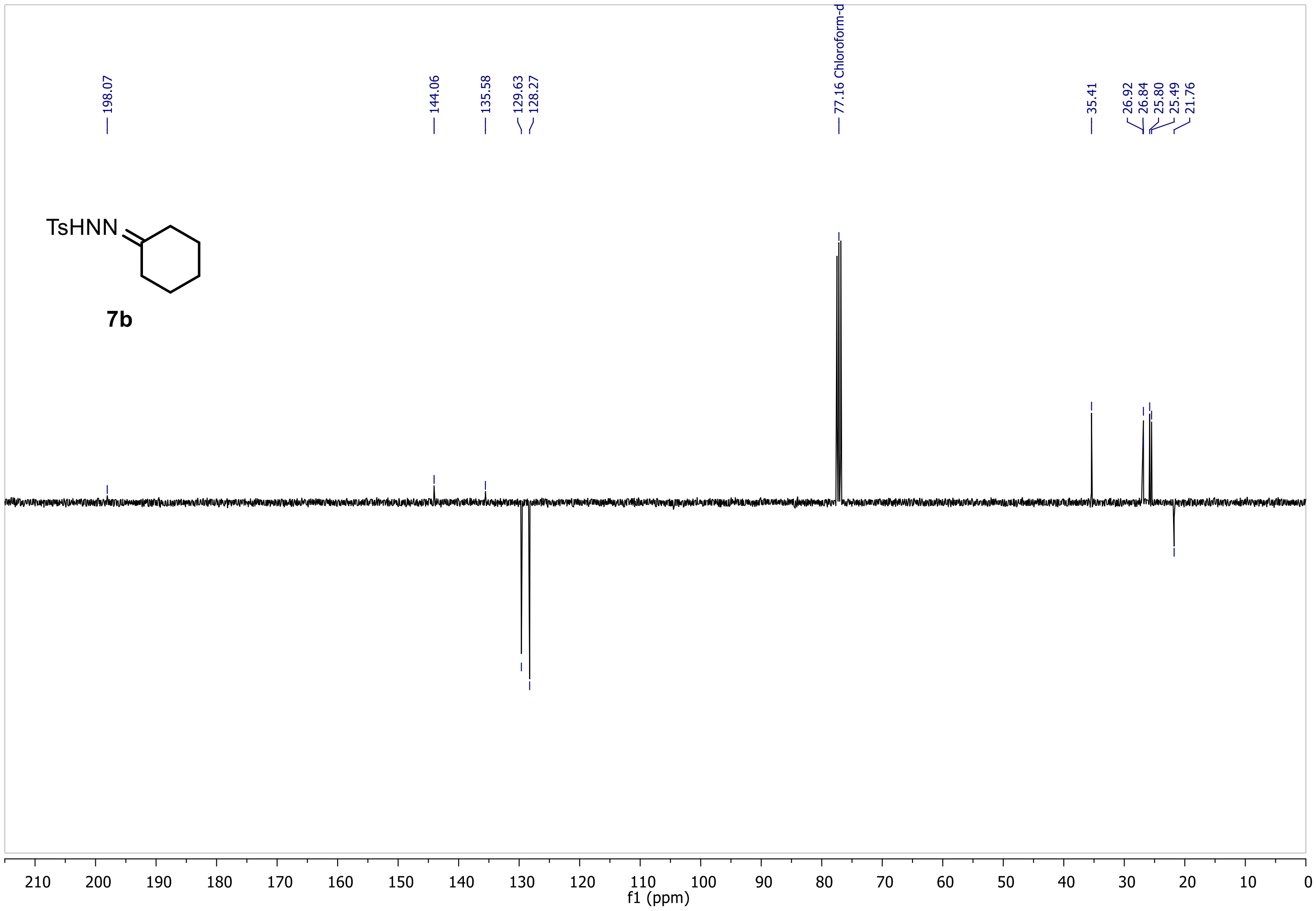

7c – 4-Methyl-*N*'-(3-methylcyclopentylidene)benzenesulfonohydrazide

^{1}H NMR (700 MHz, $CDCl_3$)

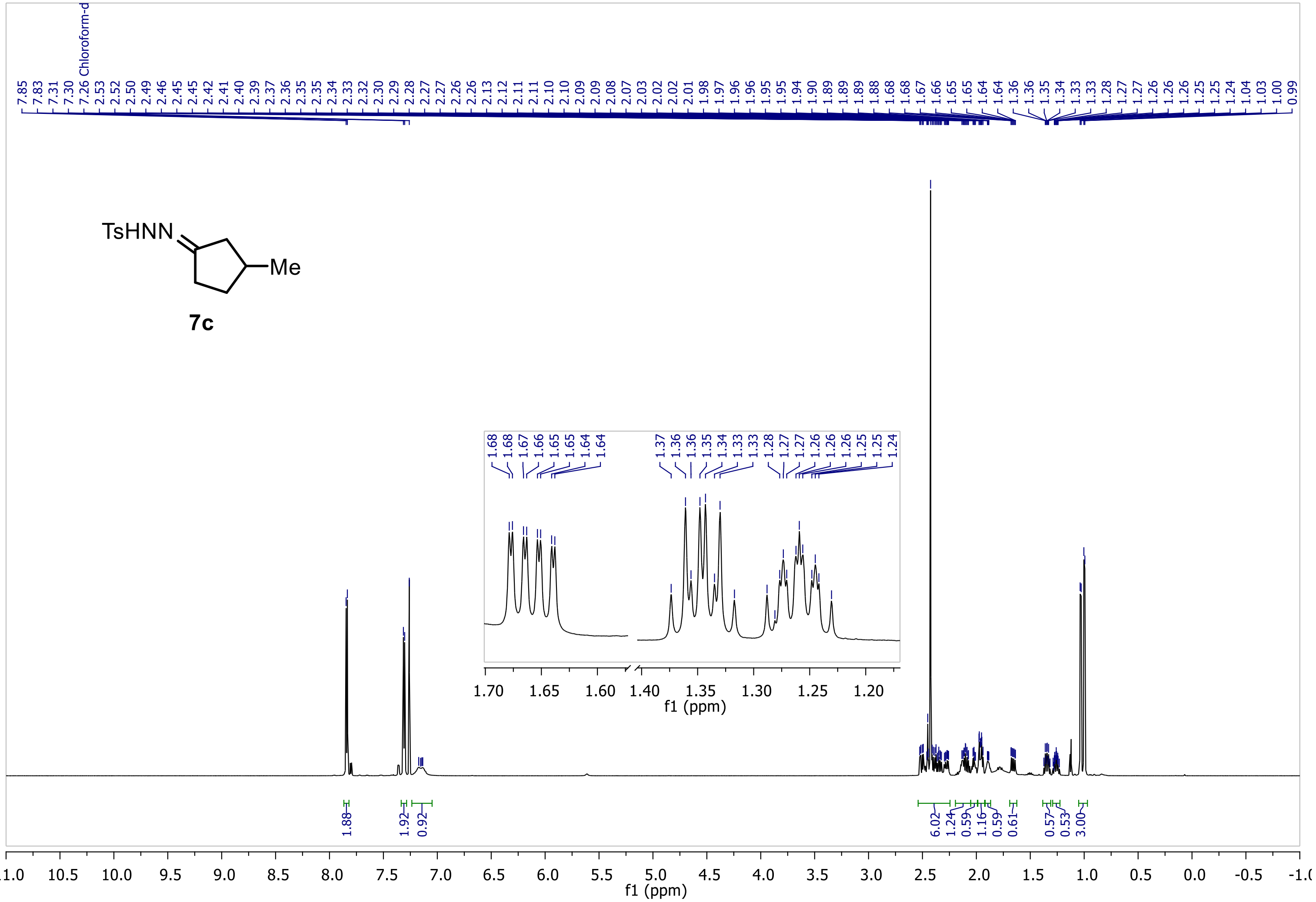

$^{13}C\{^1H\}$ NMR (176 MHz, $CDCl_3$)

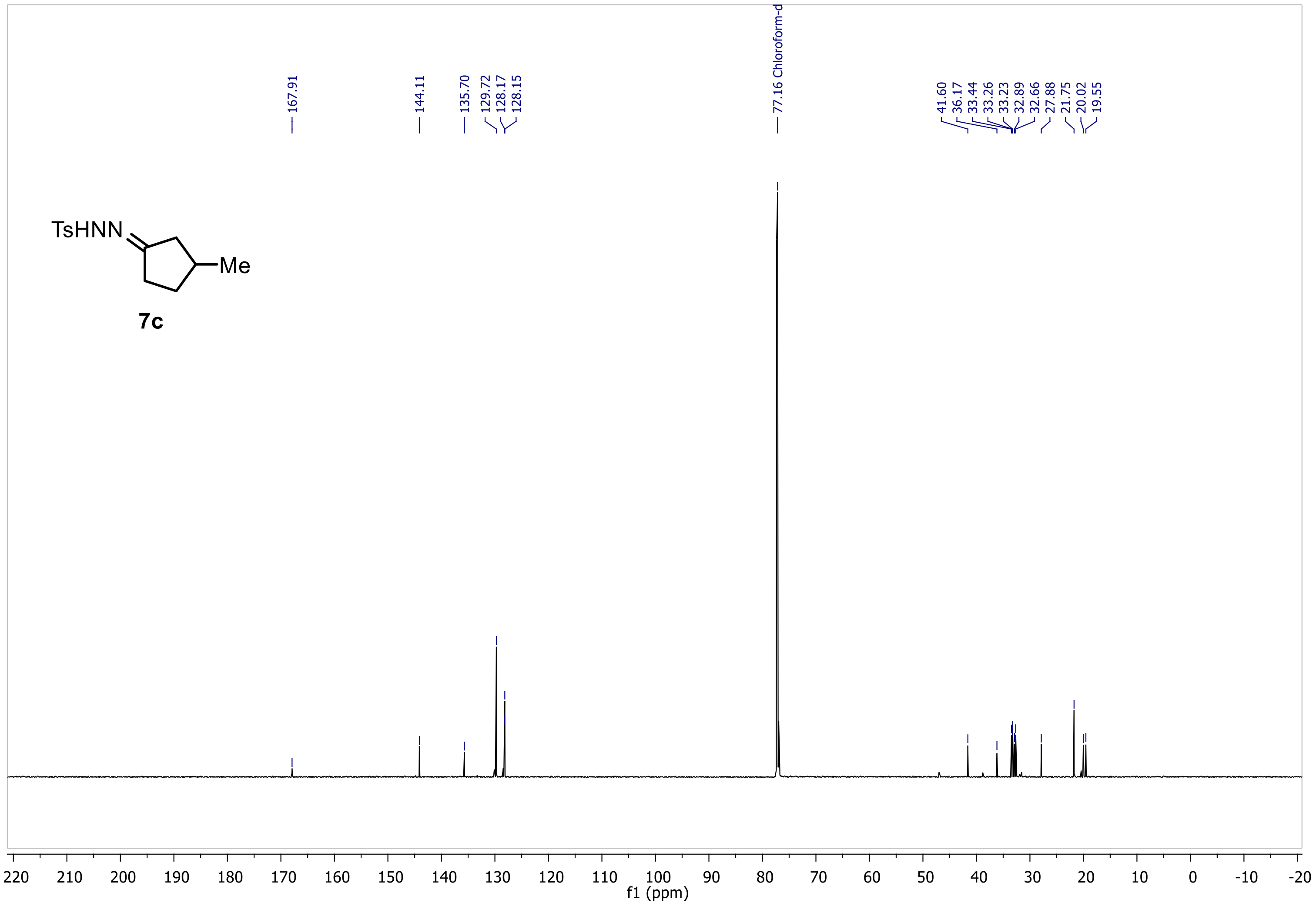

7d – 4-Methyl-*N*'-(3-methylcyclohexylidene)benzenesulfonohydrazide

^{1}H NMR (400 MHz, $CDCl_3$)

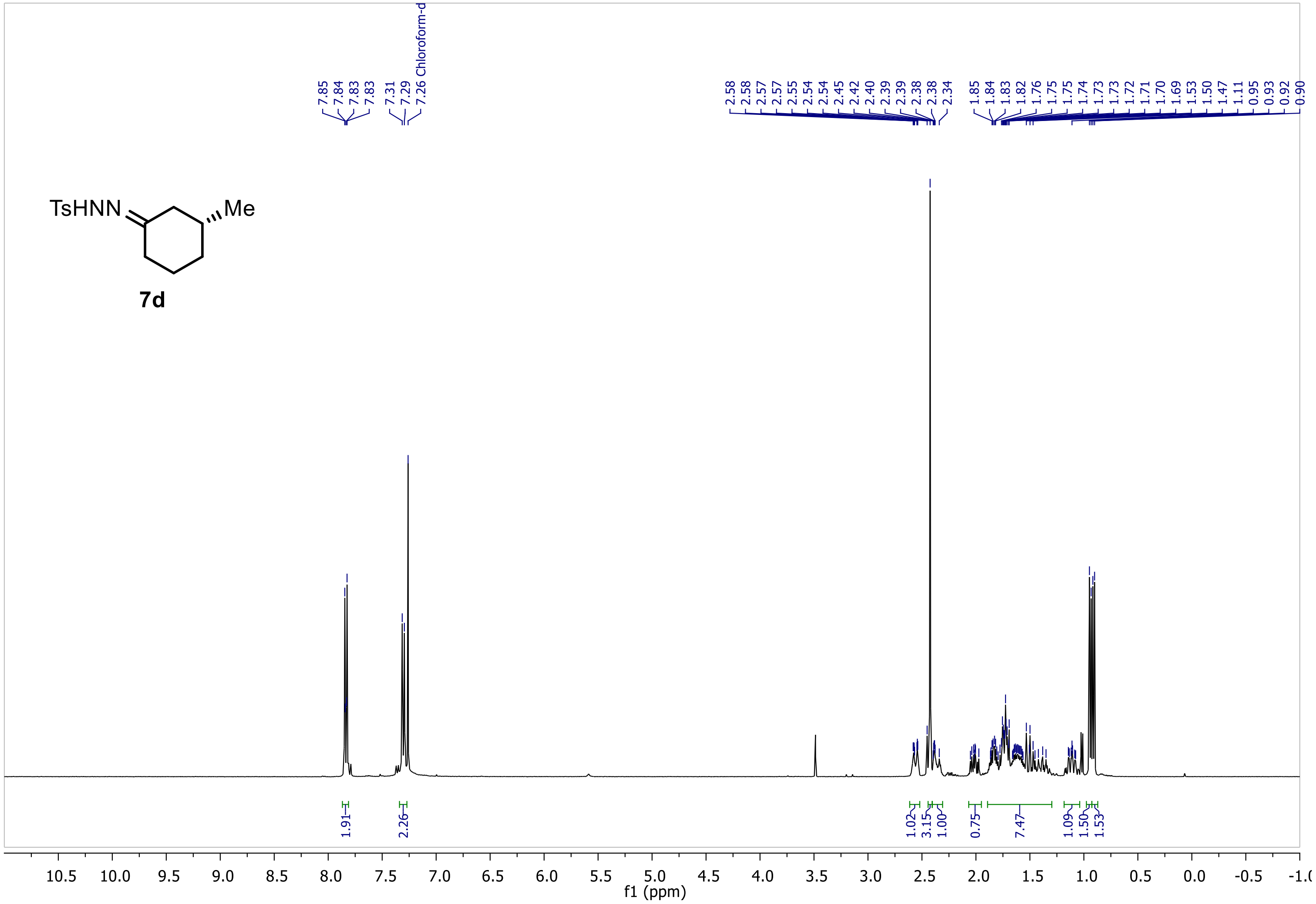

7e – *N*'-[3-(*tert*-Butyl)cyclopentylidene]-4-methylbenzenesulfonohydrazide

^{1}H NMR (400 MHz, $CDCl_3$)

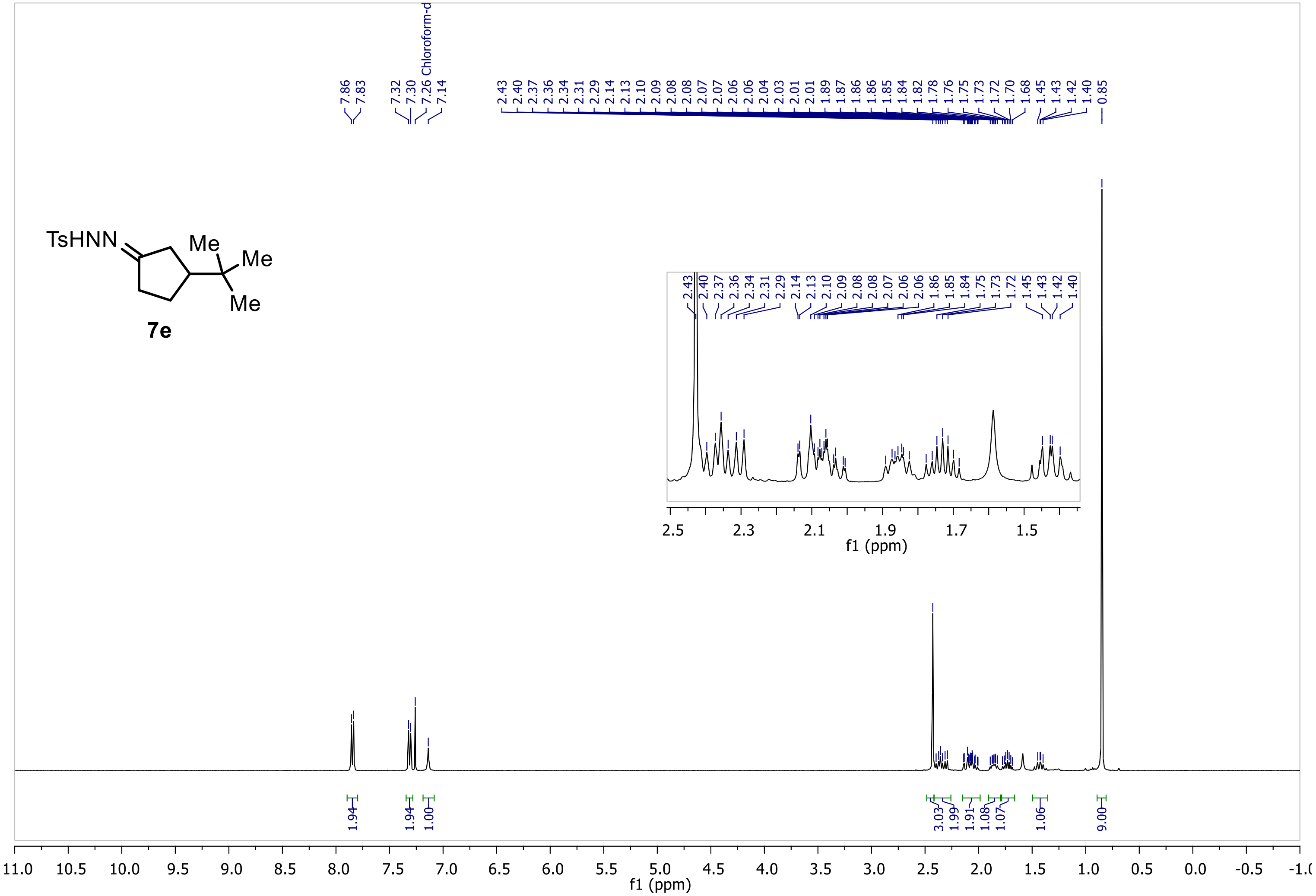

$^{13}C\{^{1}H\}$ NMR (101 MHz, $CDCl_3$)

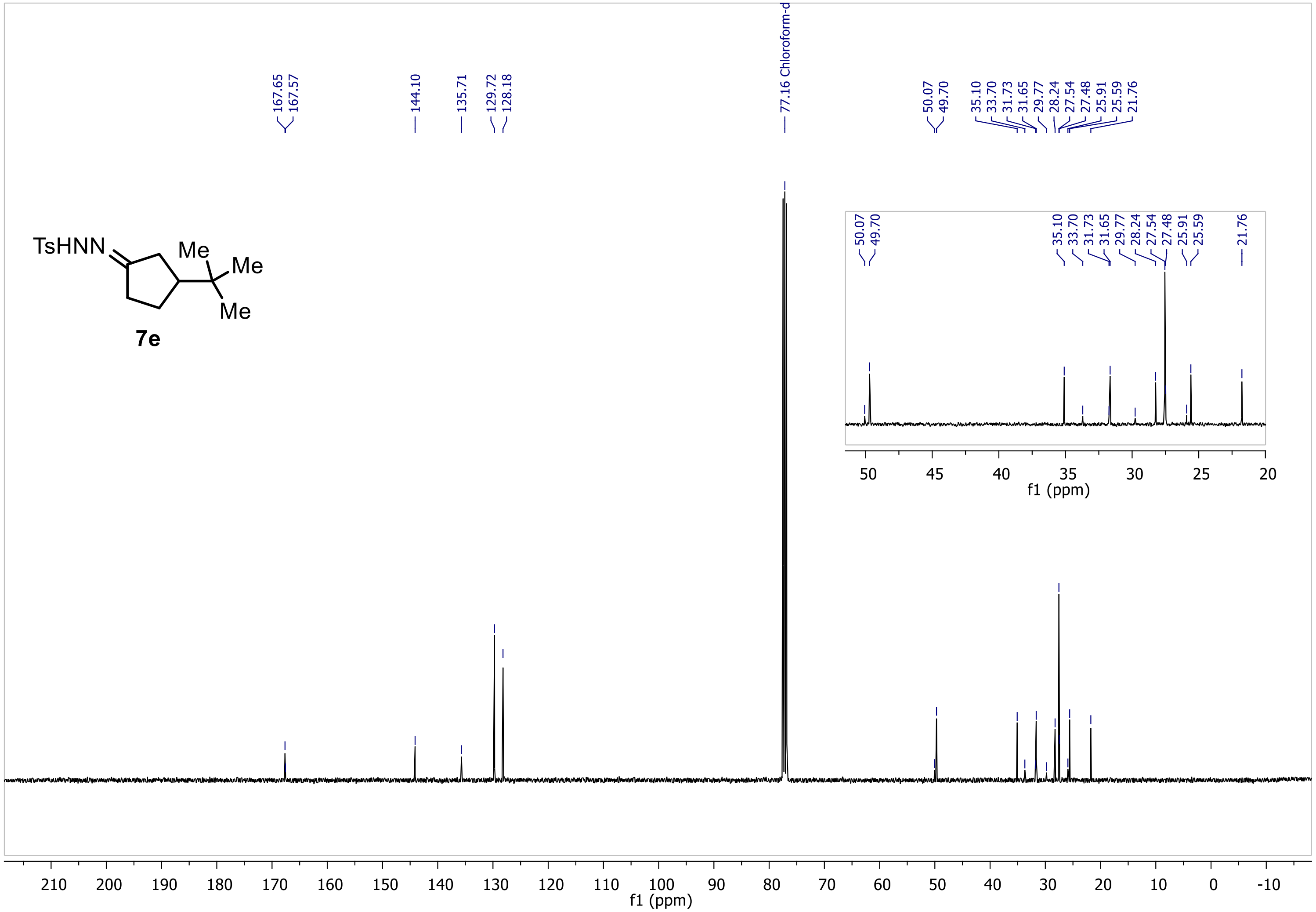

7e

7f – 4-Methyl-*N*'-(3,3,5-trimethylcyclohexylidene)benzenesulfonohydrazide

^{1}H NMR (600 MHz, $CDCl_3$)

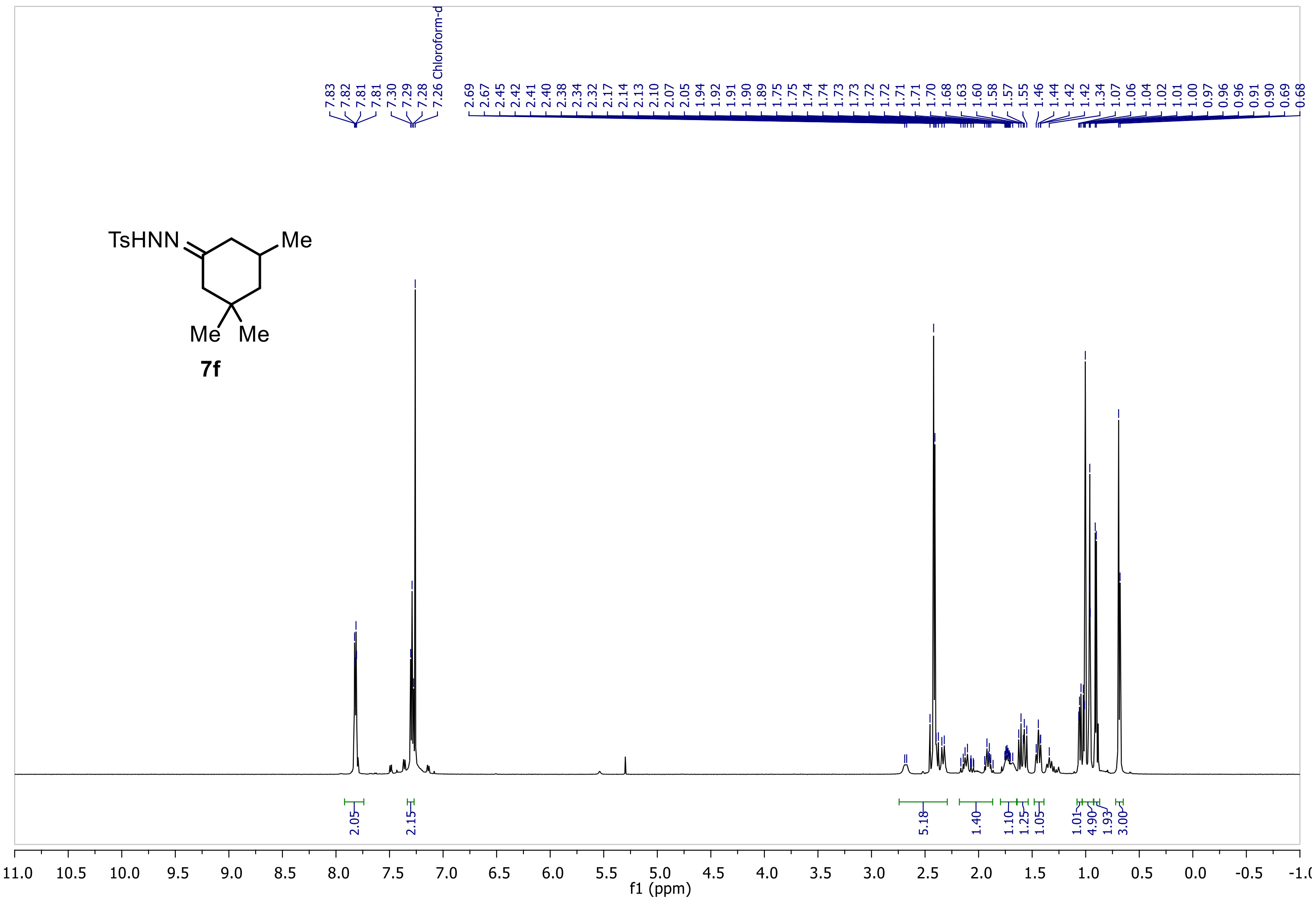

^{13}C (DEPT 135) NMR (151 MHz, $CDCl_3$)

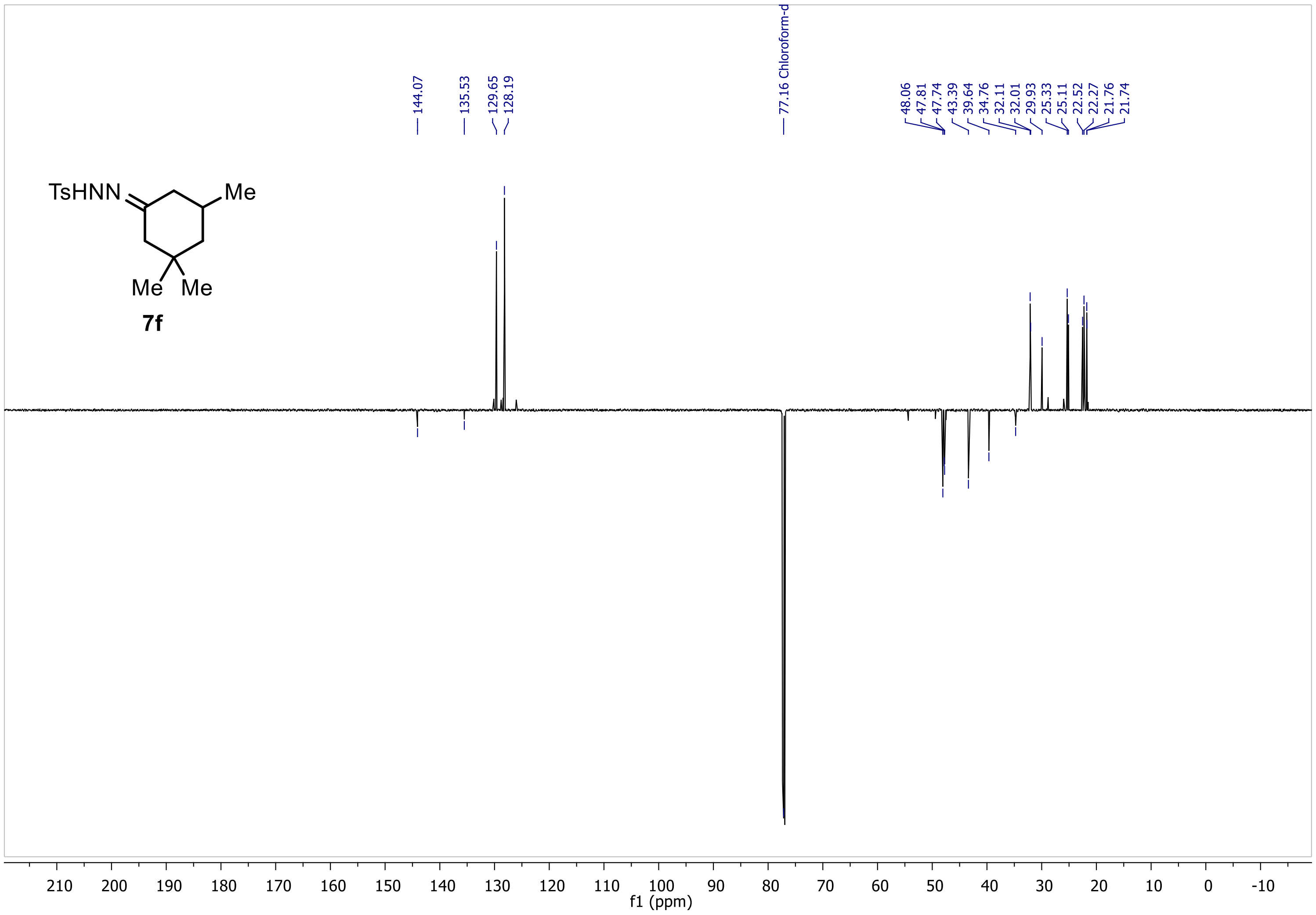

7g – 4-Methyl-*N*'-(4-methylcyclohexylidene)benzenesulfonohydrazide

^{1}H NMR (400 MHz, $CDCl_3$)

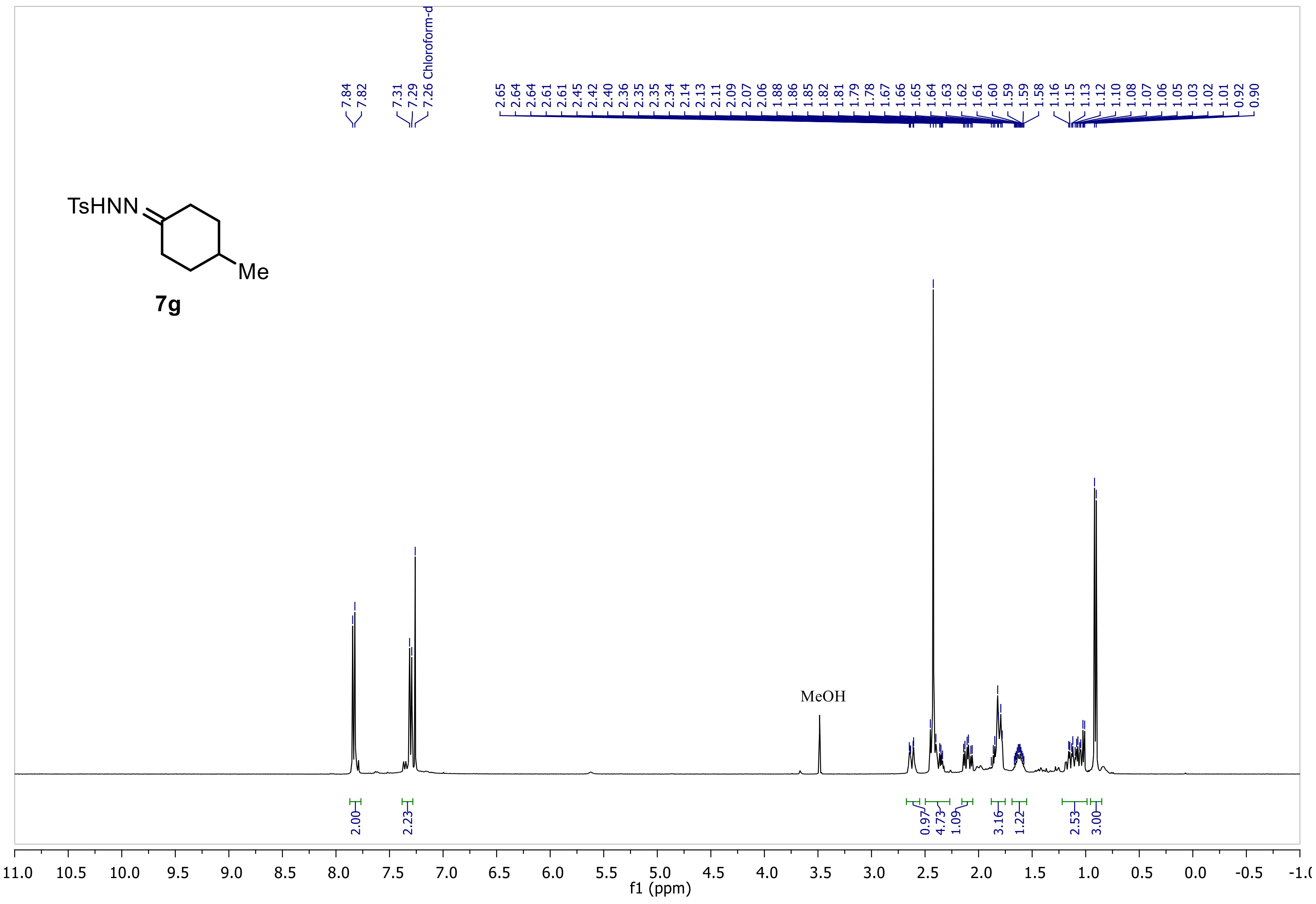

7h – *N*'-((5*S*,8*R*,9*S*,10*S*,13*R*,14*S*,17*R*,*E*)-10,13-Dimethyl-17-((*R*)-6-methylheptan-2-yl)hexa- -decahydro-3H-cyclopenta[a]phenanthren-3-ylidene)-4-methylbenzenesulfonohydrazide

^{1}H NMR (400 MHz, CDCl$_3$)

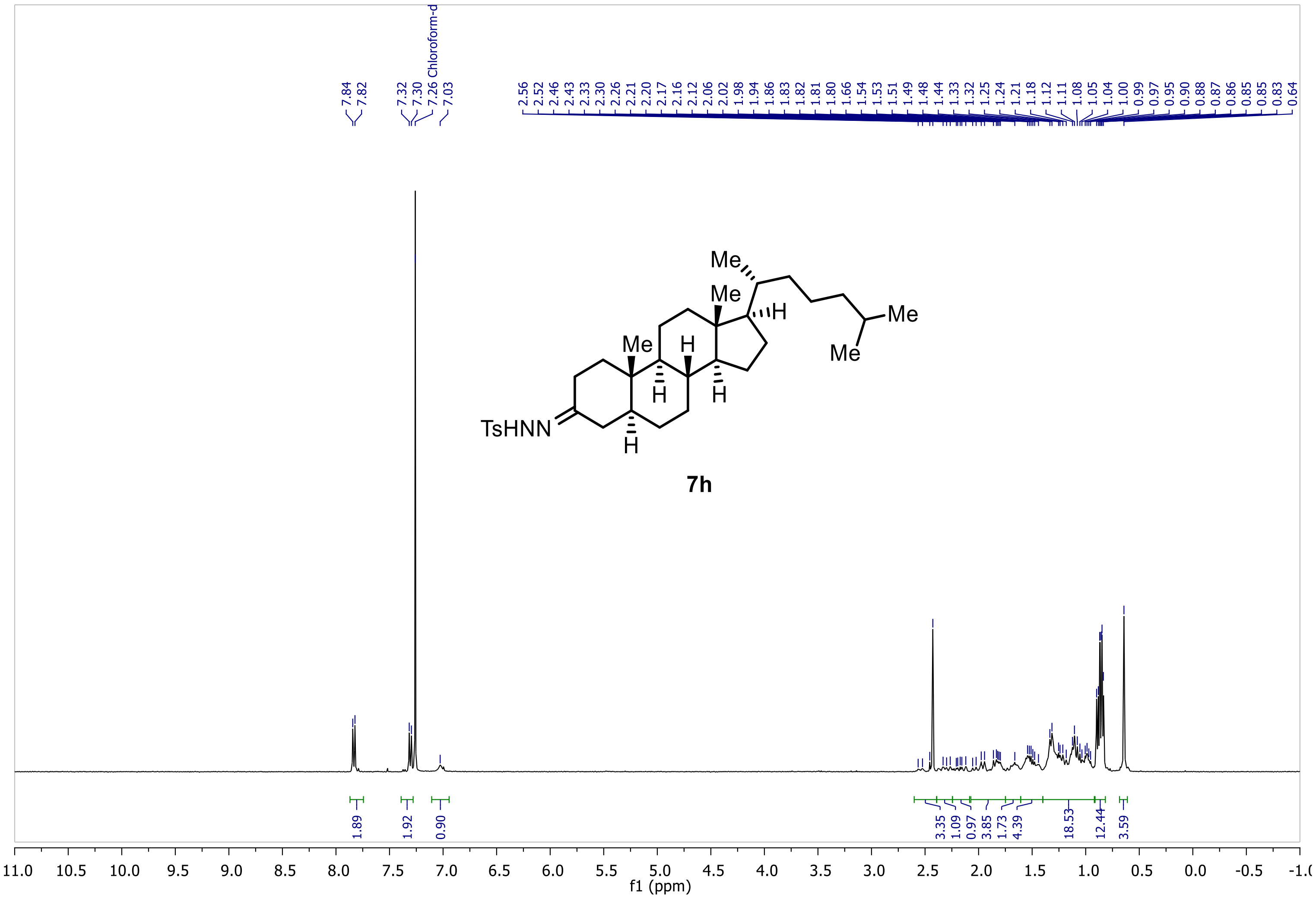

7i – *N'*-(cyclopentylidene-2,2,5,5-d_4)-4-methylbenzenesulfonohydrazide

^{1}H NMR (400 MHz, $CDCl_3$):

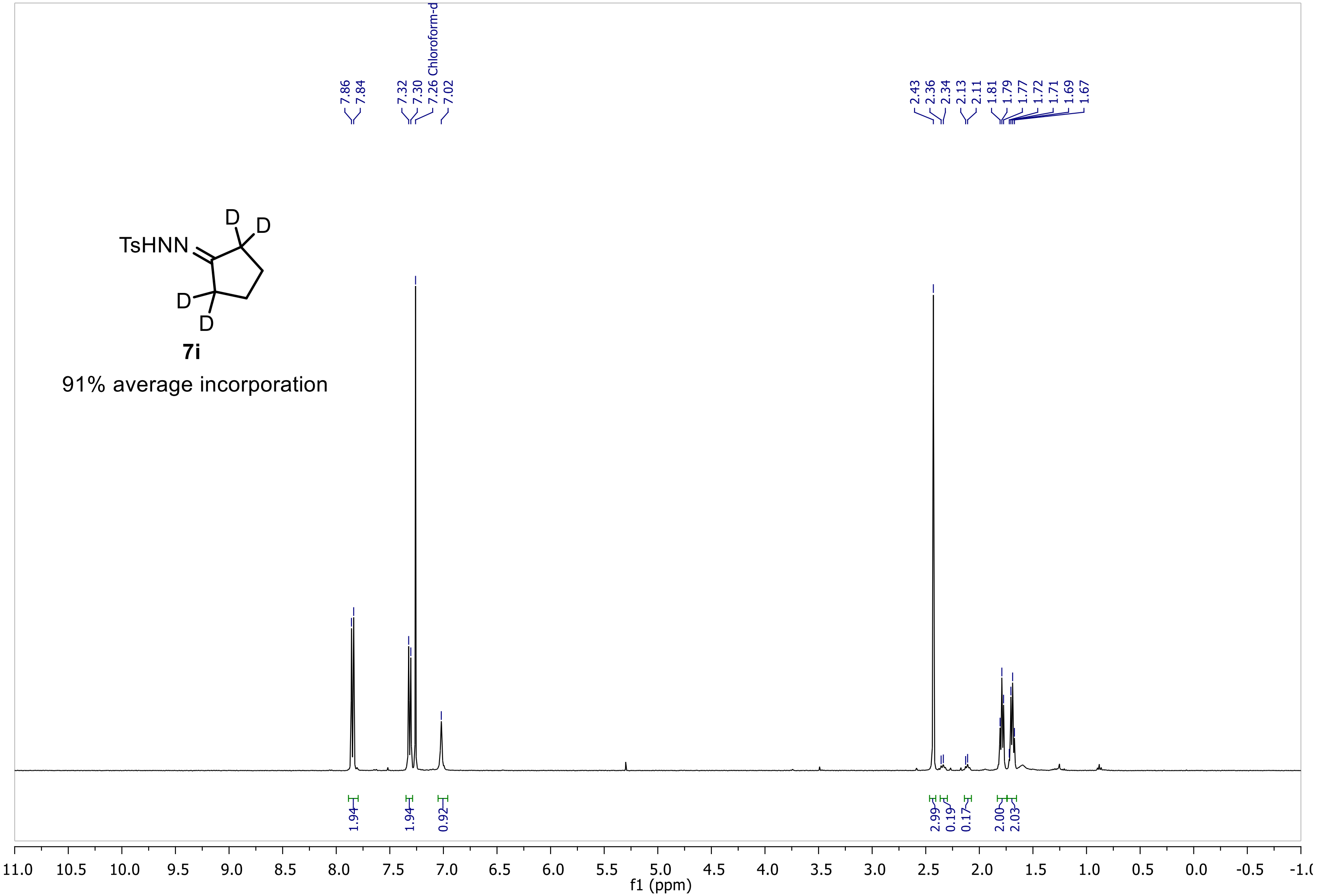

7j – 4-Methyl-*N*'-(3-methylcyclopentylidene-2,2,5,5-d_4)benzenesulfonohydrazide

^{1}H NMR (400 MHz, $CDCl_3$):

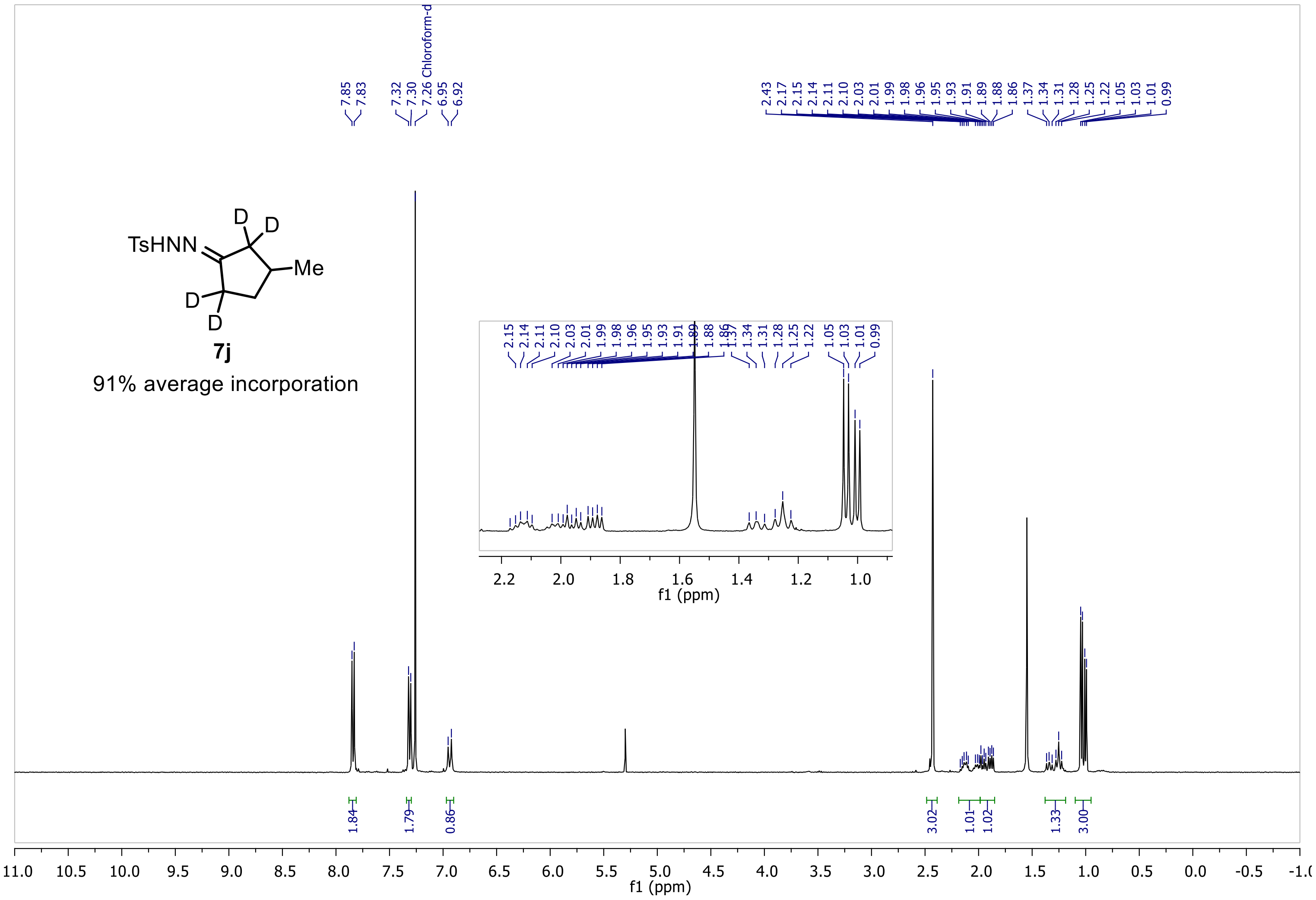

$^{13}C\{^1H\}$ NMR (101 MHz, $CDCl_3$):

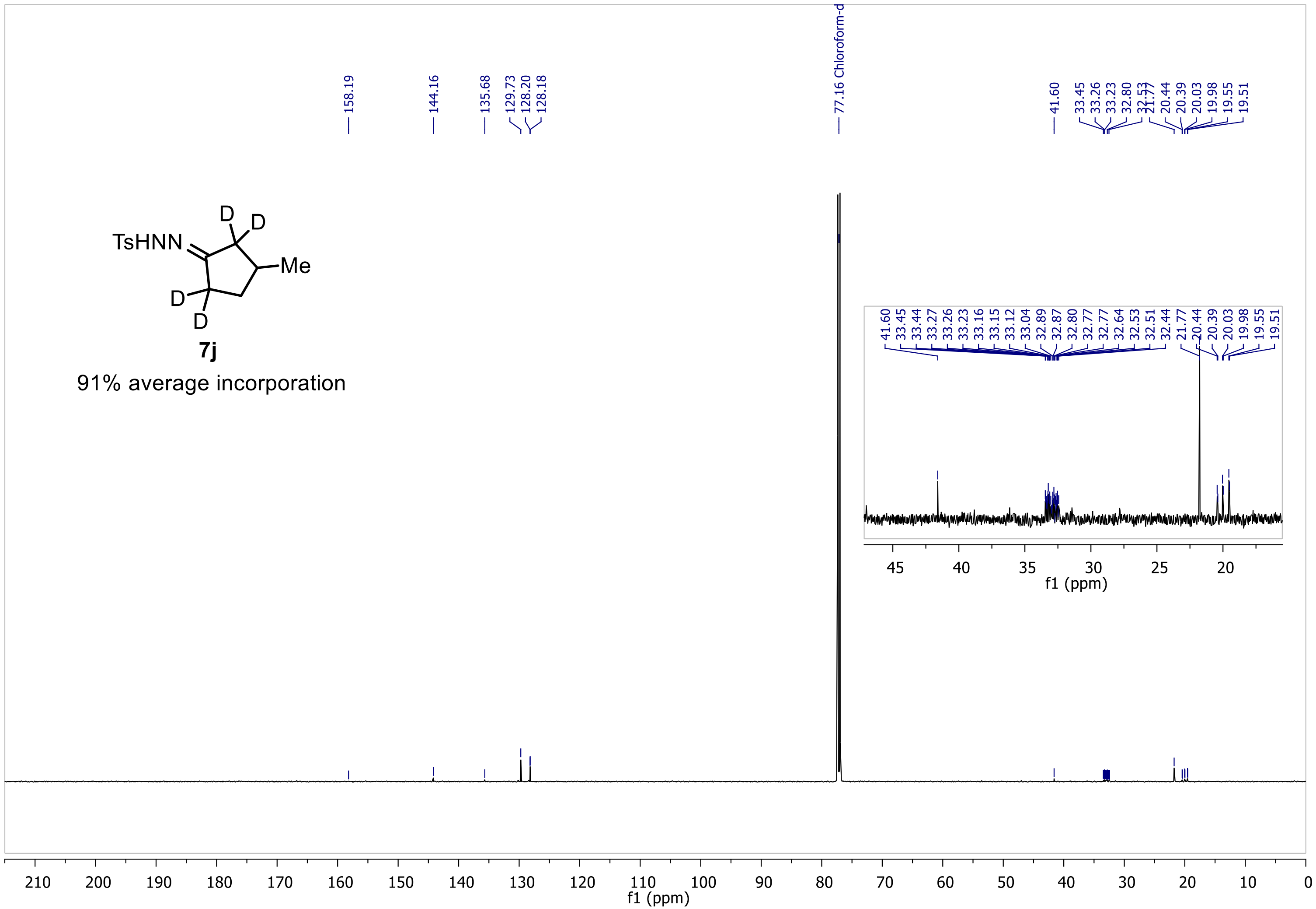

8a – (3-Methylcyclohexyl)[4-(trifluoromethyl)phenyl]methanone

^{1}H NMR (400 MHz, $CDCl_3$)

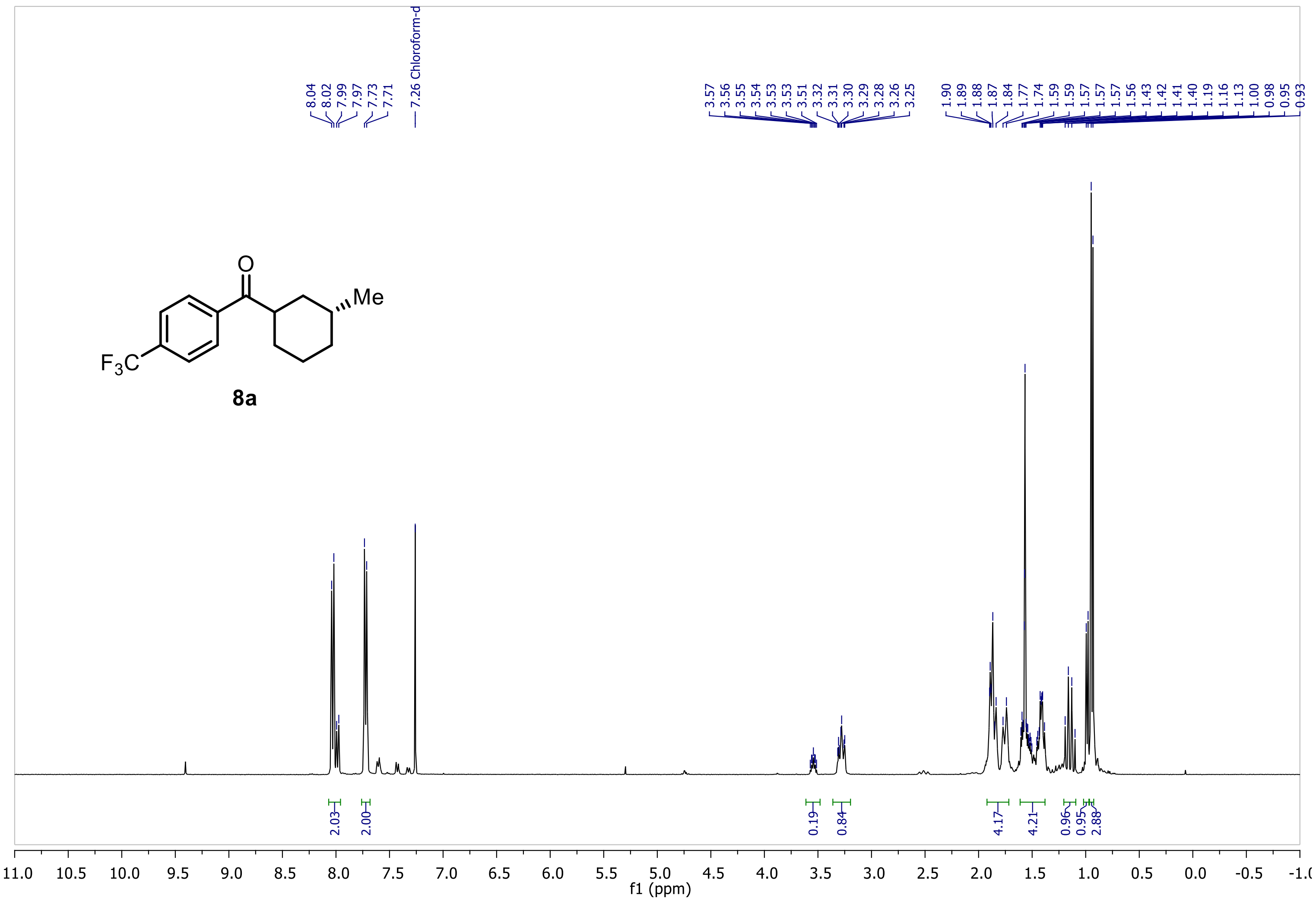

^{19}F NMR (376 MHz, $CDCl_3$)

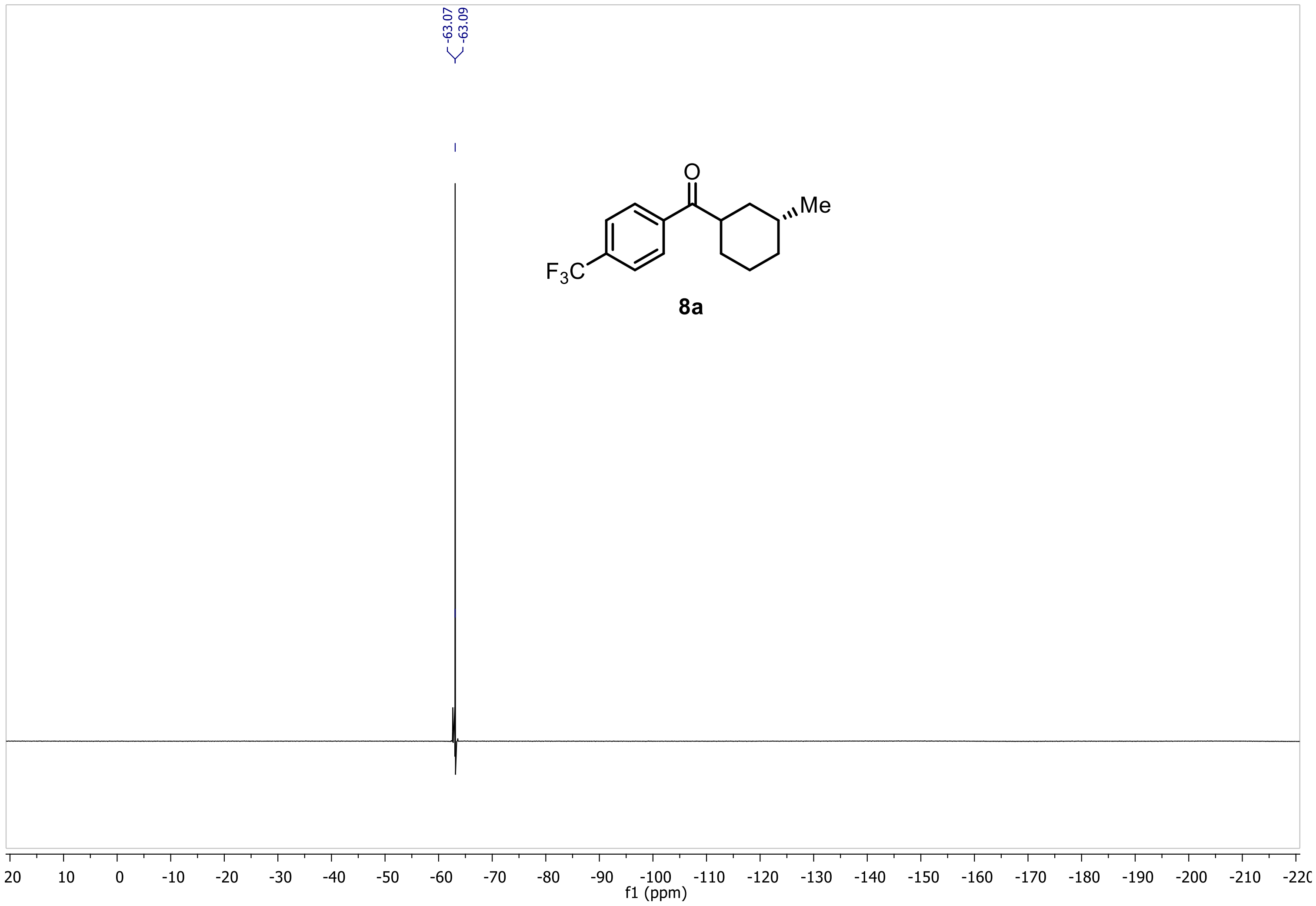

8a

8b – ((5*S*,8*R*,9*S*,10*S*,13*R*,14*S*,17*R*)-10,13-Dimethyl-17-((*R*)-6-methylheptan-2-yl)hexadecahydro-1*H*-cyclopenta[*a*]phenanthren-3-yl)(4-(trifluoromethyl)phenyl)methanone

^{1}H NMR (400 MHz, $CDCl_3$)

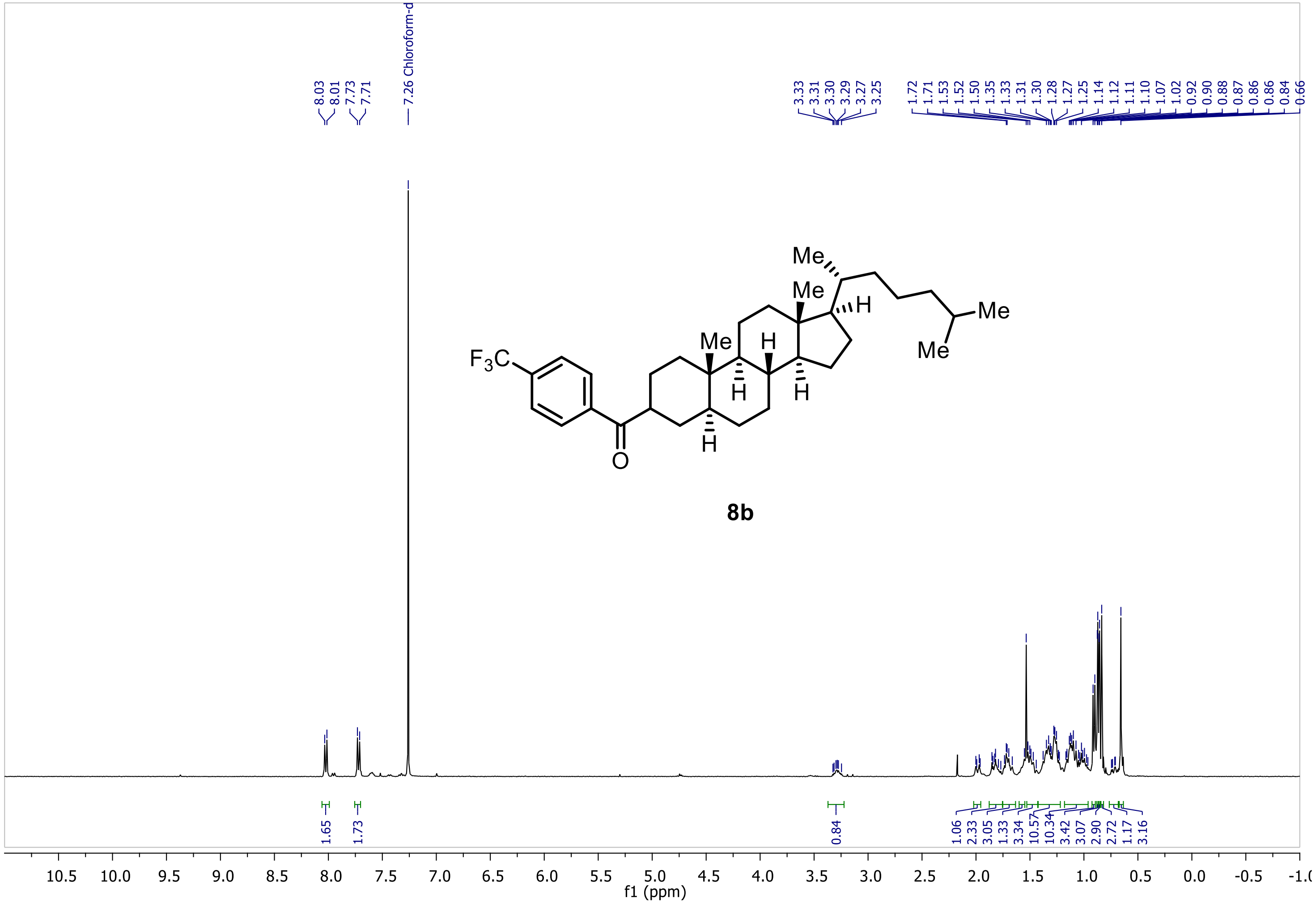

^{19}F NMR (376 MHz, $CDCl_3$)

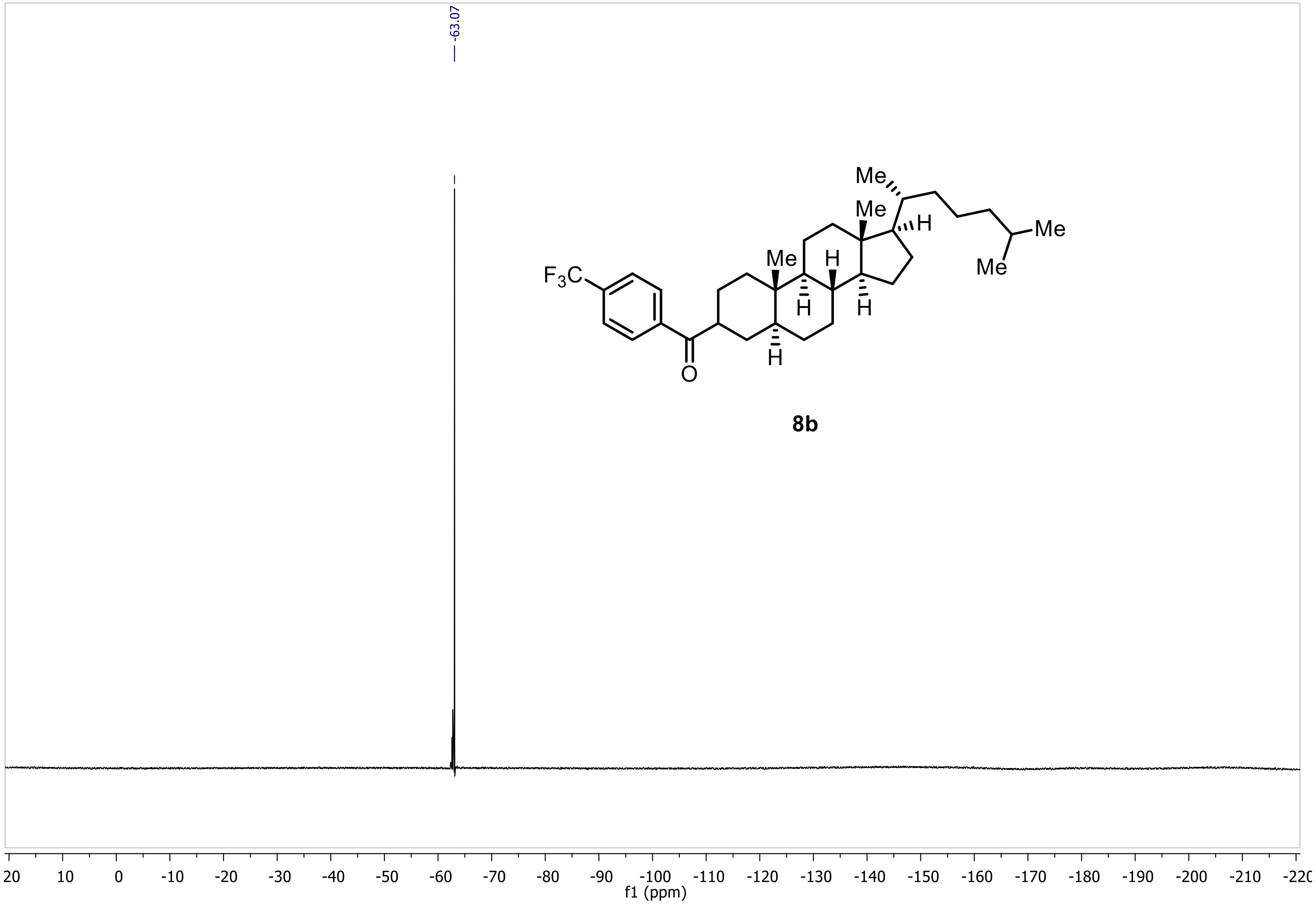

8b

8c – [4-(trifluoromethyl)phenyl](3,3,5-trimethylcyclohexyl)methanone

^{1}H NMR (400 MHz, $CDCl_3$)

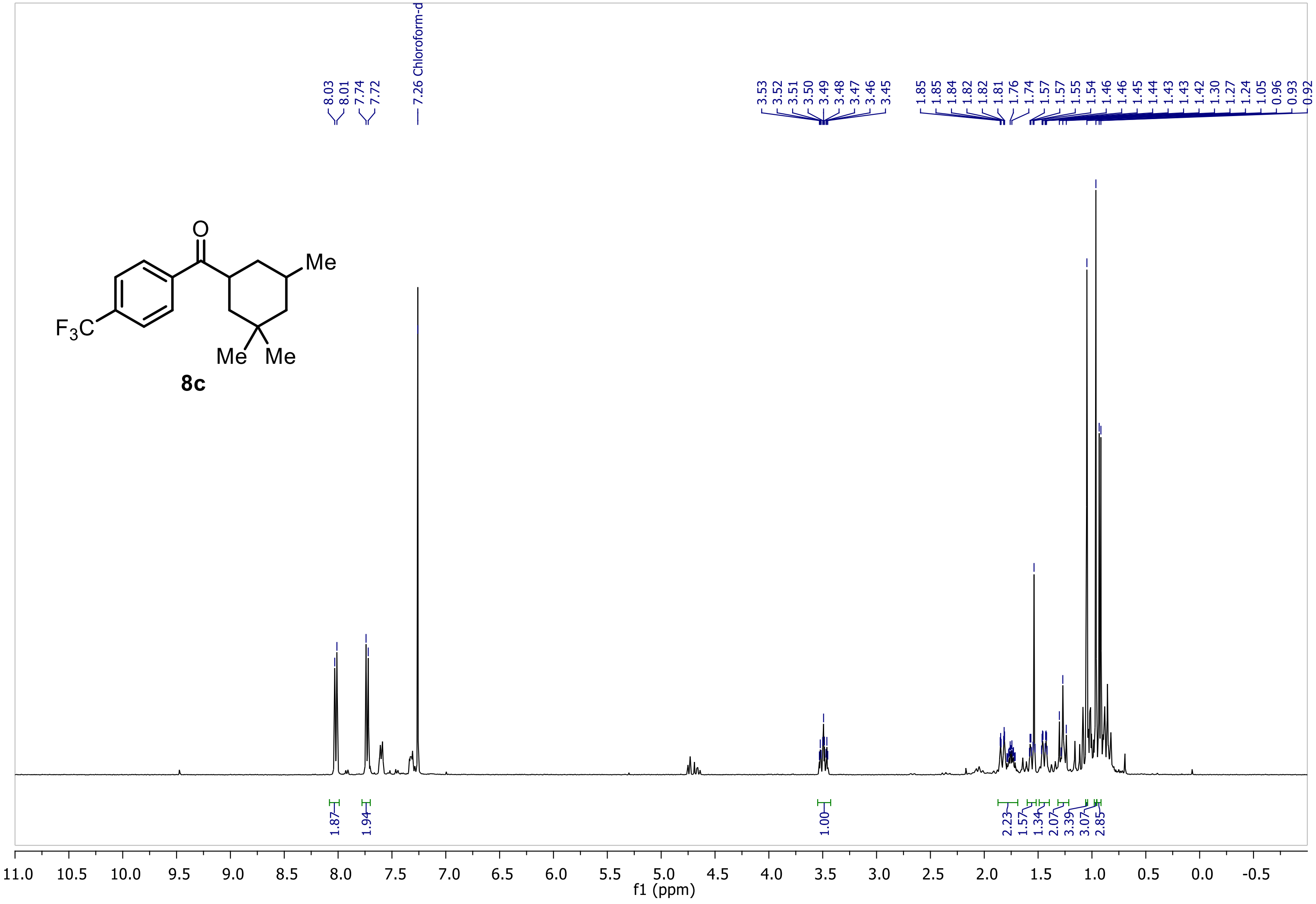

^{19}F NMR (376 MHz, CDCl$_3$)

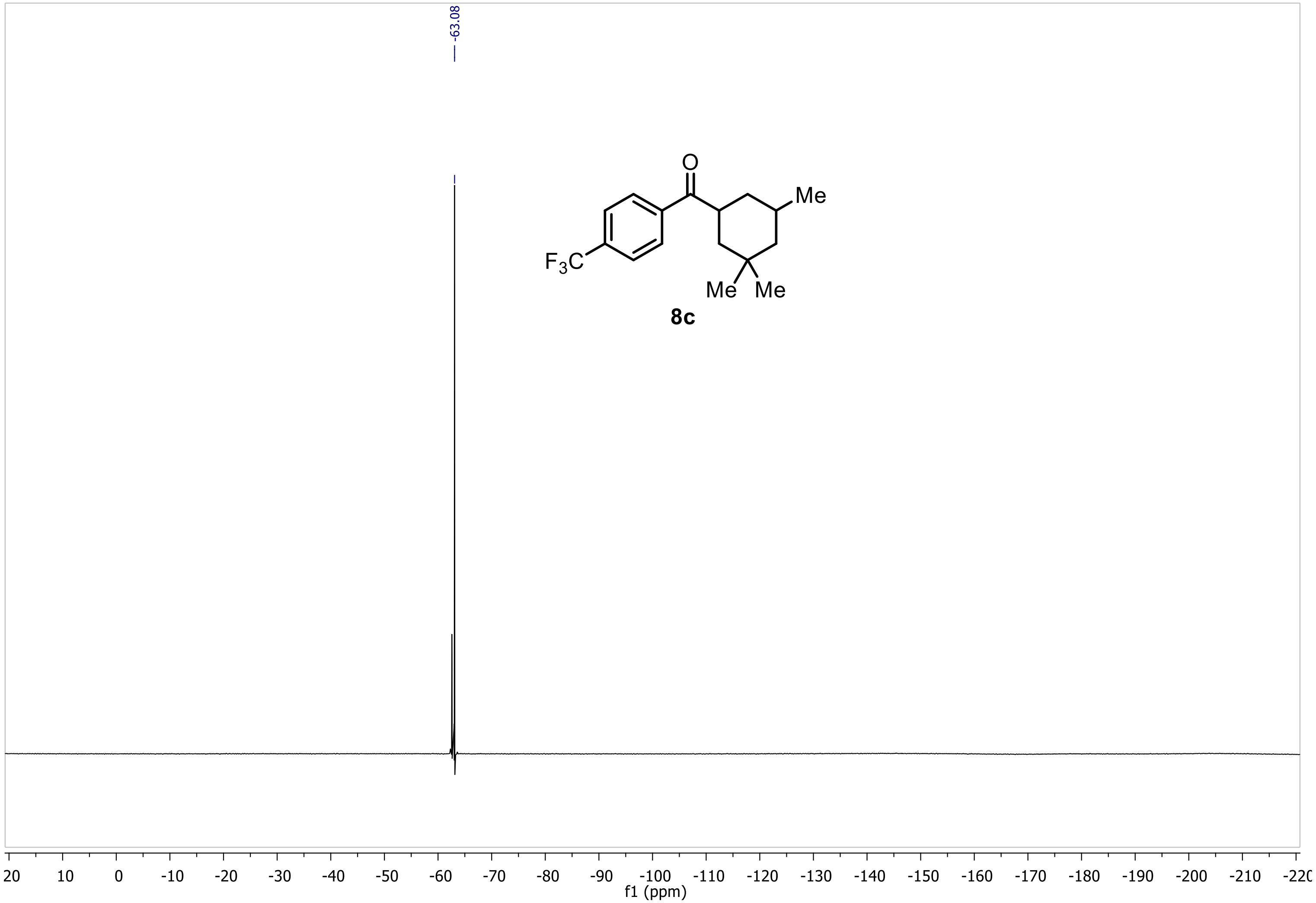

8d – (Cyclopentyl-2,2,5,5-d_4)(4-(trifluoromethyl)phenyl)methanone

^{1}H NMR (400 MHz, CDCl$_3$):

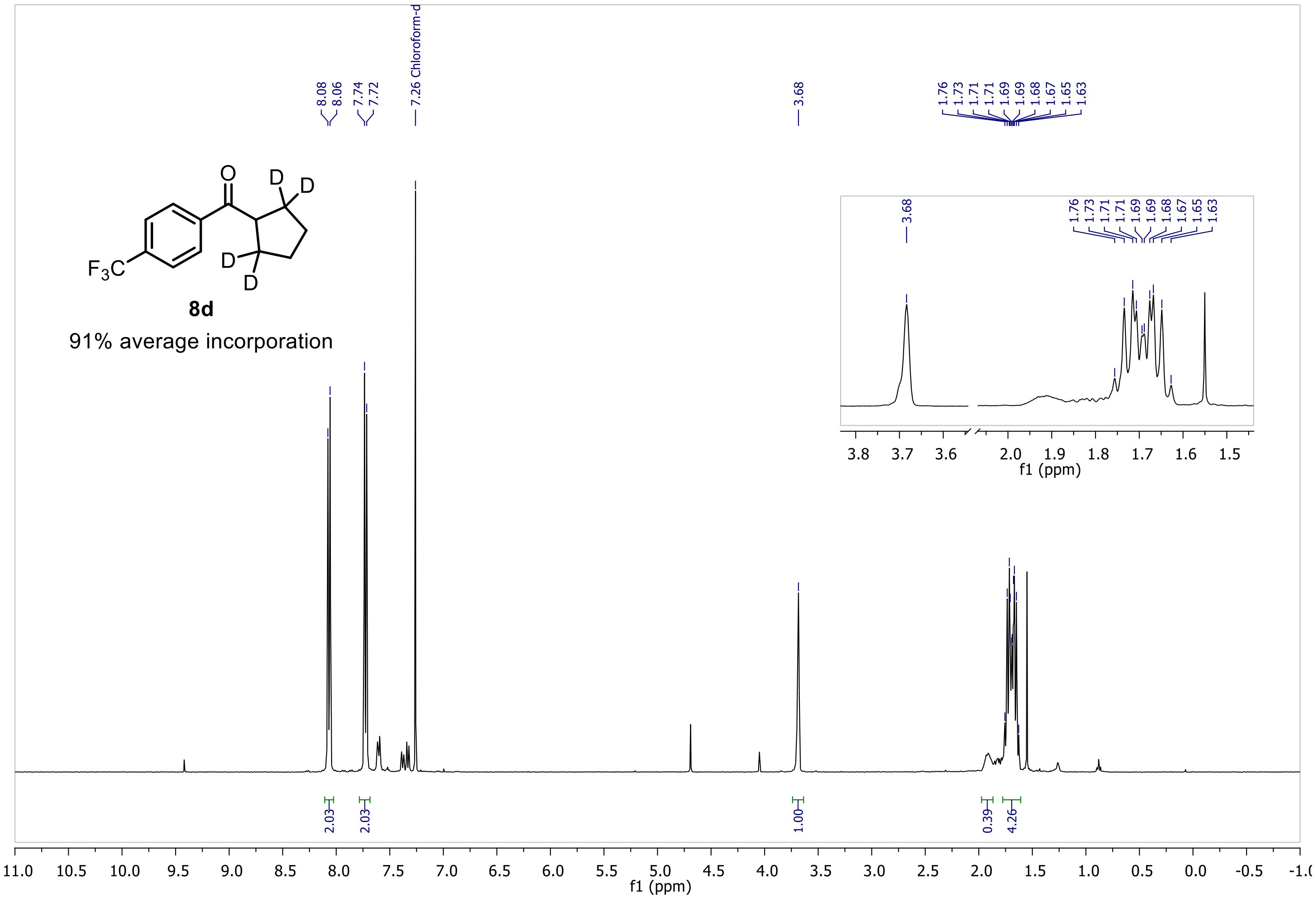

$^{13}C\{^{1}H\}$ NMR (101 MHz, $CDCl_3$):

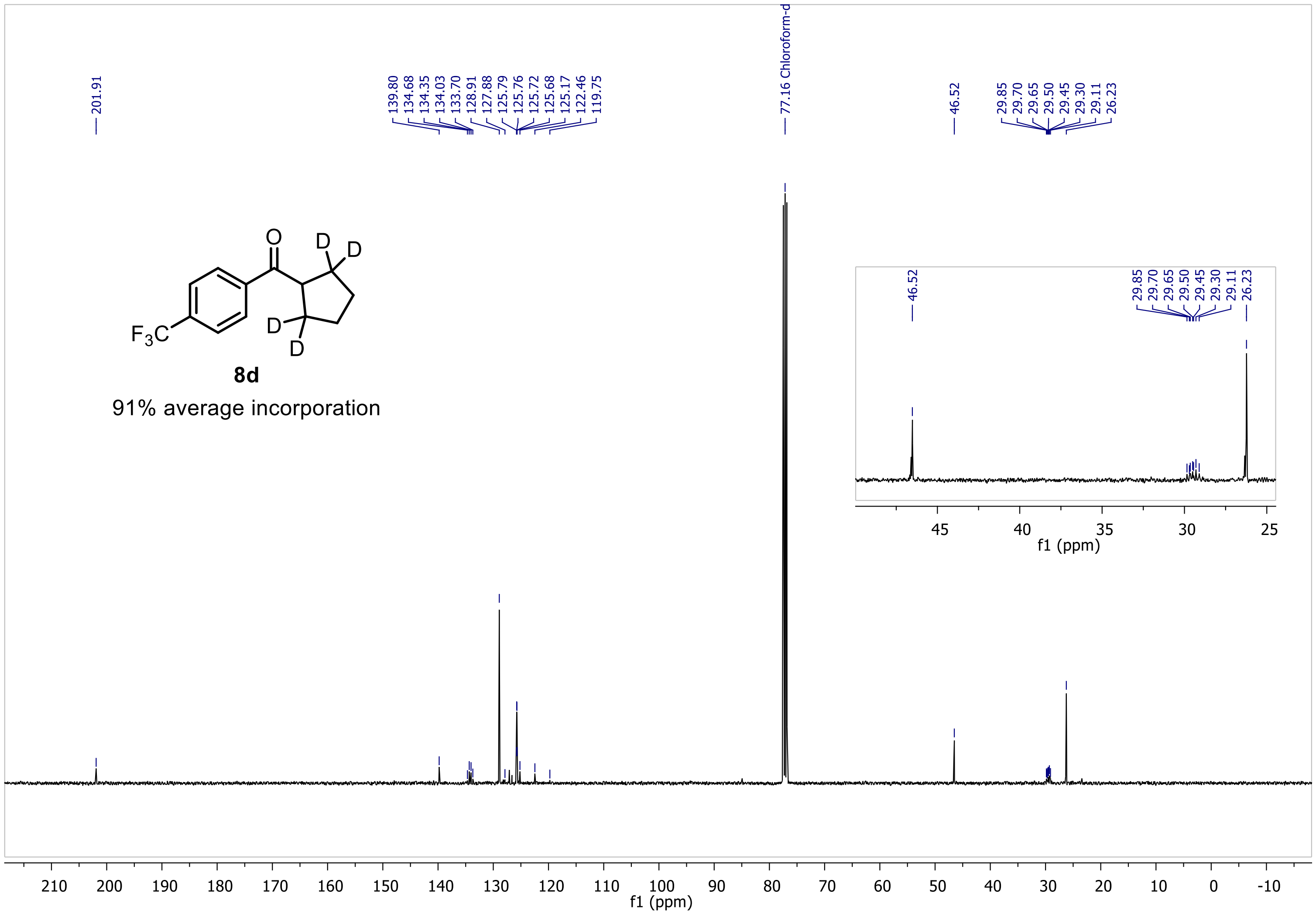

^{19}F NMR (376 MHz, $CDCl_3$):

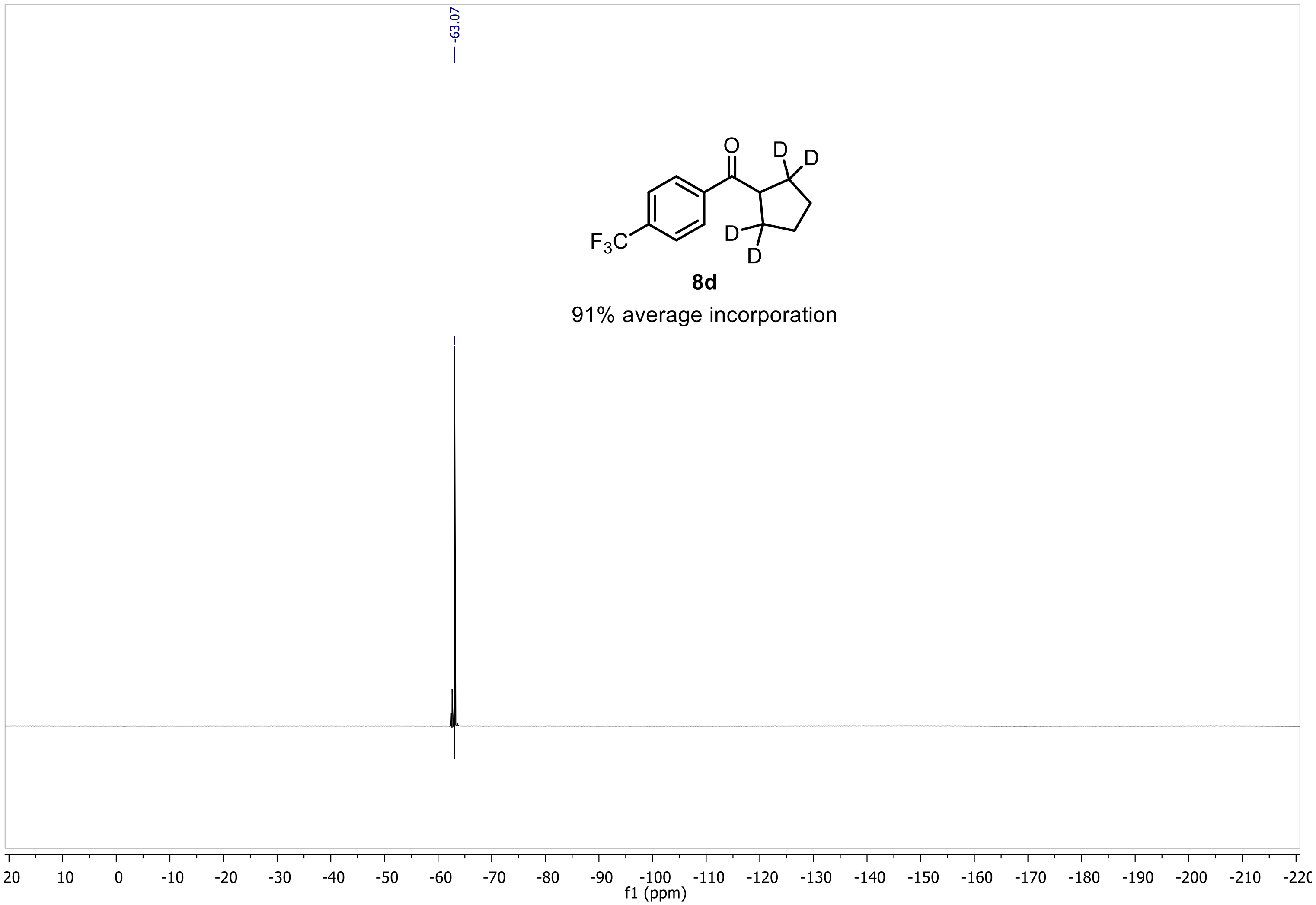

8e – (3-Methylcyclopentyl-2,2,5,5-d_4)[4-(trifluoromethyl)phenyl]methanone

^{1}H NMR (400 MHz, $CDCl_3$):

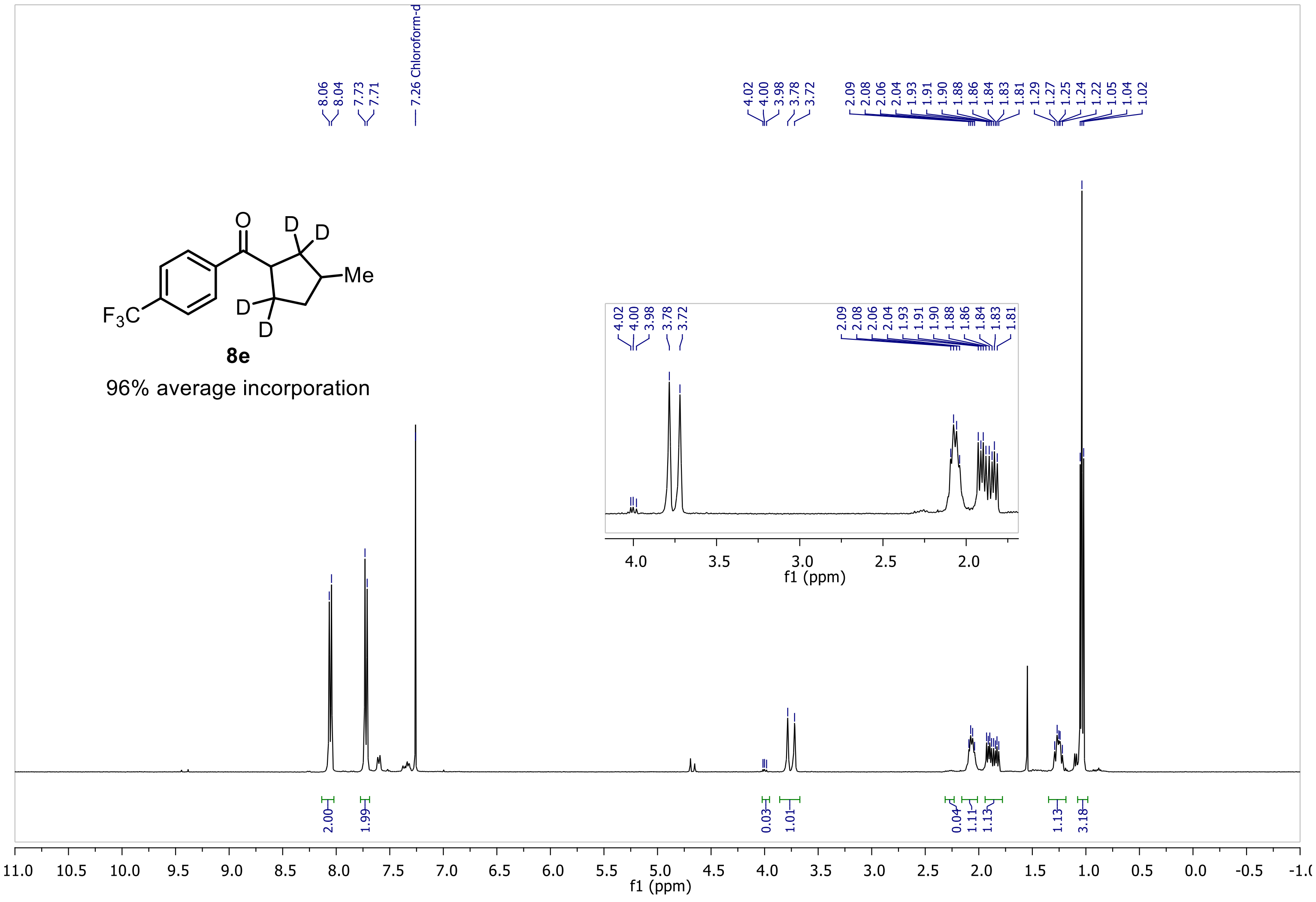

$^{13}C\{^{1}H\}$ NMR (101 MHz, $CDCl_3$):

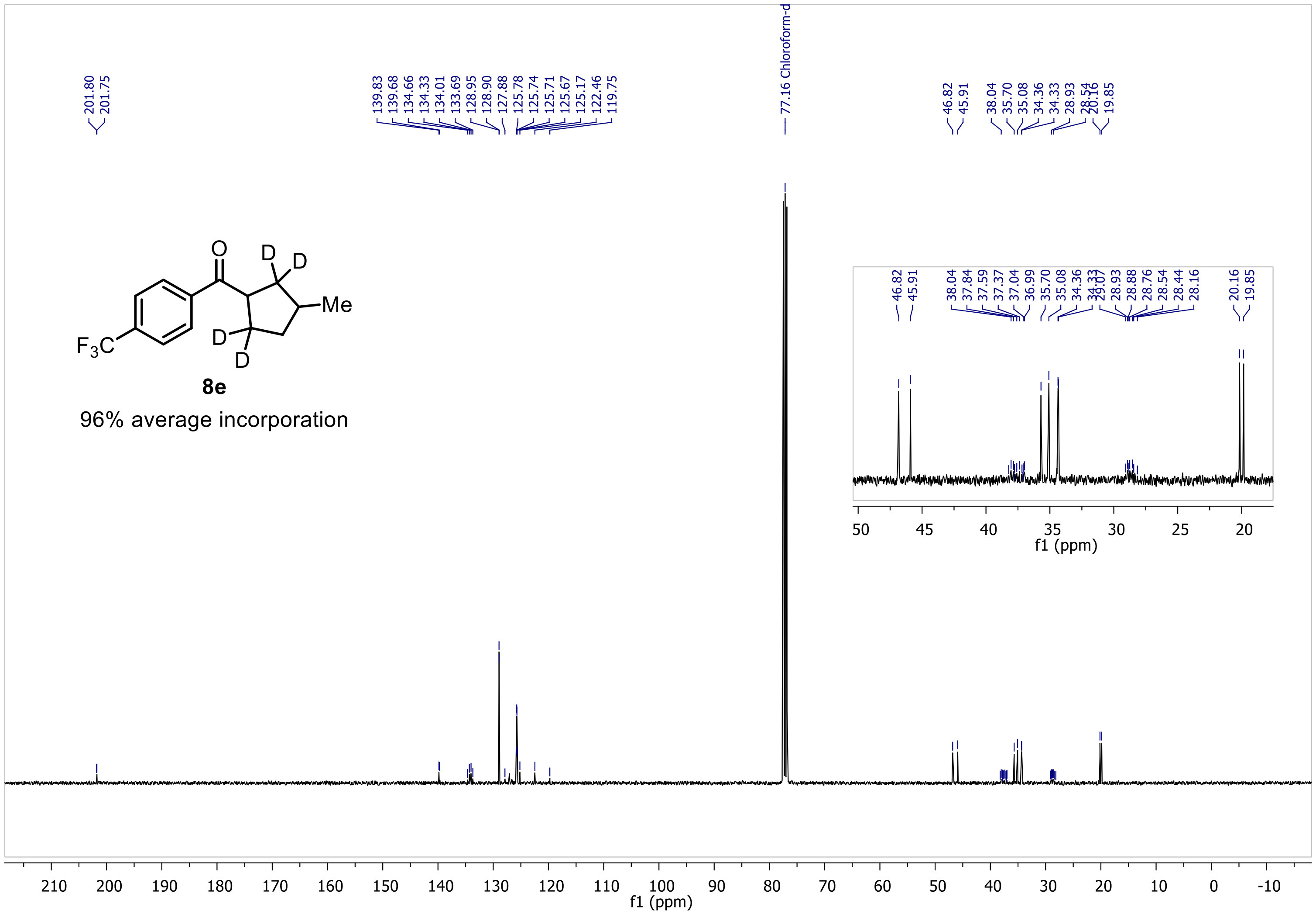

^{19}F NMR (377 MHz, $CDCl_3$):

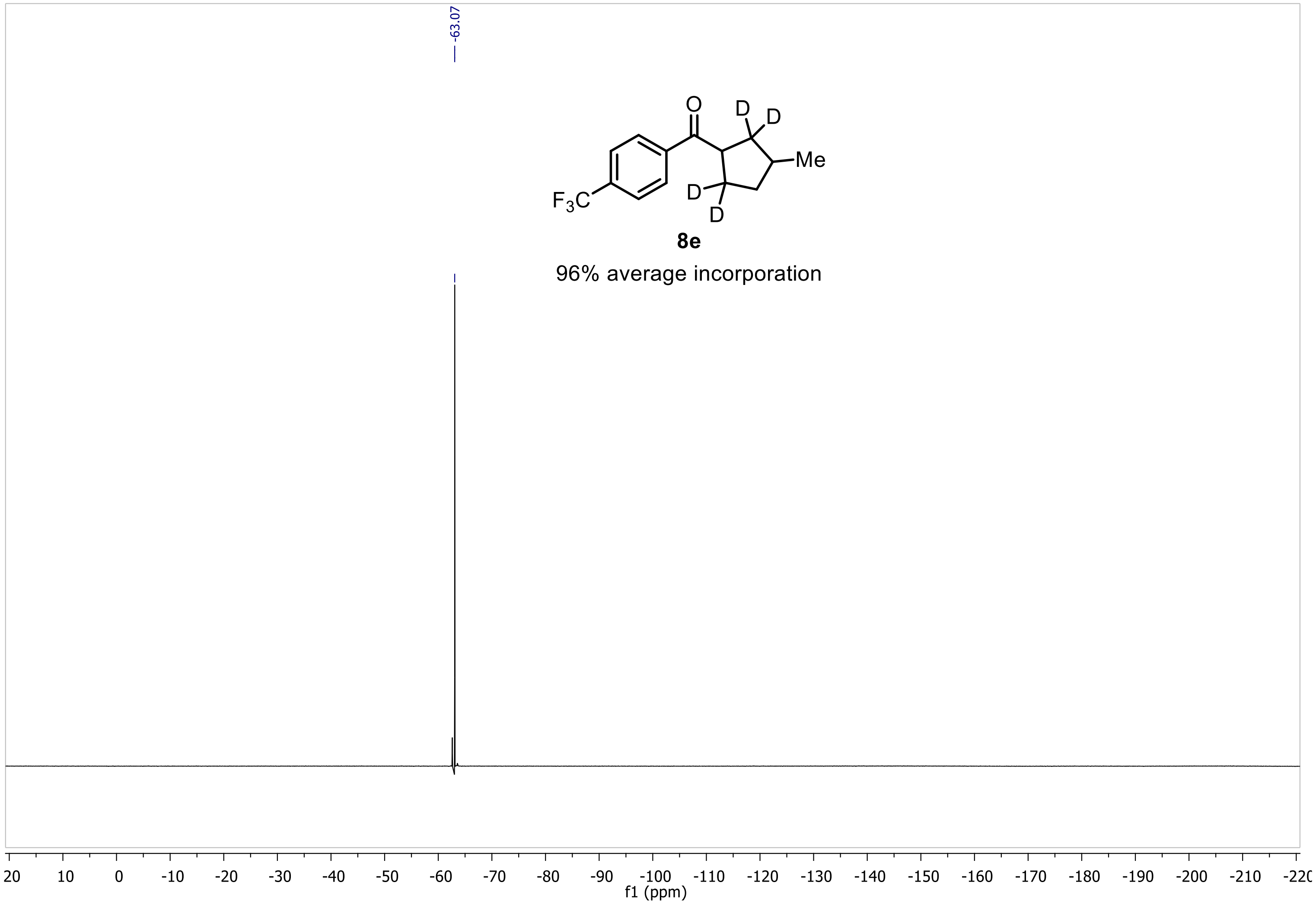

8f – Cyclopentyl[4-(trifluoromethyl)phenyl]methanone

^{1}H NMR (400 MHz, $CDCl_3$)

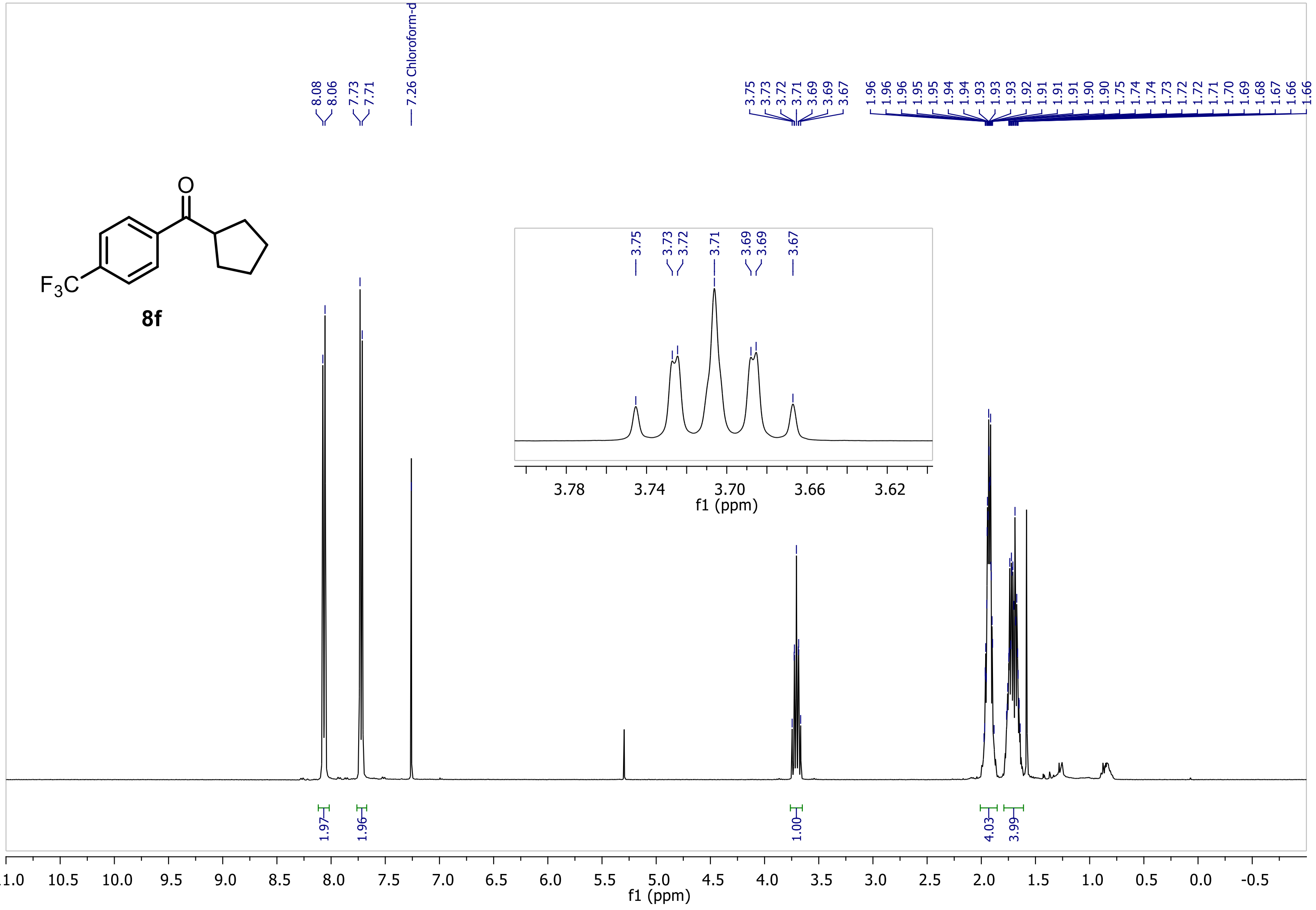

^{19}F NMR (376 MHz, $CDCl_3$)

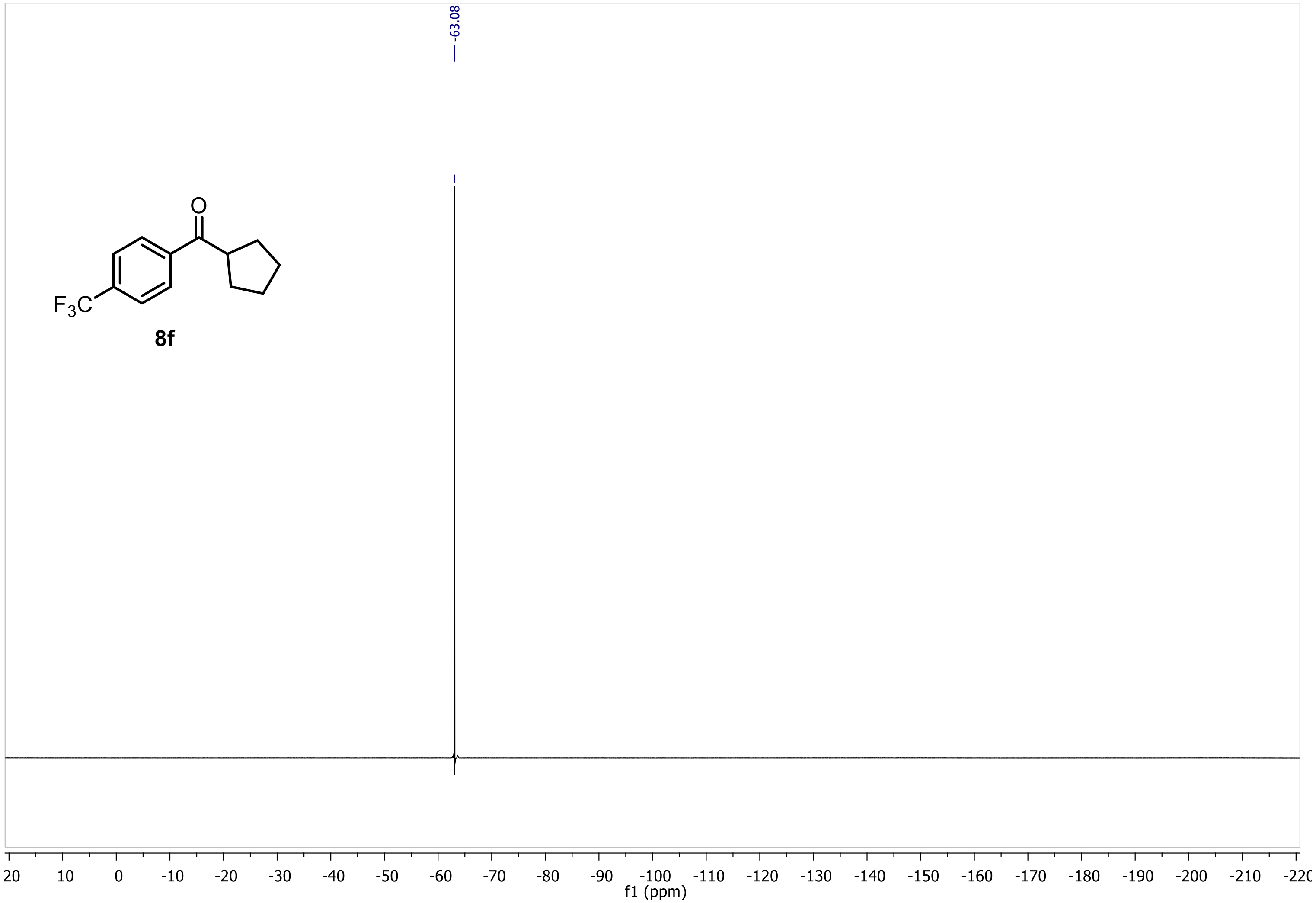

8g – Cyclopentyl[3-(trifluoromethyl)phenyl]methanone

^{1}H NMR (400 MHz, $CDCl_3$)

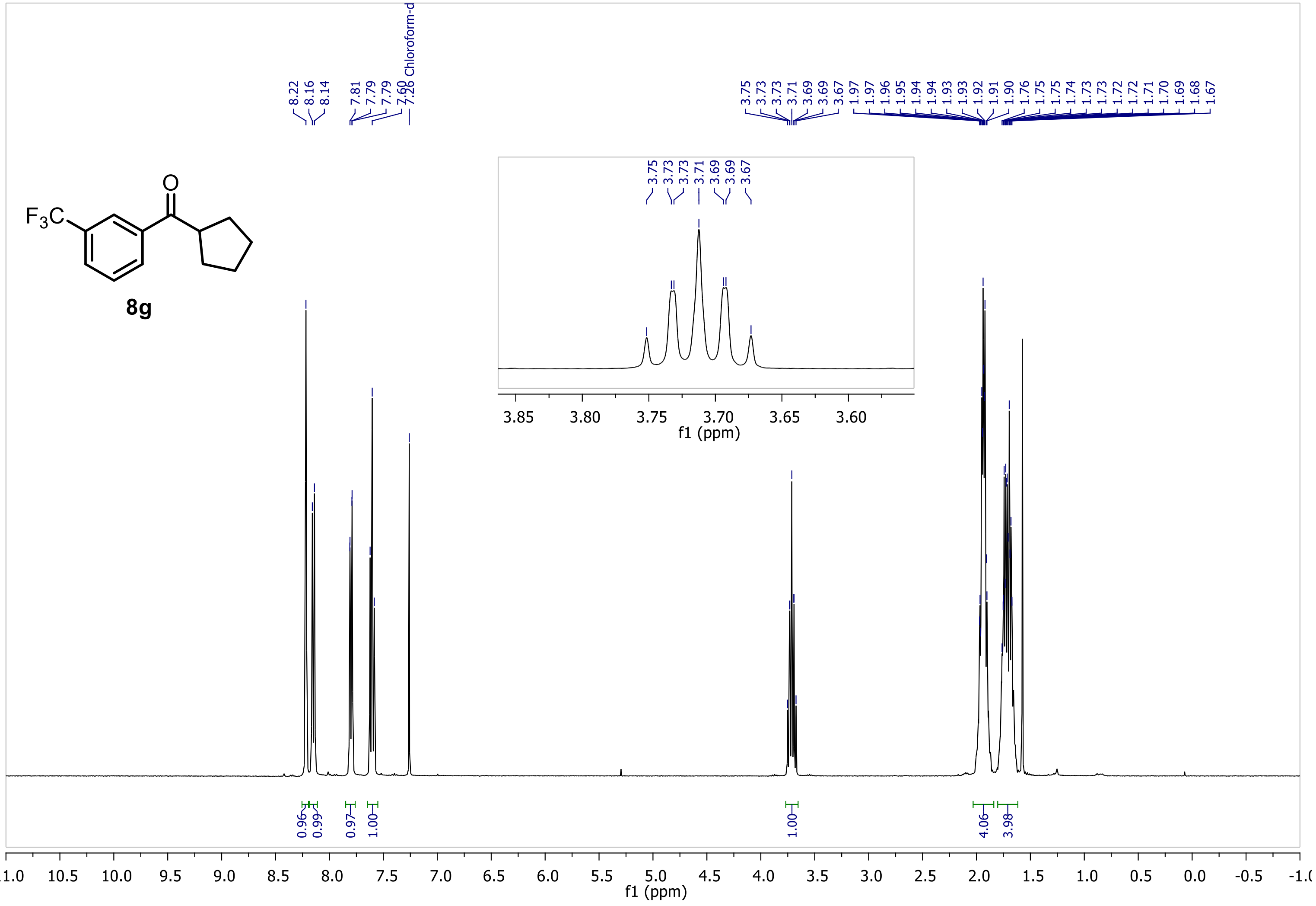

^{19}F NMR (376 MHz, $CDCl_3$)

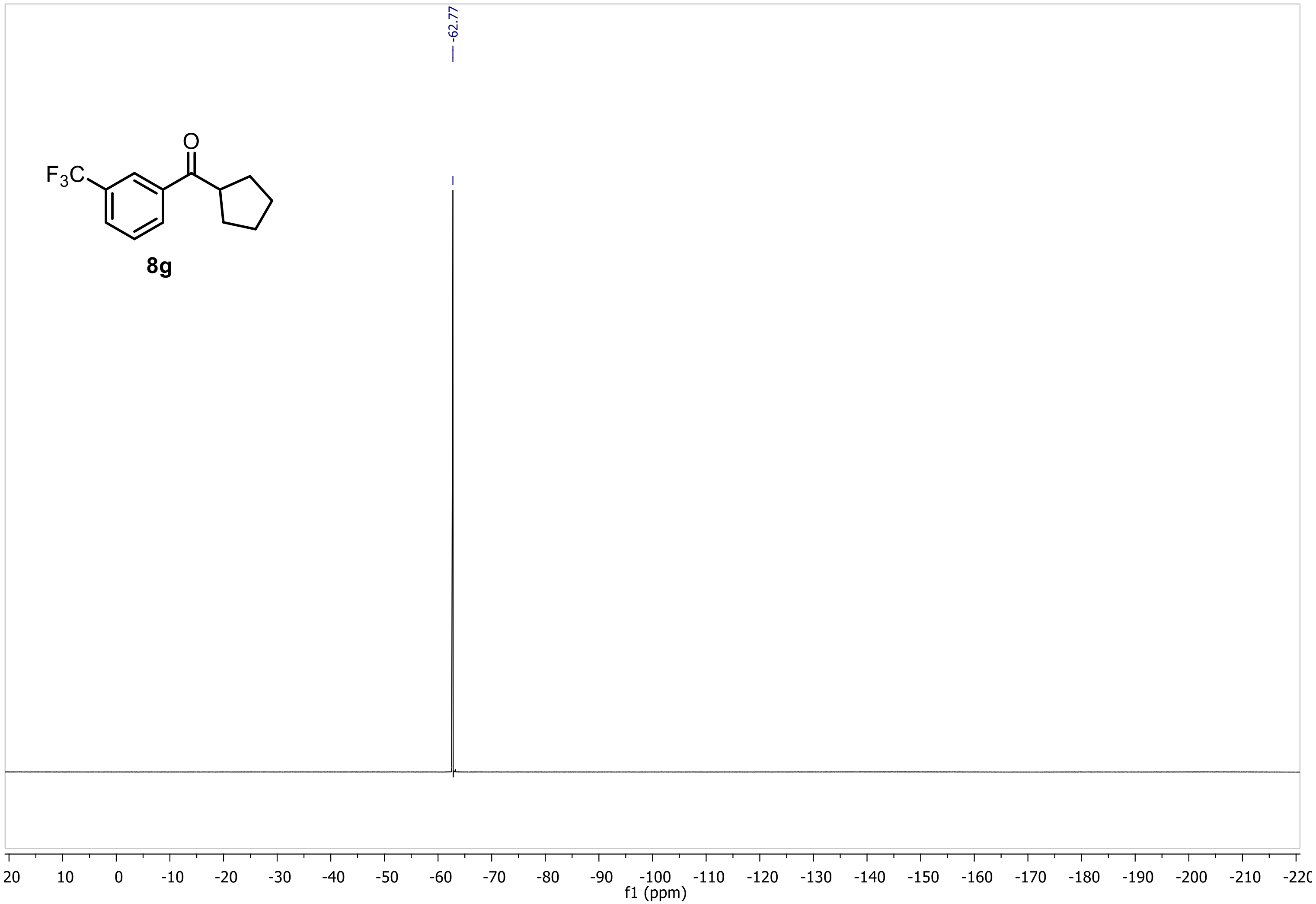

8h – Cyclopentyl[2-(trifluoromethyl)phenyl]methanone

^{1}H NMR (400 MHz, $CDCl_3$)

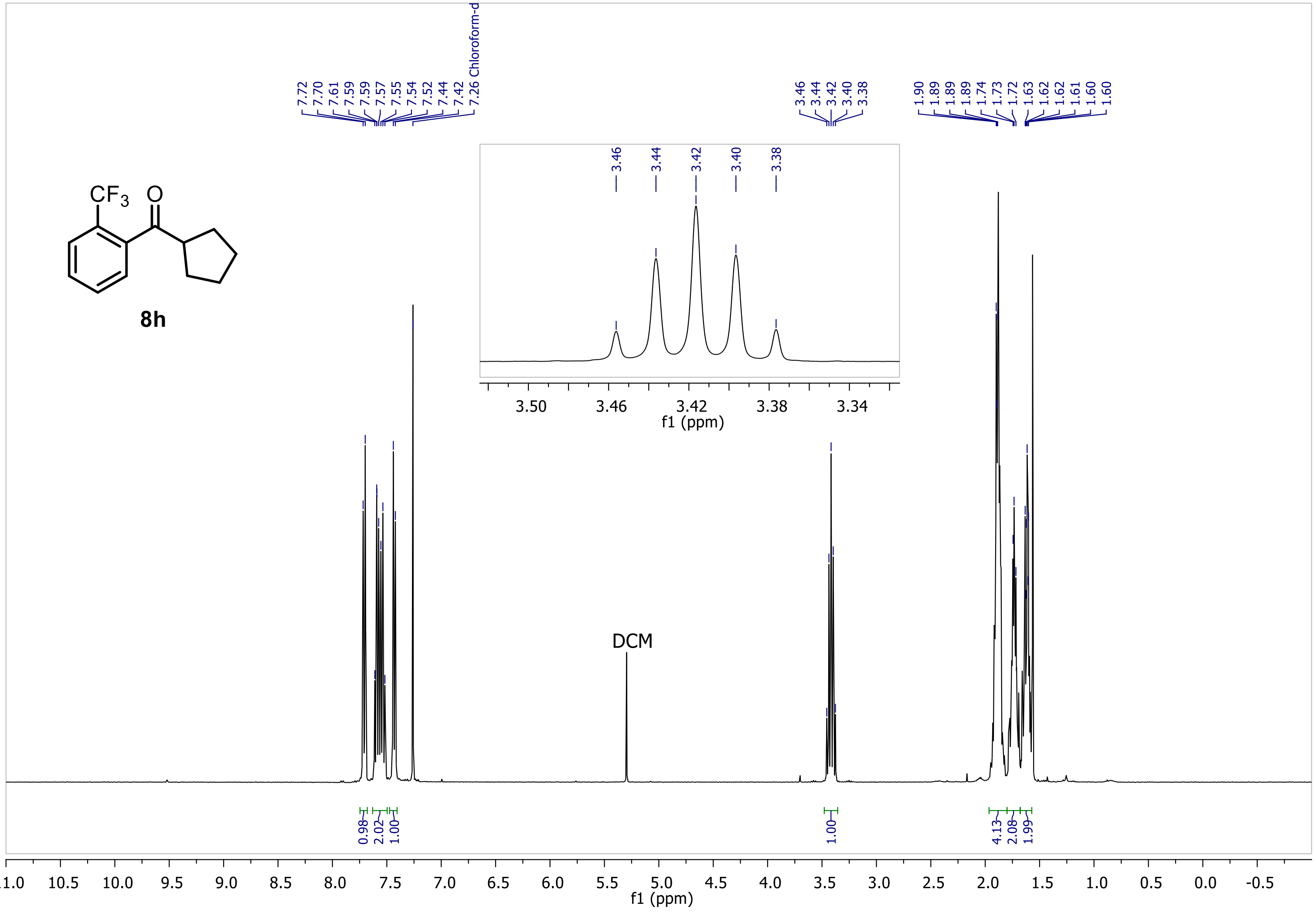

^{19}F NMR (376 MHz, CDCl$_3$)

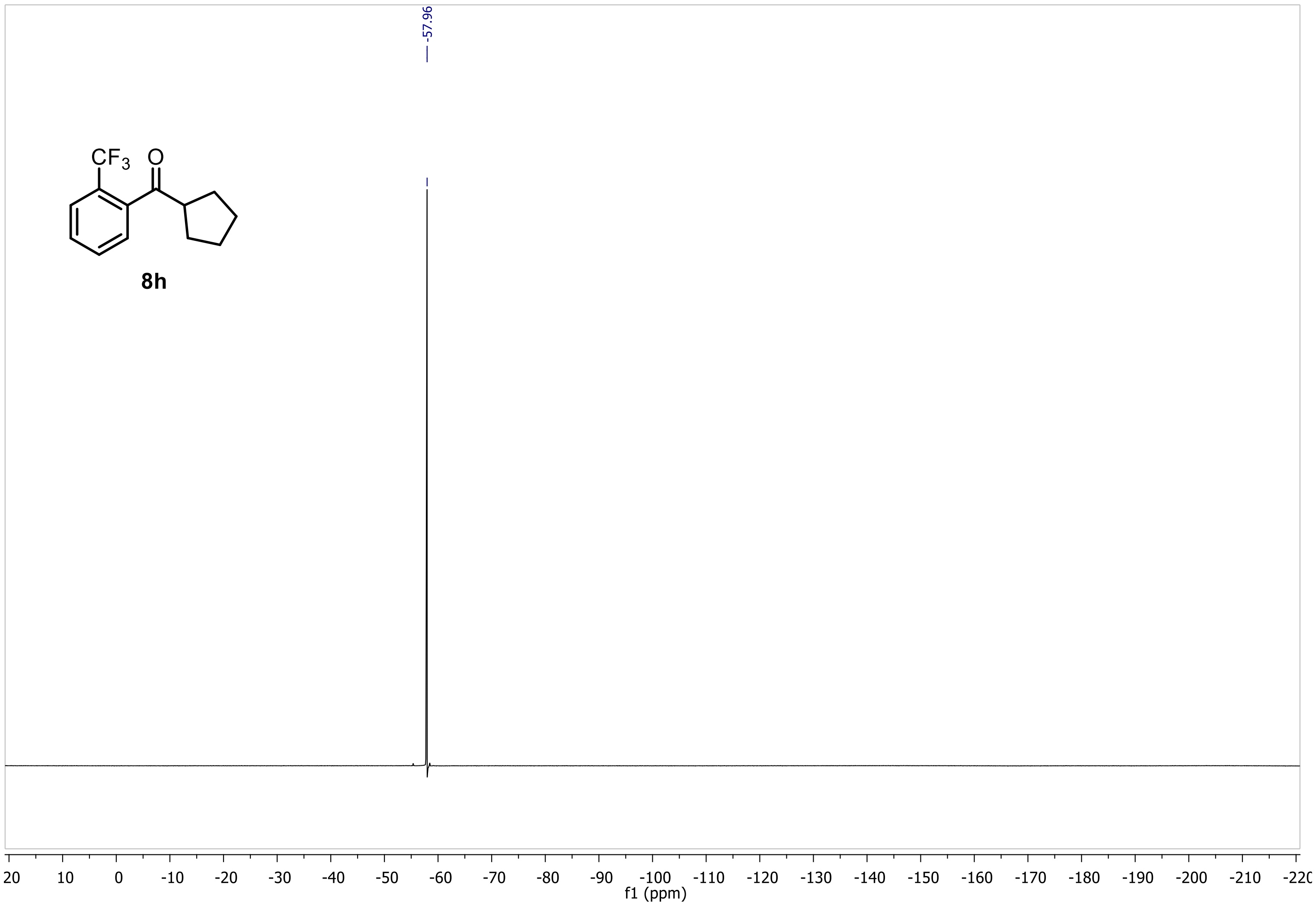

8i – 4-(Cyclopentanecarbonyl)benzonitrile

^{1}H NMR (400 MHz, $CDCl_3$)

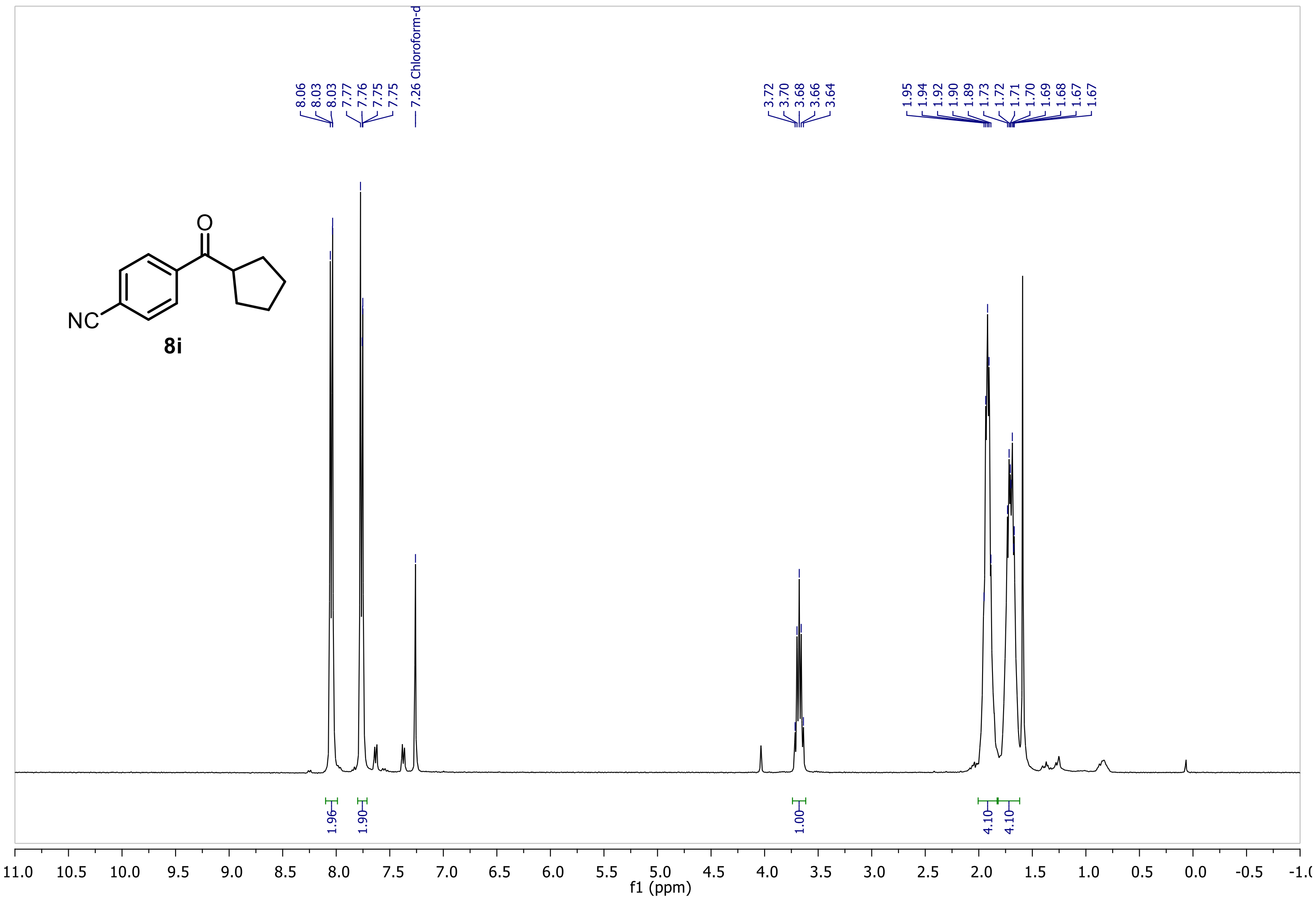

8j – Methyl 4-(cyclopentanecarbonyl)benzoate

^{1}H NMR (400 MHz, $CDCl_3$)

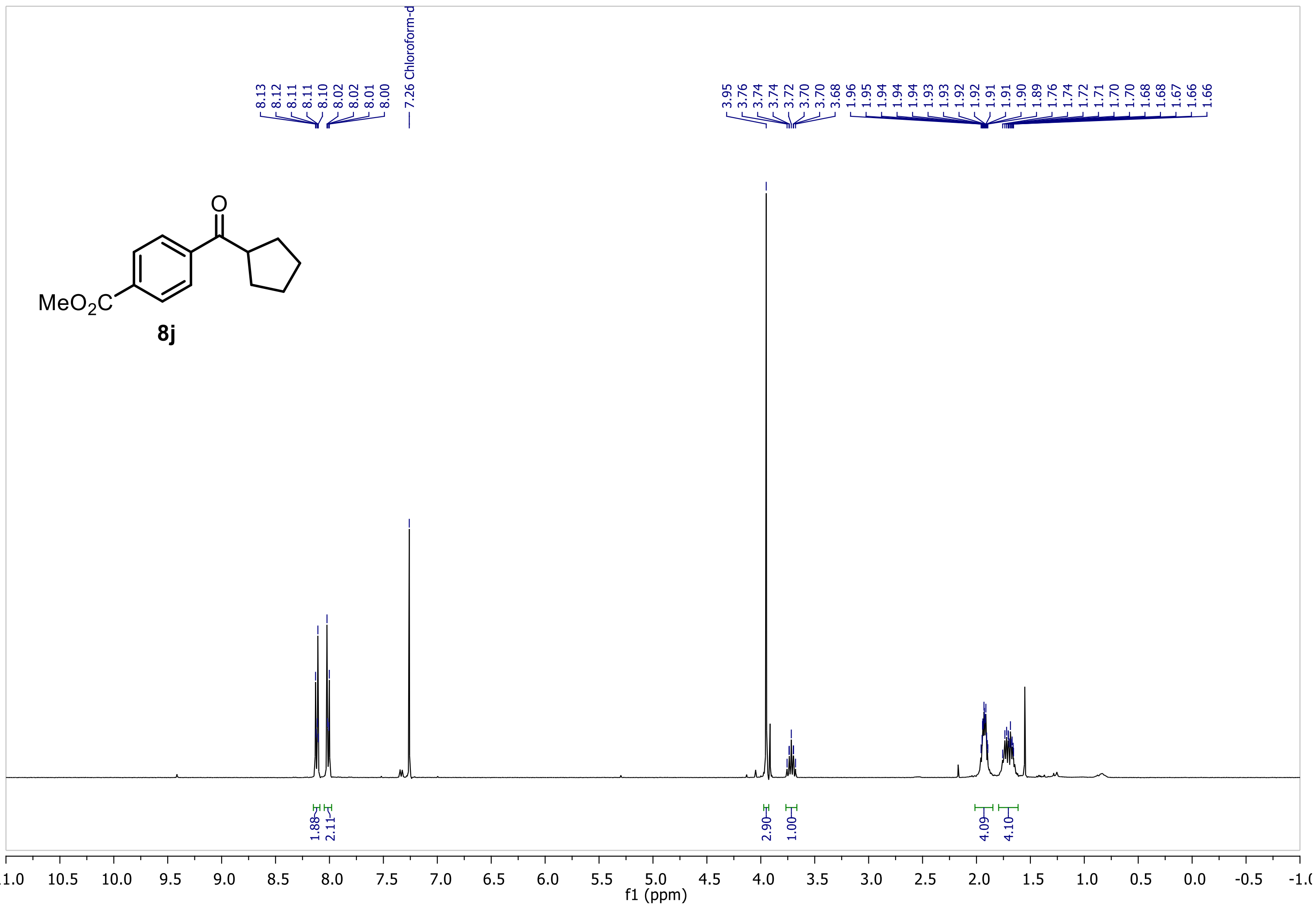
8j

8k – Cyclopentyl(2,6-dichlorophenyl)methanone

^{1}H NMR (400 MHz, $CDCl_3$)

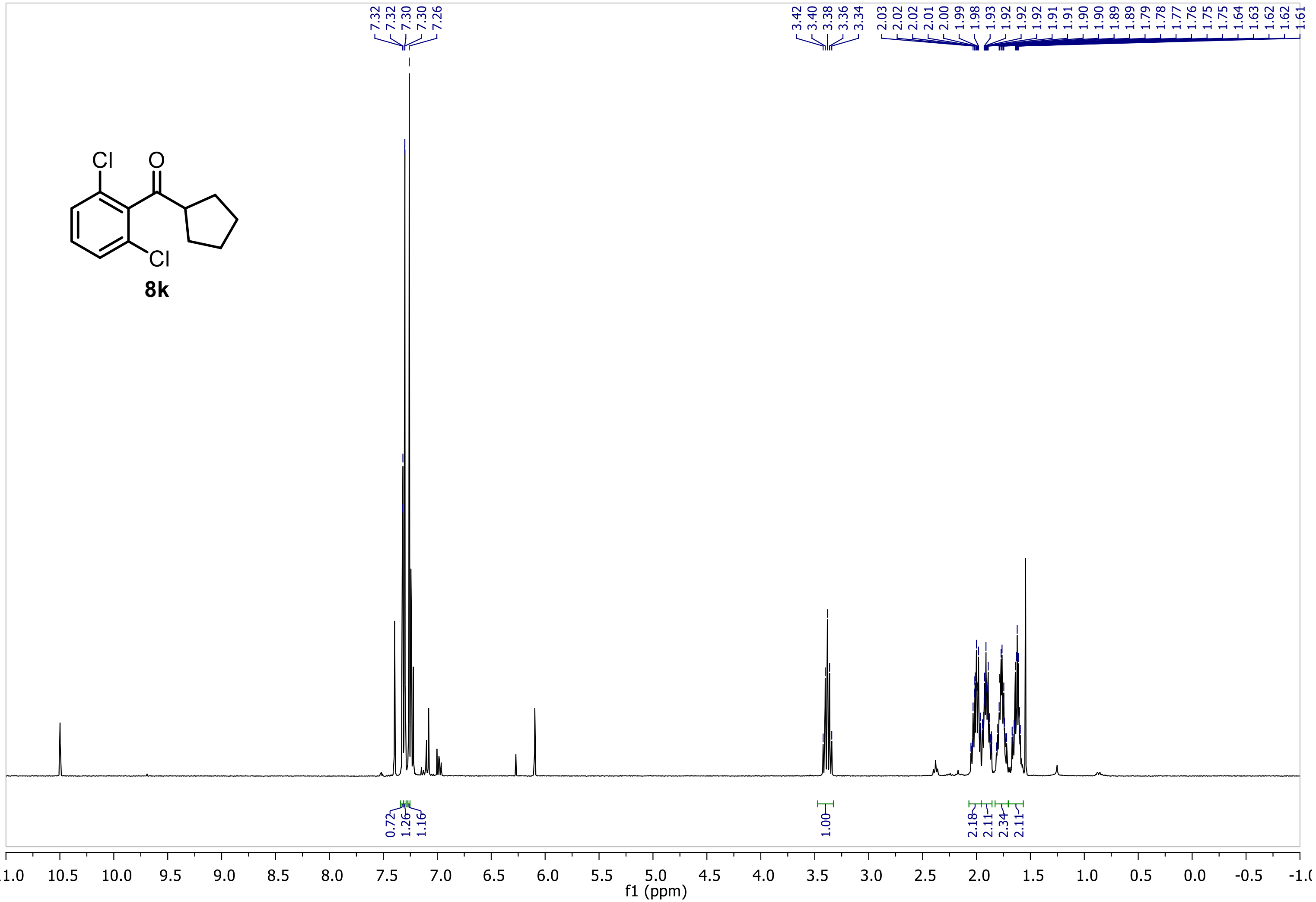

8l – (4-Bromophenyl)(cyclopentyl)methanone

^{1}H NMR (400 MHz, $CDCl_3$)

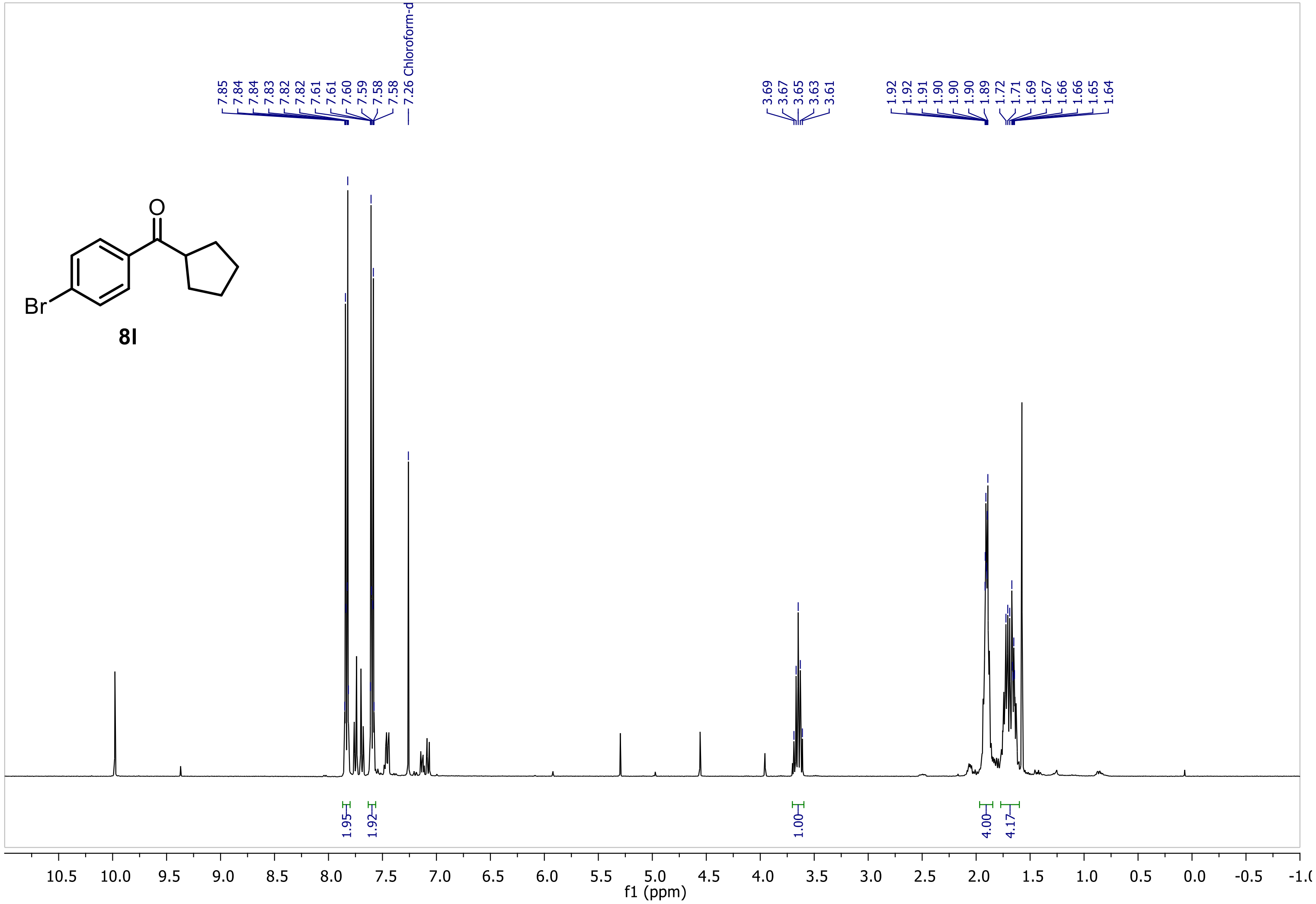

8m – Cyclopentyl(phenyl)methanone

^{1}H NMR (600 MHz, $CDCl_3$)

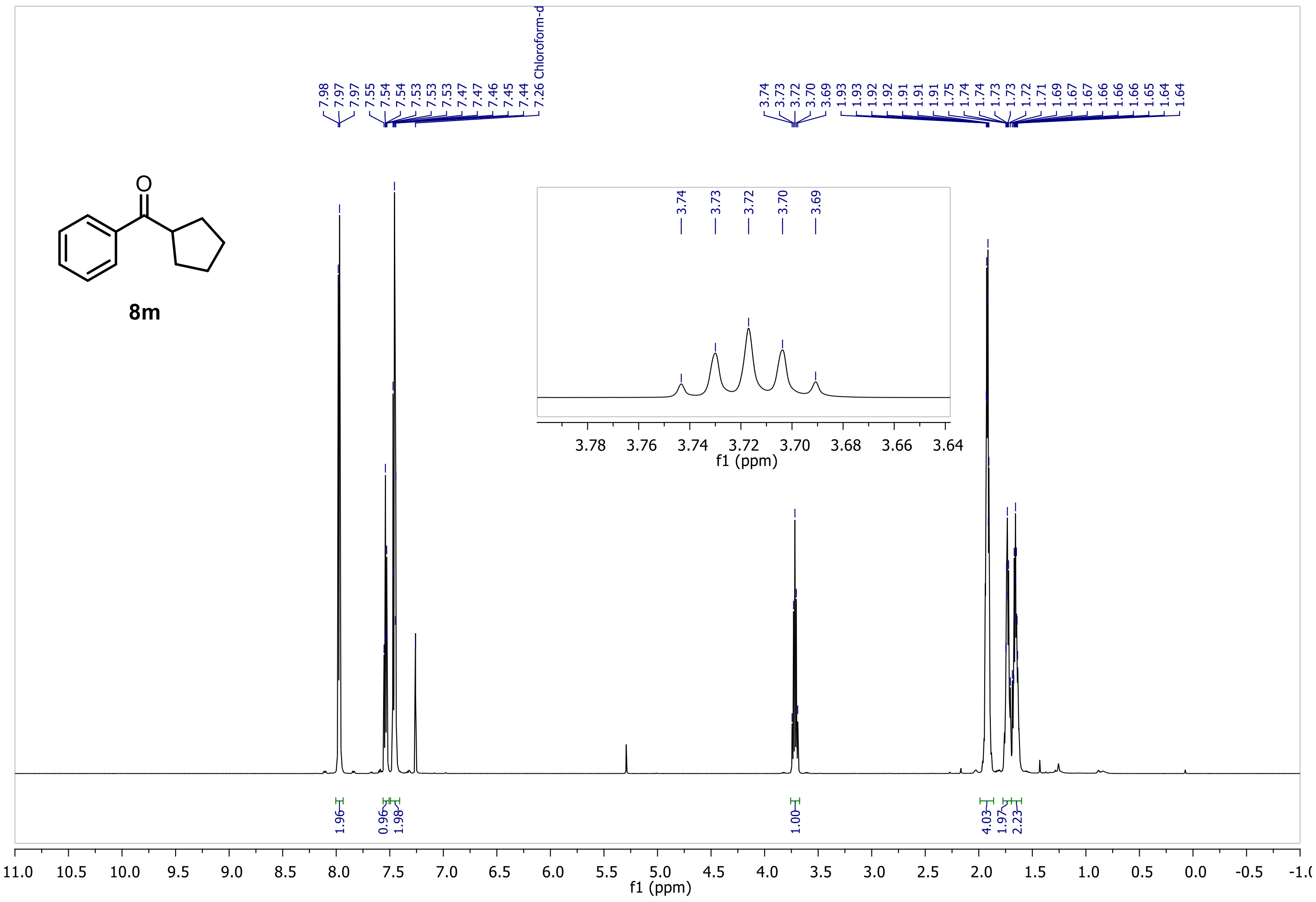

8n – cyclopentyl(naphthalen-2-yl)methanone

^{1}H NMR (400 MHz, $CDCl_3$)

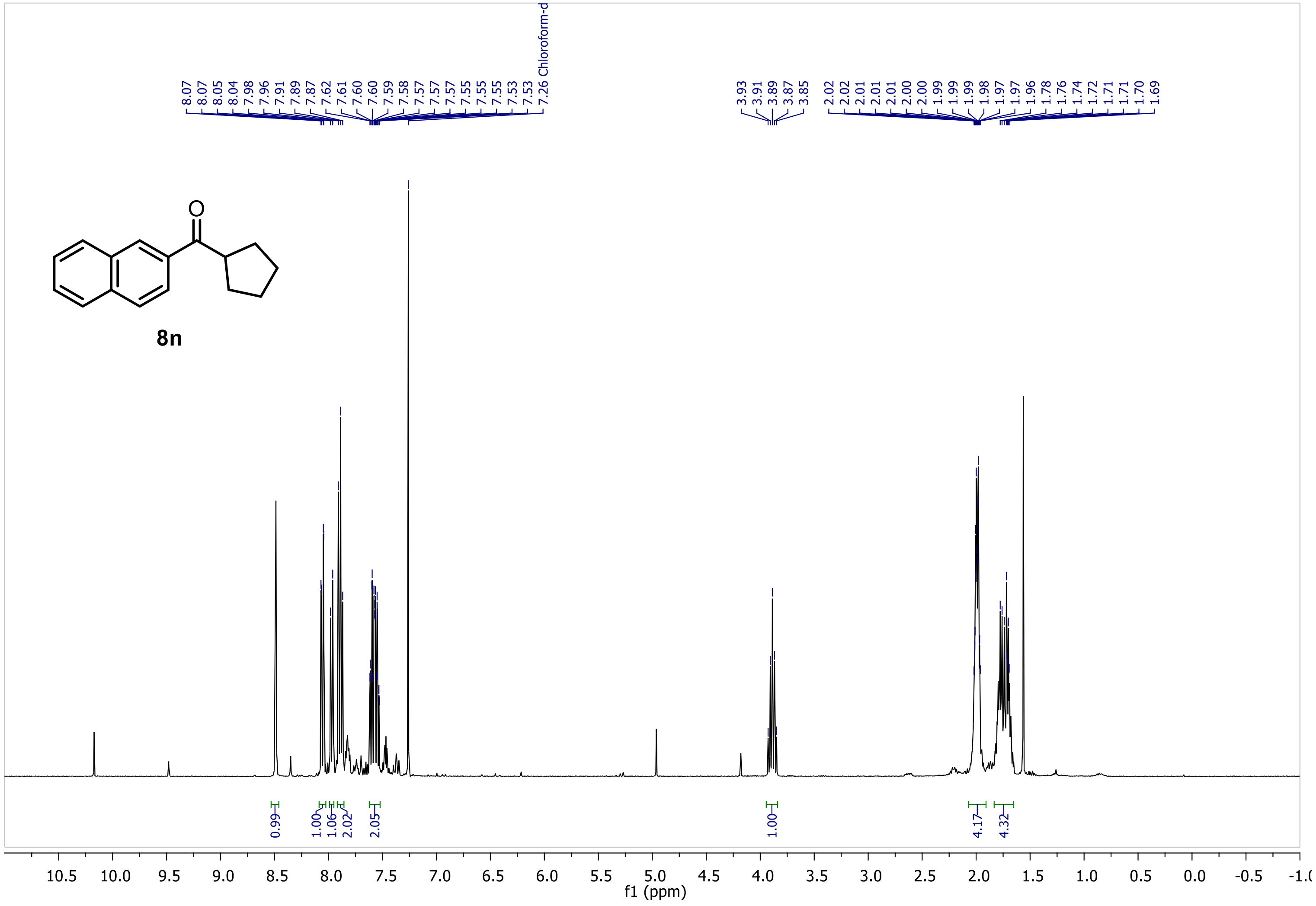

8o – Cyclohexyl[4-(trifluoromethyl)phenyl]methanone

^{1}H NMR (400 MHz, $CDCl_3$)

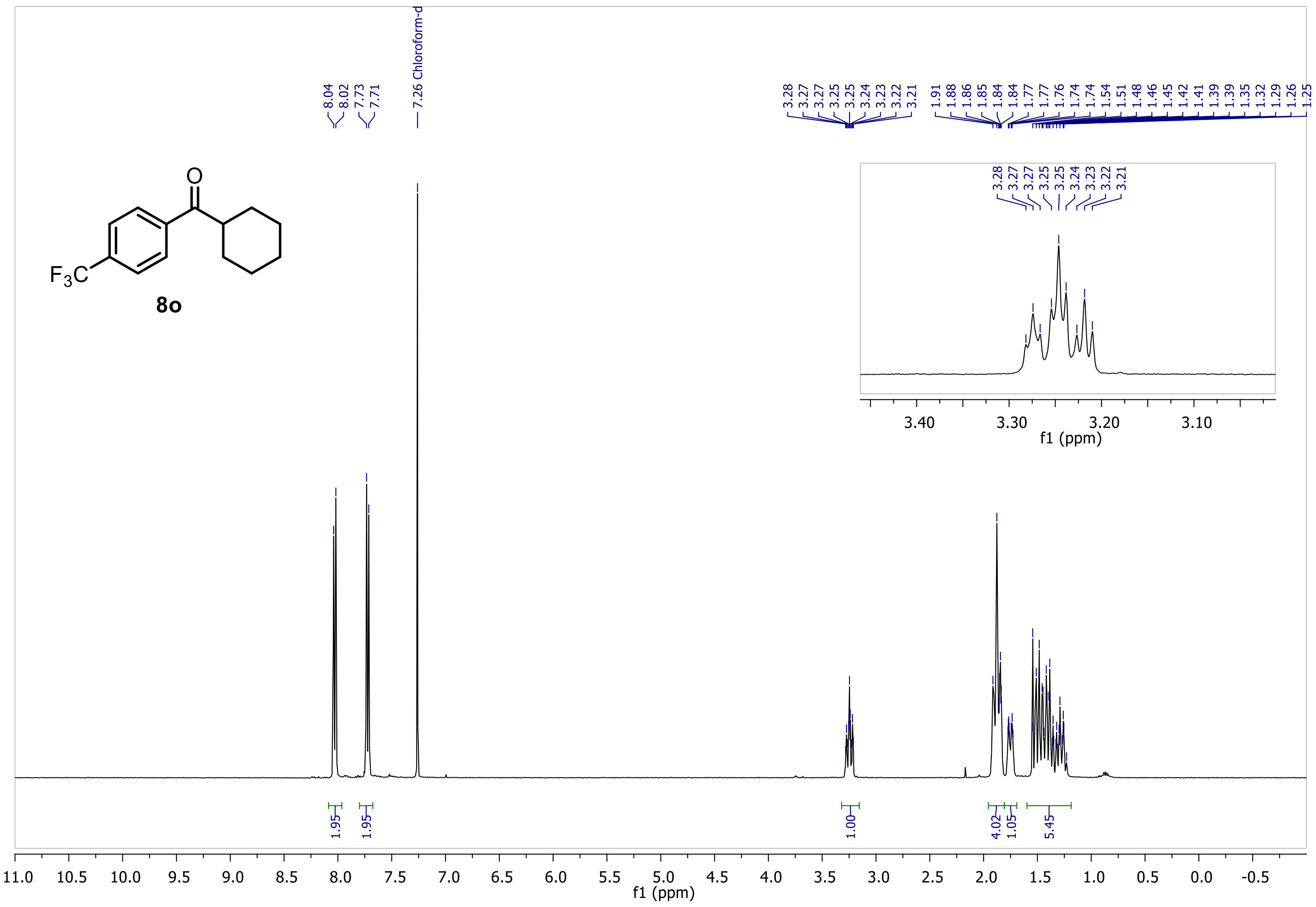

^{19}F NMR (376 MHz, $CDCl_3$):

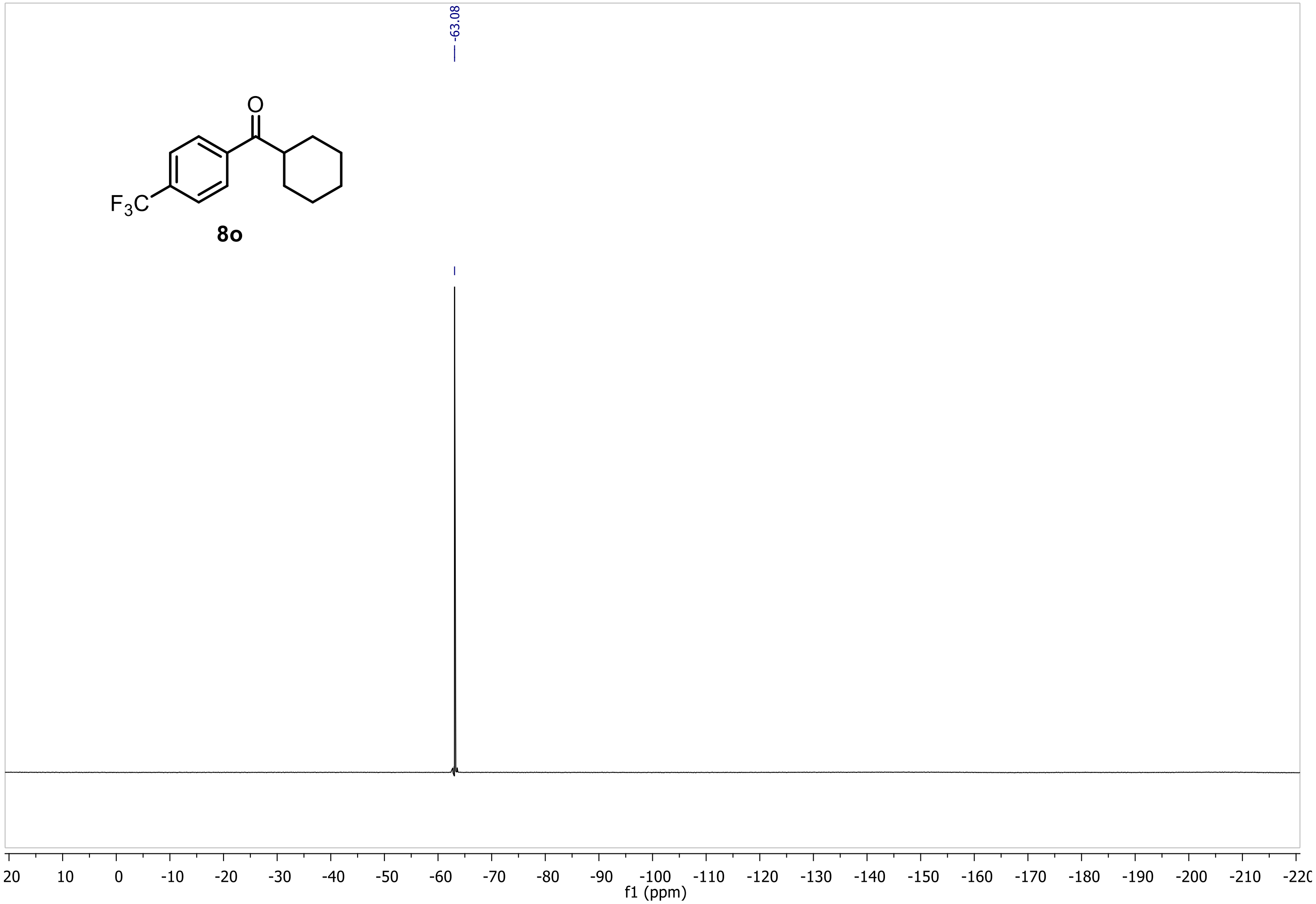

8p – Benzo[*b*]thiophen-2-yl(cyclohexyl)methanone

^{1}H NMR (400 MHz, CDCl$_3$)

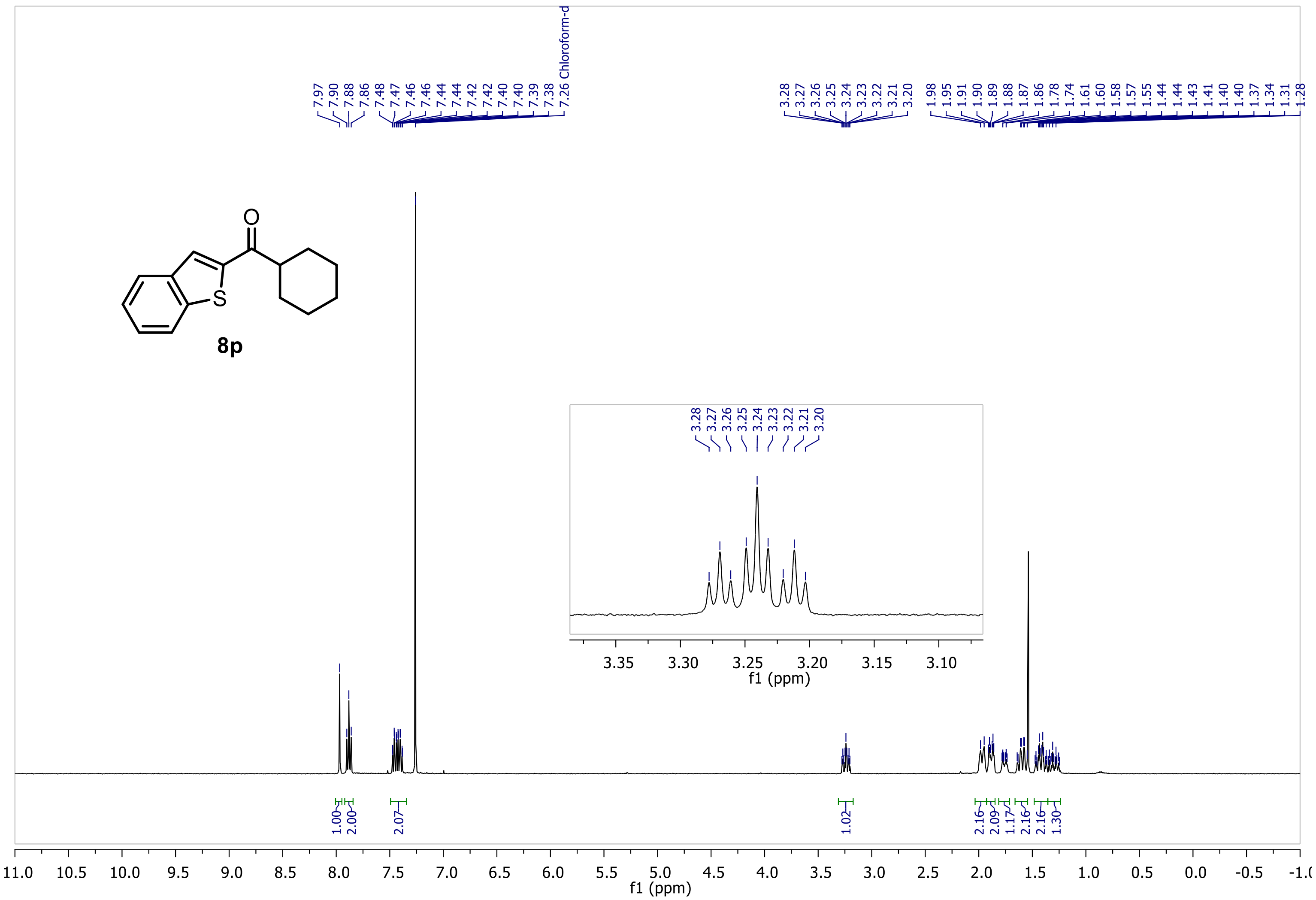

8q – [3-(*tert*-Butyl)cyclopentyl][4-(trifluoromethyl)phenyl]methanone

^{1}H NMR (400 MHz, $CDCl_3$)

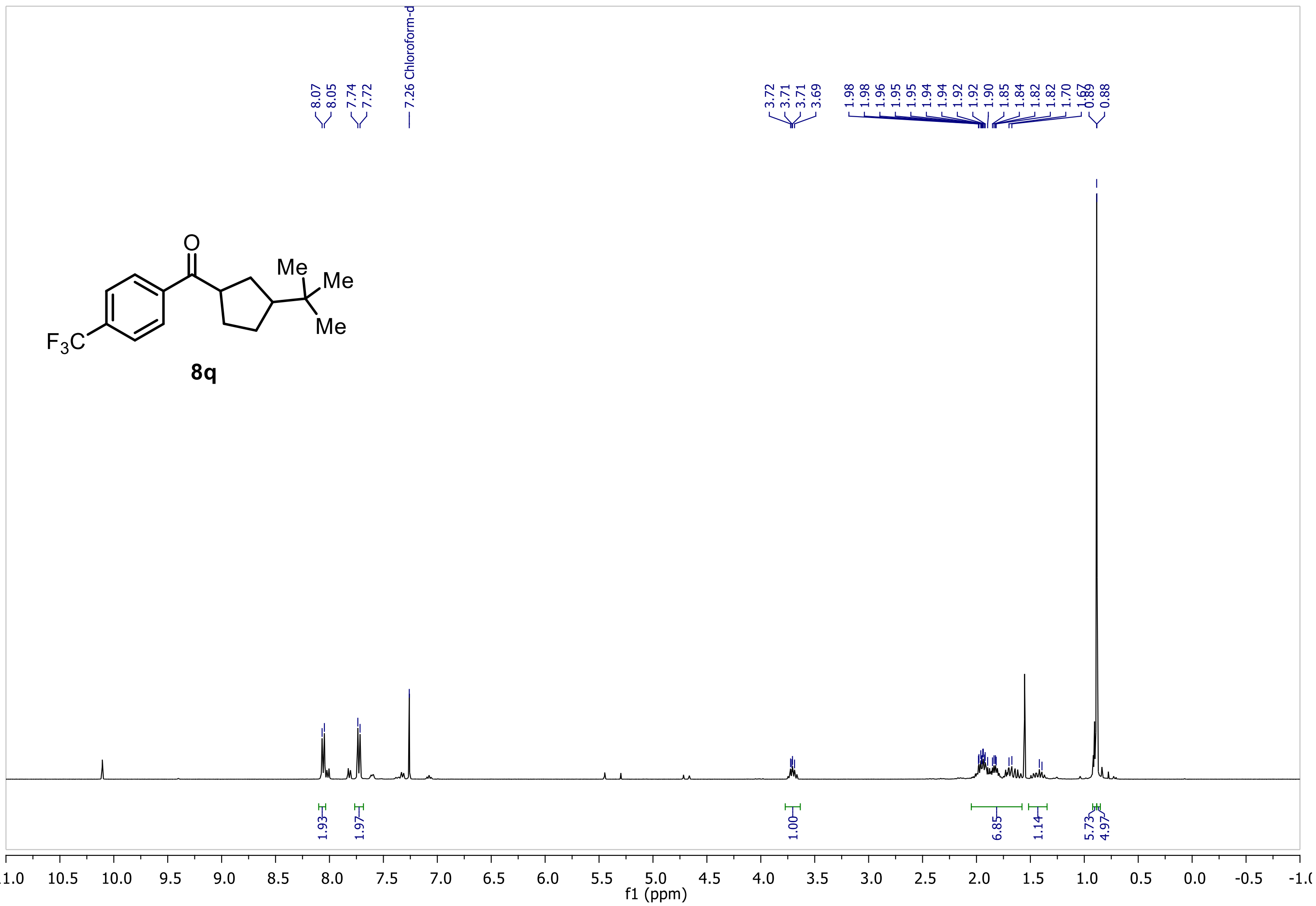

^{19}F NMR (376 MHz, CDCl$_3$)

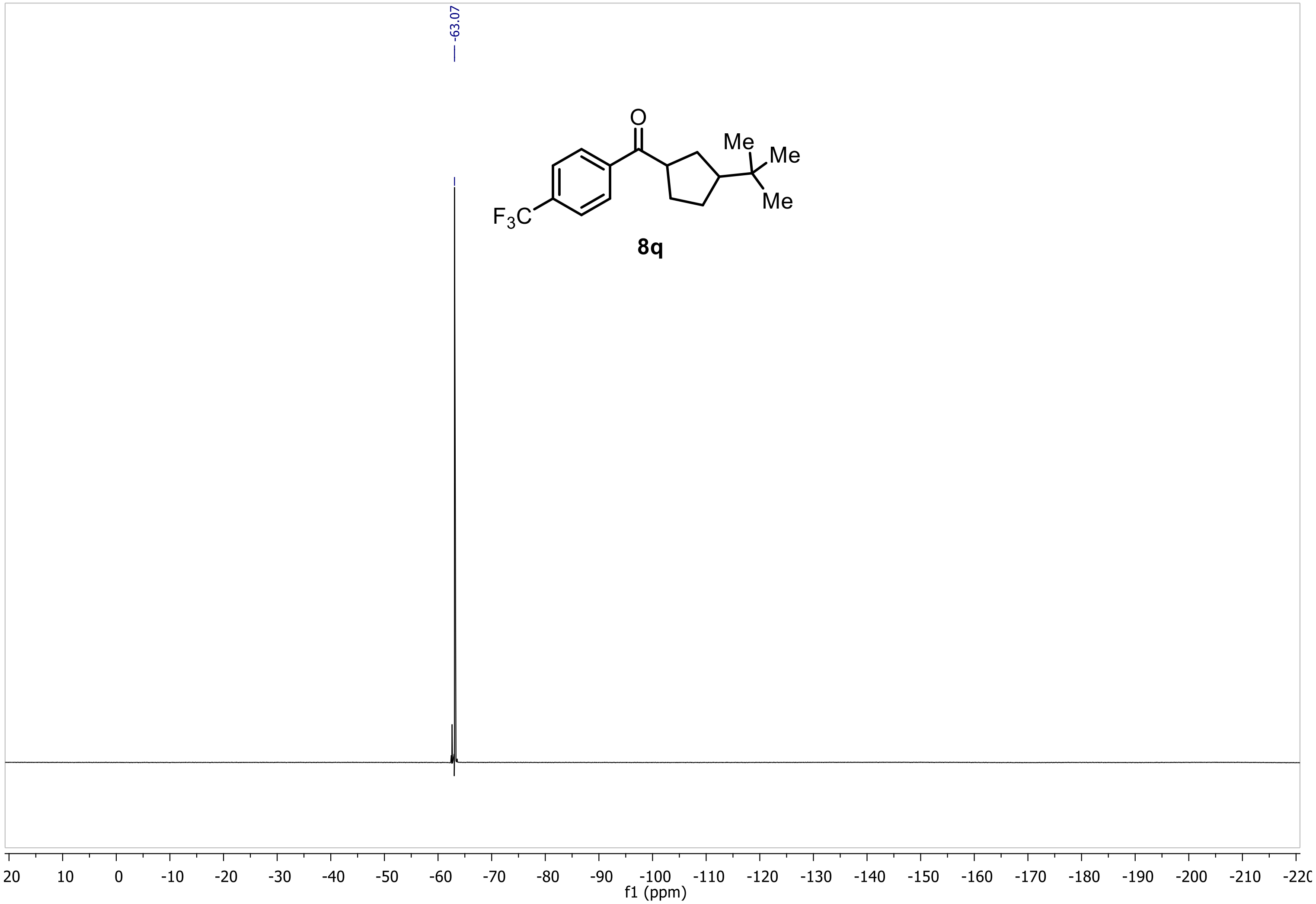

8r – (3-Methylcyclopentyl)[4-(trifluoromethyl)phenyl]methanone

^{1}H NMR (400 MHz, $CDCl_3$)

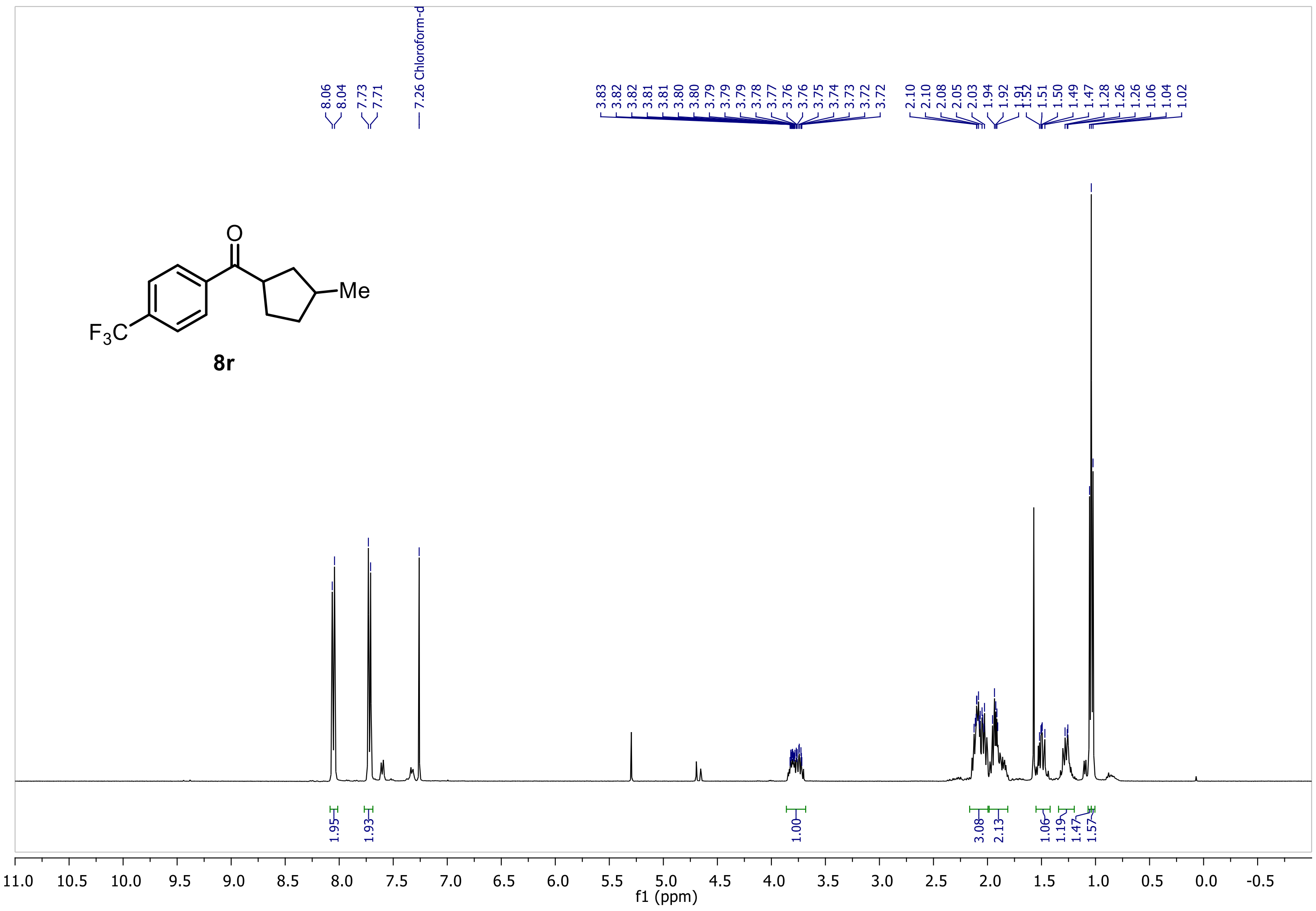

^{19}F NMR (376 MHz, CDCl$_3$)

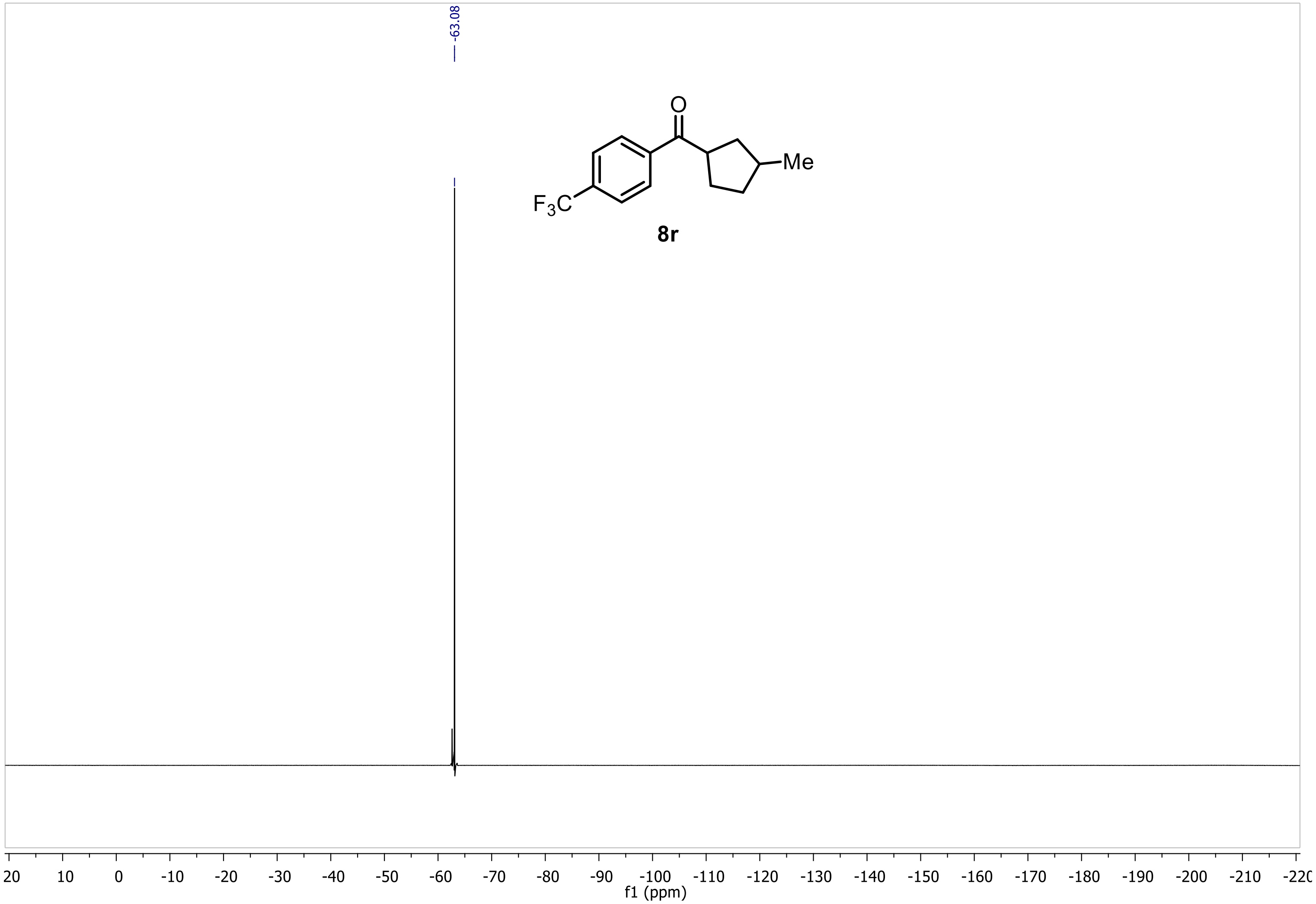

8s – (3-Methylcyclopentyl)(phenyl)methanone

^{1}H NMR (400 MHz, $CDCl_3$)

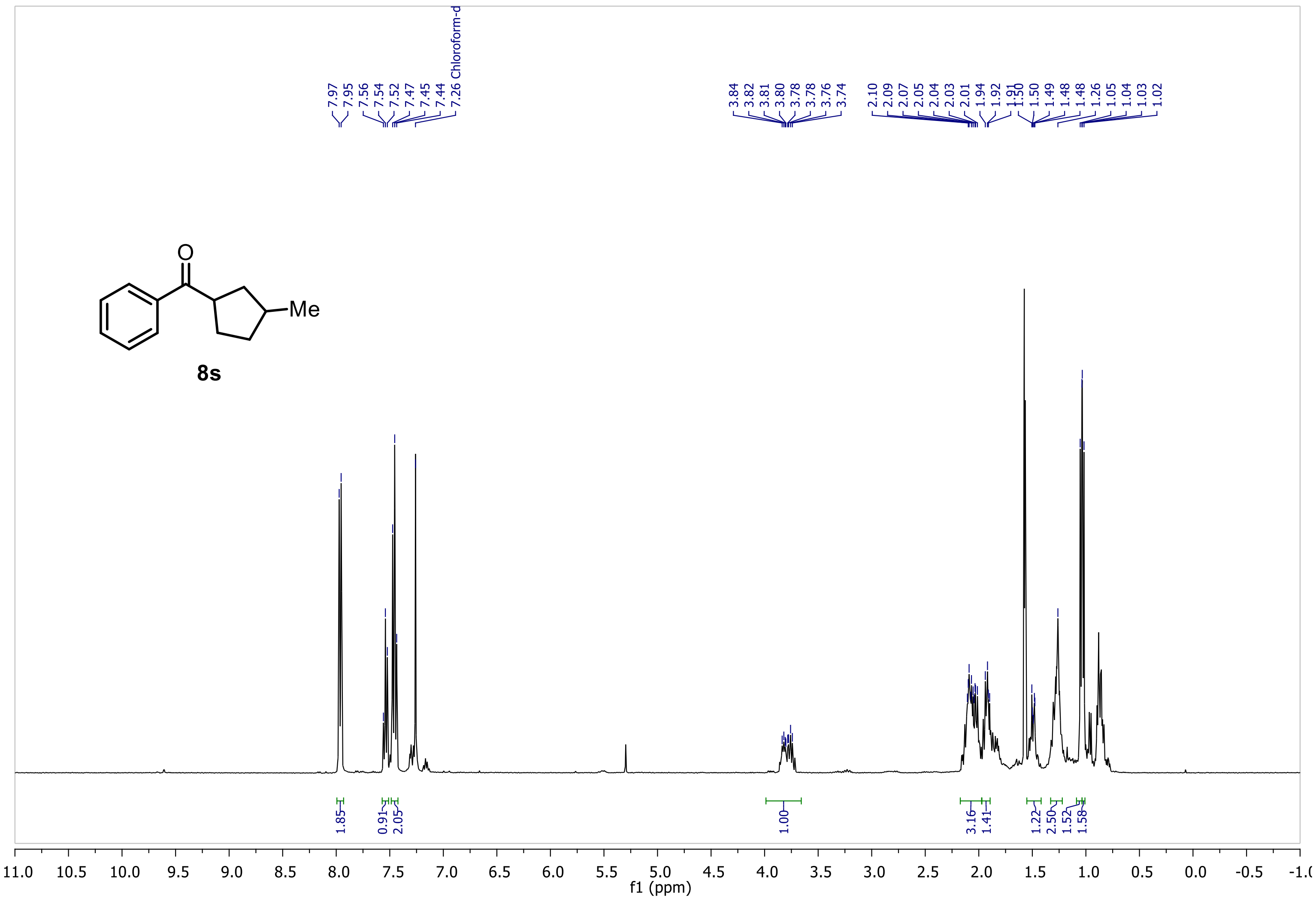

8t – *trans*-(4-Butylcyclohexyl)[4-(trifluoromethyl)phenyl]methanone

^{1}H NMR (400 MHz, $CDCl_3$)

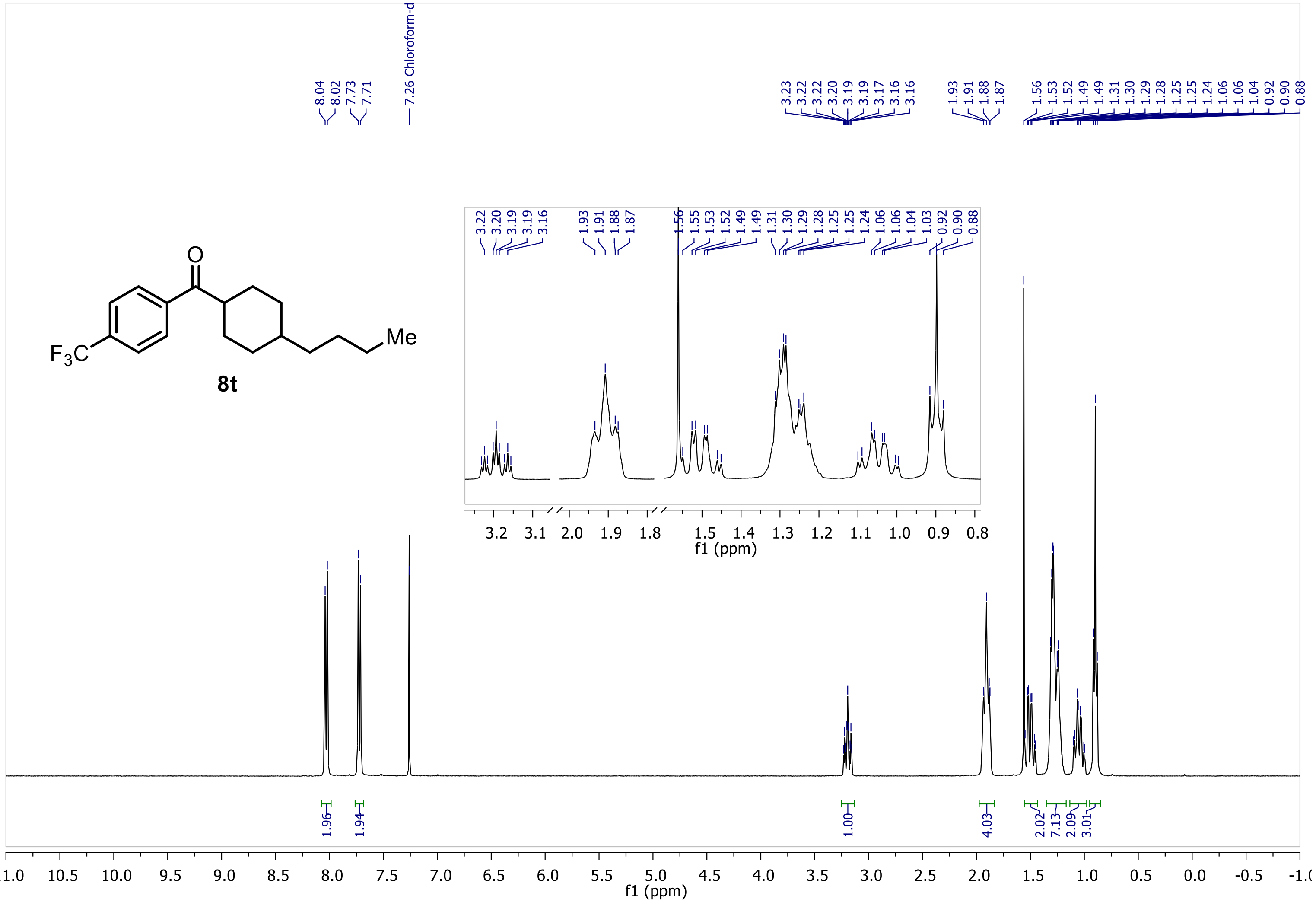

^{19}F NMR (376 MHz, $CDCl_3$)

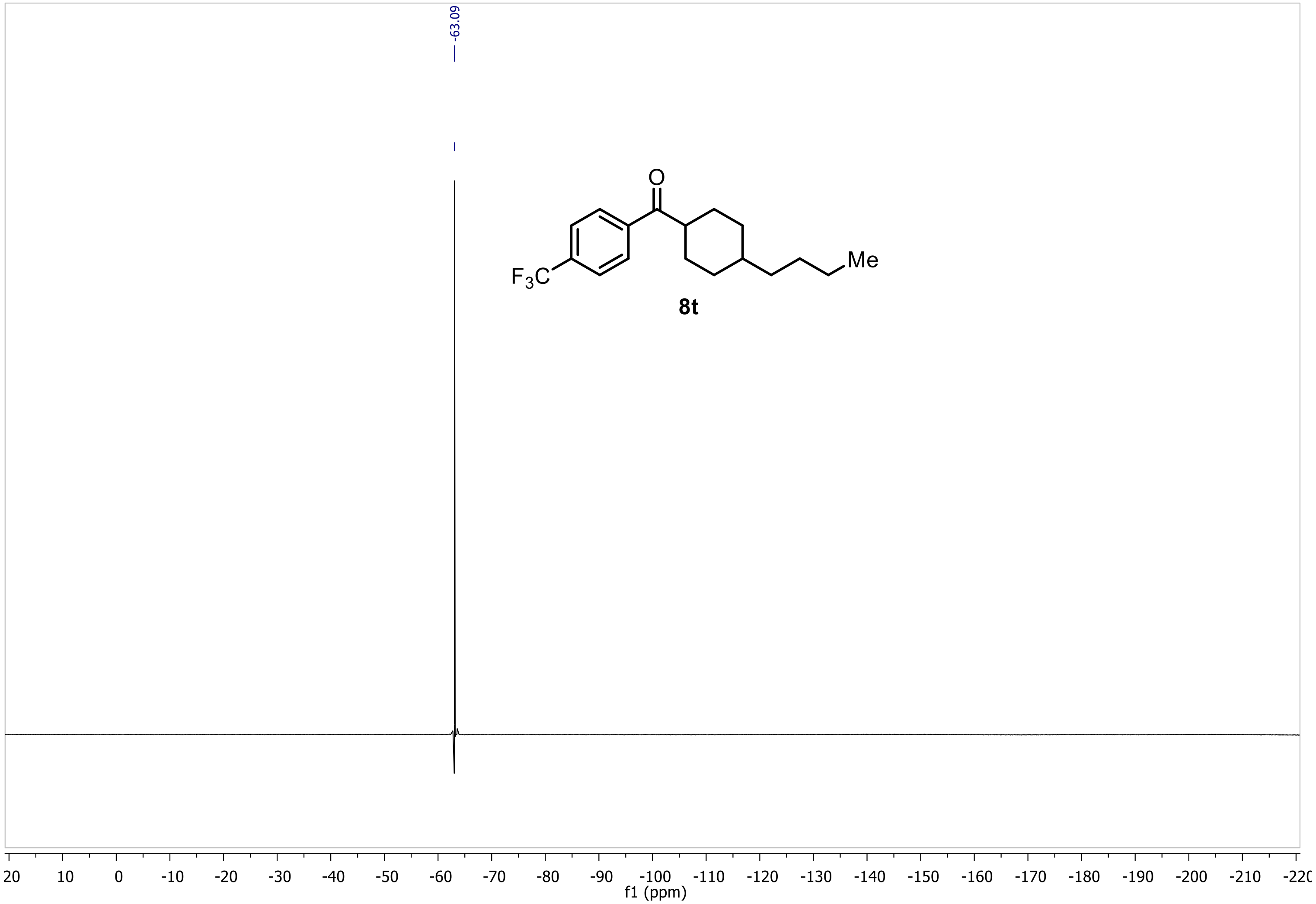

8u – [4-(*tert*-Butyl)cyclohexyl][4-(trifluoromethyl)phenyl]methanone

^{1}H NMR (400 MHz, $CDCl_3$)

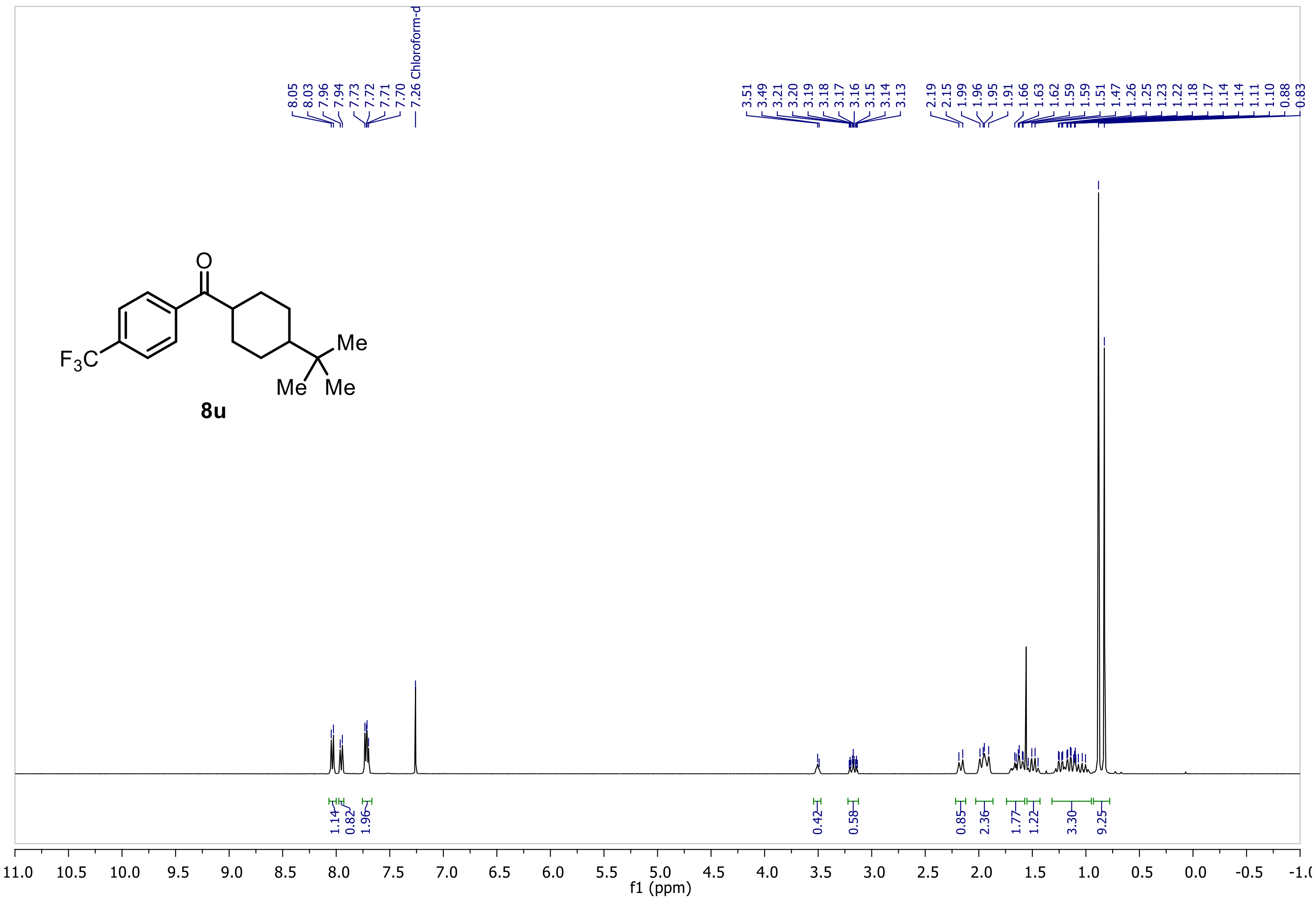

^{19}F NMR (376 MHz, CDCl$_3$)

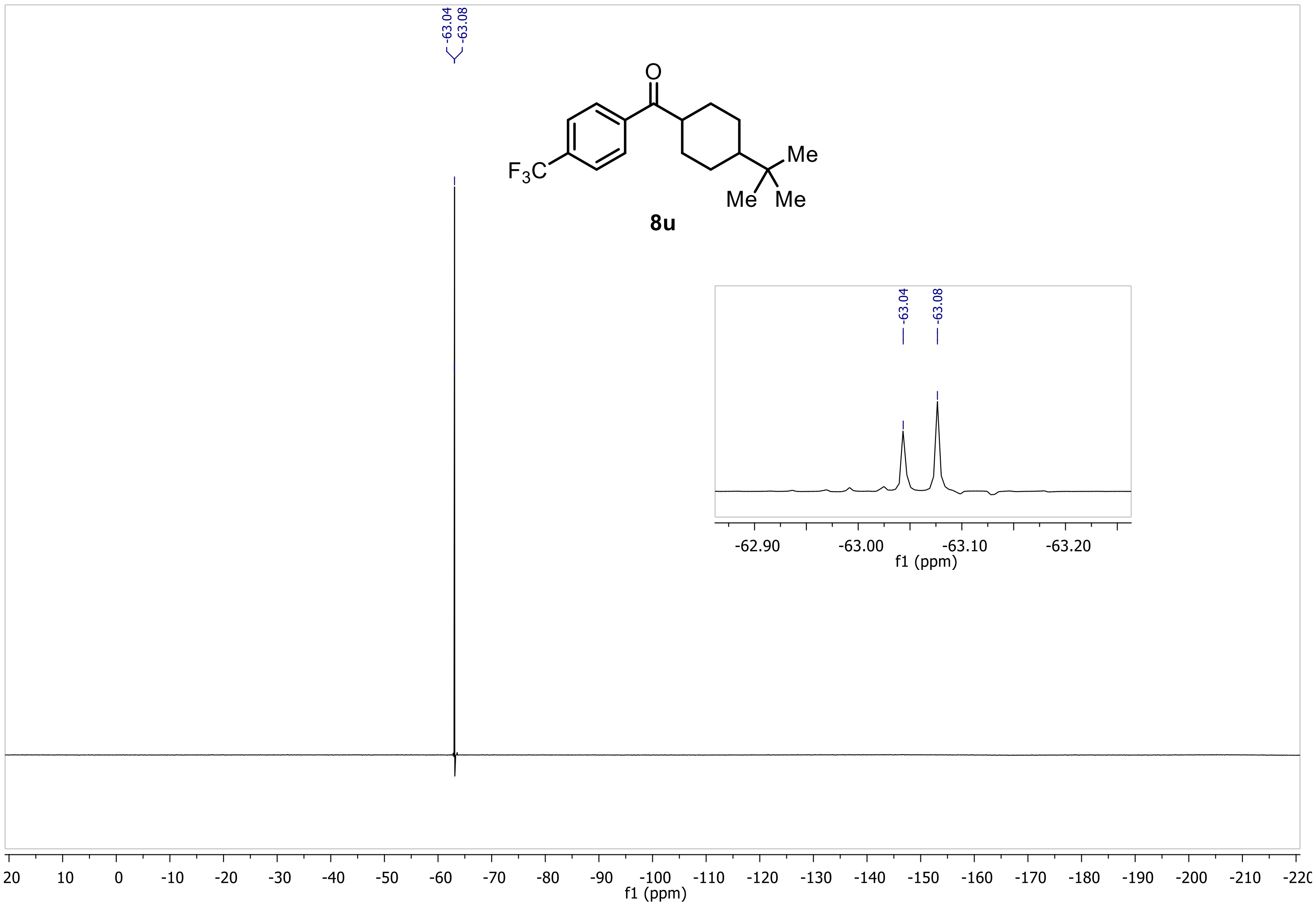

8v – [4-(*tert*-Butyl)cyclohexyl][3-(trifluoromethyl)phenyl]methanone

^{1}H NMR (700 MHz, $CDCl_3$)

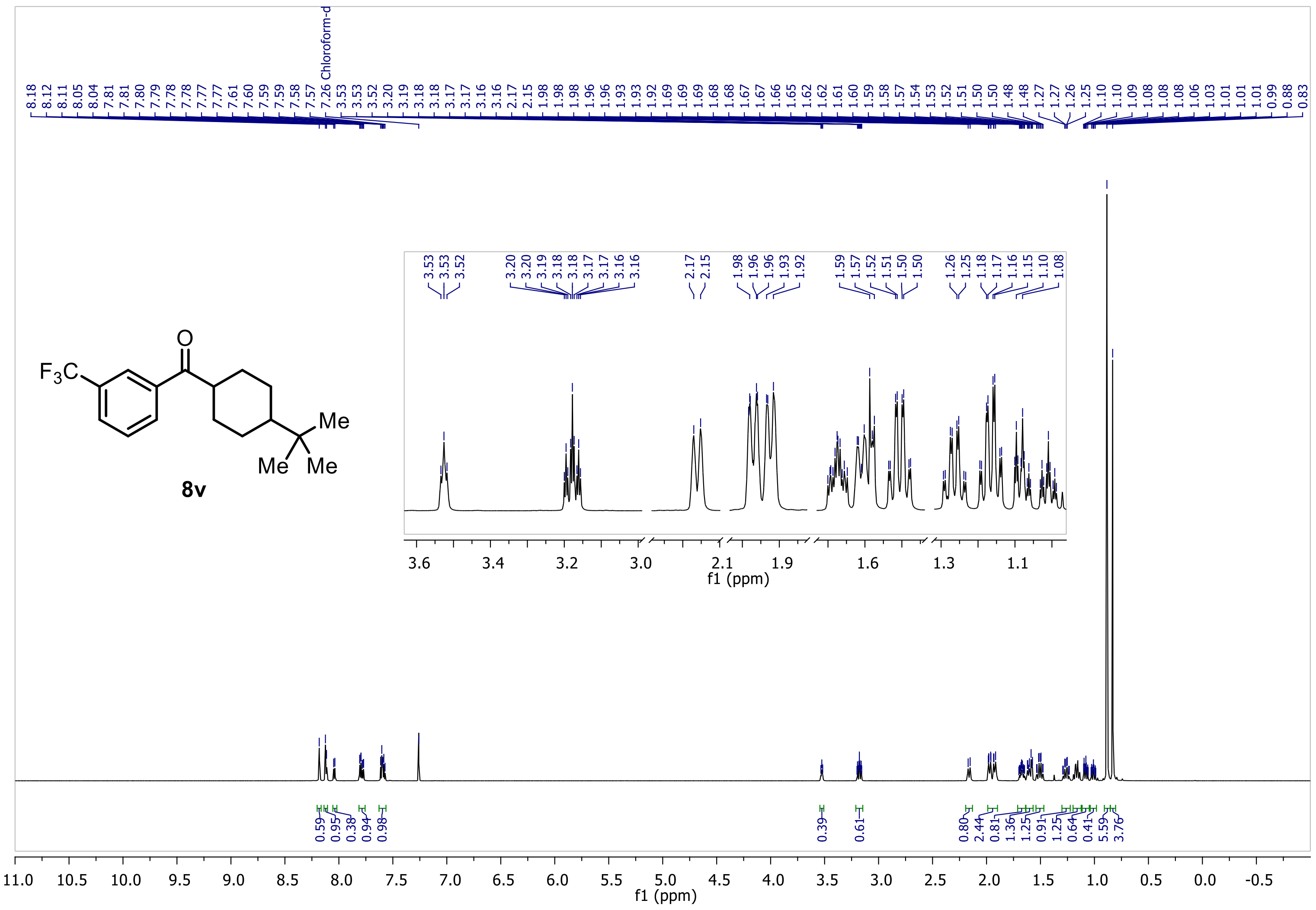

^{19}F NMR (659 MHz, $CDCl_3$)

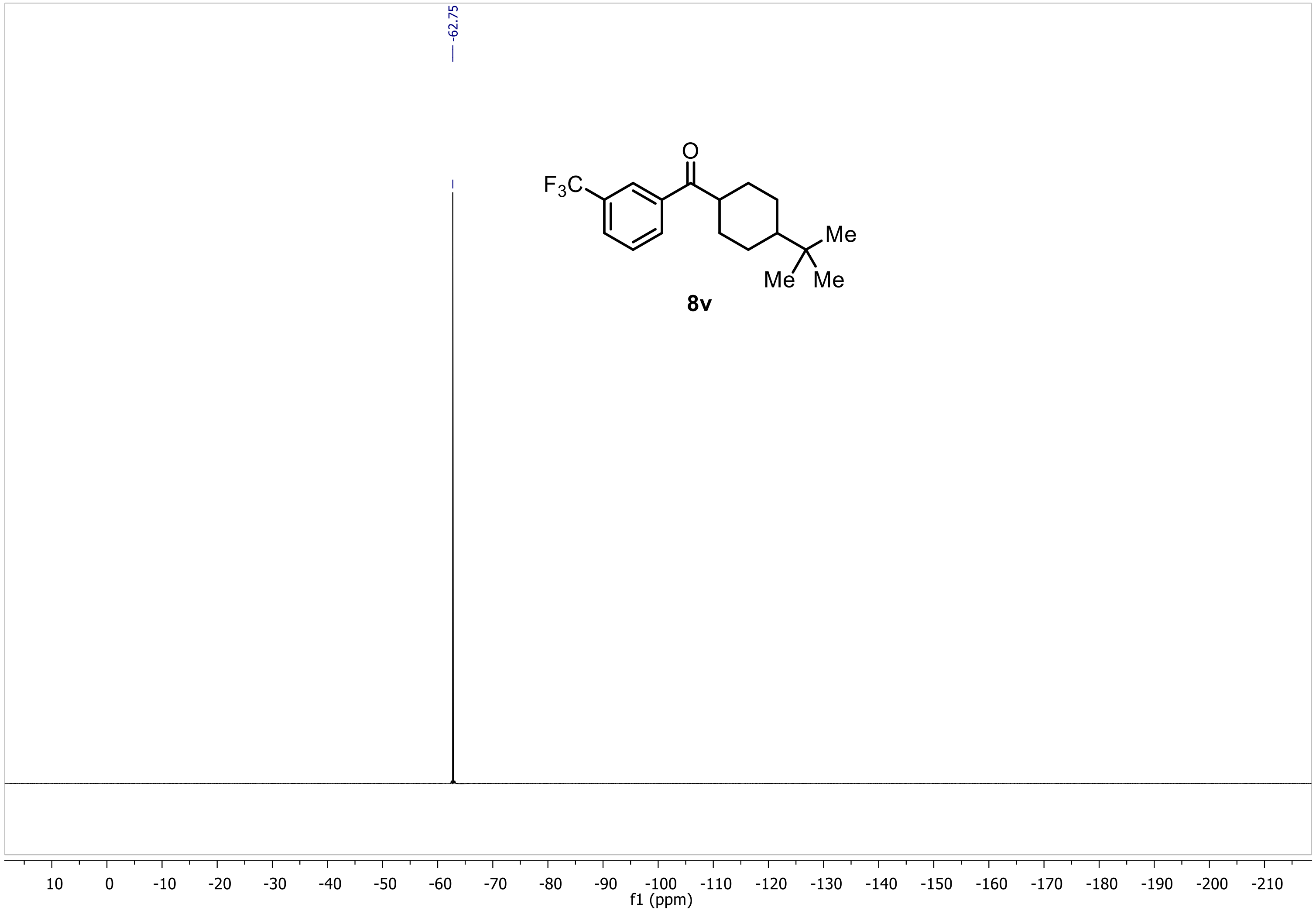

8w – (4-Methylcyclohexyl)[4-(trifluoromethyl)phenyl]methanone

^{1}H NMR (400 MHz, CDCl$_3$)

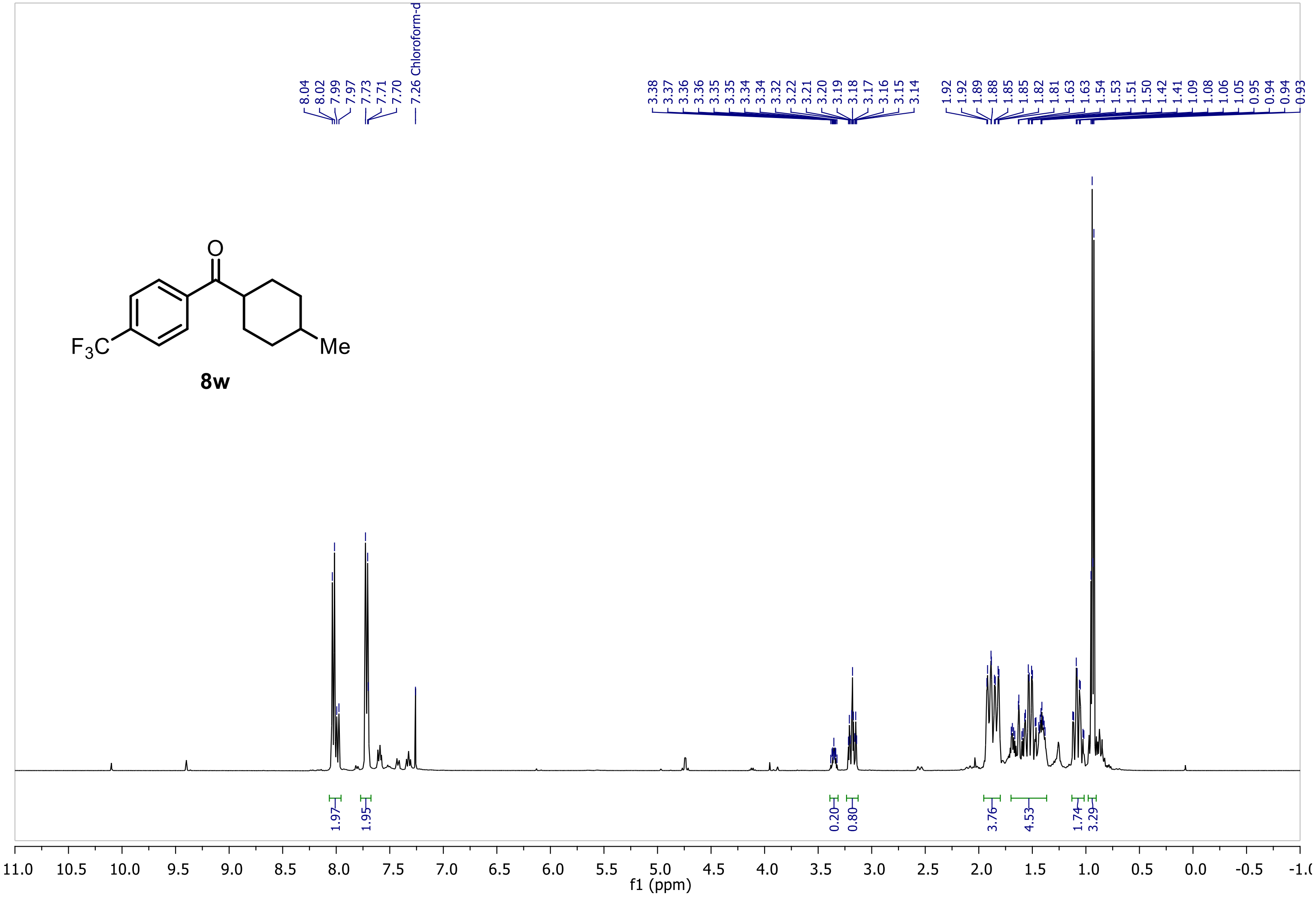

^{19}F NMR (376 MHz, $CDCl_3$)

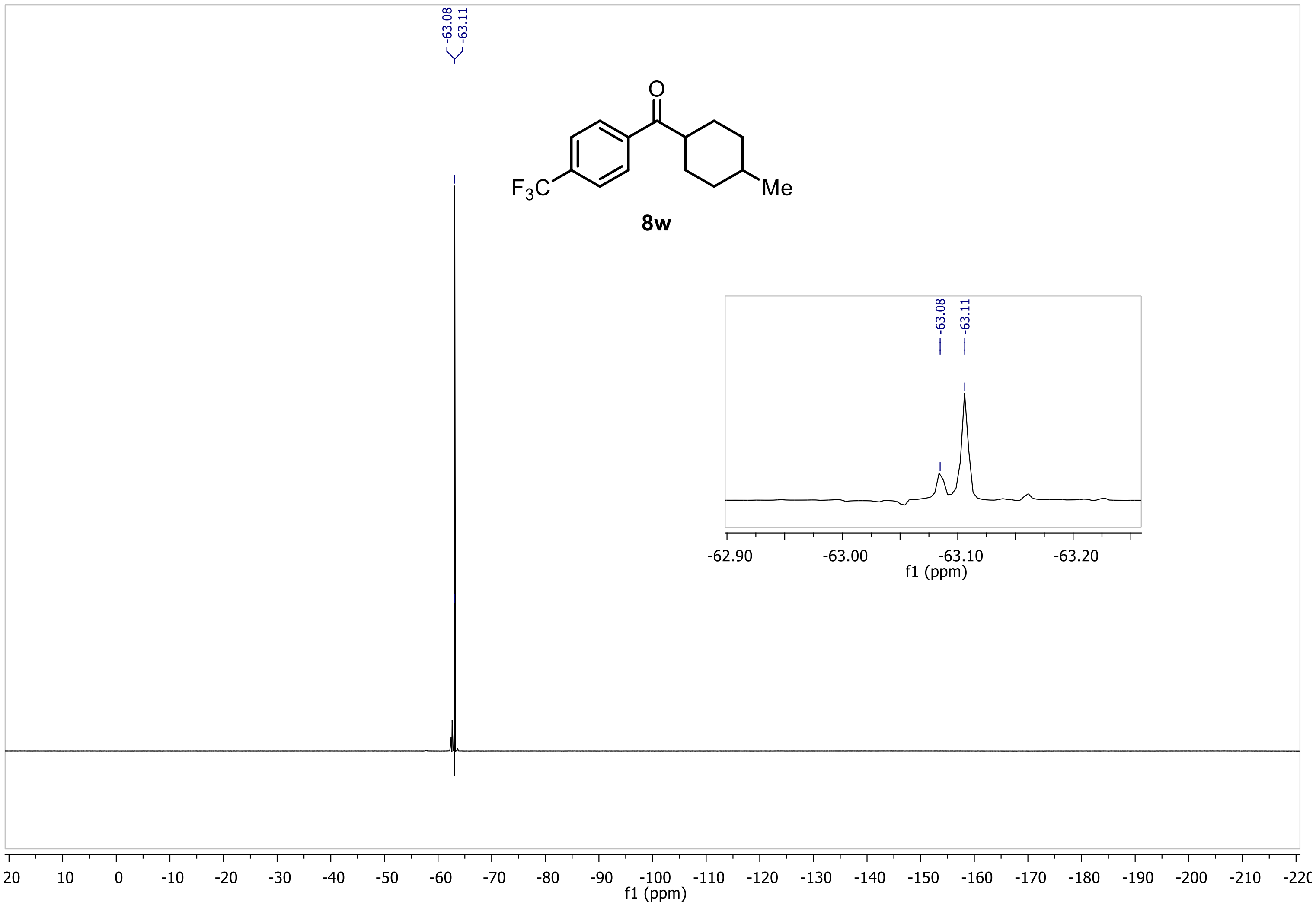

1a – Trimethyl{(3-methylcyclohexylidene)[4-(trifluoromethyl)phenyl]methoxy}silane

^{1}H NMR (400 MHz, $CDCl_3$)

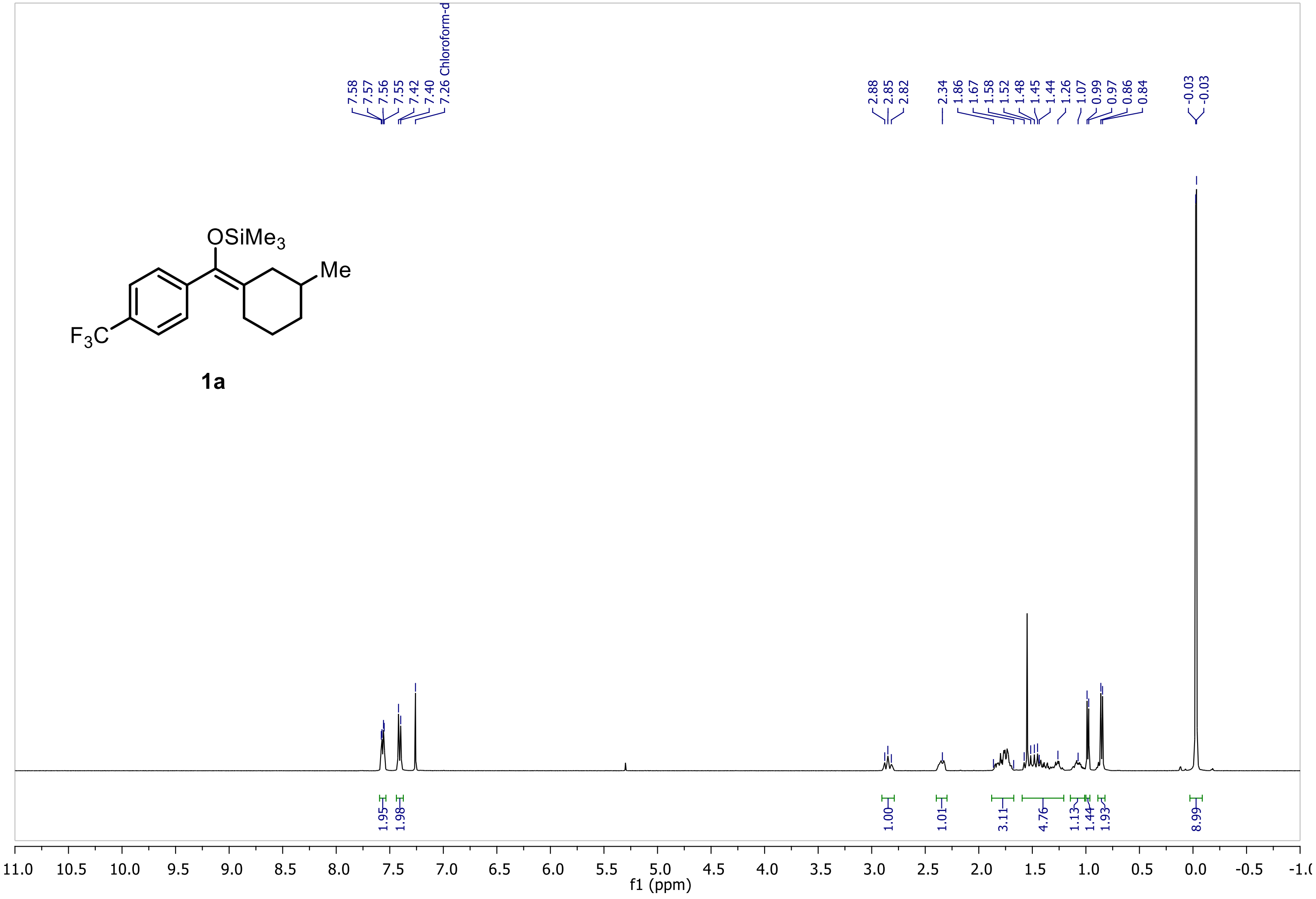

$^{13}C\{^1H\}$ NMR (151 MHz, $CDCl_3$)

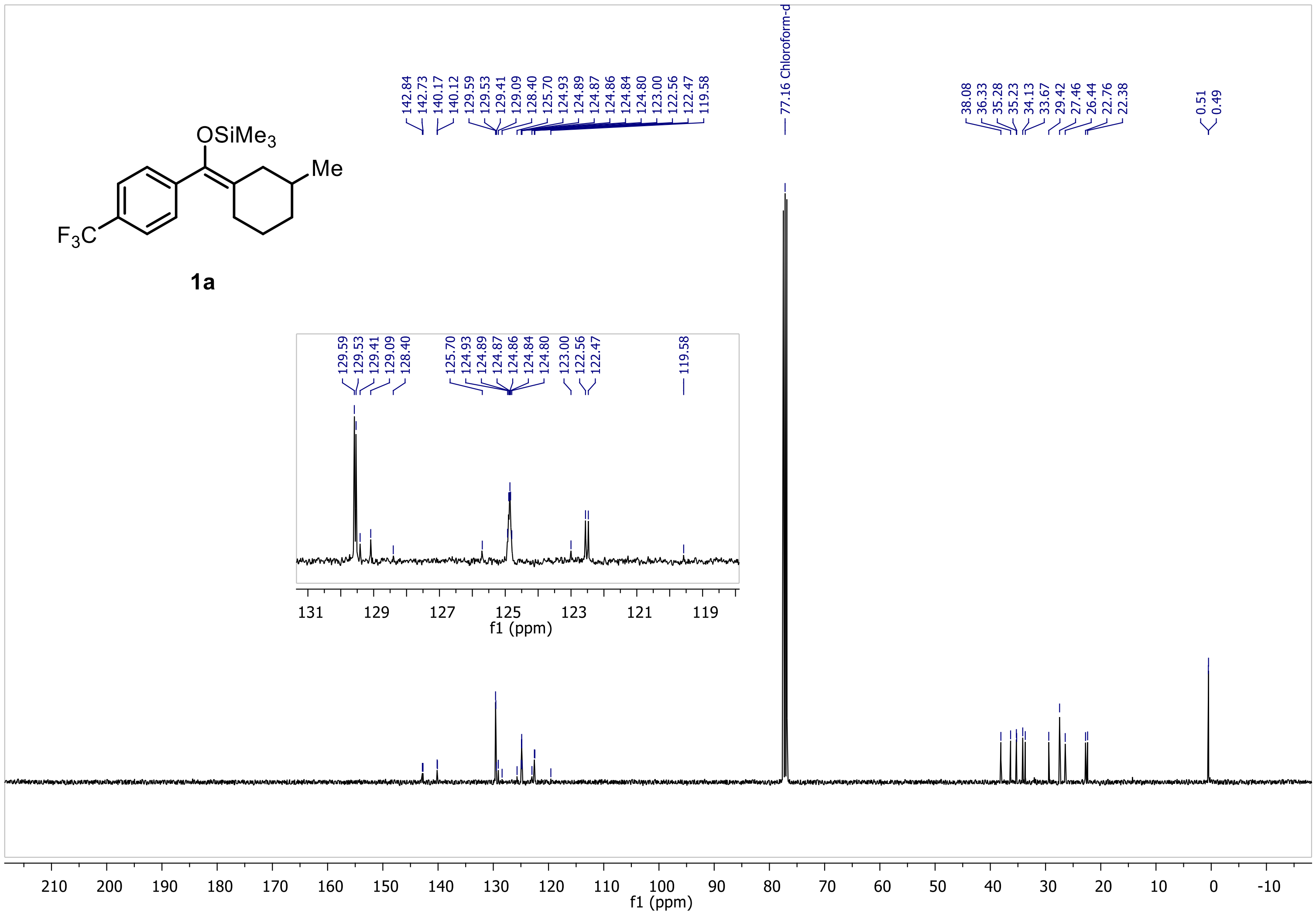

^{19}F NMR (376 MHz, $CDCl_3$)

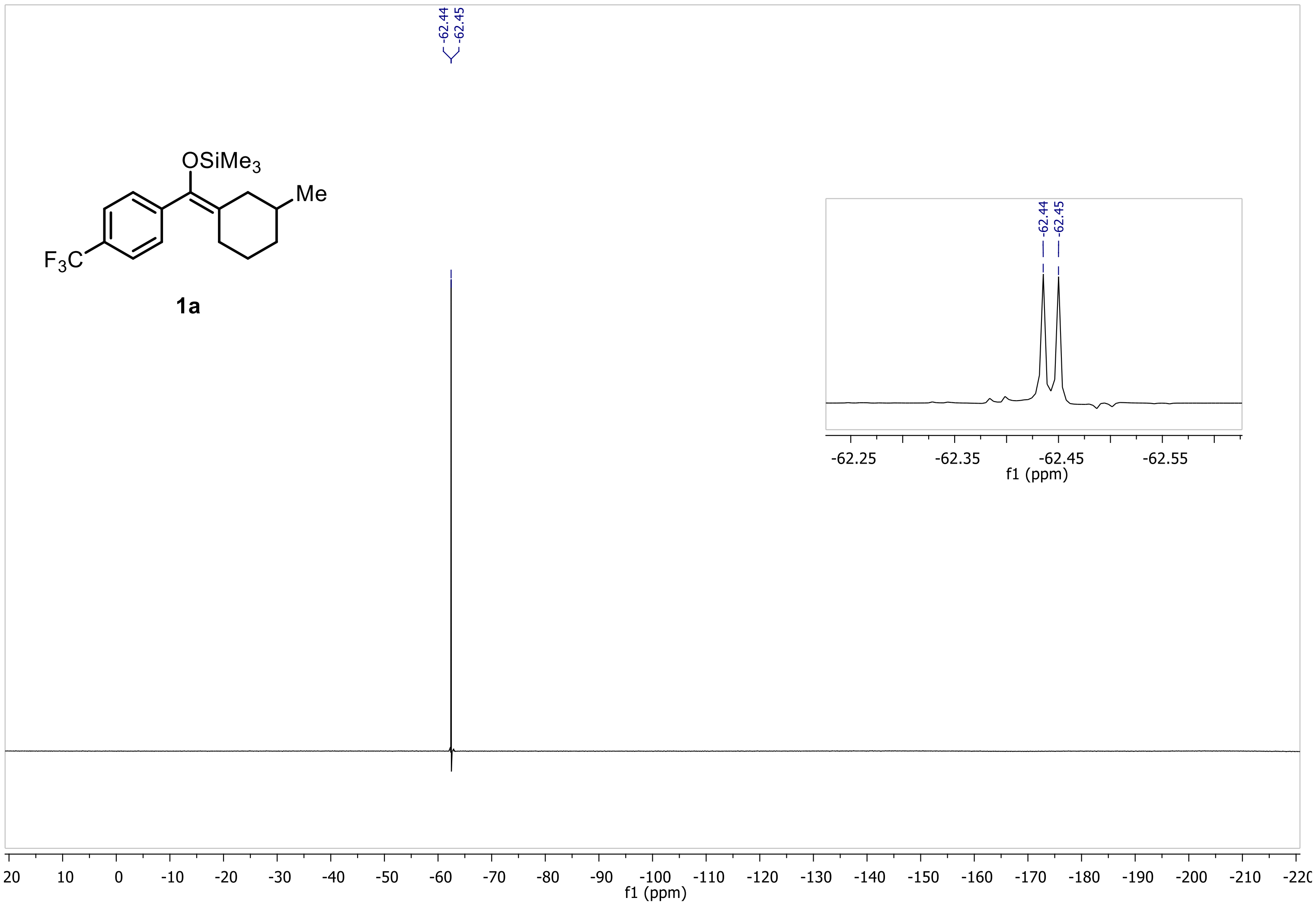

1b – (((5*S*,8*R*,9*S*,10*S*,13*R*,14*S*,17*R*)-10,13-dimethyl-17-((*R*)-6-methylheptan-2-yl)hexa decahydro-3*H*-cyclopenta[a]phenanthren-3-ylidene)(4-trifluoromethyl)phenyl)methoxy) trimethylsilane

^{1}H NMR (400 MHz, $CDCl_3$)

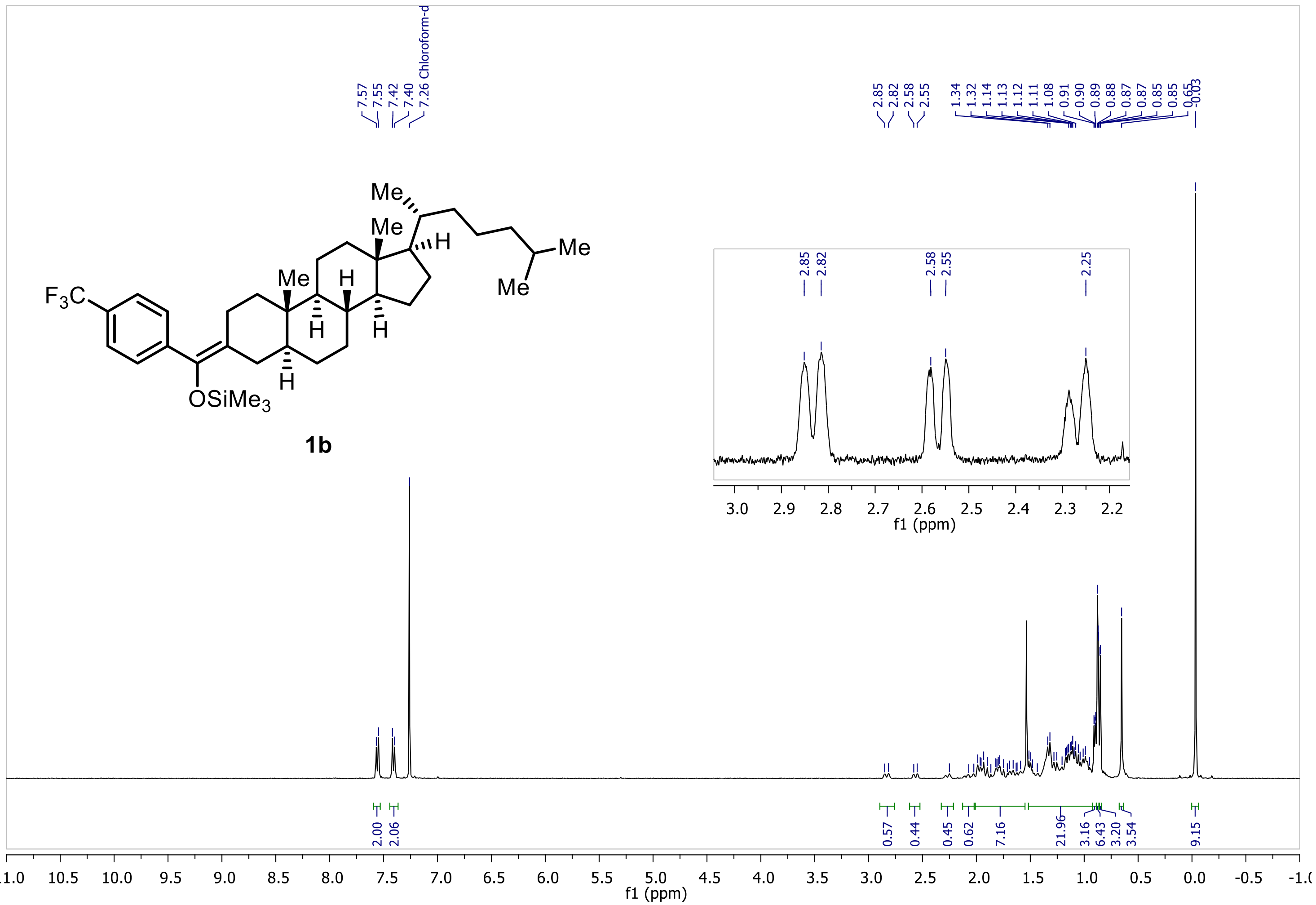

^{13}C (DEPT 135) NMR (101 MHz, $CDCl_3$)

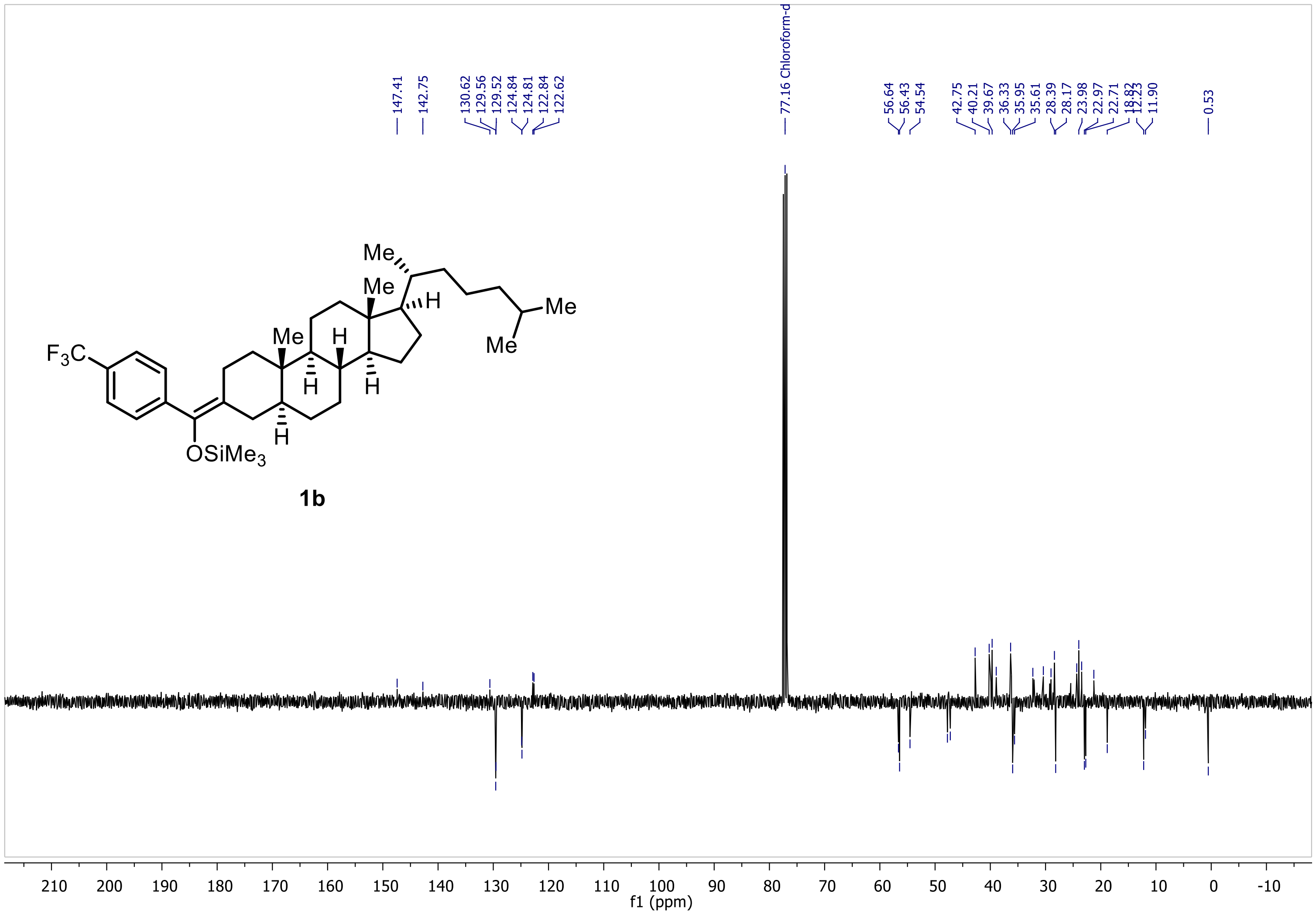

^{19}F NMR (376 MHz, $CDCl_3$)

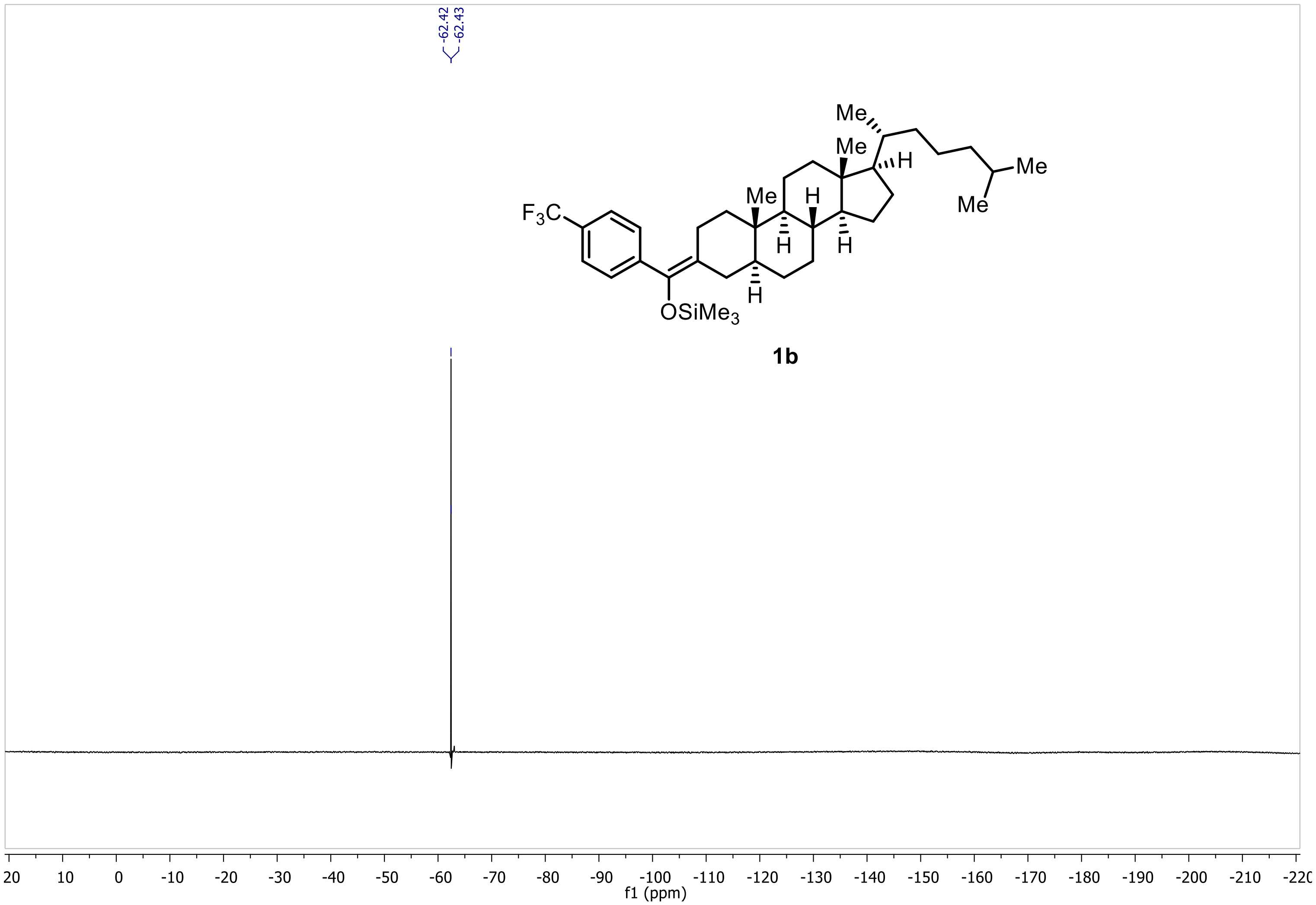

1b

1c – Trimethyl{[4-(trifluoromethyl)phenyl](3,3,5-trimethylcyclohexylidene)methoxy}silane

^{1}H NMR (600 MHz, $CDCl_3$)

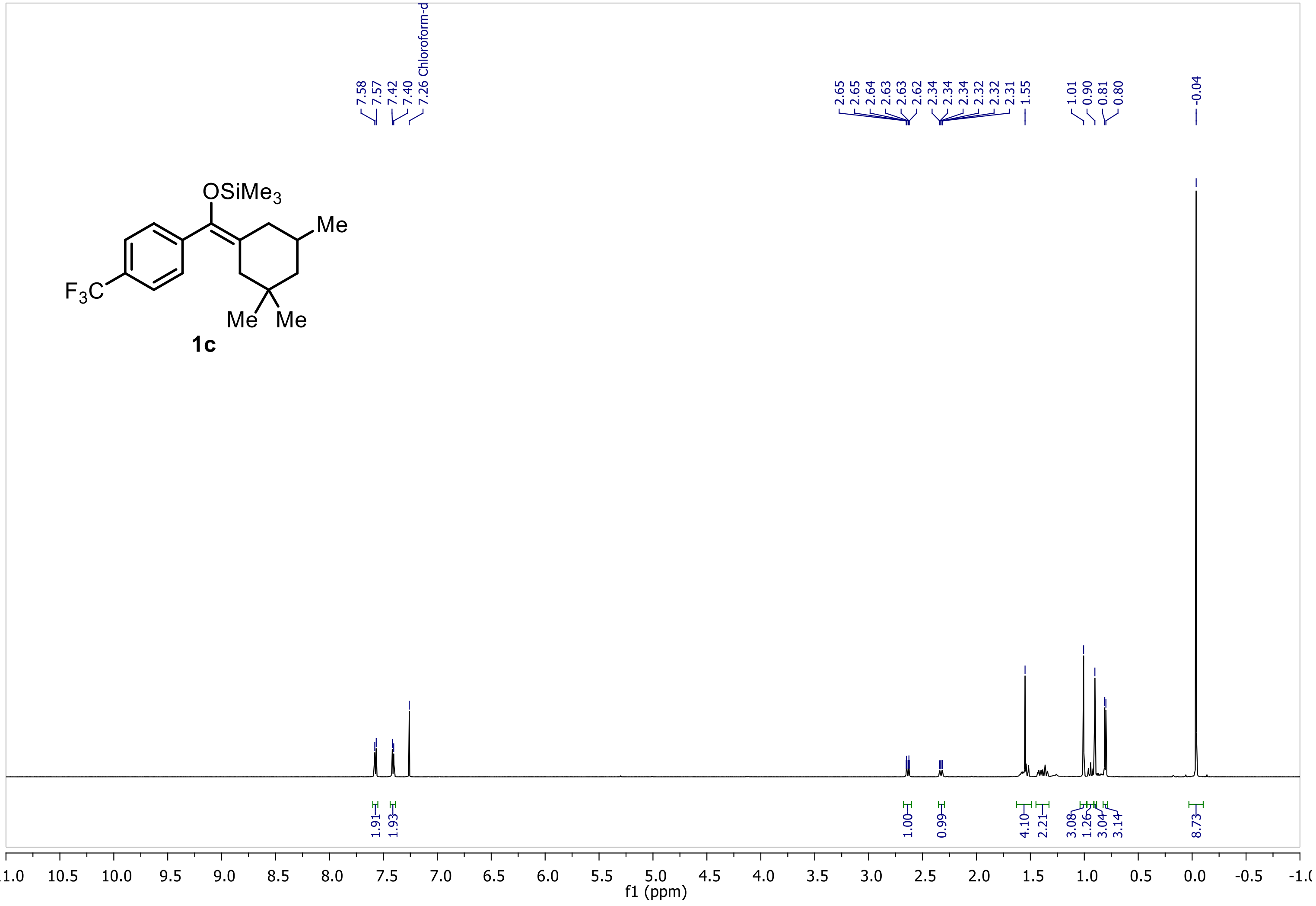

$^{13}C\{^1H\}$ NMR (151 MHz, $CDCl_3$)

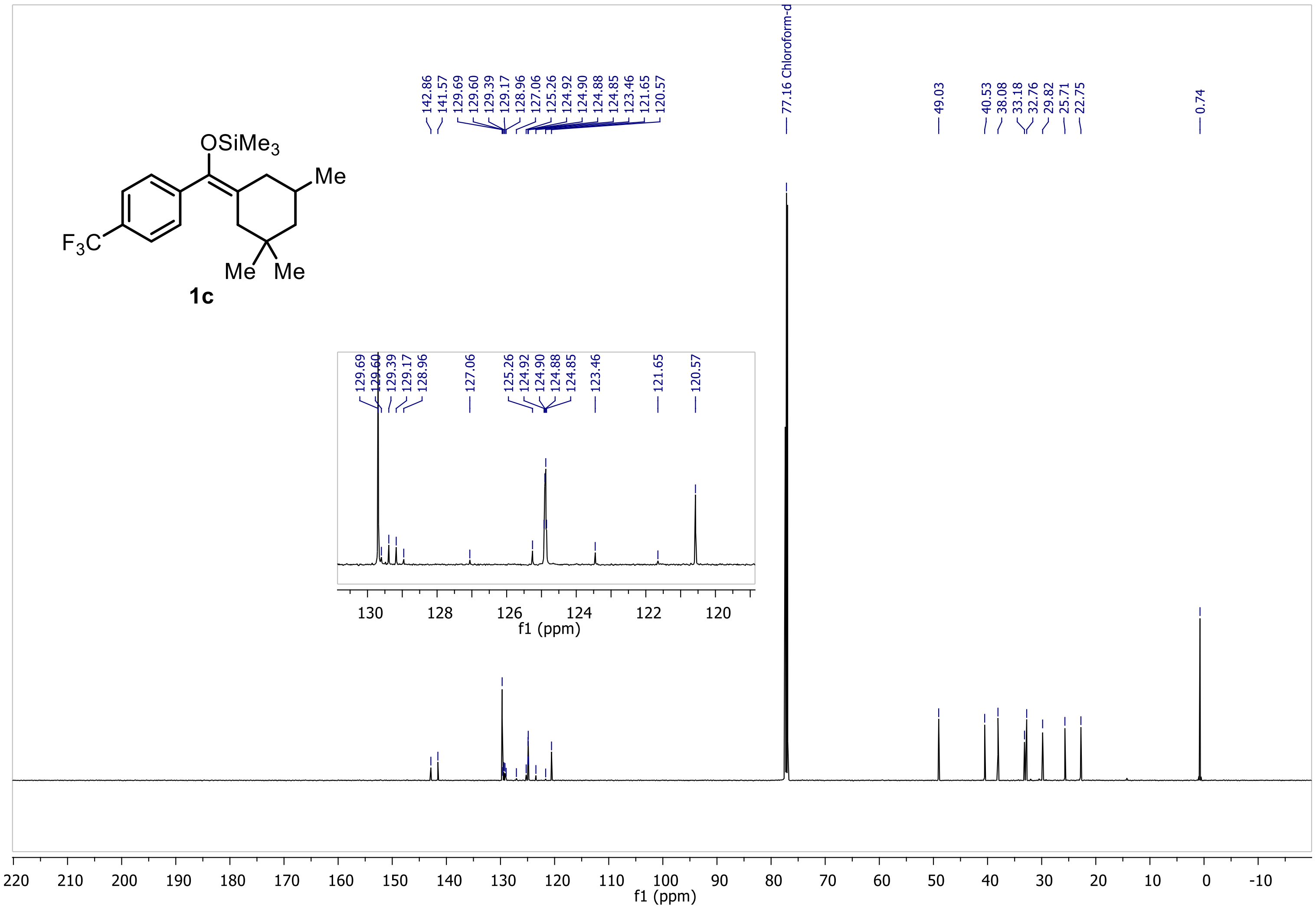

^{19}F NMR (565 MHz, CDCl$_3$)

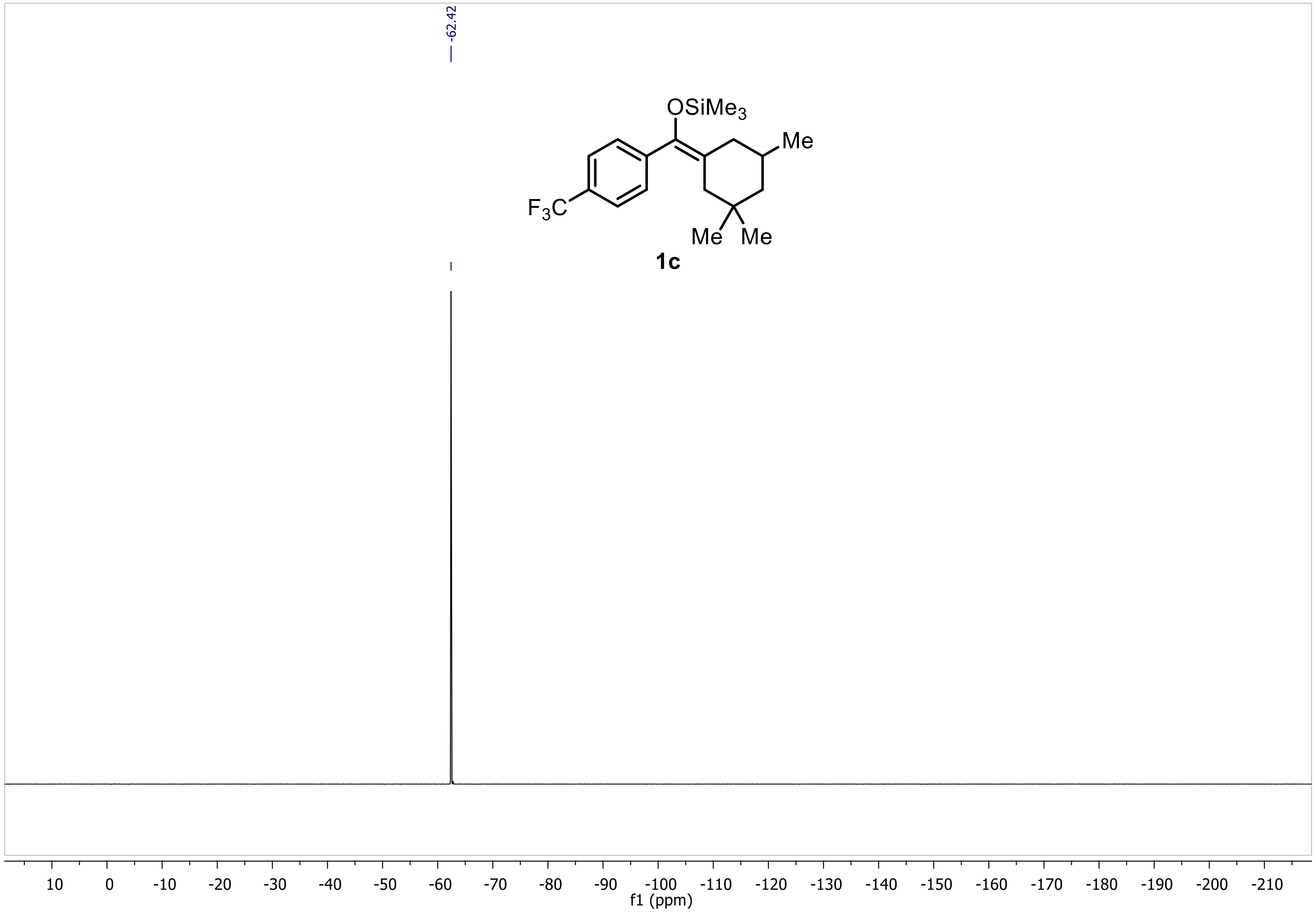

1d – {(Cyclopentylidene-2,2,5,5-d_4)[4-(trifluoromethyl)phenyl]methoxy}trimethylsilane

^{1}H NMR (700 MHz, $CDCl_3$):

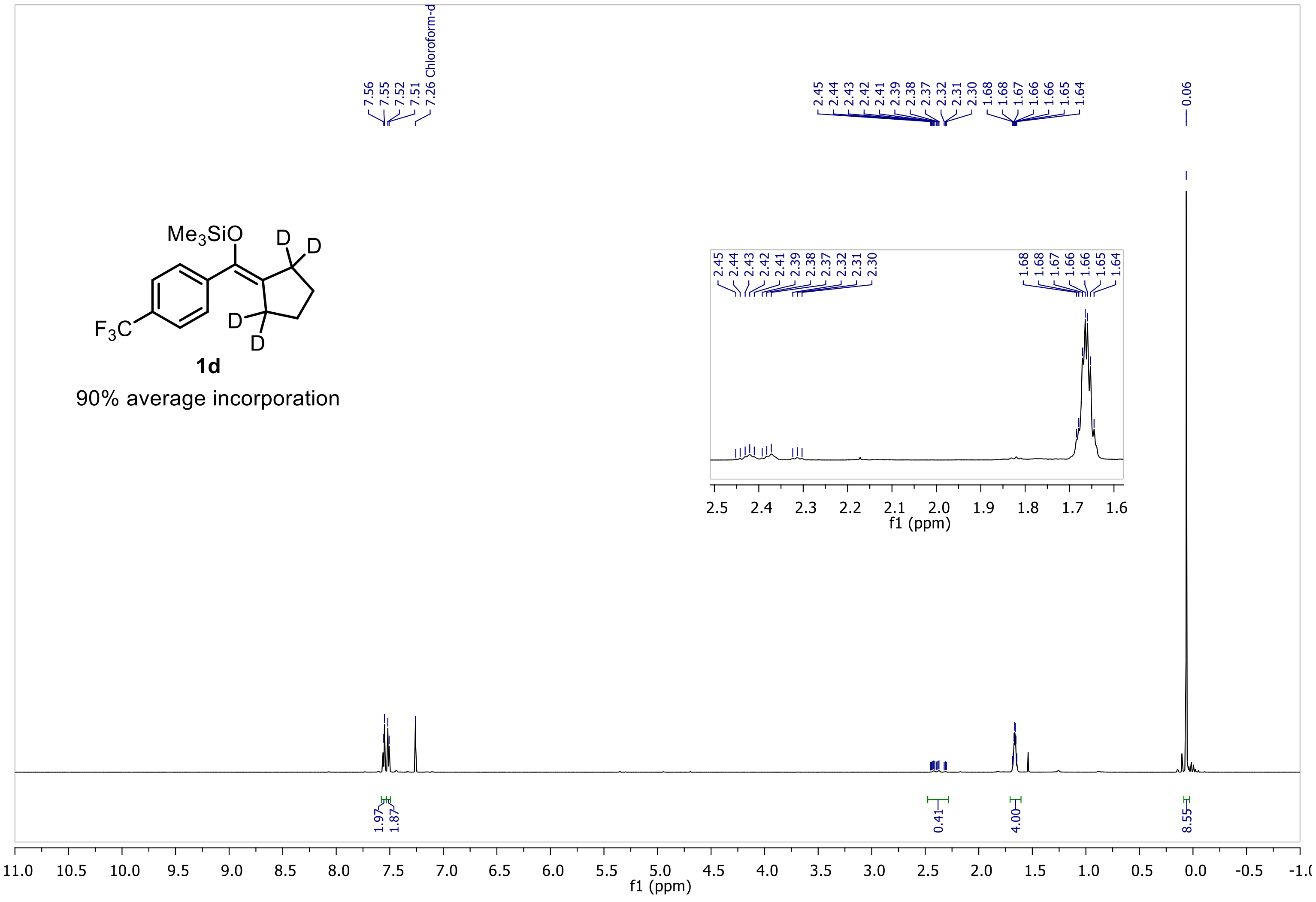

$^{13}C\{^1H\}$ NMR (176 MHz, $CDCl_3$):

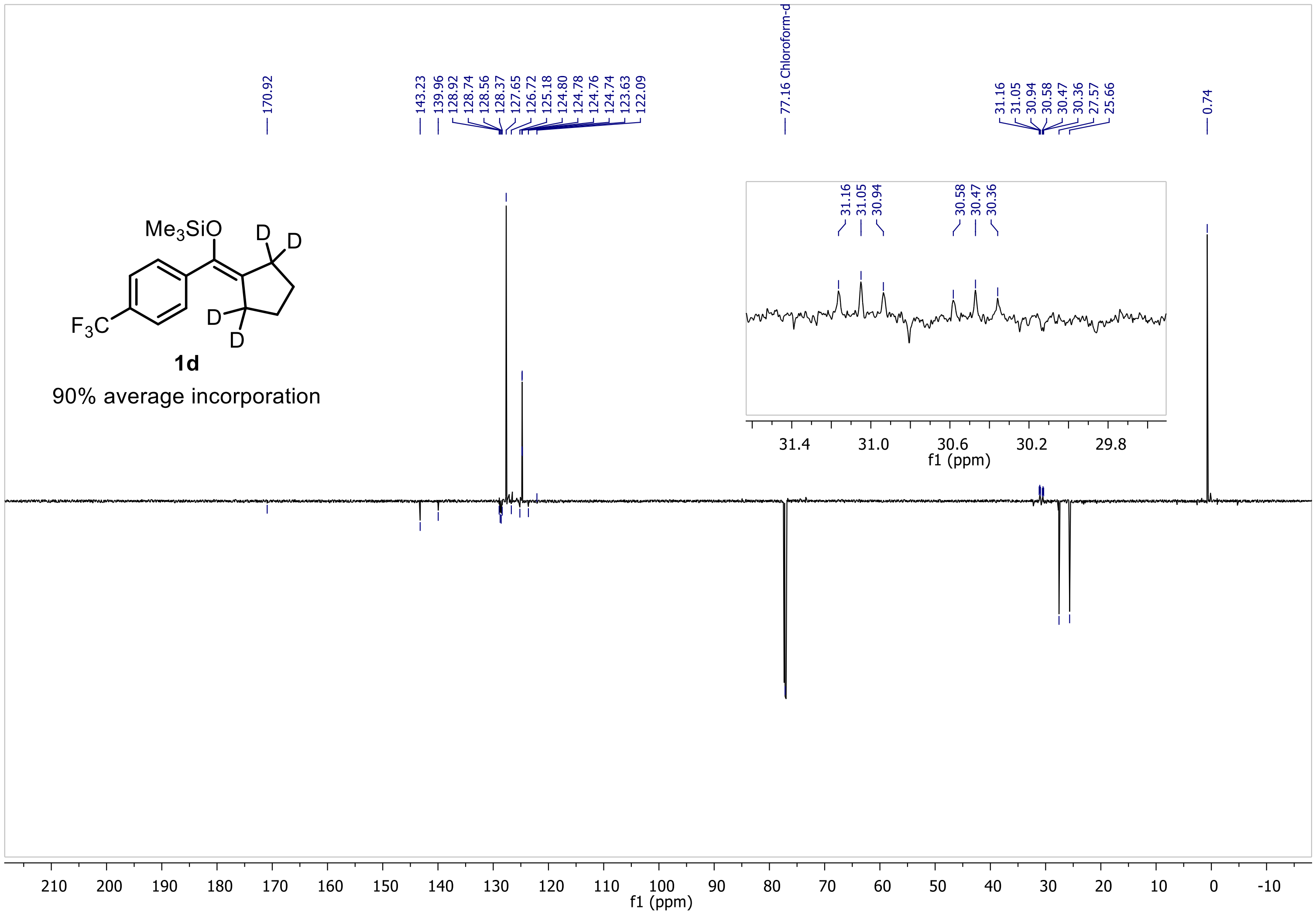

^{19}F NMR (377 MHz, CDCl$_3$):

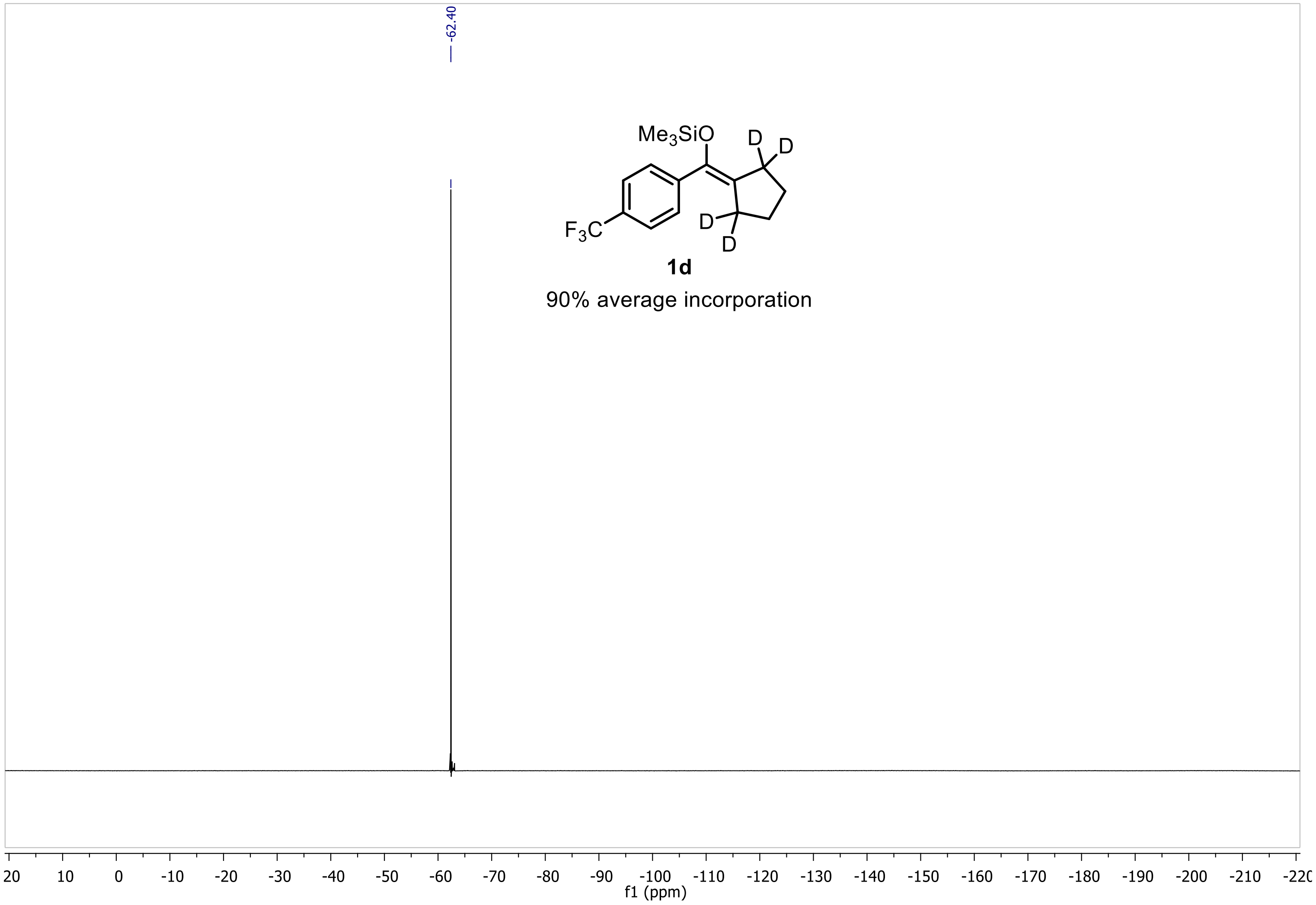

1e – Trimethyl{(3-methylcyclopentylidene-2,2,5,5-d_4)[4-(trifluoromethyl)phenyl]-methoxy}silane

^{1}H NMR (400 MHz, $CDCl_3$):

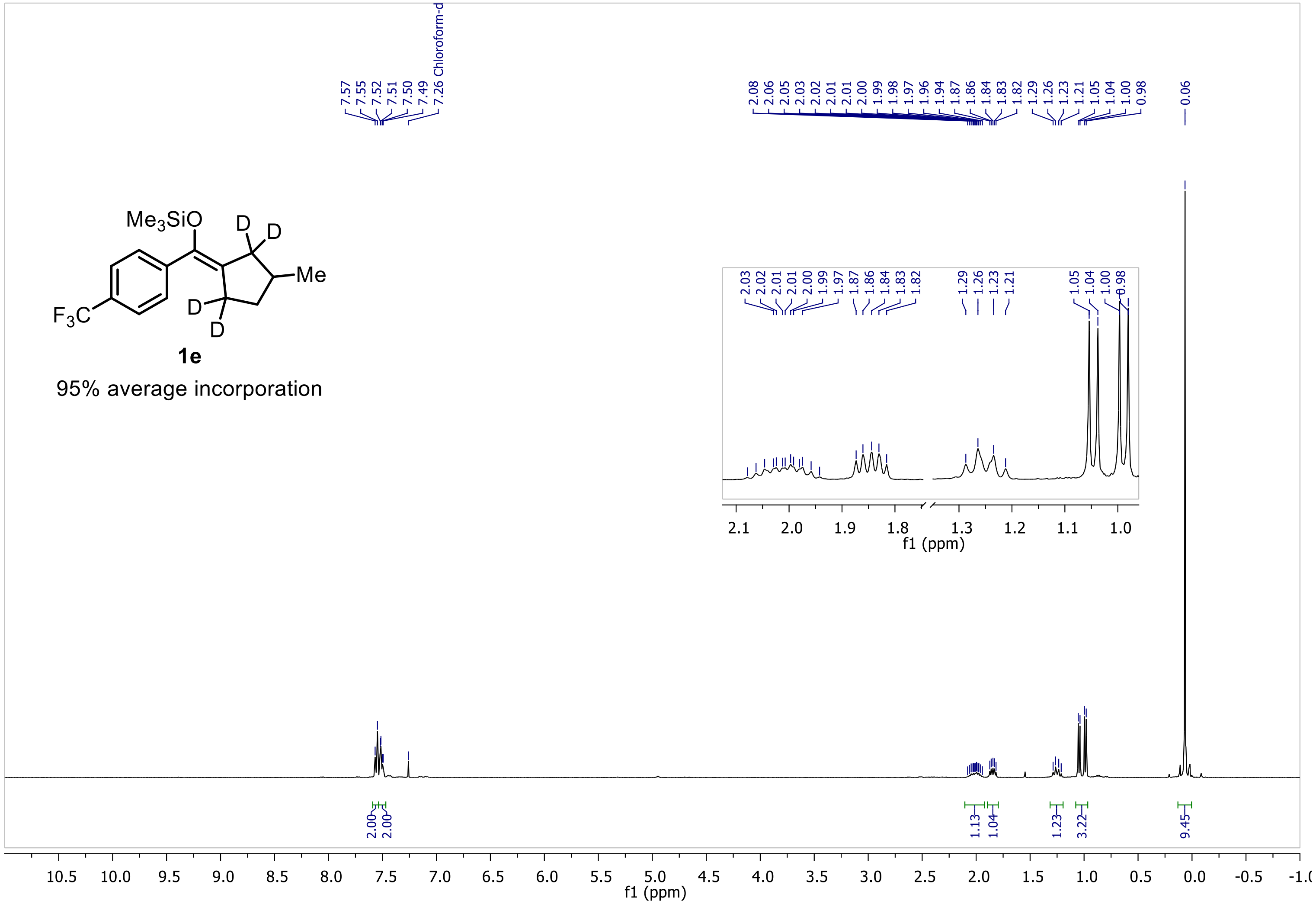

$^{13}C\{^1H\}$ NMR (101 MHz, $CDCl_3$):

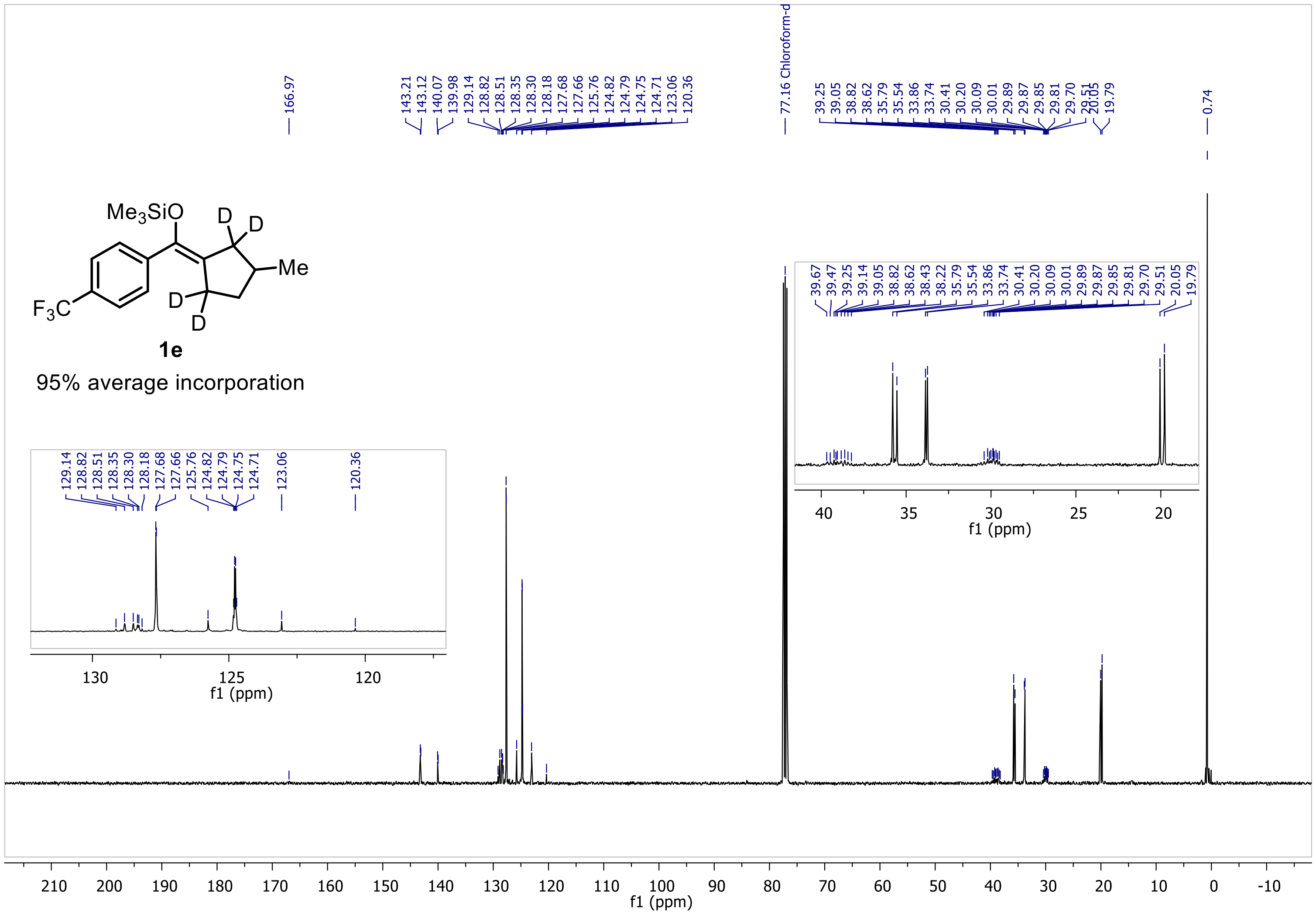

^{19}F NMR (377 MHz, $CDCl_3$):

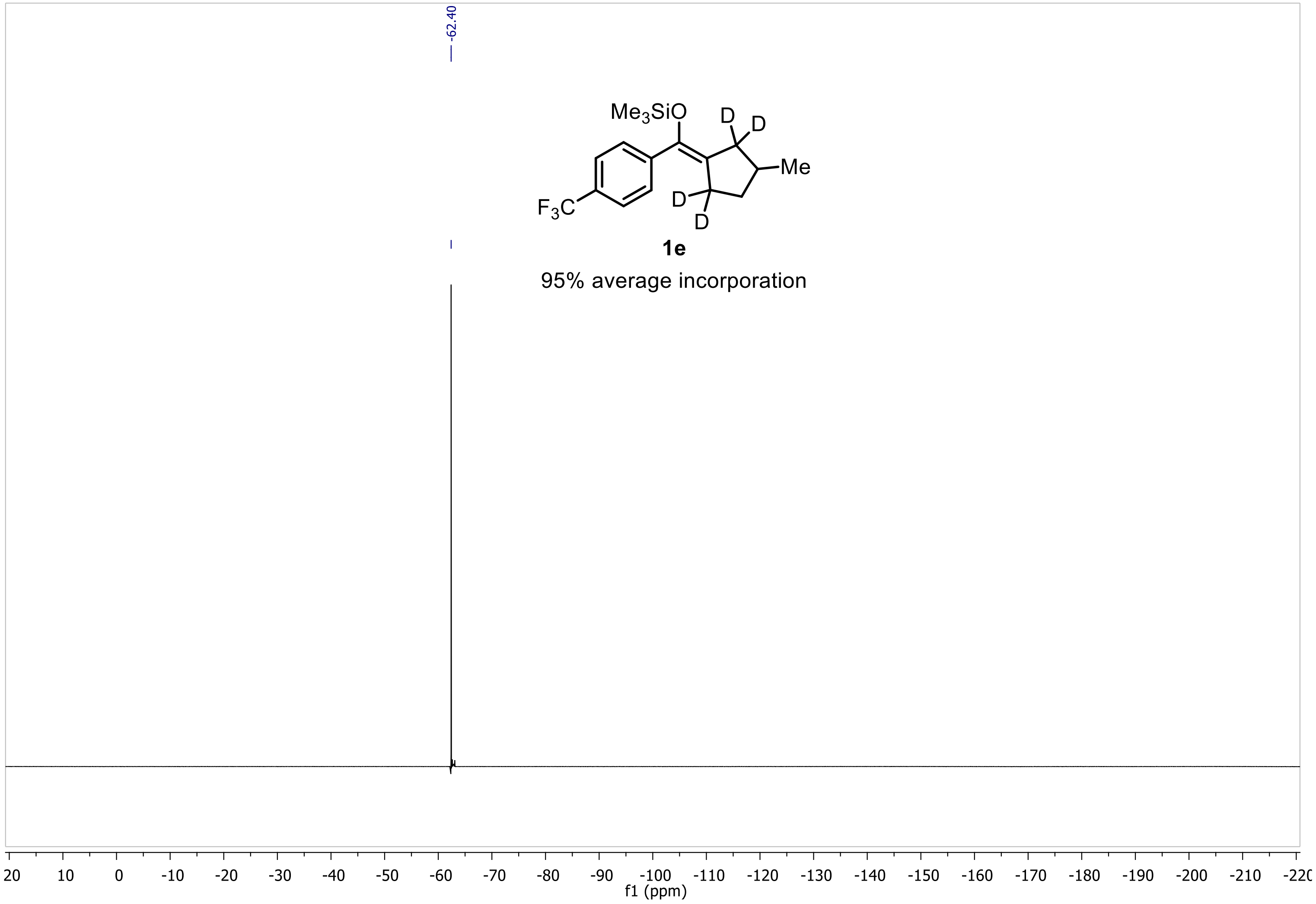

1f – {Cyclopentylidene[4-(trifluoromethyl)phenyl]methoxy}trimethylsilane

^{1}H NMR (400 MHz, $CDCl_3$)

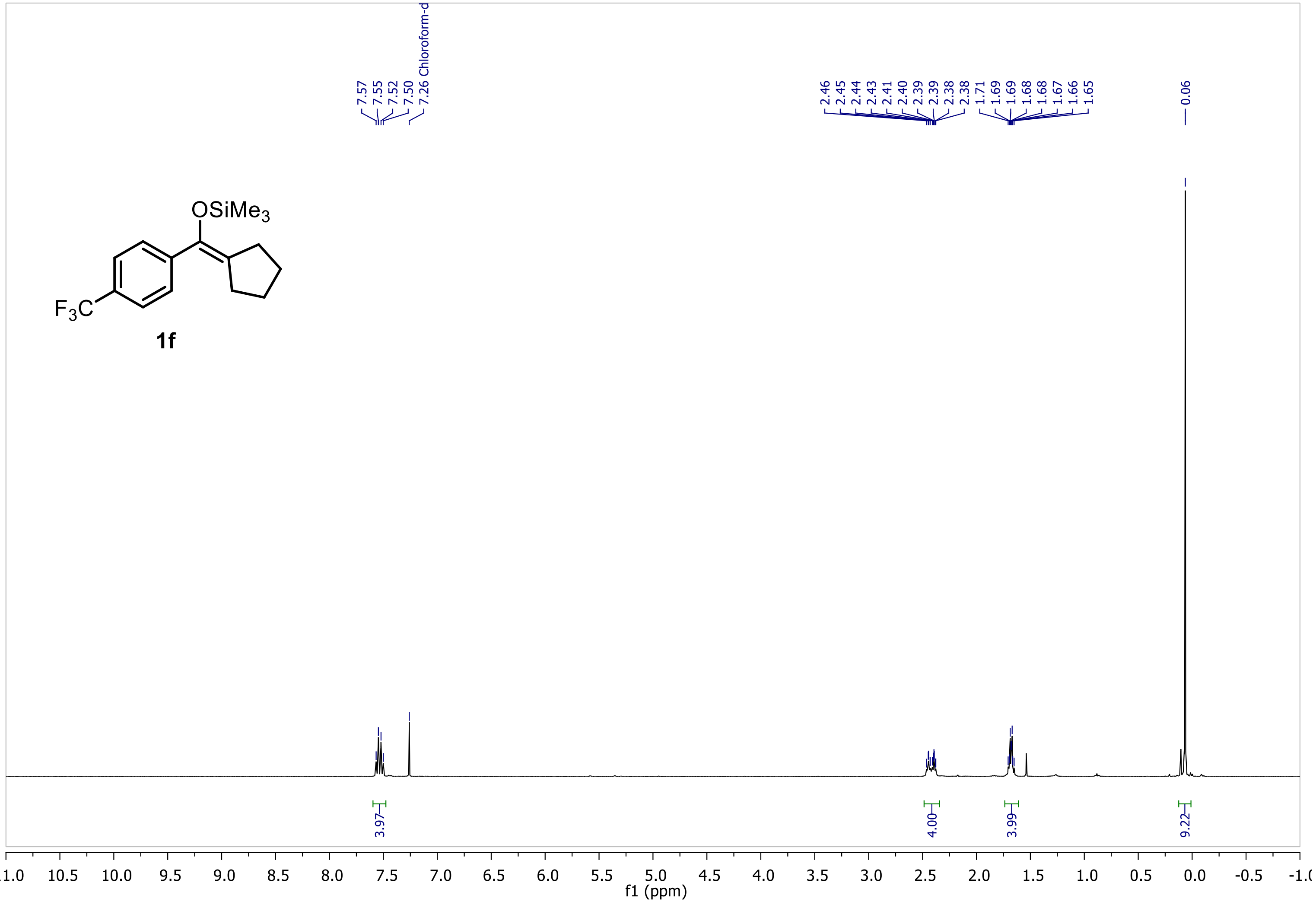

$^{13}C\{^{1}H\}$ NMR (101 MHz, $CDCl_3$)

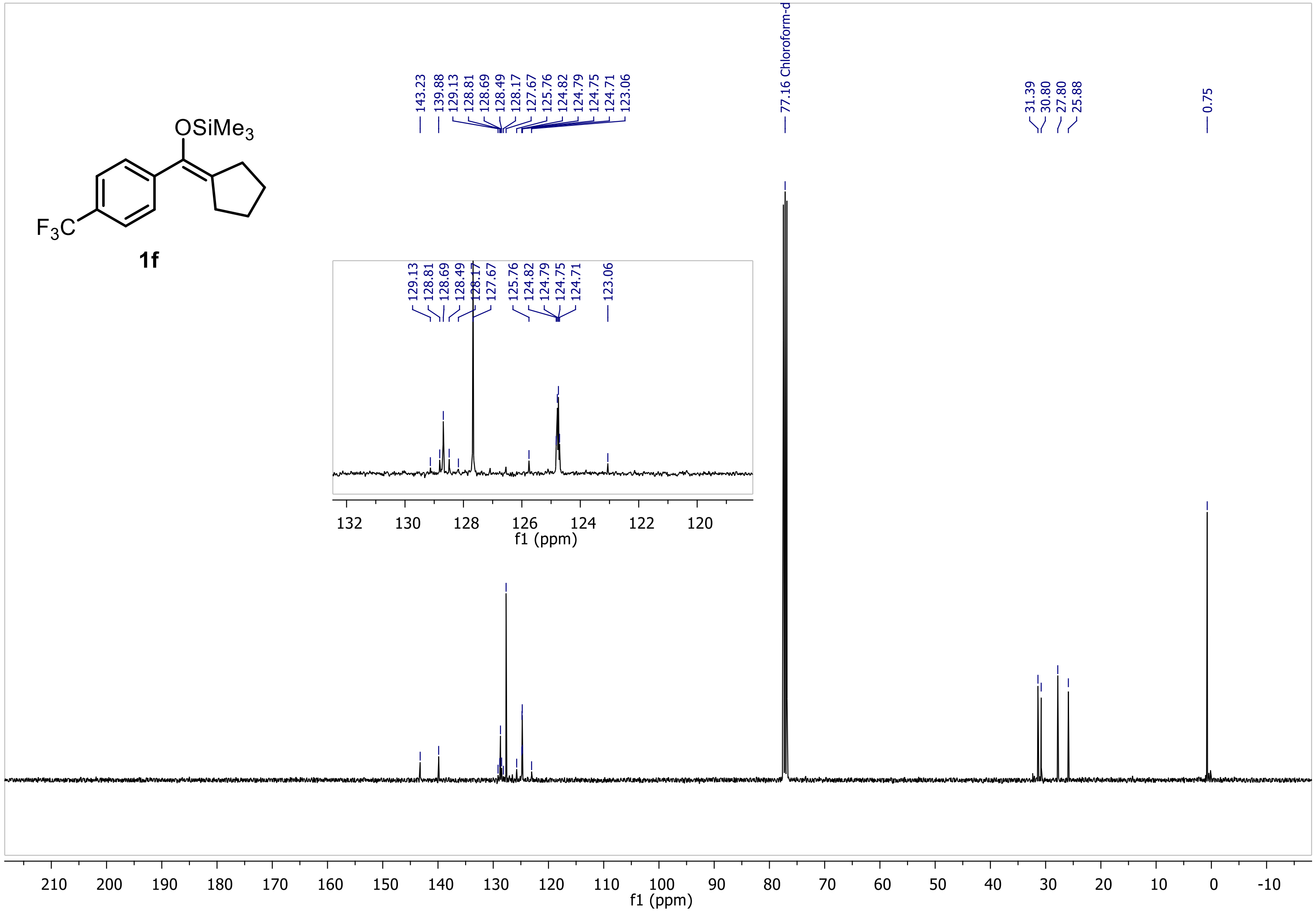

^{19}F NMR (376 MHz, $CDCl_3$)

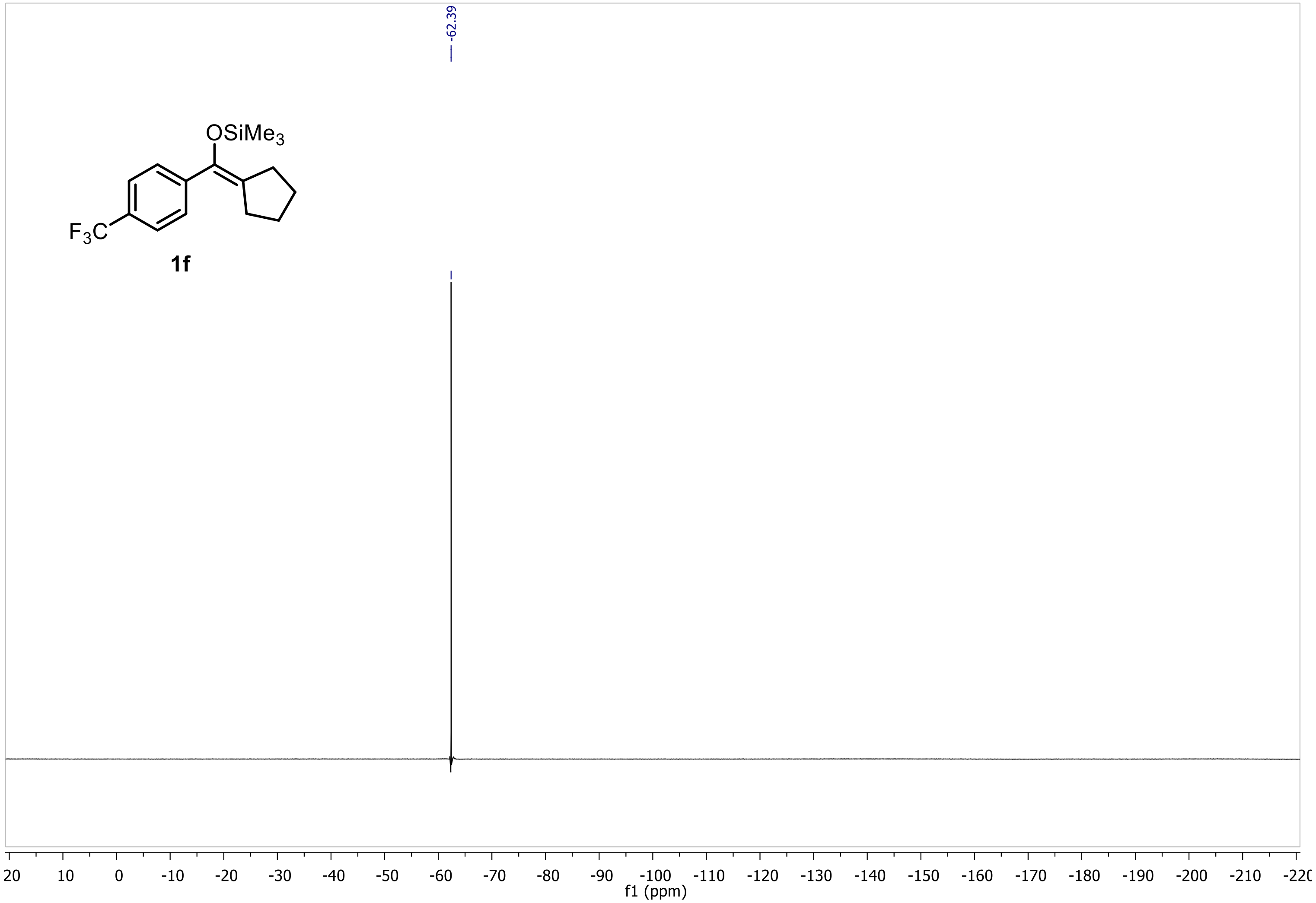

1g – {Cyclopentylidene[3-(trifluoromethyl)phenyl]methoxy}trimethylsilane

^{1}H NMR (400 MHz, $CDCl_3$)

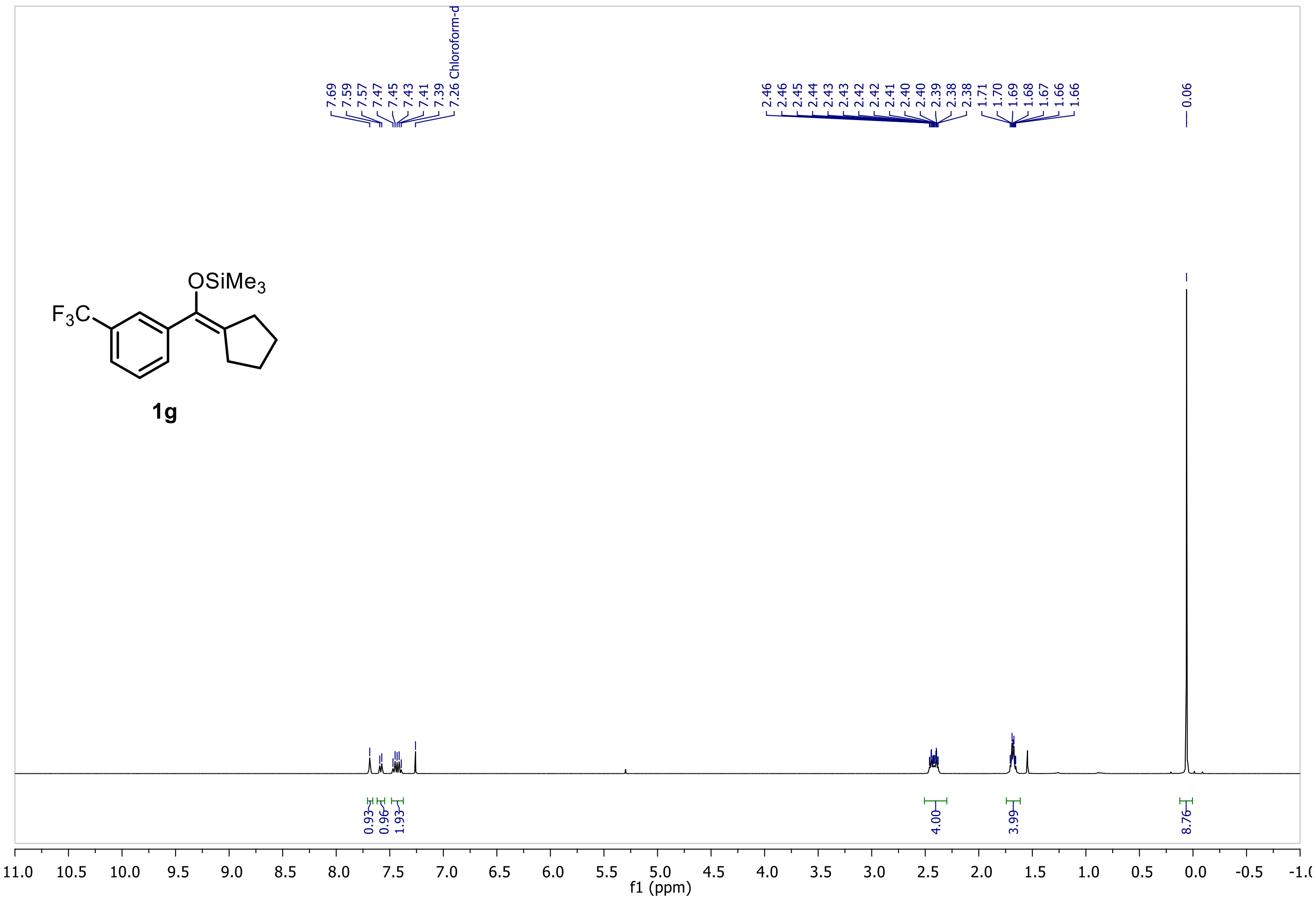

$^{13}C\{^{1}H\}$ NMR (101 MHz, $CDCl_3$)

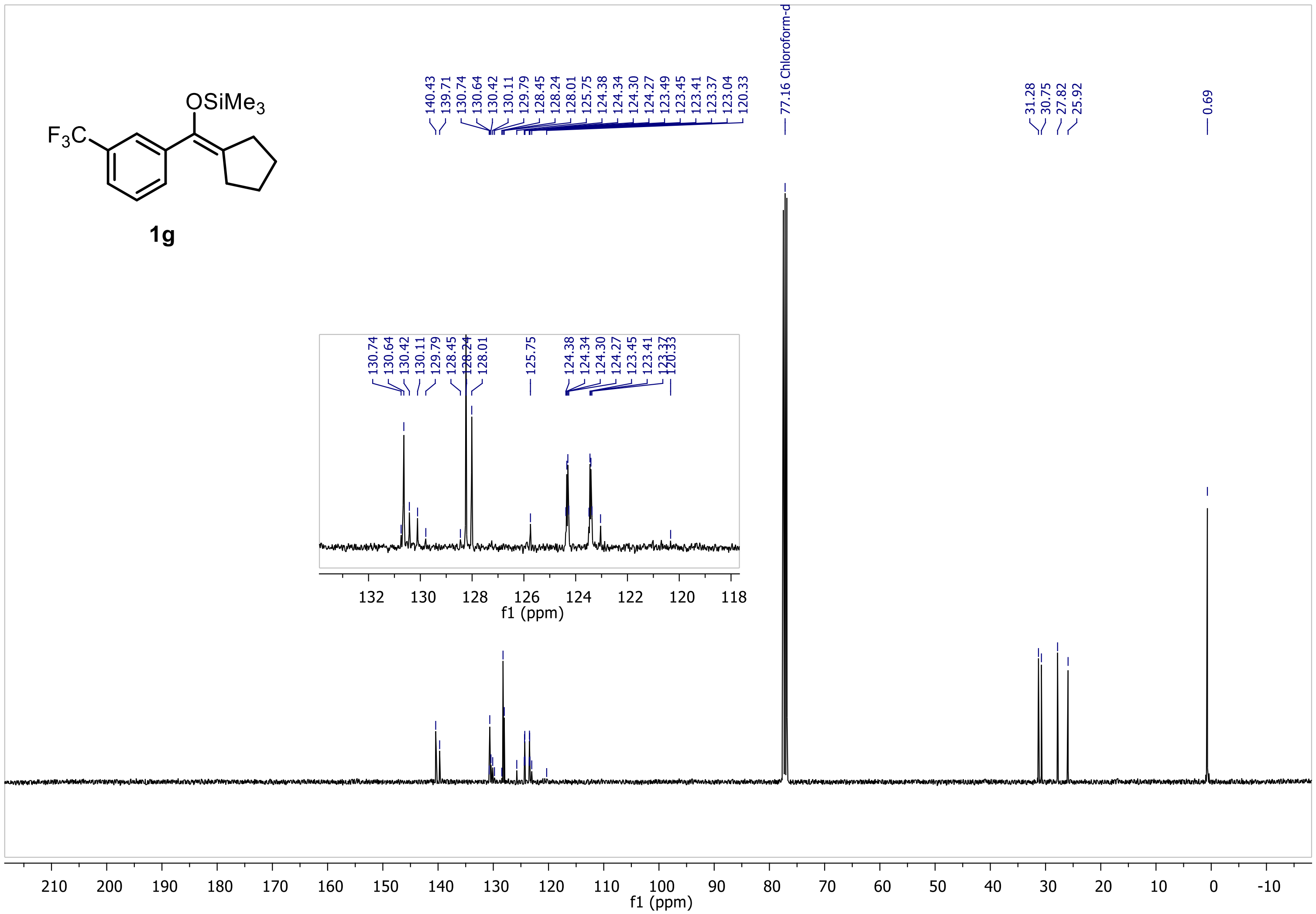

^{19}F NMR (376 MHz, $CDCl_3$)

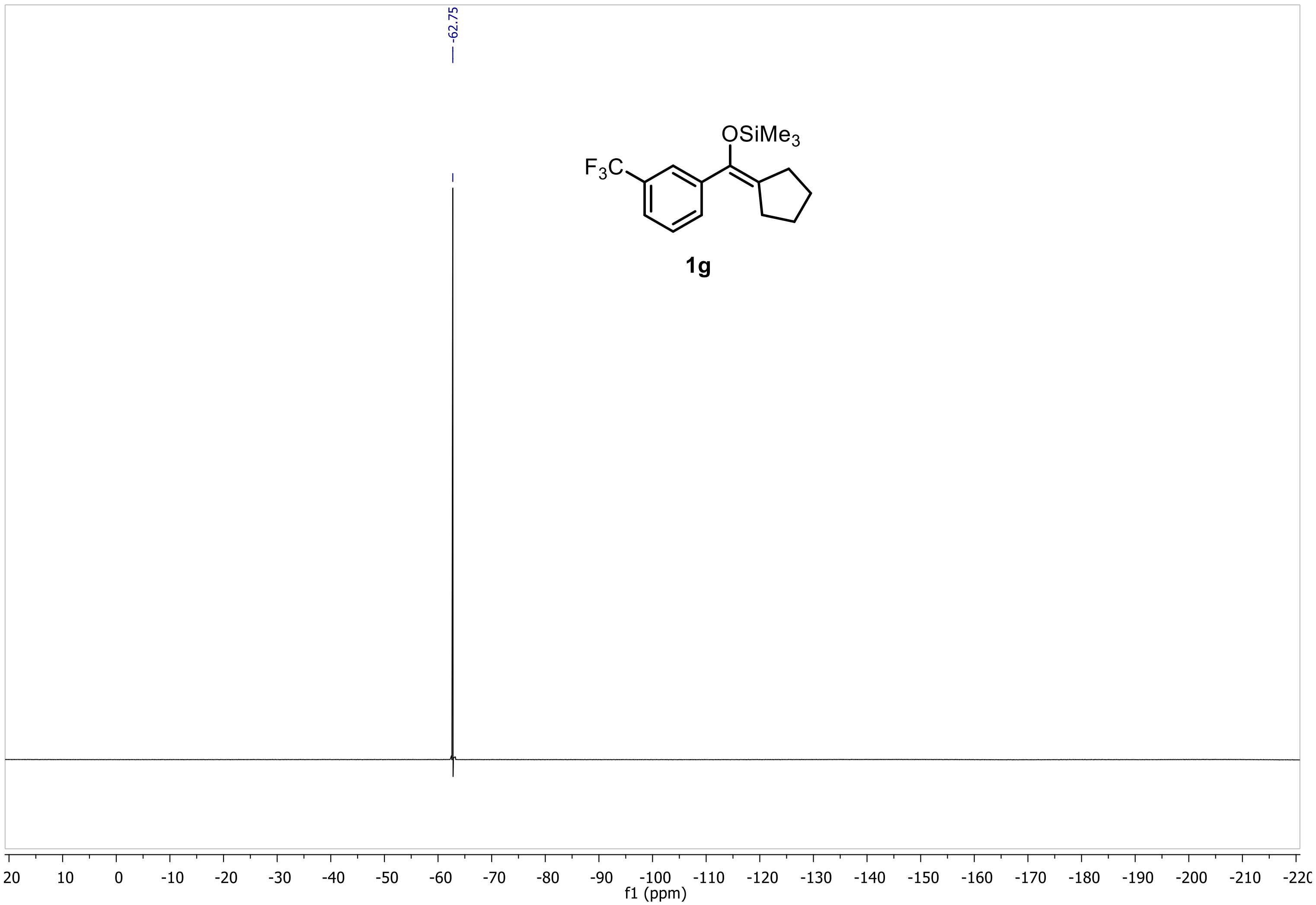

1h – {Cyclopentylidene[2-(trifluoromethyl)phenyl]methoxy}trimethylsilane

^{1}H NMR (600 MHz, $CDCl_3$)

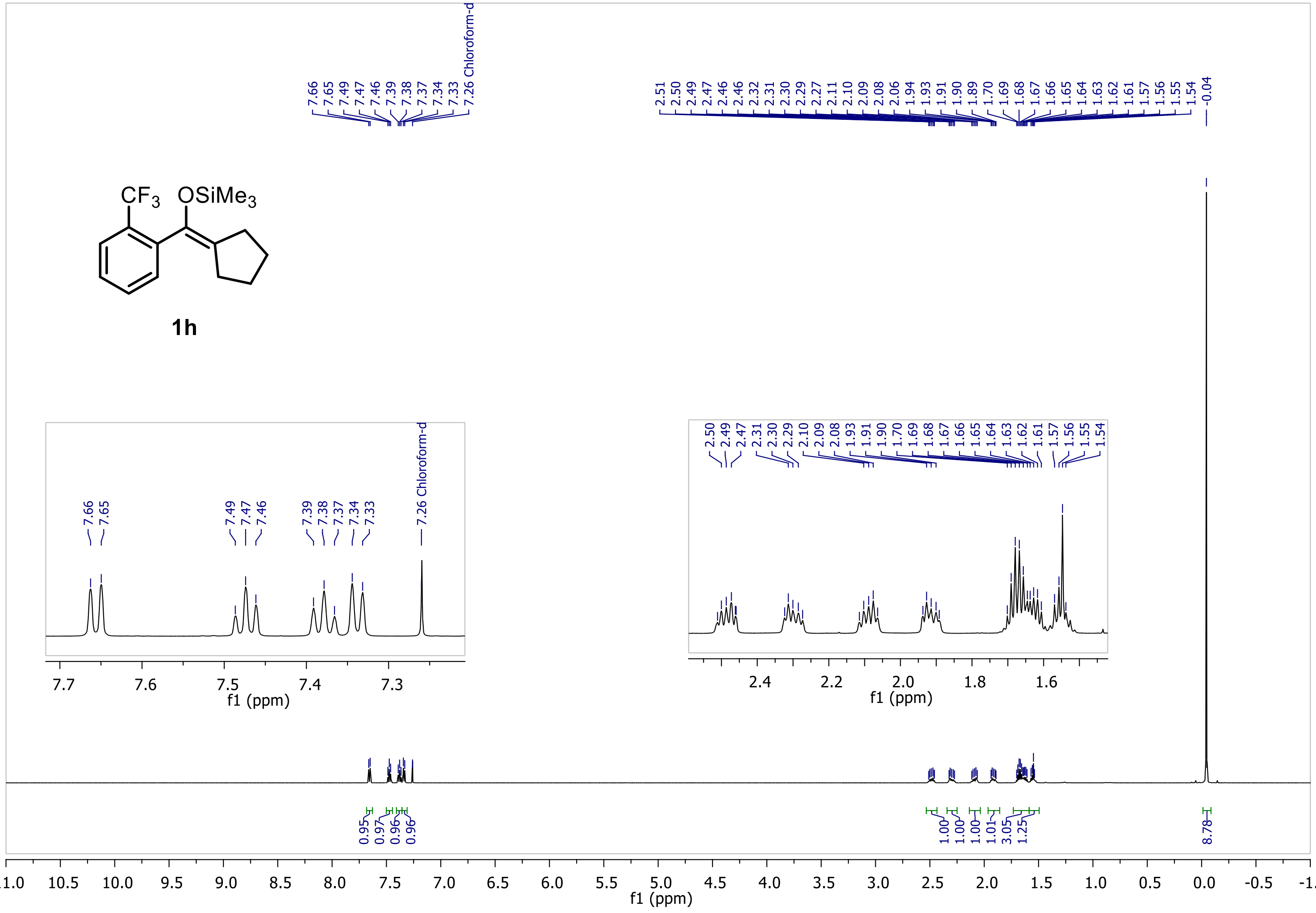

^{13}C (DEPT 135) NMR (151 MHz, $CDCl_3$)

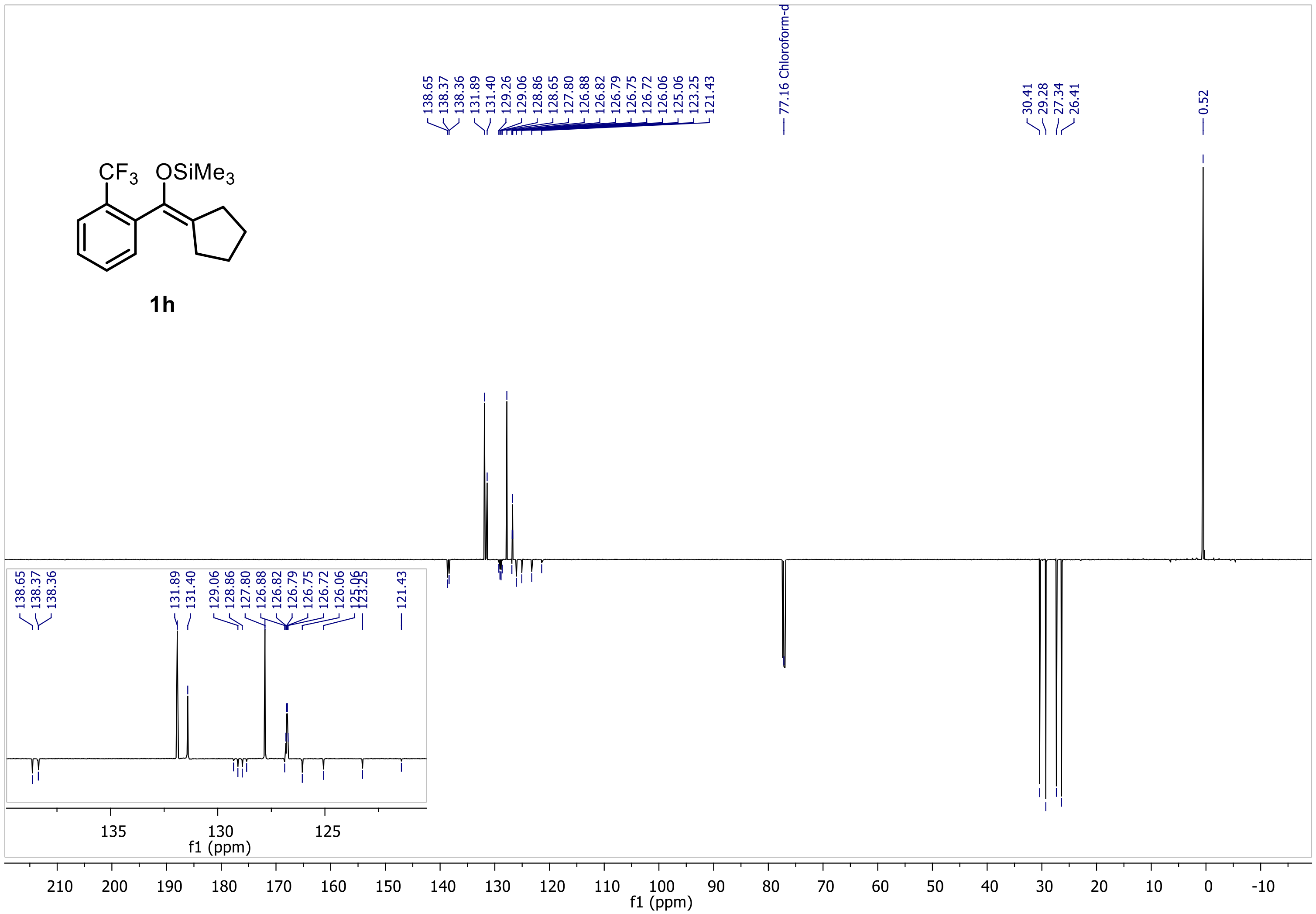

^{19}F NMR (565 MHz, $CDCl_3$)

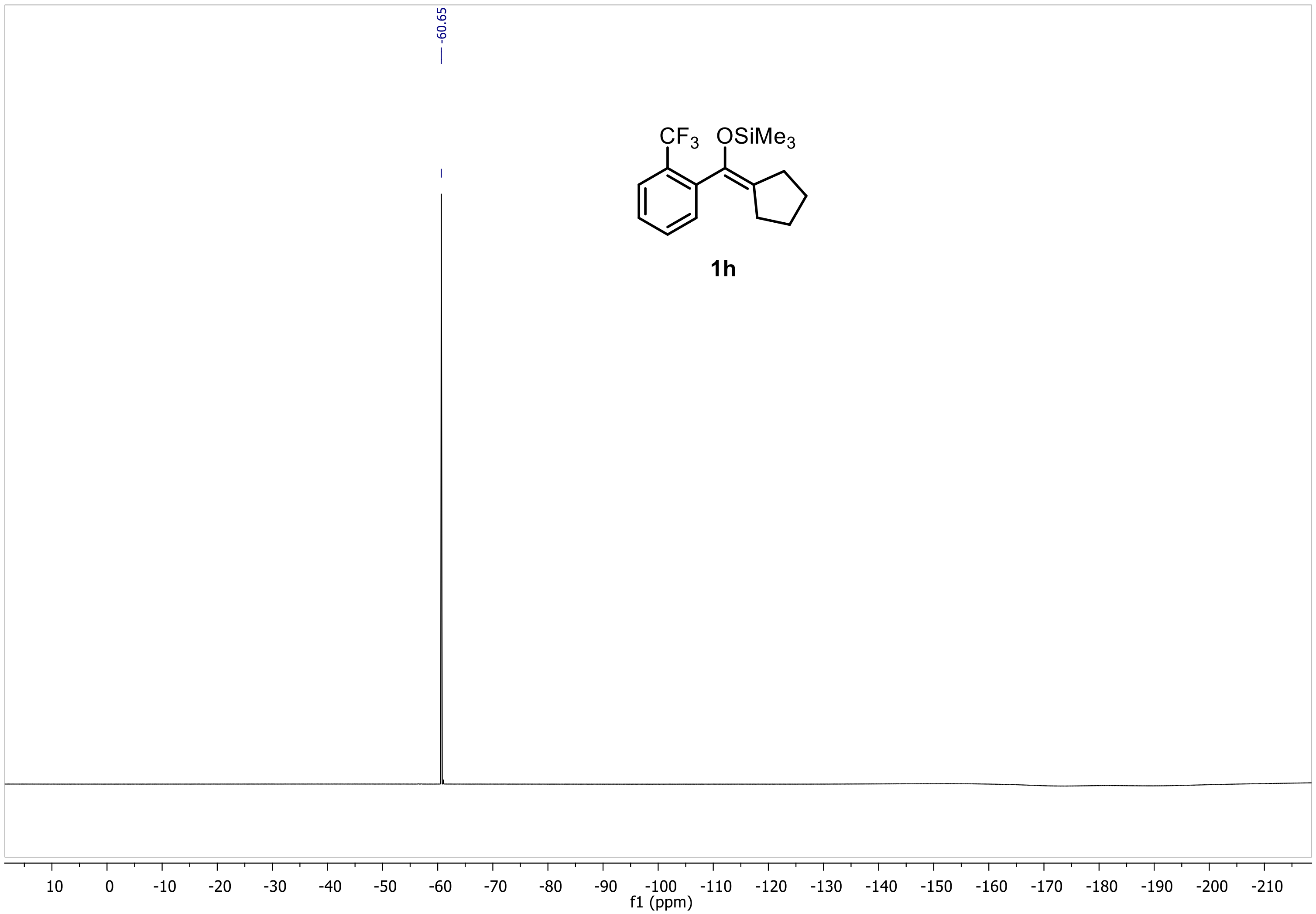

1i – 4-{Cyclopentylidene[(trimethylsilyl)oxy]methyl}benzonitrile

^{1}H NMR (700 MHz, $CDCl_3$)

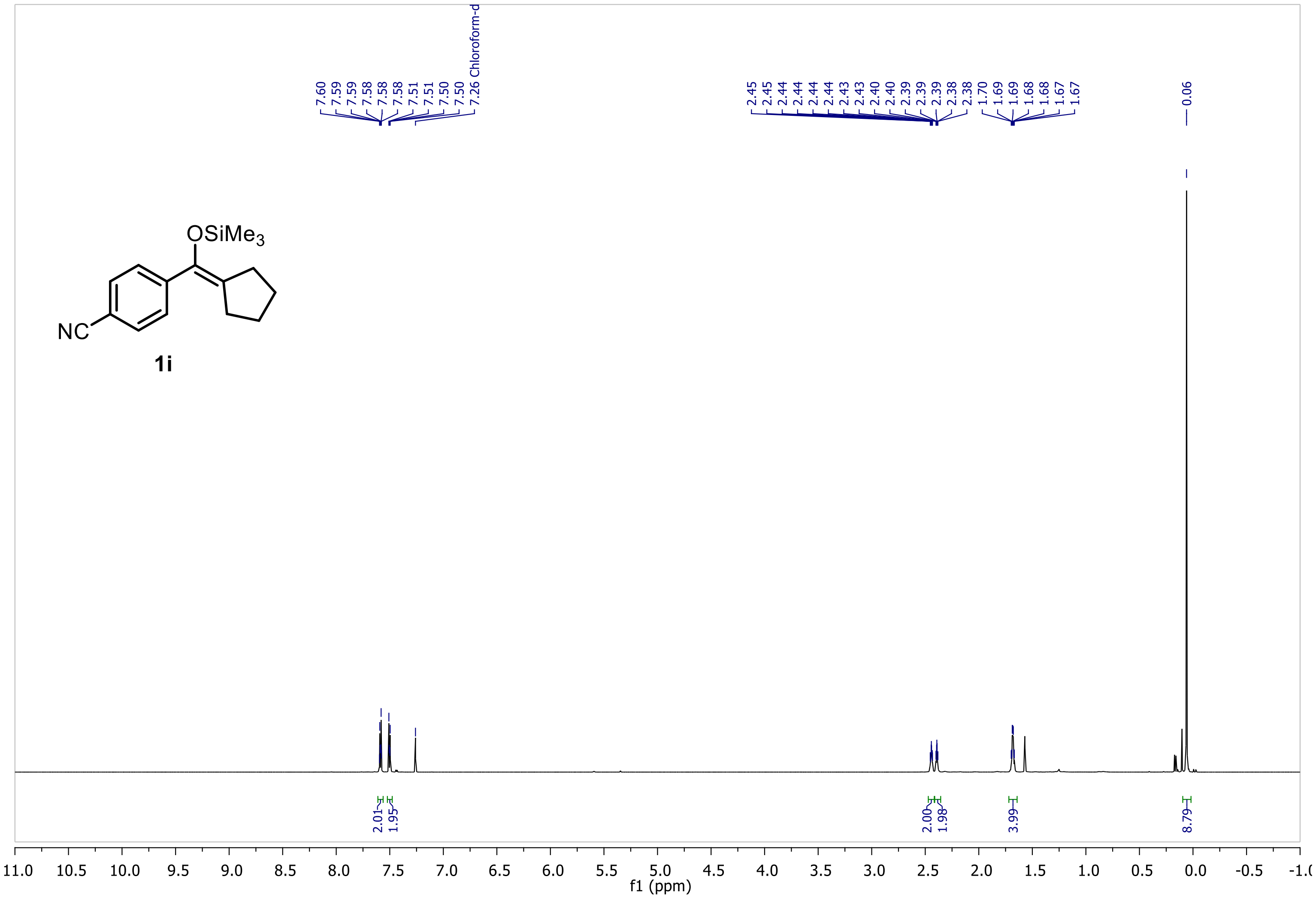

^{13}C (DEPT 135) NMR (176 MHz, $CDCl_3$)

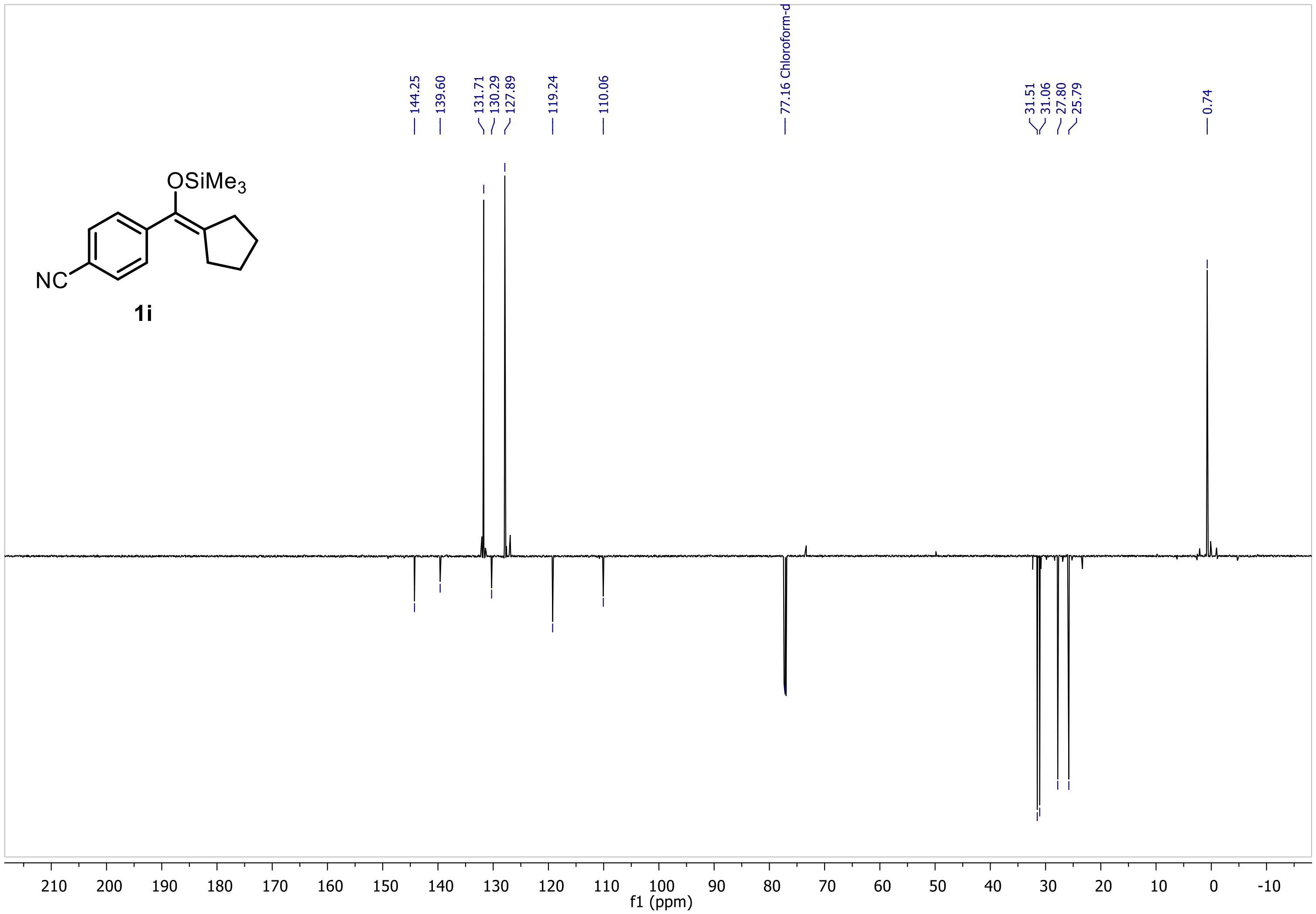

1j – Methyl 4-{cyclopentylidene[(trimethylsilyl)oxy]methyl}benzoate

^{1}H NMR (600 MHz, $CDCl_3$)

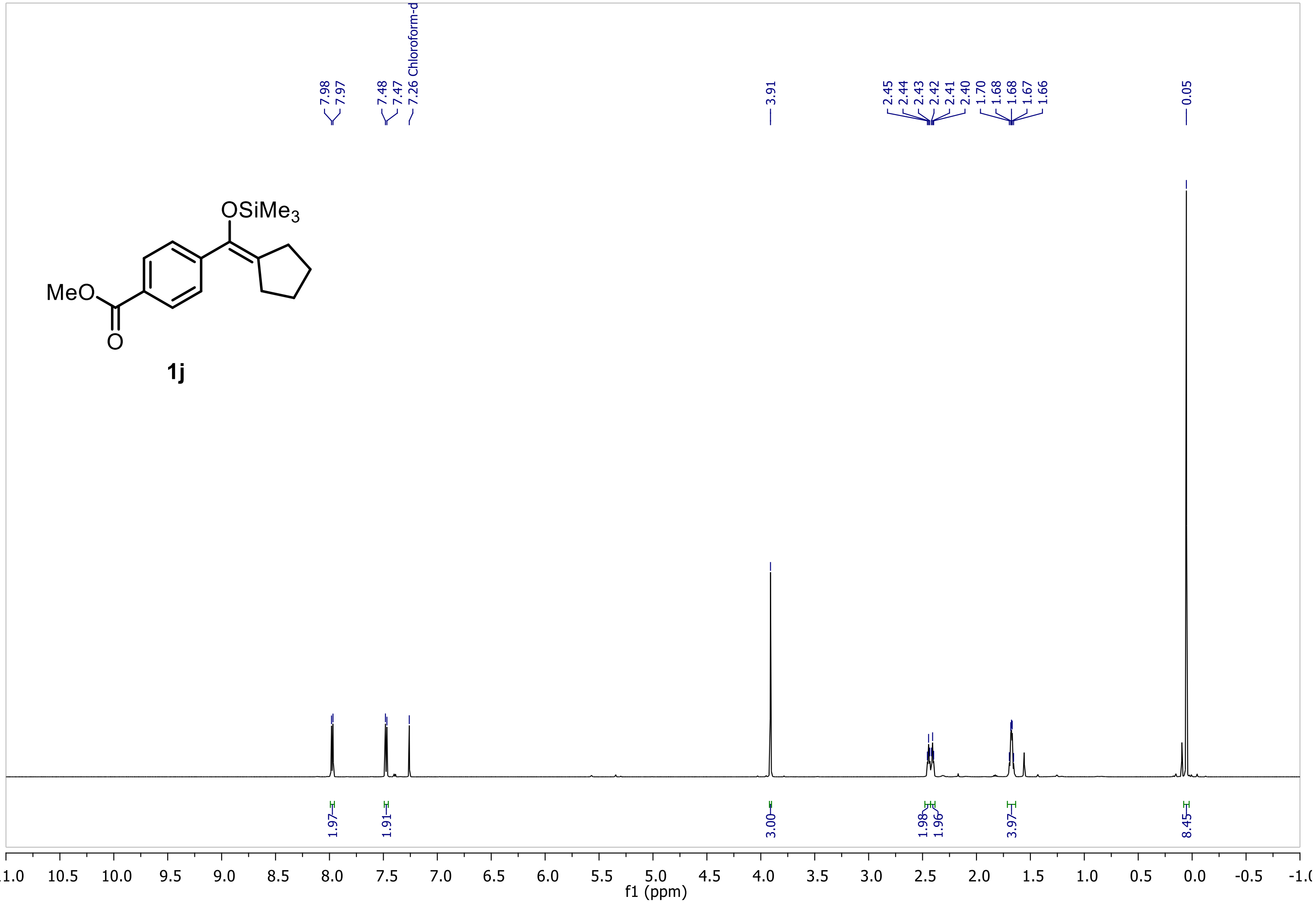

$^{13}C\{^1H\}$ NMR (151 MHz, $CDCl_3$)

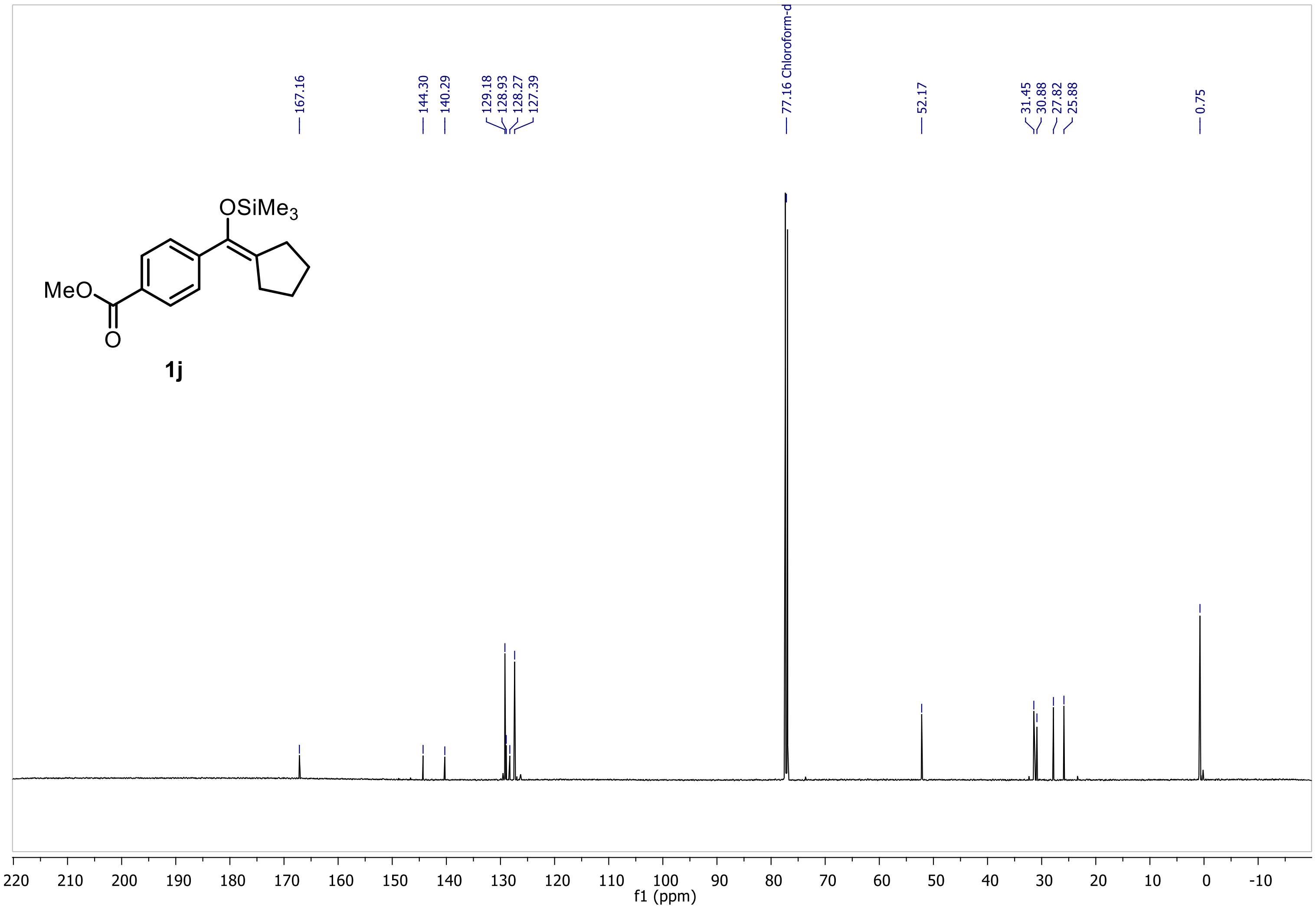

1k – (cyclopentylidene(2,6-dichlorophenyl)methoxy)trimethylsilane

^{1}H NMR (400 MHz, $CDCl_3$)

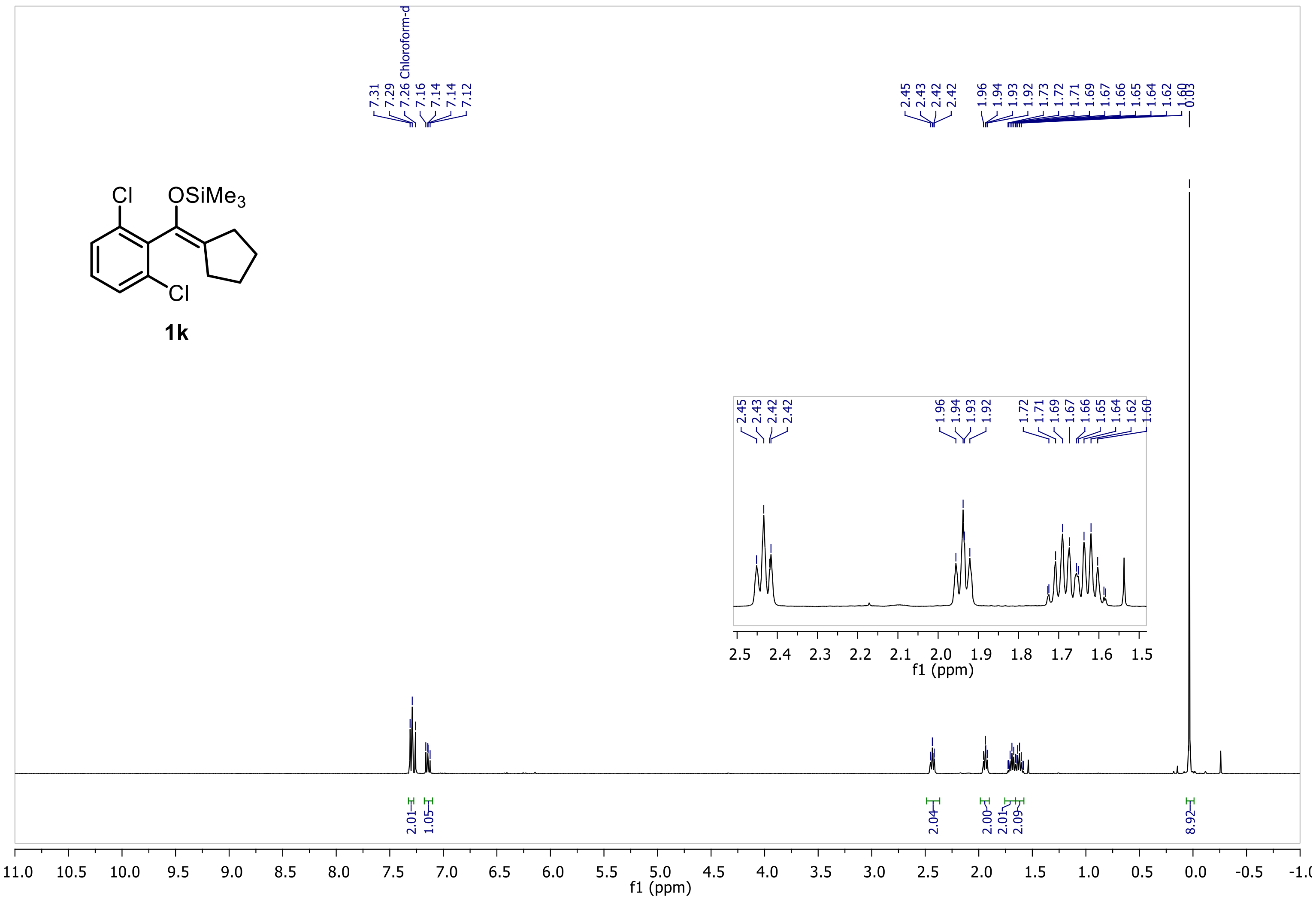

^{13}C (DEPT 135) NMR (101 MHz, $CDCl_3$)

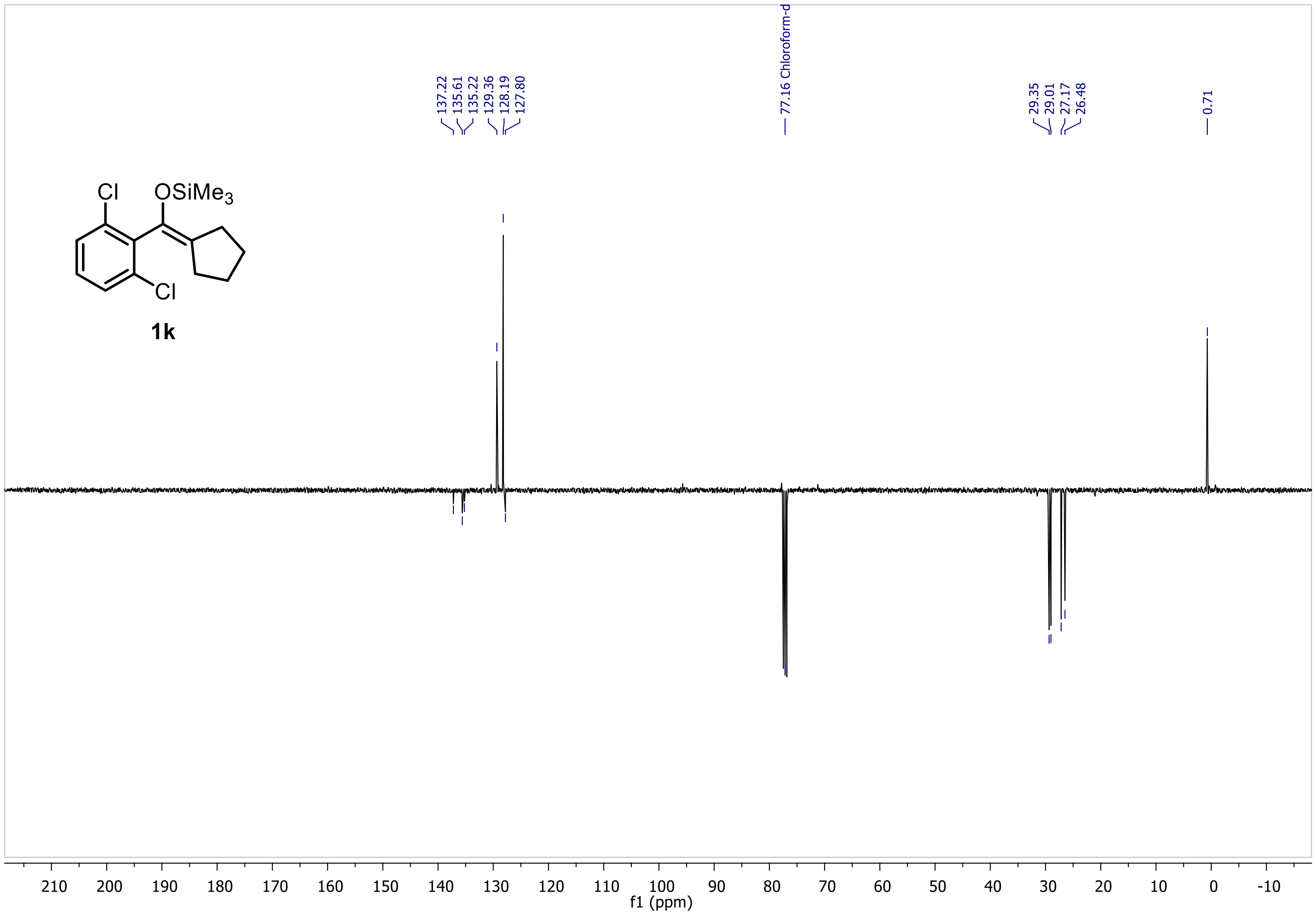

1l – [(4-Bromophenyl)(cyclopentylidene)methoxy]trimethylsilane

^{1}H NMR (400 MHz, $CDCl_3$)

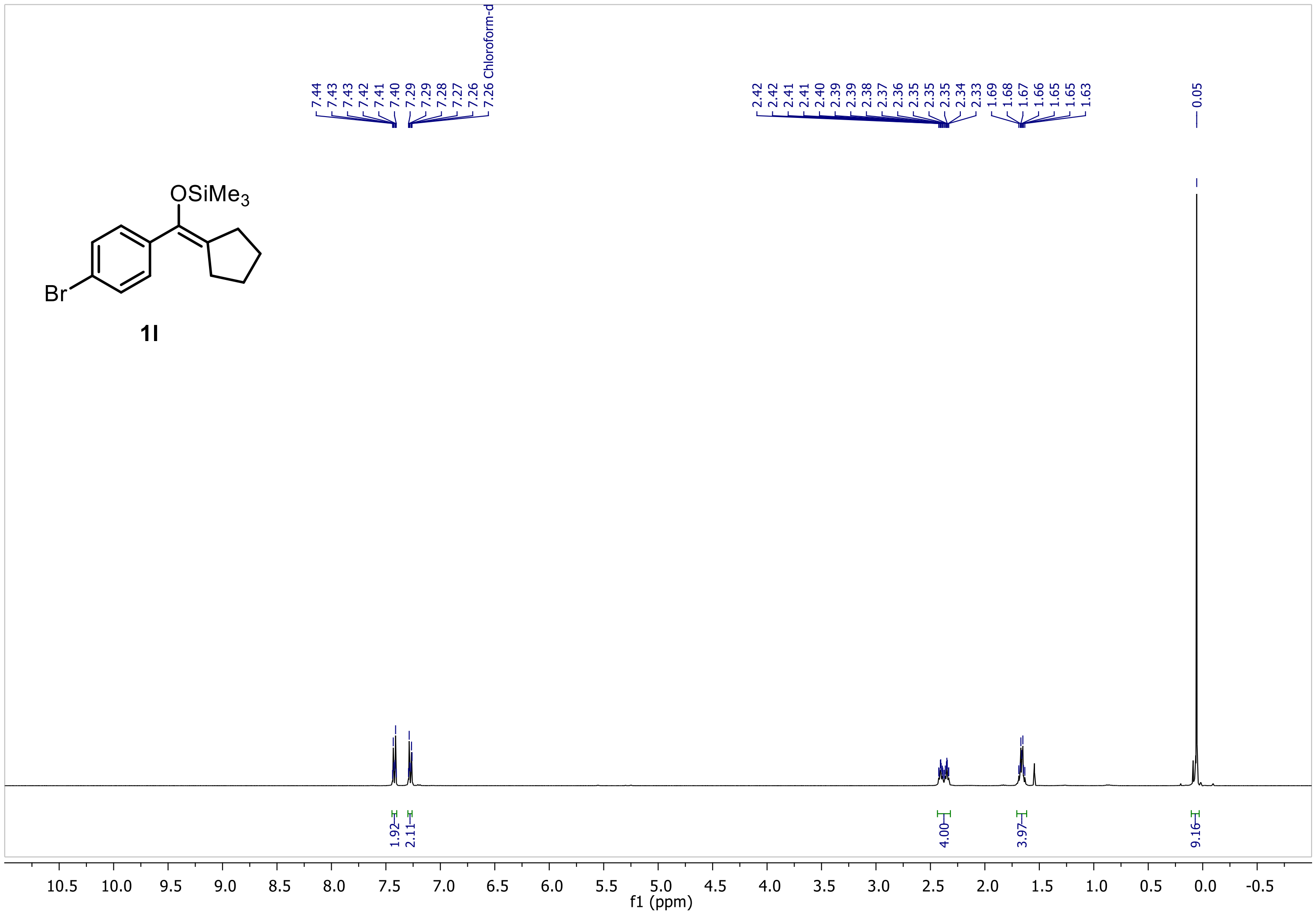

$^{13}C\{^1H\}$ NMR (101 MHz, $CDCl_3$)

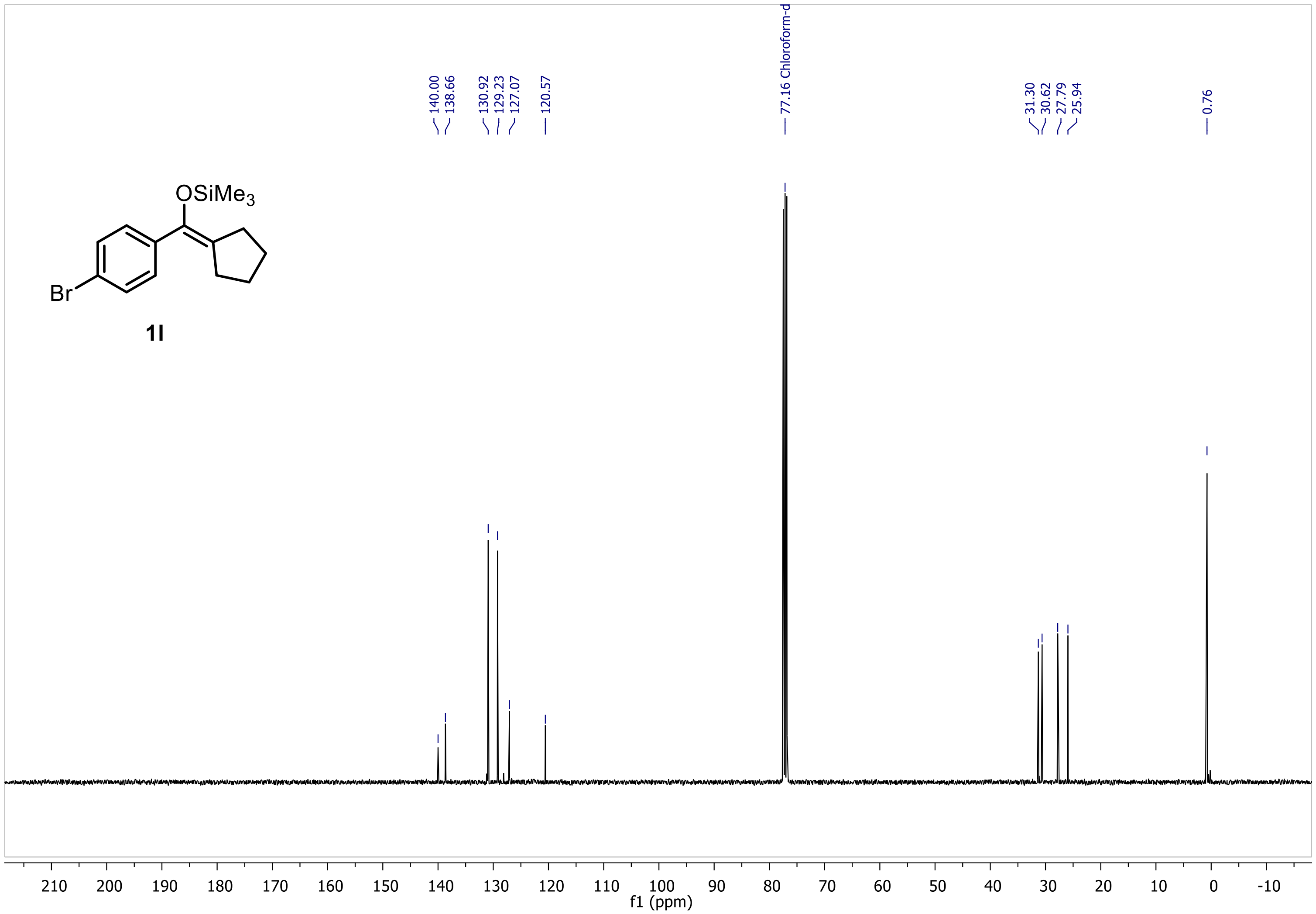

1m – [Cyclopentylidene(phenyl)methoxy]trimethylsilane

^{1}H NMR (400 MHz, $CDCl_3$)

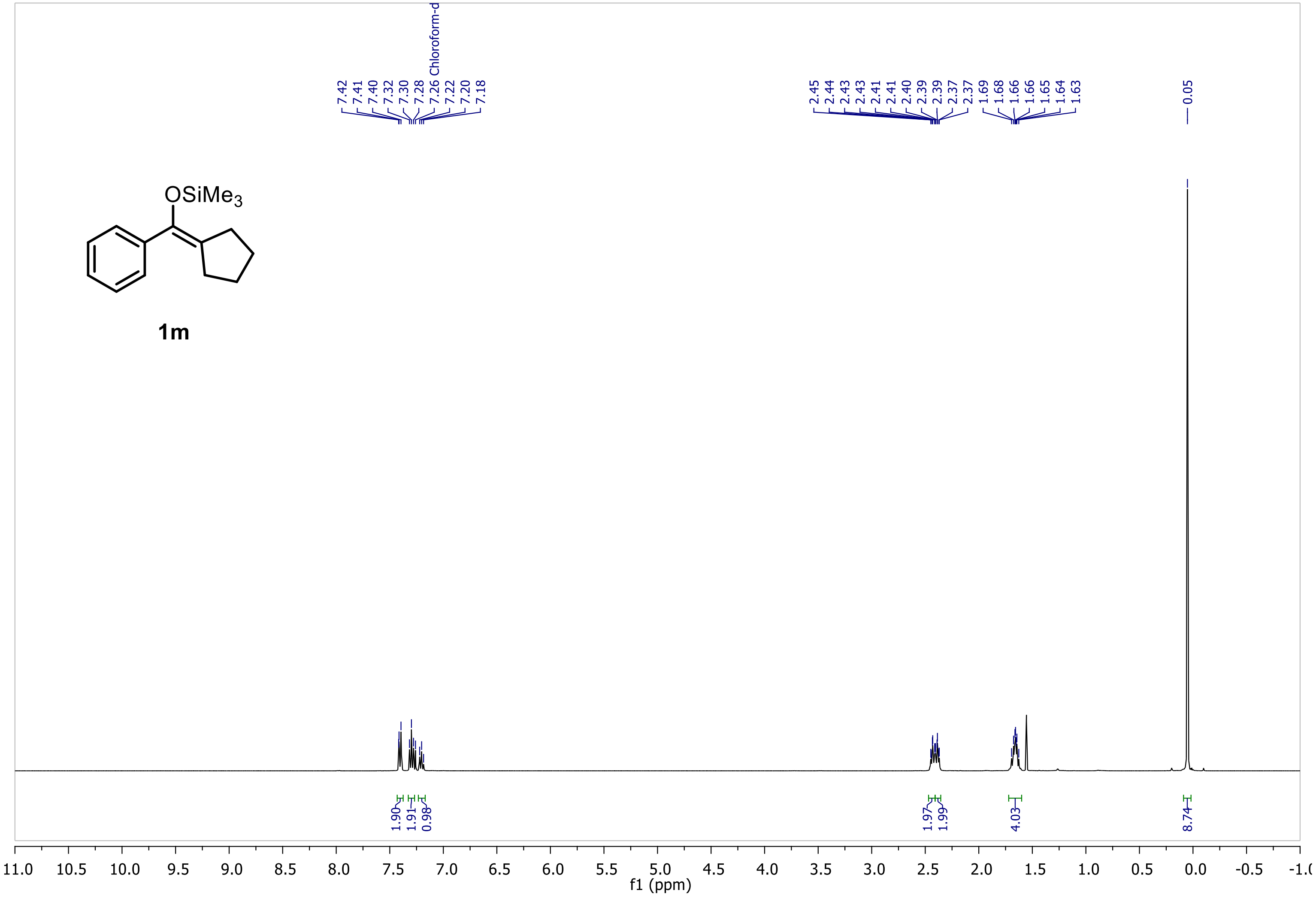

1n – [Cyclopentylidene(naphthalen-2-yl)methoxy]trimethylsilane

^{1}H NMR (400 MHz, $CDCl_3$)

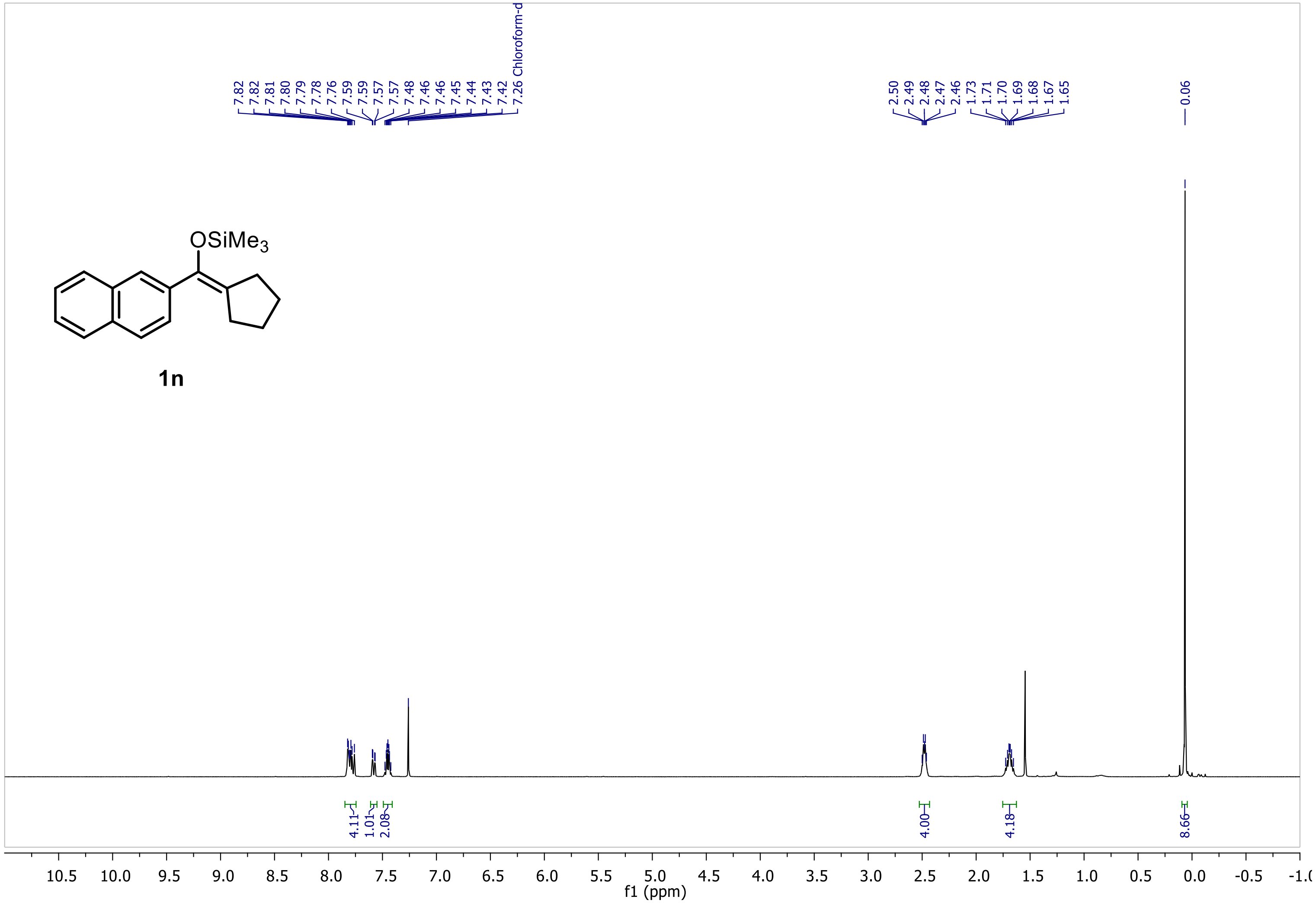

$^{13}C\{^{1}H\}$ NMR (151 MHz, $CDCl_3$)

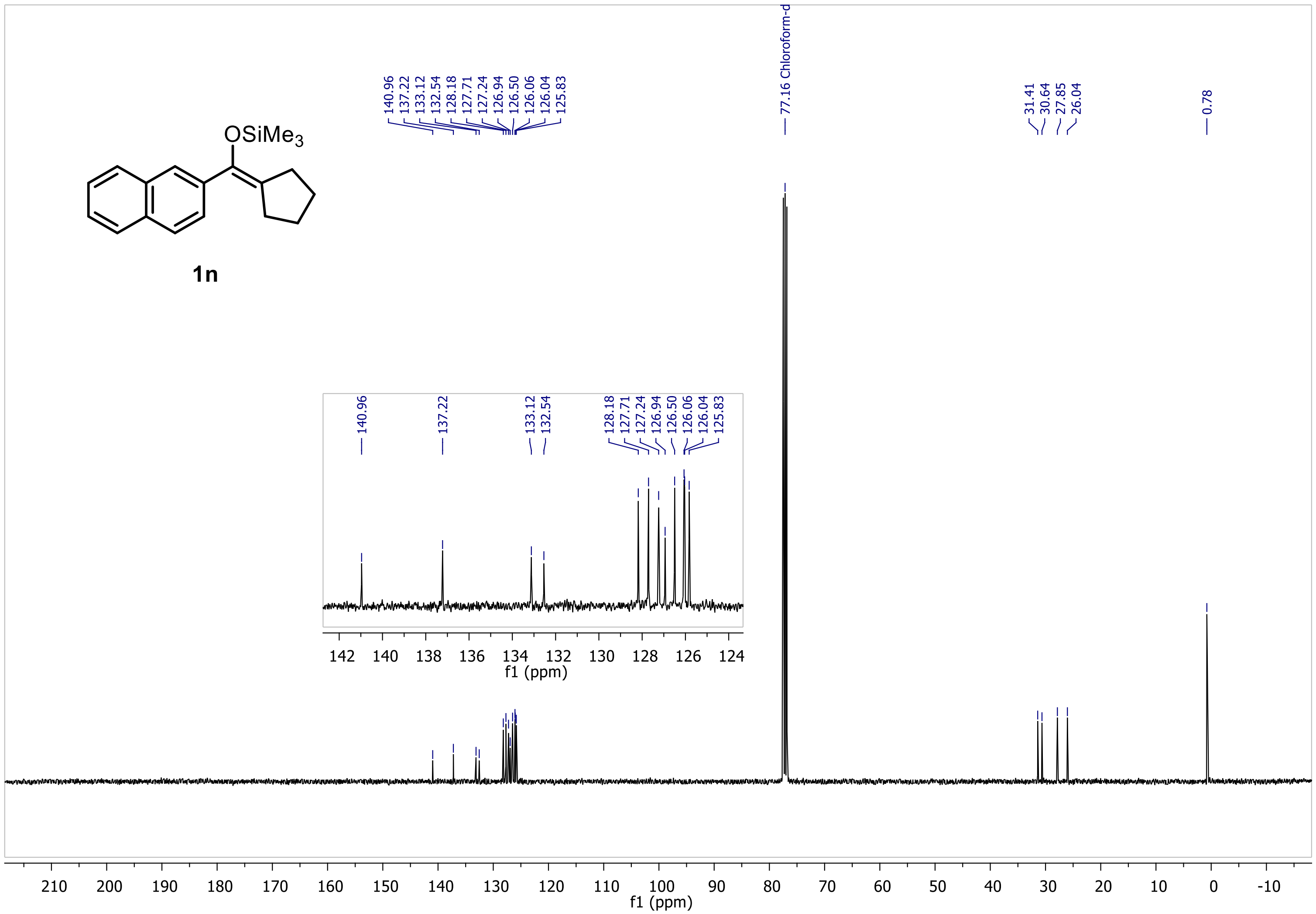

1o – {Cyclohexylidene[4-(trifluoromethyl)phenyl]methoxy}trimethylsilane

^{1}H NMR (400 MHz, $CDCl_3$)

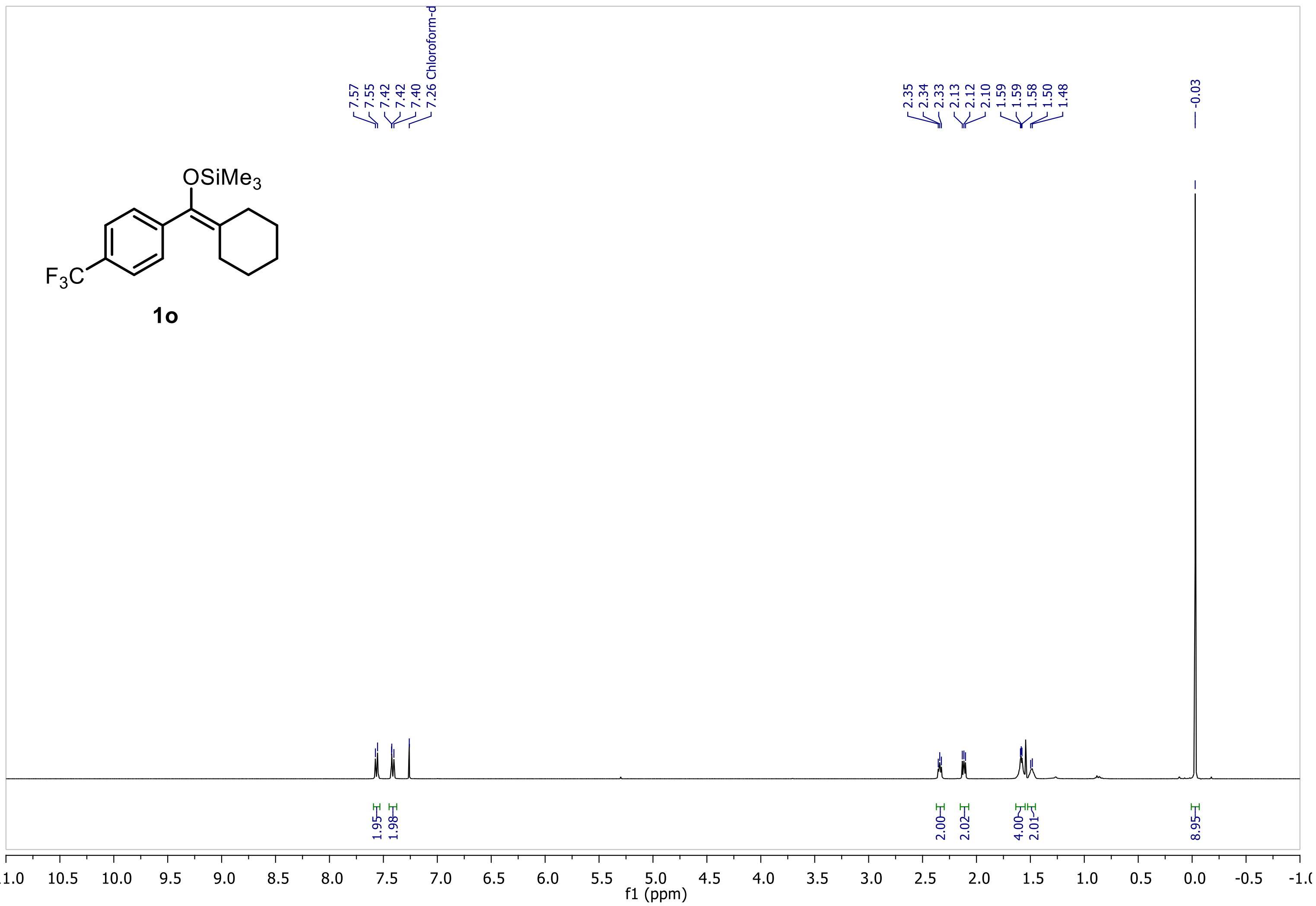

$^{13}C\{^1H\}$ NMR (101 MHz, $CDCl_3$)

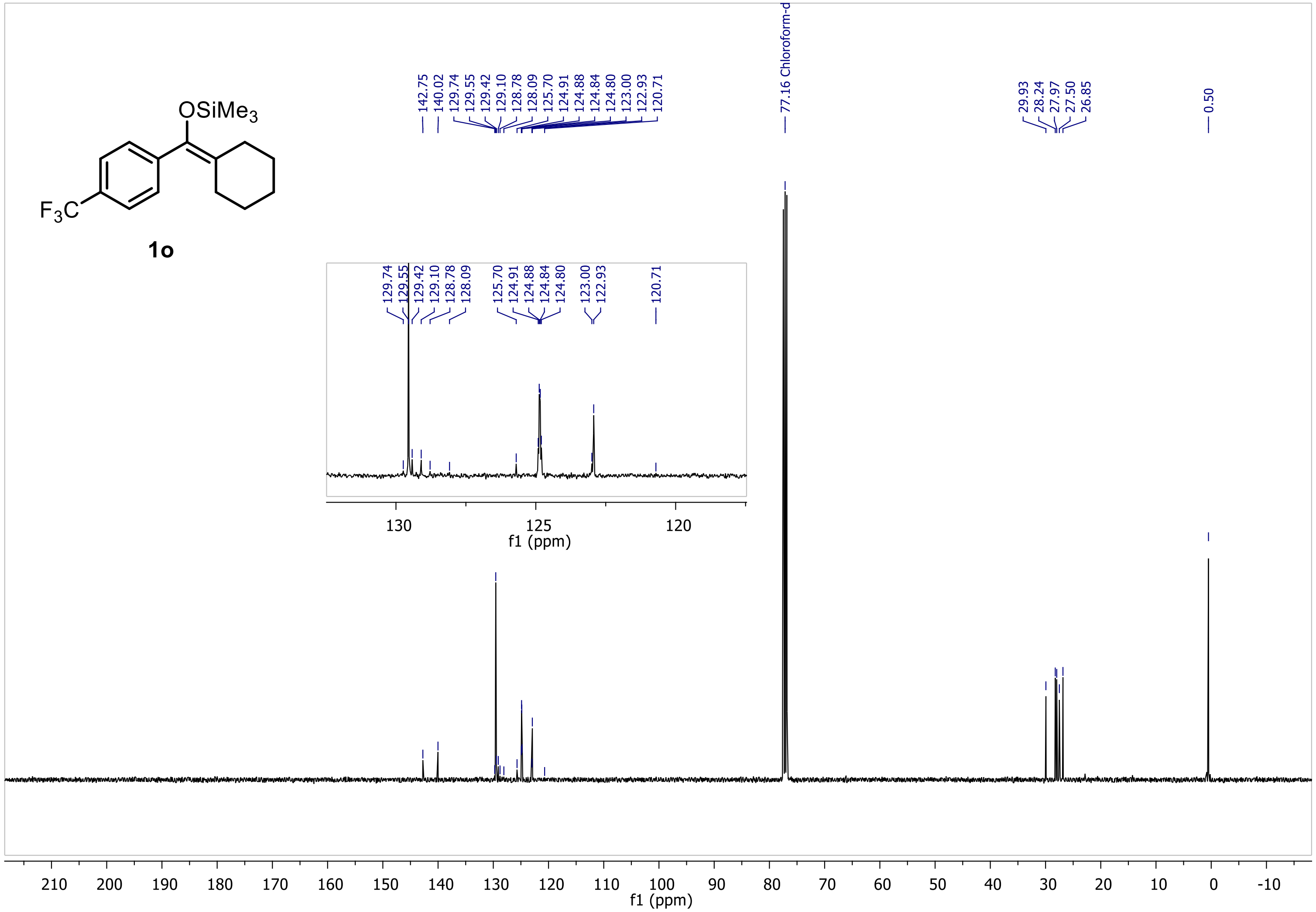

^{19}F NMR (376 MHz, $CDCl_3$)

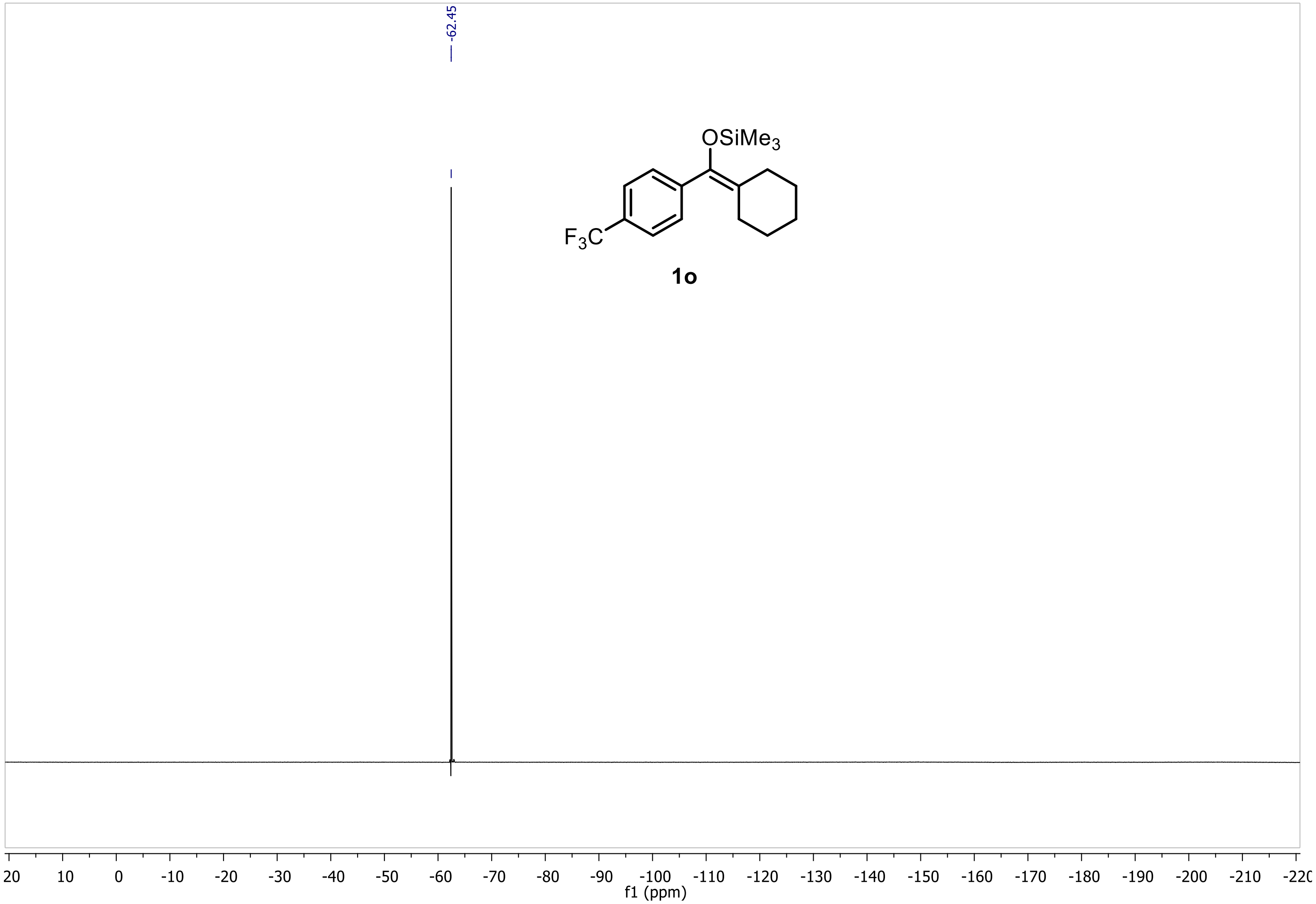

1o

1p – [Benzo[*b*]thiophen-2-yl(cyclohexylidene)methoxy]trimethylsilane

^{1}H NMR (400 MHz, $CDCl_3$)

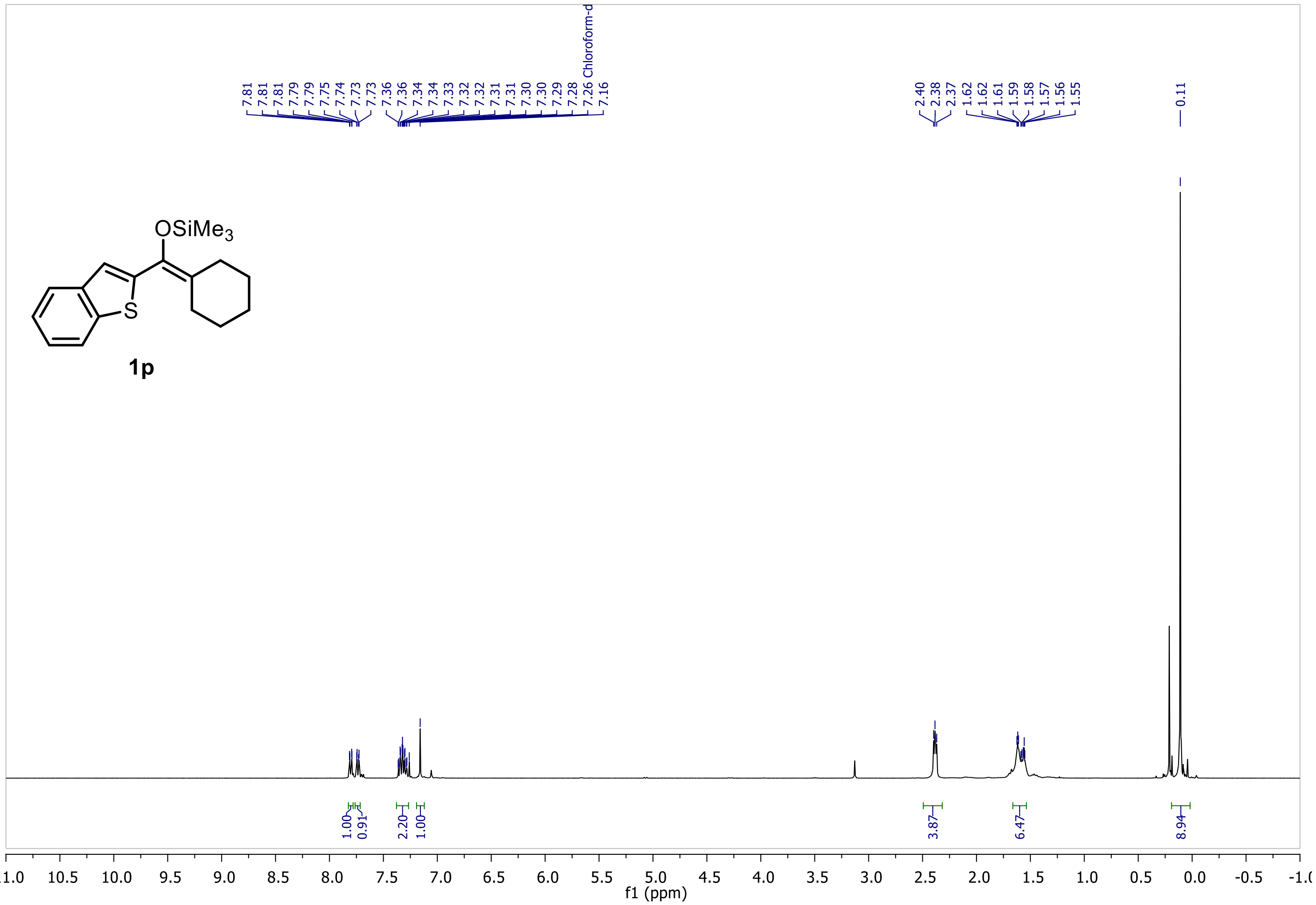

^{13}C (DEPT 135) NMR (101 MHz, $CDCl_3$)

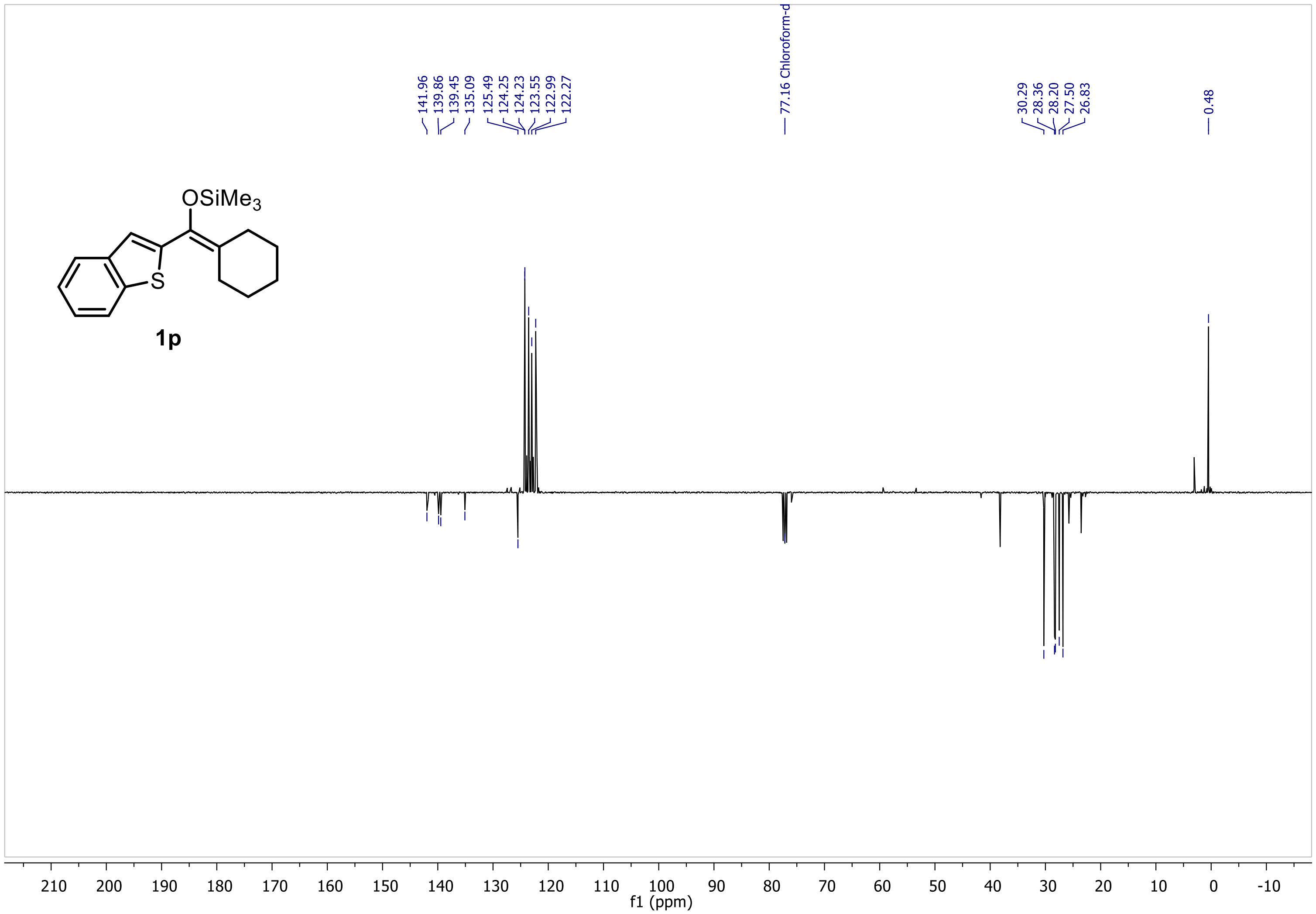

1q – {[3-(*tert*-Butyl)cyclopentylidene][4-(trifluoromethyl)phenyl]methoxy}trimethylsilane

^{1}H NMR (600 MHz, $CDCl_3$)

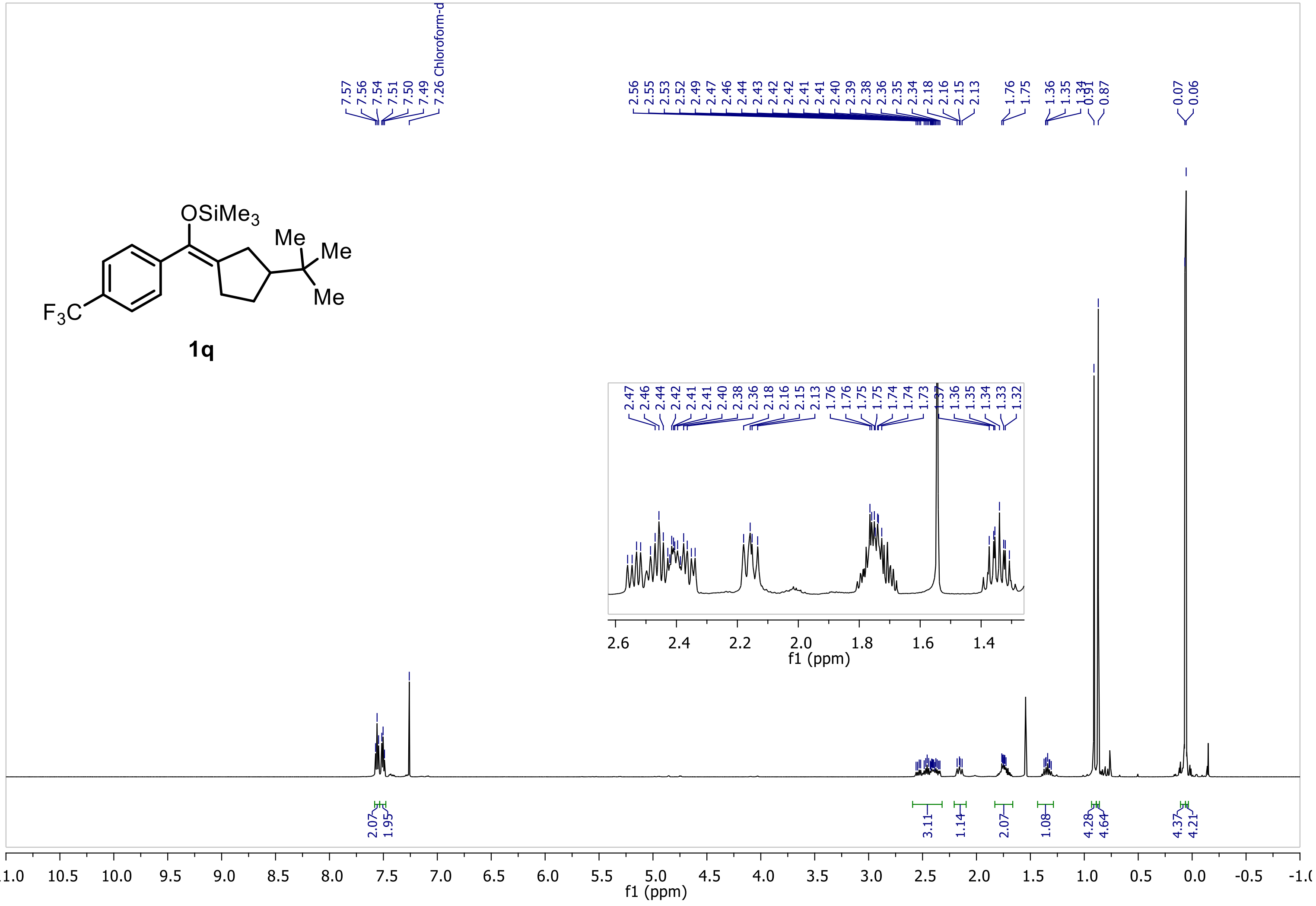

$^{13}C\{^{1}H\}$ NMR (151 MHz, $CDCl_3$)

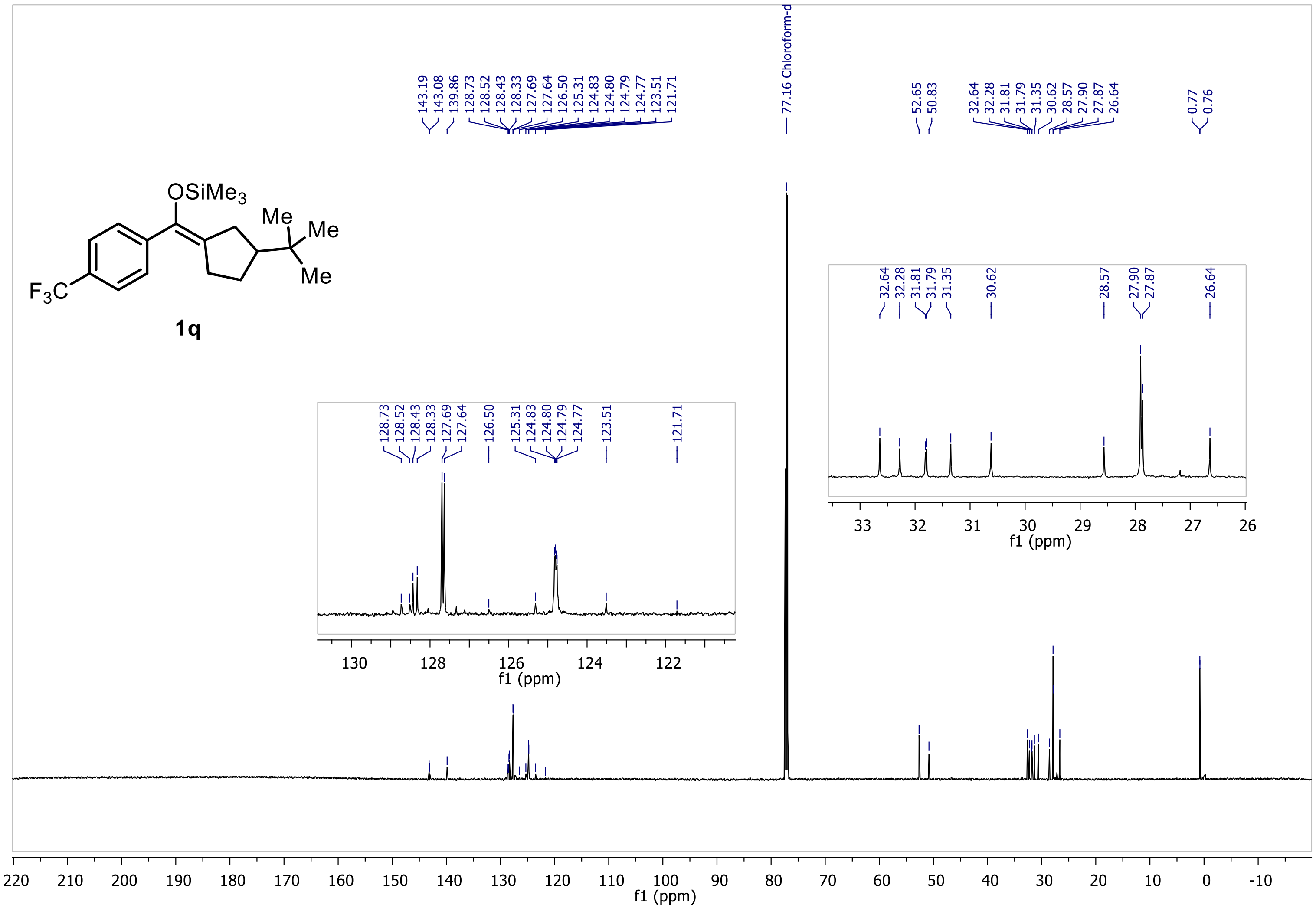

^{19}F NMR (565 MHz, $CDCl_3$)

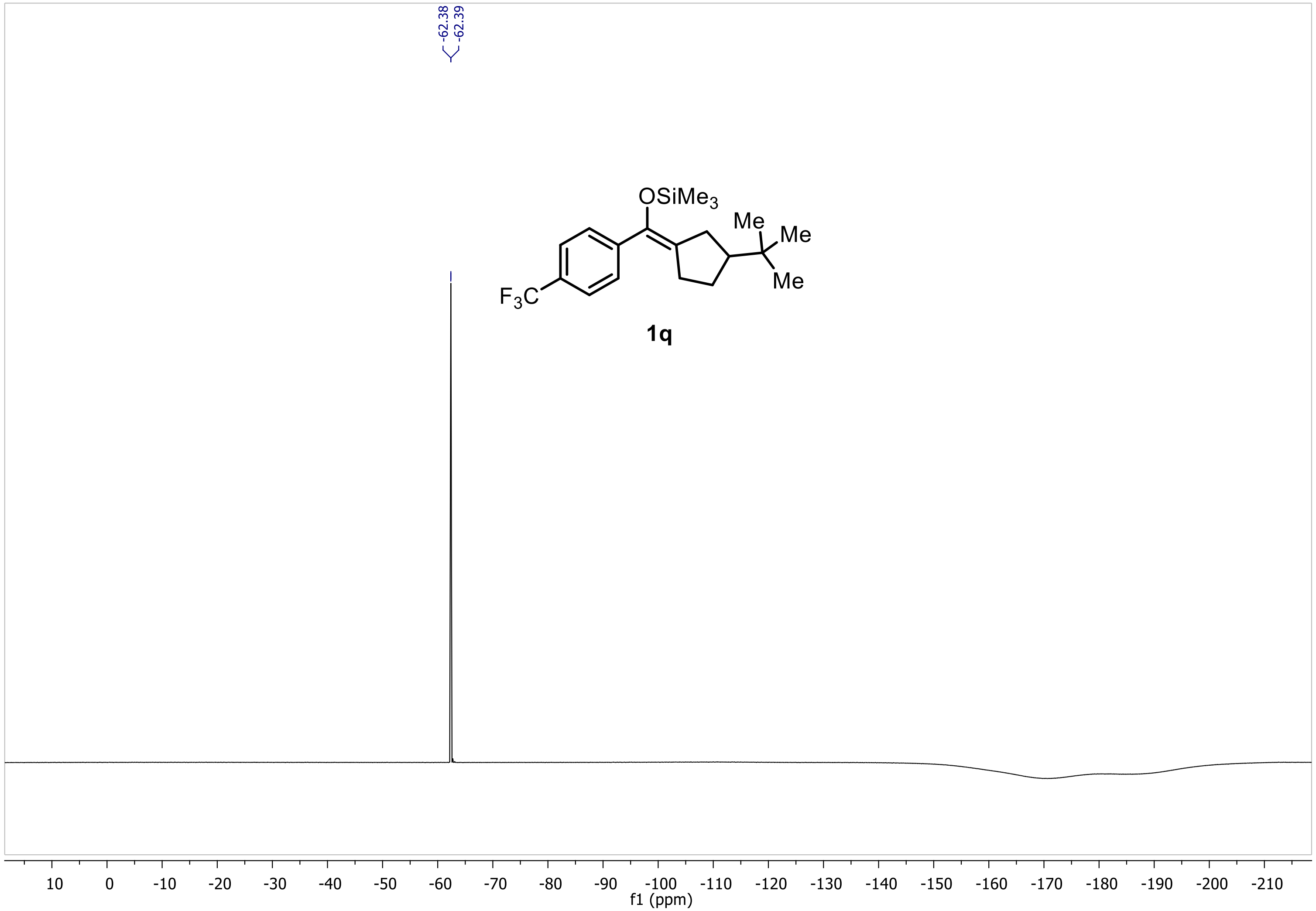

1r – Trimethyl{(3-methylcyclopentylidene)[4-(trifluoromethyl)phenyl]methoxy}silane

^{1}H NMR (400 MHz, CDCl$_3$)

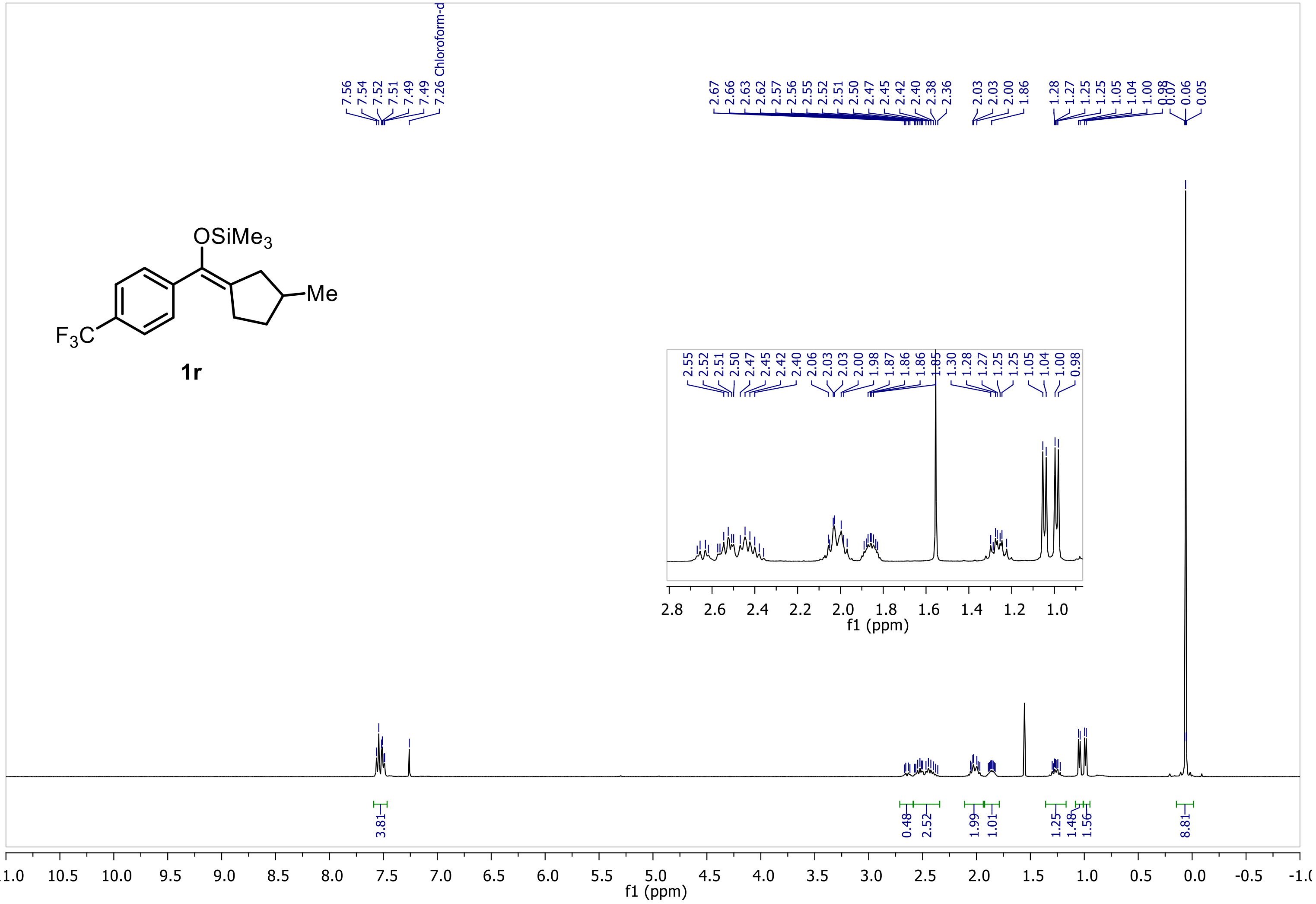

$^{13}C\{^1H\}$ NMR (151 MHz, $CDCl_3$)

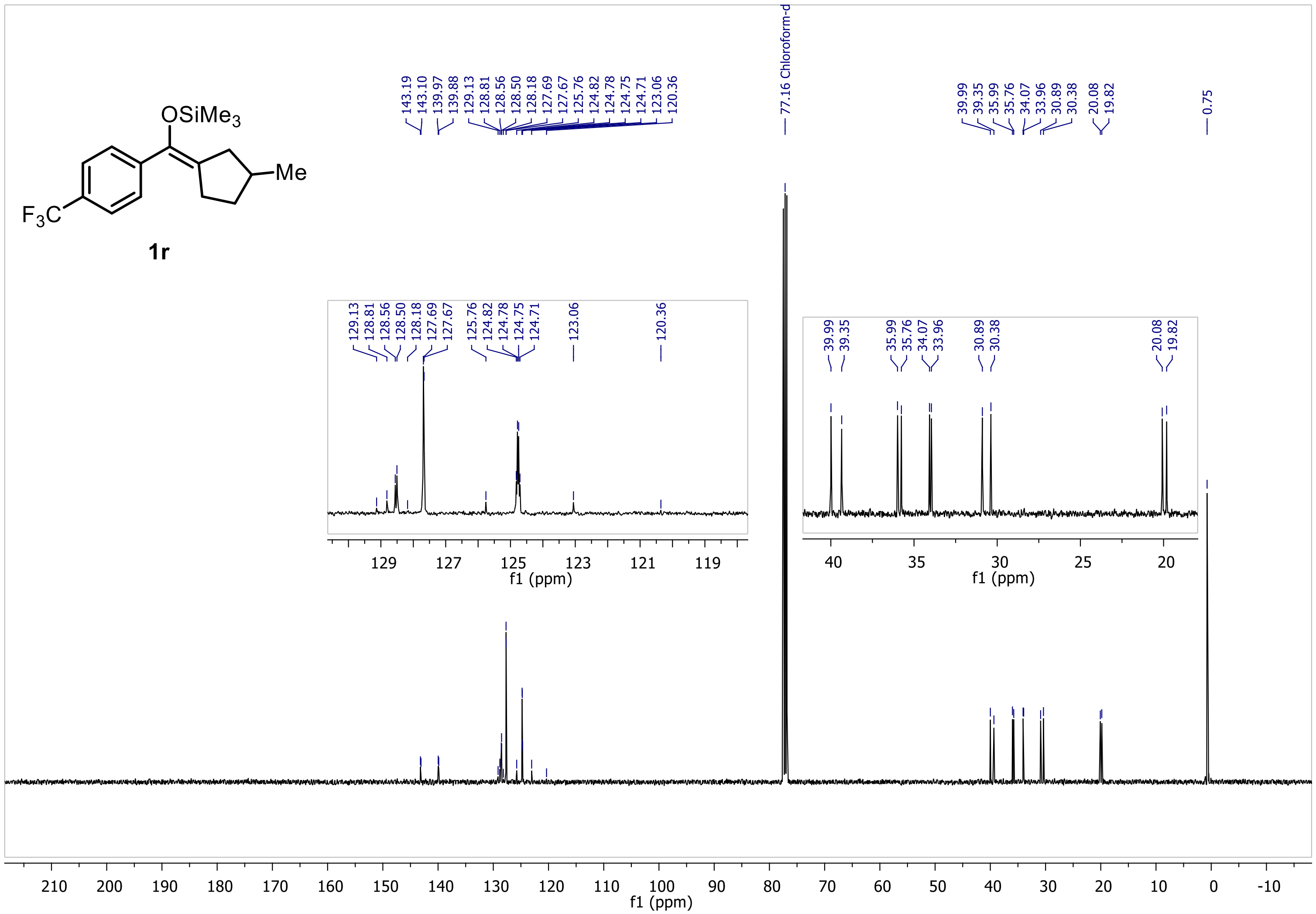

^{19}F NMR (376 MHz, $CDCl_3$)

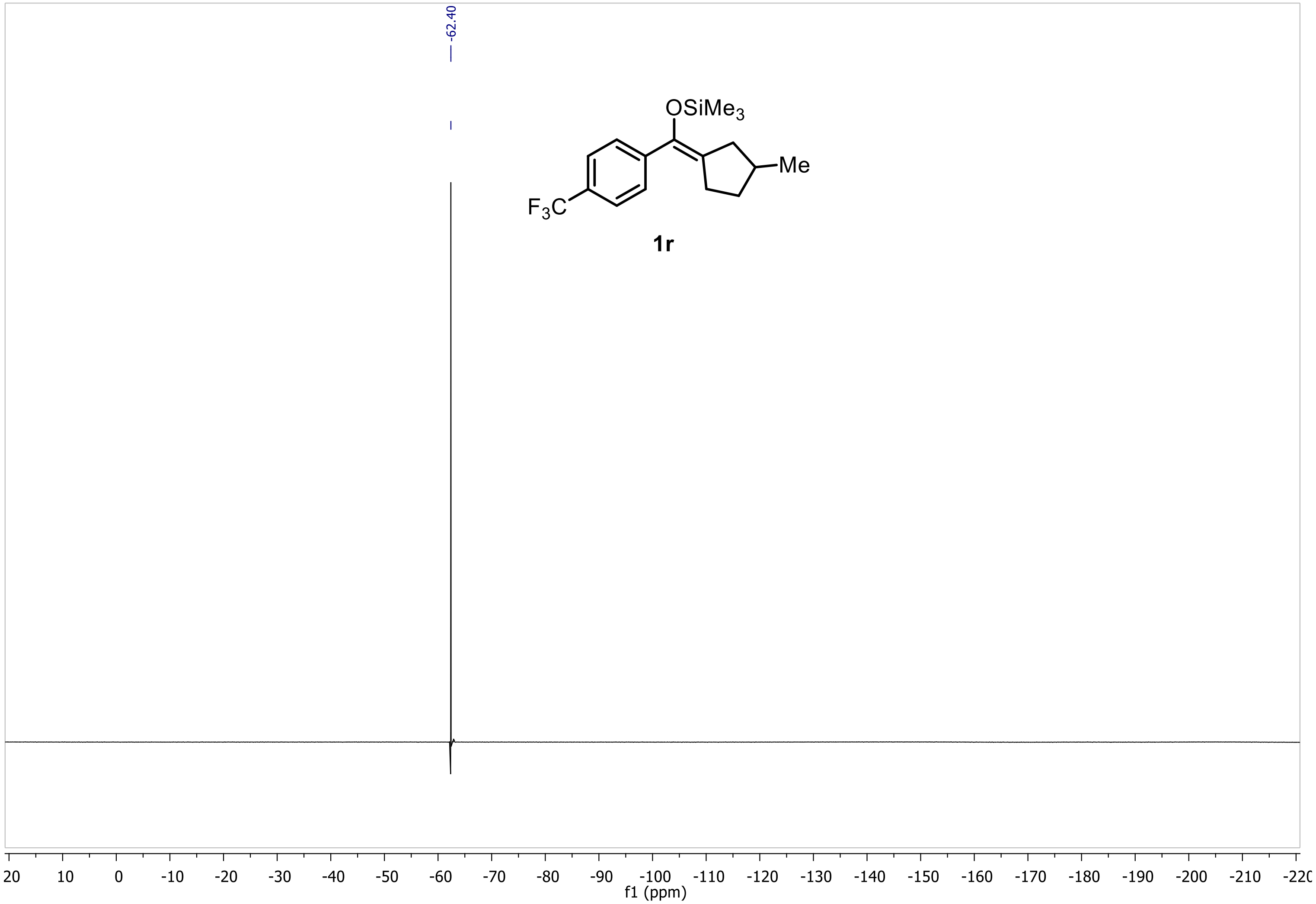

1s – Trimethyl[(3-methylcyclopentylidene)(phenyl)methoxy]silane

^{1}H NMR (400 MHz, $CDCl_3$)

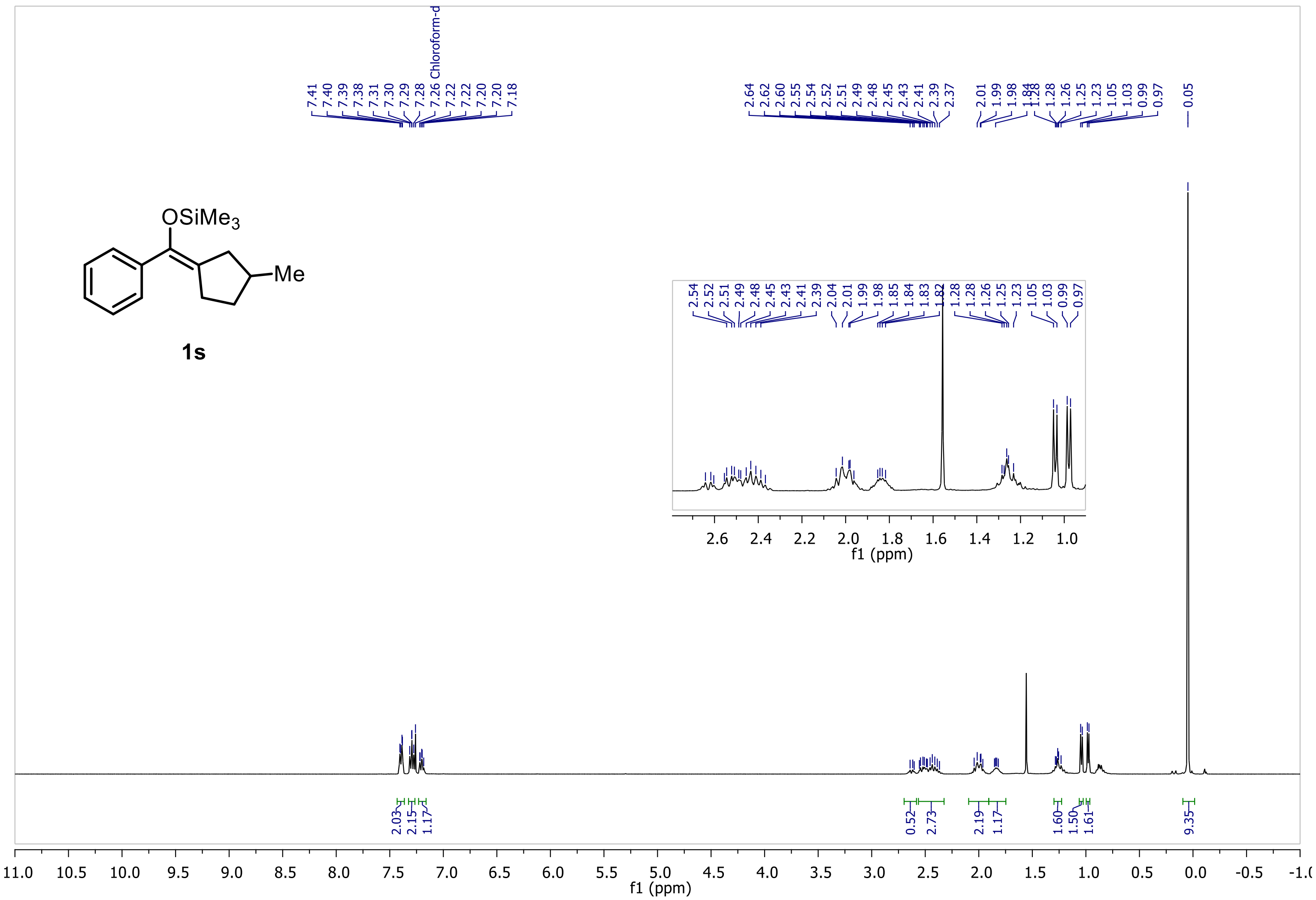

$^{13}C\{^1H\}$ NMR (151 MHz, $CDCl_3$)

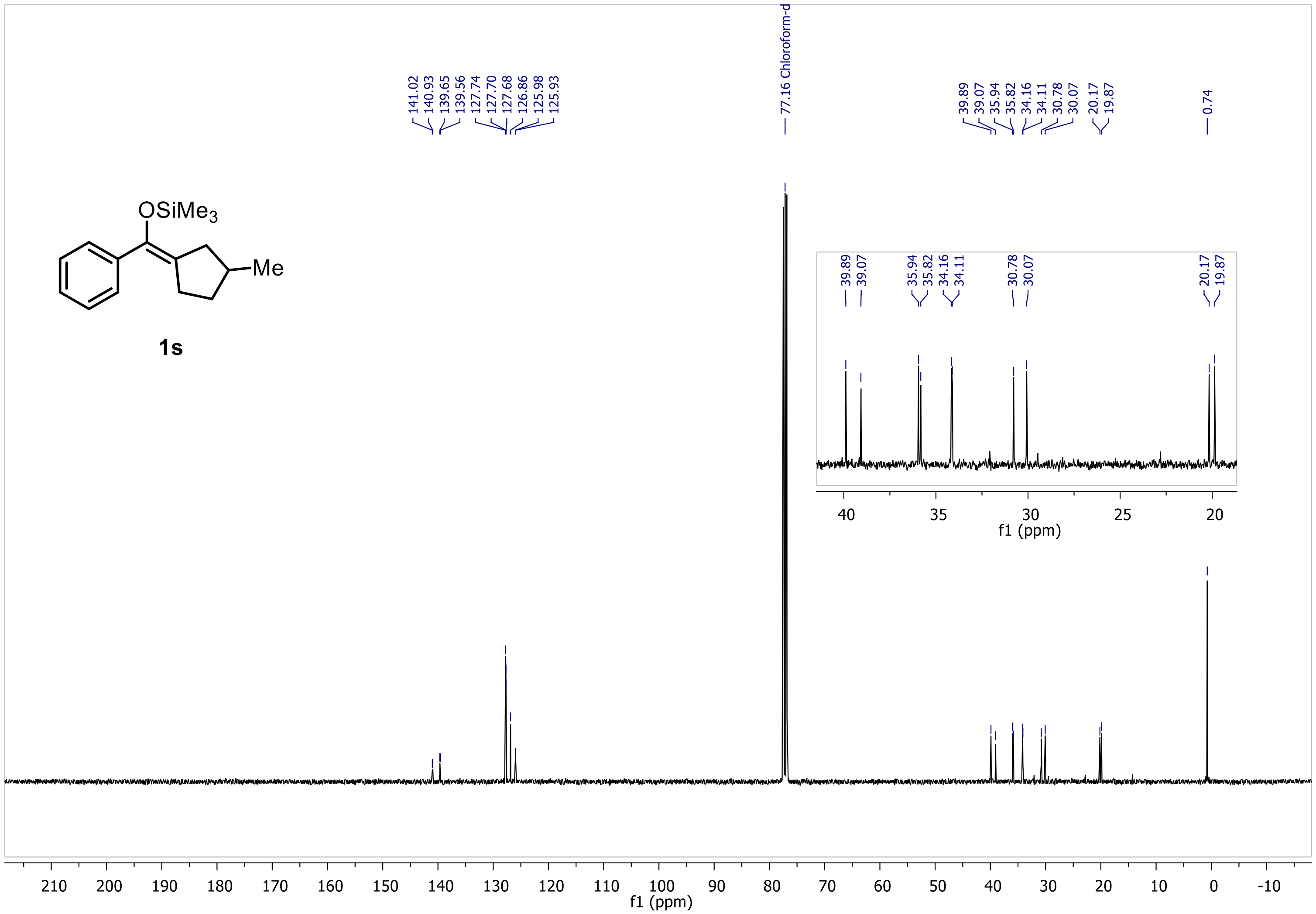

1t – {(4-Butylcyclohexylidene)[4-(trifluoromethyl)phenyl]methoxy}trimethylsilane

^{1}H NMR (400 MHz, CDCl$_3$)

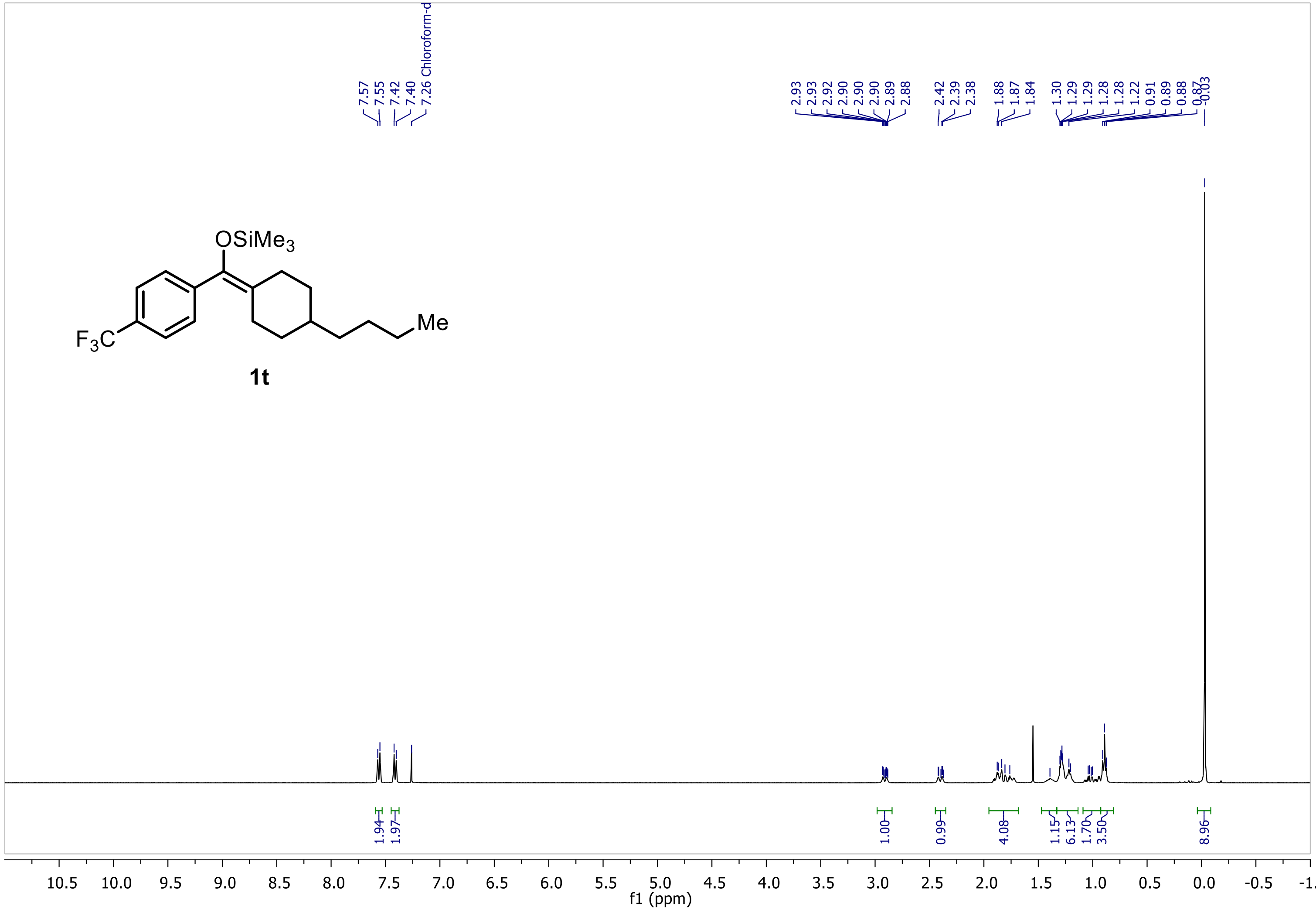

$^{13}C\{^1H\}$ NMR (151 MHz, $CDCl_3$)

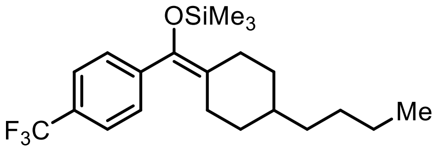

1t

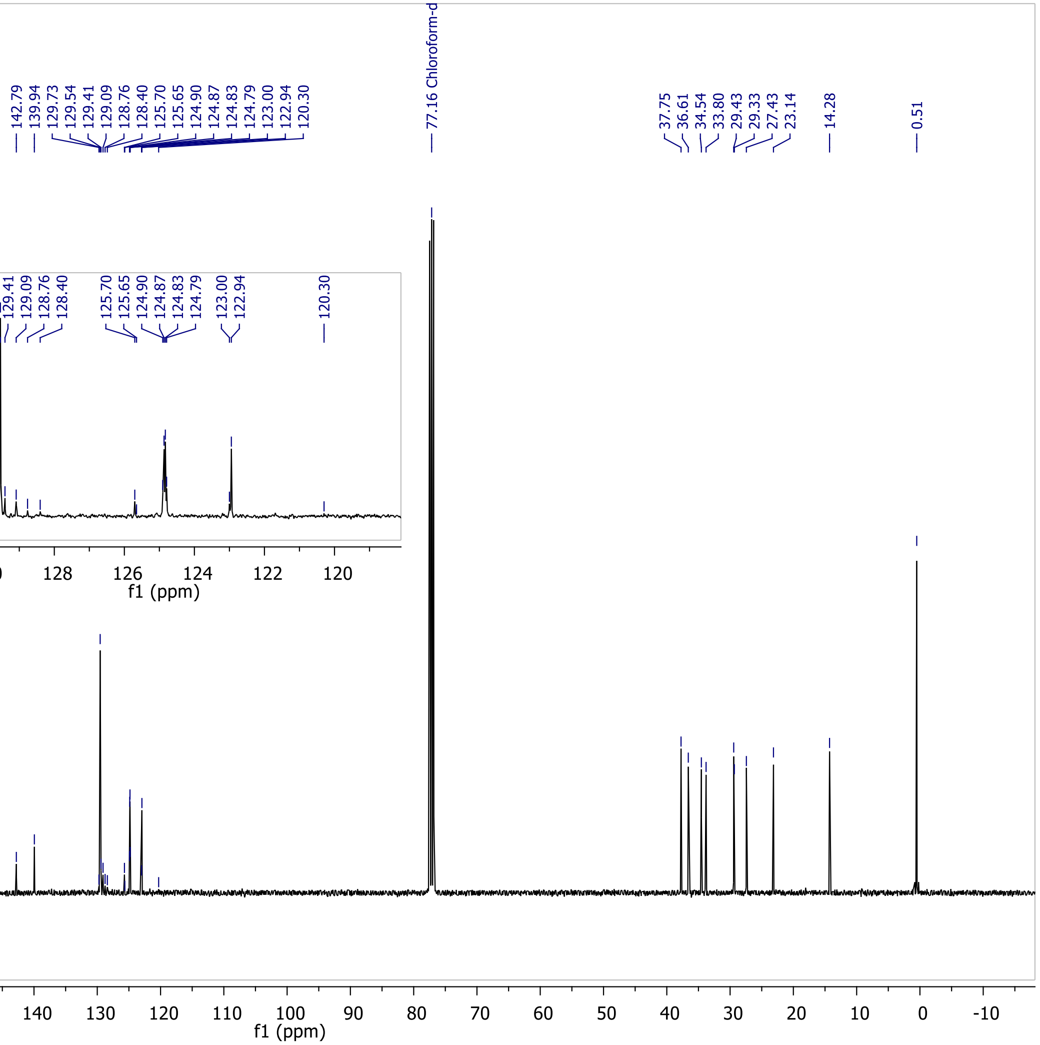

^{19}F NMR (376 MHz, $CDCl_3$)

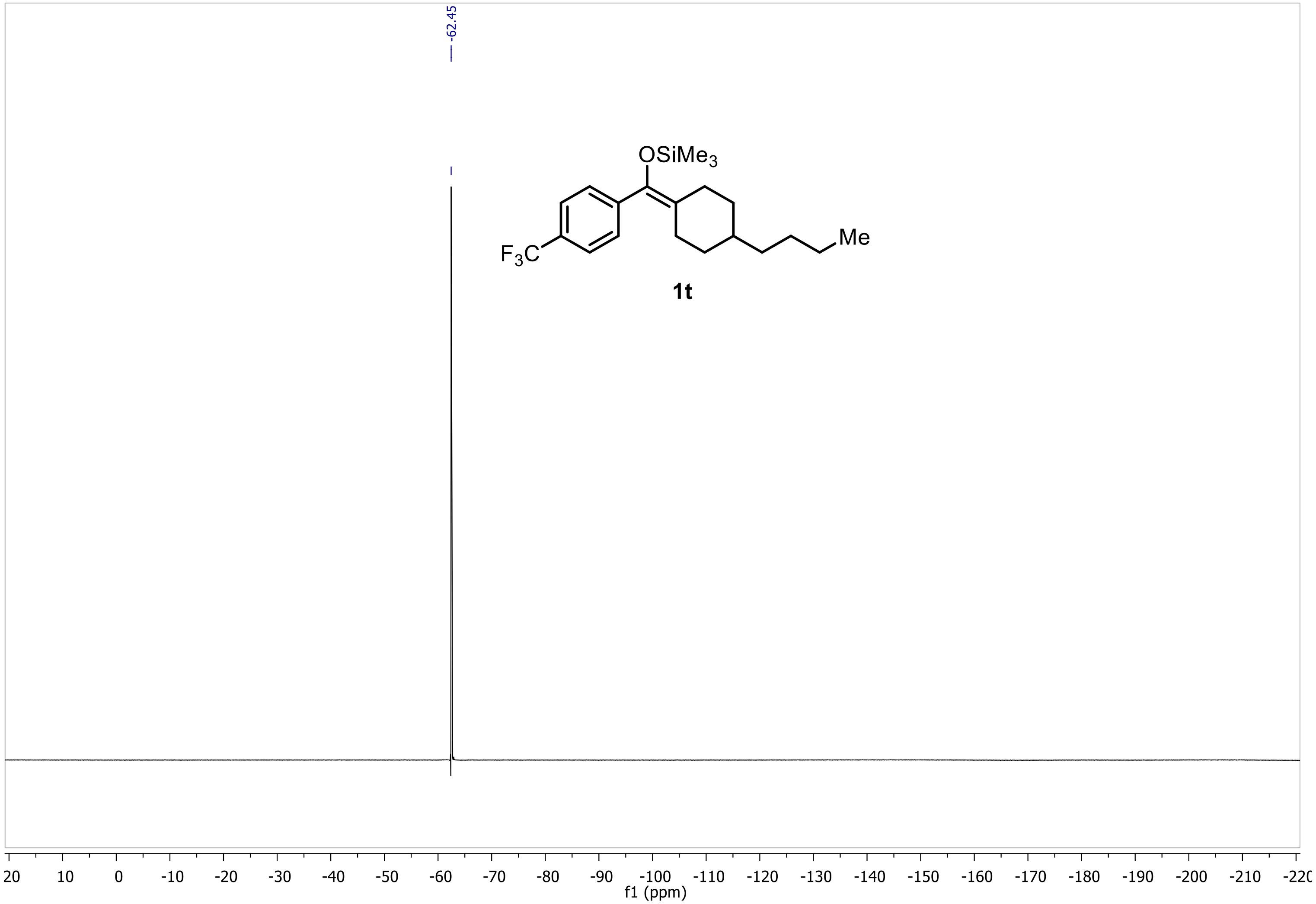

1u – {[4-(*tert*-Butyl)cyclohexylidene][4-(trifluoromethyl)phenyl]methoxy}trimethylsilane

^{1}H NMR (600 MHz, $CDCl_3$)

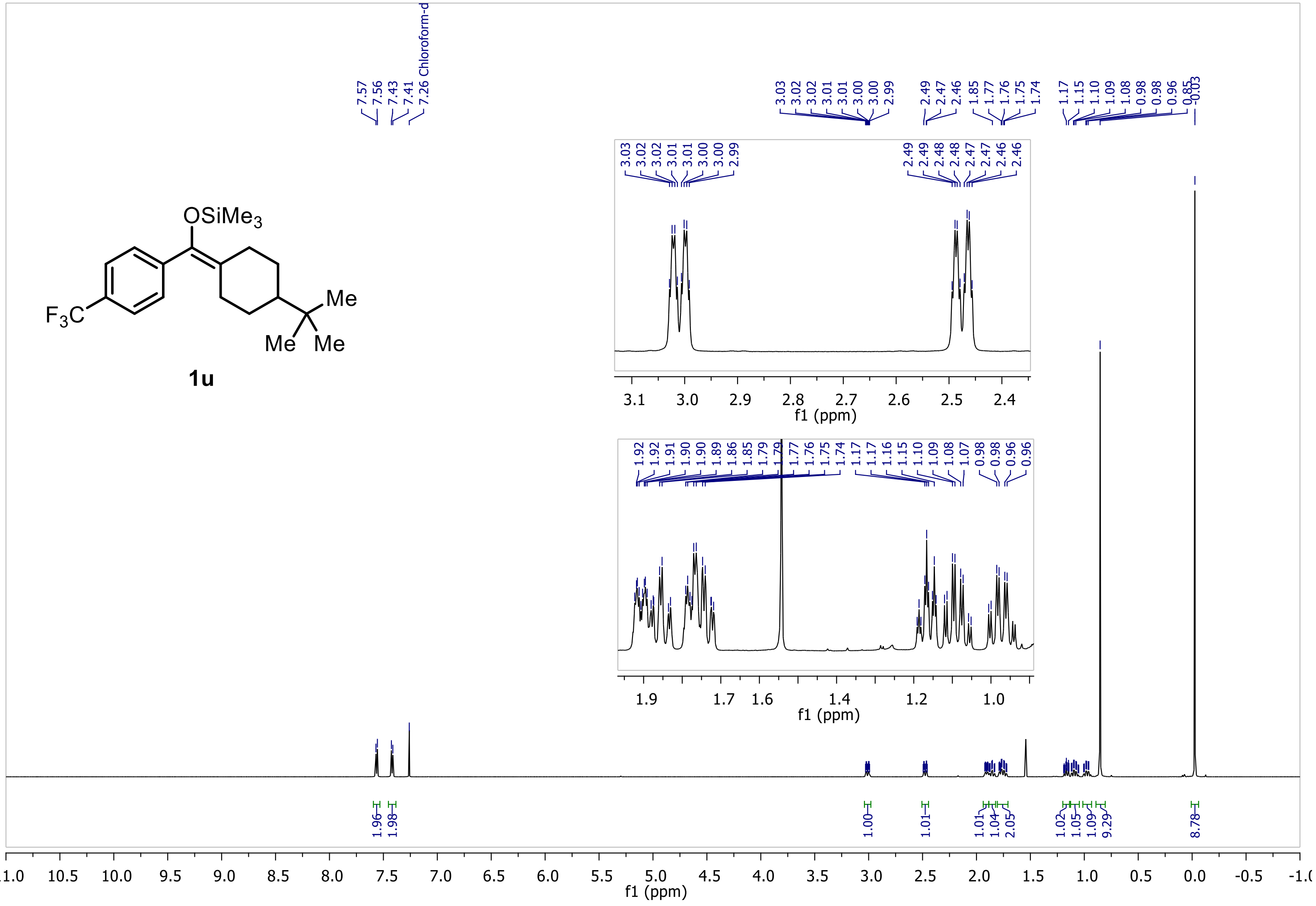

$^{13}C\{^1H\}$ NMR (151 MHz, $CDCl_3$)

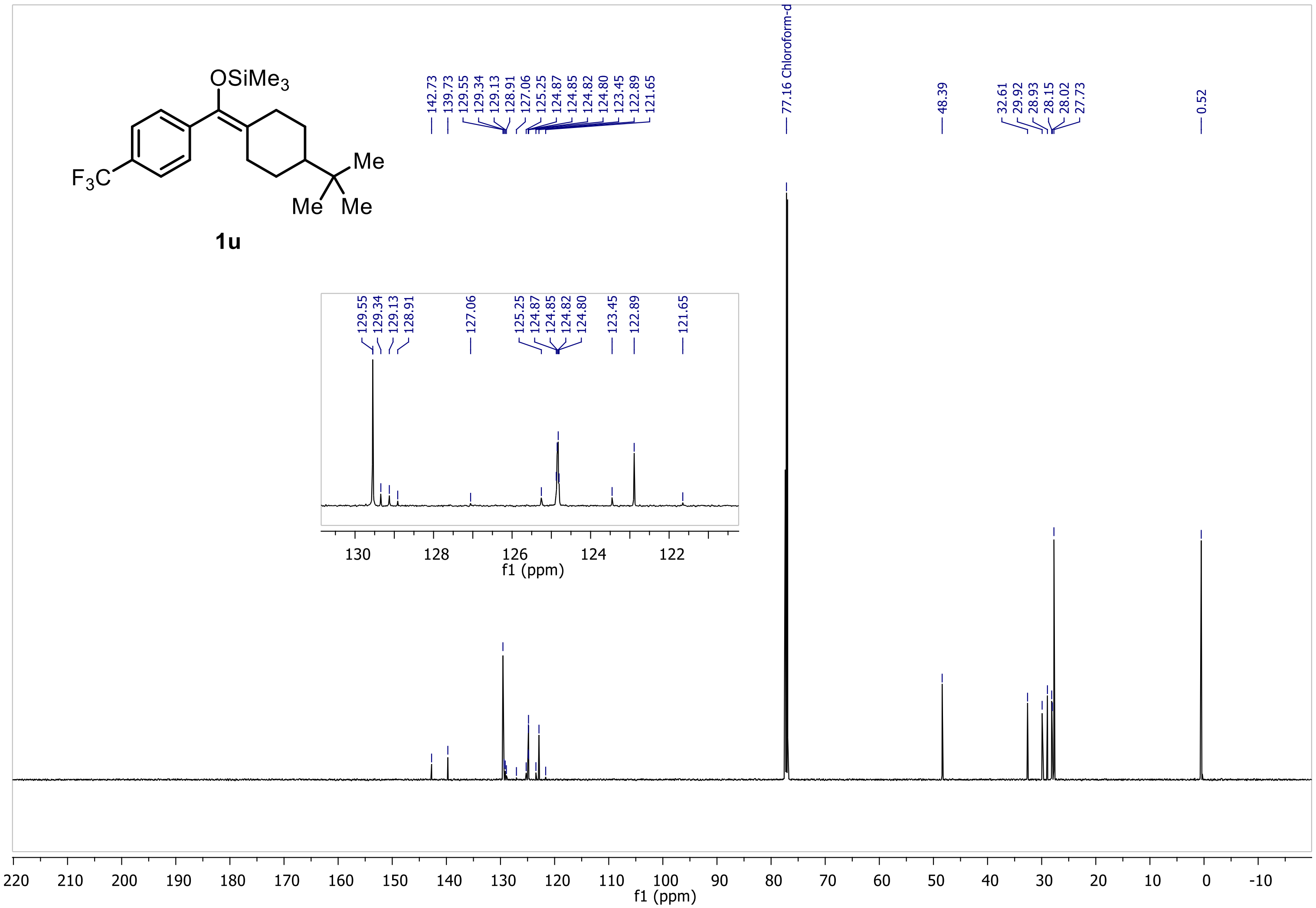

^{19}F NMR (565 MHz, CDCl$_3$)

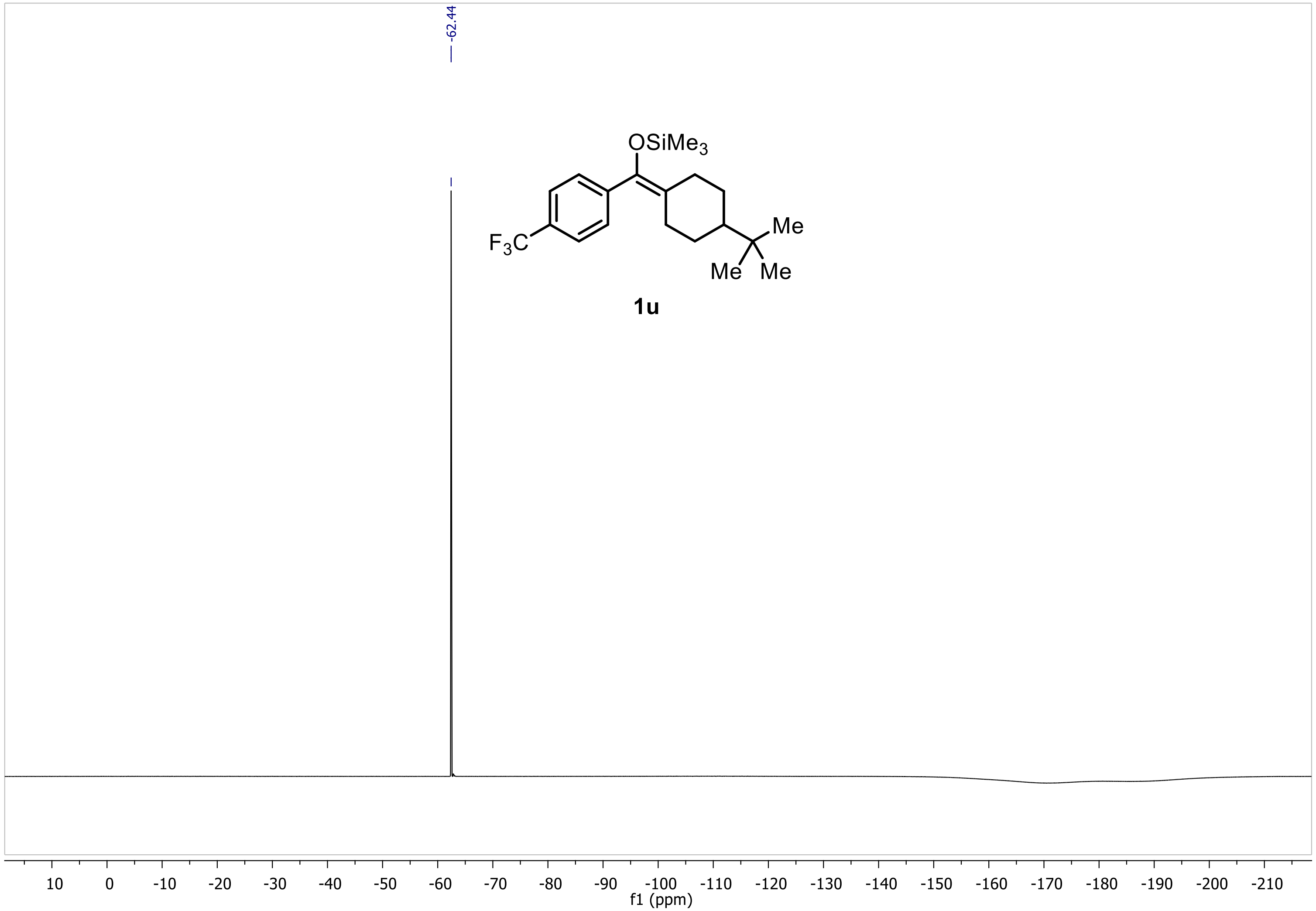

1v – {[4-(*tert*-Butyl)cyclohexylidene][3-(trifluoromethyl)phenyl]methoxy}trimethylsilane

^{1}H NMR (400 MHz, $CDCl_3$)

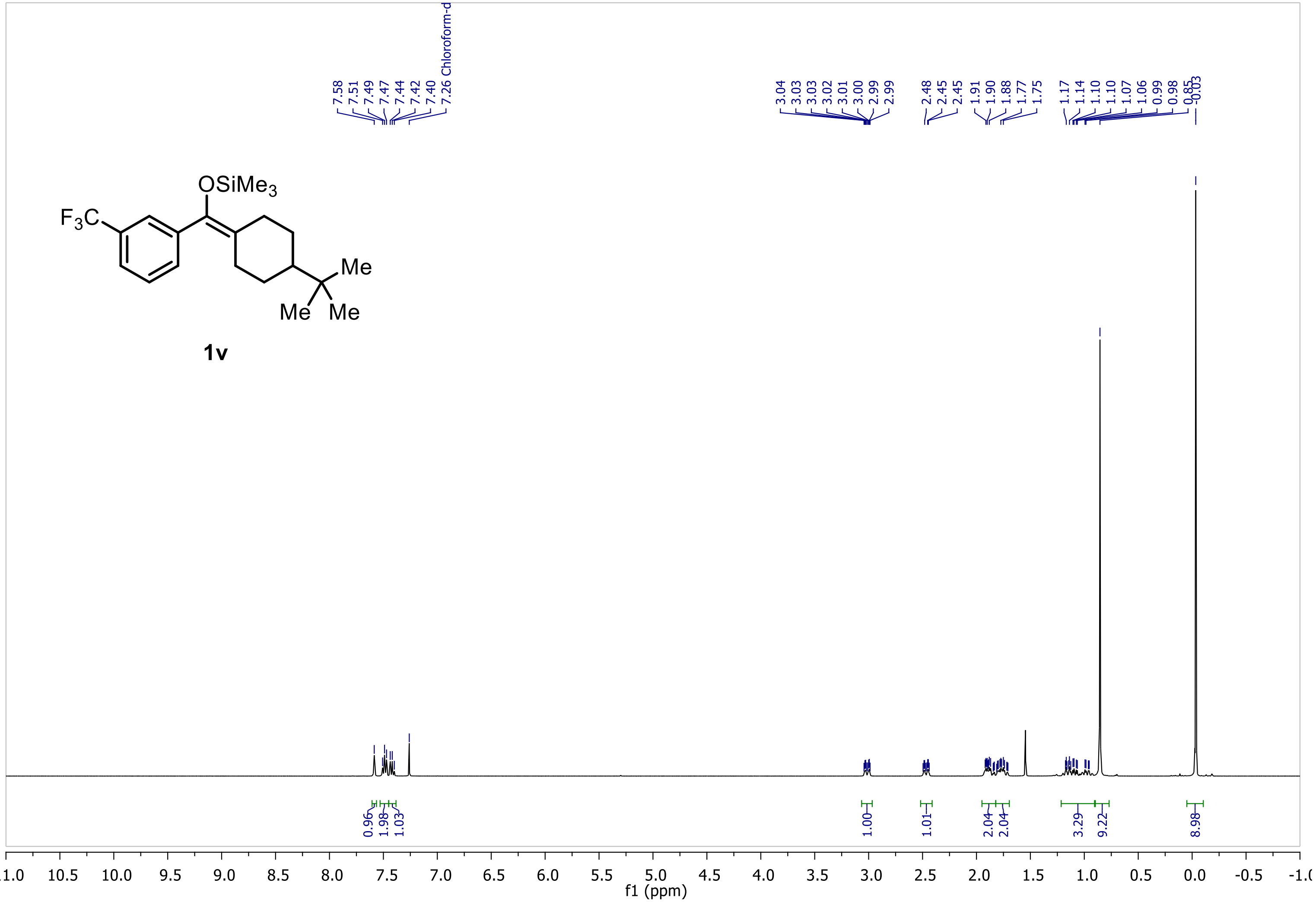

$^{13}C\{^1H\}$ NMR (101 MHz, $CDCl_3$)

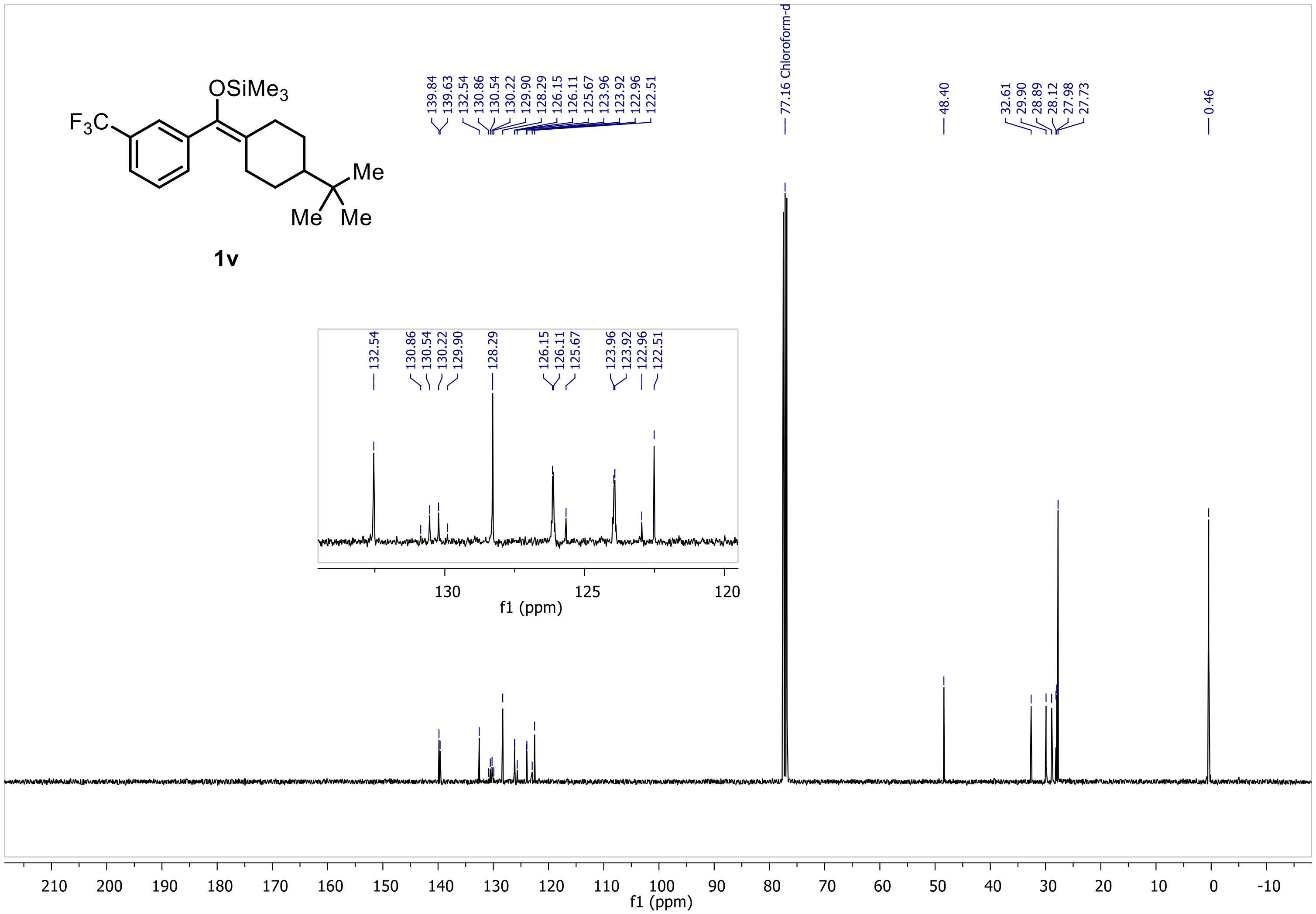

^{19}F NMR (376 MHz, $CDCl_3$)

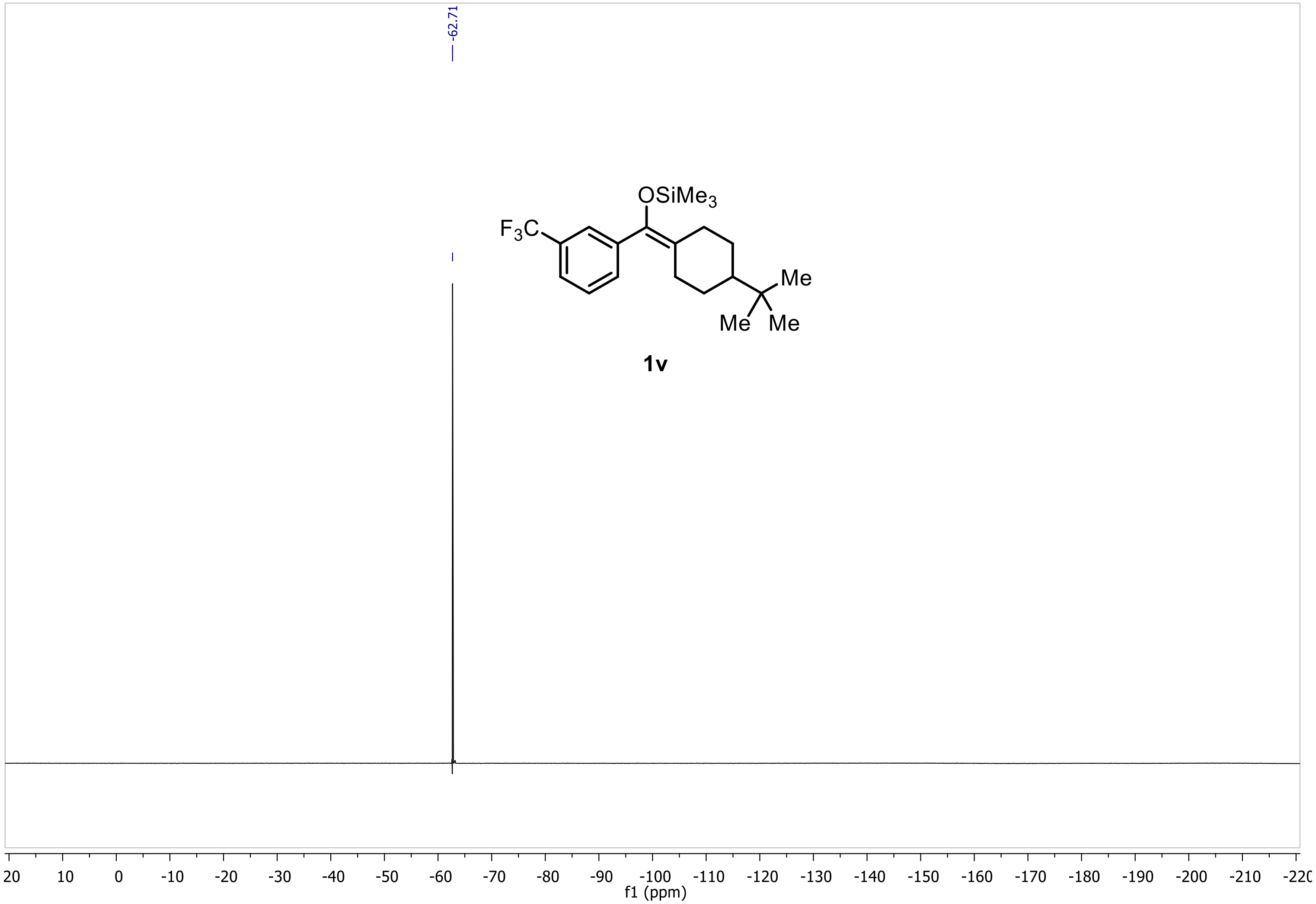

1w – Trimethyl{(4-methylcyclohexylidene)[4-(trifluoromethyl)phenyl]methoxy}silane

^{1}H NMR (400 MHz, $CDCl_3$)

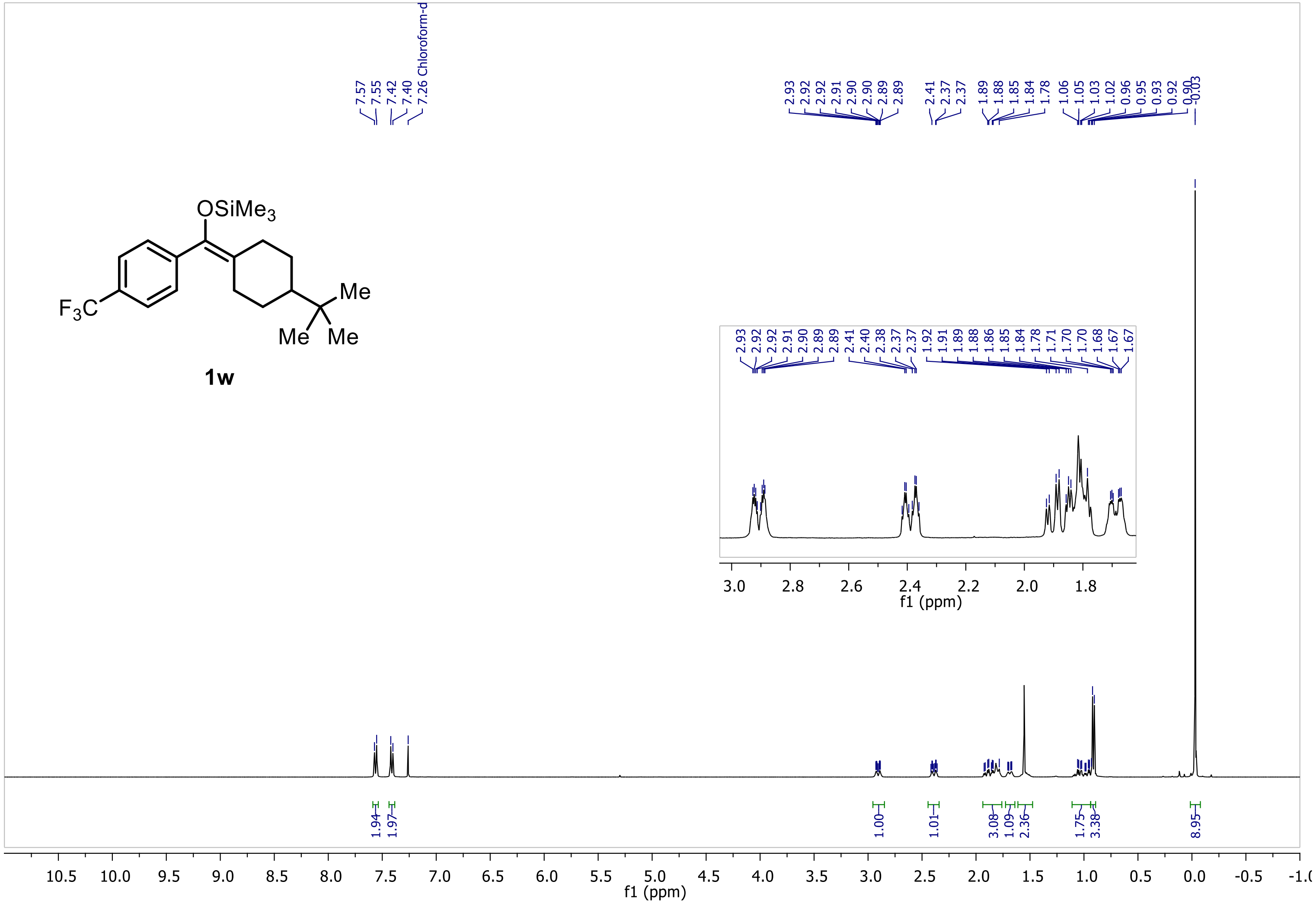

$^{13}C\{^{1}H\}$ NMR (151 MHz, $CDCl_3$)

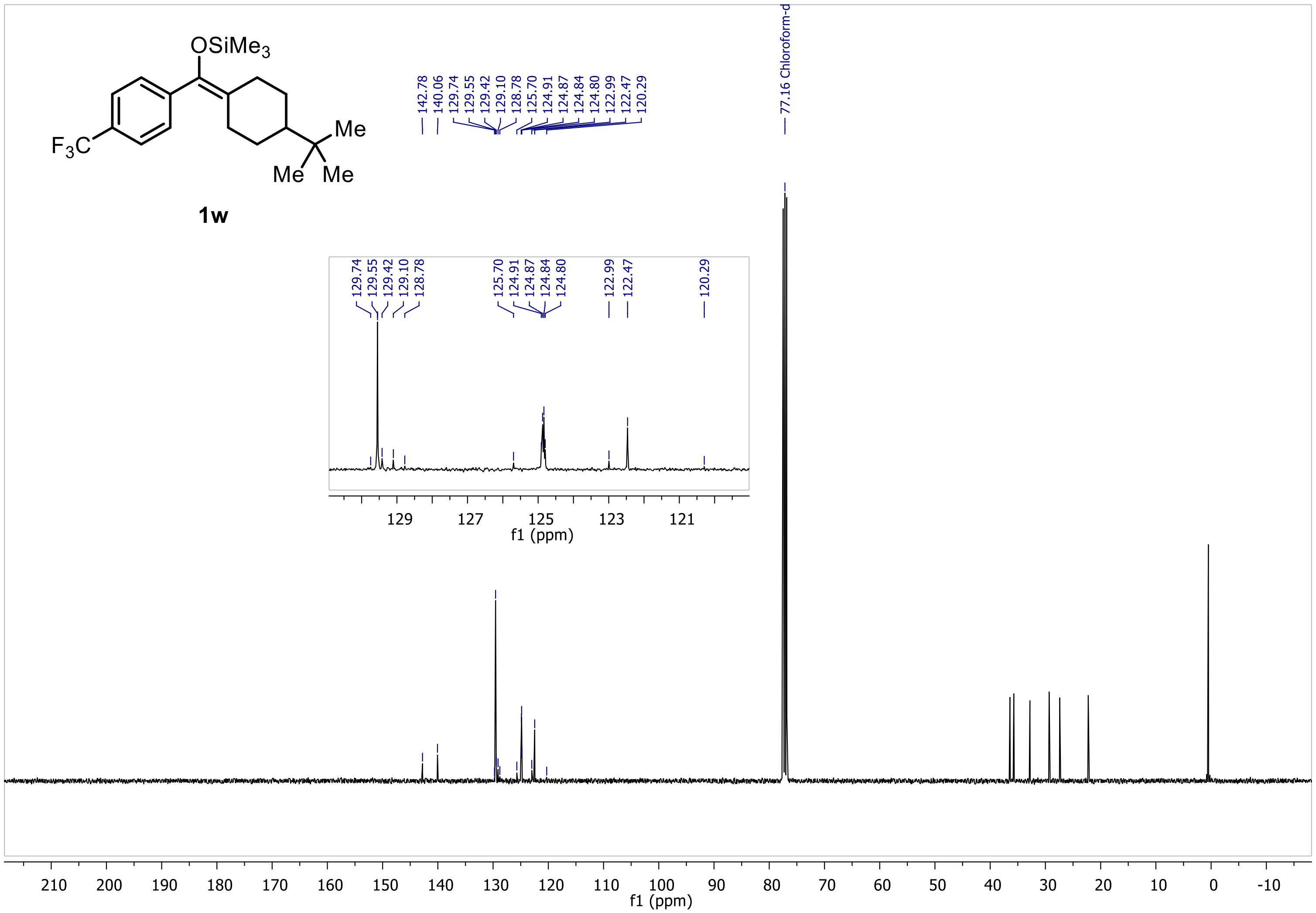

^{19}F NMR (376 MHz, CDCl$_3$)

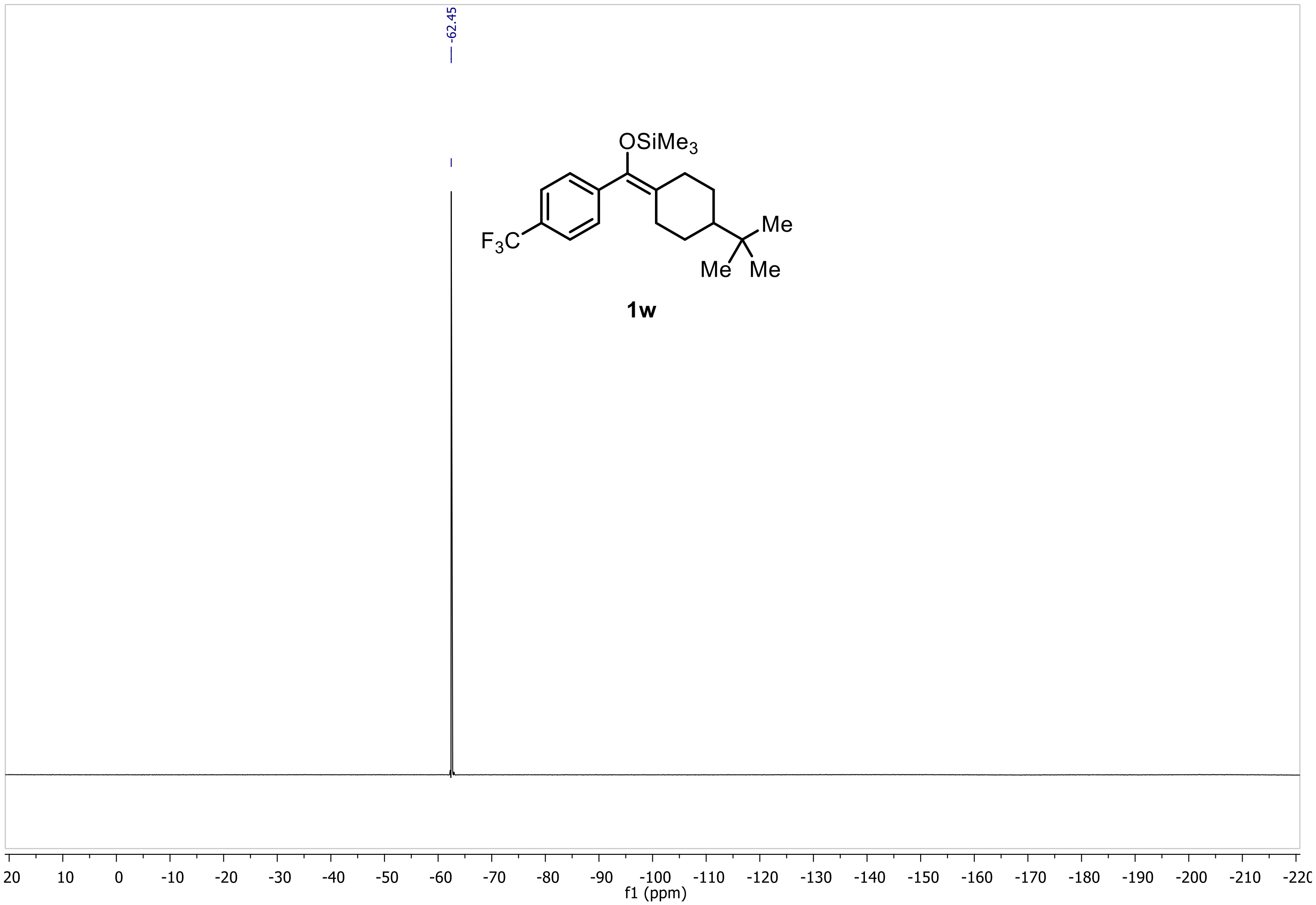

9a – $(PhI)_2O(SbF_6)_2$ – Oxybis(phenyliodonium) bis[hexafluorostilbate(V)]

^{1}H NMR (400 MHz, 2:8 DMSO-d_6/$CDCl_3$)

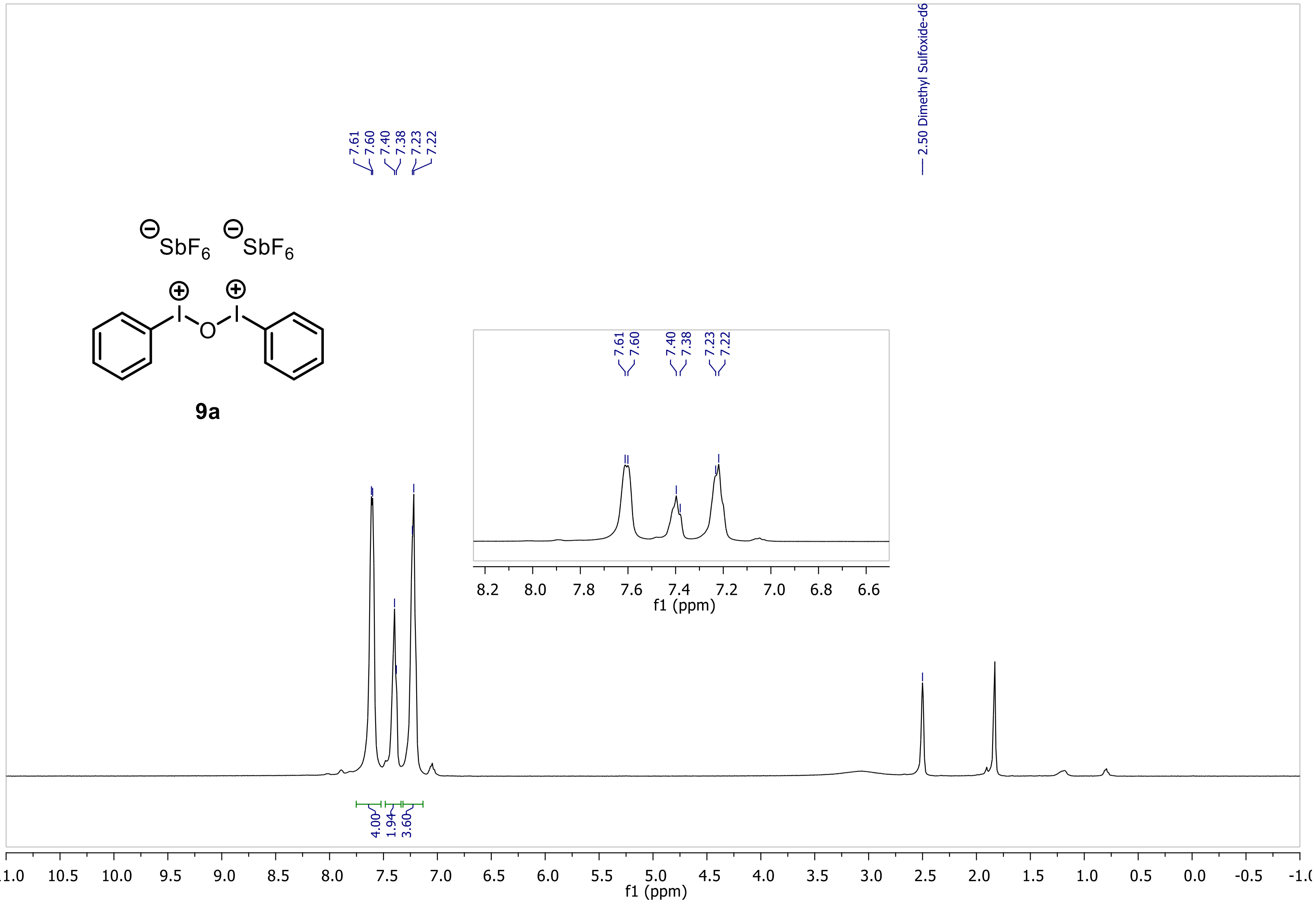

^{19}F NMR (376 MHz, $CDCl_3$)

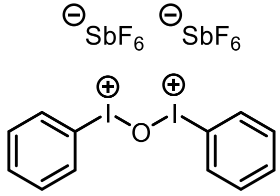

9a

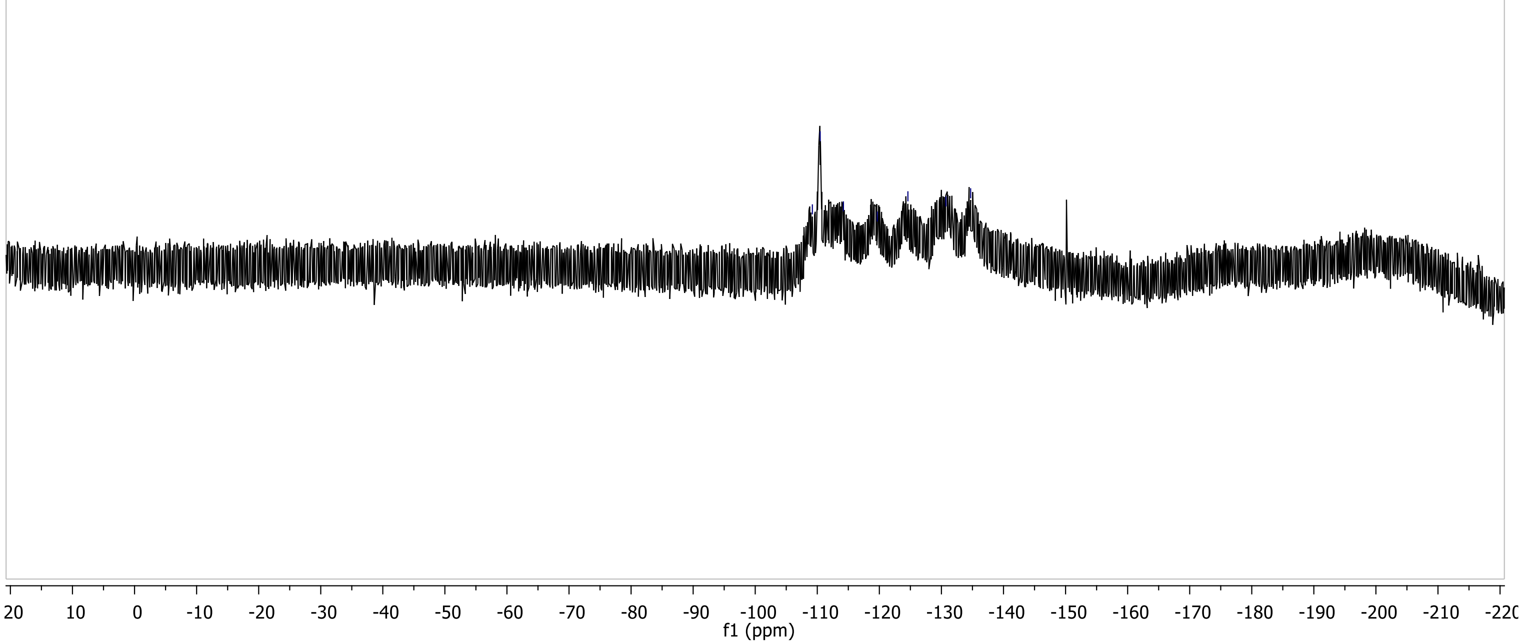

9b – $(PhI)_2O(BF_4)_2$ – Oxybis(phenyliodonium) bis[tetrafluoroborate(V)]

^{1}H NMR (400 MHz, 2:8 DMSO-d_6/$CDCl_3$)

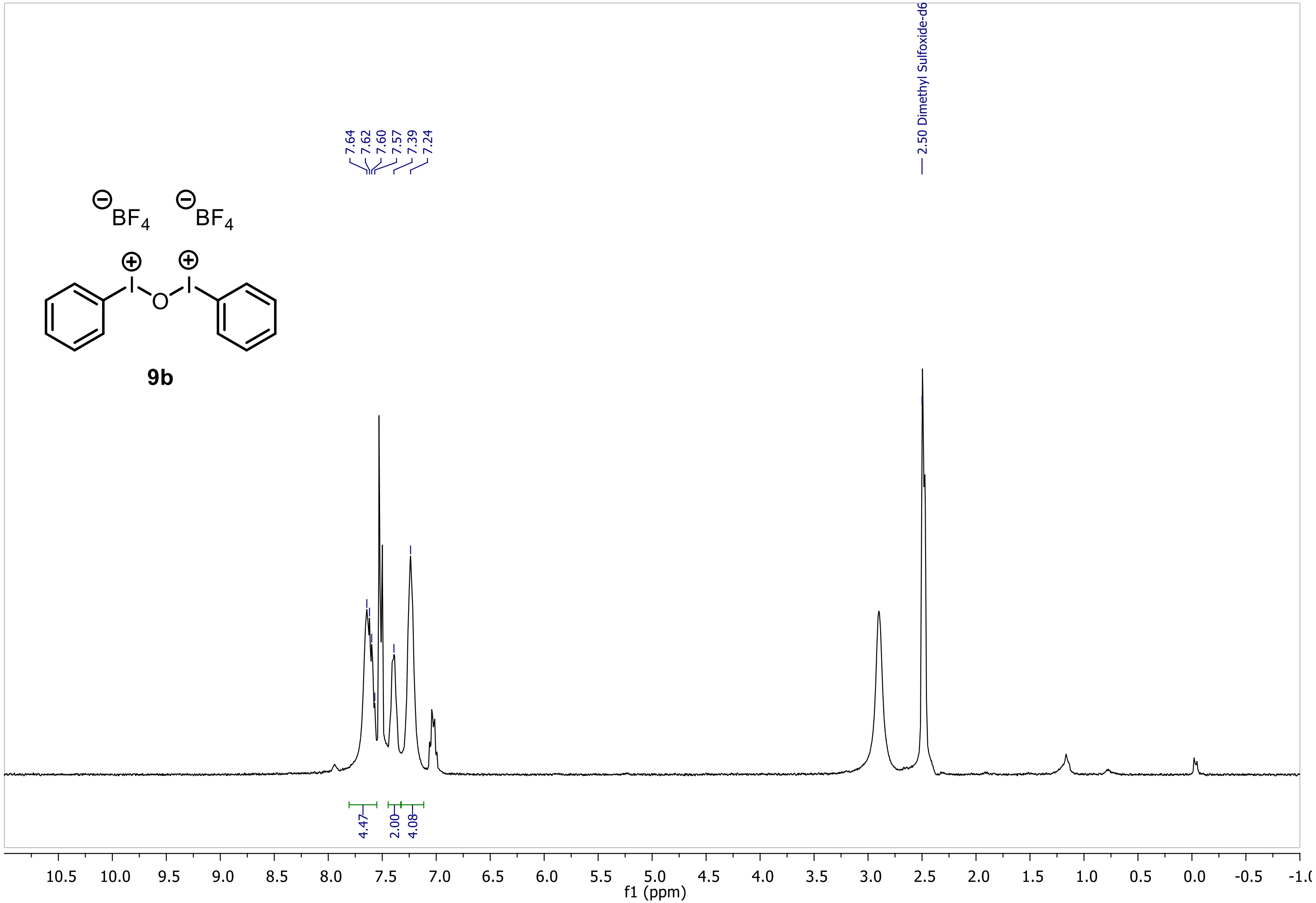

^{19}F NMR (376 MHz, $CDCl_3$)

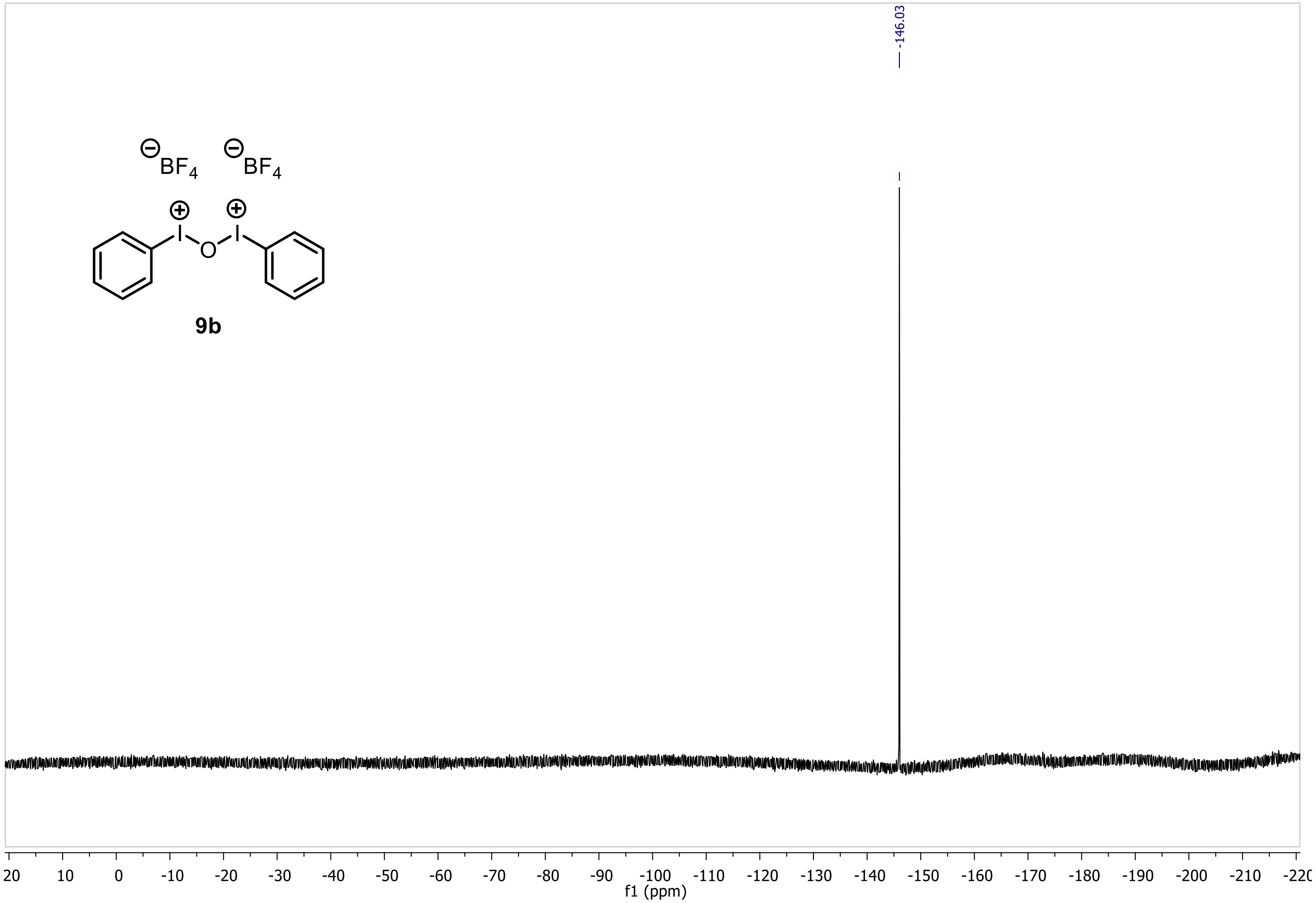

9c – $(PhI)_2O(ClO_4)_2$ – Oxybis(phenyliodonium) bis(perchlorate)

^{1}H NMR (400 MHz, 2:8 DMSO-d_6/$CDCl_3$)

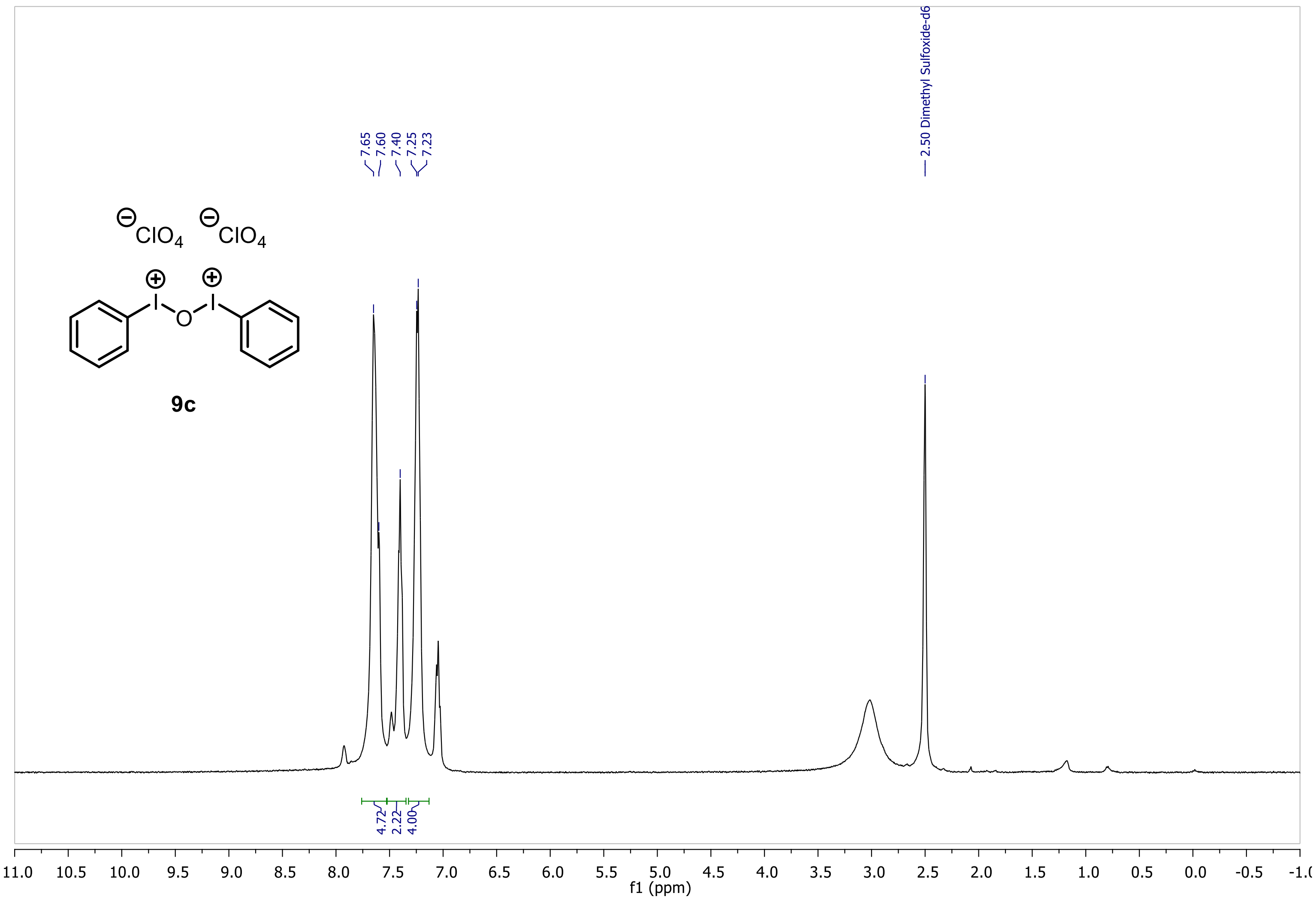

9d – (*para*-*t*-$BuC_6H_4I)_2O(SbF_6)_2$

^{1}H NMR (400 MHz, 2:8 DMSO-d_6/$CDCl_3$)

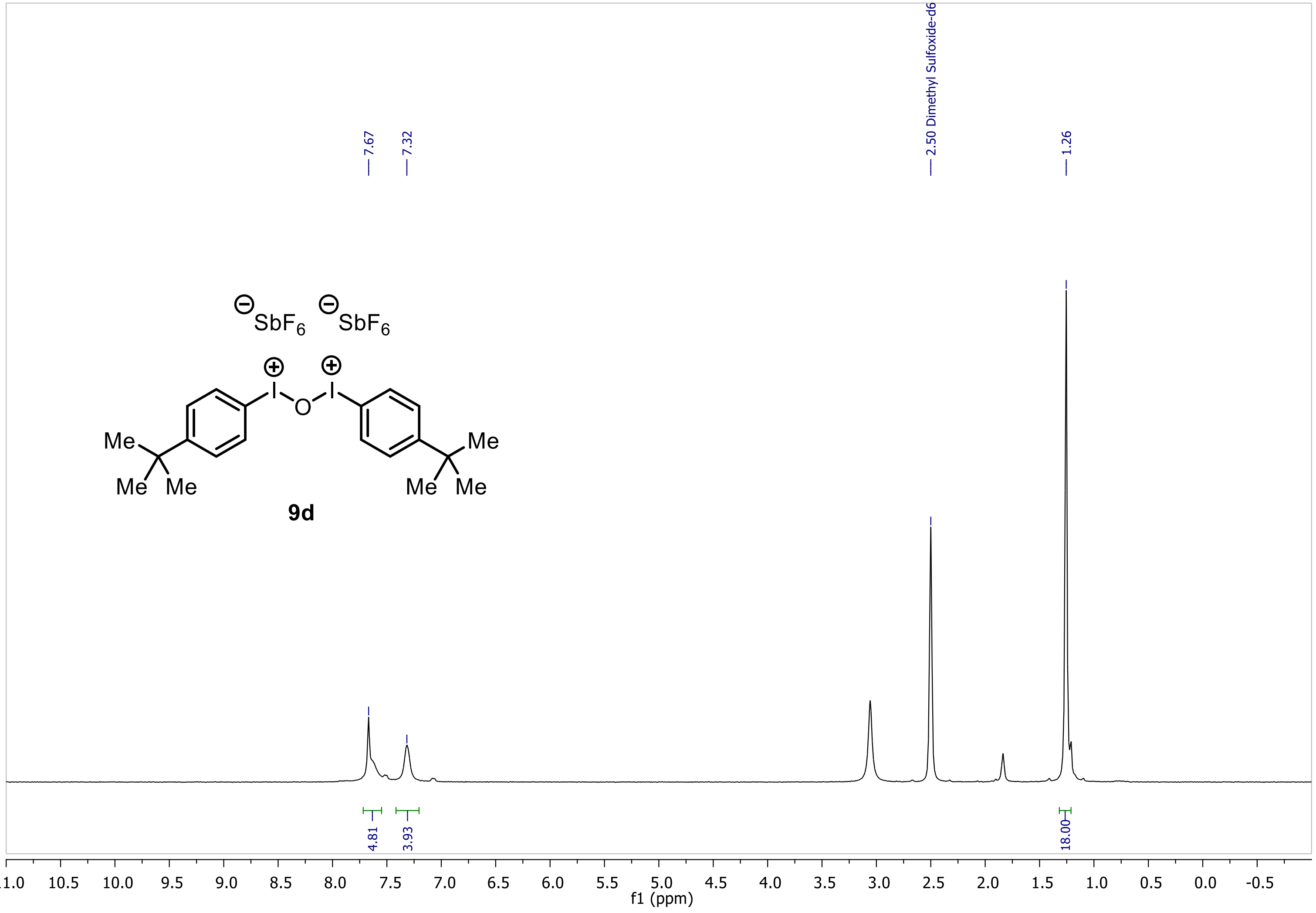

^{19}F NMR (565 MHz, 2:8 DMSO-d_6/$CDCl_3$)

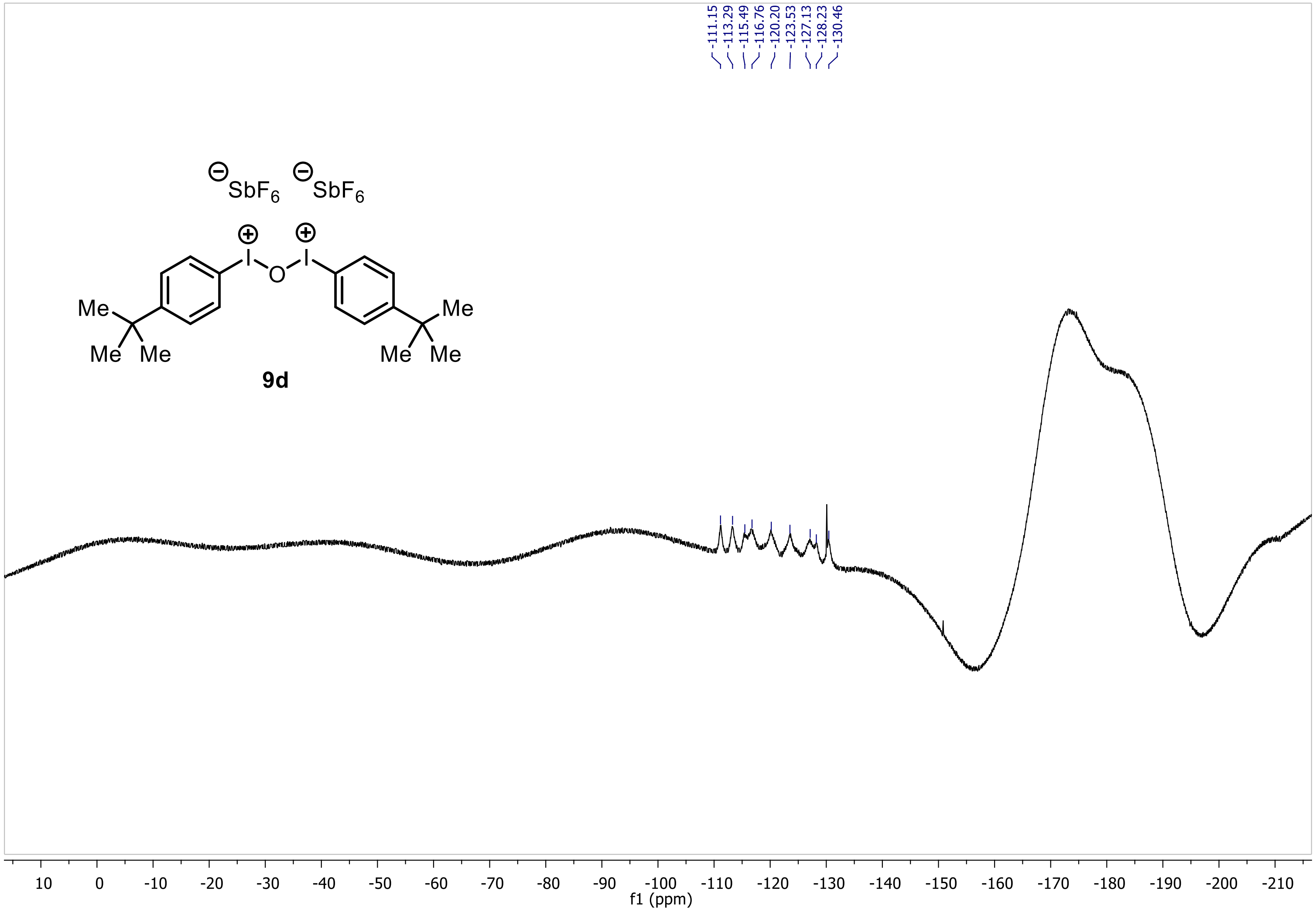

2a – *cis*-3-Hydroxy-3-methylcyclohexyl][4-(trifluoromethyl)phenyl]methanone

^{1}H NMR (700 MHz, $CDCl_3$)

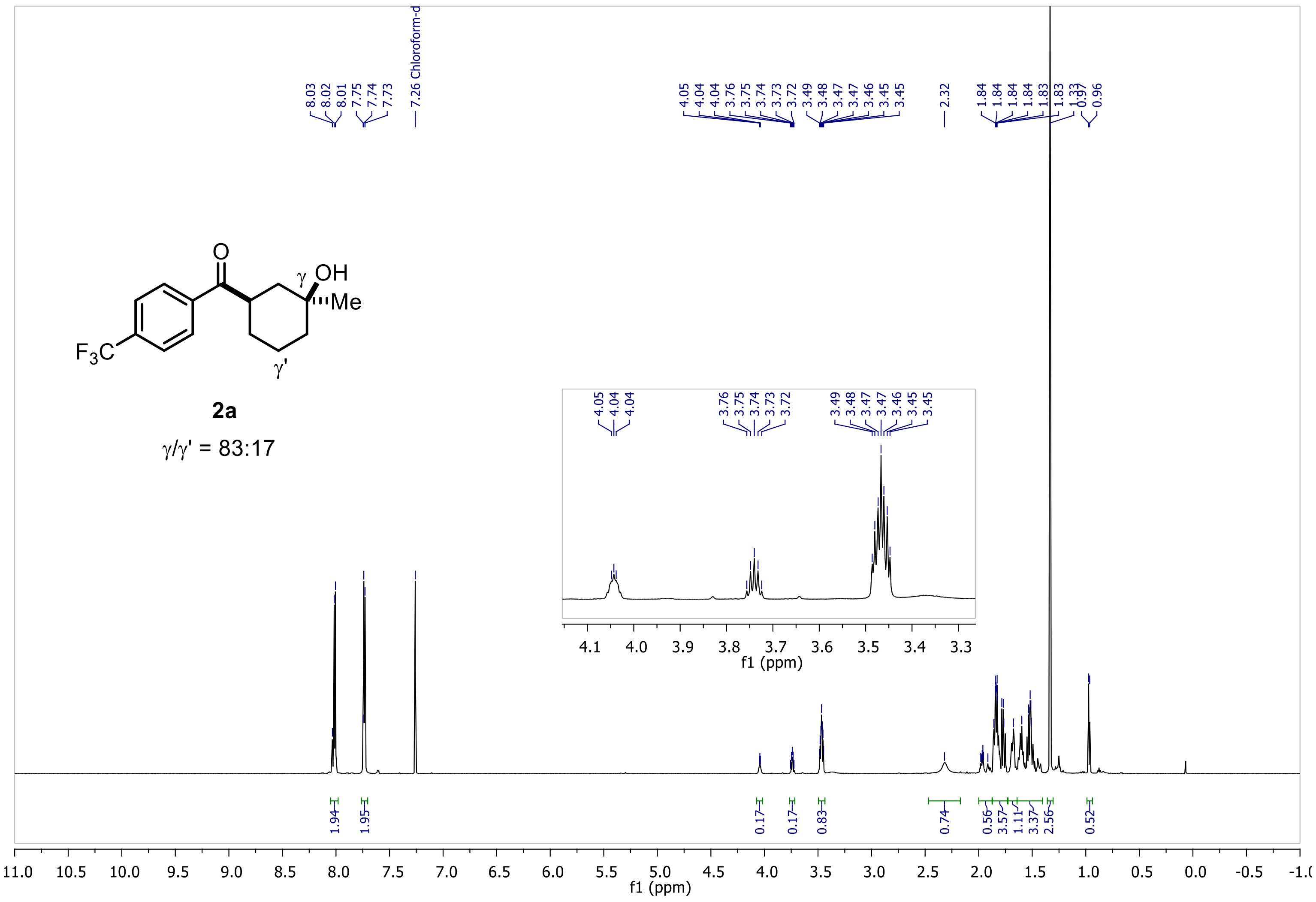

^{13}C (DEPT 135) NMR (176 MHz, $CDCl_3$)

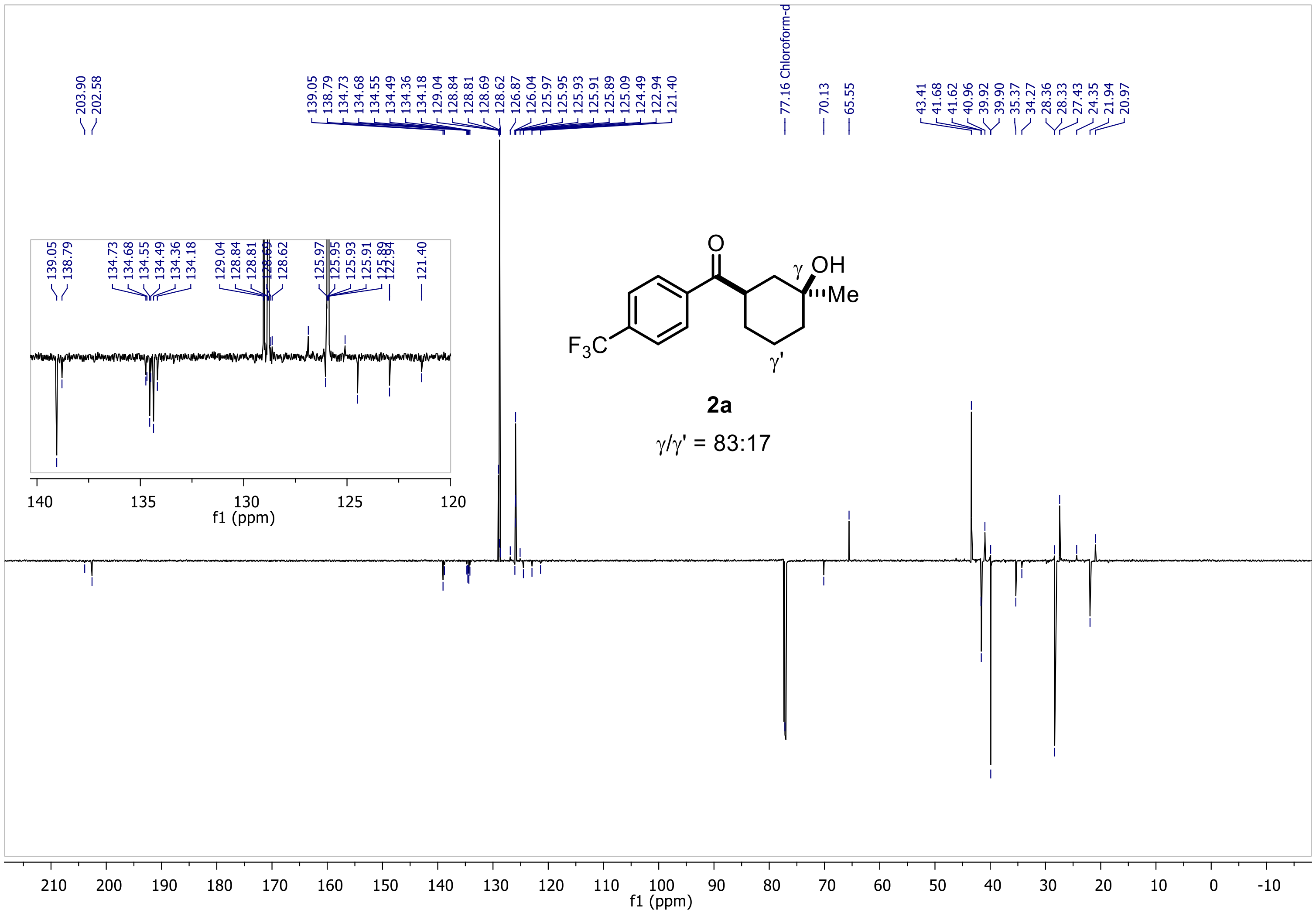

¹⁹F NMR (659 MHz, CDCl₃)

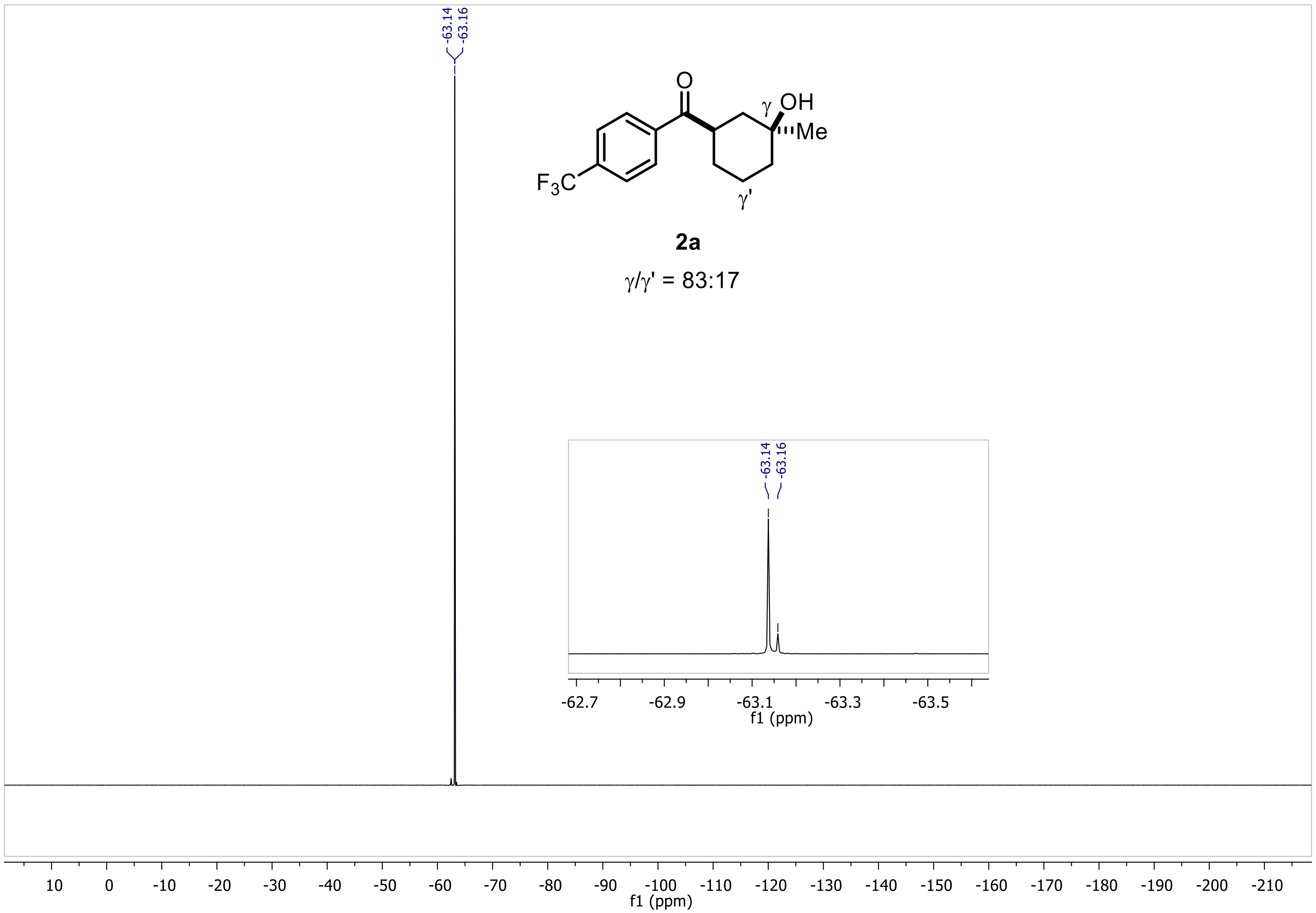

2b – ((1*R*,3*R*,8*S*,9*S*,10*S*,13*R*,14*S*,17*R*)-10-hydroxy-1,13-dimethyl-17-((*R*)-6-methylheptan-2-yl)hexadecahydro-1*H*-cyclopenta[a]phenanthren-3-yl)(4-(trifluoromethyl)phenyl)methanone

^{1}H NMR (700 MHz, $CDCl_3$)

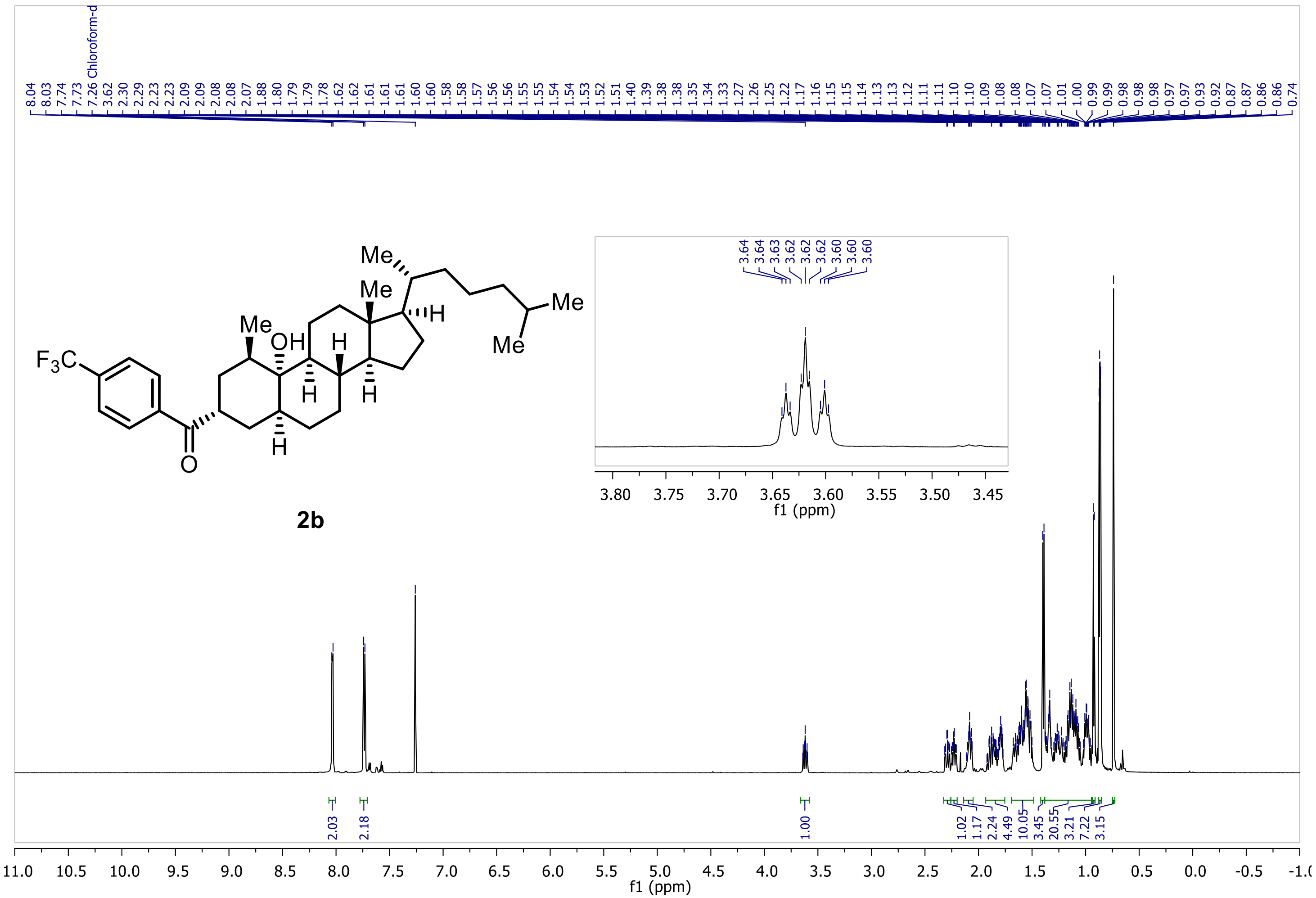

$^{13}C\{^1H\}$ NMR (176 MHz, CDCl$_3$)

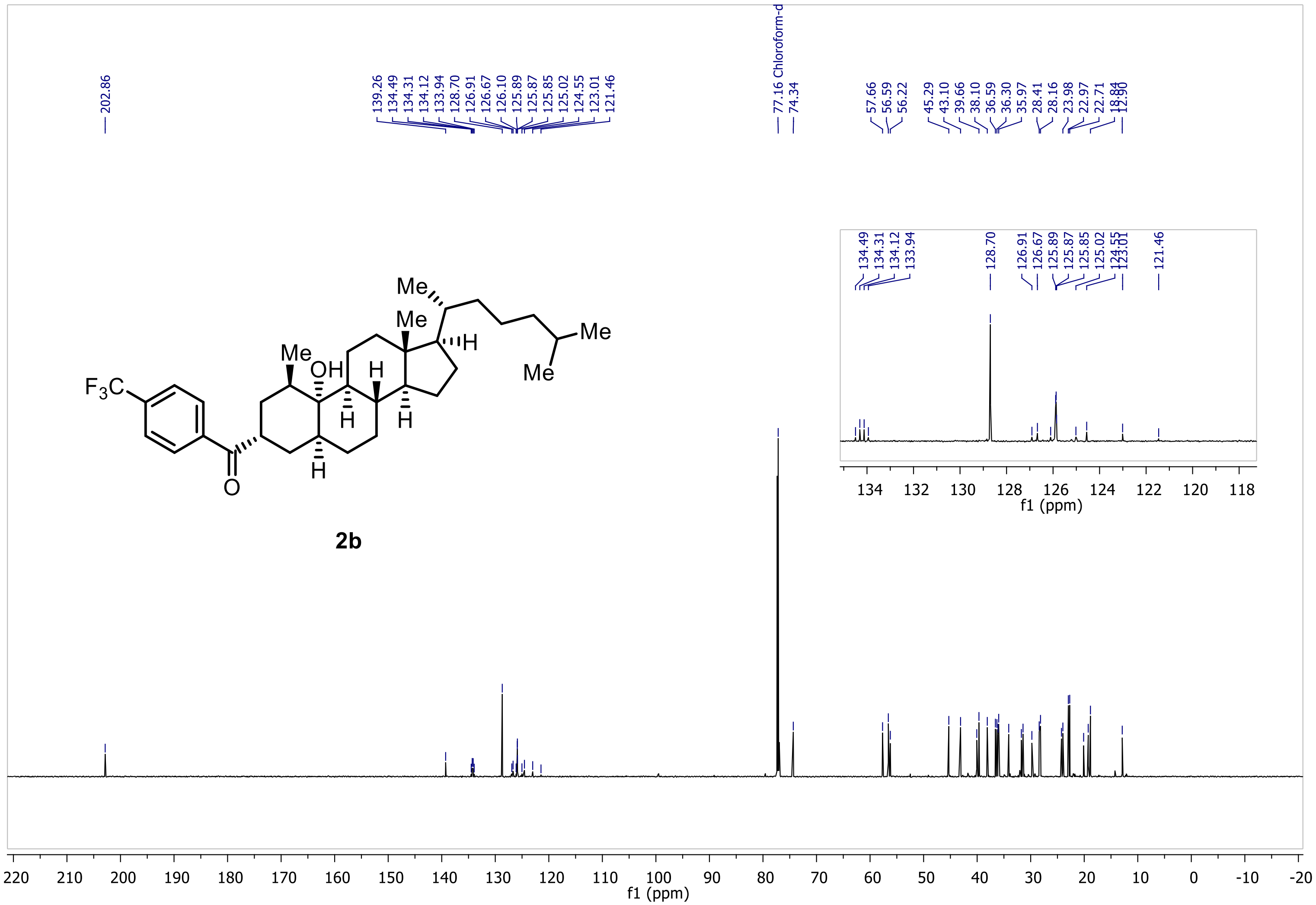

^{19}F NMR (659 MHz, $CDCl_3$)

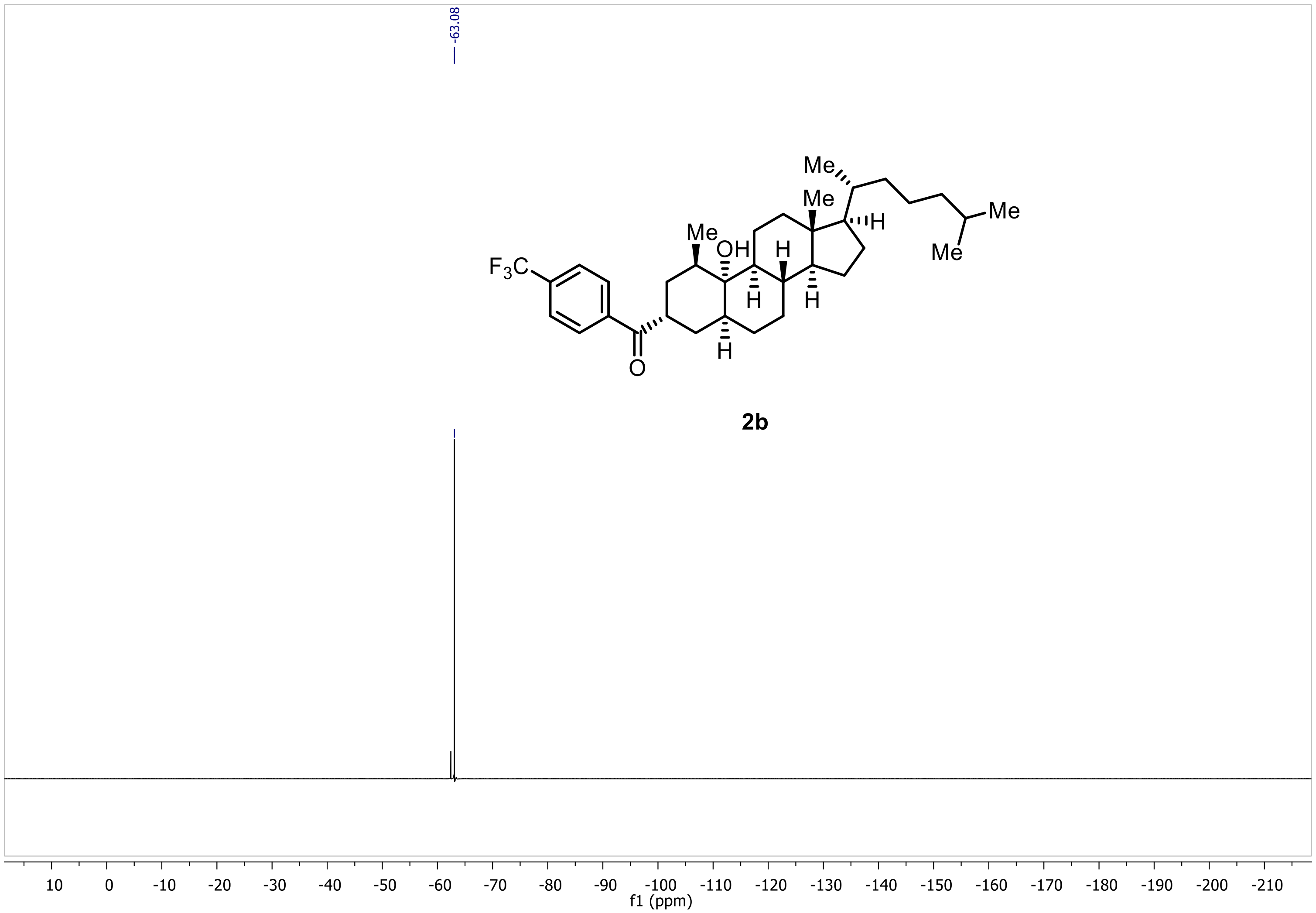

2b

2c – *cis*-(3-Hydroxy-3,5,5-trimethylcyclohexyl)[4-(trifluoromethyl)phenyl]methanone

^{1}H NMR (600 MHz, $CDCl_3$)

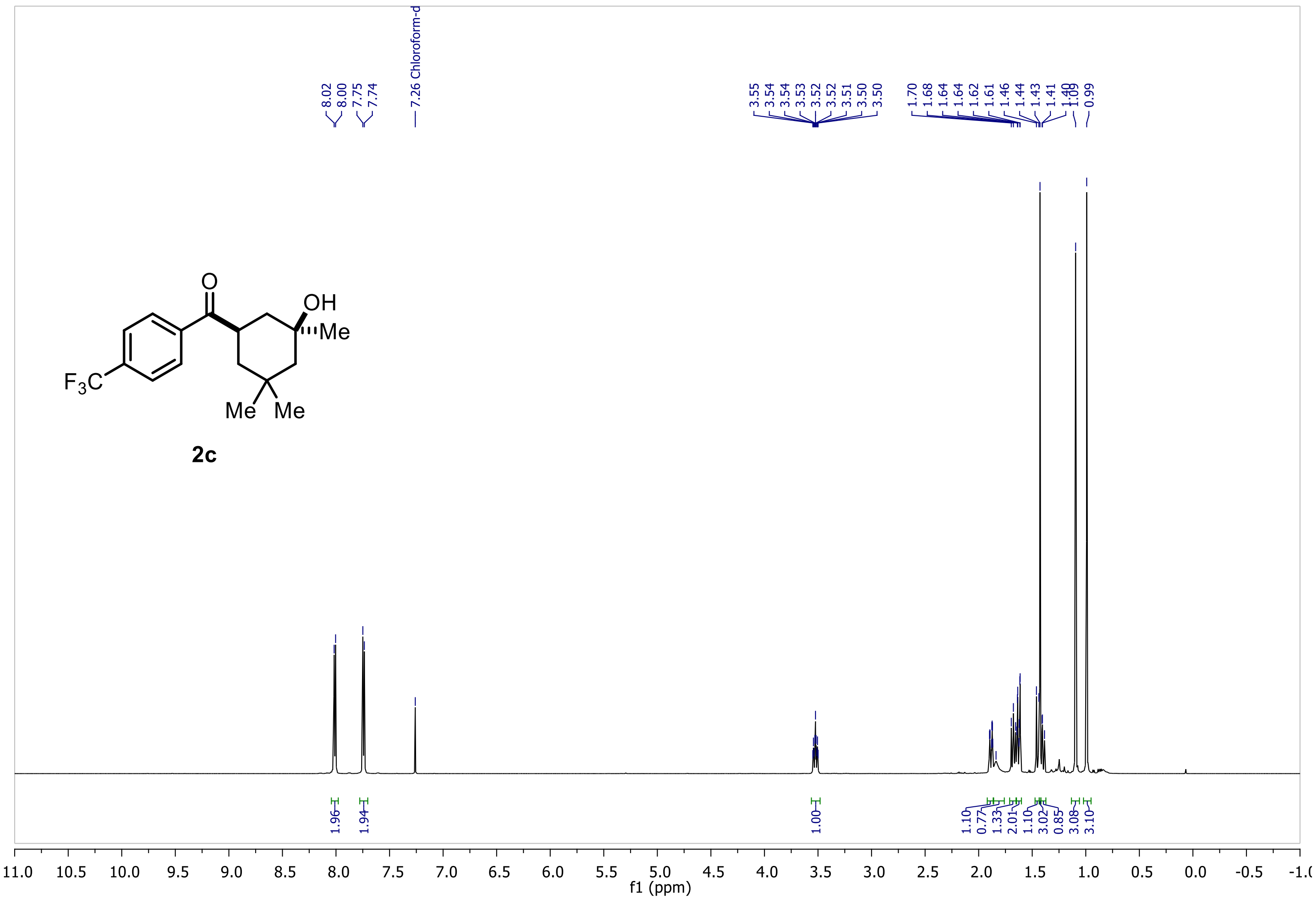

$^{13}C\{^1H\}$ NMR (151 MHz, $CDCl_3$)

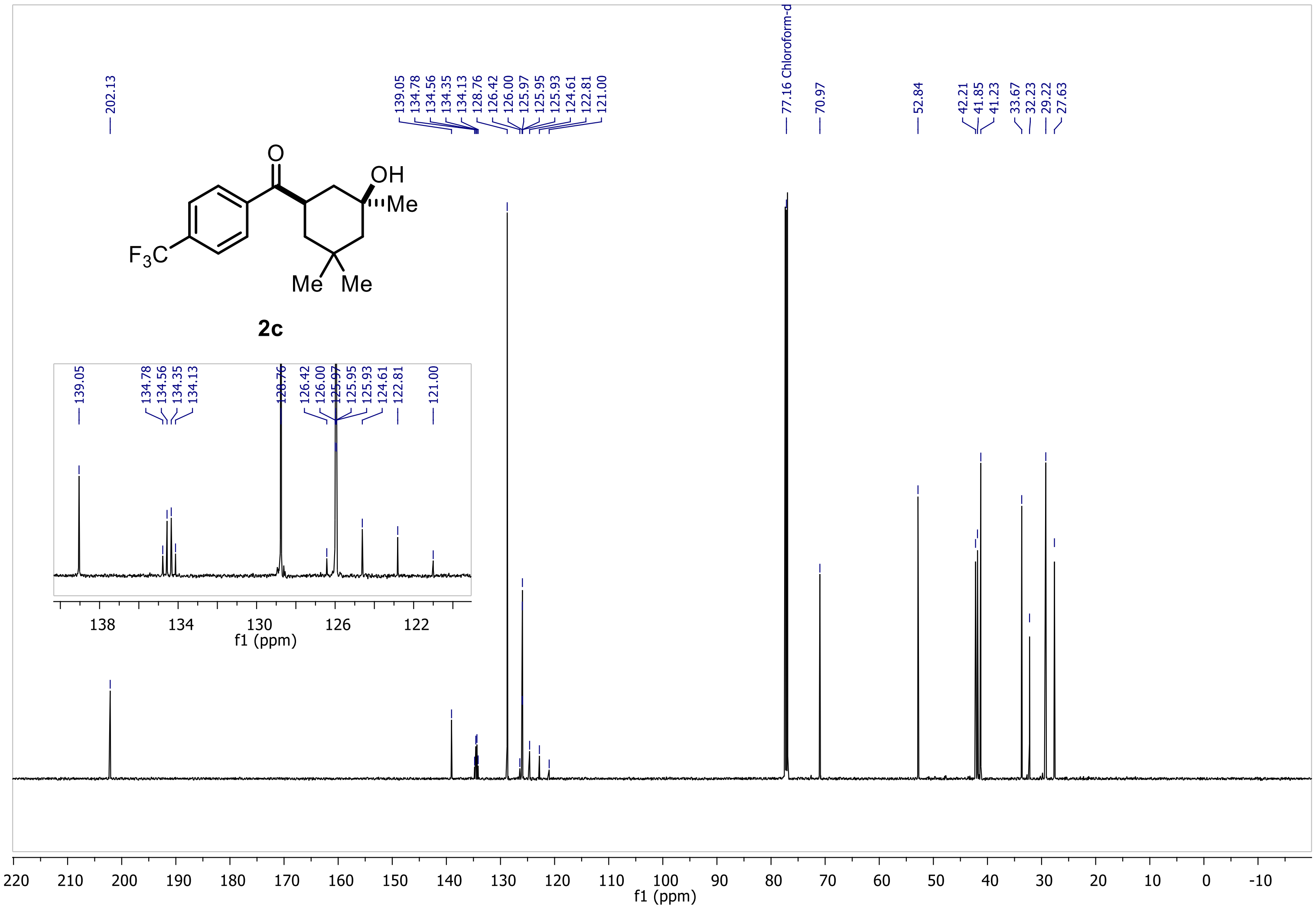

^{19}F NMR (565 MHz, $CDCl_3$)

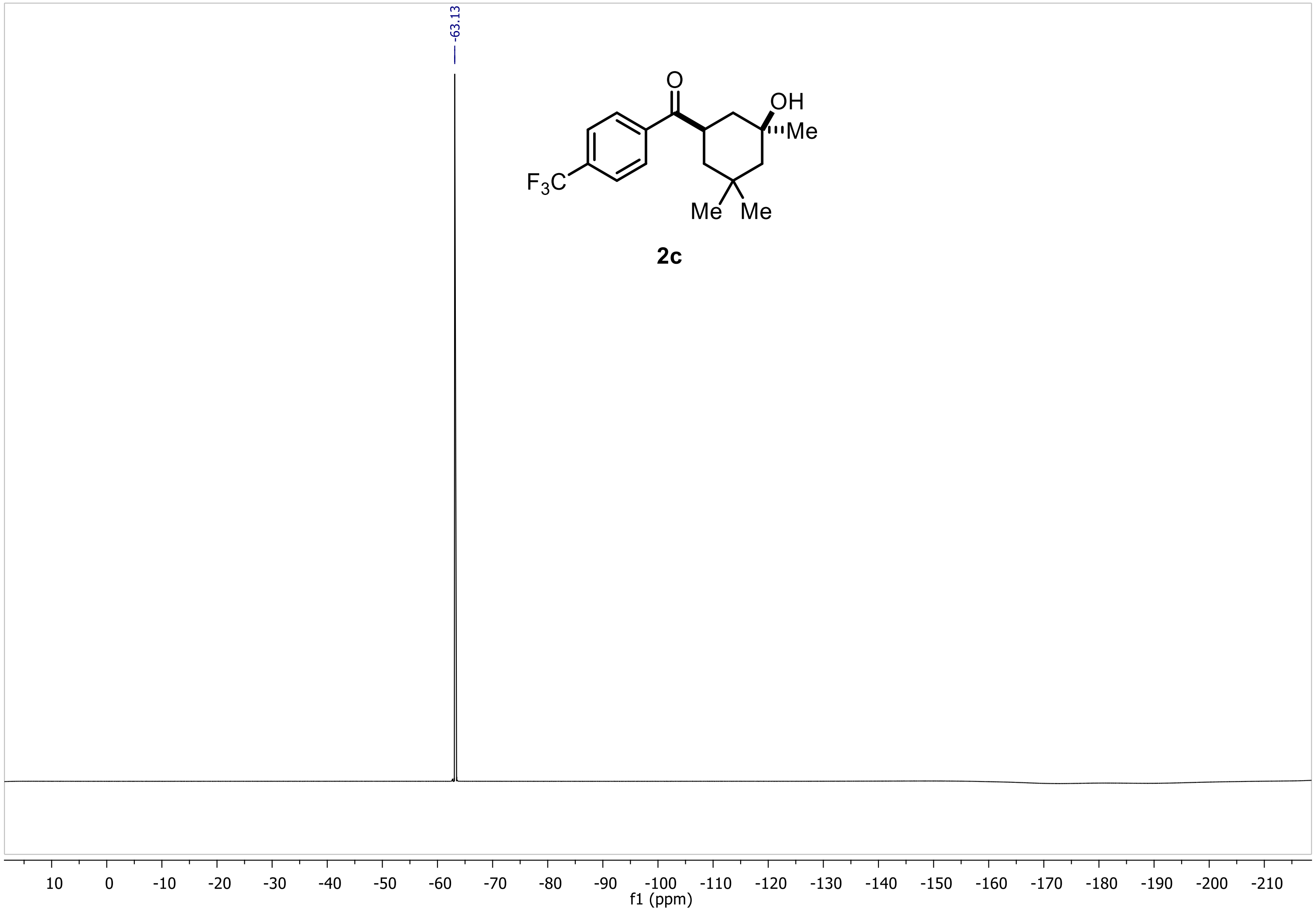

2c′ – *cis*-(1,3-*cis*-3-hydroxy-2,3,5-trimethylcyclohexyl)[4-(trifluoromethyl)phenyl]methanone

^{1}H NMR (600 MHz, CDCl$_3$)

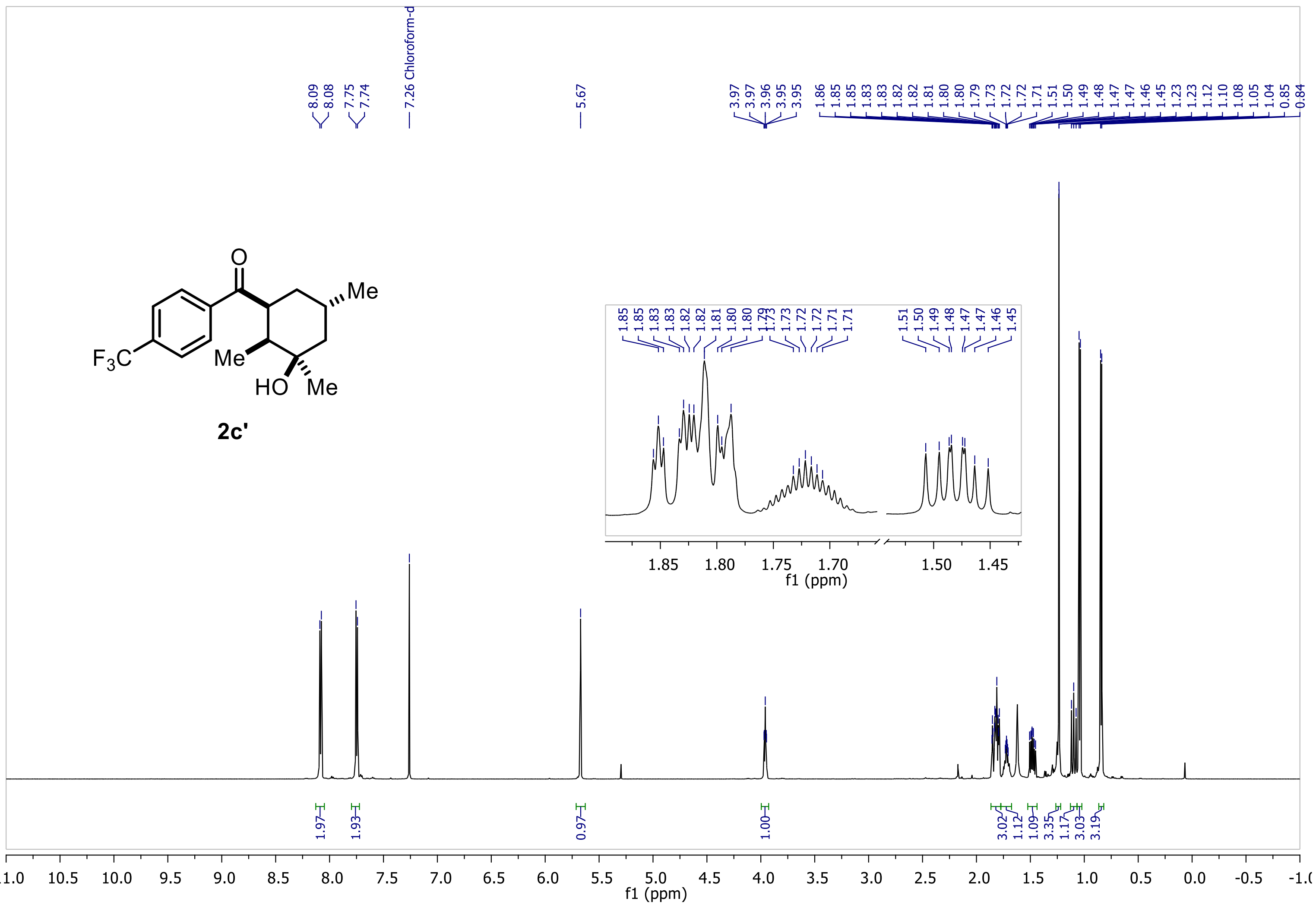

$^{13}C\{^{1}H\}$ NMR (151 MHz, $CDCl_3$)

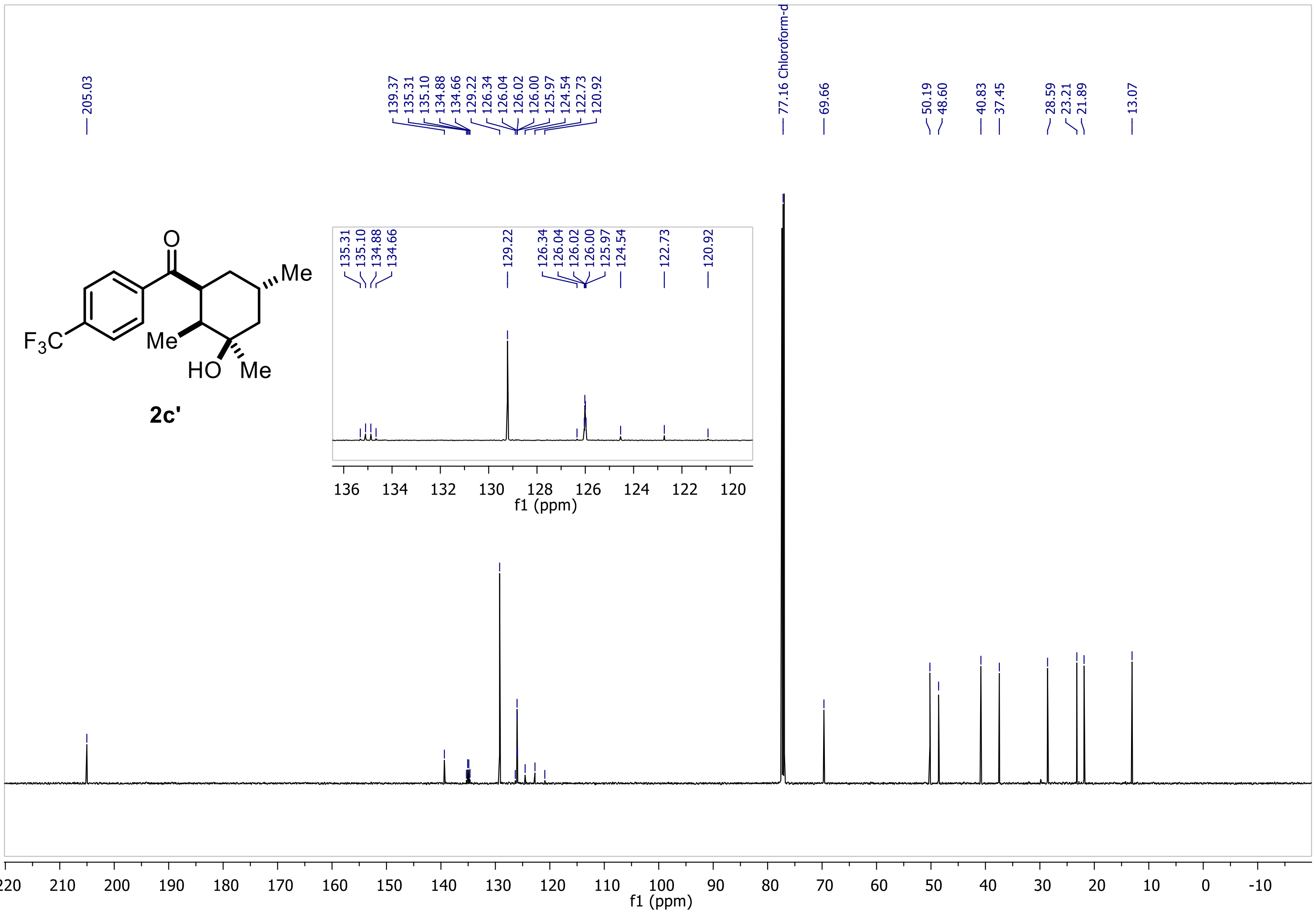

^{19}F NMR (565 MHz, CDCl$_3$)

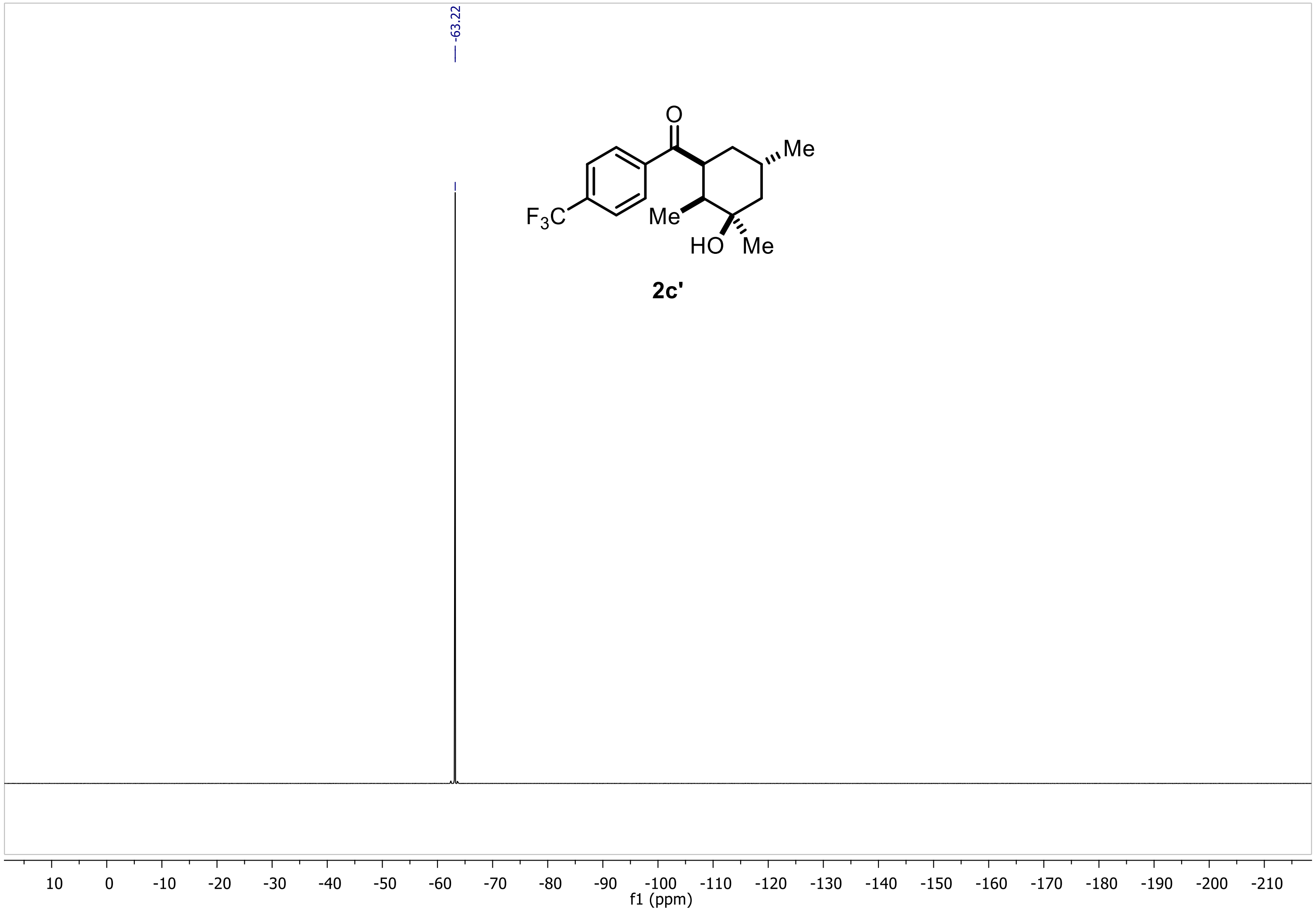

2f – *cis*-(Hydroxycyclopentyl)[4-(trifluoromethyl)phenyl]methanone

^{1}H NMR (600 MHz, $CDCl_3$)

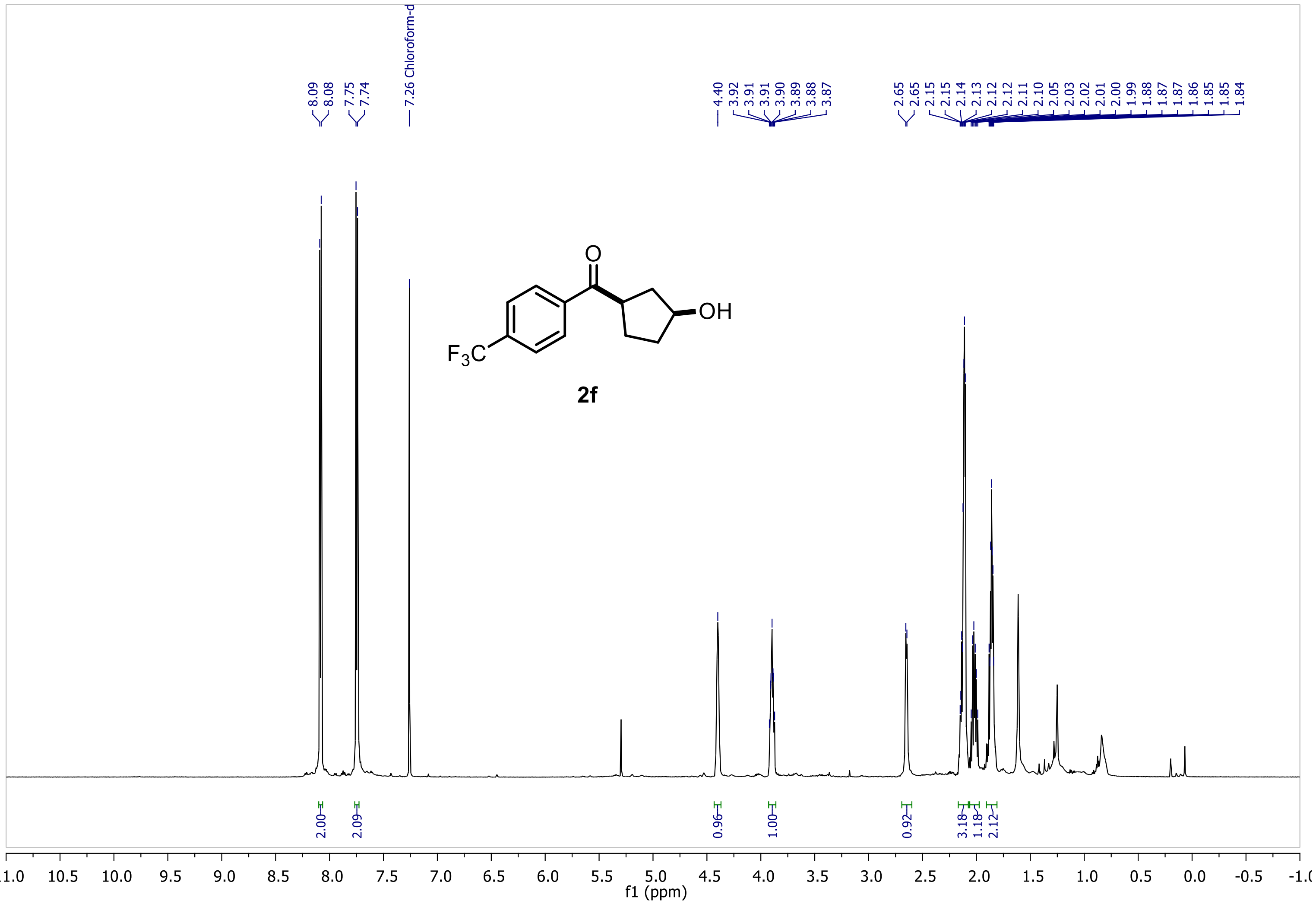

$^{13}C\{^{1}H\}$ NMR (151 MHz, $CDCl_3$)

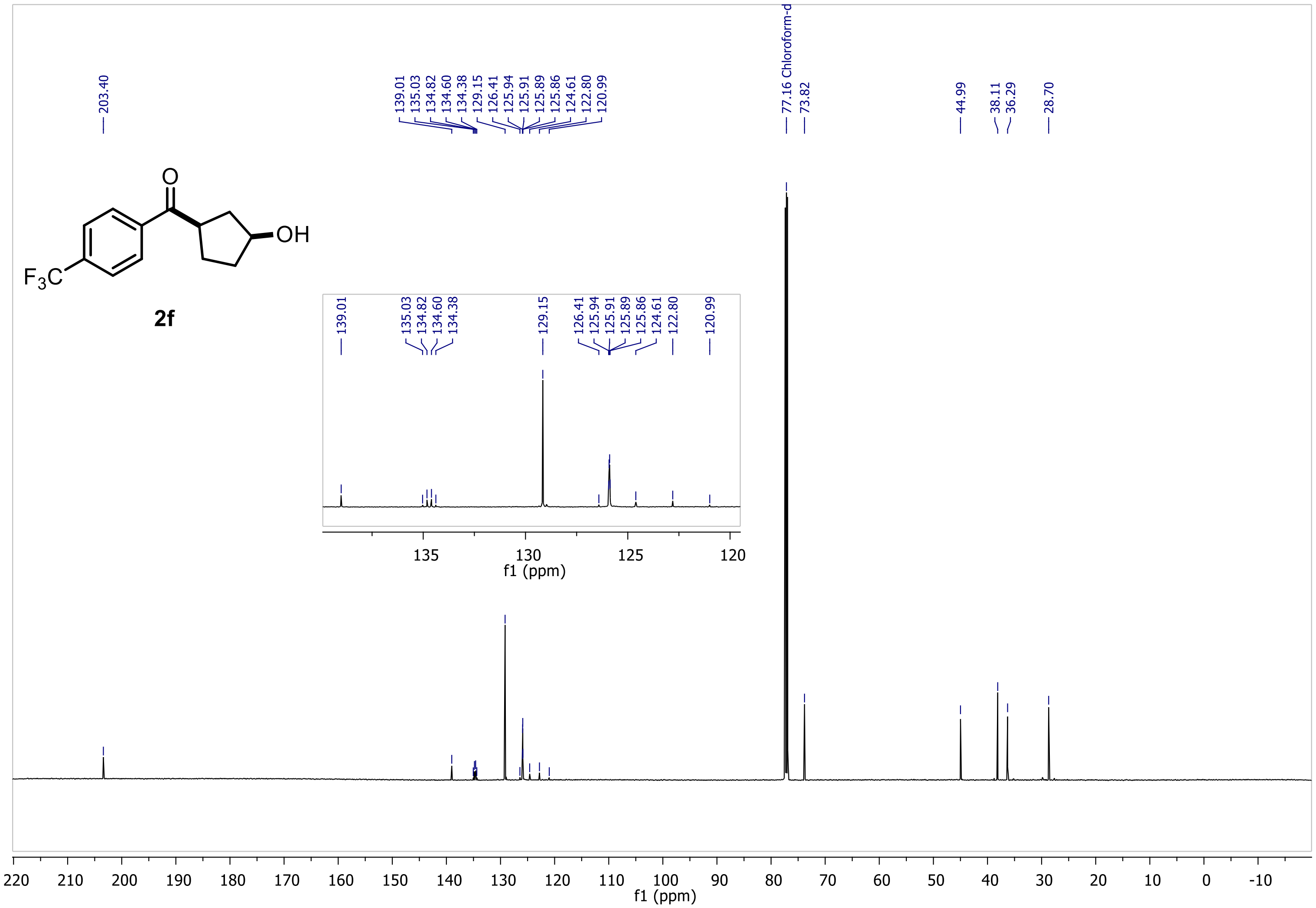

^{19}F NMR ($CDCl_3$, 565 MHz)

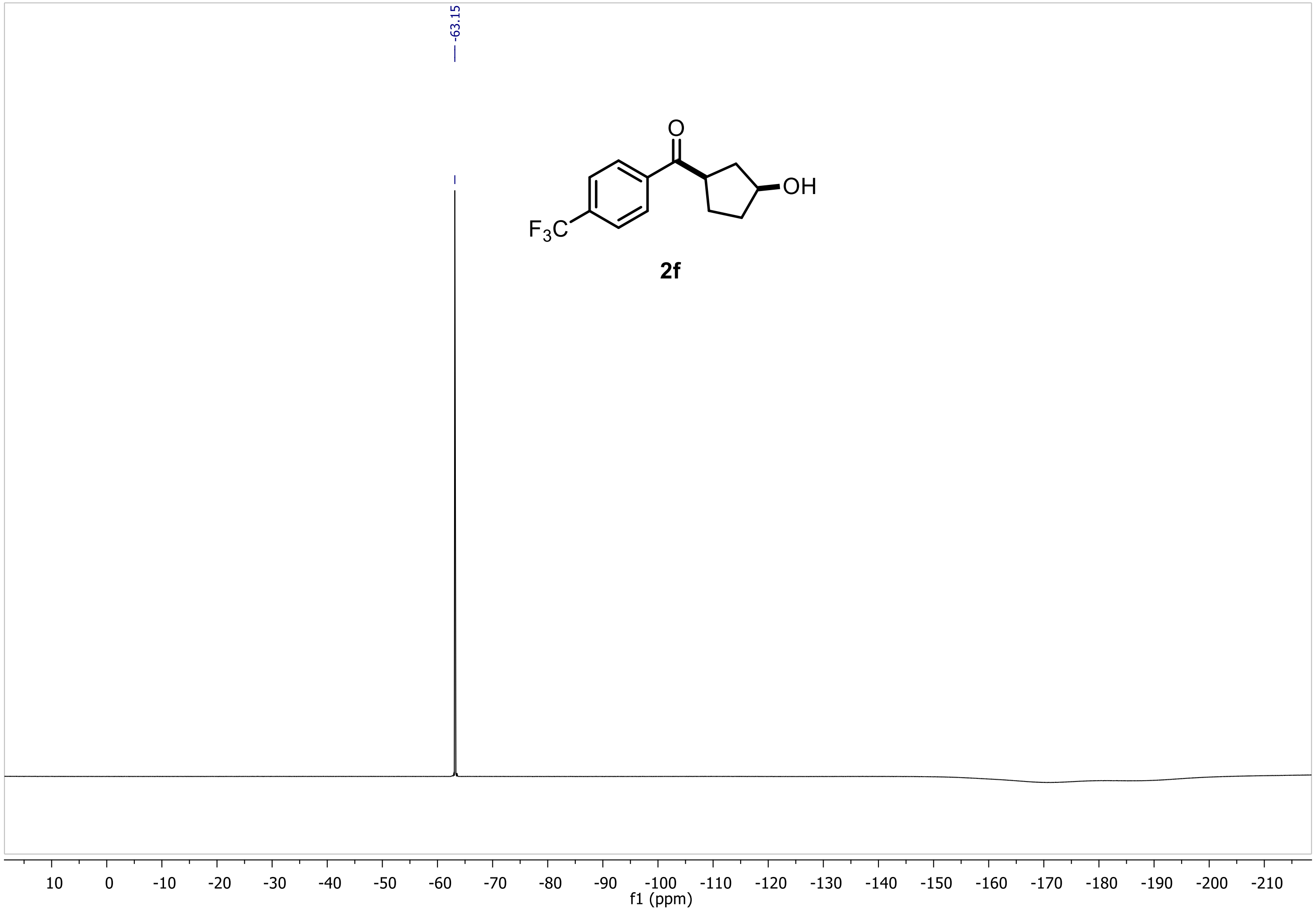

2g – *cis*-(3-Hydroxycyclopentyl)[3-(trifluoromethyl)phenyl]methanone

^{1}H NMR (700 MHz, $CDCl_3$)

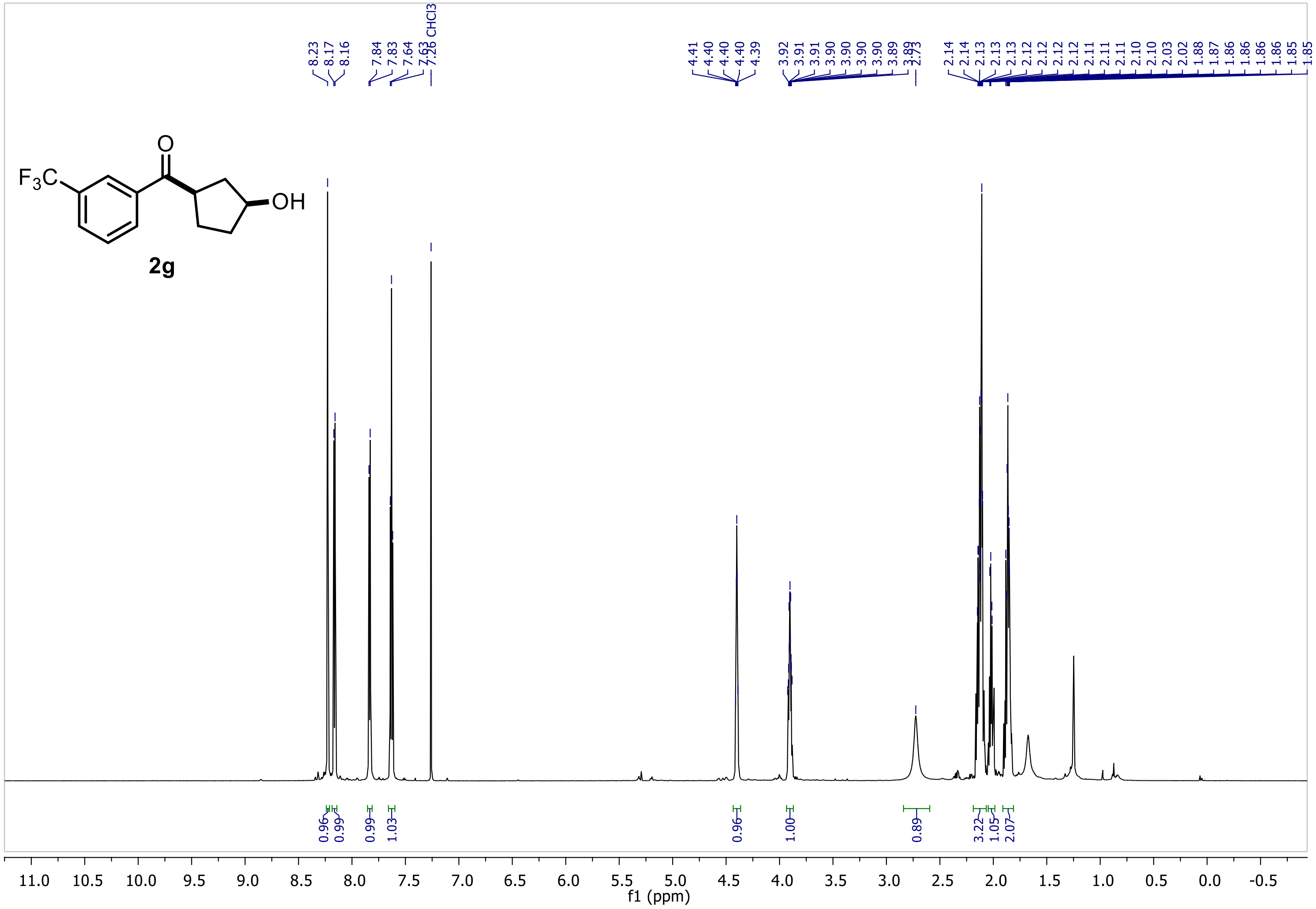

$^{13}C\{^{1}H\}$ NMR (176 MHz, $CDCl_3$)

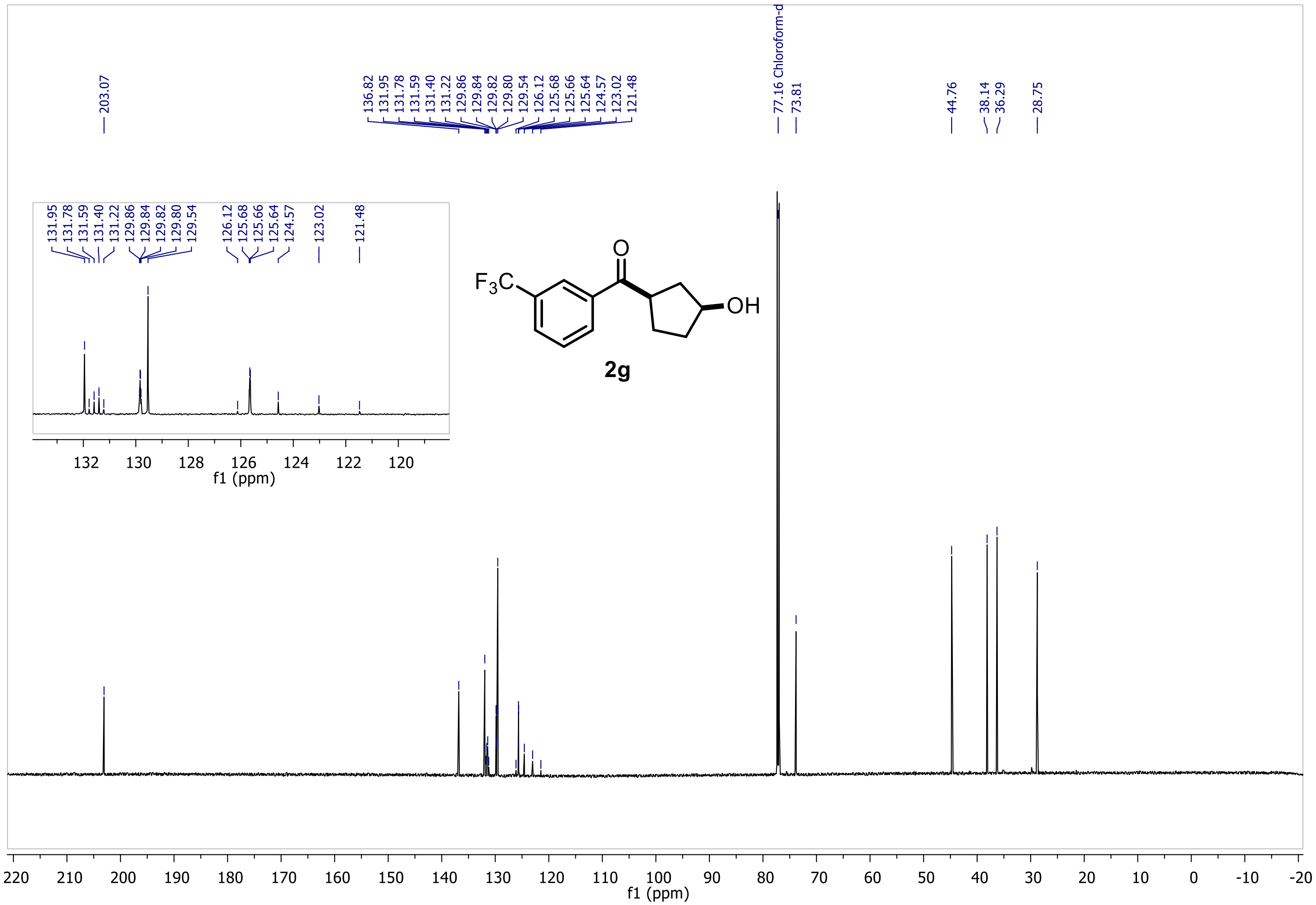

^{19}F NMR ($CDCl_3$, 659 MHz)

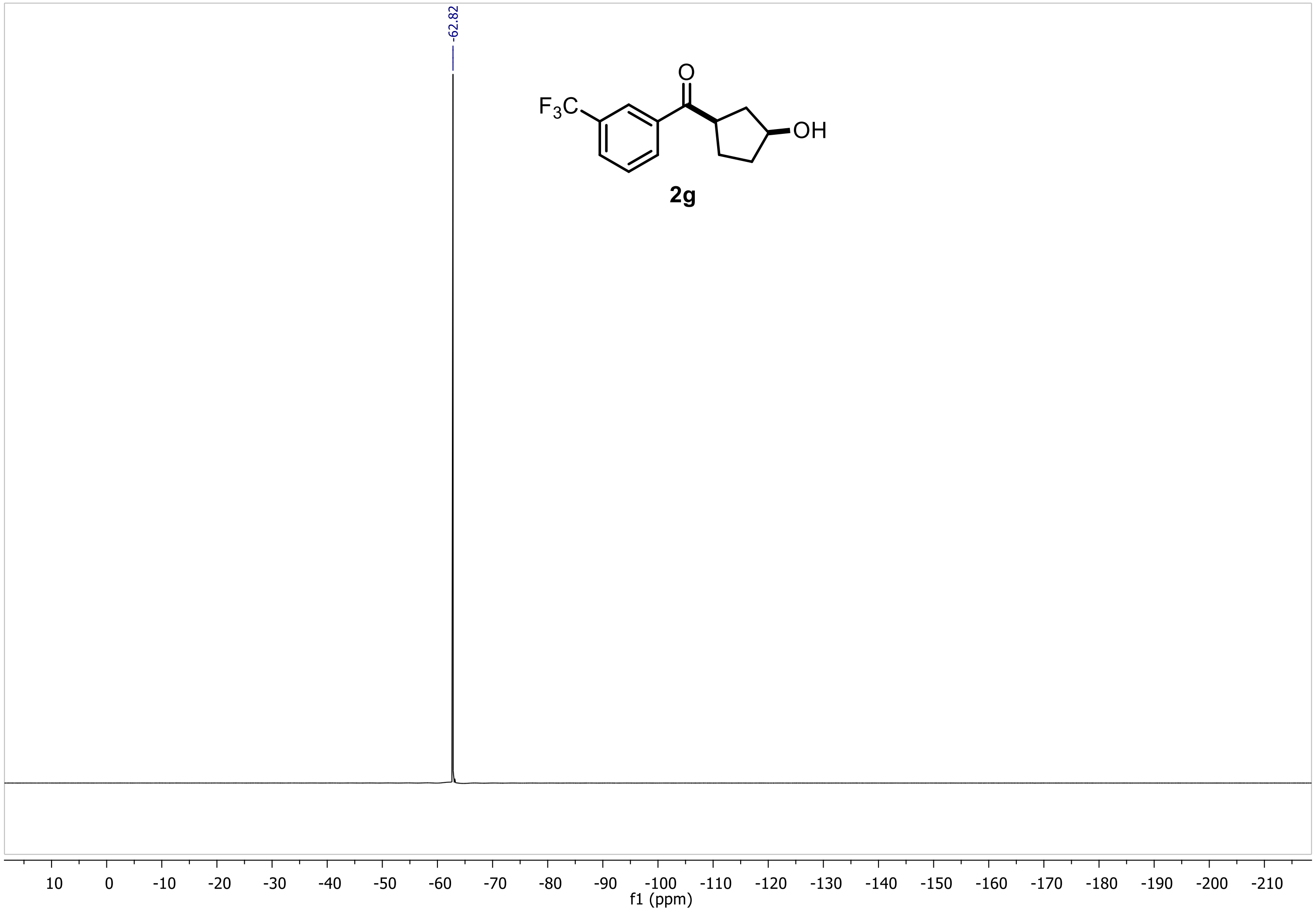

2h – *cis*-(3-Hydroxycyclopentyl)[2-(trifluoromethyl)phenyl]methanone

^{1}H NMR (400 MHz, $CDCl_3$)

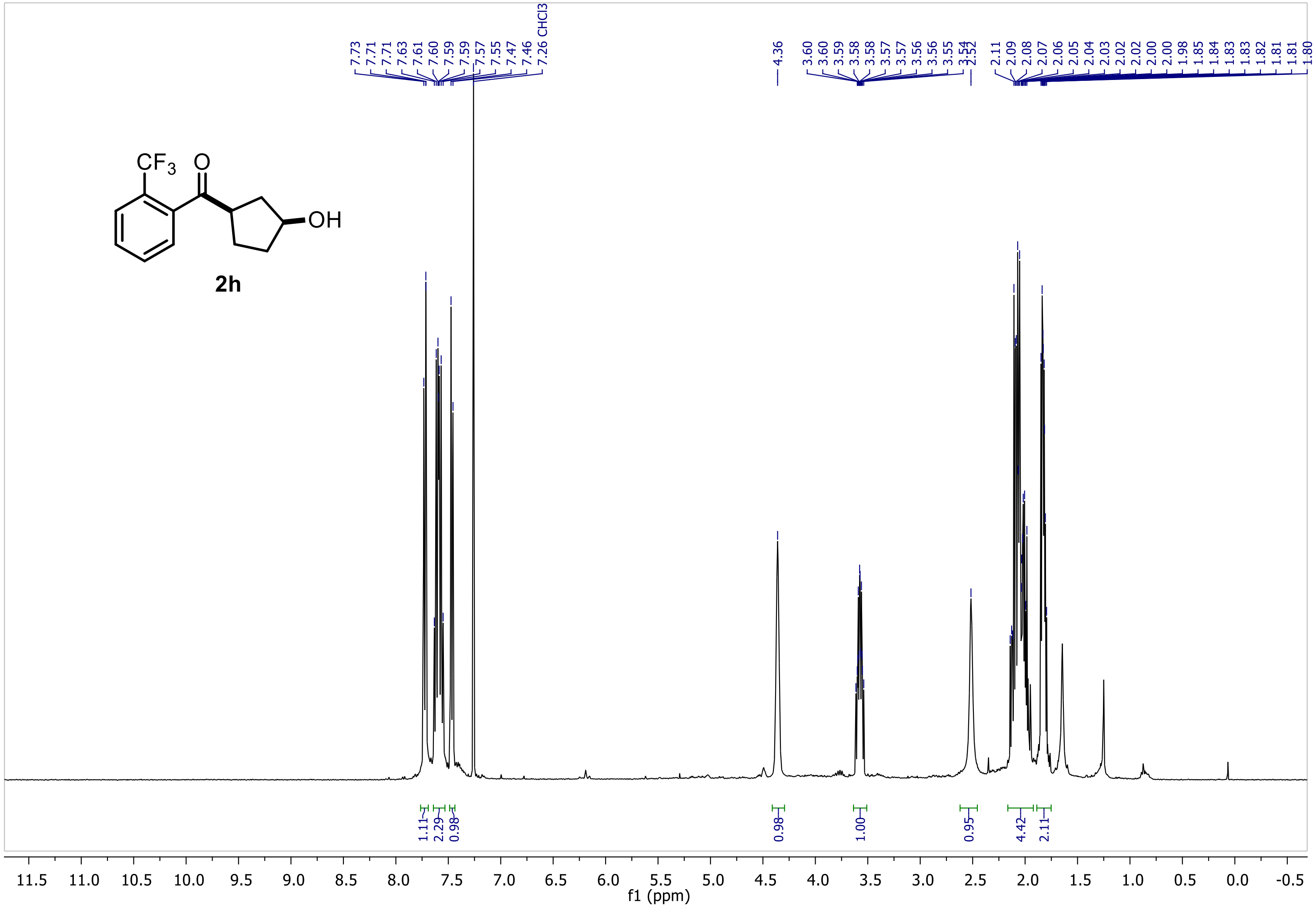

$^{13}C\{^1H\}$ NMR (101 MHz, $CDCl_3$)

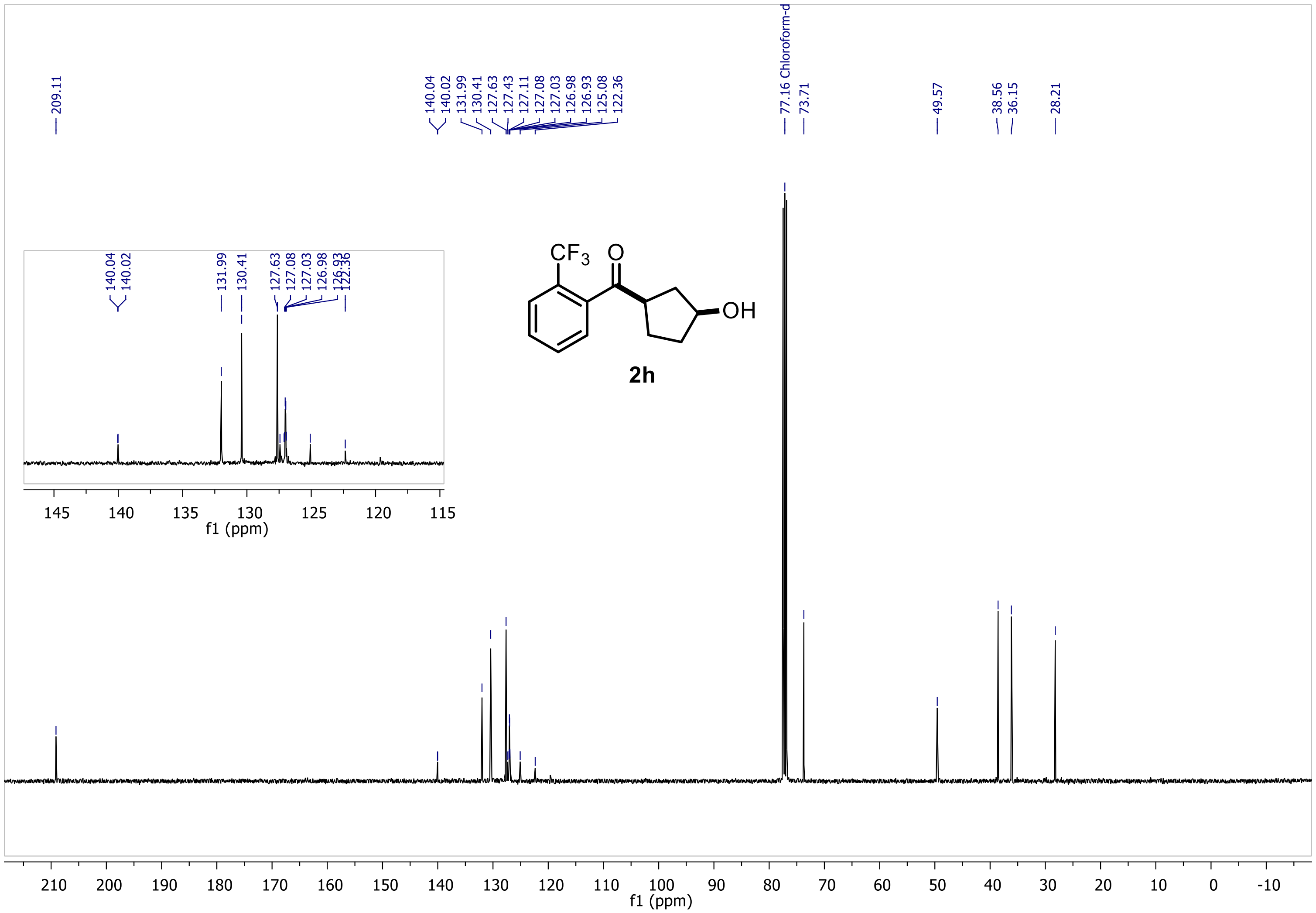

^{19}F NMR (376 MHz, $CDCl_3$)

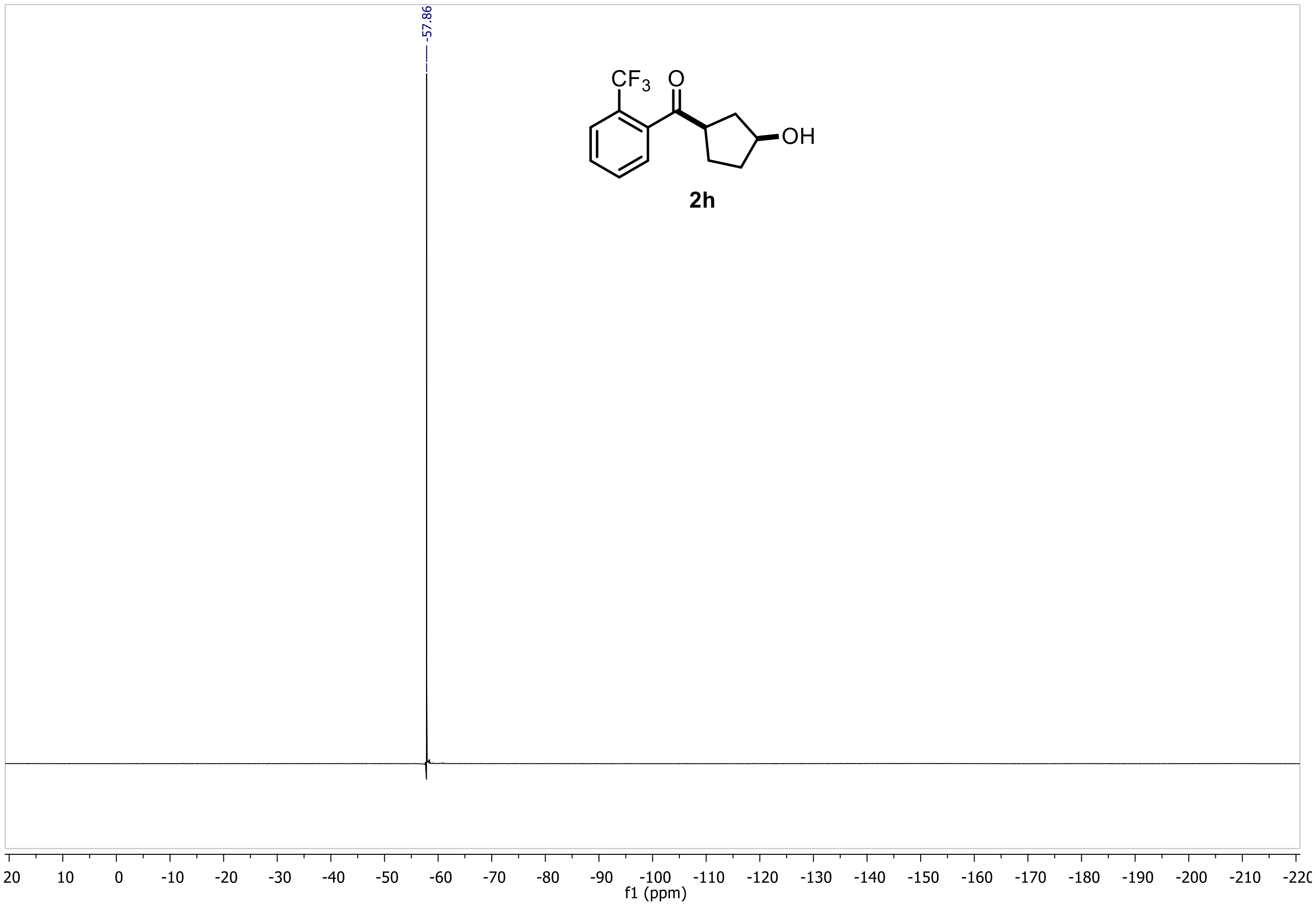

2i – *cis*-4-(3-Hydroxycyclopentane-1-carbonyl)benzonitrile

^{1}H NMR (600 MHz, $CDCl_3$)

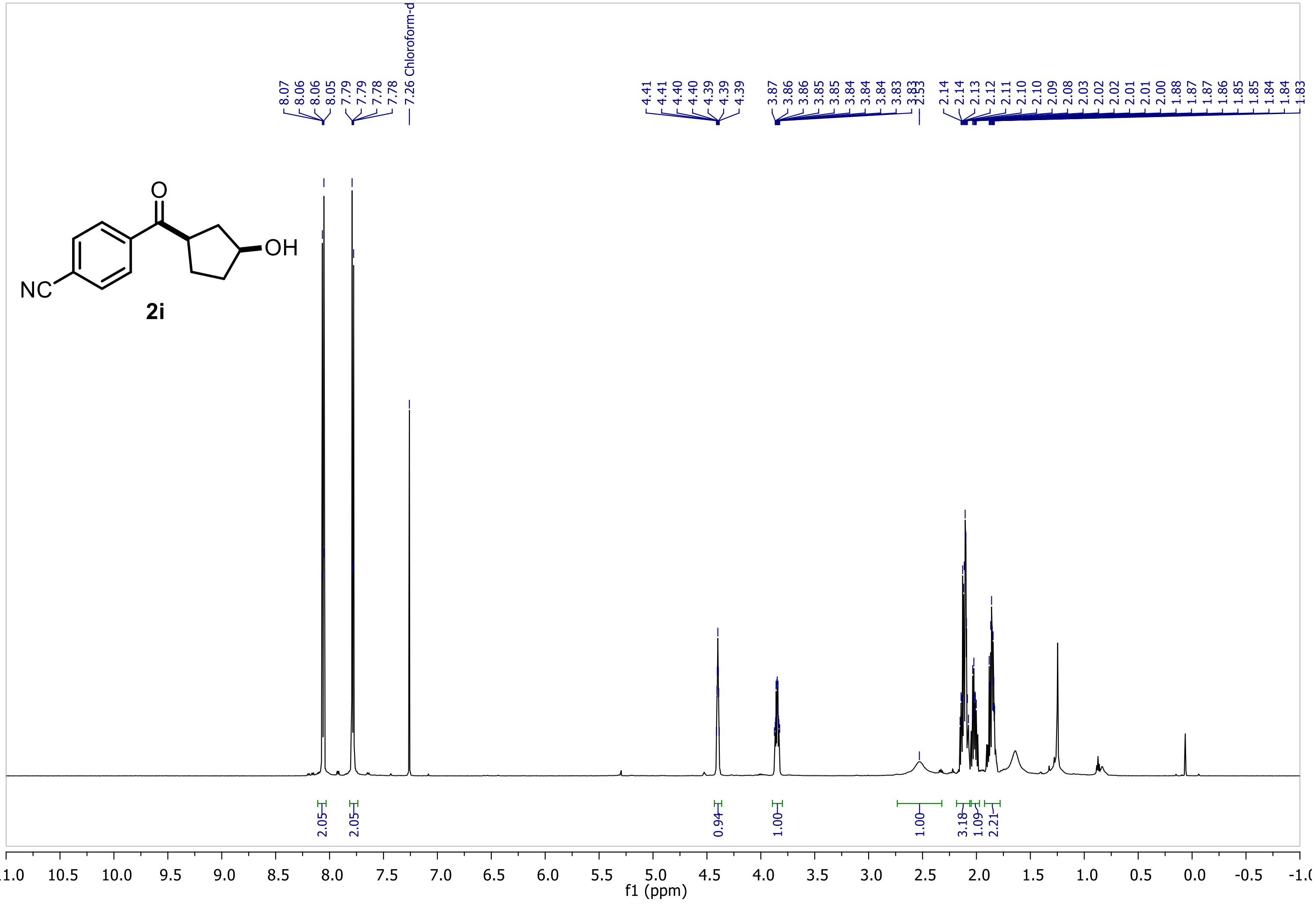

$^{13}C\{^1H\}$ NMR (151 MHz, CDCl$_3$)

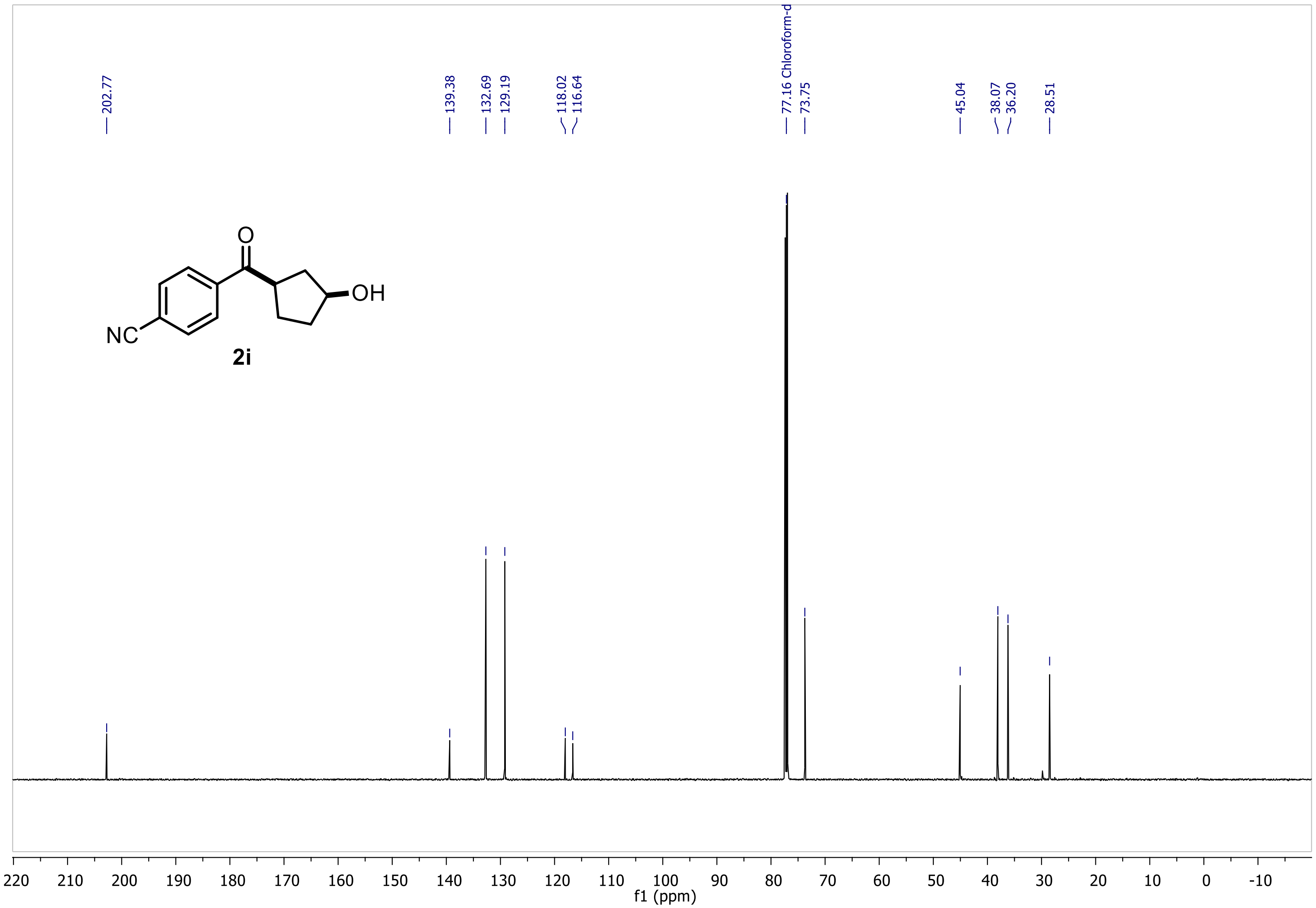

2j – *cis*-Methyl 4-(3-hydroxycyclopentane-1-carbonyl)benzoate

^{1}H NMR (400 MHz, $CDCl_3$)

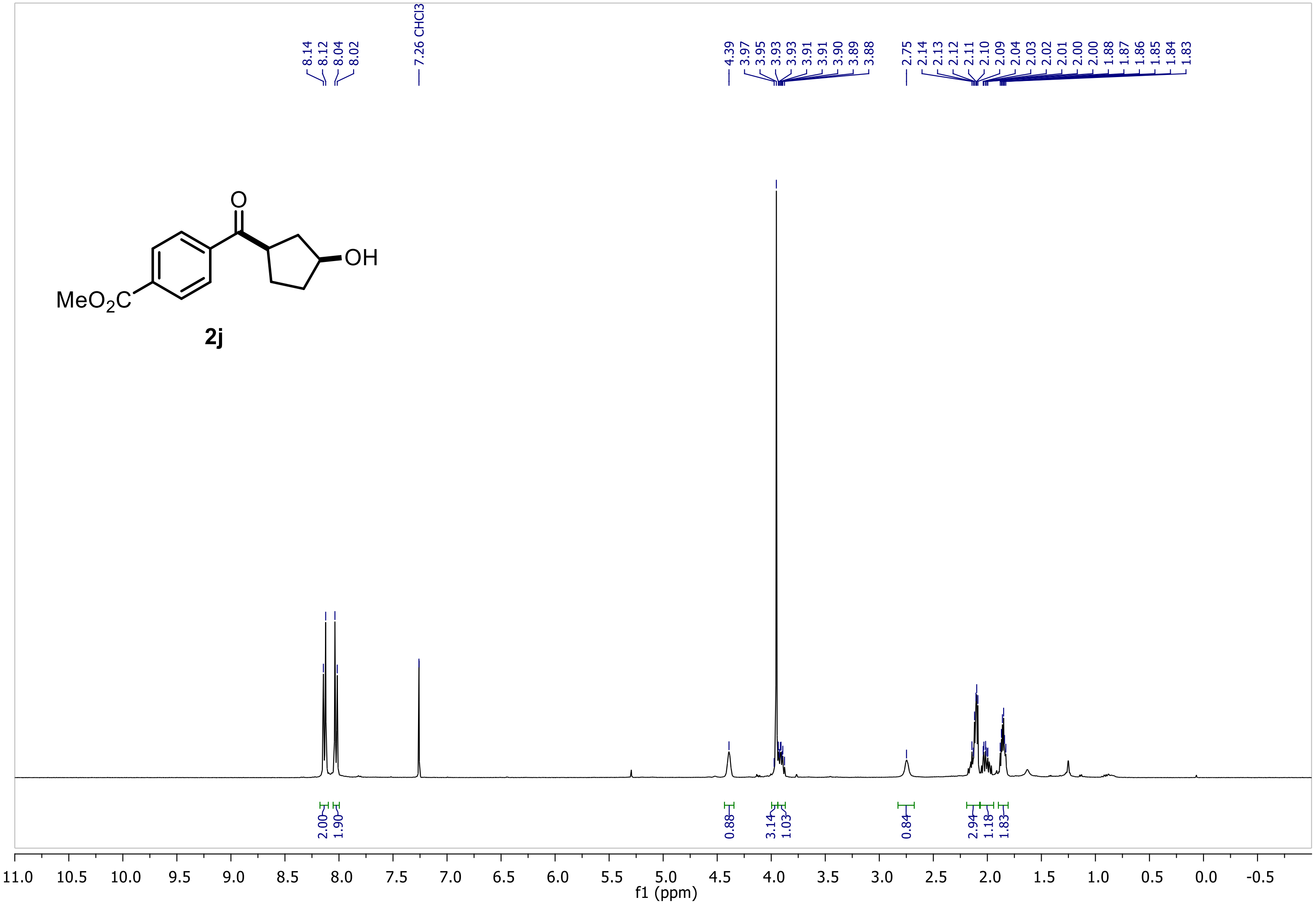

$^{13}C\{^1H\}$ NMR (101 MHz, $CDCl_3$)

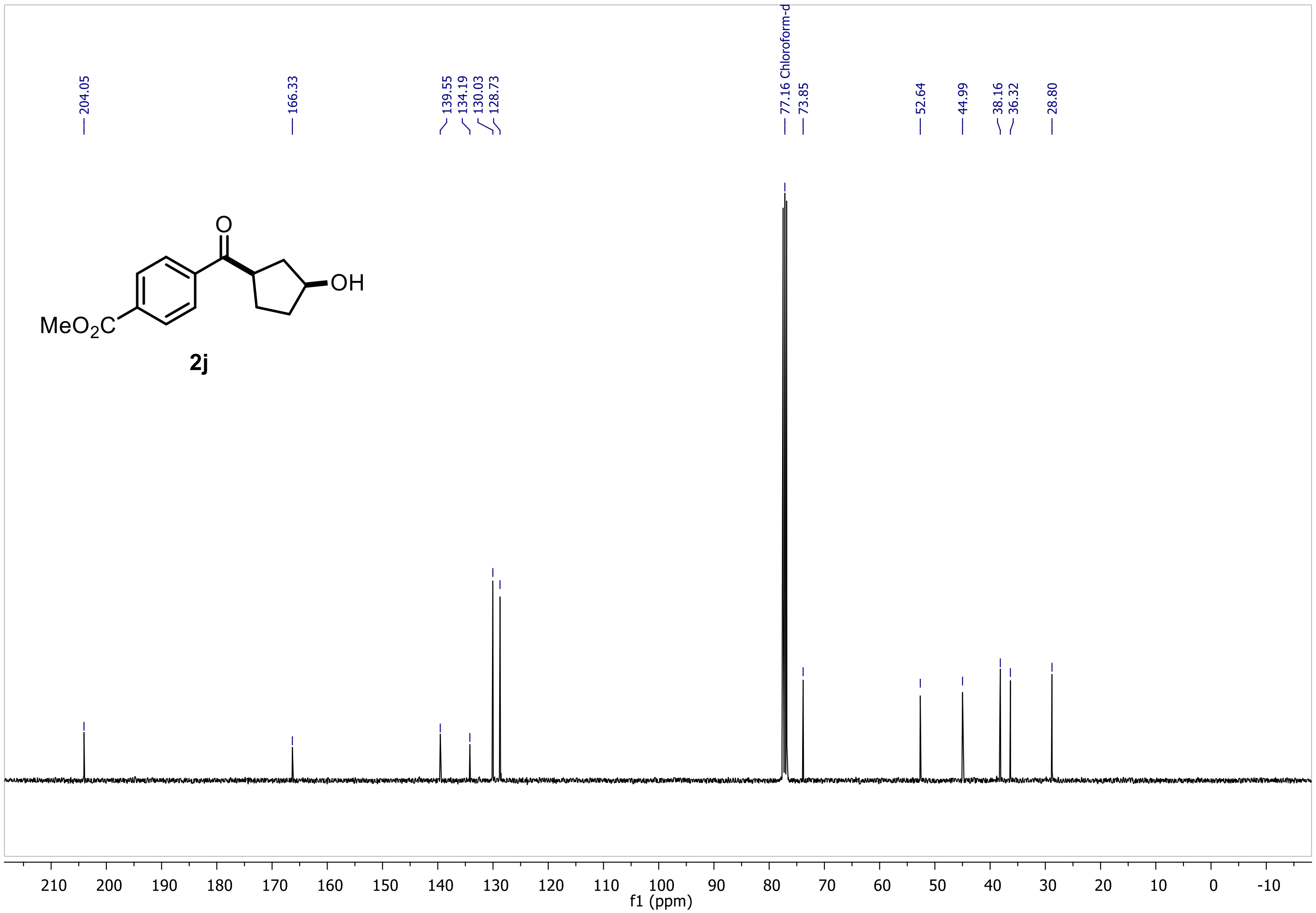

2k – (2,6-dichlorophenyl)(3-hydroxycyclopentyl)methanone

^{1}H NMR (400 MHz, $CDCl_3$)

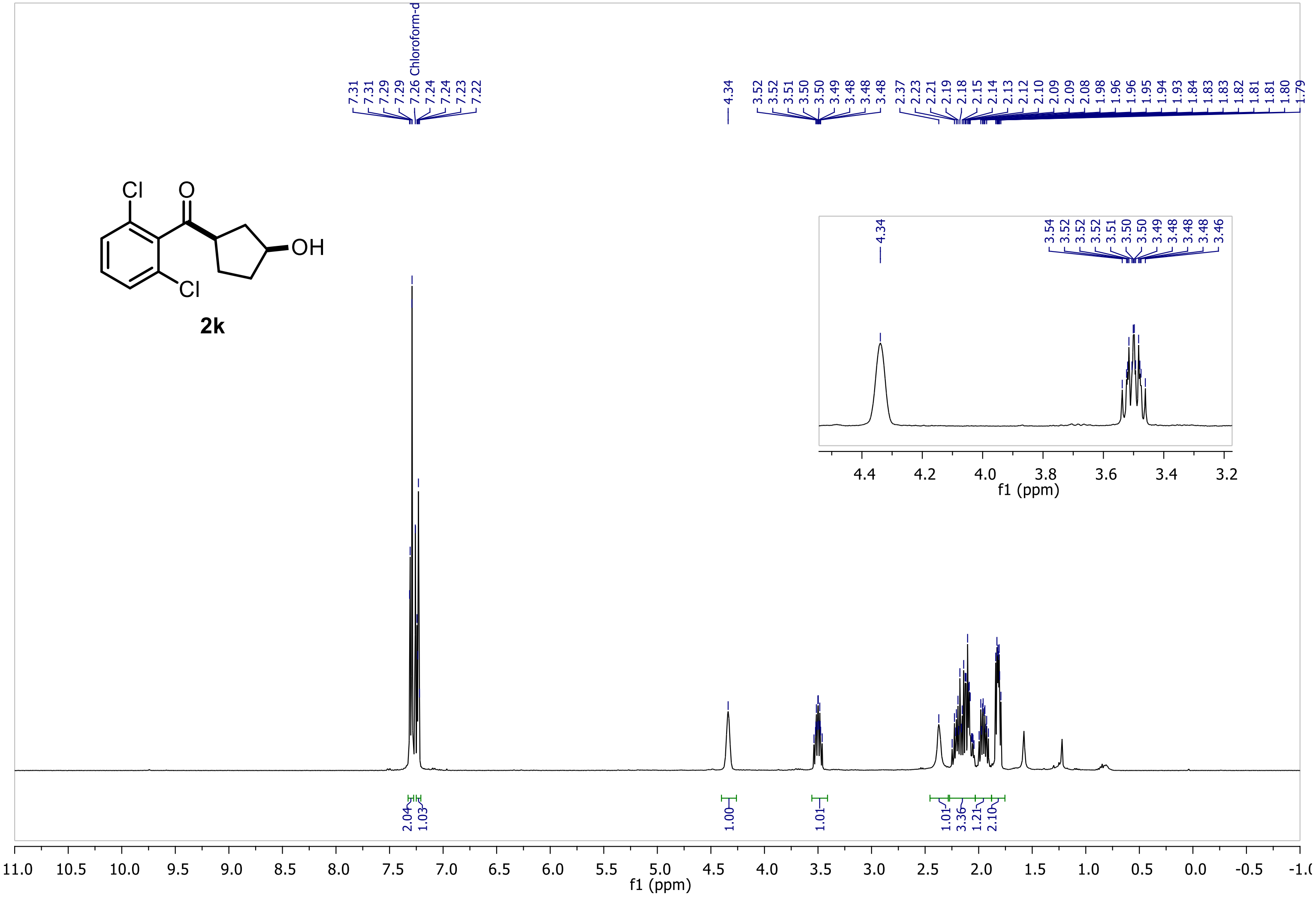

^{13}C (DEPT 135) NMR (101 MHz, $CDCl_3$)

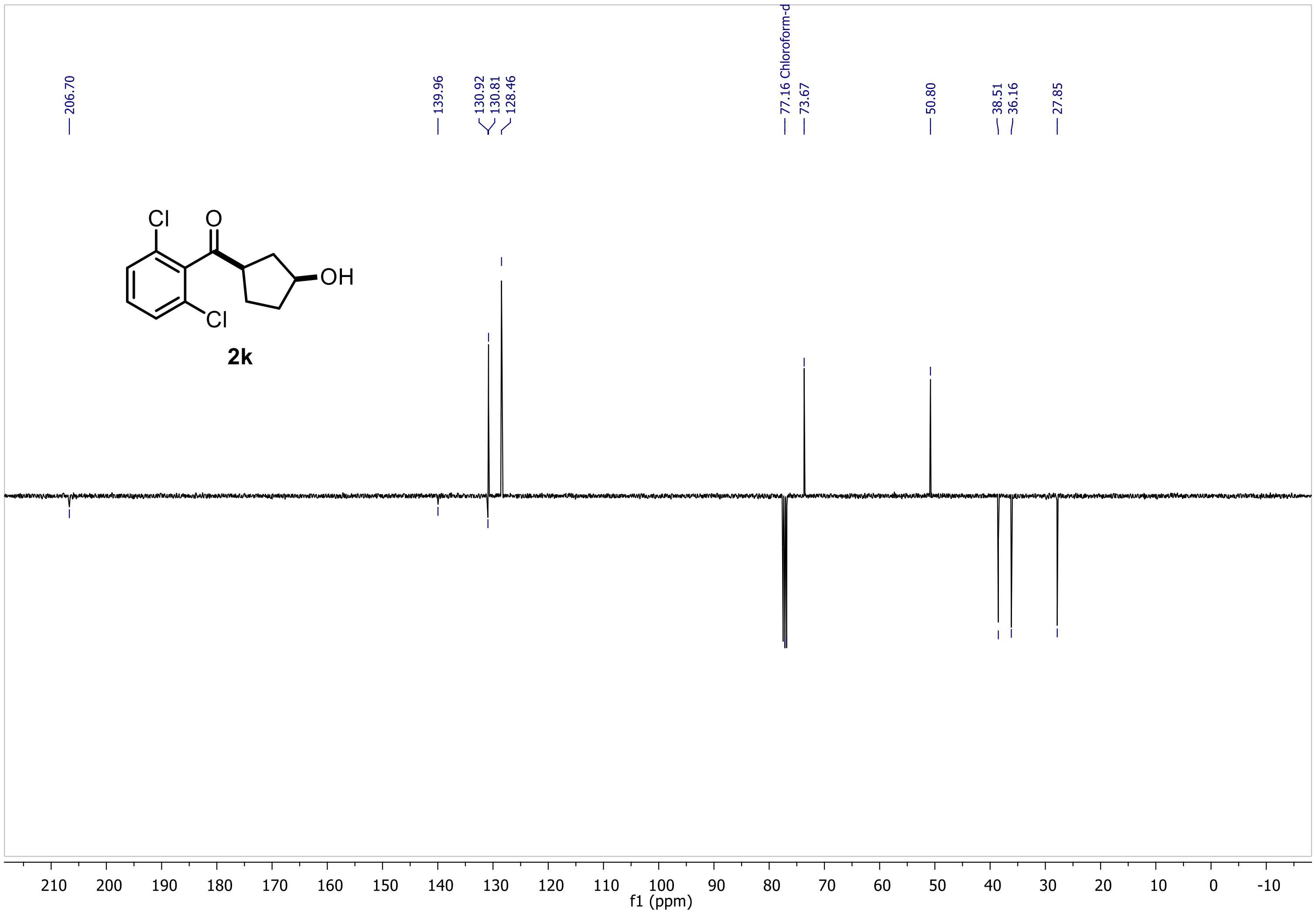

2l – *cis*-(4-bromophenyl)(3-hydroxycyclopentyl)methanone

^{1}H NMR (700 MHz, $CDCl_3$)

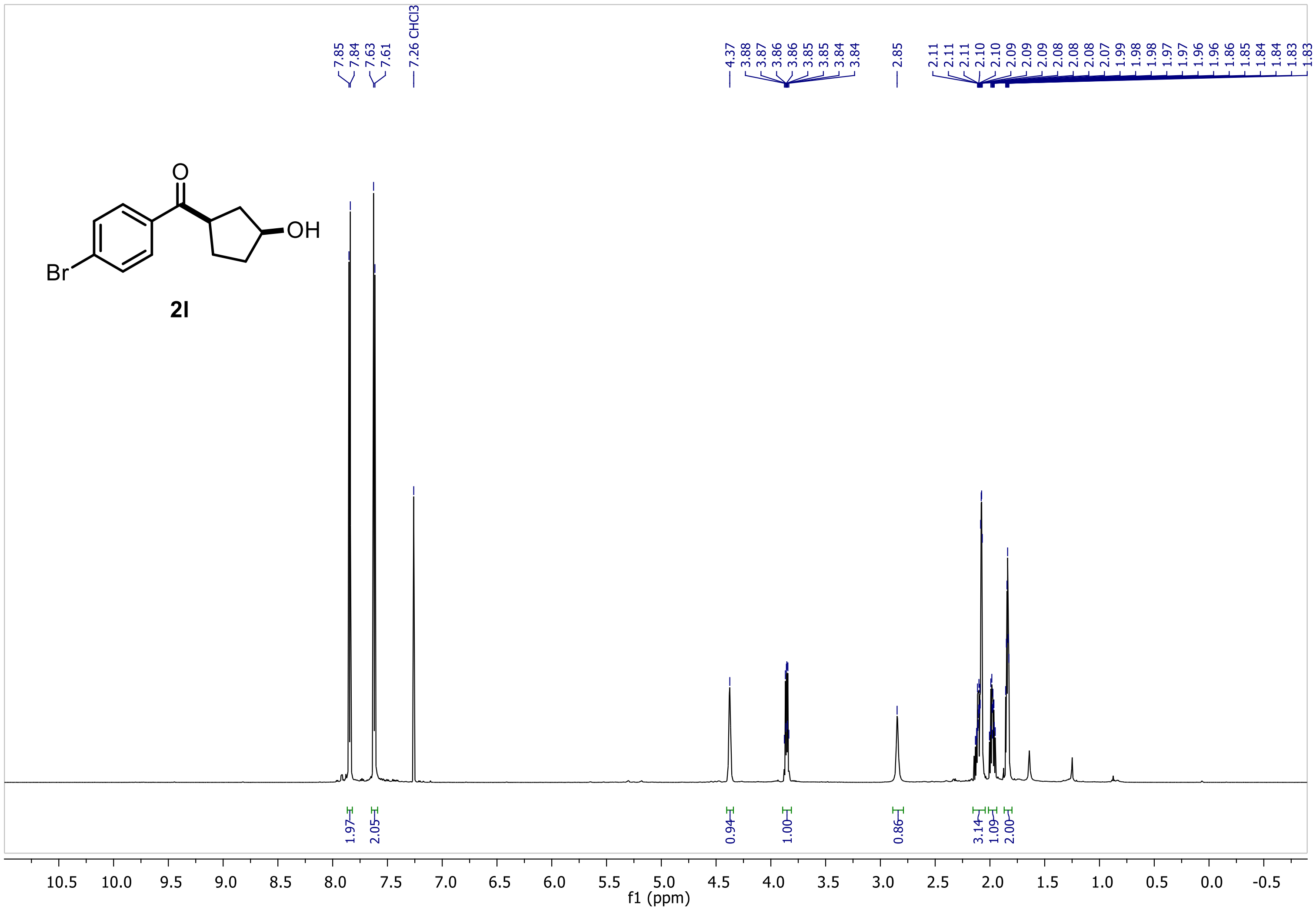

$^{13}C\{^{1}H\}$ NMR (176 MHz, $CDCl_3$)

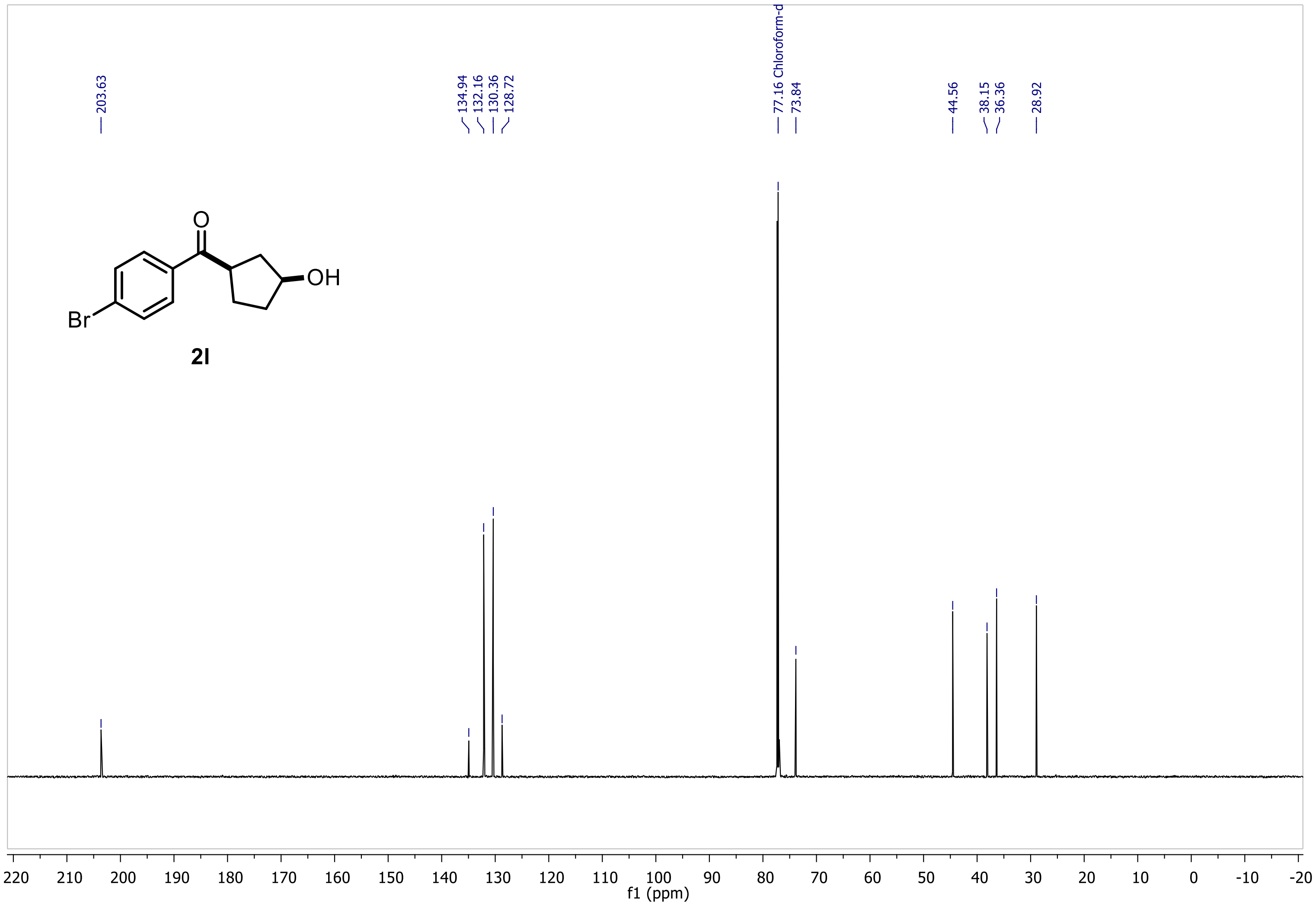

2m – *cis*-(3-Hydroxycyclopentyl)(phenyl)methanone

^{1}H NMR (400 MHz, $CDCl_3$)

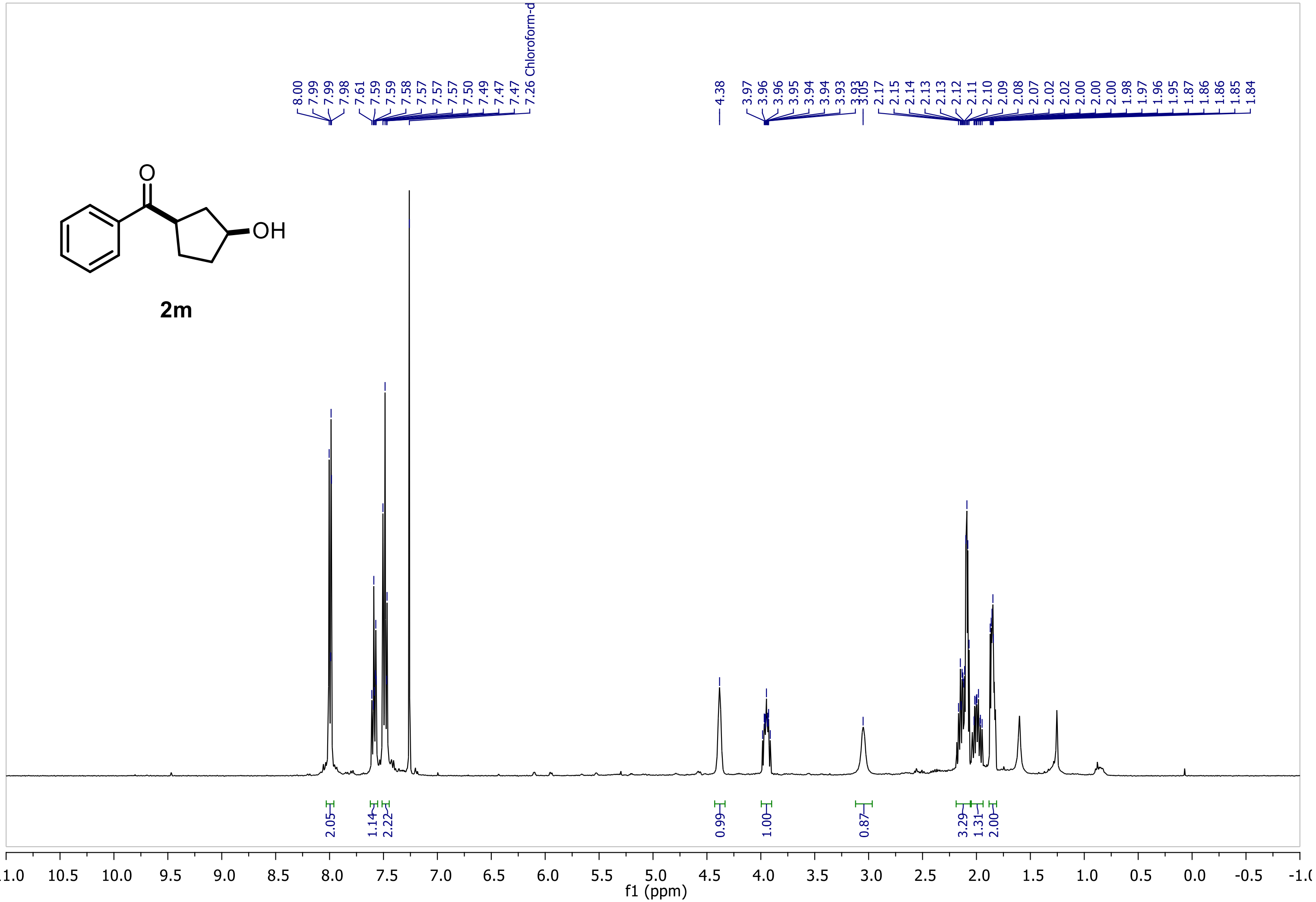

^{13}C NMR (101 MHz, $CDCl_3$)

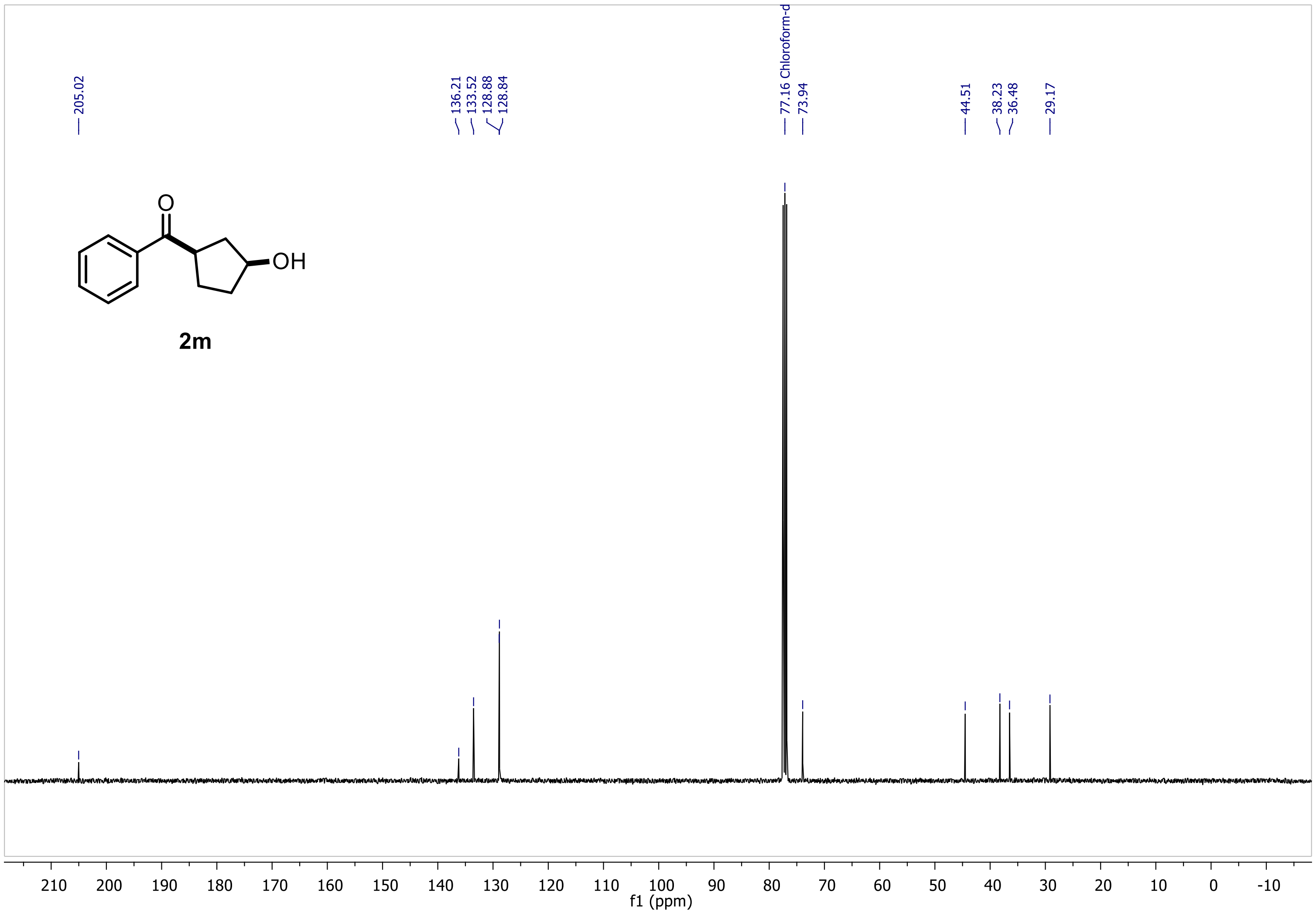

2n – *cis*-(3-Hydroxycyclopentyl)(naphthalen-2-yl)methanone

^{1}H NMR (400 MHz, $CDCl_3$)

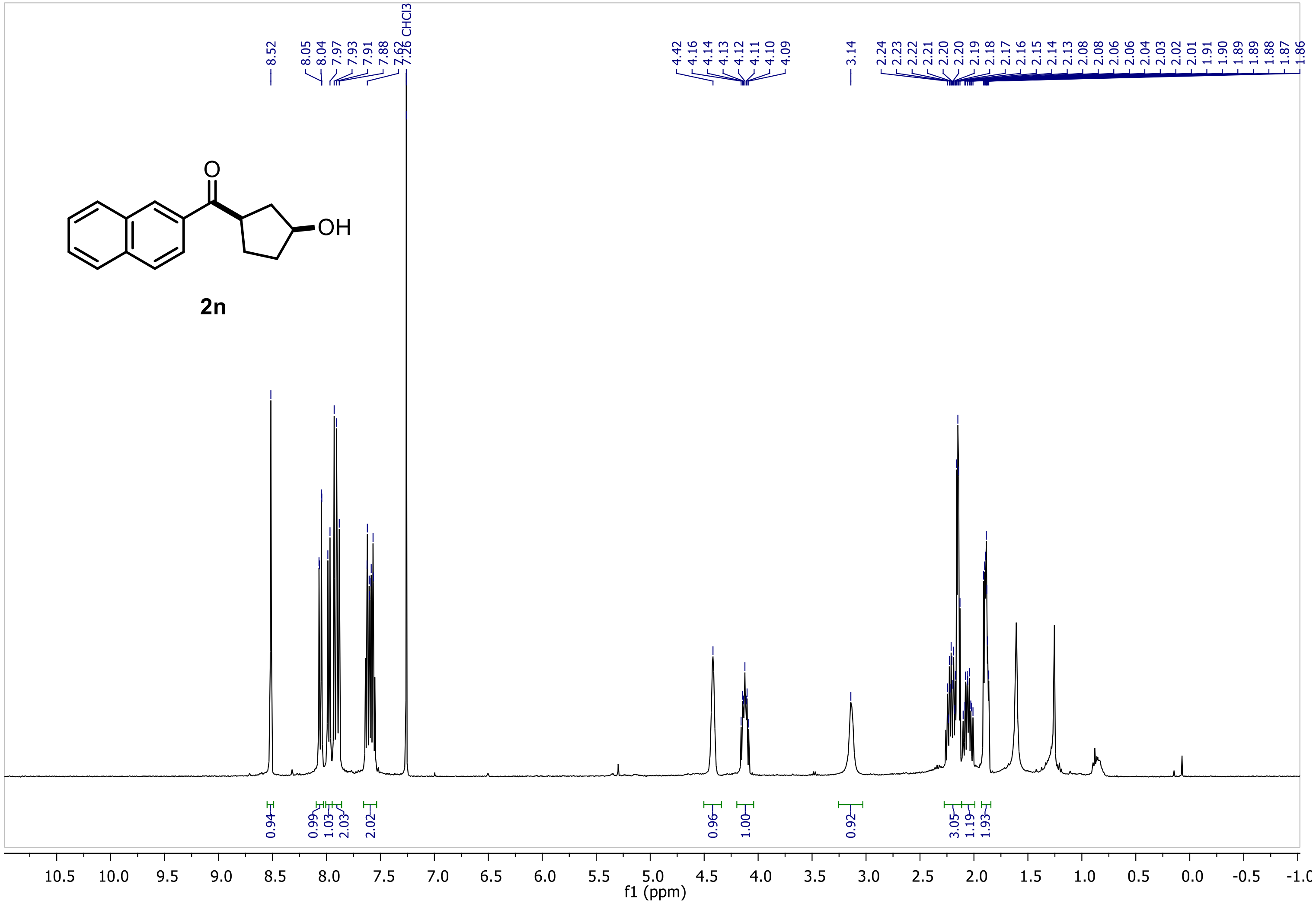

$^{13}C\{^{1}H\}$ NMR (101 MHz, $CDCl_3$)

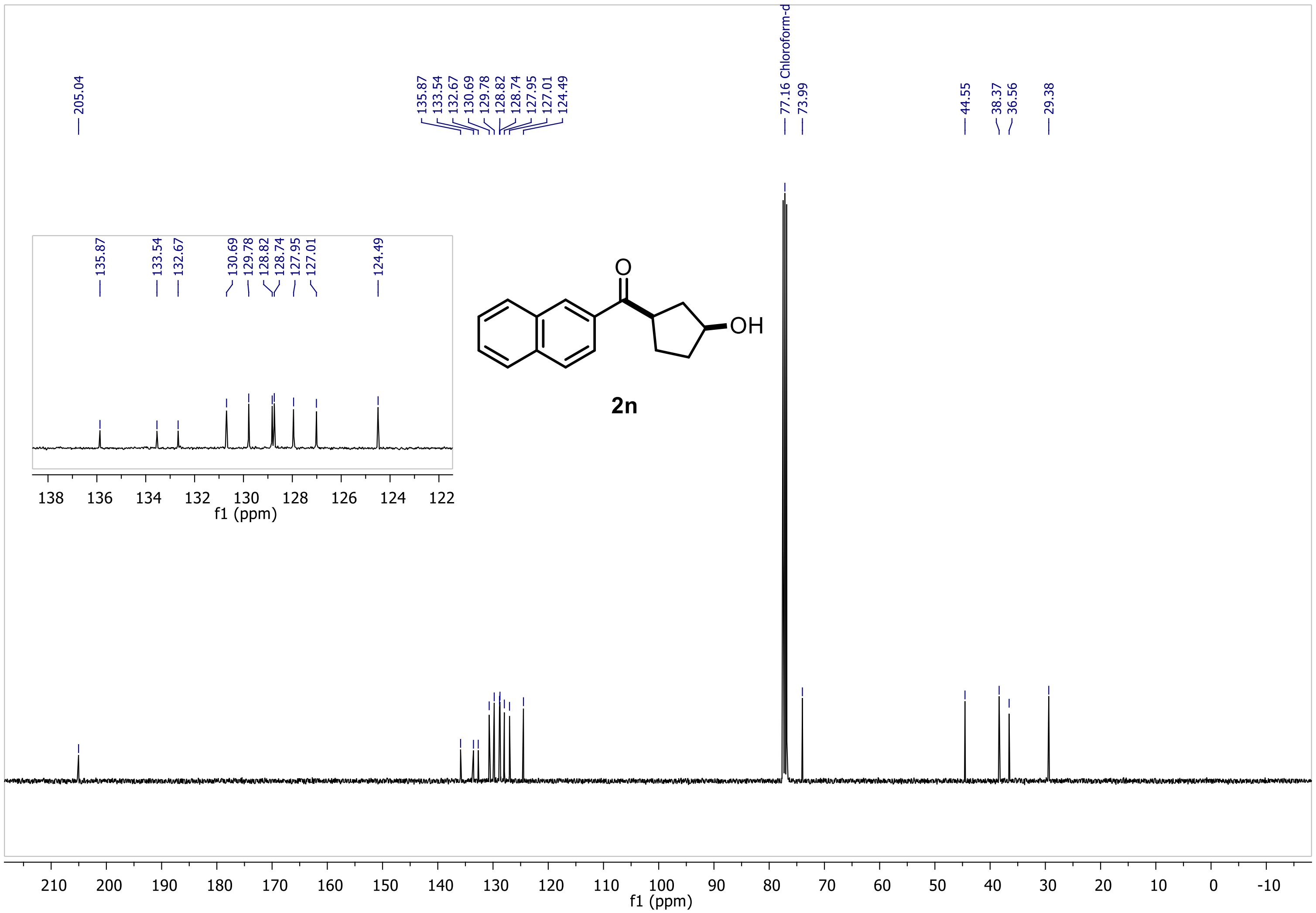

2o – *cis*-3-Hydroxy-[4-(trifluoromethyl)phenyl]methanone

^{1}H NMR (400 MHz, $CDCl_3$)

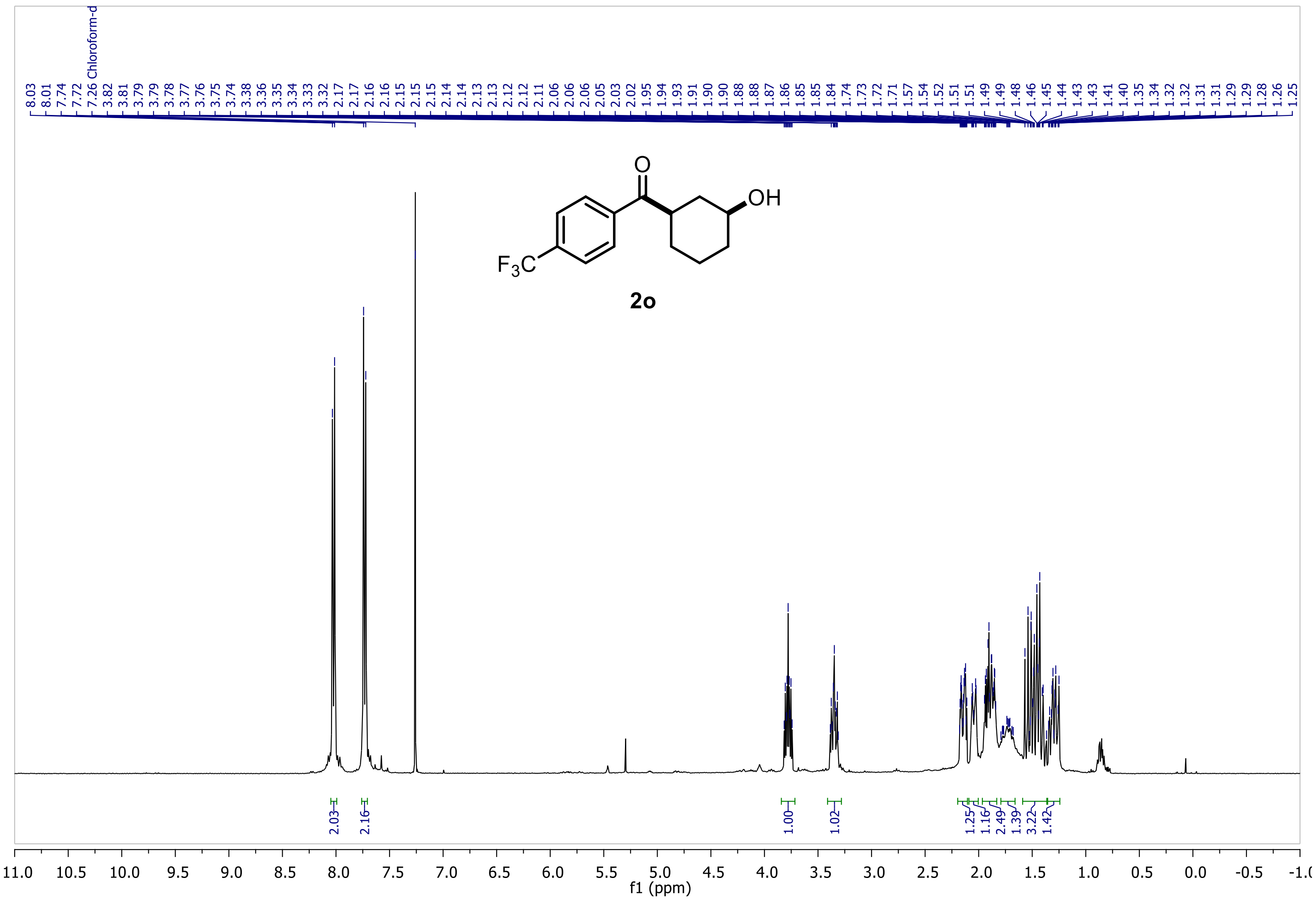

$^{13}C\{^{1}H\}$ NMR (101 MHz, $CDCl_3$)

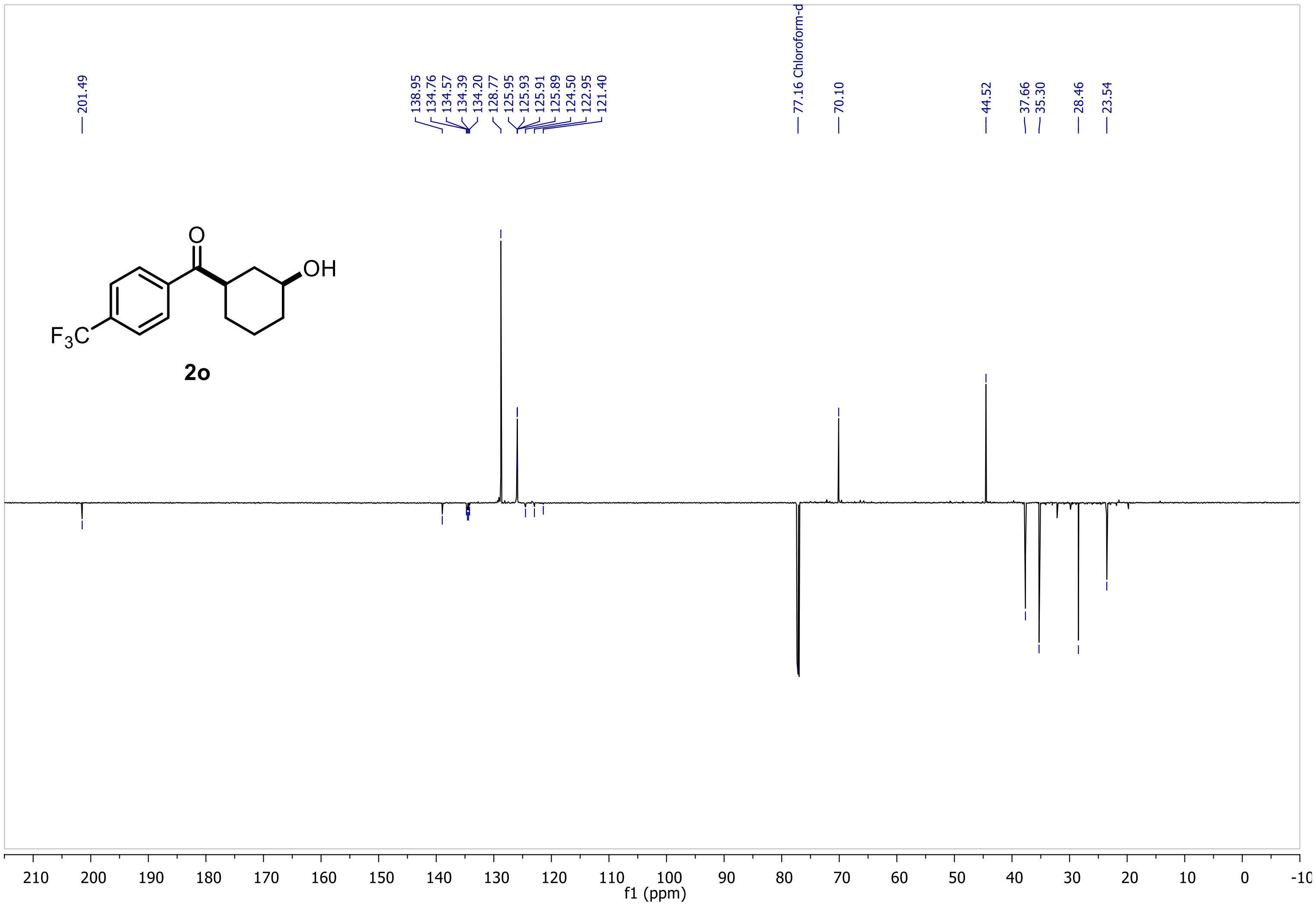

^{19}F NMR (376 MHz, $CDCl_3$)

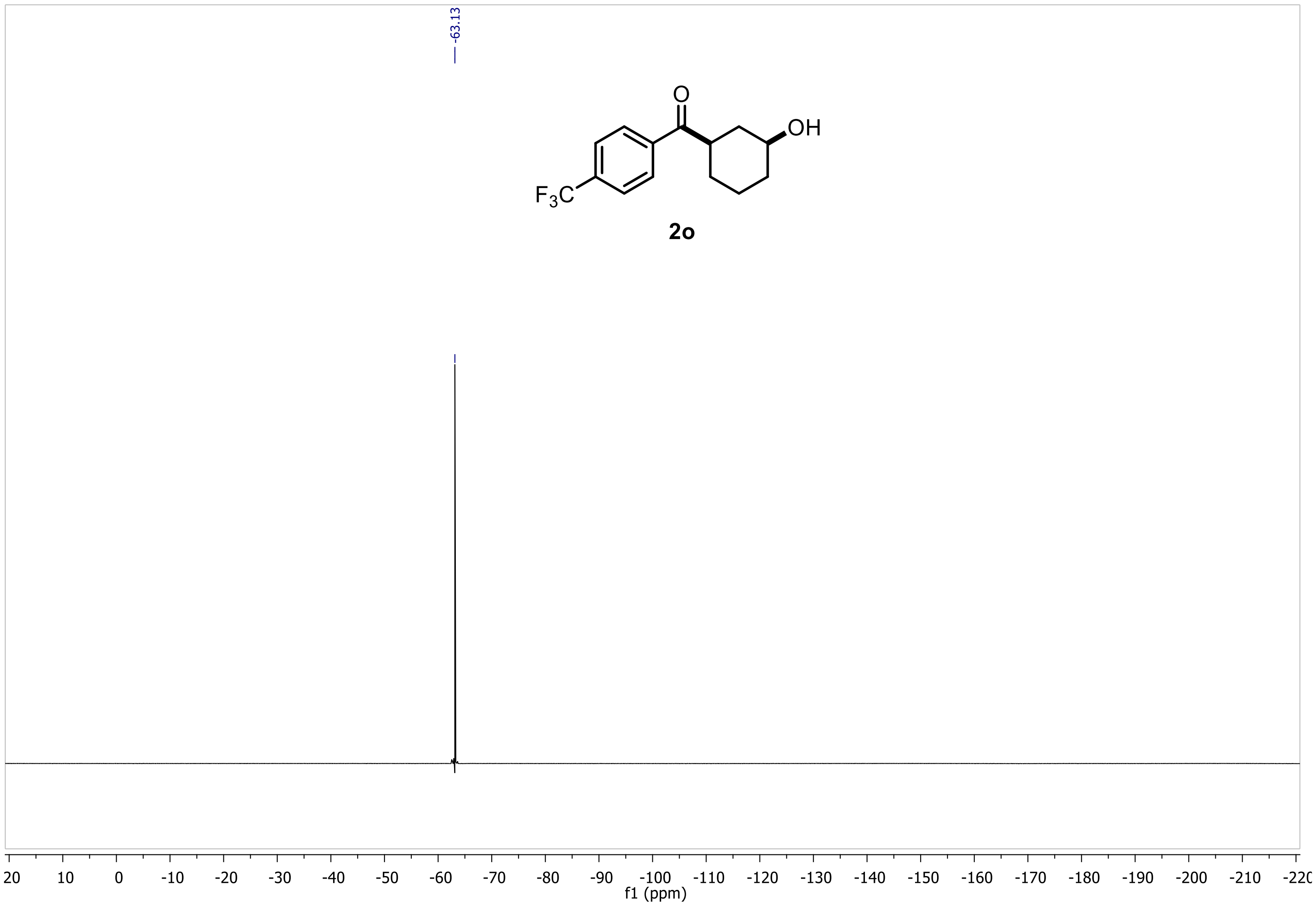

2p – cis-Benzo[*b*]thiophen-2-yl(3-hydroxycyclohexyl)methanone

^{1}H NMR (700 MHz, $CDCl_3$)

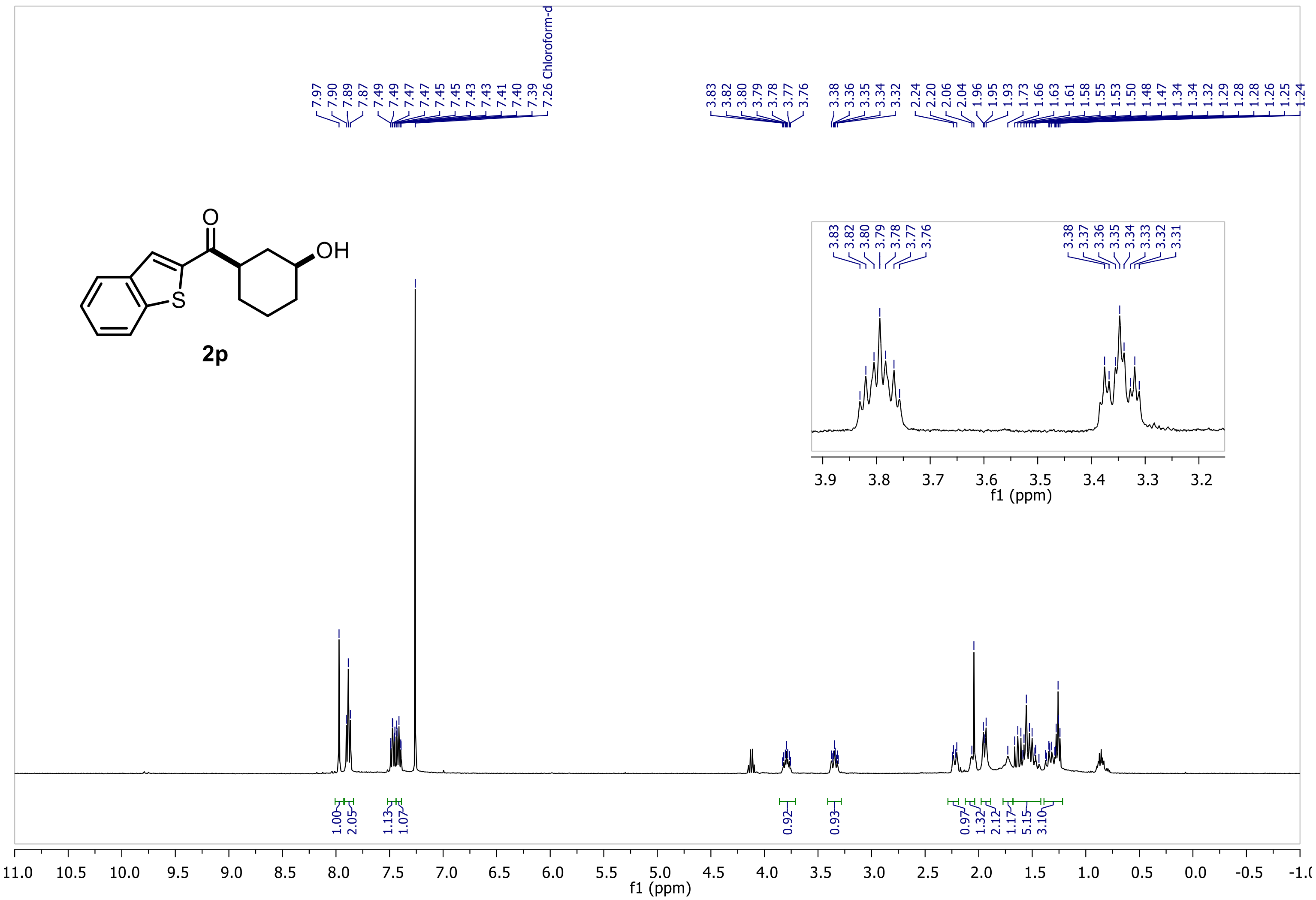

^{13}C (DEPT 135) NMR (176 MHz, $CDCl_3$)

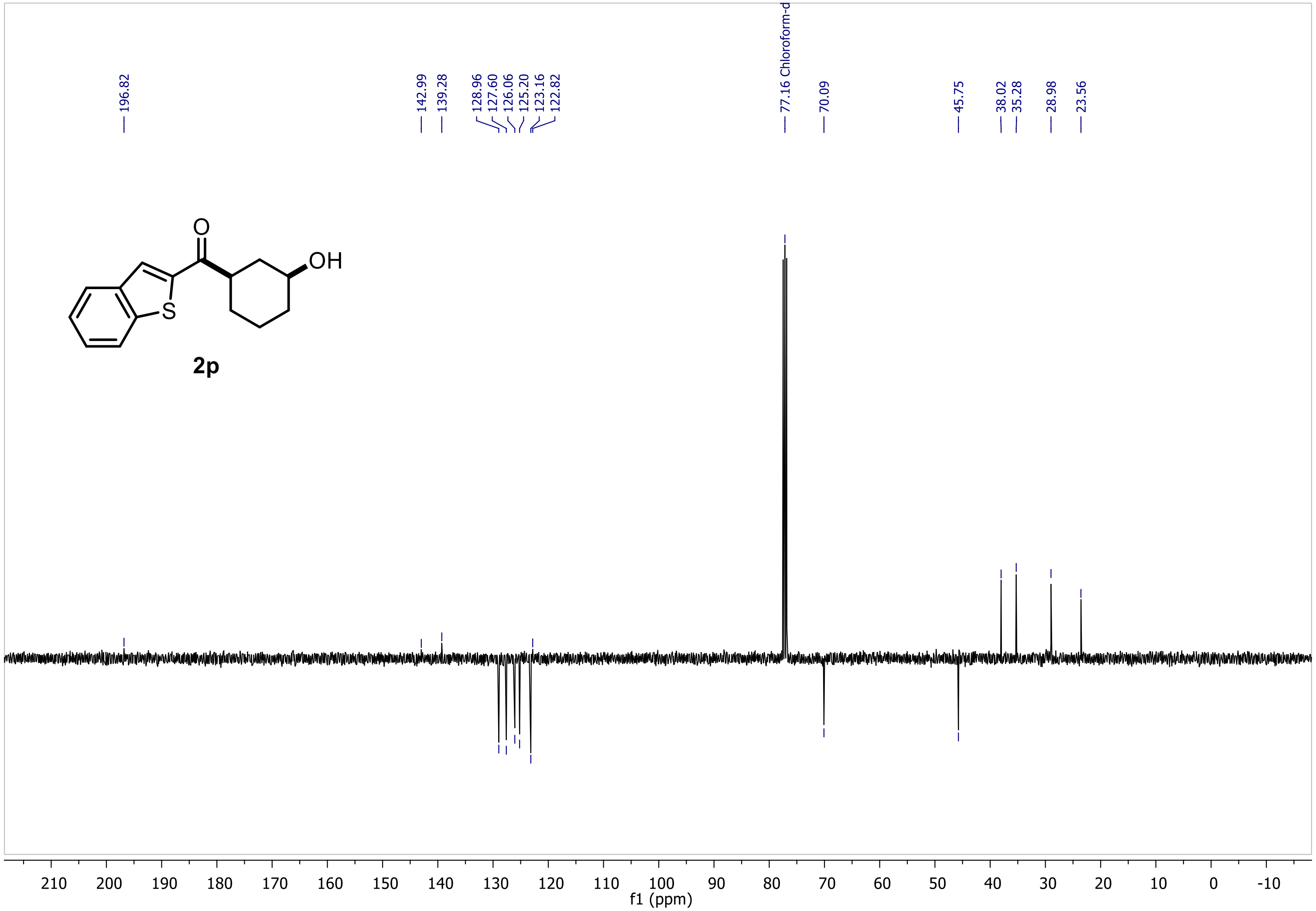

2q – *cis*-(3-(*tert*-Butyl)-3-hydroxycyclopentyl)[4-(trifluoromethyl)phenyl]methanone

^{1}H NMR (400 MHz, $CDCl_3$)

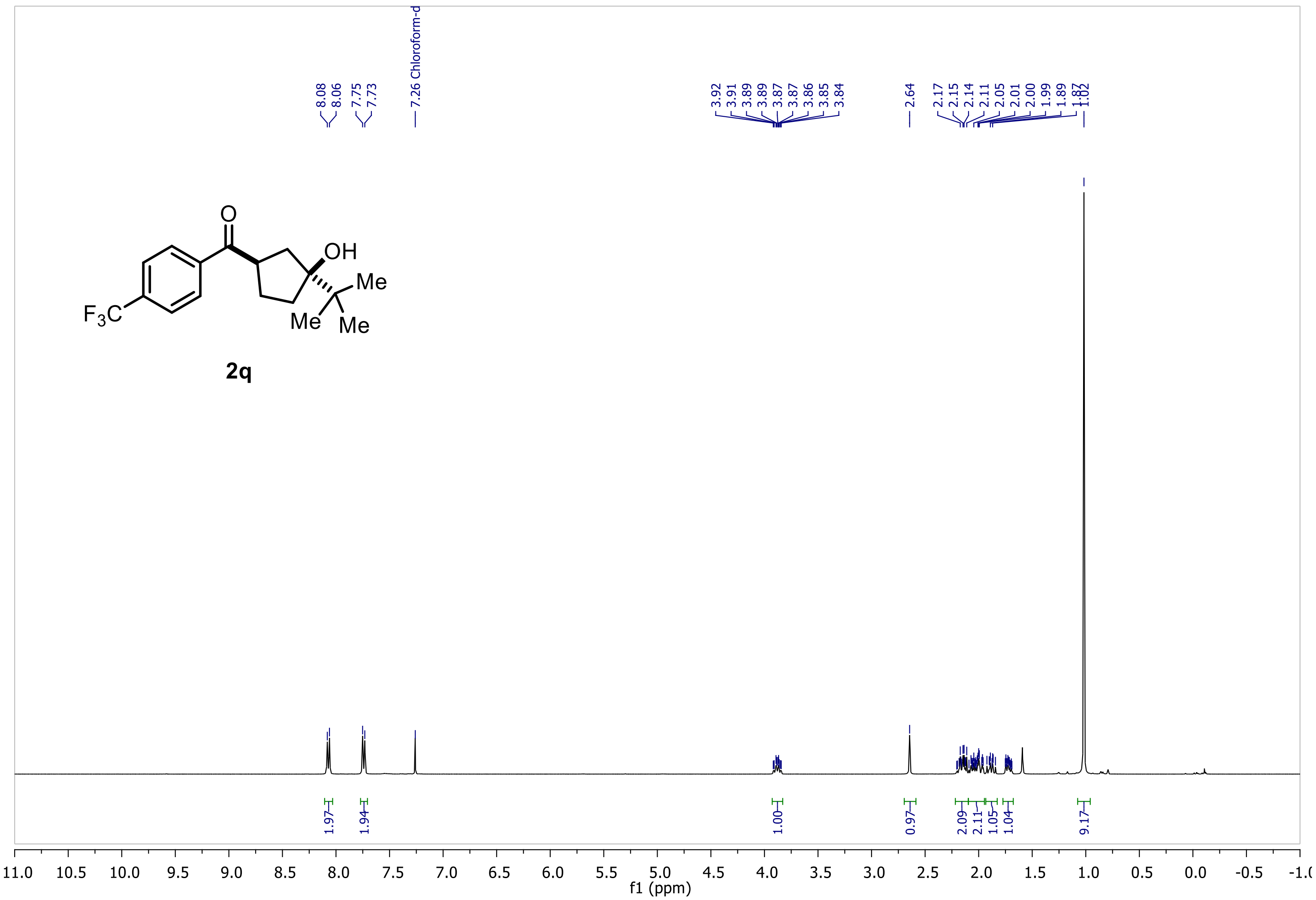

$^{13}C\{^{1}H\}$ NMR (101 MHz, $CDCl_3$)

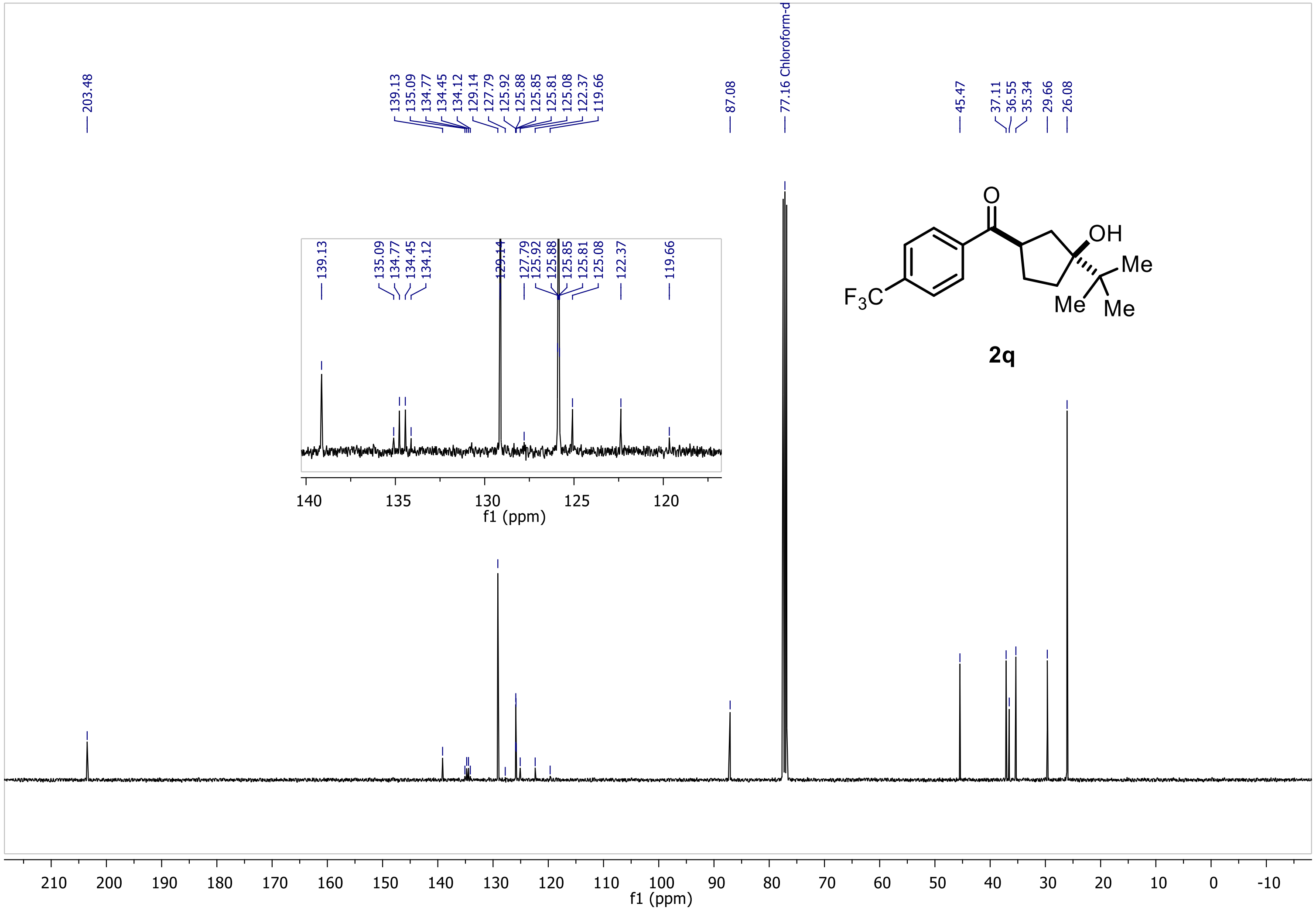

^{19}F NMR (376 MHz, $CDCl_3$)

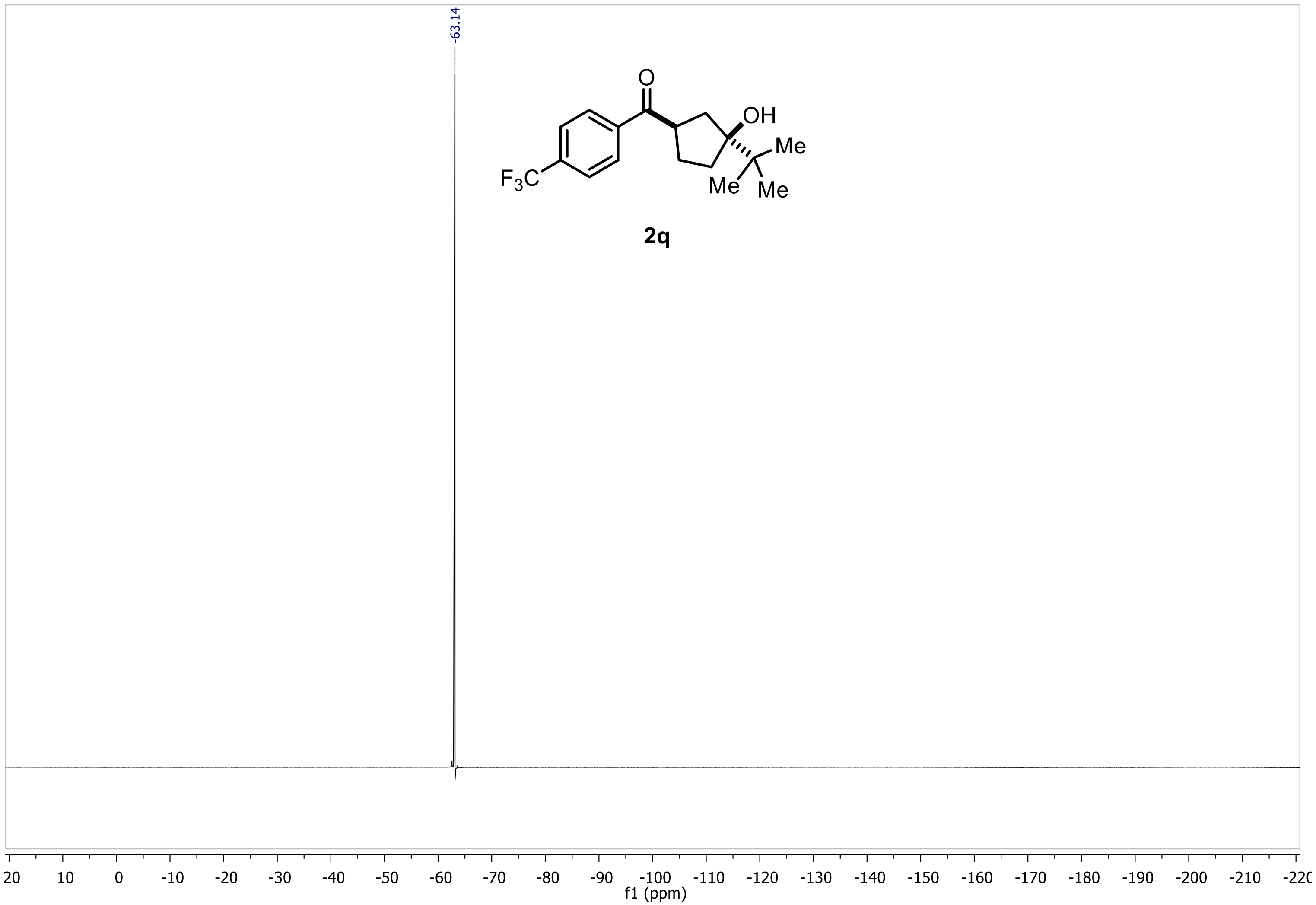

2r – *cis*-(3-Hydroxy-3-methylcyclopentyl)[4-(trifluoromethyl)phenyl]methanone

^{1}H NMR (400 MHz, $CDCl_3$)

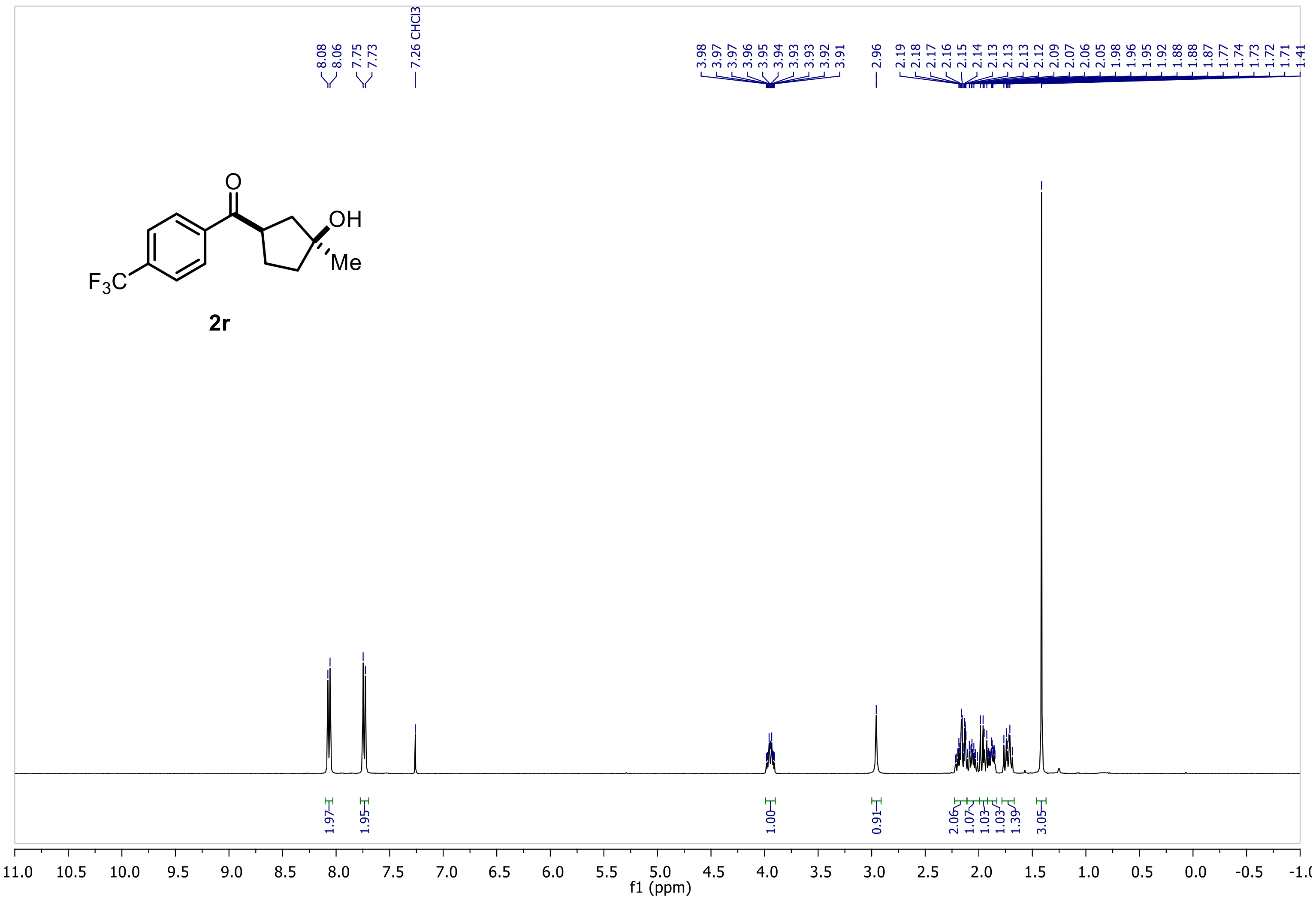

$^{13}C\{^1H\}$ NMR (101 MHz, $CDCl_3$)

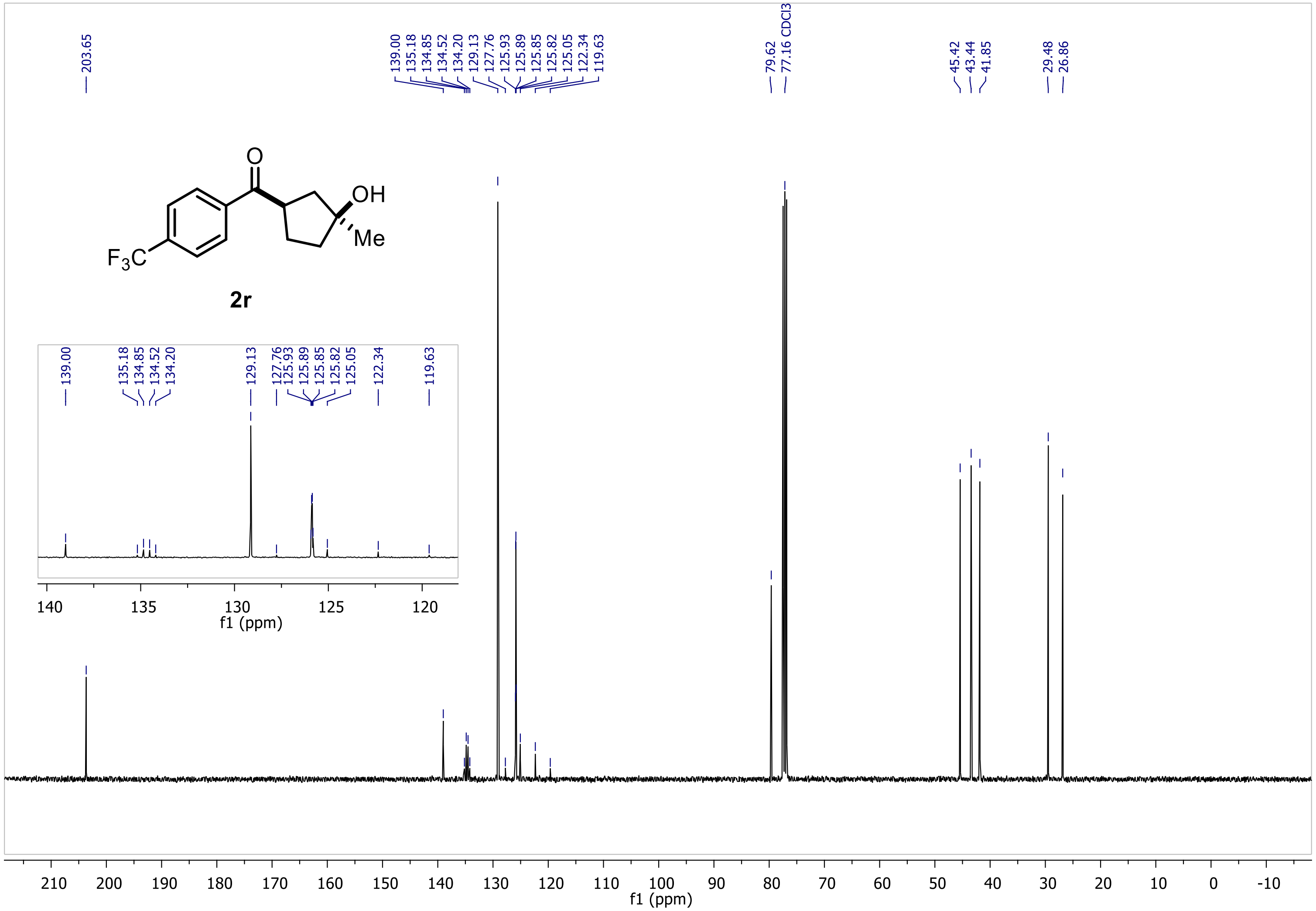

^{19}F NMR (376 MHz, $CDCl_3$)

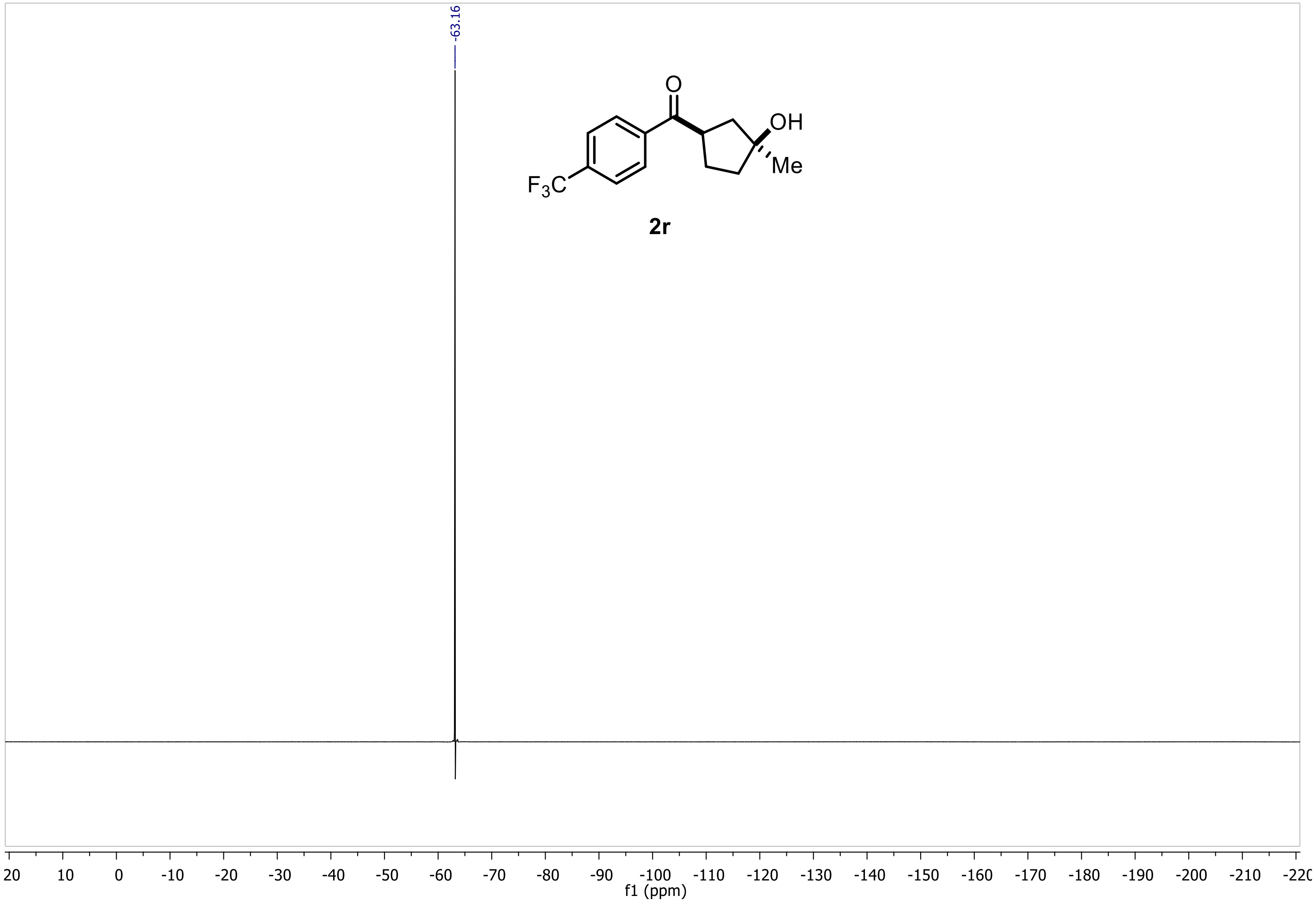

2s – *cis*-(3-Hydroxy-3-methylcyclopentyl)(phenyl)methanone

^{1}H NMR (600 MHz, $CDCl_3$)

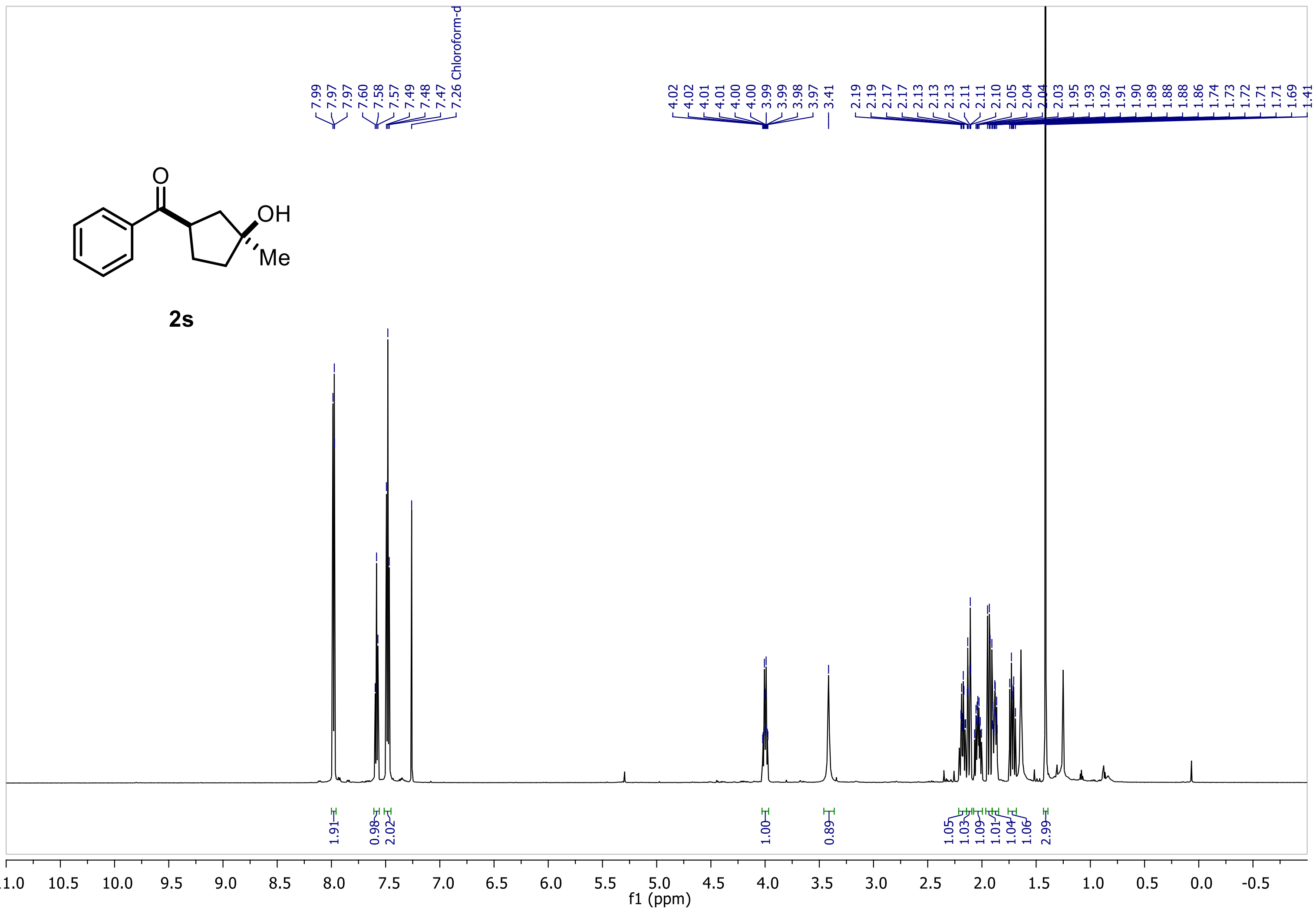

^{13}C (DEPT 135) NMR (151 MHz, $CDCl_3$)

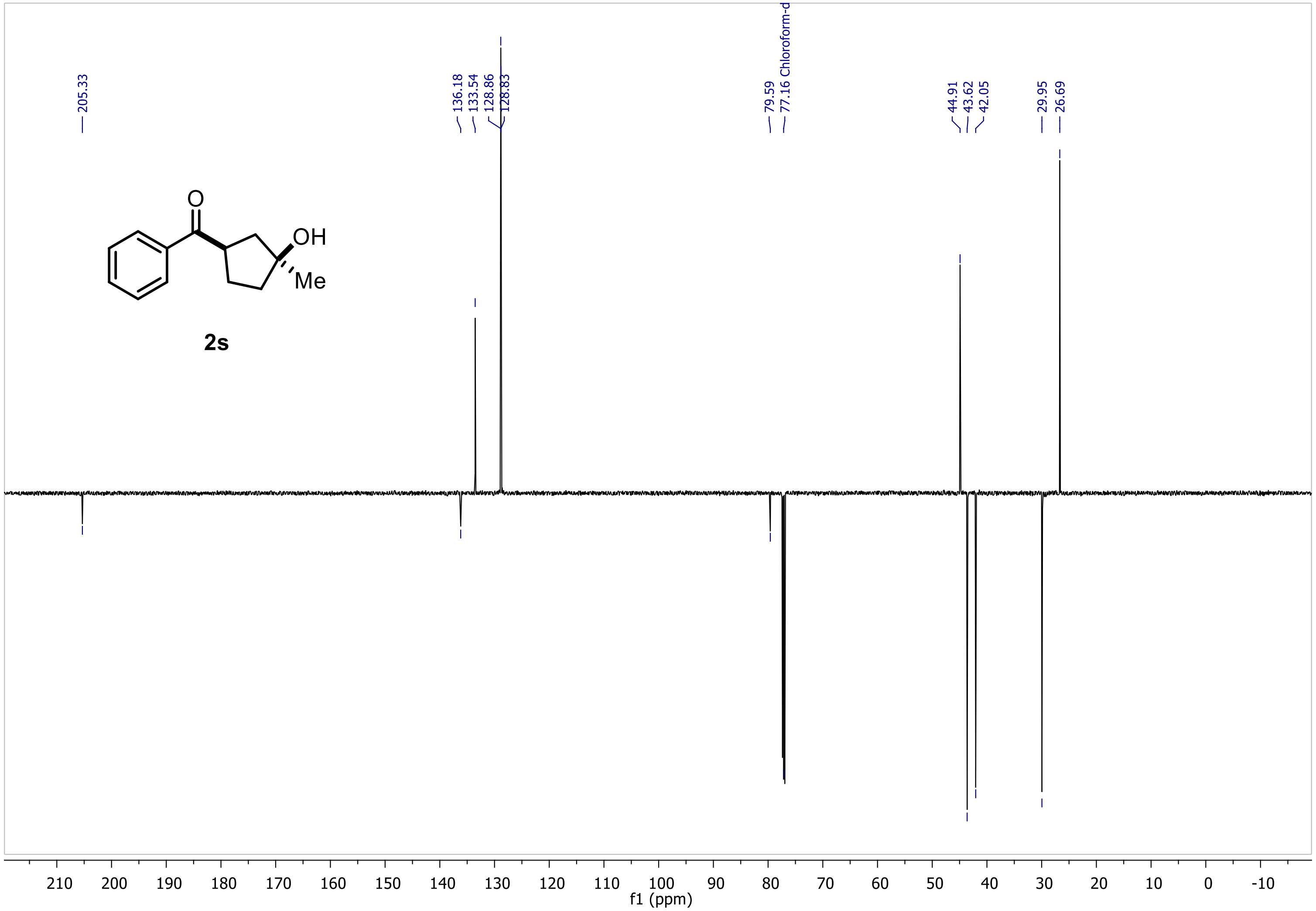

2s

2t – *cis*-(4-Butyl-4-hydroxycyclohexyl)[4-(trifluoromethyl)phenyl]methanone

^{1}H NMR (700 MHz, $CDCl_3$)

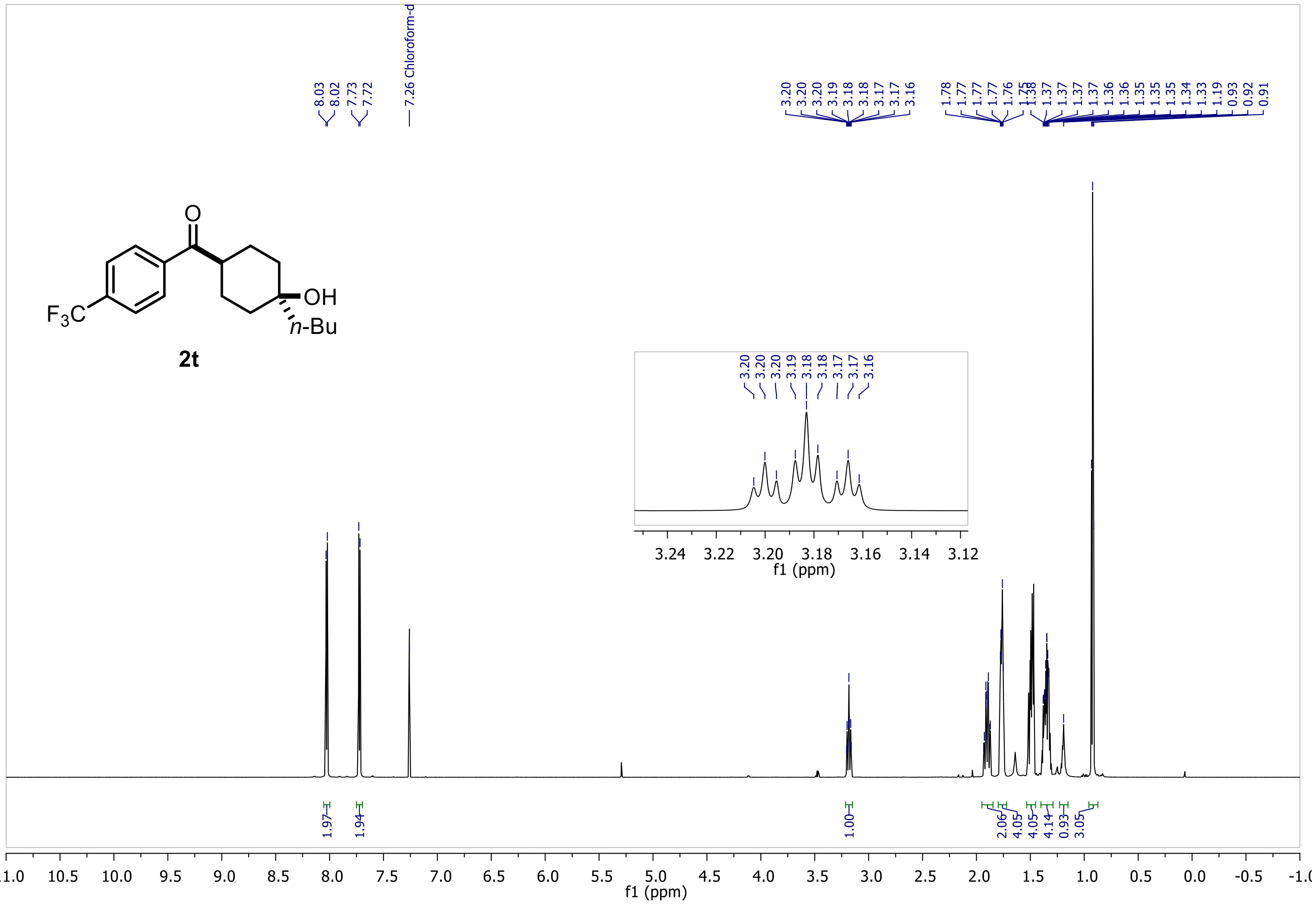

$^{13}C\{^{1}H\}$ NMR (176 MHz, $CDCl_3$)

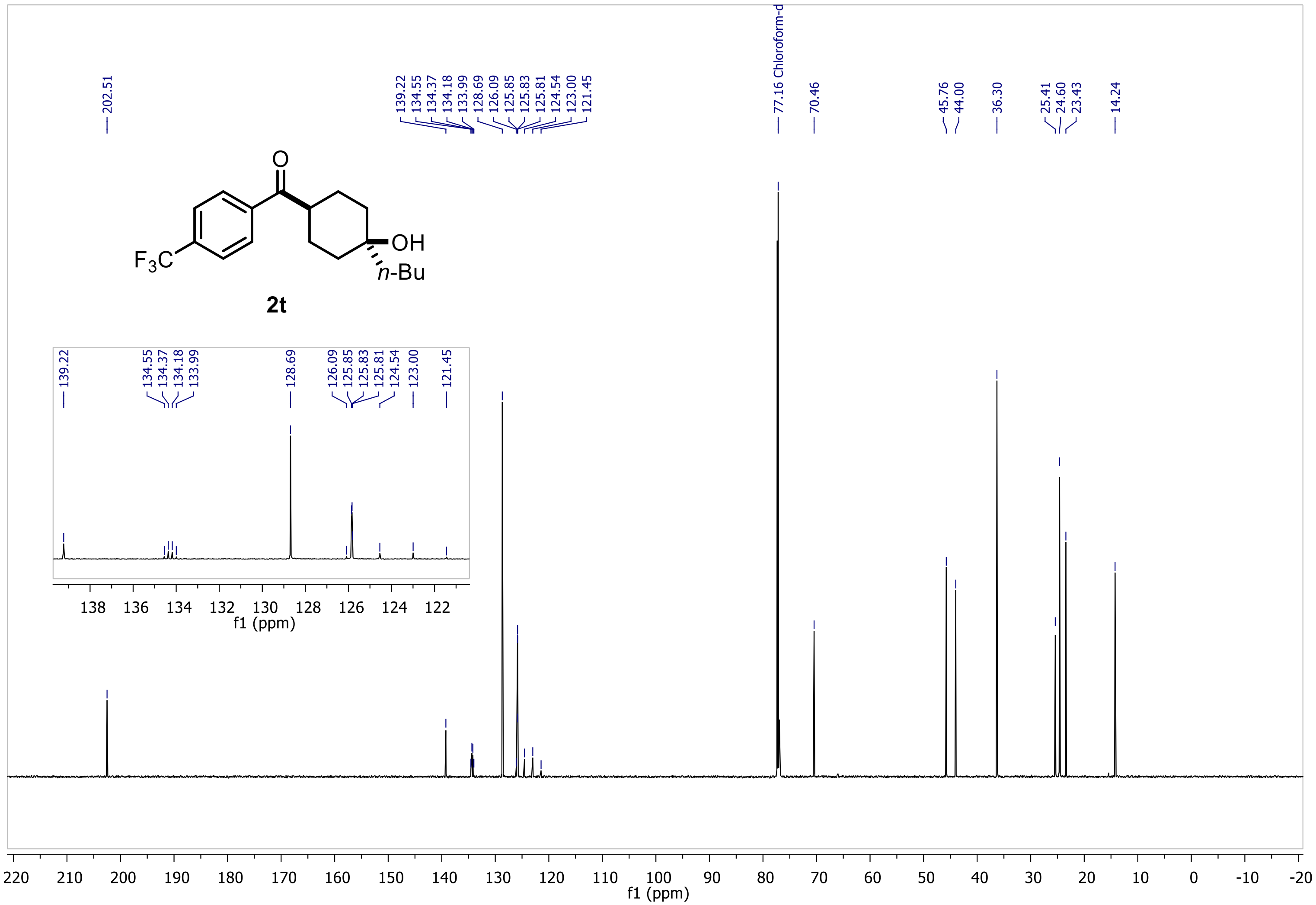

^{19}F NMR (659 MHz, $CDCl_3$)

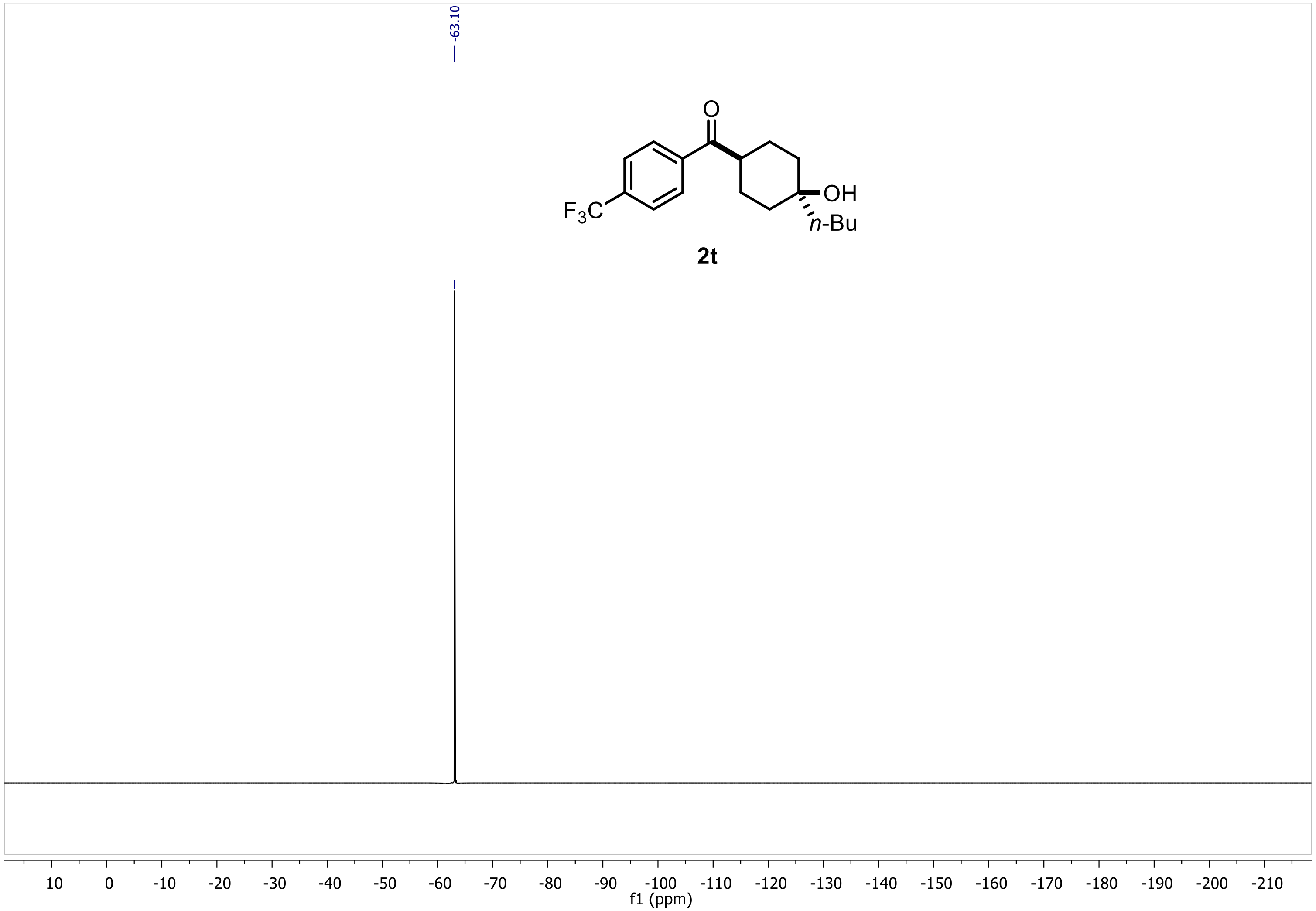

2u – *cis*-[4-(*tert*-Butyl)-4-hydroxycyclohexyl][4-(trifluoromethyl)phenyl]methanone

^{1}H NMR (600 MHz, $CDCl_3$)

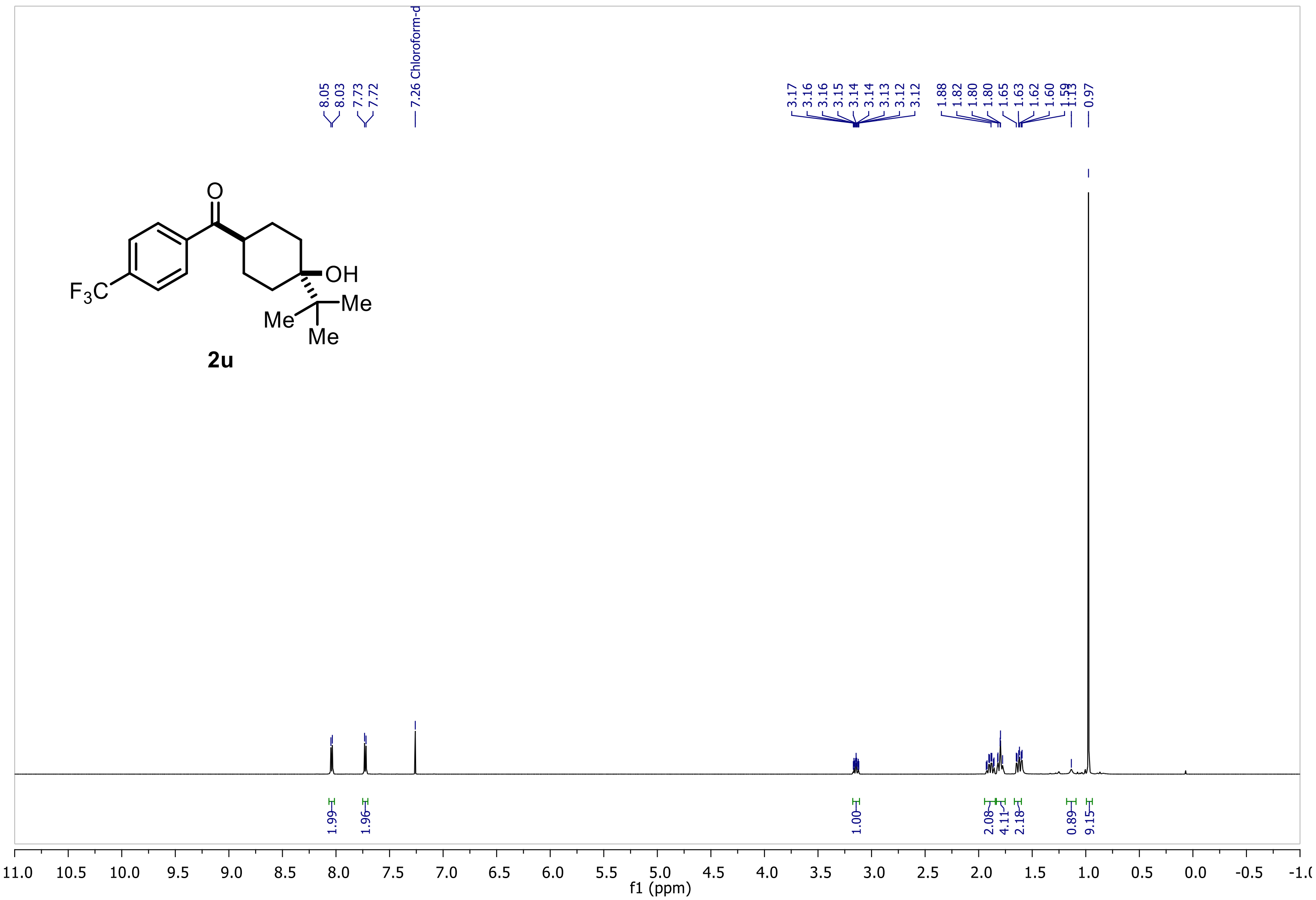

$^{13}C\{^1H\}$ NMR (151 MHz, $CDCl_3$)

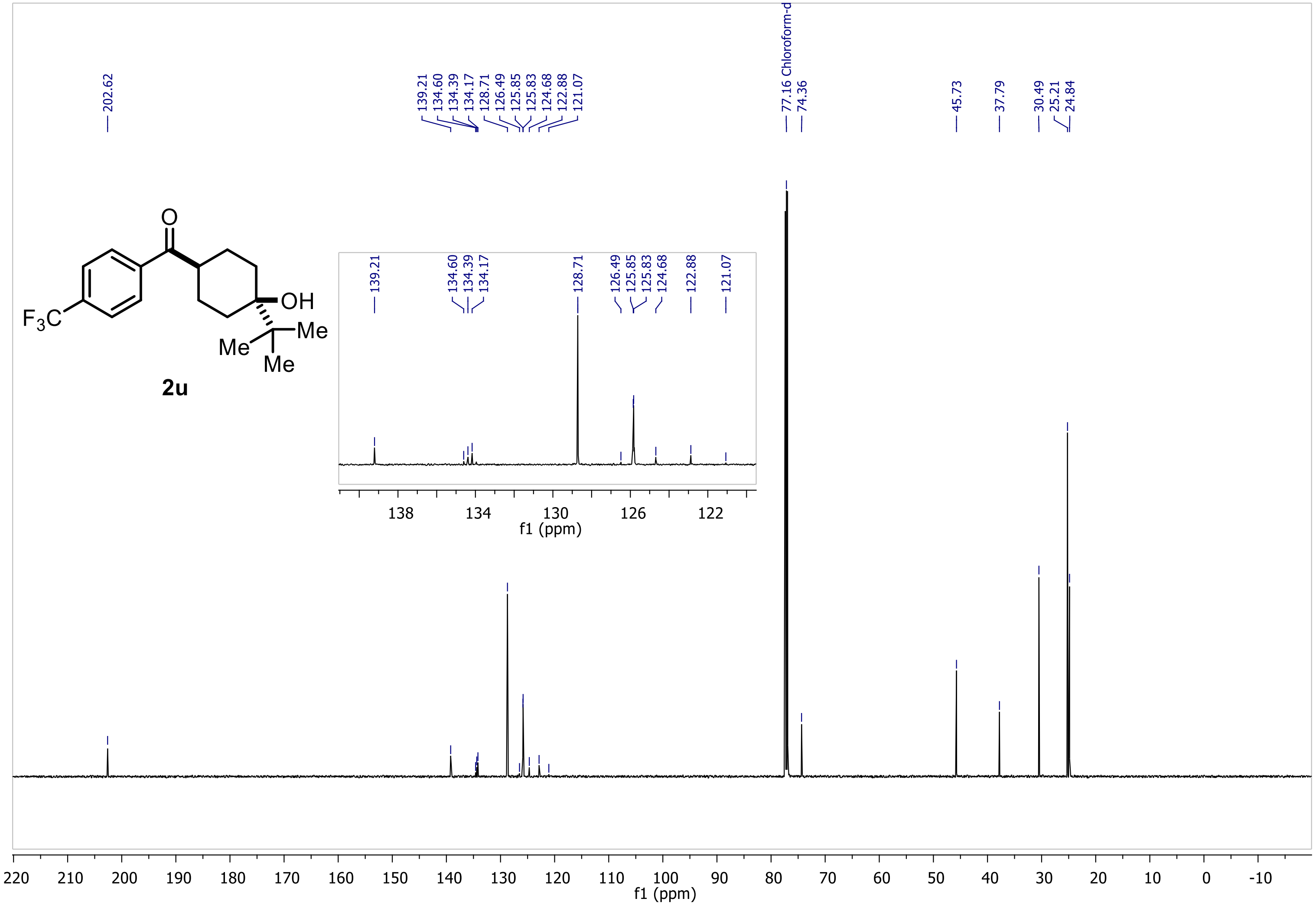

^{19}F NMR (565 MHz, $CDCl_3$)

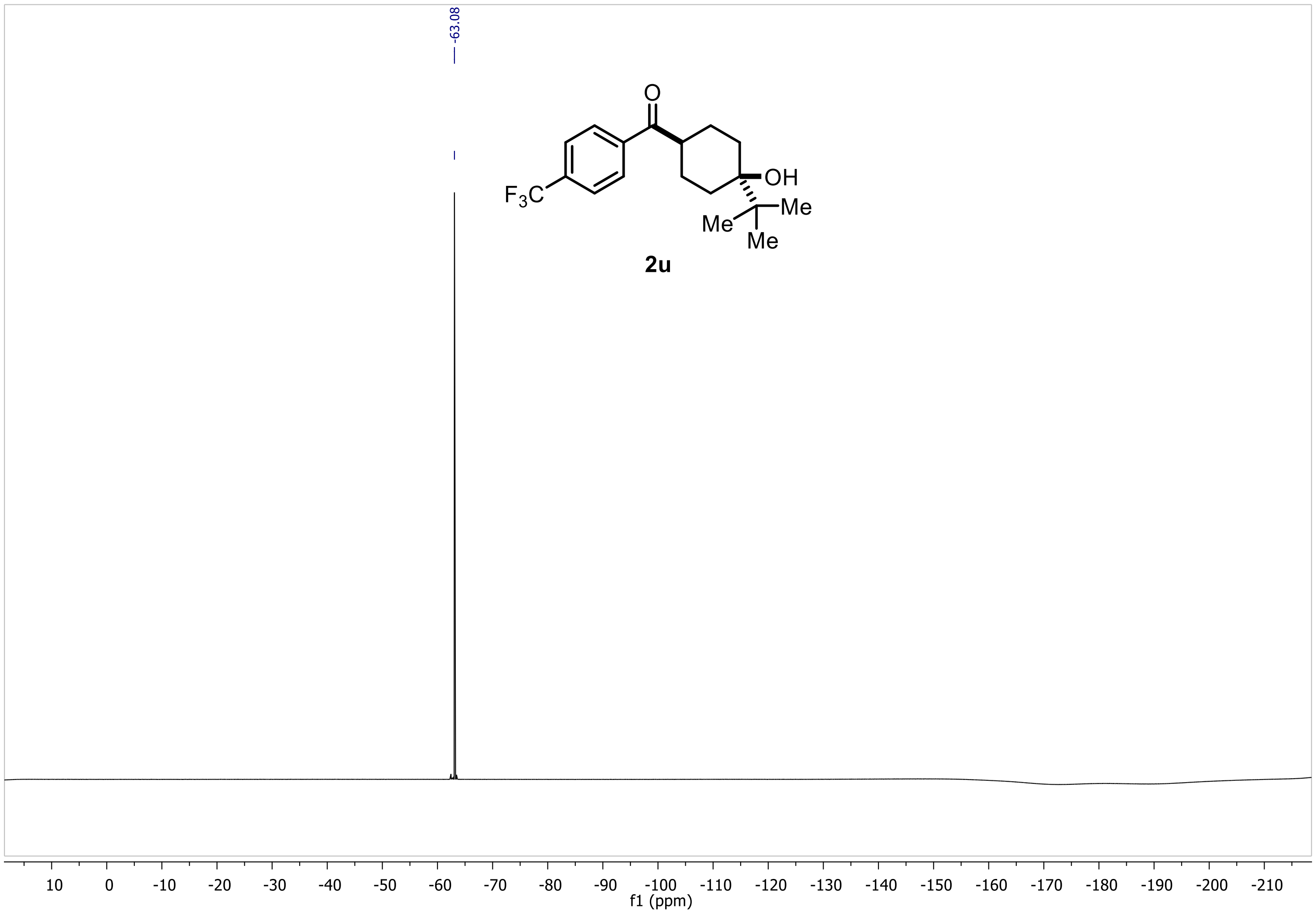

2v – *cis*-4-(*tert*-Butyl)-4-hydroxycyclohexyl)[3-(trifluoromethyl)phenyl]methanone

^{1}H NMR (700 MHz, $CDCl_3$)

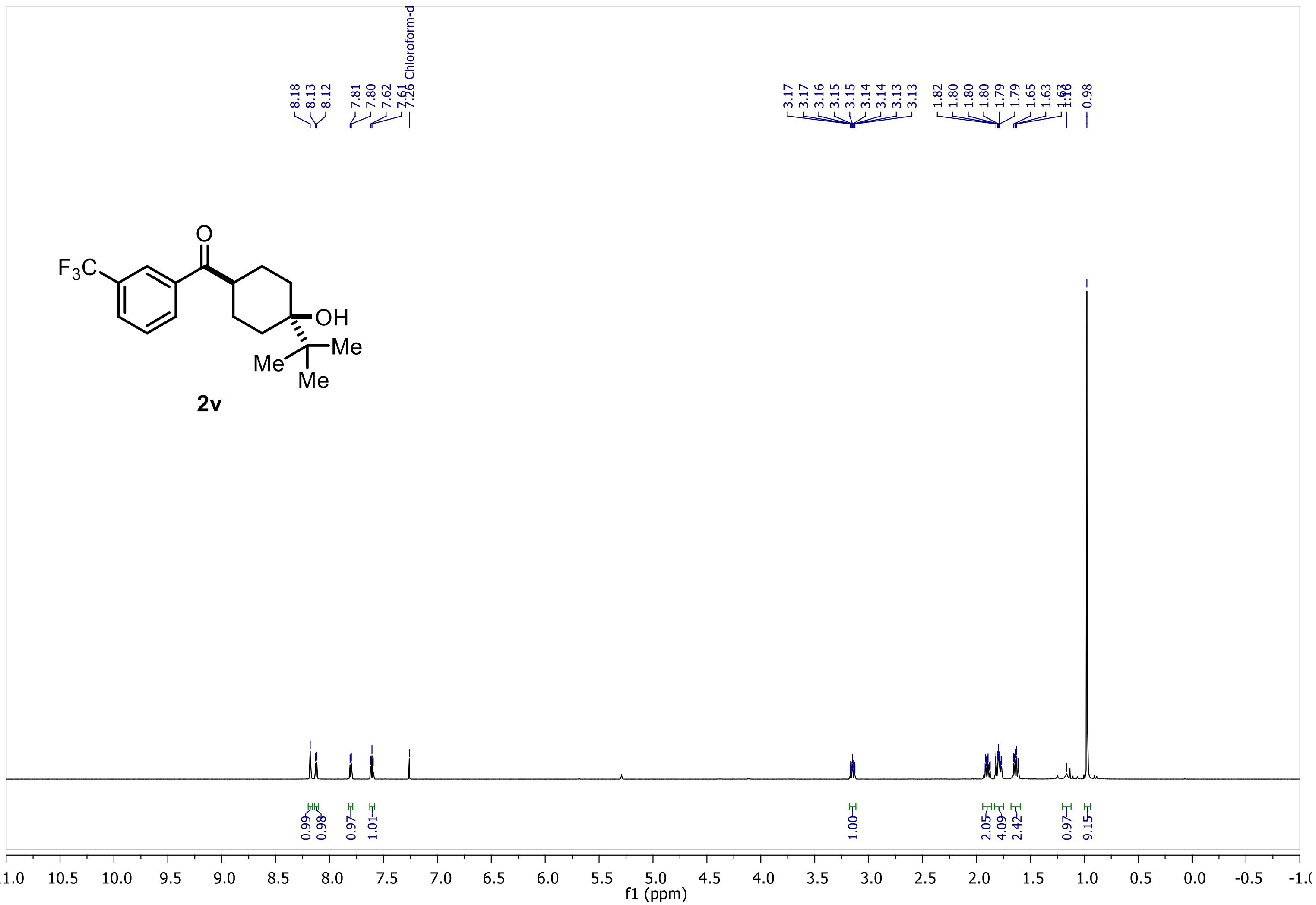

$^{13}C\{^1H\}$ NMR (176 MHz, $CDCl_3$)

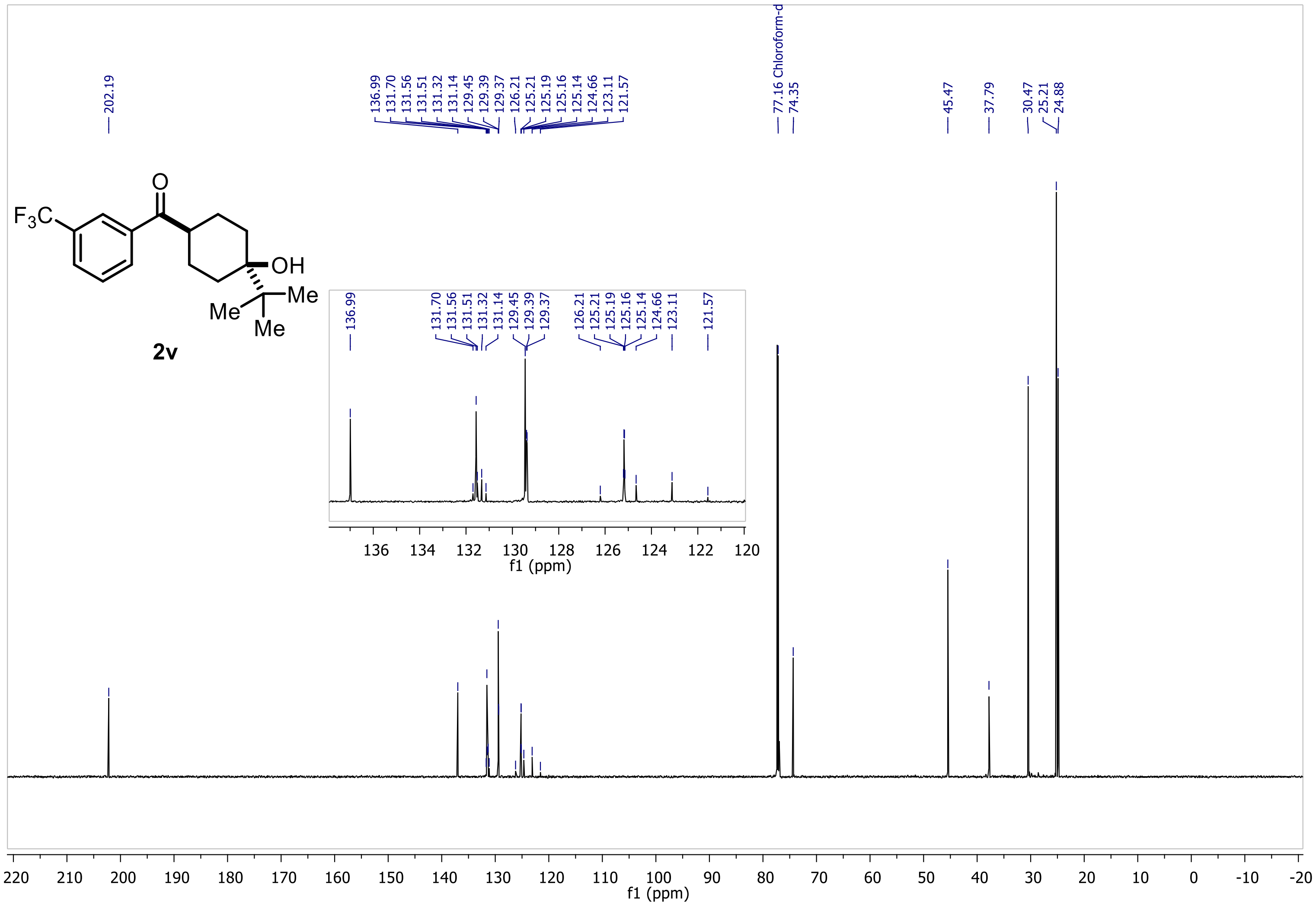

^{19}F NMR (659 MHz, CDCl$_3$)

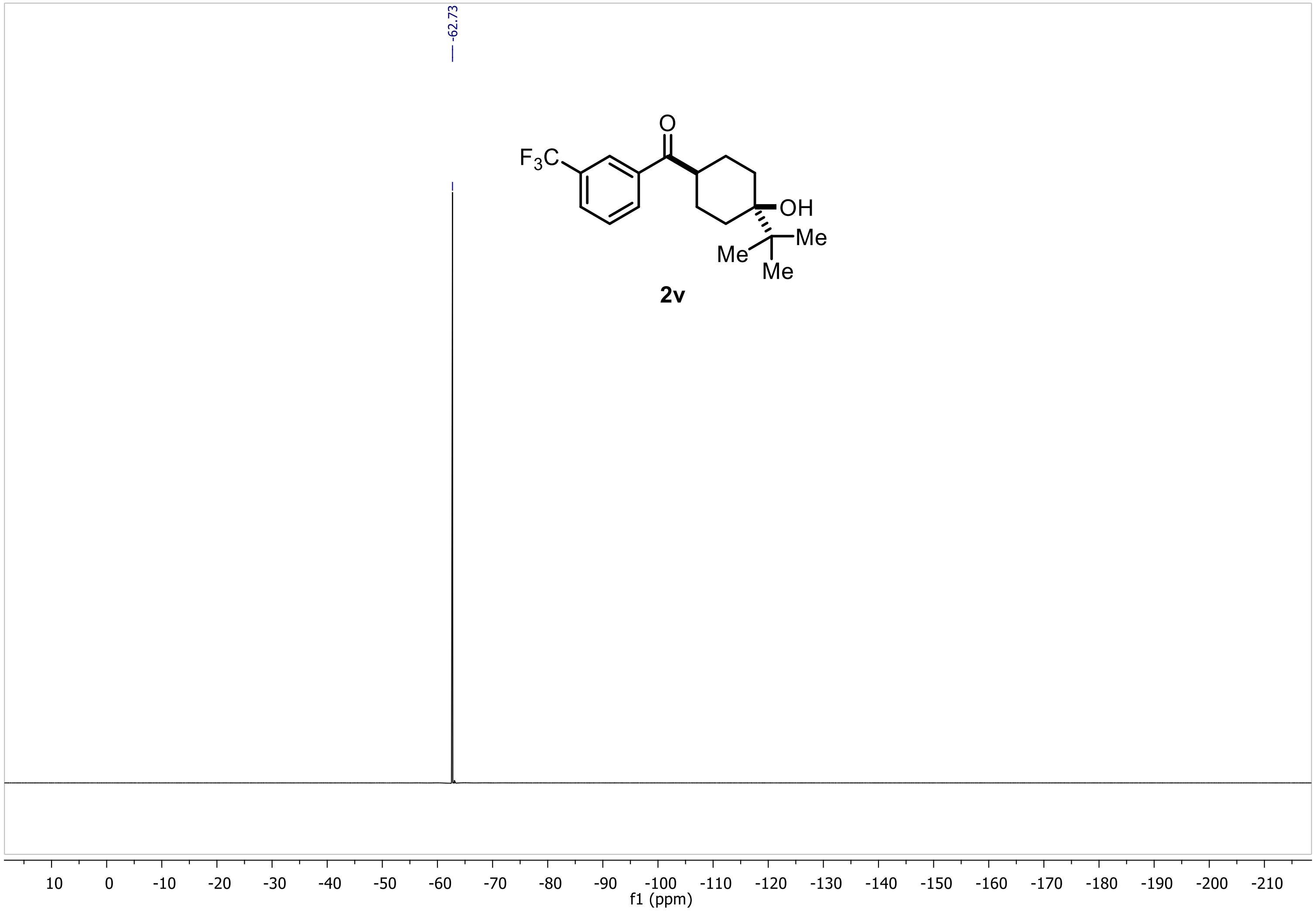

2v

2w – *cis*-(4-Hydroxy-4-methylcyclohexyl)[4-(trifluoromethyl)phenyl]methanone

^{1}H NMR (700 MHz, $CDCl_3$)

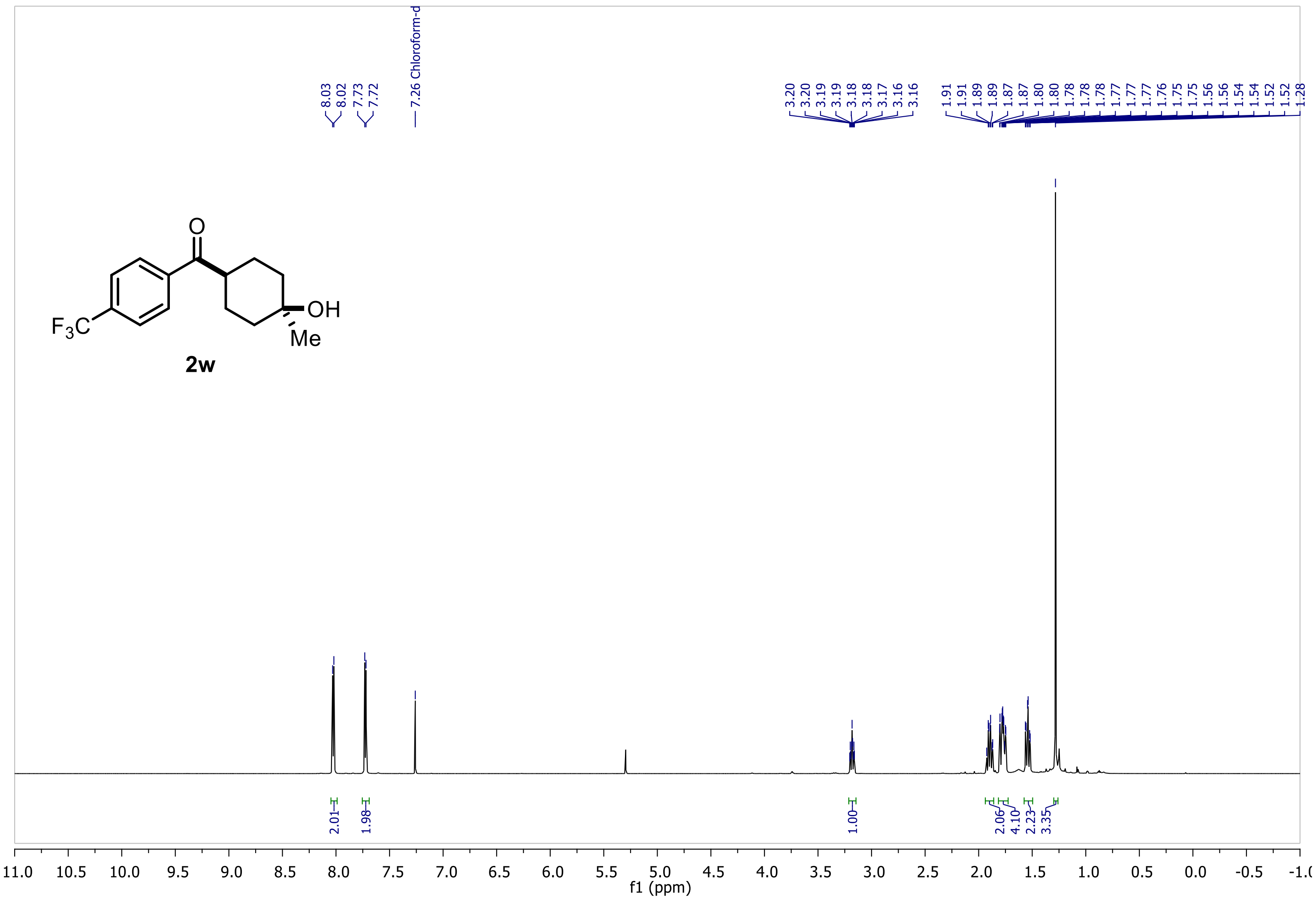

$^{13}C\{^1H\}$ NMR (176 MHz, $CDCl_3$)

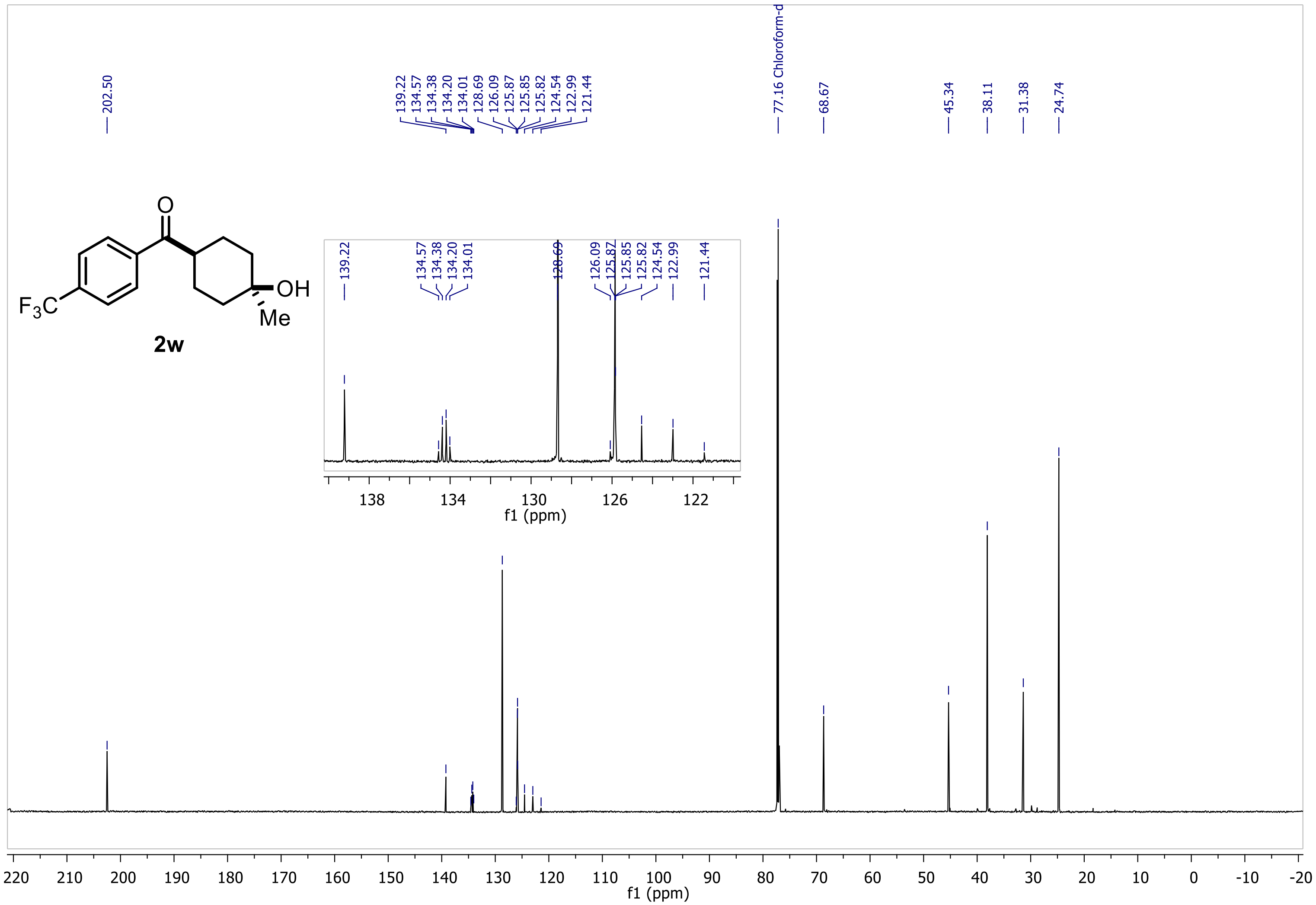

^{19}F NMR (659 MHz, $CDCl_3$)

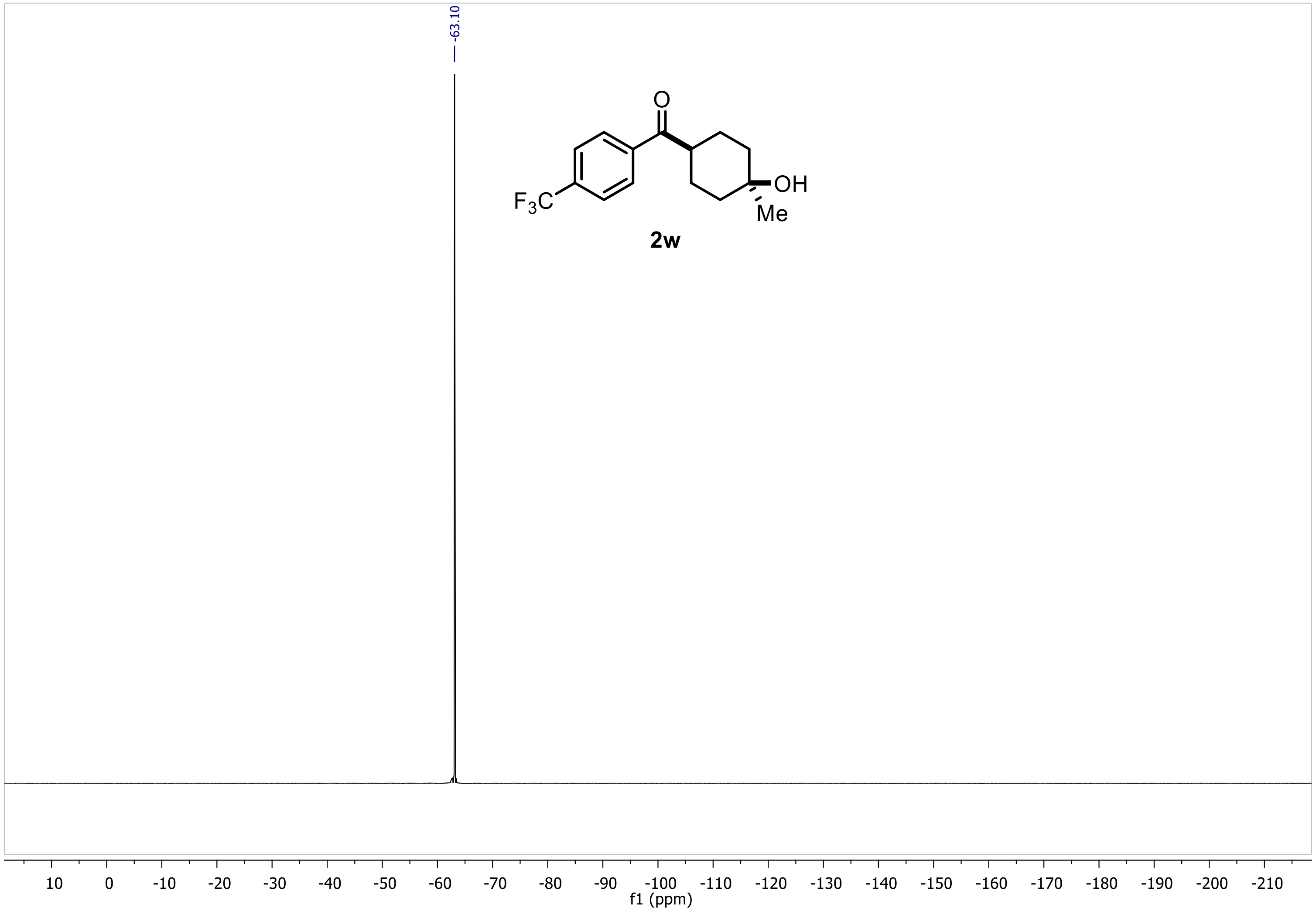

S1 – 1-(Methoxymethylene)-3-methylcyclopentane

^{1}H NMR (600 MHz, $CDCl_3$)

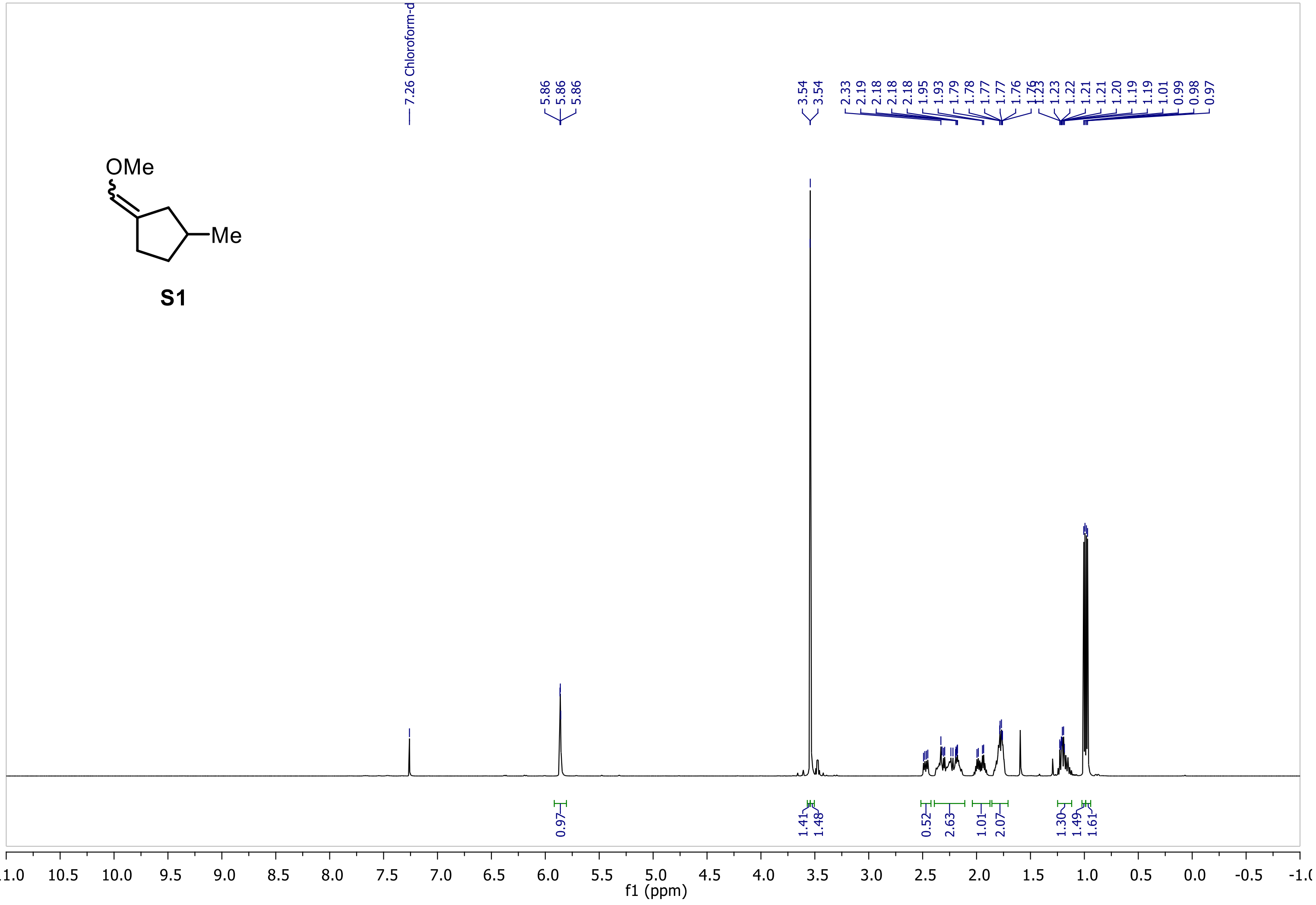

$^{13}C\{^1H\}$ NMR (151 MHz, $CDCl_3$)

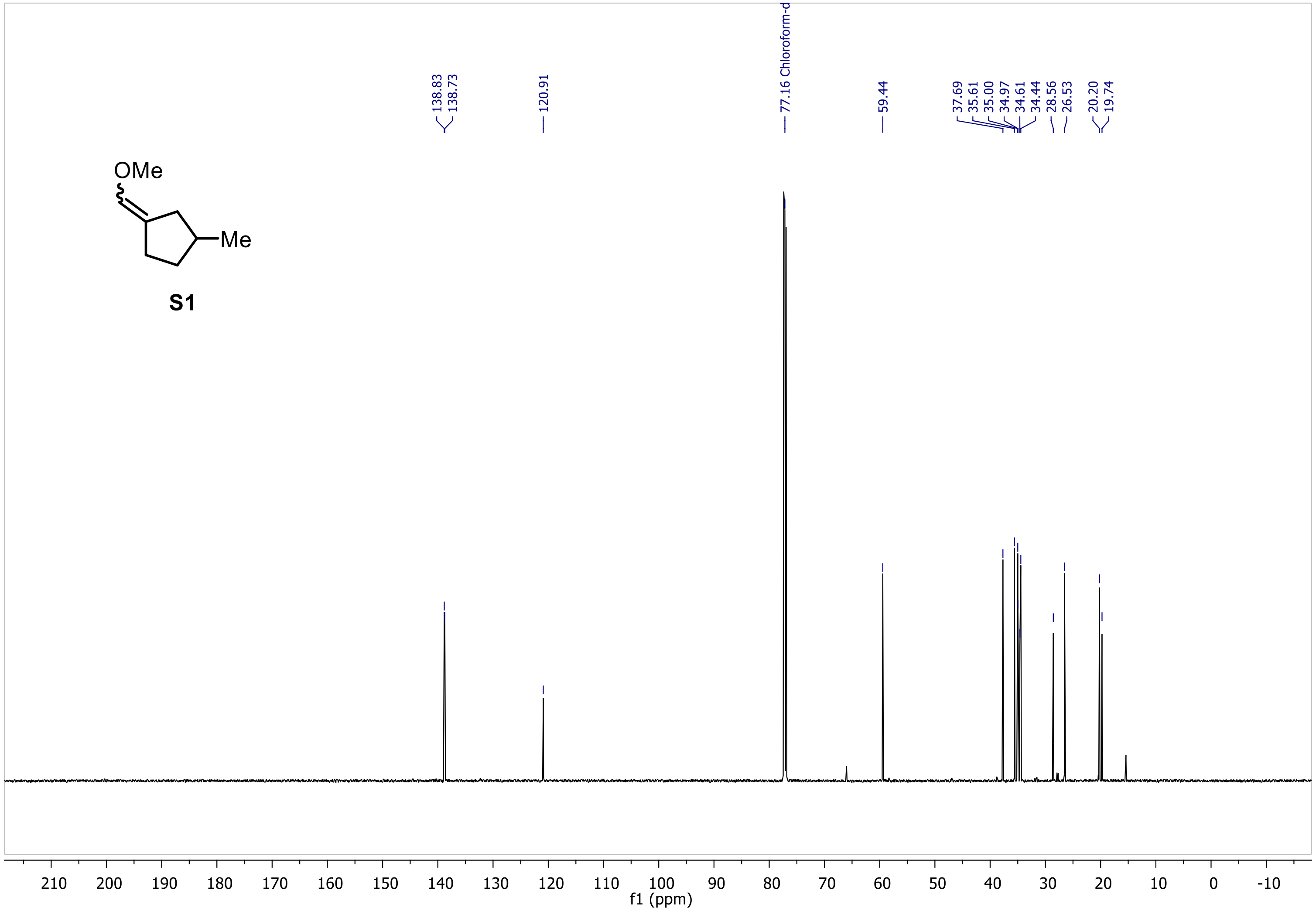

S2 – 3-Methylcyclopentane-1-carbaldehyde

^{1}H NMR (600 MHz, $CDCl_3$)

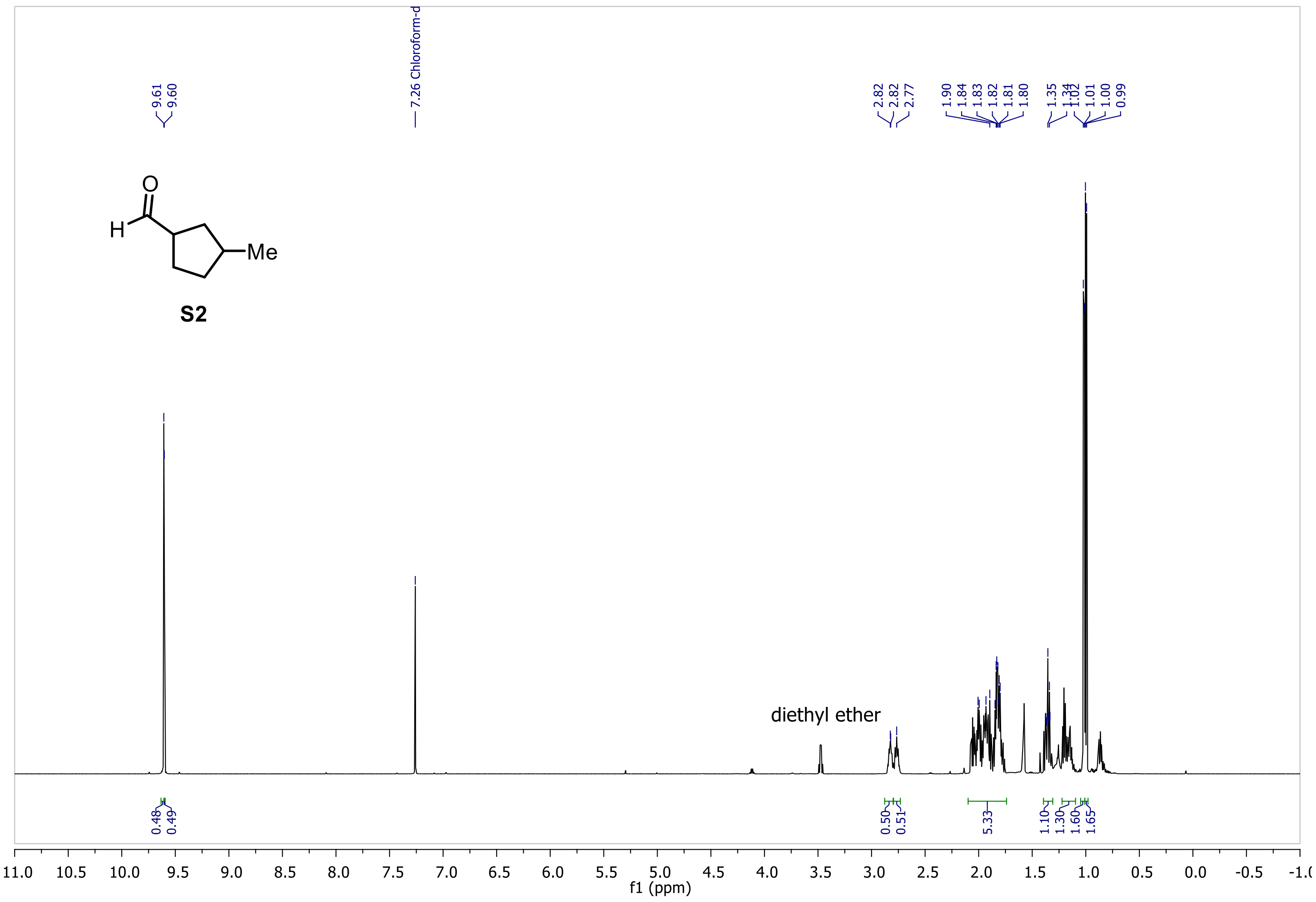

$^{13}C\{^{1}H\}$ NMR (151 MHz, $CDCl_3$)

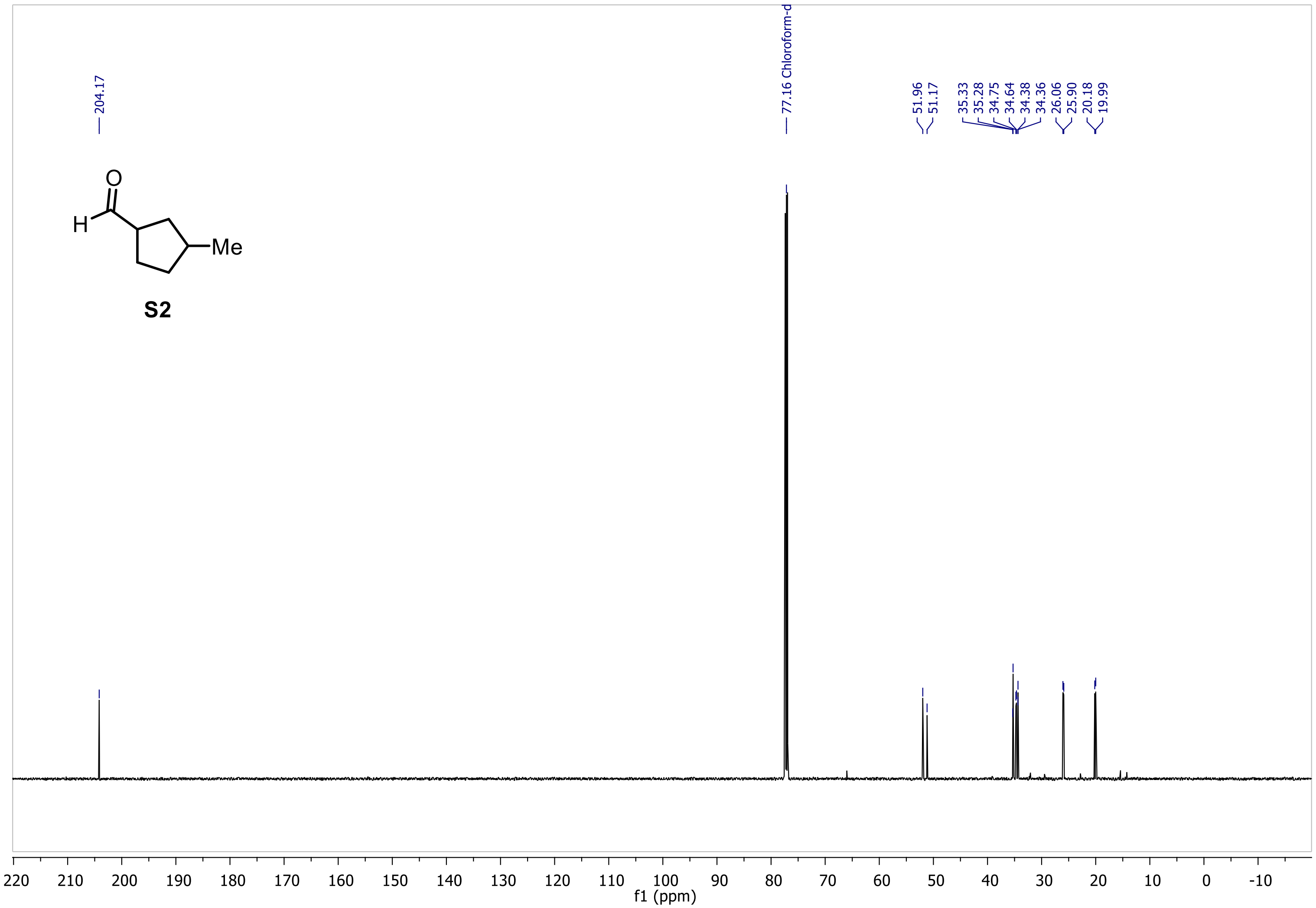

S3 – Cyclopentan-1-one-2,2,5,5-d_4

^{1}H NMR (400 MHz, $CDCl_3$):

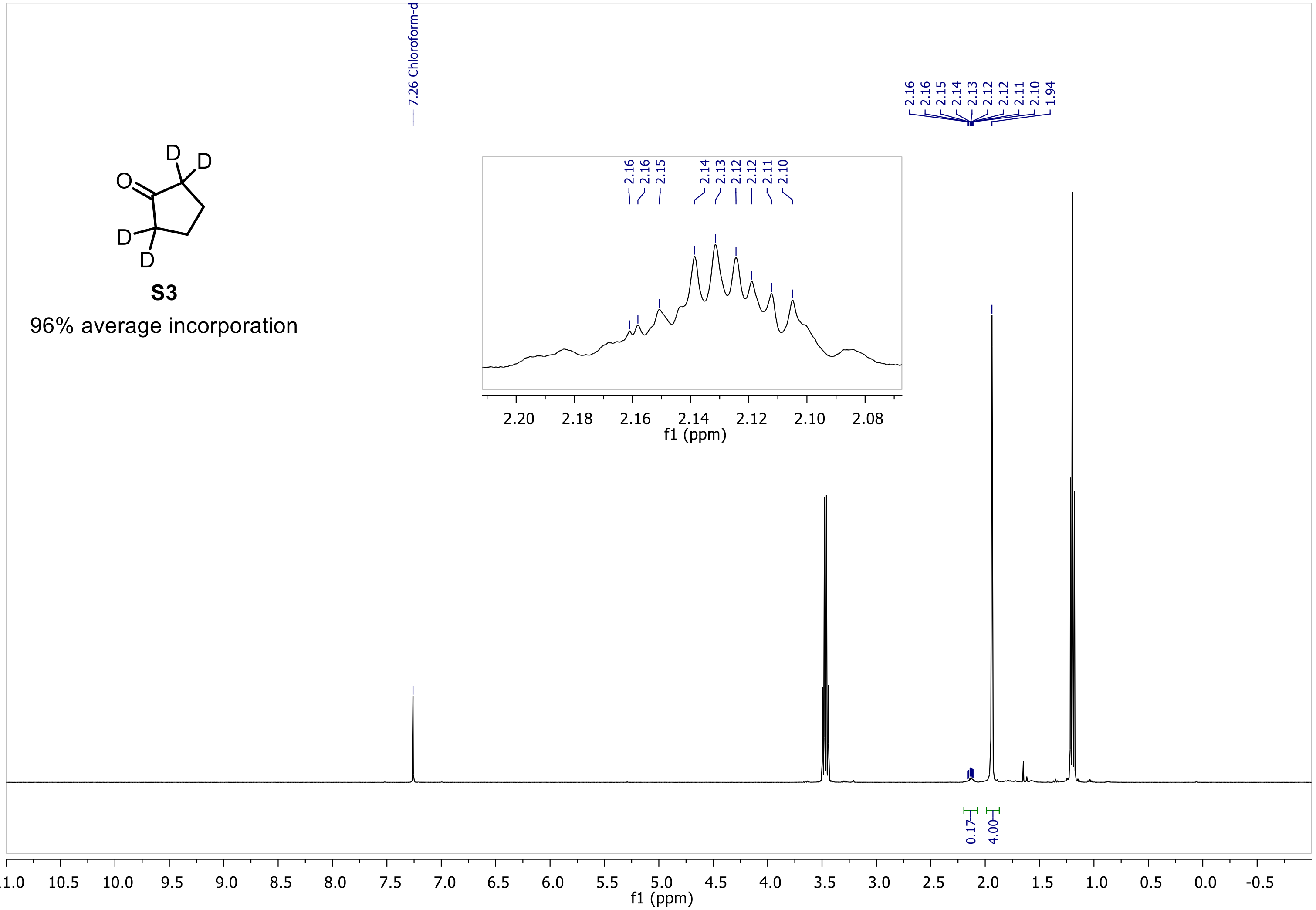

^{1}H NMR (400 MHz, $CDCl_3$): Comparison with non-deuterated product

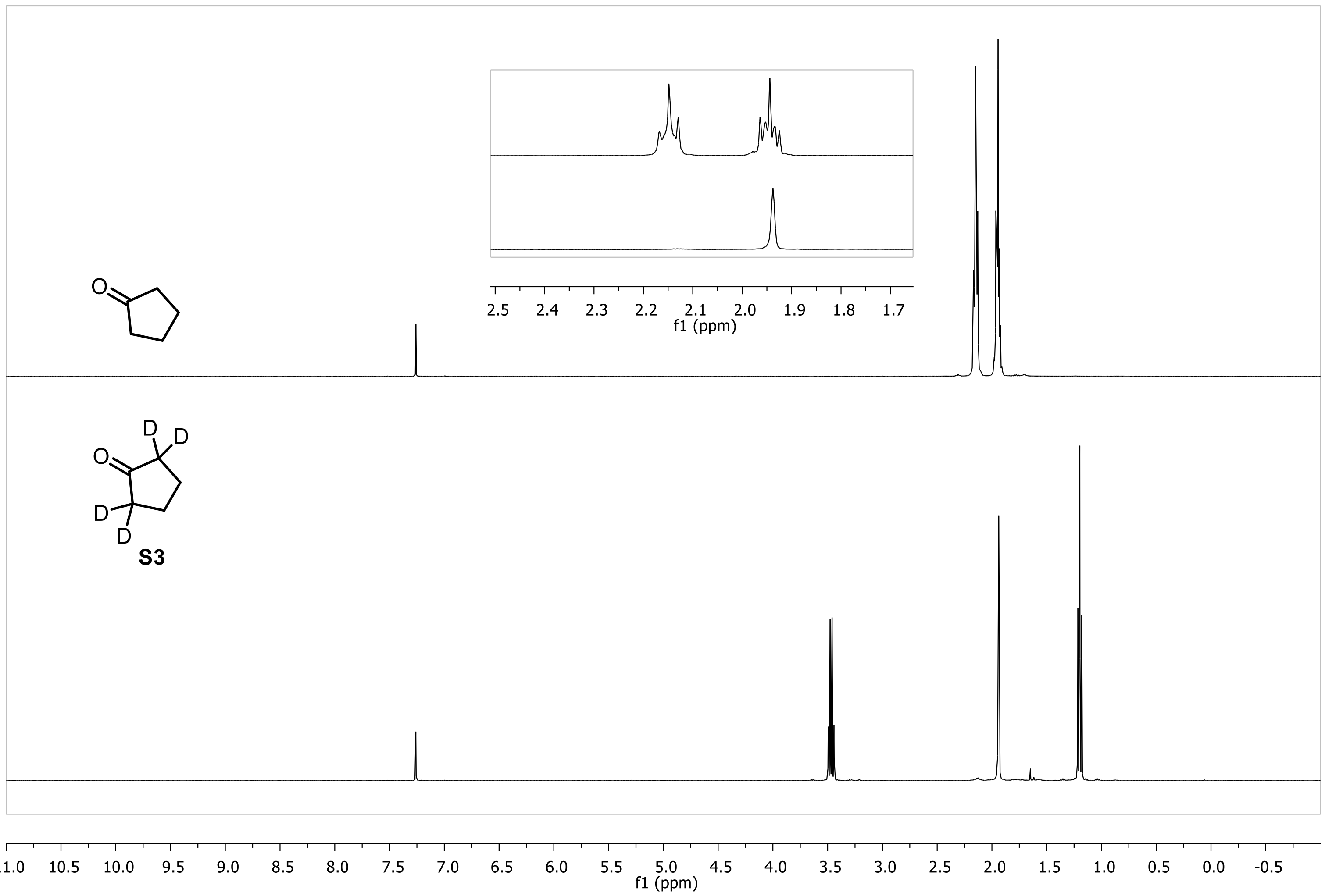

S4 – 3-Methylcyclopentan-1-one-2,2,5,5-d_4

^{1}H NMR (400 MHz, $CDCl_3$):

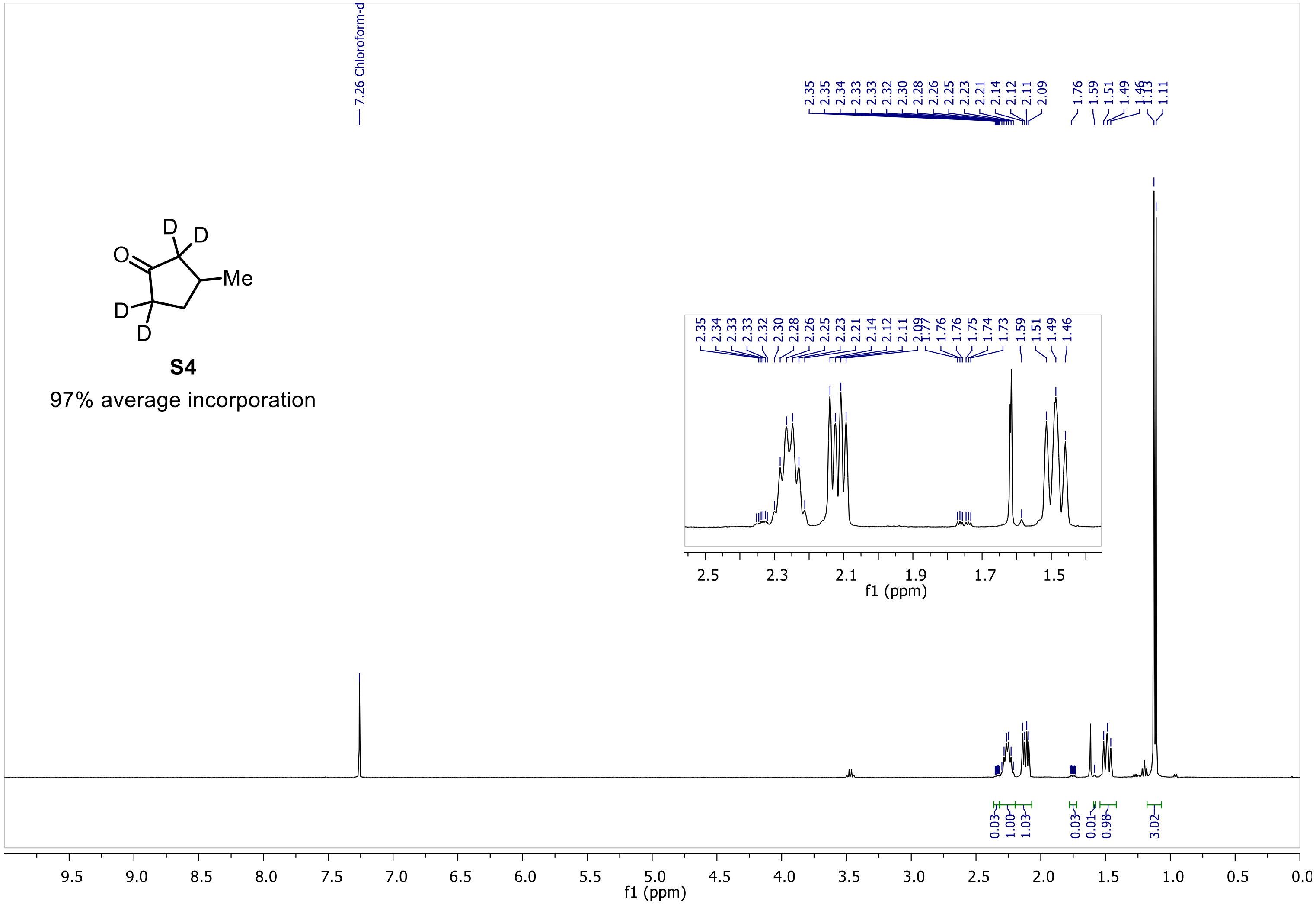

$^{13}C\{^1H\}$ NMR (101 MHz, $CDCl_3$):

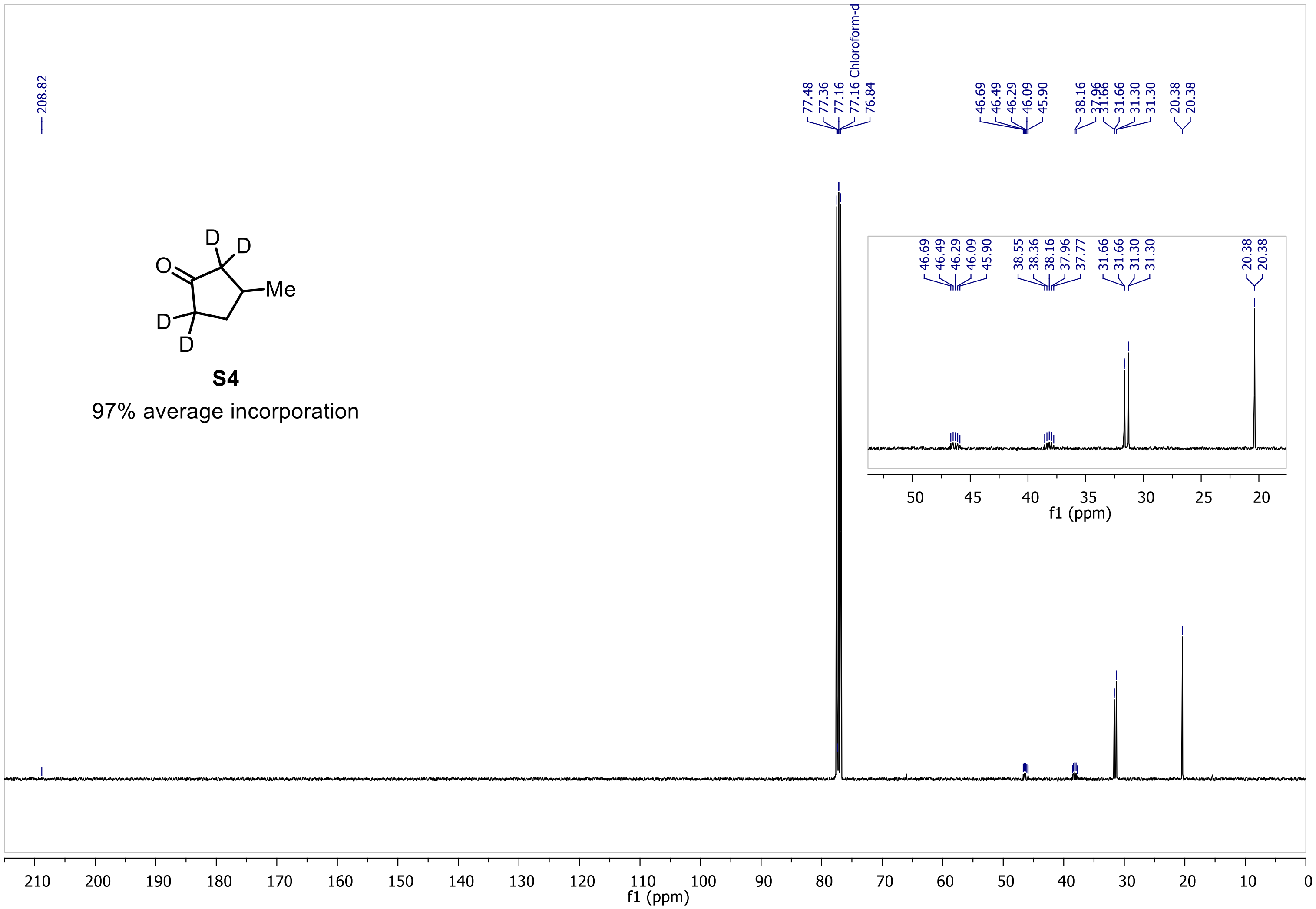

10 References (Manuscript 1-48, Supporting Information 49-81)

[1] T. Newhouse, P. S. Baran, *Angew. Chem. Int. Ed.* **2011**, *50*, 3362–3374.

[2] K. Godula, D. Sames, *Science* **2006**, *312*, 67–72.

[3] H. Sterckx, B. Morel, B. U. W. Maes, *Angew. Chem. Int. Ed.* **2019**, *58*, 7946–7970.

[4] A. Gunay, K. H. Theopold, *Chem. Rev.* **2010**, *110*, 1060–1081.

[5] P. Gandeepan, T. Müller, D. Zell, G. Cera, S. Warratz, L. Ackermann, *Chem. Rev.* **2019**, *119*, 2192–2452.

[6] M. Vijaykumar, B. Punji, *Synthesis* **2021**, *53*, 2935–2946.

[7] Z. Zhuang, T. Sheng, J. X. Qiao, K.-S. Yeung, J.-Q. Yu, *J. Am. Chem. Soc.* **2024**, DOI 10.1021/jacs.4c04043.

[8] Although transition metal catalyzed C–H activation has become a synthetic mainstay for functionalization of carbon hydrogen bonds (formally *via* organometallic species that can be seen as “masked” carbanions, C–H oxygenation methods *via* the metal-catalyzed C–H activation paradigm remain scarce, requiring harsh conditions to proceed and often facing issues of polyfunctionalization). For additional references, See: (a) M. Berger, R. Chauhan, C. A. B. Rodrigues, N. Maulide *Chem. – Eur. J.* **2016**, *22*, 16805–16808. (b) L. S. Fitzgerald, M. L. O’Duill *Chem. – Eur. J.* **2021**, *27*, 8411–8436. (c) F. Wang, X. Li, Z. Li, S. Zhou, W. Zhang *ACS Omega* **2019**, *4*, 331–343. (d) F.-J. Chen, S. Zhao, F. Hu, K. Chen, Q. Zhang, S.-Q. Zhang, B.-F. Shi *Chem. Sci.* **2013**, *4*, 4187–4192.

[9] S. J. Blanksby, G. B. Ellison, *Acc. Chem. Res.* **2003**, *36*, 255–263.

[10] M. Saito, Y. Kawamata, M. Meanwell, R. Navratil, D. Chiodi, E. Carlson, P. Hu, L. Chen, S. Udyavara, C. Kingston, M. Tanwar, S. Tyagi, B. P. McKillican, M. G. Gichinga, M. A. Schmidt, M. D. Eastgate, M. Lamberto, C. He, T. Tang, C. A. Malapit, M. S. Sigman, S. D. Minteer, M. Neurock, P. S. Baran, *J. Am. Chem. Soc.* **2021**, *143*, 7859–7867.

[11] While intramolecular 1,5-hydrogen atom transfer (1,5-HAT) is a highly regioselective process, there is a pronounced dearth of oxygenation protocols following this approach. For leading references, See: (a) L. M. Stateman, K. M. Nakafuku, D. A. Nagib *Synthesis* **2018**, *50*, 1569–1586. (b) E. M. Dauncey, S. P. Morcillo, J. J. Douglas, N. S. Sheikh, D. Leonori *Angew. Chem. Int. Ed.* **2018**, *57*, 744–748. (c) S. P. Morcillo, E. M. Dauncey, J. H. Kim, J. J. Douglas, N. S. Sheikh, D. Leonori *Angew. Chem. Int. Ed.* **2018**, *57*, 12945–12949. (d) V. Koch, S. Bräse *Eur. J. Org. Chem.* **2021**, 3478–3483.

[12] R. Mello, M. Fiorentino, C. Fusco, R. Curci, *J. Am. Chem. Soc.* **1989**, *111*, 6749–6757.

[13] G. Asensio, G. Castellano, R. Mello, M. E. González Núñez, *J. Org. Chem.* **1996**, *61*, 5564–5566.

[14] D. H.R. Barton, E. Csuhai, N. Ozbalik, *Tetrahedron* **1990**, *46*, 3743–3752.

[15] J. D. Griffin, D. B. Vogt, J. Du Bois, M. S. Sigman, *ACS Catal.* **2021**, *11*, 10479–10486.

[16] E. McNeill, J. D. Bois, *Chem. Sci.* **2012**, *3*, 1810–1813.

[17] M. S. Chen, M. C. White, *Science* **2007**, *318*, 783–787.

[18] N. A. Vermeulen, M. S. Chen, M. C. White, *Tetrahedron* **2009**, *65*, 3078–3084.

[19] J. Zhao, T. Nanjo, E. C. de Lucca, M. C. White, *Nat. Chem.* **2019**, *11*, 213–221.

[20] J. M. Howell, K. Feng, J. R. Clark, L. J. Trzepkowski, M. C. White, *J. Am. Chem. Soc.* **2015**, *137*, 14590–14593.

[21] M. S. Chen, M. C. White, *Science* **2010**, *327*, 566–571.

[22] Y. Kawamata, M. Yan, Z. Liu, D.-H. Bao, J. Chen, J. T. Starr, P. S. Baran, *J. Am. Chem. Soc.* **2017**, *139*, 7448–7451.

[23] G. Laudadio, S. Govaerts, Y. Wang, D. Ravelli, H. F. Koolman, M. Fagnoni, S. W. Djuric, T. Noël, *Angew. Chem. Int. Ed.* **2018**, *57*, 4078–4082.

[24] A. Call, G. Capocasa, A. Palone, L. Vicens, E. Aparicio, N. Choukairi Afailal, N. Siakavaras, M. E. López Saló, M. Bietti, M. Costas, *J. Am. Chem. Soc.* **2023**, *145*, 18094–18103.

[25] A. Palone, G. Casadevall, S. Ruiz-Barragan, A. Call, S. Osuna, M. Bietti, M. Costas, *J. Am. Chem. Soc.* **2023**, *145*, 15742–15753.

[26] V. Dantignana, M. Milan, O. Cussó, A. Company, M. Bietti, M. Costas, *ACS Cent. Sci.* **2017**, *3*, 1350–1358.

[27] J. P. Bégué, D. Bonnet, M. Charpentier-Morize, C. Pardo, *Tetrahedron* **1975**, *31*, 2505–2511.

[28] X. Creary, C. C. Geiger, *J. Am. Chem. Soc.* **1982**, *104*, 4151–4162.

[29] M. H. Lien, A. C. Hopkinson, *J. Am. Chem. Soc.* **1988**, *110*, 3788–3792.

[30] G. Carr, D. Whittaker, *J. Chem. Soc. Perkin Trans. 2* **1987**, 1877–1880.

[31] M. Artault, N. Mokhtari, T. Cantin, A. Martin-Mingot, S. Thibaudeau, *Chem. Commun.* **2020**, *56*, 5905–5908.

[32] I. S. Akhrem, D. V. Avetisyan, I. M. Churilova, L. V. Afanas’eva, O. I. Artyushin, N. D. Kagramanov, *Tetrahedron Lett.* **2013**, *54*, 6037–6040.

[33] I. Akhrem, A. Orlinkov, *Chem. Rev.* **2007**, *107*, 2037–2079.

[34] I. S. Akhrem, *J. Organomet. Chem.* **2015**, *793*, 54–77.

[35] R. Properzi, P. S. J. Kaib, M. Leutzsch, G. Pupo, R. Mitra, C. K. De, L. Song, P. R. Schreiner, B. List, *Nat. Chem.* **2020**, *12*, 1174–1179.

[36] M. A. Horwitz, A. B. Dürr, K. Afratis, Z. Chen, J. Soika, K. E. Christensen, M. Fushimi, R. S. Paton, V. Gouverneur, *J. Am. Chem. Soc.* **2023**, *145*, 9708–9717.

[37] B. R. Brutiu, G. Iannelli, M. Riomet, D. Kaiser, N. Maulide, *Nature* **2024**, *626*, 92–97.

[38] S. Arava, J. N. Kumar, S. Maksymenko, M. A. Iron, K. N. Parida, P. Fristrup, A. M. Szpilman, *Angew. Chem. Int. Ed.* **2017**, *56*, 2599–2603.

[39] S. Maksymenko, K. N. Parida, G. K. Pathe, A. A. More, Y. B. Lipisa, A. M. Szpilman, *Org. Lett.* **2017**, *19*, 6312–6315.

[40] K. N. Parida, G. K. Pathe, S. Maksymenko, A. M. Szpilman, *Beilstein J. Org. Chem.* **2018**, *14*, 992–997.

[41] J. P. Begue, M. Charpentier-Morize, *Acc. Chem. Res.* **1980**, *13*, 207–212.

[42] J. P. Begue, M. Charpentier-Morize, D. Bonnet-Delpon, J. Sansoulet, *J. Org. Chem.* **1980**, *45*, 3357–3359.

[43] V. V. Zhdankin, *ARKIVOC* **2009**, *2009*, 1–62.

[44] A. Yoshimura, V. V. Zhdankin, *Chem. Rev.* **2024**, DOI 10.1021/acs.chemrev.4c00303.

[45] V. V. Zhdankin, R. Tykwinski, R. Caple, B. Berglund, A. S. Koz'min, N. S. Zefirov, *Tetrahedron Lett.* **1988**, *29*, 3717–3720.

[46] V. V. Zhdankin, R. Tykwinski, B. Berglund, M. Mullikin, R. Caple, N. S. Zefirov, A. S. Koz'min, *J. Org. Chem.* **1989**, *54*, 2609–2612.

[47] The formation of two regioisomeric alcohols, which contrasts the method reported in [37], which would have delivered isomers differing in the position of the acyl substituent, opens the question of directionality of charge relocation. This will be further discussed below.

[48] J. P. Begue, *J. Org. Chem.* **1982**, *47*, 4268–4271.

[49] A. Bauer, G. Di Mauro, J. Li, N. Maulide, *Angew. Chem. Int. Ed.* **2020**, *59*, 18208–18212.

[50] B. S. Martins, D. Kaiser, A. Bauer, I. Tiefenbrunner, N. Maulide, *Org. Lett.* **2011**, *23*, 2094–2098.

[51] B. E. Love, E. G. Jones, *J. Org. Chem.* **1999**, *64*, 3755–3756.

[52] D. M. Allwood, D. C. Blakemore, S. V. Ley, *Org. Lett.* **2014**, *16*, 3064–3067.

[53] J. M. White, A. R. Tunoori, B. J. Turunen, G. I. Georg, *J. Org. Chem.* **2004**, *69*, 2573–2576.

[54] T. Niu *et al.*, *Org. Lett.* **2009**, *11*, 4474–4477.

[55] T. J. Nitz, D. L. Volkots, D. J. Aldous, R. C. Oglesby, *J. Org. Chem.* **1994**, *59*, 5828–5832.

[56] R. K. Dieter, M. Tokles, *J. Am. Chem. Soc.* **1987**, *109*, 2040–2046.

[57] T. Katsina, K. E. Papoulidou, A. L. Zografos, *Org. Lett.* **2019**, *21*, 8110–8115.

[58] M. Suzuki, T. Suzuki, T. Kawagishi, Y. Morita, R. Noyori, *Isr. J. Chem.* **1984**, *24*, 118–124.

[59] C. Petrier, J.-L. Luche, *Tetrahedron Lett.* **1987**, *28*, 2347–2350.

[60] Z. Li, V. Gevorgyan, *Angew. Chem. Int. Ed.* **2011**, *50*, 2808–2810.

[61] X. Xi, *et al.*, *Angew. Chem. Int. Ed.* **2022**, *61*, e202114731.

[62] X. Zhang, Z. Wang, X. Fan, J. Wang, *J. Org. Chem.* **2015**, *80*, 10660–10667.

[63] C. D. Aretz, H. Escobedo, B. J. Cowen, *Eur. J. Org. Chem.* **2018**, *2018*, 1880–1884.

[64] Z. Zheng, *et al.*, *Org. Biomol. Chem.* **2018**, *16*, 8020–8024.

[65] M. Mato, C. García-Morales, A. M. Echavarren, *ACS Catal.* **2020**, *10*, 3564–3570.

[66] Xiao, H.-Q., Shu, X.-Z., Ji, K.-G., Qi, C.-Z. & Liang, Y.-M.. Catal. Commun. **2009**, *10*, 1824–1827.

[67] Y. Hu, *et al.*, *Angew. Chem. Int. Ed.* **2019**, *58*, 15813–15818.

[68] V. V. Zhdankin, *et al.*, *J. Org. Chem.* **1989**, *54*, 2609–2612.

[69] A. Watanabe, K. Miyamoto, T. Okada, T., Asawa, M. Uchiyama, *J. Org. Chem.* **2018**, *83*, 14262–14268.

[70] A. McKillop, D. Kemp, *Tetrahedron* **1989**, *45*, 3299–3306.

[71] M. S. Chen, M. C. White, *Science* **2010**, *327*, 566–571.

[72] M. D. Vu, M. Das, X.-W. Liu, *Chem. – Eur. J.* **2017**, *23*, 15899–15902.

[73] F. Wu, W. Lu, Q. Qian, Q. Ren, H. Gong, *Org. Lett.* **2012**, *14*, 3044–3047.

[74] K. Sato *et al.*, *Org. Lett.* **2008**, *10*, 2405–2408.

[75] F. Mo, G. Dong, *Science* **2014**, *345*, 68–72.

[76] Version 1.37.0.0 (STOE, 2021).

[77] Version 1.31.186.0 (STOE, 2022).

[78] O. V. Dolomanov, L. J. Bourhis, R. J. Gildea, J. A. K. Howard, H. Puschmann, *J. Appl. Crystallogr.* **2009**, *42*, 339–341.

[79] C. B. Hübschle, G. M. Sheldrick, B. Dittrich, *J. Appl. Crystallogr.* **2011**, *44*, 1281–1284.

[80] G. M. Sheldrick (2015). SHELXS v 2016/4 University of Göttingen, Germany.

[81] A. L. Spek, *Acta Crystallogr. D Biol. Crystallogr.* **2009**, *65*, 148–155.
